# Low expression of the dynamic network markers *FOS*/*JUN* in pre-deteriorated epithelial cells is associated with the progression of colorectal adenoma to carcinoma

**DOI:** 10.1186/s12967-023-03890-5

**Published:** 2023-01-25

**Authors:** Xiaoqi Huang, Chongyin Han, Jiayuan Zhong, Jiaqi Hu, Yabin Jin, Qinqin Zhang, Wei Luo, Rui Liu, Fei Ling

**Affiliations:** 1 Guangdong Key Laboratory of Fermentation and Enzyme Engineering, School of Biology and Biological Engineering, South China University of Technology, Guangzhou, 510006 China; 2 School of Mathematics, South China University of Technology, Guangzhou, 510641 China; 3 Institute of Clinical Research, The First People’s Hospital of Foshan, Foshan, 528000 China; 4 Pazhou Lab, Guangzhou, 510330 China

**Keywords:** Colorectal cancer, Dynamic network biomarker, Epithelial cell deterioration, Pre-deteriorated epithelial cell, *FOS/JUN*, *P53*

## Abstract

**Background:**

Deterioration of normal intestinal epithelial cells is crucial for colorectal tumorigenesis. However, the process of epithelial cell deterioration and molecular networks that contribute to this process remain unclear.

**Methods:**

Single-cell data and clinical information were downloaded from the Gene Expression Omnibus (GEO) database. We used the recently proposed dynamic network biomarker (DNB) method to identify the critical stage of epithelial cell deterioration. Data analysis and visualization were performed using R and Cytoscape software. In addition, Single-Cell rEgulatory Network Inference and Clustering (SCENIC) analysis was used to identify potential transcription factors, and CellChat analysis was conducted to evaluate possible interactions among cell populations. Gene Ontology (GO), Kyoto Encyclopedia of Genes and Genomes (KEGG), and gene set variation analysis (GSVA) analyses were also performed.

**Results:**

The trajectory of epithelial cell deterioration in adenoma to carcinoma progression was delineated, and the subpopulation of pre-deteriorated epithelial cells during colorectal cancer (CRC) initialization was identified at the single-cell level. Additionally, *FOS*/*JUN* were identified as biomarkers for pre-deteriorated epithelial cell subpopulations in CRC. Notably, *FOS*/*JUN* triggered low expression of P53-regulated downstream pro-apoptotic genes and high expression of anti-apoptotic genes through suppression of *P53* expression, which in turn inhibited P53-induced apoptosis. Furthermore, malignant epithelial cells contributed to the progression of pre-deteriorated epithelial cells through the GDF signaling pathway.

**Conclusions:**

We demonstrated the trajectory of epithelial cell deterioration and used DNB to characterize pre-deteriorated epithelial cells at the single-cell level. The expression of DNB-neighboring genes and cellular communication were triggered by DNB genes, which may be involved in epithelial cell deterioration. The DNB genes *FOS/JUN* provide new insights into early intervention in CRC.

**Supplementary Information:**

The online version contains supplementary material available at 10.1186/s12967-023-03890-5.

## Background

Colorectal cancer (CRC) is the third most frequent disease and second leading cause of cancer-related fatalities globally [1]. The genomic and transcriptomic landscapes of familial adenomatous polyposis have been delineated at the single-cell level [2]. In addition, previous study has mapped the single-cell resolution of colorectal adenomas and serrated polyps [3]. Joanito et al. identified two epithelial tumor cell states based on single-cell and bulk transcriptomic analysis and further refined the consensus molecular classification of CRC [4]. In vitro colorectal cancer organoid culture systems were systematically evaluated at the single-cell scale [5]. Teng et al. revealed the molecular basis of the impact of gut microbes on the efficacy of neoadjuvant radiotherapy in locally advanced rectal cancer based on host-bacterial colony interactions [6]. Based on clinical studies, early detection is necessary for timely intervention in patients with CRC. However, most studies have concentrated on the analysis of advanced-stage tumors [7–9] and have largely ignored precancerous lesions. Consequently, the transition process from the precancerous to cancerous state and the molecular drivers of this transition remain underexplored. Deterioration of intestinal epithelial cells is crucial for colorectal tumorigenesis. It has been demonstrated that a variety of variables, such as genetic mutations, growth factors, and cytokines, contribute to the deterioration of epithelial cells [10]. In addition, the hypothesis that a population of carcinoma precursor epithelial cells exists during epithelial cell deterioration has been proposed [11]. However, the process of epithelial cell deterioration in CRC remains unclear. Therefore, if pre-deteriorated epithelial cells and regulatory molecular networks can be found during CRC epithelial cell deterioration to intervene with the development of the adenoma-carcinoma sequence, this may be a breakthrough in preventing the early occurrence of CRC.

Dynamic network biomarkers (DNBs), which are a small group of closely connected variables that can be used to provide early warning signals of impending critical transitions through drastic statistical indicators, offer a statistical method for assessing variables related to critical states [12–14]. DNBs are a group of biomolecules that can signal critical states prior to the rapid deterioration of complex diseases. The DNB method has previously been applied in CRC research. For example, Hu discovered a subpopulation of pre-exhausted CD8 + T cells based on DNB and single-cell data, which contributes to T cell exhaustion in CRC. The main causes of T cell exhaustion were found to be the hub genes *CCT6A* and *TUBA1B* [15]. According to single-cell analysis of CRC adjacent tissue B cells, stage II was a crucial stage before lymph node metastasis, and the *DHX9* gene participated in dynamic network changes during CRC development [16]. In addition, several research teams have applied the DNB method to examine lung metastasis in hepatocellular carcinoma, as well as irreversible alterations during cell differentiation [17, 18]. Currently, single-cell RNA sequencing (scRNA-seq) is a potent method to address the heterogeneity of epithelial cells in the CRC microenvironment. By combining scRNA-seq and DNB, we may be able to identify pre-deteriorated epithelial cells and essential functional molecular networks in the CRC microenvironment.

In this study, we aimed to clarify the mechanism underlying epithelial cell deterioration in CRC and to identify pertinent targets and biomarkers in pre-deteriorated epithelial cells. To identify biomarkers of pre-deteriorated epithelial cells in CRC, we constructed an epithelial cell deterioration trajectory in CRC using the scRNA-seq dataset (GSE161277) supplied by the research team of Hubing Shi [11, 19]. We also evaluated gene network modifications in epithelial cells during deterioration using the DNB method, and interpreted the roles of these genes in terms of networks. Finally, we investigated the cellular interactions between pre-deteriorated and malignant epithelial cells. In summary, this study not only succeeded in identifying a subpopulation of pre-deteriorated epithelial cells during CRC initialization but also discovered a core molecular network that plays a critical role in this subpopulation. We hope that these findings will provide novel biomarkers or useful targets for early CRC intervention.

## Methods

### Theoretical basis

**Fig. 1:**
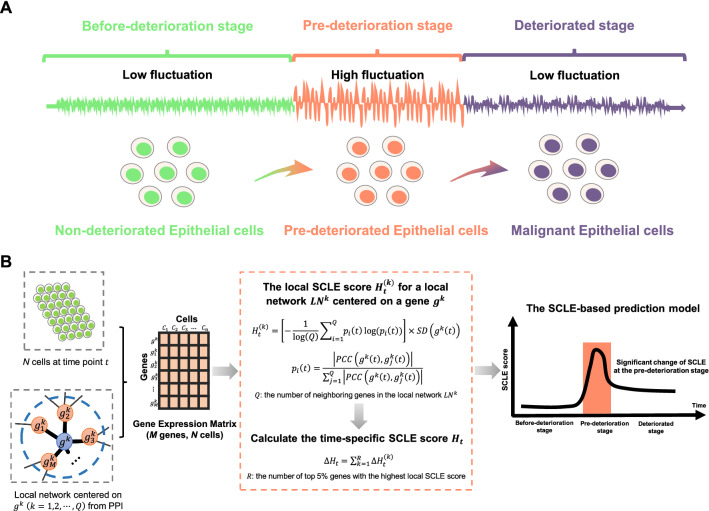
Overall project design together with algorithm details. **A** The three stages transition of PPI network during epithelial cell deterioration progression in classic dynamic network biomarker theory. **B** Single-cell landscape entropy algorithm

We developed a single-cell landscape entropy (SCLE) method to detect a critical state before a critical transition from a relatively normal state to a deteriorated state (Fig. 1A). A group of molecules known as dynamic network biomarker (DNB) biomolecules exists and satisfies the following three properties:

1. The standard deviation (*SD*_*in*_) for genes in the DNB group increases drastically;
2. The Pearson’s correlation coefficient (*PCC*_*in*_) for genes in the DNB group increases drastically;
3. The Pearson’s correlation coefficient (*PCC*_*out*_) between any one member in the DNB group and any other non-DNB member decreases rapidly;

These three properties are necessary for phase transition in biological systems. Clearly, the early-warning signals of the critical transition in a system are detected through quantifying the perturbation in the local networks of some drastically fluctuating variables.

The time period can be viewed as a pseudotime that reflects the process of epithelial cell deterioration. Based on various intracellular gene expression patterns, the deterioration trajectory of epithelial cells is divided into six subpopulations, each of which is considered as a time point.

### Algorithm for identifying the signal of critical transition based on single-cell landscape entropy (SCLE)

Based on the time series of scRNA-seq data, the following algorithm was used to predict critical transition (Fig. 1B):

- **[Step 1]** Normalize scRNA-seq data. The original gene expression matrix with M rows/genes and N columns/cells was normalized using the logarithm $\mathrm{log}\left(1+x\right)$ at each time point.
- **[Step 2]**Define the global template network ${N}^{G}$. The global template network ${N}^{G}$ was constructed by mapping genes to a protein–protein interaction (PPI) network obtained from the STRING database, with all isolated nodes discarded.
- **[Step 3]**Extract each local network from the global template network ${N}^{G}$. Specifically, there are *M* genes in the global template network ${N}^{G}$ corresponding to *M* local networks ${LN}^{k}$(*k* = 1, 2, 3, …, *M*). The local network ${LN}^{k}$ is centered on gene ${g}^{k}$, with its first-order neighbors {${g}_{1}^{k}$, ${g}_{2}^{k}$, …, ${g}_{Q}^{k}$} serving as edges.
- **[Step 4]**Calculate the gene-specific local SCLE score ${H}_{t}^{(k)}$ for each local network at a time point *t*. The associated local SCLE score for a local network ${LN}^{k}$ centered on a gene ${g}^{k}$ was obtained as follows:
  1 $${H}_{t}^{(k)} =[-\frac{1}{\mathrm{log}\left(Q\right)}\sum_{i=1}^{Q}{p}_{i}(t)\mathrm{log}({p}_{i}(t))]\times SD({g}^{k}\left(t\right))$$
  with
  2 $${p}_{i}\left(t\right)=\frac{|PCC({g}^{k}\left(t\right),{g}_{j }^{k}\left(t\right))|}{\sum_{j=1}^{Q}|PCC\left({g}^{k}\left(t\right),{g}_{j }^{k}\left(t\right)\right)|}$$
  where *SD*(${g}^{k}\left(t\right)$) denotes the standard deviations of the central gene ${g}^{k}$ at a time point *t* and *PCC*(${g}^{k}\left(t\right)$,${g}_{j}^{k}\left(t\right)$) denotes the Pearson’s correlation coefficient between the central gene ${g}^{k}$ and a neighboring gene ${g}_{j}^{k}$ at a time point *t*. The constant $Q$ is the number of neighboring genes in the local network ${LN}^{k}$.
- **[Step 5]**Calculate the time-specific SCLE score ${H}_{t}$ based on a set of genes with the greatest local SCLE values, that is:
  3 $${H}_{t}={\sum }_{k=1}^{R}{H}_{t}^{(k)},$$
  where the constant *R*, representing the number of top 5% of genes with the highest local SCLE value, is an adjustable parameter. ${H}_{t}$, the SCLE score of time point *t* in Eq. 3, can be applied to identify the early warning signals for the critical transition. At each time point, the SCLE value of a particular cell population is employed as the time-specific SCLE score to identify the critical point.
  As the system approaches the vicinity of the critical point, the DNB molecules exhibit fluctuating collective behavior, causing the dependent properties of the DNB members in the critical state to differ from those in the before-transition state. Moreover, the local SCLE value ${H}_{t}^{\left(k\right)}$ in Eq. 1 increases sharply as the system approaches the critical point (Fig. 1B).

### Data processing

The scRNA-seq data used for this research were obtained from the Gene Expression Omnibus (GEO) database with accession number GSE161277 [11]. We processed the scRNA-seq data using *Seurat* (version 4.1.1) pipelines [20]. Owing to biological differences between tissues, we removed batch effects from patients using the R package *Harmony* (version 0.1.0) [21]. The resolution parameter of *FindClusters* function was set at 1 for all cell types and 0.6 for the epithelial cell subpopulation.

### Copy number variants (CNV) analysis

The R package *infercnv* (version 1.10.1) was used to calculate CNVs in epithelial cells and to identify malignant cells using default parameters. Epithelial cells from normal tissues were used as the controls.

### Trajectory analysis

The R package *Monocle* (version 2.22.0) [19] was implemented to infer the epithelial cell deterioration trajectory. For trajectory inference, differentially expressed genes (DEGs) of the malignant cell subpopulation were used as ordering genes. We then obtained the epithelial cell deterioration trajectory after dimension reduction and cell ordering with the ordered genes.

### DEG identification and functional enrichment analysis

The *FindMarkers* function in Seurat (version 4.1.1) [20] was used to identify DEGs for each cluster. Gene Ontology (GO) and Kyoto Encyclopedia of Genes and Genomes (KEGG) pathway enrichment analyses were performed using the *clusterProfiler* package (version 4.2.2) [22]. The R package Gene Set Variation Analysis (*GSVA*; version 1.42.0) [23] was used for functional enrichment analysis.

### Protein–protein interaction network analysis

The PPI network of DNB genes was constructed using the STRING database (version 11.5) [24]. We exported the adjacency matrix by visualizing it in *Cytoscape* (version 3.9.1) [25], calculating the degree of each gene using the *CytoHubba* plugin, and selecting the top 50 genes for visualization.

### Cell–cell interaction and single-cell regulatory network inference and clustering (SCENIC) analyses

Cell–cell interaction analysis was performed using *CellChat* (version 1.5.0) [26]. We evaluated the possible interactions among the cell populations based on the ligand-receptor pair data in CellChatDB. Using the R package *SCENIC* (version 1.3.1) [27], SCENIC analysis was used to identify potential transcription factors in cells on the pseudotime trajectory and to analyze their transcriptional activity.

### Statistical analysis

Statistical analysis and visualization were conducted and implemented using R software (version 4.1.2), and the statistical threshold for significance was set at p < 0.05. The detailed code is available from the link of GitHub (https://github.com/Katherine776666/CRC_Epi_DNB).

## Results

### Identification of epithelial cell subtypes and their deterioration trajectory

**Fig. 2:**
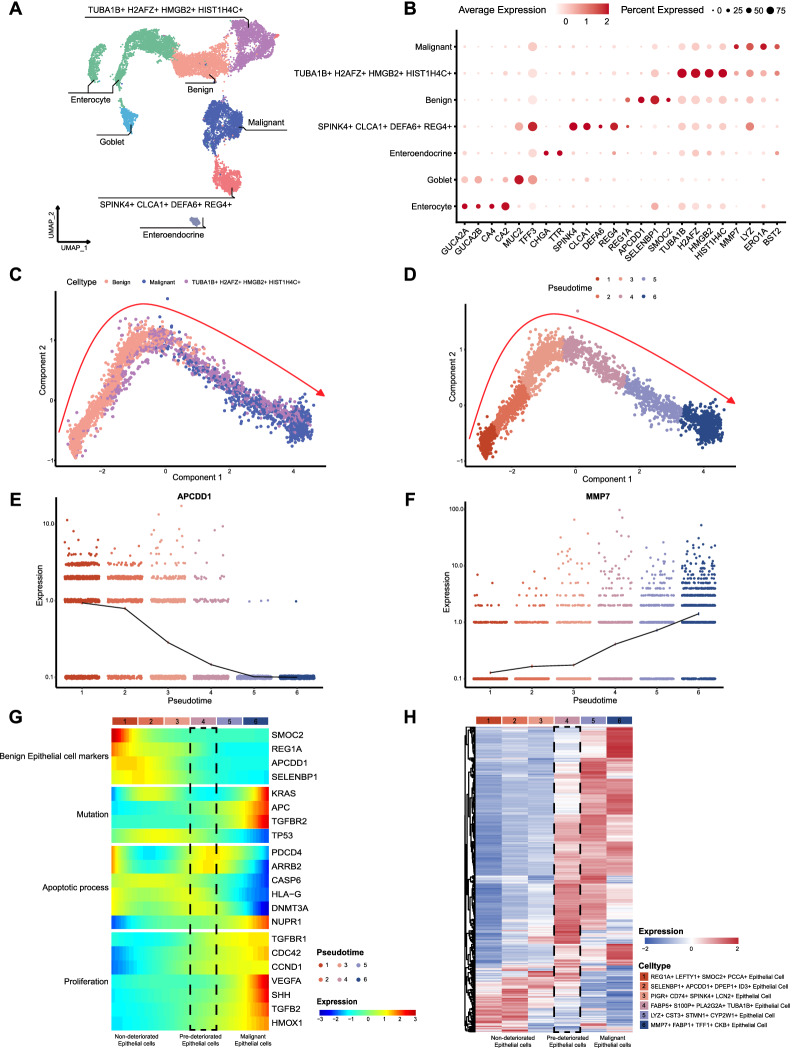
Trajectory of epithelial cell deterioration. **A** UMAP clustering of epithelial cells (n = 11,635) from scRNA-seq of patients with CRC. **B** Expression of marker genes in seven clusters of epithelial cells. **C**, **D** Potential trajectory of epithelial cell deterioration in adenoma and carcinoma tissues (n = 3688) inferred using Monocle2 based on gene expression. Pseudotime is shown numbered 1- 6 and the red dash indicates the direction of the pseudotime. **E**, **F** The expression levels of *APCDD1* and *MMP7* in different pseudotime of epithelial cell subpopulation. The average gene expression is indicated by the black dash. **G** The heatmap shows dynamic changes in gene expression, including benign epithelial markers, mutant genes associated with epithelial cell deterioration, epithelial apoptotic processes, and cell proliferation. **H** The heatmap shows expression of cell type-specific gene markers in different epithelial cell clusters. Marker genes for each cluster identified by Seurat analysis, with four genes selected for each cluster highlighted at the top

Strict quality control standards were implemented to screen the processed scRNA-seq data in the original article, and Uniform Manifold Approximation and Projection (UMAP) was performed on the cell populations in normal, adenoma, and carcinoma tissues for visualization (Additional file 1: Fig. S1A). Based on canonical markers for known cell lineages, the identified clusters were annotated as biological cell types (Additional file 1: Fig. S1B and Additional file 8: Table S1), epithelial cells (*EPCAM*), T cells (*CD3D*), Follicular B cells (*MS4A1*), Plasma B cells (*MZB1*), Macrophages (*CD68*), and Fibroblasts (*DCN*). To better investigate colorectal carcinogenesis, we extracted 11,635 labeled epithelial cell samples from three tissues for further analysis. InferCNV analysis was then performed on epithelial cells to identify malignant epithelial cells (Additional file 2: Fig. S2). Based on the results of the inferCNV analysis, epithelial pathological genetic markers [28] and canonical colorectal epithelial markers, 11,635 epithelial cells were reclustered into seven subpopulations (Fig. 2A, B and Additional file 9: Table S2).

To better understand the molecular mechanisms of colorectal carcinogenesis, we constructed the deterioration trajectory of epithelial cells based on three subpopulations of epithelial cells: benign, TUBA1B + H2AFZ + HMGB2 + HIST1H4C + , and malignant cells. Based on various intracellular gene expression patterns, epithelial cell deterioration was divided into six clusters (Fig. 2C, D). *APCDD1*, *REG1A*, and *SELENBP1* were significantly highly expressed in the epithelial cell subpopulation at the beginning of the differentiation trajectory (Fig. 2E and Additional file 1: Fig. S1C, D). As epithelial cell deterioration had not yet begun, the expression of benign epithelial cell markers was high, whereas that of malignant epithelial cell markers shown in Fig. 2B was low (Fig. 2E, F and Additional file 1: Fig. S1C–F). During the deterioration process, non-deteriorated, pre-deteriorated, and malignant epithelial cell subpopulations have unique characteristics. In addition to the increased expression of mutated genes with the deterioration of epithelial cells, key functional genes in epithelial cells were also altered (Fig. 2G). With the exception of *NUPR1*, low expression of epithelial cell apoptotic genes was associated with the deterioration of epithelial cells. Additionally, the expression of *NUPR1* increased with tumor aggressiveness [29]. Malignant epithelial cells showed elevated *NUPR1* expression, which may have boosted tumor aggressiveness. Furthermore, cell proliferation genes were highly expressed in malignant epithelial cells, suggesting that the terminal subpopulations were active in response to additional proliferative signals (Fig. 2G).

To identify the specific cell populations responsible for deterioration, we conducted a DEG analysis on six epithelial cell subpopulations. At the beginning of the deterioration trajectory, we identified the epithelial cell subpopulation as REG1A + LEFTY1 + SMOC2 + PCCA + epithelial cell. It is possible that this subpopulation will deteriorate. Typical defensive, secretory, and absorptive capabilities were still present, with high expression of genes that characterize benign epithelial cells (Fig. 2E and Additional file 1: Fig. S1C, D). In contrast, the epithelial cell subpopulation at the end of the deterioration trajectory highly expressed malignant epithelial cell marker genes, including *MMP7* and *ERO1A* (Fig. 2F, Additional file 1: Fig. S1E and Additional file 10: Table S3). Additionally, we found that malignant epithelial cell subpopulations were different. Malignant epithelial cells were identified as LYZ + CST3 + STMN1 + CYP2W1 + epithelial cell and MMP7 + FABP1 + TFF1 + CKB + epithelial cell (Fig. 2H). Finally, we investigated the trajectory of epithelial cell deterioration in CRC adenomas and carcinomas.

### Identification of pre-deteriorated epithelial cell subpopulation in colorectal tumorigenesis

**Fig. 3:**
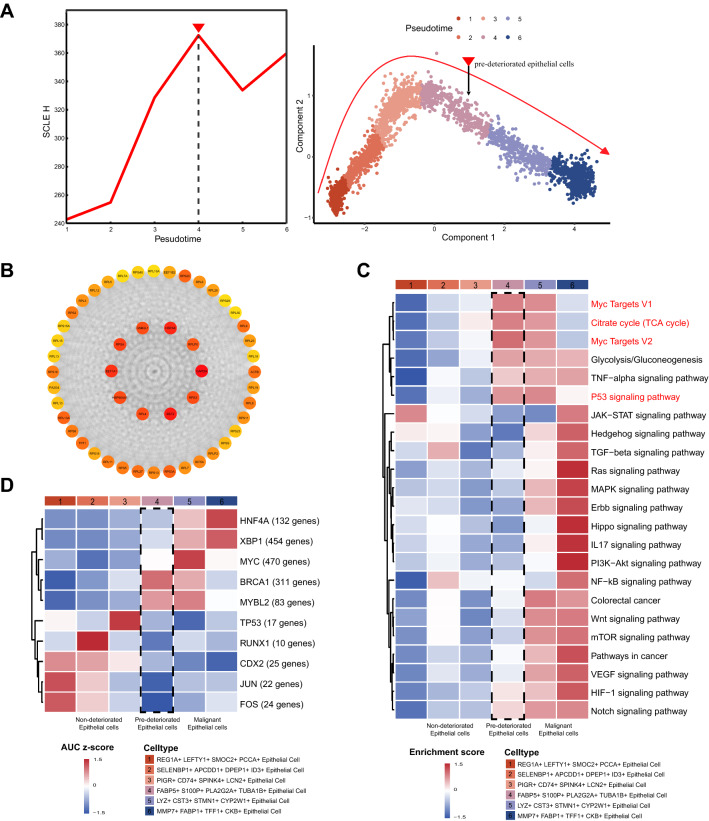
Identification of epithelial cell subtype. **A** The graph on the left demonstrates that the curve of SCLE score ${H}_{t}$ defined in *Materials and Methods* suddenly increases as the system approaches the critical point (p = 0), which is considered as a critical state transition at a bifurcation point. The graph on the right demonstrates that pre-deteriorated epithelial cells were in the critical state and potential trajectory of epithelial cell deterioration is same as Fig. 2D. **B** The graph shows the PPI network of DNB core genes. **C** Function analysis (GSVA) demonstrates that various gene sets may have varying effects on the progression of epithelial cell deterioration. Pathways that were significantly enriched in the pre-deterioration stage are marked in red. Limma was used to compare enrichment score between before-deterioration stage and pre-deterioration stage, as well as between pre-deterioration stage and deteriorated stage. **D** The heatmap demonstrates the TF regulatory activity (AUC z-score) estimated using SCENIC analysis. The five TFs with increased transcriptional activity were chosen for visualization in before-deterioration stage and deteriorated stage, respectively. Limma was used to compare AUC discrepancies between before-deterioration stage and pre-deterioration stage, as well as between pre-deterioration stage and deteriorated stage

Using the DNB approach, we discovered a subpopulation of pre-deteriorated epithelial cells with a strong signal of the critical state prior to epithelial cell deterioration, as shown by the considerable shift in single-cell landscape entropy (SCLE) in the fourth period (Fig. 3A). We identified the fourth-period subpopulation as FABP5 + S100P + PLA2G2A + TUBA1B + epithelial cells. Notably, *S100P*, the pre-deteriorated marker, was both a DNB gene and a DEG for these pre-deteriorated epithelial cells, demonstrating that this cell subpopulation had already begun to exhibit deterioration characteristics and can be defined as pre-deteriorated epithelial cells. In total, 260 DNB genes were identified in this study. In the critical state, the DNB module genes fluctuated drastically, with a wide deviation in gene expression and a strong association within the module. Therefore, we constructed a network for DNB core genes, such as *GAPDH*, *EEF2*, *HSPA8* and *EEF1A1*, which ranked highly in the network in terms of molecular degree and may be crucial for the deterioration of epithelial cells (Fig. 3B and Additional file 3: Fig. S3A-D). Based on GO enrichment analysis, DNB genes were enriched in several regulatory pathways related to proliferation or apoptosis, such as epithelial cell proliferation, apoptotic signaling pathways, signal transduction by p53-like mediators, and NIK/NF-kappaB signaling (Additional file 4: Fig. S4A).

To further discover functional changes in DNB genes in pre-deteriorated epithelial cells as defined by DNB, GSVA analysis was applied to these epithelial cells. The results showed that the p53 signaling pathway was considerably enriched in pre-deteriorated epithelial cells (Fig. 3C), which is consistent with the results of other study [11]. Furthermore, after the DNB-derived pre-deterioration period, oncogenic signaling pathways such as JAK-STAT, Wnt, PI3K-Akt, VEGF, and TGF-β were significantly altered. In addition, the citrate cycle (TCA cycle) and glycolysis/gluconeogenesis were downregulated in the early stages of the proposed chronotropic trajectory, and then upregulated at the end of the proposed chronotropic trajectory (Fig. 3C). This result is consistent with previous studies showing that malignant cells primarily use glycolysis rather than the TCA pathway to generate energy and intermediate precursors for metabolite biosynthesis, a phenomenon known as the Warburg effect [2, 30]. Consequently, we identified a subpopulation of pre-deteriorated epithelial cells with unique gene expression patterns and functional transition states.

We further performed SCENIC analysis of these epithelial cells to investigate the transcription factors that may play a regulatory role in the deterioration of epithelial cells. Specific co-expressed TFs and their potential target genes were identified (Fig. 3D and Additional file 5: Fig. S5). The results revealed a significant difference in the regulatory activity of the TFs obtained from screening before and after the critical period. Apoptosis-induction-related TFs [31] such as *FOS*, *JUN*, and *TP53* exhibited significantly higher transcriptional activity in before-deteriorated cell populations than in deteriorated cell populations. Notably, *FOS* and *JUN* form AP-1 transcription factor dimers that affect cell life and death by regulating the expression and transcriptional activity of the tumor suppressor gene, *TP53* [32]. In contrast, the upregulation of the transcriptional activity of *MYC*, *MYBL2*, and *XBP1* is associated with cell proliferation [33–35], which may contribute to the progression of pre-deteriorated epithelial cells toward deterioration.

### Low expression of *P53* triggered by *FOS/JUN* suppressed P53-induced apoptosis in pre-deteriorated epithelial cells, contributing to epithelial cell deterioration

**Fig. 4:**
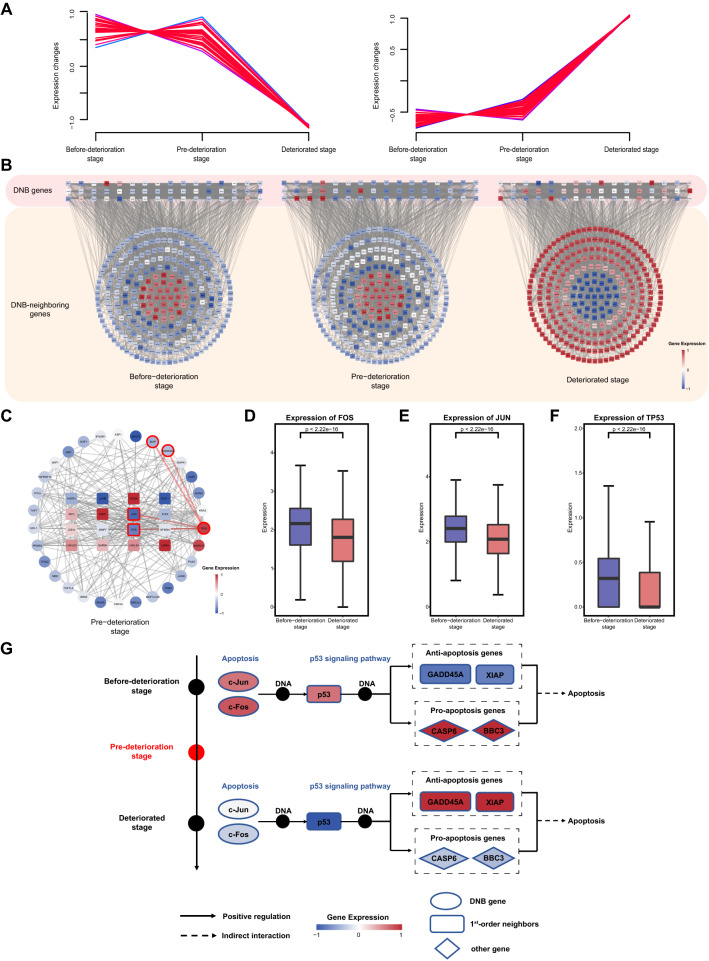
The reversed expression of DNB-neighboring genes is driven by DNB genes. **A** The series of graphs illustrates the pattern of dynamic changes in DNB neighboring genes between Pre-deterioration stage and Deteriorated stage using MFUZZ. **B** Cytoscape visualization of the interaction network between the DNB genes and their neighboring genes, including three stages of epithelial cell deterioration. The DNB genes are represented by square-shaped network nodes from the pink region. The DNB-neighboring genes are represented by the network nodes grouped in circles from the orange region. All three types of deterioration states involve the same genes and their locations in the network, and a gradient from blue to red denotes low to high levels of gene expression. **C** Cytoscape visualization of DNB genes and their neighboring genes interaction network in the pre-deterioration stage. Rectangles represent the DNB genes, and circles represent the DNB-neighboring genes. The gradient from blue to red denotes low to high levels of gene expression. **D**–**F** The expression of *FOS*, *JUN* and *TP53* in before-deterioration stage and deteriorated stage. Differences in expression are checked using the Wilcox test. **G** *FOS* and *JUN* regulated the expression of *P53* and its downstream apoptosis-related genes to suppress P53-induced apoptosis. The oval represents DNB genes, the rectangle represents DNB-neighboring genes, and the diamond represents additional genes that do not belong to any category. The gradient from blue to red denotes low to high levels of gene expression

We used a soft clustering algorithm to categorize DNB-neighboring genes based on their expression trends and discovered drastic changes in the gene expression levels of the epithelial cell subpopulation between the critical state and after the critical state (Fig. 4A). Furthermore, the critical period detected using the DNB method was considered the period of pre-deteriorated epithelial cells (Fig. 3A). Once the critical period is complete, these cells become malignant epithelial cells.

To systematically explore the roles of DNB genes in the pre-deteriorated epithelial cell subpopulation, we constructed a PPI network for DNB and DNB-neighboring genes. We found that the expression of DNB-neighboring genes showed flip-flop changes after the critical period (Fig. 4B). Therefore, these DNB-neighboring genes are regarded as reversed genes that play a significant role in epithelial cell deterioration. DNB genes also interact with reversed genes in the network, which may change gene expression patterns or regulate downstream interactions. Remarkably, these DNB-neighboring genes were enriched in pathways such as colorectal cancer, apoptosis, and metabolism-related pathways (Additional file 4: Fig. S4B). Subsequently, we mapped DNB genes and DNB-neighboring genes to these pathways and observed a regulatory role of DNB genes on DNB-neighboring genes in the apoptosis pathway. Therefore, we selected this section for further analysis.

Furthermore, we constructed a subnetwork of DNB genes and DNB-neighboring genes mapped to the apoptotic pathway. These DNB-neighboring genes are driven by DNB genes. We discovered that *FOS* and *JUN* act as DNB genes to drive the reversal alterations of *P53* expression (Fig. 4C and Additional file 6: Fig. S6A). We observed that the expression of the DNB genes *FOS* and *JUN* significantly decreased with epithelial cell deterioration (Fig. 4D, E), which is consistent with a previous study [36]. Additionally, the expression of *P53*, a DNB-neighboring gene in pre-deteriorated epithelial cells, significantly decreased with epithelial cell deterioration (Fig. 4F). Subsequently, we selected four apoptosis-related genes with known functions (two pro-apoptotic and two anti-apoptotic) and analyzed their gene expression to identify apoptotic trends during epithelial cell deterioration. The results demonstrated that during the deterioration of epithelial cells, the expression of pro-apoptotic genes, such as *BBC3* and *CASP6*, dramatically decreased, whereas the expression of anti-apoptotic genes, such as *GADD45A* and *XIAP*, significantly increased (Additional file 6: Fig. S6B). This suggests that apoptosis was inhibited during epithelial cell deterioration. SCENIC analysis demonstrated that the transcriptional activities of *FOS* and *JUN* had similar expression patterns (Fig. 3D), suggesting that they act synergistically. *JUN* and *FOS* are proto-oncogenes with expression products that can dimerize to form the activator protein-1 (AP-1) complex [37, 38], which is involved in tumorigenesis by regulating the expression and transcriptional activity of the target gene *P53* to affect apoptosis [32]. In our gene regulatory role analysis, *FOS* and *JUN* formed the AP-1 complex that regulates the expression of downstream apoptosis-related genes of the P53 pathway by inhibiting the expression of *P53*, contributing to the suppression of P53-dependent apoptosis (Fig. 4G). Consequently, *FOS* and *Jun* drive low expression of *P53* to suppress P53-induced apoptosis, which may contribute to epithelial cell deterioration.

### Malignant epithelial cells contributed to the progression of pre-deteriorated epithelial cells toward deterioration through the GDF signaling pathway

**Fig. 5:**
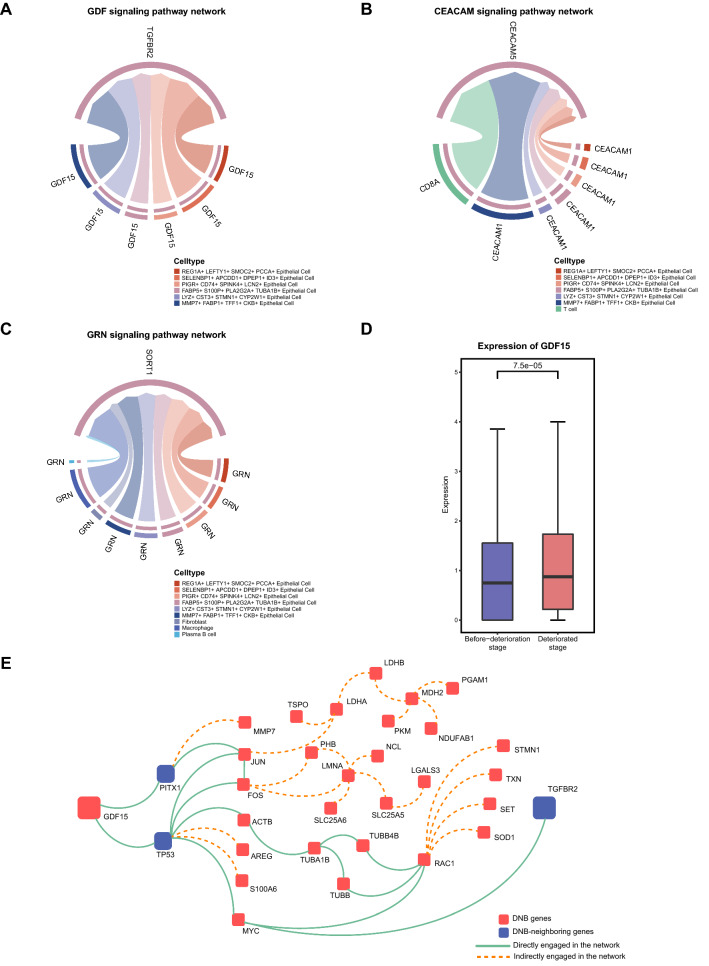
Intercellular communication contributes to epithelial cell deterioration. **A** Chord plot shows the interactions among cell subpopulations on the epithelial cell deterioration trajectory through the GDF signaling pathway. **B** Chord plot shows the interactions among cell populations in the TME through the CEACAM signaling pathway. **C** Chord plot shows the interactions among cell populations in the TME through the GRN signaling pathway. **D** The expression of *GDF15* in before-deterioration stage and deteriorated stage. Differences in expression are checked using the Wilcox test. **E** Cytoscape visualization of the protein–protein interaction network. Red rectangles represent DNB-genes, and blue rectangles represent DNB-neighboring genes. Genes that directly engaged in the network are represented by the solid green line, and genes that indirectly engaged in the network are represented by the orange dotted line

We performed CellChat analysis to explore the interactions between the six epithelial cell populations on the deterioration trajectory. *GDF15* promotes cell proliferation by binding to its receptor *TGFBR2* [39]. Compared to the other five subpopulations of epithelial cells, MMP7 + FABP1 + TFF1 + CKB + epithelial cell showed the highest expression of ligand-receptor pair in the GDF signaling pathway (Additional file 7: Fig. S7D). As a sender of the GDF signaling pathway, MMP7 + FABP1 + TFF1 + CKB + epithelial cells conveyed the strongest proliferation signal to FABP5 + S100P + PLA2G2A + TUBA1B + epithelial cells (Fig. 5A and Additional file 7: Fig. S7A), which may contribute to the progression of pre-deteriorated epithelial cells toward deterioration. In addition, the expression level of *GDF15* was significantly upregulated with epithelial cell deterioration (Fig. 5D), which is consistent with a previous study [40]. In this study, *GDF15* was a DNB gene in the pre-deteriorated epithelial cell subpopulation, and *TGFBR2* is a DNB-neighboring gene. DNB genes interact with their neighboring genes and may provide feedback to the DNB gene population to influence cellular communication (Fig. 5E). In conclusion, we suggest that malignant epithelial cells may accelerate the progression of pre-deterioration of epithelial cells toward deterioration through the GDF signaling pathway.

Furthermore, we investigated whether intercellular communication among cell populations in the tumor microenvironment (TME) affects the deterioration of cell populations at the critical point. The results showed that T cells influenced the deterioration of FABP5 + S100P + PLA2G2A + TUBA1B + epithelial cells through *CD8A-CEACAM5*, along with the highest communication probability and increased expression of the ligand-receptor pair (Fig. 5B and Additional file 7: Fig. S7B, E). Additionally, macrophages, plasma B cells, and fibroblasts affected the deterioration of FABP5 + S100P + PLA2G2A + TUBA1B + epithelial cells through *GRN-SORT1* (Fig. 5C). Notably, macrophages possessed the highest expression of ligand-receptor pairs and the highest communication probability in the GRN signaling pathway (Additional file 7: Fig. S7C, F). These results suggest that T cells and macrophages may be involved in epithelial cell deterioration through the CEACAM/GRN signaling pathway.

## Discussion

Determining the process of epithelial cell deterioration is a significant challenge when dissecting colorectal tumorigenesis. However, scRNA-seq is a useful technique for characterizing cell subpopulations in CRC. This study describes the trajectory of epithelial cell deterioration. We used DNB to identify biomarkers for pre-deteriorated epithelial cells during the critical deterioration period. At the level of intercellular communication, we discovered that malignant epithelial cells contribute to the deteriorating progression of pre-deteriorated epithelial cells through the GDF signaling pathway.

We identified a subpopulation of pre-deteriorated epithelial cells during the deterioration process using the DNB method. Compared to conventional static methods based on differential expression of molecular biomarkers to detect molecular alterations in cell subpopulations, DNB provides advantages in identifying the deterioration of epithelial cells in CRC [41]. Pre-deteriorated epithelial cells represent a critical transition period in which the expression patterns of DNB-neighboring genes are flipped. DNB genes were enriched in the regulation of NIK/NF-kappaB signaling. It has been shown that NF-κB is a major regulator of gene expression in inflammatory- associated malignancies [42], and suppression of this pathway may be a potential therapy strategy for cancer [43]. In addition, DNB-neighboring genes were enriched in cancer and apoptosis pathways. Dysregulation of apoptosis is correlated with uncontrolled cell proliferation and cancer progression [44]. And the dysregulation of apoptosis is correlated with mitochondrial dysfunction [45]. The release of cytochrome c into the cytoplasm and the opening of the mitochondrial transition pore can activate the apoptotic process [46, 47]. DNB-neighboring reverse genes in pre-deteriorated epithelial cells may provide clues for the intervention in deterioration. Furthermore, we discovered that the enrichment scores of the Wnt and PI3K-Akt pathways were significantly elevated after the critical period of deterioration. Aberrant Wnt signaling and overexpression of PI3K-Akt signaling have been reported in numerous malignancies, particularly CRC [48, 49]. Moreover, we discovered that MYC signaling pathways associated with cell proliferation were significantly enriched in pre-deteriorated epithelial cells. Sustaining proliferative signaling is considered as a hallmark of cancer [50]. Maintenance of cell proliferation signals in pre-deteriorated epithelial cells may contribute to their progression toward deterioration. In conclusion, pre-deteriorated epithelial cell populations may be a new cell subpopulation for cellular targeting in early intervention studies in CRC. Prevention of epithelial cell deterioration requires further investigation.

*FOS/JUN* interacted with *P53* in the DNB gene interaction network. The expression of c-Jun has consistently been shown to be involved in growth inhibition and apoptosis induction by several anticancer drugs [51]. Previous research has shown that in prostate cancer cell lines, a lack of *FOS* promotes cell proliferation and results in changes to oncogenic pathways [52]. Additionally, previous studies have demonstrated that the AP-1 motif, which binds to c-Fos/c-Jun, is required for efficient transcription of the human *P53* promoter [53, 54]. Moreover, downregulation of c-Fos and c-Jun expression leads to reduced expression of endogenous *P53* [53]. Furthermore, *P53* is an important tumor suppressor gene that induces apoptosis [55]. P53-induced apoptosis in epithelial cells is a crucial mechanism for the prevention of tumor progression. In our gene regulatory role analysis, *FOS* and *JUN* induced low expression of P53-regulated downstream pro-apoptotic genes and high expression of anti-apoptotic genes through suppressing *P53* expression, which in turn inhibited P53-induced apoptosis. However, the transcriptional profile during epithelial cell deterioration was aberrant. *FOS/JUN*, as the DNB gene, drive *P53* into the critical state of deterioration, which may have an impact on the P53-induced apoptotic process, suggesting that the disturbed apoptotic process in epithelial cells prior to deterioration may not ensure their normal proliferation. These studies of *FOS/JUN* mechanisms, along with our DNB gene expression patterns, indicated that *FOS/JUN* as a DNB gene has the potential to be an intervention target for pre-deteriorated cells.

We found that the intercellular signaling network contributes to epithelial cell deterioration. *GDF15*, a ligand of malignant epithelial cells, interacts with *TGFBR2*, the receptor of pre-deteriorated epithelial cells. *GDF15* is a member of the TGF-β superfamily [56] and has been previously identified as a new potential biomarker for cervical cancer [57]. In addition, *GDF15* overexpression accelerated the growth and progression of oral squamous cell carcinoma [58], whereas *GDF15* knockdown in malignant gliomas decreased cell proliferation in vitro and carcinogenesis in vivo [59]. Furthermore, *GDF15* promoted the progression of Esophageal Squamous Cell Carcinoma through the activation of *TGFBR2* [39]. Therefore, we speculated that malignant epithelial cells promote pre-deteriorated epithelial cells toward deterioration through GDF signaling. These results provide new insights into the mechanism of epithelial cell deterioration.

We discovered that the citrate cycle (TCA cycle) and glycolysis/gluconeogenesis were increased near the end of the proposed chronotropic trajectory after being downregulated early in the proposed chronotropic trajectory. This result ties well with previous study on the transcriptomic analysis of FAP patients [2]. In line with the previous study [36], the expression of *FOS* and *JUN* decreased significantly with epithelial cell deterioration. In addition, the transcriptional activity of *HNF4A* was upregulated after the critical period, this is consistent with what has been found in previous study [60]. Furthermore, the p53 signaling pathway was significantly enriched in pre-deteriorated epithelial cells, which is broadly in line with the result of another study [11]. Noteworthily, we clearly identified the subpopulation of pre-deteriorated epithelial cells at the single-cell level. And *FOS*/*JUN* were found to be biomarkers for the pre-deteriorated epithelial cell subpopulation in CRC.

However, this study has several limitations. The pre-deteriorated epithelial cells selected for the critical period included only 450 cells, but the cell number satisfied our analysis criteria (n > 6). Further confirmation of the subpopulation of pre-deteriorated epithelial cells using flow cytometry is required. In addition, further knockdown of *FOS* and *JUN* genes in cellular and animal models are required to confirm the precise mechanism by which *FOS/JUN* drive pre-deteriorated epithelial cells towards deterioration. It would be better to develop biomarkers in combination with more advanced nanomaterials, such as carbon nanotubes and nanostructured lipid nanocarriers [61–64].

## Conclusions

**Fig. 6:**
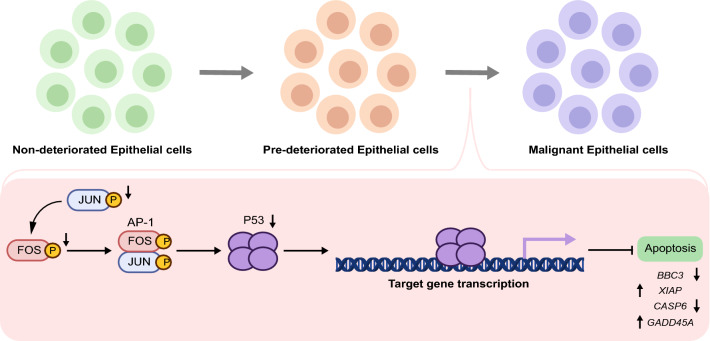
Diagram of the mechanism speculated based on the results of the study

In summary, we demonstrated the trajectory of epithelial cell deterioration and used DNB to characterize pre-deteriorated epithelial cells from adenoma and carcinoma tissues of CRC patients at the single-cell level. *FOS*/*JUN* regulated the expression of downstream apoptosis-related genes of the P53 pathway through inhibiting the expression of *P53*, thereby contributing to the suppression of P53-dependent apoptosis (Fig. 6). Malignant epithelial cells contributed to the progression of pre-deteriorated epithelial cells through GDF signaling (Fig. 6). These findings provide new insights into the mechanism of epithelial cell deterioration and toward early intervention in CRC.

## Supplementary Information

**Additional file 1: Figure S1.** Genes expression of epithelial cell deterioration.

**Additional file 2: Figure S2.** Inference of copy number variation based on scRNA data.

**Additional file 3: Figure S3.** The expression levels of DNB core genes in different pseudotime of epithelial cell subpopulation.

**Additional file 4: Figure S4.** Enrichment analysis of DNB genes and DNB neighboring genes.

**Additional file 5: Figure S5.** Co-expression network analysis.

**Additional file 6: Figure S6.** DNB genes drive the reversed expression of DNB neighbors.

**Additional file 7: Figure S7.** Heatmap and dot plot of GDF, CEACAM and GRN signaling pathway.

**Additional file 8: Table S1.** Signature genes for 6 clusters of all cells.

**Additional file 9: Table S2.** Signature genes for 7 clusters of epithelial cells.

**Additional file 10: Table S3.** Signature genes for 6 clusters of cells on pseudotime trajectory.

## Data Availability

Publicly available data set was analyzed in this study. This data can be found here: GSE161277: https://www.ncbi.nlm.nih.gov/geo/query/acc.cgi?acc=GSE161277.

## Abbreviations

CRC: Colorectal cancer
DNB: Dynamic network biomarker
scRNA-seq: Single-cell RNA sequencing
SCLE: Single-cell landscape entropy
SD: Standard deviation
PCC: Pearson’s correlation coefficient
GEO: Gene expression omnibus
PPI: Protein–protein interaction
DEG: Differentially expressed gene
GO: Gene ontology
KEGG: Kyoto encyclopedia of genes and genomes
GSVA: Gene set variation analysis
SCENIC: Single-cell regulatory network inference and clustering
TF: Transcription factor
UMAP: Uniform manifold approximation and projection
