# Supplementary figures and images for: Low expression of the dynamic network markers FOS/JUN in pre-deteriorated epithelial cells is associated with the progression of colorectal adenoma to carcinoma
Source: J Transl Med. 2023 Jan 25;21:45. doi: 10.1186/s12967-023-03890-5 (PMC9875500; doi:10.1186/s12967-023-03890-5)

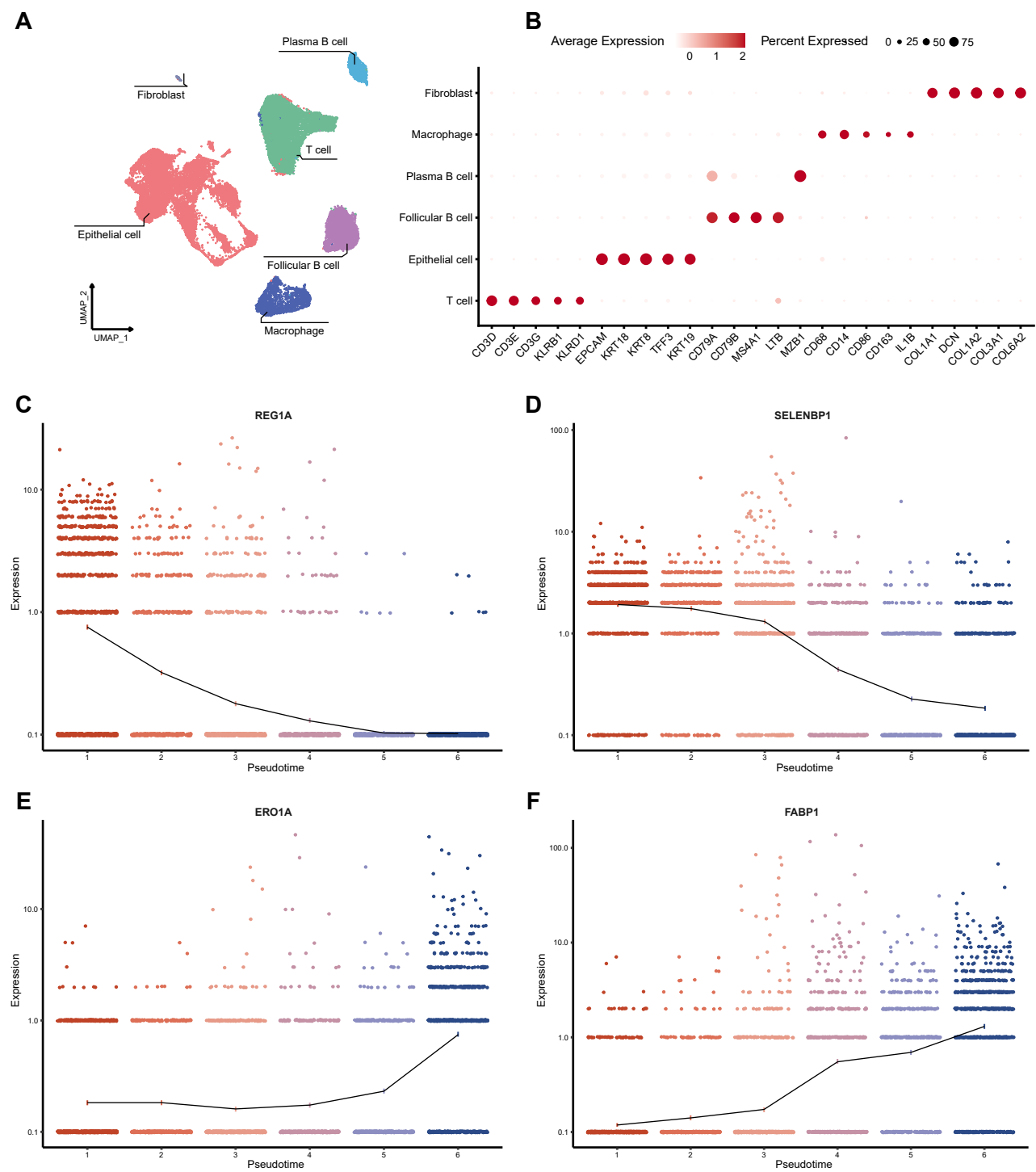

Supplement: Supplementary file 1 — Additional file 1: Figure S1. Genes expression of epithelial cell deterioration. [file 12967_2023_3890_MOESM1_ESM.pdf]

inferCNV

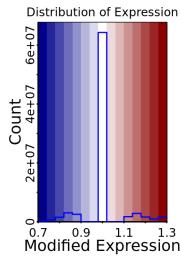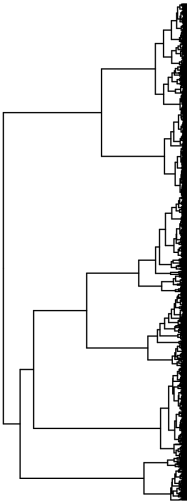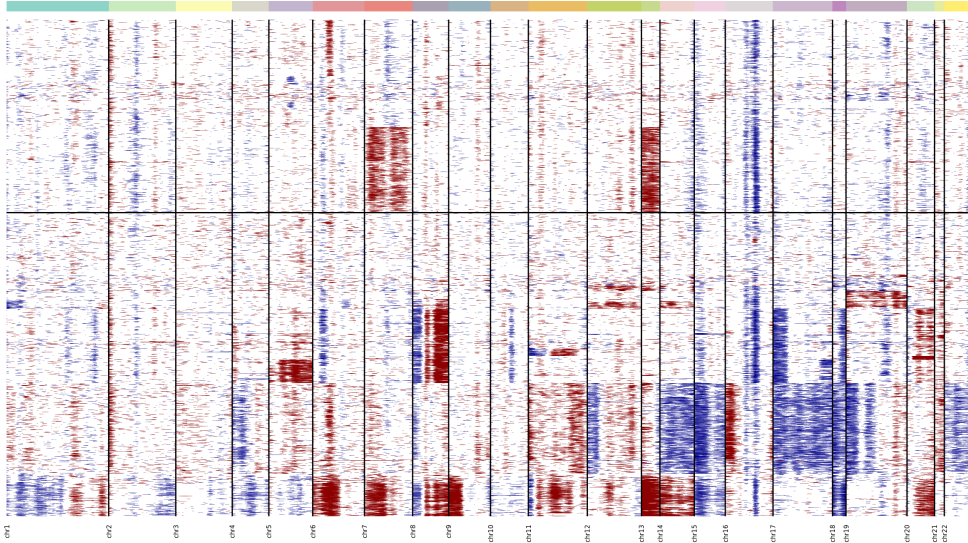

Genomic Region

Observations (Cells)

References (Cells)

normal

carcinoma adenoma

Supplement: Supplementary file 2 — Additional file 2: Figure S2. Inference of copy number variation based on scRNA data. [file 12967_2023_3890_MOESM2_ESM.pdf]

**A****GAPDH**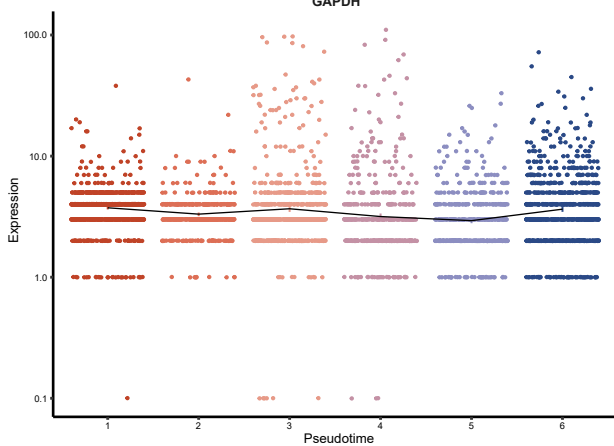**B****EEF2**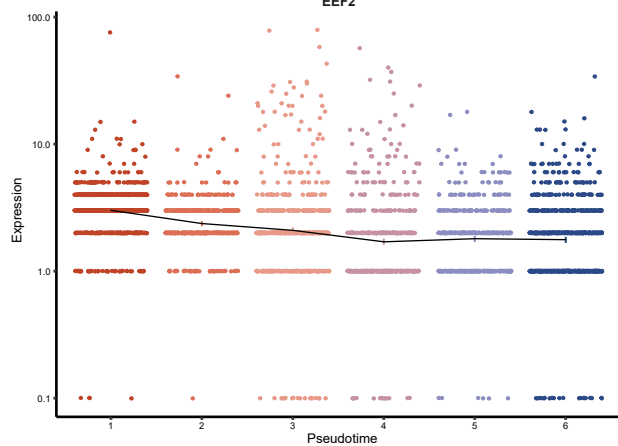**C****HSPA8**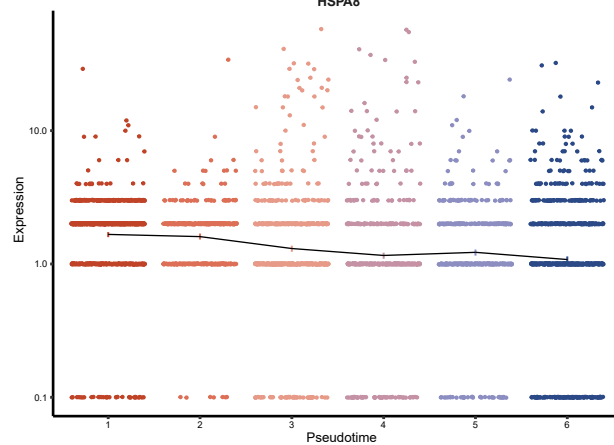**D****EEF1A1**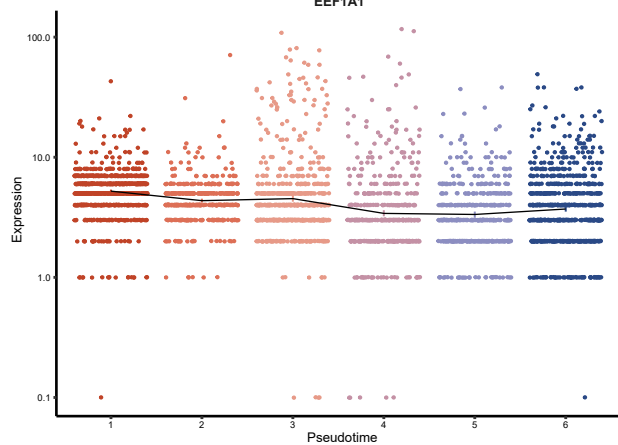

Supplement: Supplementary file 3 — Additional file 3: Figure S3. The expression levels of DNB core genes in different pseudotime of epithelial cell subpopulation. [file 12967_2023_3890_MOESM3_ESM.pdf]

**A**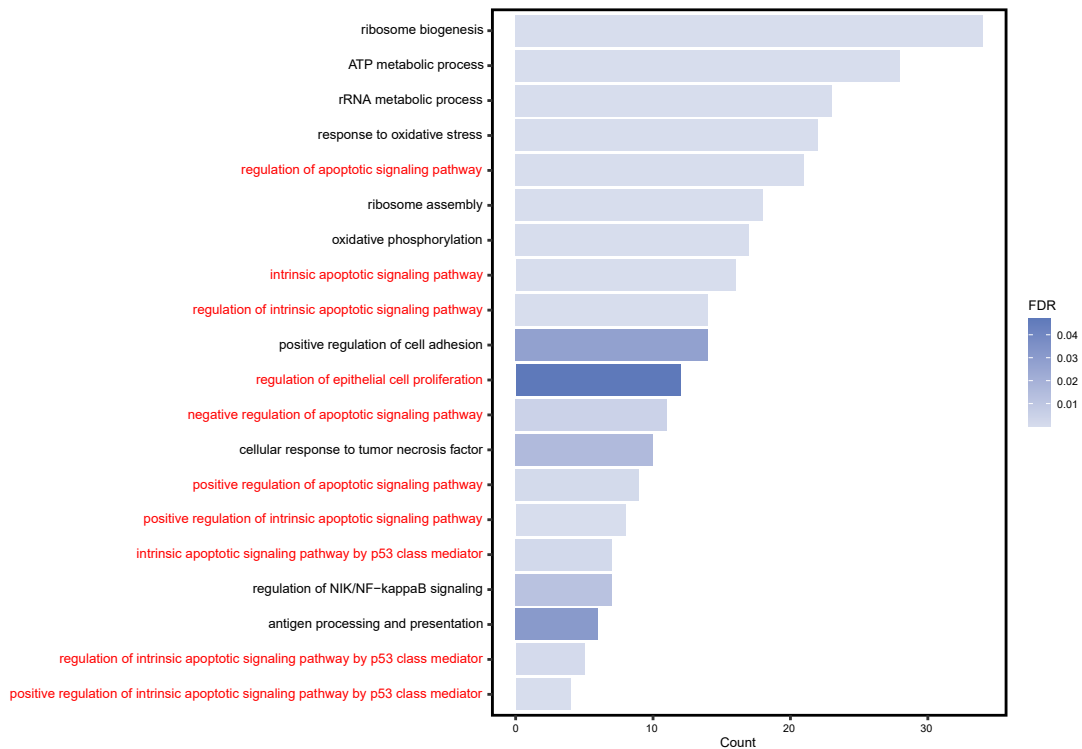**B**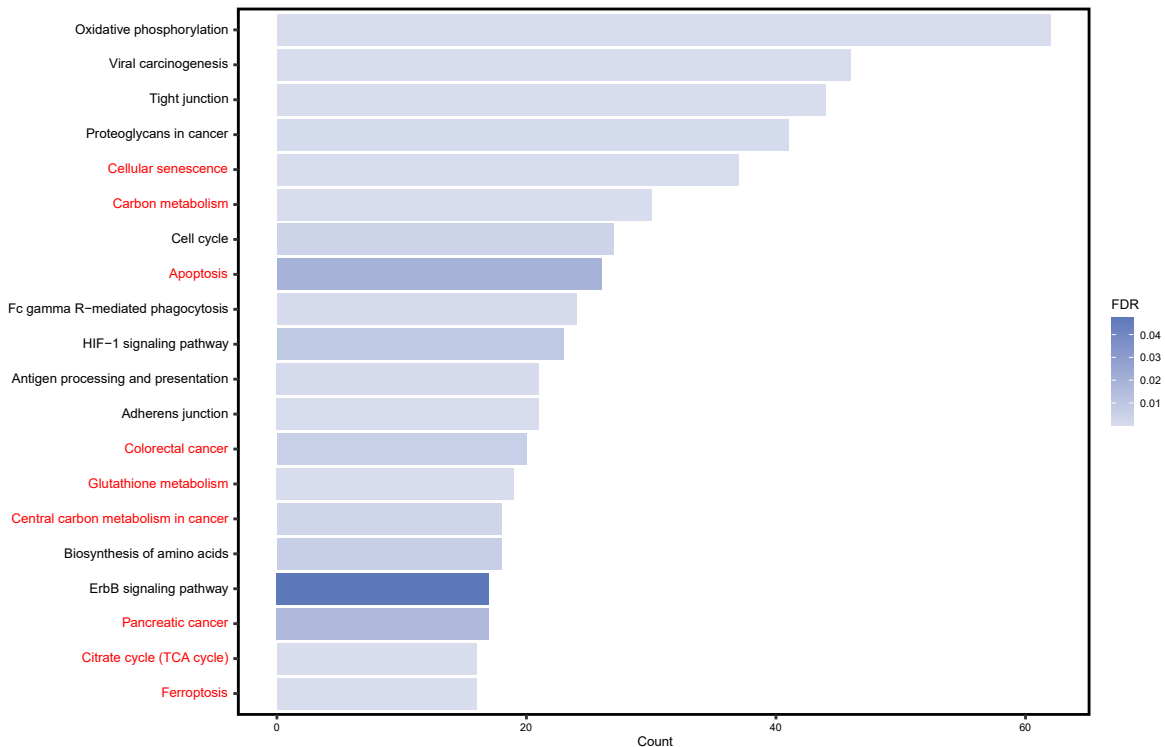

Supplement: Supplementary file 4 — Additional file 4: Figure S4. Enrichment analysis of DNB genes and DNB neighboring genes. [file 12967_2023_3890_MOESM4_ESM.pdf]

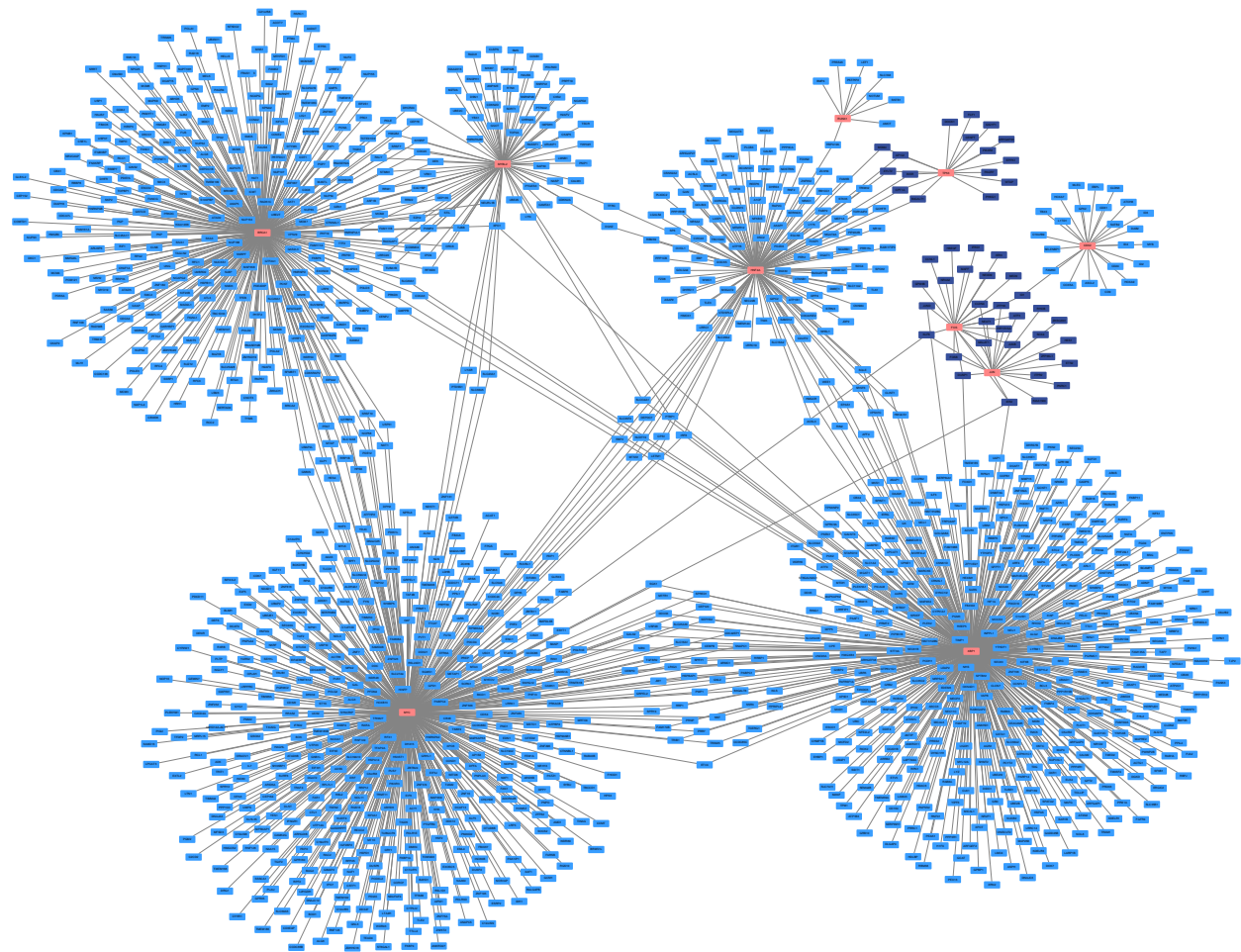

Supplement: Supplementary file 5 — Additional file 5: Figure S5. Co-expression network analysis. [file 12967_2023_3890_MOESM5_ESM.pdf]

**A**

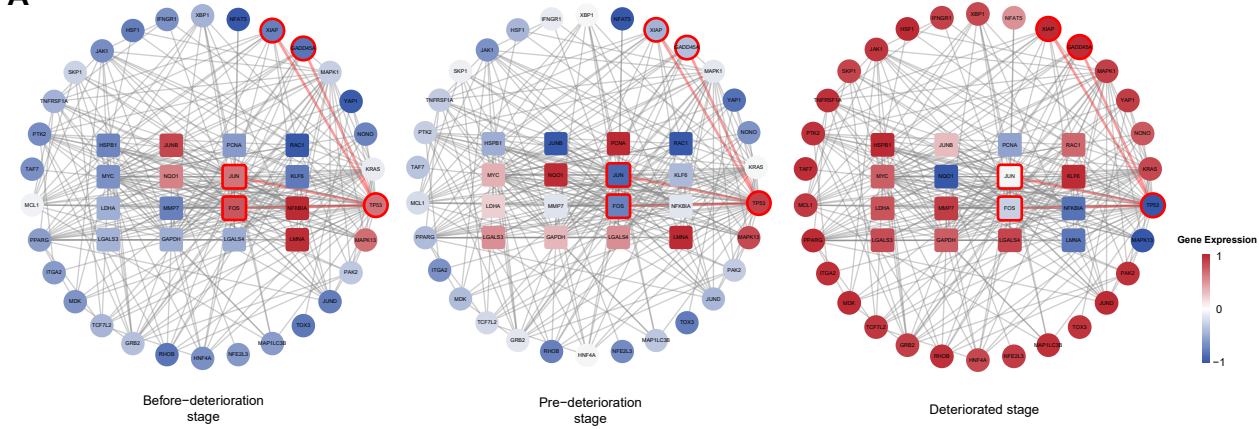

**B**

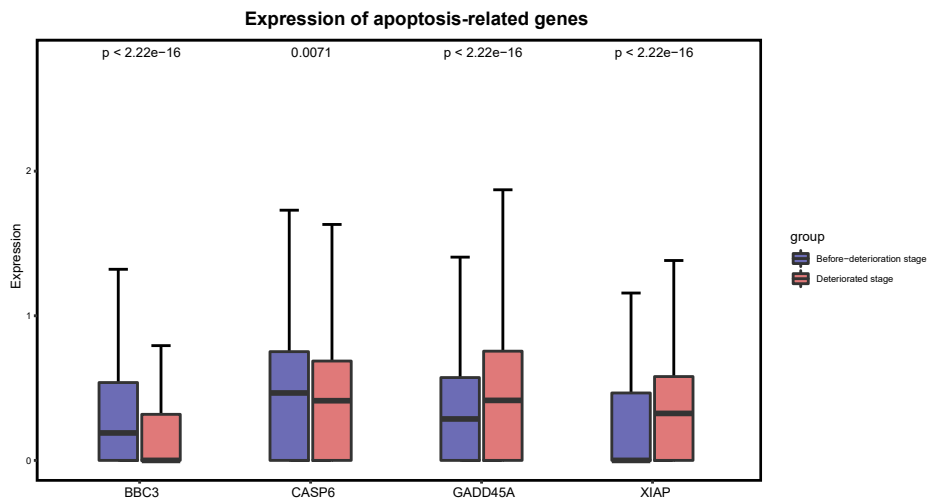

Supplement: Supplementary file 6 — Additional file 6: Figure S6. DNB genes drive the reversed expression of DNB neighbors. [file 12967_2023_3890_MOESM6_ESM.pdf]

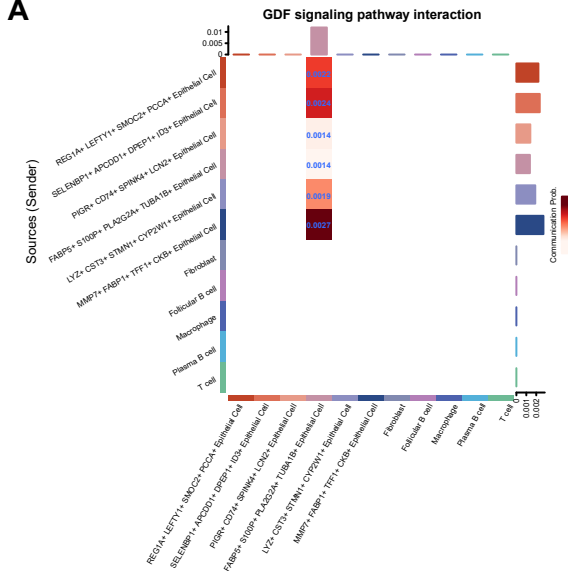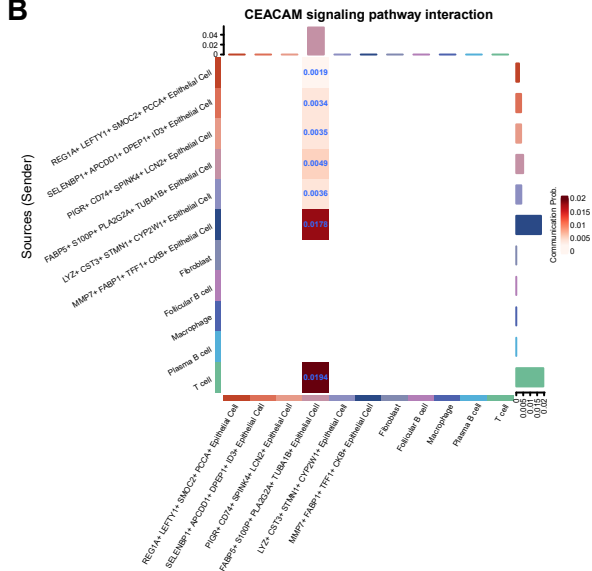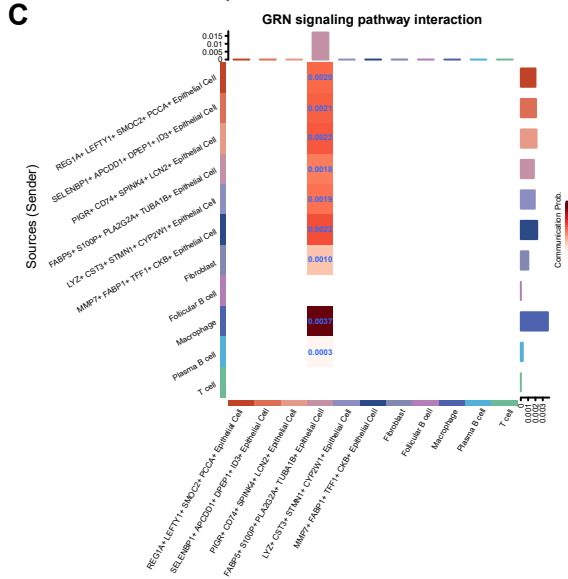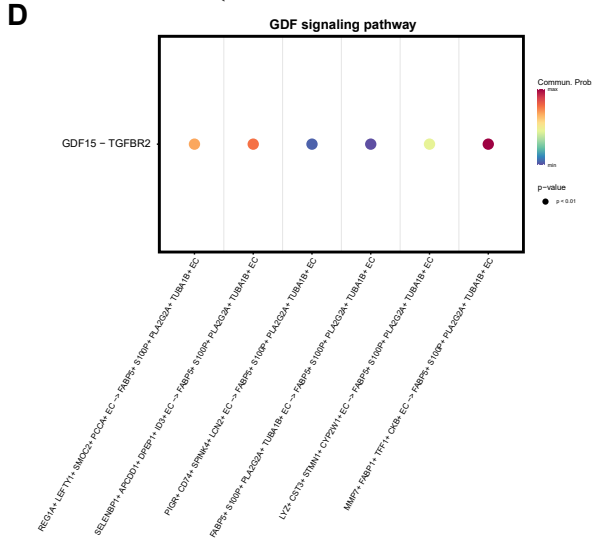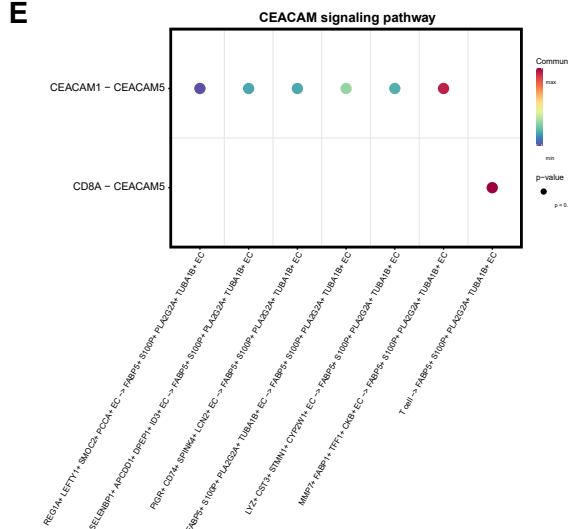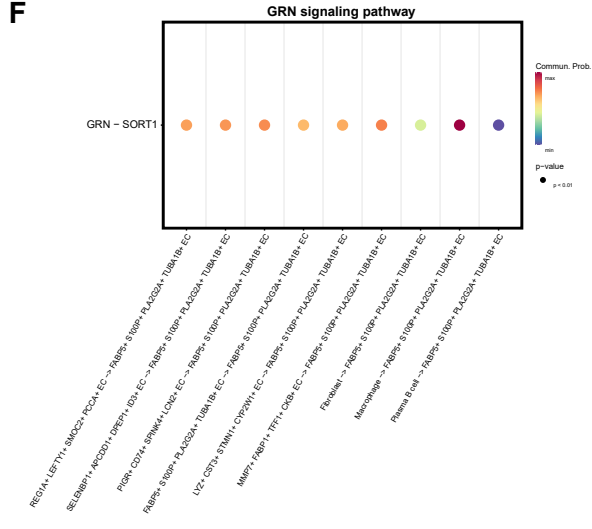

Supplement: Supplementary file 7 — Additional file 7: Figure S7. Heatmap and dot plot of GDF, CEACAM and GRN signaling pathway. [file 12967_2023_3890_MOESM7_ESM.pdf]
