# Supplementary material for: Low expression of the dynamic network markers FOS/JUN in pre-deteriorated epithelial cells is associated with the progression of colorectal adenoma to carcinoma
Source: J Transl Med. 2023 Jan 25;21:45. doi: 10.1186/s12967-023-03890-5 (PMC9875500; doi:10.1186/s12967-023-03890-5)
Supplement: Supplementary file 8 — Additional file 8: Table S1. Signature genes for 6 clusters of all cells. [file 12967_2023_3890_MOESM8_ESM.pdf]

Table S1. Signature genes for 6 clusters of all cells

cluster	gene	p_val	avg_log2FC	pct.1	pct.2	p_val_adj
Epithelial cell	TFF3	0	4.775532553	0.91	0.202	0
Epithelial cell	ZG16	2.11E-128	4.713703139	0.273	0.157	4.90E-124
Epithelial cell	PHGR1	0	4.310676349	0.924	0.207	0
Epithelial cell	PIGR	0	3.941929027	0.663	0.145	0
Epithelial cell	OLFM4	0	3.759079124	0.632	0.065	0
Epithelial cell	KRT18	0	3.723419847	0.964	0.1	0
Epithelial cell	KRT8	0	3.718383643	0.969	0.144	0
Epithelial cell	IFI27	0	3.696834658	0.949	0.089	0
Epithelial cell	AGR2	0	3.668342346	0.923	0.107	0
Epithelial cell	EPCAM	0	3.664947362	0.963	0.094	0
Epithelial cell	TSPAN8	0	3.659246121	0.932	0.087	0
Epithelial cell	LGALS4	0	3.637632395	0.943	0.129	0
Epithelial cell	FABP1	0	3.623056912	0.552	0.145	0
Epithelial cell	SPINK1	0	3.541833746	0.811	0.052	0
Epithelial cell	FXYD3	0	3.455606539	0.946	0.119	0
Epithelial cell	SPINK4	0	3.377527816	0.28	0.06	0
Epithelial cell	KRT19	0	3.201384782	0.893	0.076	0
Epithelial cell	CKB	0	3.100417955	0.784	0.081	0
Epithelial cell	ELF3	0	3.098718444	0.941	0.083	0
Epithelial cell	LGALS3	0	3.03517615	0.944	0.243	0
Epithelial cell	LCN2	0	3.032748834	0.68	0.038	0
Epithelial cell	CLDN3	0	3.00629614	0.926	0.057	0
Epithelial cell	ATP1B1	0	2.814853293	0.873	0.054	0
Epithelial cell	FCGBP	0	2.792958114	0.405	0.061	0
Epithelial cell	CD24	0	2.695465998	0.872	0.144	0
Epithelial cell	CLDN4	0	2.681584031	0.924	0.101	0
Epithelial cell	CEACAM5	0	2.656014227	0.84	0.021	0
Epithelial cell	GPX2	0	2.629889455	0.831	0.057	0
Epithelial cell	SMIM22	0	2.521569074	0.923	0.03	0
Epithelial cell	ID1	0	2.485223711	0.69	0.06	0
Epithelial cell	FAM3D	0	2.481147072	0.85	0.035	0
Epithelial cell	PLA2G2A	0	2.448017178	0.43	0.036	0
Epithelial cell	MUC2	0	2.41708342	0.27	0.038	0
Epithelial cell	C15orf48	0	2.40669194	0.818	0.13	0
Epithelial cell	C19orf33	0	2.36724946	0.815	0.028	0
Epithelial cell	S100A6	0	2.350411829	0.988	0.714	0
Epithelial cell	SLC12A2	0	2.298650114	0.785	0.049	0
Epithelial cell	TXN	0	2.265233535	0.916	0.347	0
Epithelial cell	SPINT2	0	2.217438158	0.922	0.152	0
Epithelial cell	TSPAN1	0	2.216403888	0.718	0.034	0
Epithelial cell	S100A10	0	2.207343838	0.965	0.472	0

Epithelial cell	IGFBP2	0	2.191709824	0.55	0.015	0
Epithelial cell	SOX4	0	2.185481791	0.731	0.031	0
Epithelial cell	MT1G	0	2.167424225	0.416	0.044	0
Epithelial cell	CLDN7	0	2.159619517	0.882	0.055	0
Epithelial cell	CD9	0	2.157984333	0.882	0.149	0
Epithelial cell	LEFTY1	0	2.076731615	0.569	0.018	0
Epithelial cell	TFF1	0	2.03241995	0.419	0.011	0
Epithelial cell	SELENBP1	0	1.994331238	0.664	0.032	0
Epithelial cell	TMEM54	0	1.951686614	0.837	0.027	0
Epithelial cell	REG4	0	1.935770303	0.285	0.014	0
Epithelial cell	NUPR1	0	1.909179057	0.593	0.032	0
Epithelial cell	TPM1	0	1.889989185	0.88	0.068	0
Epithelial cell	S100A14	0	1.87958231	0.77	0.022	0
Epithelial cell	AGR3	0	1.862255561	0.771	0.02	0
Epithelial cell	S100P	0	1.858232145	0.612	0.024	0
Epithelial cell	MDK	0	1.842578808	0.677	0.026	0
Epithelial cell	KLF5	0	1.838986652	0.807	0.019	0
Epithelial cell	MUC13	0	1.821465612	0.716	0.02	0
Epithelial cell	MT1E	0	1.798453856	0.519	0.051	0
Epithelial cell	CYSTM1	0	1.776123714	0.853	0.1	0
Epithelial cell	MMP7	0	1.738023682	0.253	0.007	0
Epithelial cell	MGST1	0	1.699670448	0.744	0.054	0
Epithelial cell	TMEM141	0	1.694224184	0.833	0.113	0
Epithelial cell	PRSS3	0	1.689890822	0.728	0.019	0
Epithelial cell	HES1	0	1.682026864	0.703	0.048	0
Epithelial cell	PPDPF	0	1.670314772	0.96	0.546	0
Epithelial cell	STARD10	0	1.649265591	0.802	0.034	0
Epithelial cell	MT-ND1	0	1.648585272	0.981	0.952	0
Epithelial cell	MUC12	0	1.64676821	0.599	0.033	0
Epithelial cell	CEACAM6	0	1.637680599	0.451	0.012	0
Epithelial cell	UQCRQ	0	1.63761178	0.925	0.431	0
Epithelial cell	CAMK2N1	0	1.627815593	0.769	0.026	0
Epithelial cell	APP	0	1.612839564	0.841	0.066	0
Epithelial cell	TSC22D1	0	1.606047325	0.776	0.056	0
Epithelial cell	KRT20	0	1.604552635	0.439	0.014	0
Epithelial cell	PPP1R1B	0	1.60312721	0.748	0.012	0
Epithelial cell	GSN	0	1.595778464	0.577	0.095	0
Epithelial cell	LGALS3BP	0	1.587876601	0.796	0.042	0
Epithelial cell	PERP	0	1.573818759	0.822	0.103	0
Epithelial cell	C10orf99	0	1.564024941	0.635	0.016	0
Epithelial cell	CA2	0	1.552732746	0.344	0.039	0
Epithelial cell	PRDX5	0	1.536667975	0.905	0.477	0
Epithelial cell	MT-CO3	0	1.526020878	0.994	0.996	0

Epithelial cell	MLEC	0	1.51603042	0.804	0.182	0
Epithelial cell	WFDC2	0	1.507392029	0.362	0.014	0
Epithelial cell	DSTN	0	1.496715111	0.904	0.263	0
Epithelial cell	IFITM3	0	1.4911112	0.747	0.099	0
Epithelial cell	ASCL2	0	1.483269205	0.524	0.03	0
Epithelial cell	SRI	0	1.482058399	0.901	0.312	0
Epithelial cell	GDF15	0	1.480585366	0.501	0.021	0
Epithelial cell	MUC1	0	1.478273738	0.574	0.016	0
Epithelial cell	GSTP1	0	1.471447511	0.92	0.506	0
Epithelial cell	MT-CO2	0	1.463117261	0.992	0.994	0
Epithelial cell	ANXA2	0	1.456496252	0.886	0.313	0
Epithelial cell	ST14	0	1.455131497	0.809	0.037	0
Epithelial cell	ITM2C	0	1.449361674	0.826	0.276	0
Epithelial cell	NPDC1	0	1.444727439	0.664	0.016	0
Epithelial cell	COX5A	0	1.437573185	0.889	0.346	0
Epithelial cell	CDX1	0	1.425034686	0.736	0.014	0
Epithelial cell	CDH17	0	1.422187376	0.759	0.022	0
Epithelial cell	MARCKSL1	0	1.414852625	0.863	0.156	0
Epithelial cell	SERINC2	0	1.400728745	0.766	0.017	0
Epithelial cell	PPA1	0	1.397126422	0.826	0.264	0
Epithelial cell	LSR	0	1.39587052	0.809	0.054	0
Epithelial cell	ATP5MC3	0	1.385179266	0.885	0.429	0
Epithelial cell	CDC42EP5	0	1.375317871	0.735	0.01	0
Epithelial cell	CDX2	0	1.374387952	0.72	0.011	0
Epithelial cell	COX5B	0	1.362790566	0.947	0.555	0
Epithelial cell	ADH1C	0	1.359543096	0.392	0.014	0
Epithelial cell	ITGA6	0	1.356675292	0.698	0.035	0
Epithelial cell	NBL1	0	1.355428986	0.803	0.064	0
Epithelial cell	RAB25	0	1.325172675	0.749	0.01	0
Epithelial cell	SLPI	0	1.318161504	0.553	0.02	0
Epithelial cell	DSG2	0	1.316896303	0.743	0.011	0
Epithelial cell	GOLM1	0	1.31527571	0.699	0.016	0
Epithelial cell	UQCRH	0	1.314799174	0.898	0.525	0
Epithelial cell	ALDH2	0	1.313610023	0.762	0.067	0
Epithelial cell	S100A16	0	1.311741468	0.71	0.015	0
Epithelial cell	GOLIM4	0	1.308444194	0.734	0.072	0
Epithelial cell	SLC44A4	0	1.306935788	0.724	0.013	0
Epithelial cell	CXADR	0	1.306887881	0.711	0.007	0
Epithelial cell	SLC25A5	0	1.30017307	0.891	0.42	0
Epithelial cell	PRAC1	0	1.283234257	0.361	0.013	0
Epithelial cell	MISP	0	1.27541136	0.645	0.013	0
Epithelial cell	DSP	0	1.270604508	0.716	0.009	0
Epithelial cell	MAL2	0	1.268374717	0.692	0.01	0

Epithelial cell	PHLDA2	0	1.266001797	0.611	0.034	0
Epithelial cell	GMDS	0	1.263285129	0.722	0.055	0
Epithelial cell	TUBB4B	0	1.254860474	0.784	0.156	0
Epithelial cell	SFN	0	1.248301798	0.61	0.016	0
Epithelial cell	NQO1	0	1.235218818	0.634	0.019	0
Epithelial cell	RAC1	0	1.234939668	0.924	0.428	0
Epithelial cell	CD151	0	1.230599798	0.829	0.113	0
Epithelial cell	ETHE1	0	1.222656183	0.744	0.115	0
Epithelial cell	LIMA1	0	1.212626133	0.766	0.064	0
Epithelial cell	VSIG2	0	1.207405437	0.555	0.016	0
Epithelial cell	FAM84A	0	1.206945738	0.671	0.01	0
Epithelial cell	RRBP1	0	1.201053748	0.823	0.14	0
Epithelial cell	GPRC5A	0	1.200318378	0.597	0.01	0
Epithelial cell	PDLIM1	0	1.194304689	0.799	0.095	0
Epithelial cell	PCBD1	0	1.193601914	0.763	0.081	0
Epithelial cell	CSTB	0	1.192674272	0.844	0.377	0
Epithelial cell	SELENOP	0	1.189948266	0.395	0.036	0
Epithelial cell	BACE2	0	1.189849174	0.66	0.015	0
Epithelial cell	TMC5	0	1.188904826	0.658	0.006	0
Epithelial cell	MPST	0	1.184141954	0.788	0.123	0
Epithelial cell	CYC1	0	1.181368887	0.799	0.213	0
Epithelial cell	HSPB1	0	1.178387162	0.761	0.23	0
Epithelial cell	MUC4	0	1.177214044	0.52	0.01	0
Epithelial cell	CDH1	0	1.175360662	0.702	0.008	0
Epithelial cell	MGST2	0	1.16828356	0.749	0.07	0
Epithelial cell	TMEM176B	0	1.162170032	0.7	0.053	0
Epithelial cell	DST	0	1.156188394	0.555	0.028	0
Epithelial cell	ATP5F1B	0	1.15586895	0.868	0.369	0
Epithelial cell	FHL2	0	1.151993109	0.594	0.013	0
Epithelial cell	ATP5MD	0	1.150062911	0.917	0.518	0
Epithelial cell	MT-CO1	0	1.148314076	0.993	0.997	0
Epithelial cell	ASS1	0	1.147548657	0.559	0.013	0
Epithelial cell	ATP1A1	0	1.139492754	0.829	0.246	0
Epithelial cell	ANXA4	0	1.135102098	0.751	0.09	0
Epithelial cell	MT-ATP6	0	1.129802813	0.985	0.988	0
Epithelial cell	CCND1	0	1.124876849	0.544	0.018	0
Epithelial cell	PLPP2	0	1.12391946	0.662	0.009	0
Epithelial cell	COX6C	0	1.123866067	0.935	0.595	0
Epithelial cell	PDZK1IP1	0	1.108001784	0.597	0.008	0
Epithelial cell	GAPDH	0	1.107406607	0.976	0.842	0
Epithelial cell	SPATS2L	0	1.105269607	0.637	0.05	0
Epithelial cell	ETS2	0	1.097483319	0.687	0.074	0
Epithelial cell	LDHA	0	1.091892837	0.872	0.471	0

Epithelial cell	SPINT1	0	1.091872211	0.692	0.031	0
Epithelial cell	HSPE1	0	1.082655458	0.865	0.402	0
Epithelial cell	H2AFJ	0	1.079566653	0.803	0.133	0
Epithelial cell	HSPD1	0	1.077169203	0.799	0.291	0
Epithelial cell	TXNDC17	0	1.070729647	0.796	0.156	0
Epithelial cell	COX7B	0	1.06994557	0.919	0.505	0
Epithelial cell	KRTCAP3	0	1.068832373	0.667	0.012	0
Epithelial cell	MPC2	0	1.06825307	0.809	0.196	0
Epithelial cell	COX7A2	0	1.064767436	0.943	0.622	0
Epithelial cell	SERPINA1	0	1.058727863	0.32	0.087	0
Epithelial cell	RNF43	0	1.051036013	0.584	0.02	0
Epithelial cell	MT-CYB	0	1.049685435	0.986	0.985	0
Epithelial cell	LMNA	0	1.046688118	0.795	0.099	0
Epithelial cell	MT-ND2	0	1.046686274	0.989	0.989	0
Epithelial cell	COX6A1	0	1.043819467	0.932	0.539	0
Epithelial cell	VIL1	0	1.041063096	0.667	0.009	0
Epithelial cell	MT-ND4	0	1.039236022	0.99	0.992	0
Epithelial cell	NANS	0	1.035227879	0.724	0.117	0
Epithelial cell	PPIC	0	1.03481848	0.647	0.012	0
Epithelial cell	VDAC1	0	1.032941323	0.827	0.282	0
Epithelial cell	ERBB3	0	1.032324406	0.673	0.01	0
Epithelial cell	TSPO	0	1.030367344	0.866	0.28	0
Epithelial cell	TMEM106C	0	1.030166463	0.723	0.07	0
Epithelial cell	CTTN	0	1.029197126	0.671	0.013	0
Epithelial cell	KLK1	0	1.027837597	0.414	0.024	0
Epithelial cell	CHMP4B	0	1.026808576	0.818	0.175	0
Epithelial cell	PRSS23	0	1.023240997	0.491	0.023	0
Epithelial cell	MINOS1	0	1.022524729	0.877	0.383	0
Epithelial cell	STMN1	0	1.020443853	0.479	0.106	0
Epithelial cell	H1F0	0	1.016022358	0.623	0.022	0
Epithelial cell	TM4SF1	0	1.014404353	0.41	0.008	0
Epithelial cell	PRSS8	0	1.014303448	0.605	0.01	0
Epithelial cell	ID3	0	1.013514552	0.602	0.213	0
Epithelial cell	ATP5PF	0	1.013191897	0.902	0.403	0
Epithelial cell	AOC1	0	1.01265501	0.484	0.008	0
Epithelial cell	LIPH	0	1.00788931	0.52	0.003	0
Epithelial cell	SOX9	0	1.005698363	0.574	0.009	0
Epithelial cell	COX8A	0	1.003398244	0.914	0.498	0
Epithelial cell	SLIRP	0	1.001903608	0.813	0.234	0
Epithelial cell	ATP5MC1	0	0.999261304	0.778	0.291	0
Epithelial cell	DPEP1	0	0.99400727	0.333	0.009	0
Epithelial cell	CES2	0	0.993032586	0.519	0.052	0
Epithelial cell	EMP2	0	0.99076876	0.622	0.01	0

Epithelial cell	FABP5	0	0.984909327	0.596	0.111	0
Epithelial cell	GGH	0	0.97944897	0.572	0.032	0
Epithelial cell	FERMT1	0	0.977675522	0.55	0.004	0
Epithelial cell	FAM162A	0	0.975940137	0.772	0.169	0
Epithelial cell	TRIM31	0	0.972727664	0.355	0.008	0
Epithelial cell	PRDX2	0	0.971944826	0.795	0.274	0
Epithelial cell	PTPRF	0	0.970878747	0.665	0.007	0
Epithelial cell	TMEM45B	0	0.968357854	0.582	0.009	0
Epithelial cell	UQCRC1	0	0.96320736	0.789	0.187	0
Epithelial cell	QPRT	0	0.962947933	0.576	0.032	0
Epithelial cell	ANXA3	0	0.959909293	0.533	0.006	0
Epithelial cell	TCEA3	0	0.959738953	0.545	0.01	0
Epithelial cell	S100A11	0	0.959352795	0.891	0.494	0
Epithelial cell	CLTA	0	0.95599544	0.844	0.261	0
Epithelial cell	ATP5IF1	0	0.953104364	0.871	0.369	0
Epithelial cell	CLTB	0	0.95254044	0.813	0.212	0
Epithelial cell	MGST3	0	0.952204126	0.795	0.256	0
Epithelial cell	TRIM2	0	0.951844626	0.639	0.008	0
Epithelial cell	DSC2	0	0.950105758	0.617	0.012	0
Epithelial cell	TIMM13	0	0.947501885	0.767	0.188	0
Epithelial cell	HMGCS2	0	0.946211156	0.446	0.009	0
Epithelial cell	SDCBP2	0	0.944858445	0.356	0.012	0
Epithelial cell	SERPINB6	0	0.944479381	0.75	0.162	0
Epithelial cell	GPR160	0	0.94315528	0.661	0.044	0
Epithelial cell	TSPAN3	0	0.941571465	0.715	0.098	0
Epithelial cell	ADIRF	0	0.940173878	0.404	0.016	0
Epithelial cell	PCCA	0	0.939226306	0.377	0.023	0
Epithelial cell	MT-ND3	0	0.937785849	0.984	0.986	0
Epithelial cell	HEBP2	0	0.933463174	0.766	0.12	0
Epithelial cell	COX4I1	0	0.933368846	0.962	0.777	0
Epithelial cell	TMSB10	0	0.932334558	0.983	0.948	0
Epithelial cell	IER3	0	0.927484311	0.679	0.103	0
Epithelial cell	HES6	0	0.925459694	0.439	0.027	0
Epithelial cell	SCD	0	0.92427357	0.406	0.015	0
Epithelial cell	RCN1	0	0.923603239	0.588	0.045	0
Epithelial cell	TM9SF3	0	0.920302544	0.792	0.147	0
Epithelial cell	NDUFC2	0	0.919046999	0.826	0.304	0
Epithelial cell	GPA33	0	0.918997396	0.525	0.023	0
Epithelial cell	QTRT1	0	0.918958387	0.687	0.091	0
Epithelial cell	NDUFAB1	0	0.91820167	0.789	0.229	0
Epithelial cell	MT1M	0	0.915743243	0.257	0.008	0
Epithelial cell	DBI	0	0.911999986	0.869	0.43	0
Epithelial cell	AMN	0	0.911613511	0.535	0.022	0

Epithelial cell	PRAP1	0	0.91158188	0.351	0.011	0
Epithelial cell	CYB5A	0	0.910992072	0.702	0.082	0
Epithelial cell	CDHR5	0	0.907344607	0.381	0.01	0
Epithelial cell	LMO7	0	0.907224154	0.55	0.029	0
Epithelial cell	ST6GALNAC1	0	0.905925254	0.467	0.006	0
Epithelial cell	DYNLL1	0	0.900816095	0.904	0.462	0
Epithelial cell	TMEM176A	0	0.898393722	0.572	0.025	0
Epithelial cell	MYH14	0	0.898102876	0.588	0.007	0
Epithelial cell	CMBL	0	0.897494782	0.584	0.006	0
Epithelial cell	ARPC1A	0	0.894753735	0.742	0.11	0
Epithelial cell	KLF4	0	0.890578573	0.612	0.07	0
Epithelial cell	COX6B1	0	0.887329268	0.945	0.638	0
Epithelial cell	MDH2	0	0.886103127	0.799	0.258	0
Epithelial cell	SLC25A3	0	0.883389456	0.879	0.441	0
Epithelial cell	ABCC3	0	0.882795666	0.587	0.013	0
Epithelial cell	BSG	0	0.882653567	0.864	0.341	0
Epithelial cell	SLC26A2	0	0.882358174	0.299	0.056	0
Epithelial cell	STAP2	0	0.881097396	0.612	0.012	0
Epithelial cell	NDUFB4	0	0.879857722	0.858	0.332	0
Epithelial cell	NEAT1	0	0.878977669	0.942	0.665	0
Epithelial cell	PCK1	0	0.87589938	0.314	0.011	0
Epithelial cell	MALL	0	0.875845898	0.343	0.005	0
Epithelial cell	CD63	0	0.874710124	0.949	0.555	0
Epithelial cell	AP1M2	0	0.874577078	0.62	0.007	0
Epithelial cell	HINT1	0	0.873838498	0.917	0.666	0
Epithelial cell	NDUFA1	0	0.873426626	0.909	0.473	0
Epithelial cell	TNFRSF12A	0	0.87322955	0.395	0.019	0
Epithelial cell	PKP3	0	0.8711217	0.612	0.012	0
Epithelial cell	NET1	0	0.870217676	0.62	0.018	0
Epithelial cell	MARCKS	0	0.86967727	0.793	0.144	0
Epithelial cell	PPP1R14B	0	0.866015288	0.675	0.081	0
Epithelial cell	NDUFC1	0	0.864498518	0.8	0.201	0
Epithelial cell	PRDX1	0	0.86391696	0.853	0.377	0
Epithelial cell	NDUFB9	0	0.863045788	0.827	0.337	0
Epithelial cell	USH1C	0	0.86034538	0.609	0.006	0
Epithelial cell	CHCHD2	0	0.858121446	0.929	0.631	0
Epithelial cell	MGLL	0	0.857974552	0.587	0.043	0
Epithelial cell	CHCHD10	0	0.856735698	0.823	0.403	0
Epithelial cell	RAB13	0	0.856034263	0.627	0.028	0
Epithelial cell	TUBA1C	0	0.853124689	0.642	0.068	0
Epithelial cell	TPD52	0	0.845023321	0.807	0.216	0
Epithelial cell	JUP	0	0.844759001	0.608	0.016	0
Epithelial cell	TGFBI	0	0.839995857	0.468	0.07	0

Epithelial cell	CTNND1	0	0.838342714	0.65	0.034	0
Epithelial cell	ACTN4	0	0.837732458	0.814	0.182	0
Epithelial cell	CYP3A5	0	0.835893929	0.586	0.045	0
Epithelial cell	ATP8B1	0	0.830891835	0.58	0.012	0
Epithelial cell	EHF	0	0.82983856	0.572	0.004	0
Epithelial cell	SOD1	0	0.827883494	0.877	0.473	0
Epithelial cell	PLS1	0	0.826316598	0.561	0.007	0
Epithelial cell	LAD1	0	0.825984616	0.579	0.004	0
Epithelial cell	ATP5ME	0	0.825488494	0.917	0.564	0
Epithelial cell	GFPT1	0	0.825135705	0.655	0.055	0
Epithelial cell	SEM1	0	0.823439167	0.876	0.354	0
Epithelial cell	ATP5MF	0	0.821833191	0.891	0.466	0
Epithelial cell	TCIM	0	0.820147879	0.32	0.011	0
Epithelial cell	CST3	0	0.820071152	0.951	0.213	0
Epithelial cell	NDUFA4	0	0.82002808	0.888	0.605	0
Epithelial cell	CEACAM1	0	0.817782556	0.34	0.018	0
Epithelial cell	MSRB2	0	0.817531213	0.659	0.058	0
Epithelial cell	P4HB	0	0.815929706	0.826	0.319	0
Epithelial cell	TMEM98	0	0.815861921	0.584	0.009	0
Epithelial cell	PRR15L	0	0.814939842	0.554	0.007	0
Epithelial cell	TST	0	0.814838255	0.558	0.018	0
Epithelial cell	BRI3	0	0.81459014	0.719	0.122	0
Epithelial cell	DNAJC15	0	0.814438462	0.781	0.218	0
Epithelial cell	ASPH	0	0.814228849	0.611	0.041	0
Epithelial cell	PAWR	0	0.813714614	0.657	0.036	0
Epithelial cell	VDAC2	0	0.813651646	0.837	0.298	0
Epithelial cell	ZG16B	0	0.812174685	0.522	0.008	0
Epithelial cell	NDUFS5	0	0.811740282	0.86	0.428	0
Epithelial cell	IMPDH2	0	0.810833087	0.669	0.145	0
Epithelial cell	HNF4A	0	0.809741163	0.485	0.006	0
Epithelial cell	PPP1R14D	0	0.807323917	0.51	0.007	0
Epithelial cell	HIST1H4C	0	0.807193062	0.697	0.31	0
Epithelial cell	SPTBN1	0	0.804246523	0.66	0.061	0
Epithelial cell	PSMB5	0	0.802484506	0.699	0.129	0
Epithelial cell	TAX1BP3	0	0.802397998	0.637	0.065	0
Epithelial cell	TSPAN13	0	0.801930095	0.702	0.083	0
Epithelial cell	RBM47	0	0.799934049	0.655	0.062	0
Epithelial cell	KIF5B	0	0.799851434	0.796	0.257	0
Epithelial cell	TMPRSS2	0	0.799517684	0.522	0.007	0
Epithelial cell	NDUFS6	0	0.798284006	0.828	0.307	0
Epithelial cell	NDRG1	0	0.797449468	0.465	0.057	0
Epithelial cell	ELOB	0	0.79596915	0.944	0.611	0
Epithelial cell	ROMO1	0	0.794858955	0.844	0.313	0

Epithelial cell	MT2A	0	0.790338304	0.564	0.186	0
Epithelial cell	MRPL14	0	0.789849246	0.752	0.144	0
Epithelial cell	ERGIC3	0	0.789510763	0.801	0.251	0
Epithelial cell	EPHB2	0	0.789040117	0.518	0.005	0
Epithelial cell	PHB	0	0.789008014	0.748	0.198	0
Epithelial cell	PEBP1	0	0.788936134	0.822	0.381	0
Epithelial cell	UQCRFS1	0	0.786072477	0.782	0.254	0
Epithelial cell	ATP10B	0	0.784707748	0.546	0.006	0
Epithelial cell	KDELR1	0	0.784312094	0.752	0.17	0
Epithelial cell	FTH1	0	0.783934766	0.99	0.95	0
Epithelial cell	PRR15	0	0.782562883	0.503	0.004	0
Epithelial cell	LINC01133	0	0.781981925	0.367	0.006	0
Epithelial cell	HNRNPAB	0	0.779891803	0.761	0.186	0
Epithelial cell	RHOBTB3	0	0.776831724	0.524	0.014	0
Epithelial cell	CA9	0	0.776313206	0.253	0.001	0
Epithelial cell	ATP5F1C	0	0.775119086	0.829	0.342	0
Epithelial cell	TRPM4	0	0.774382678	0.516	0.013	0
Epithelial cell	PPIA	0	0.77255032	0.912	0.709	0
Epithelial cell	YWHAE	0	0.770145452	0.857	0.323	0
Epithelial cell	H3F3A	0	0.769420711	0.972	0.868	0
Epithelial cell	EPS8	0	0.768340823	0.587	0.039	0
Epithelial cell	S100A13	0	0.767869217	0.591	0.025	0
Epithelial cell	EI24	0	0.766739837	0.707	0.114	0
Epithelial cell	SOD3	0	0.766522153	0.4	0.011	0
Epithelial cell	NDUFB3	0	0.765813208	0.814	0.264	0
Epithelial cell	KLF3	0	0.764953853	0.644	0.062	0
Epithelial cell	AGPAT2	0	0.764859893	0.673	0.102	0
Epithelial cell	GGCT	0	0.764418907	0.633	0.087	0
Epithelial cell	ABHD11	0	0.763079793	0.608	0.042	0
Epithelial cell	LLGL2	0	0.761653264	0.635	0.049	0
Epithelial cell	UQCR10	0	0.761040203	0.883	0.446	0
Epithelial cell	RPLP0	0	0.760804997	0.936	0.894	0
Epithelial cell	SLC44A1	0	0.76006423	0.687	0.09	0
Epithelial cell	MIF	0	0.759910096	0.878	0.517	0
Epithelial cell	MYO6	0	0.75900396	0.629	0.051	0
Epithelial cell	ATF3	0	0.757212725	0.518	0.094	0
Epithelial cell	KDELR2	0	0.757041527	0.824	0.277	0
Epithelial cell	PGRMC1	0	0.755738444	0.647	0.06	0
Epithelial cell	MRPL12	0	0.755068334	0.679	0.122	0
Epithelial cell	ZNF703	0	0.752137429	0.545	0.014	0
Epithelial cell	NDUFB2	0	0.751983401	0.868	0.44	0
Epithelial cell	MCRIP2	0	0.751414264	0.669	0.092	0
Epithelial cell	RNF186	0	0.749997234	0.434	0.006	0

Epithelial cell	ATP5PD	0	0.749095125	0.815	0.29	0
Epithelial cell	PLCB4	0	0.747844653	0.487	0.004	0
Epithelial cell	RHOB	0	0.747509194	0.685	0.137	0
Epithelial cell	AC103702.2	0	0.746987569	0.592	0.035	0
Epithelial cell	AP1S1	0	0.746640115	0.673	0.084	0
Epithelial cell	PON2	0	0.744063732	0.584	0.03	0
Epithelial cell	CTNNA1	0	0.743858782	0.687	0.087	0
Epithelial cell	TPI1	0	0.740909022	0.905	0.571	0
Epithelial cell	AKR1C3	0	0.739716676	0.467	0.011	0
Epithelial cell	ANKRD36C	3.07E-266	0.739285356	0.299	0.125	7.14E-262
Epithelial cell	FABP2	0	0.735412103	0.327	0.006	0
Epithelial cell	LGALS2	0	0.733736522	0.313	0.052	0
Epithelial cell	HMGA1	0	0.732583228	0.706	0.189	0
Epithelial cell	CXCL3	0	0.732498845	0.397	0.036	0
Epithelial cell	HSBP1	0	0.732001231	0.818	0.217	0
Epithelial cell	DDT	0	0.731505939	0.78	0.273	0
Epithelial cell	COA3	0	0.731133759	0.729	0.156	0
Epithelial cell	TUBA1B	0	0.729786466	0.796	0.418	0
Epithelial cell	HOXB7	0	0.7255699	0.547	0.012	0
Epithelial cell	SLC39A4	0	0.72437375	0.575	0.053	0
Epithelial cell	TSPAN6	0	0.724330799	0.548	0.007	0
Epithelial cell	SDC4	0	0.724238974	0.522	0.015	0
Epithelial cell	AURKAIP1	0	0.723112748	0.841	0.302	0
Epithelial cell	RPL7A	0	0.723001195	0.941	0.9	0
Epithelial cell	MUC3A	0	0.722870116	0.518	0.004	0
Epithelial cell	SNRPF	0	0.719359741	0.786	0.278	0
Epithelial cell	SH3YL1	0	0.719145669	0.66	0.07	0
Epithelial cell	SUCLG1	0	0.717602225	0.707	0.16	0
Epithelial cell	AXIN2	0	0.714099253	0.431	0.006	0
Epithelial cell	KIAA1324	0	0.713115047	0.445	0.013	0
Epithelial cell	CFDP1	0	0.711345318	0.699	0.16	0
Epithelial cell	SHROOM3	0	0.710879087	0.509	0.004	0
Epithelial cell	COX7C	0	0.709969663	0.937	0.782	0
Epithelial cell	ECHS1	0	0.709839135	0.742	0.189	0
Epithelial cell	GIPC1	0	0.709319308	0.609	0.061	0
Epithelial cell	BAG1	0	0.707735646	0.73	0.151	0
Epithelial cell	CNN3	0	0.706723653	0.5	0.012	0
Epithelial cell	GNG5	0	0.704382824	0.856	0.327	0
Epithelial cell	BICDL2	0	0.704239508	0.526	0.006	0
Epithelial cell	PDCD5	0	0.704154142	0.703	0.147	0
Epithelial cell	CTBP2	0	0.703253287	0.595	0.028	0
Epithelial cell	ZFAS1	0	0.702878567	0.85	0.445	0
Epithelial cell	MAPK13	0	0.702629615	0.61	0.042	0

Epithelial cell	PTTG1IP	0	0.702463866	0.647	0.086	0
Epithelial cell	TINAGL1	0	0.702385909	0.509	0.006	0
Epithelial cell	SLC40A1	0	0.701920846	0.523	0.032	0
Epithelial cell	RPS27L	0	0.701686094	0.884	0.462	0
Epithelial cell	NAPRT	0	0.700286179	0.613	0.065	0
Epithelial cell	YBX3	0	0.700170216	0.708	0.117	0
Epithelial cell	TSTA3	0	0.698891366	0.691	0.142	0
Epithelial cell	TSPAN15	0	0.696433436	0.546	0.008	0
Epithelial cell	AHCY	0	0.696137121	0.56	0.056	0
Epithelial cell	GAS6	0	0.695075566	0.526	0.036	0
Epithelial cell	SRPK1	0	0.69248679	0.638	0.095	0
Epithelial cell	AFDN	0	0.691034928	0.574	0.023	0
Epithelial cell	EGR1	0	0.690638082	0.659	0.136	0
Epithelial cell	PKP2	0	0.686320267	0.518	0.009	0
Epithelial cell	NENF	0	0.684969387	0.733	0.189	0
Epithelial cell	EBPL	0	0.683805811	0.652	0.108	0
Epithelial cell	MGAT4B	0	0.683778126	0.554	0.024	0
Epithelial cell	JPT1	0	0.683203312	0.734	0.229	0
Epithelial cell	EDN1	0	0.682143209	0.293	0.004	0
Epithelial cell	SQSTM1	0	0.681851446	0.781	0.251	0
Epithelial cell	C1QBP	0	0.678357279	0.729	0.246	0
Epithelial cell	CXCL1	0	0.677102394	0.253	0.018	0
Epithelial cell	SELENOW	0	0.67600911	0.811	0.245	0
Epithelial cell	MZT2B	0	0.675258714	0.858	0.416	0
Epithelial cell	CDCA7	0	0.675020243	0.446	0.023	0
Epithelial cell	EFNA1	0	0.674454653	0.438	0.009	0
Epithelial cell	CREB3L1	0	0.673800107	0.419	0.003	0
Epithelial cell	SNRPE	0	0.672864691	0.799	0.316	0
Epithelial cell	MYO10	0	0.670516948	0.516	0.005	0
Epithelial cell	SERPINB1	0	0.669772123	0.787	0.258	0
Epithelial cell	DCTPP1	0	0.669379075	0.646	0.096	0
Epithelial cell	NDUFB7	0	0.669091277	0.834	0.348	0
Epithelial cell	ASL	0	0.668444252	0.594	0.059	0
Epithelial cell	ATP5F1D	0	0.666852383	0.873	0.508	0
Epithelial cell	MLXIP	0	0.666596246	0.649	0.095	0
Epithelial cell	HOXB9	0	0.665808322	0.482	0.005	0
Epithelial cell	ZKSCAN1	0	0.665250771	0.66	0.113	0
Epithelial cell	DDR1	0	0.665182145	0.525	0.011	0
Epithelial cell	PLBD1	0	0.664497002	0.564	0.048	0
Epithelial cell	ATP5PO	0	0.662276895	0.852	0.446	0
Epithelial cell	CA12	0	0.661991948	0.46	0.006	0
Epithelial cell	SDC1	0	0.661658053	0.554	0.044	0
Epithelial cell	CD59	0	0.65973583	0.622	0.093	0

Epithelial cell	TCF7L2	0	0.658498476	0.504	0.018	0
Epithelial cell	TMEM59	0	0.65821256	0.915	0.542	0
Epithelial cell	SMAGP	0	0.656317699	0.616	0.049	0
Epithelial cell	CISD1	0	0.656197162	0.632	0.086	0
Epithelial cell	HSD11B2	0	0.651003321	0.447	0.008	0
Epithelial cell	SMIM31	0	0.649688531	0.402	0.002	0
Epithelial cell	AREG	0	0.649555985	0.474	0.131	0
Epithelial cell	CHP1	0	0.649195419	0.666	0.125	0
Epithelial cell	SATB2	0	0.647643525	0.448	0.006	0
Epithelial cell	EPS8L3	0	0.647484529	0.496	0.007	0
Epithelial cell	RND3	0	0.646494452	0.366	0.004	0
Epithelial cell	HDGF	0	0.645779339	0.617	0.08	0
Epithelial cell	CLDN2	0	0.644105752	0.319	0.002	0
Epithelial cell	ACSL5	0	0.643105849	0.628	0.07	0
Epithelial cell	HMGB3	0	0.641746159	0.453	0.015	0
Epithelial cell	QSOX1	0	0.64169113	0.486	0.058	0
Epithelial cell	ADI1	0	0.641681403	0.674	0.127	0
Epithelial cell	TOB1	0	0.641357673	0.622	0.079	0
Epithelial cell	EIF6	0	0.640691154	0.738	0.21	0
Epithelial cell	ATP5PB	0	0.639039178	0.799	0.327	0
Epithelial cell	MACROD1	0	0.636301102	0.5	0.016	0
Epithelial cell	FCGRT	0	0.636263807	0.736	0.166	0
Epithelial cell	FAM177A1	0	0.636173633	0.738	0.178	0
Epithelial cell	CCL15	0	0.63605657	0.416	0.005	0
Epithelial cell	FLNB	0	0.634278441	0.467	0.016	0
Epithelial cell	TCEAL9	0	0.634150576	0.462	0.022	0
Epithelial cell	RAB11A	0	0.633178972	0.765	0.208	0
Epithelial cell	BCAS1	0	0.633064225	0.356	0.006	0
Epithelial cell	FAM213A	0	0.632530086	0.517	0.031	0
Epithelial cell	STOML2	0	0.632276955	0.697	0.159	0
Epithelial cell	TDP2	0	0.632242575	0.615	0.104	0
Epithelial cell	LRRC26	0	0.63184074	0.262	0.004	0
Epithelial cell	IDH1	0	0.629801467	0.579	0.06	0
Epithelial cell	MAOA	0	0.629512748	0.448	0.009	0
Epithelial cell	EPB41L2	0	0.628185273	0.496	0.042	0
Epithelial cell	NFIB	0	0.627897808	0.478	0.006	0
Epithelial cell	MRPS33	0	0.627456177	0.701	0.156	0
Epithelial cell	REPIN1	0	0.627437638	0.59	0.092	0
Epithelial cell	DNPH1	0	0.626991103	0.702	0.174	0
Epithelial cell	CISD3	0	0.626971882	0.704	0.16	0
Epithelial cell	POF1B	0	0.625066389	0.49	0.004	0
Epithelial cell	MAP7	0	0.624713033	0.524	0.007	0
Epithelial cell	NME1	0	0.623127664	0.615	0.142	0

Epithelial cell	PARM1	0	0.622175037	0.412	0.026	0
Epithelial cell	STXBP6	0	0.620468813	0.488	0.005	0
Epithelial cell	CYCS	0	0.619457239	0.844	0.447	0
Epithelial cell	RAB2A	0	0.6190128	0.807	0.263	0
Epithelial cell	SNORC	0	0.618039889	0.439	0.005	0
Epithelial cell	HIGD1A	0	0.617915611	0.659	0.123	0
Epithelial cell	PKM	0	0.617160032	0.803	0.397	0
Epithelial cell	PPP1R16A	0	0.614341032	0.522	0.023	0
Epithelial cell	TIMM8B	0	0.614212806	0.733	0.189	0
Epithelial cell	RHOC	0	0.613783667	0.804	0.274	0
Epithelial cell	INSR	0	0.613587033	0.437	0.033	0
Epithelial cell	OCIAD2	0	0.613424575	0.809	0.29	0
Epithelial cell	CERS6	0	0.613017385	0.542	0.03	0
Epithelial cell	NDUFA6	0	0.612319044	0.76	0.231	0
Epithelial cell	MTCH2	0	0.61224693	0.665	0.131	0
Epithelial cell	IFT172	0	0.611864983	0.498	0.012	0
Epithelial cell	HEPH	0	0.611609814	0.472	0.006	0
Epithelial cell	FDPS	0	0.610428798	0.644	0.189	0
Epithelial cell	GNG12	0	0.610265716	0.48	0.005	0
Epithelial cell	NFIA	0	0.609899559	0.531	0.019	0
Epithelial cell	BCL2L15	0	0.60969431	0.47	0.005	0
Epithelial cell	GALE	0	0.609021868	0.52	0.033	0
Epithelial cell	SPINT1-AS1	0	0.608650431	0.51	0.013	0
Epithelial cell	POMP	0	0.608153796	0.877	0.457	0
Epithelial cell	IFI6	0	0.60673236	0.357	0.137	0
Epithelial cell	PLAC8	0	0.605138584	0.419	0.189	0
Epithelial cell	H2AFY	0	0.604804145	0.806	0.254	0
Epithelial cell	ESRP1	0	0.604477677	0.504	0.004	0
Epithelial cell	SH3BGRL2	0	0.603843711	0.48	0.004	0
Epithelial cell	TMED3	0	0.603120798	0.64	0.11	0
Epithelial cell	NDUFB10	0	0.60258042	0.828	0.328	0
Epithelial cell	PXMP2	0	0.600037391	0.5	0.024	0
Epithelial cell	FNBP1L	0	0.597662922	0.486	0.005	0
Epithelial cell	CD2AP	0	0.592143913	0.593	0.063	0
Epithelial cell	LAPTM4B	0	0.591211846	0.479	0.022	0
Epithelial cell	ACOT13	0	0.590800684	0.56	0.048	0
Epithelial cell	APIP	0	0.59068945	0.521	0.081	0
Epithelial cell	PTMS	0	0.590528182	0.655	0.139	0
Epithelial cell	HSD17B12	0	0.589864774	0.655	0.129	0
Epithelial cell	PRDX4	0	0.588918508	0.668	0.136	0
Epithelial cell	NTHL1	0	0.588614098	0.488	0.043	0
Epithelial cell	AKAP1	0	0.587617003	0.54	0.032	0
Epithelial cell	TMC4	0	0.586279878	0.473	0.006	0

Epithelial cell	CLRN3	0	0.586087792	0.452	0.004	0
Epithelial cell	COX20	0	0.586087406	0.716	0.175	0
Epithelial cell	DDAH1	0	0.585958003	0.49	0.004	0
Epithelial cell	TKT	0	0.585077137	0.733	0.218	0
Epithelial cell	ANG	0	0.583144849	0.469	0.023	0
Epithelial cell	FOXA3	0	0.583026863	0.431	0.003	0
Epithelial cell	DDAH2	0	0.582909052	0.634	0.135	0
Epithelial cell	LSM4	0	0.582713702	0.736	0.224	0
Epithelial cell	FUT3	0	0.581542053	0.441	0.006	0
Epithelial cell	HSP90AB1	0	0.581282745	0.882	0.607	0
Epithelial cell	TRABD2A	0	0.581223111	0.484	0.017	0
Epithelial cell	REXO2	0	0.581005717	0.637	0.125	0
Epithelial cell	DMAC1	0	0.580958378	0.692	0.151	0
Epithelial cell	SUCLG2	0	0.580131061	0.69	0.162	0
Epithelial cell	LAMB3	0	0.577645202	0.364	0.006	0
Epithelial cell	TMPRSS4	0	0.577364145	0.428	0.005	0
Epithelial cell	GALNT3	0	0.577210871	0.526	0.026	0
Epithelial cell	HADH	0	0.576578584	0.555	0.058	0
Epithelial cell	CENPW	0	0.576390012	0.317	0.012	0
Epithelial cell	MUC5B	0	0.572926234	0.338	0.007	0
Epithelial cell	C9orf152	0	0.571881357	0.464	0.005	0
Epithelial cell	SCNN1A	0	0.571715748	0.328	0.005	0
Epithelial cell	CFTR	0	0.571601551	0.429	0.004	0
Epithelial cell	MT1F	0	0.570447109	0.311	0.069	0
Epithelial cell	FAT1	0	0.570441772	0.454	0.004	0
Epithelial cell	RPL37A	0	0.567950054	0.967	0.942	0
Epithelial cell	ALDH1A1	0	0.567631908	0.328	0.02	0
Epithelial cell	BIK	0	0.566811673	0.474	0.039	0
Epithelial cell	EPHB3	0	0.563349635	0.346	0.005	0
Epithelial cell	MRPL13	0	0.563034914	0.658	0.131	0
Epithelial cell	SNHG18	0	0.562725598	0.468	0.005	0
Epithelial cell	IDH2	0	0.562696363	0.7	0.27	0
Epithelial cell	CAMTA1	0	0.561134534	0.734	0.174	0
Epithelial cell	PLA2G10	0	0.560658873	0.3	0.004	0
Epithelial cell	USP53	0	0.558020307	0.473	0.038	0
Epithelial cell	RABL6	0	0.556680836	0.596	0.087	0
Epithelial cell	TGIF1	0	0.556591745	0.606	0.095	0
Epithelial cell	TUFM	0	0.556156632	0.785	0.335	0
Epithelial cell	RAN	0	0.555877992	0.829	0.478	0
Epithelial cell	PMEPA1	0	0.555667526	0.423	0.041	0
Epithelial cell	CENPX	0	0.555160497	0.64	0.136	0
Epithelial cell	IRF2BP2	0	0.554669857	0.701	0.155	0
Epithelial cell	CKAP4	0	0.55401841	0.495	0.048	0

Epithelial cell	NDUFA12	0	0.553822043	0.761	0.24	0
Epithelial cell	ASRGL1	0	0.553780079	0.402	0.013	0
Epithelial cell	MYC	0	0.552846413	0.487	0.071	0
Epithelial cell	ISOC2	0	0.552060647	0.589	0.089	0
Epithelial cell	GGT6	0	0.552046839	0.435	0.005	0
Epithelial cell	HOOK2	0	0.551215705	0.546	0.048	0
Epithelial cell	CKS2	0	0.549768407	0.504	0.094	0
Epithelial cell	LDLR	0	0.548538457	0.425	0.039	0
Epithelial cell	ITGB4	0	0.548372075	0.441	0.008	0
Epithelial cell	C1orf21	0	0.547586583	0.481	0.03	0
Epithelial cell	NEDD4L	0	0.547403795	0.394	0.012	0
Epithelial cell	HIST1H1C	0	0.547221342	0.356	0.086	0
Epithelial cell	H2AFZ	3.99E-267	0.545464384	0.815	0.512	9.27E-263
Epithelial cell	HOXA10	0	0.544452819	0.444	0.006	0
Epithelial cell	SNRPG	0	0.54430858	0.845	0.411	0
Epithelial cell	HDHD3	0	0.544006508	0.557	0.051	0
Epithelial cell	MT-ND4L	0	0.543979185	0.862	0.394	0
Epithelial cell	IGFBP4	0	0.543642891	0.499	0.018	0
Epithelial cell	NECTIN2	0	0.543453284	0.455	0.019	0
Epithelial cell	NDUFA8	0	0.542634226	0.647	0.113	0
Epithelial cell	PPARG	0	0.542318094	0.401	0.005	0
Epithelial cell	NDUFS4	0	0.541783578	0.686	0.141	0
Epithelial cell	ACTN1	0	0.540384782	0.547	0.041	0
Epithelial cell	UQCRC2	0	0.5397753	0.758	0.277	0
Epithelial cell	APLP2	0	0.539316626	0.739	0.17	0
Epithelial cell	HDLBP	0	0.5389513	0.691	0.155	0
Epithelial cell	NOXO1	0	0.538628465	0.399	0.003	0
Epithelial cell	ECI1	0	0.537162239	0.538	0.048	0
Epithelial cell	MVP	0	0.536224041	0.659	0.14	0
Epithelial cell	ABHD17C	0	0.535707346	0.445	0.005	0
Epithelial cell	SQLE	0	0.534527793	0.399	0.062	0
Epithelial cell	MYB	0	0.534241081	0.441	0.007	0
Epithelial cell	CKMT1A	0	0.534030795	0.388	0.004	0
Epithelial cell	CD46	0	0.534026358	0.725	0.187	0
Epithelial cell	IMPA2	0	0.533298181	0.484	0.028	0
Epithelial cell	SLC35A3	0	0.532896694	0.625	0.087	0
Epithelial cell	CCT5	0	0.531567104	0.672	0.165	0
Epithelial cell	CAST	0	0.531395062	0.827	0.287	0
Epithelial cell	GDE1	0	0.530129755	0.585	0.069	0
Epithelial cell	BAIAP2L1	0	0.529891693	0.42	0.005	0
Epithelial cell	C4orf48	0	0.528464258	0.692	0.253	0
Epithelial cell	CANX	0	0.52784405	0.779	0.272	0
Epithelial cell	MARVELD3	0	0.525516026	0.433	0.003	0

Epithelial cell	ACAA2	0	0.524912944	0.583	0.111	0
Epithelial cell	SLC25A6	0	0.52482206	0.902	0.743	0
Epithelial cell	CENPV	0	0.524224651	0.525	0.038	0
Epithelial cell	TENT5A	0	0.524214727	0.37	0.054	0
Epithelial cell	ERBB2	0	0.523814975	0.46	0.009	0
Epithelial cell	MAP3K13	0	0.523146742	0.598	0.075	0
Epithelial cell	UGDH	0	0.522988286	0.489	0.055	0
Epithelial cell	TRIB1	0	0.522032563	0.453	0.063	0
Epithelial cell	NDUFS3	0	0.521793163	0.669	0.15	0
Epithelial cell	SLC6A8	0	0.521176366	0.251	0.004	0
Epithelial cell	NDUFA2	0	0.521131799	0.799	0.286	0
Epithelial cell	ZNHIT1	0	0.519538131	0.805	0.277	0
Epithelial cell	CMAS	0	0.519464991	0.603	0.091	0
Epithelial cell	C6orf132	0	0.51826555	0.421	0.004	0
Epithelial cell	ENC1	0	0.517210201	0.394	0.01	0
Epithelial cell	ROCK2	0	0.517168068	0.546	0.049	0
Epithelial cell	BOLA3	0	0.51700553	0.582	0.086	0
Epithelial cell	NCKAP1	0	0.516856749	0.471	0.007	0
Epithelial cell	PPA2	0	0.516307084	0.643	0.135	0
Epithelial cell	RAB11FIP1	0	0.516000882	0.7	0.177	0
Epithelial cell	ACADVL	0	0.514833757	0.709	0.209	0
Epithelial cell	CHP2	0	0.514104043	0.305	0.005	0
Epithelial cell	EBP	0	0.513906197	0.599	0.139	0
Epithelial cell	F11R	0	0.513859927	0.467	0.025	0
Epithelial cell	ALKBH7	0	0.512908101	0.697	0.186	0
Epithelial cell	CASP6	0	0.512674876	0.501	0.031	0
Epithelial cell	YBX1	0	0.512063057	0.913	0.768	0
Epithelial cell	TJP3	0	0.511428881	0.419	0.016	0
Epithelial cell	ARF4	0	0.511376393	0.728	0.205	0
Epithelial cell	PSMA7	0	0.510720659	0.872	0.539	0
Epithelial cell	PTGR1	0	0.51068674	0.399	0.008	0
Epithelial cell	ETFB	0	0.510128496	0.748	0.241	0
Epithelial cell	PYCR1	0	0.509645817	0.43	0.023	0
Epithelial cell	CANT1	0	0.509186478	0.528	0.042	0
Epithelial cell	SLC44A3	0	0.508890179	0.444	0.004	0
Epithelial cell	SEC61G	0	0.508821284	0.876	0.473	0
Epithelial cell	RPS24	0	0.507874607	0.958	0.95	0
Epithelial cell	SERINC5	0	0.507503611	0.606	0.075	0
Epithelial cell	CHMP3	0	0.507091769	0.698	0.137	0
Epithelial cell	NACA	0	0.50637019	0.925	0.832	0
Epithelial cell	CHMP4C	0	0.506324337	0.429	0.005	0
Epithelial cell	SNX7	0	0.506032974	0.463	0.007	0
Epithelial cell	EFNB2	0	0.505807731	0.391	0.003	0

Epithelial cell	HTATIP2	0	0.505352968	0.634	0.112	0
Epithelial cell	QARS	0	0.504662954	0.669	0.143	0
Epithelial cell	SLC39A5	0	0.504252082	0.387	0.006	0
Epithelial cell	PFKL	0	0.504030916	0.62	0.105	0
Epithelial cell	ATP2C2	0	0.503951775	0.423	0.004	0
Epithelial cell	HOXA9	0	0.503315196	0.398	0.004	0
Epithelial cell	PDXDC1	0	0.503079675	0.581	0.069	0
Epithelial cell	PYGB	0	0.502734101	0.451	0.038	0
Epithelial cell	NHP2	0	0.502678532	0.732	0.236	0
Epithelial cell	SULT1B1	0	0.502659157	0.342	0.009	0
Epithelial cell	C12orf75	0	0.502505426	0.663	0.187	0
Epithelial cell	ARL14	0	0.50231394	0.299	0.009	0
Epithelial cell	ANKRD9	0	0.502277607	0.426	0.012	0
Epithelial cell	MLPH	0	0.502074928	0.361	0.005	0
Epithelial cell	NDUFB6	0	0.501868404	0.696	0.176	0
Epithelial cell	HOXB6	0	0.501521434	0.387	0.004	0
Epithelial cell	FAM120A	0	0.50056639	0.687	0.145	0
Epithelial cell	CRB3	0	0.500214144	0.439	0.007	0
Epithelial cell	MECOM	0	0.499524316	0.429	0.003	0
Epithelial cell	CRYL1	0	0.499487066	0.514	0.056	0
Epithelial cell	HNMT	0	0.499336862	0.536	0.053	0
Epithelial cell	SQOR	0	0.499186889	0.596	0.113	0
Epithelial cell	SCARB2	0	0.498057941	0.556	0.076	0
Epithelial cell	GALNT7	0	0.497692856	0.492	0.032	0
Epithelial cell	BSPRY	0	0.49713199	0.432	0.003	0
Epithelial cell	NR2F6	0	0.494904155	0.441	0.014	0
Epithelial cell	SPTSSA	0	0.494722105	0.618	0.118	0
Epithelial cell	GMNN	0	0.49463822	0.41	0.032	0
Epithelial cell	CCT6A	0	0.494405404	0.709	0.224	0
Epithelial cell	GPI	0	0.49426243	0.624	0.127	0
Epithelial cell	SYNGR2	0	0.493799111	0.726	0.214	0
Epithelial cell	PAFAH1B3	0	0.49247054	0.539	0.074	0
Epithelial cell	ATP5MPL	0	0.492397038	0.898	0.59	0
Epithelial cell	SORD	0	0.492220559	0.422	0.015	0
Epithelial cell	DYNLT1	0	0.491575649	0.799	0.26	0
Epithelial cell	PRDX6	0	0.491053382	0.755	0.281	0
Epithelial cell	OLA1	0	0.489334241	0.637	0.137	0
Epithelial cell	BLVRB	0	0.487487043	0.666	0.123	0
Epithelial cell	ZNF706	0	0.487462013	0.792	0.314	0
Epithelial cell	PROM1	0	0.48738782	0.348	0.003	0
Epithelial cell	WASL	0	0.487284501	0.505	0.035	0
Epithelial cell	GLUL	0	0.487284494	0.684	0.106	0
Epithelial cell	INAVA	0	0.487172986	0.371	0.008	0

Epithelial cell	ERN2	0	0.486512327	0.38	0.005	0
Epithelial cell	CKS1B	0	0.486053999	0.46	0.076	0
Epithelial cell	NDUFA10	0	0.485661055	0.676	0.158	0
Epithelial cell	HSBP1L1	0	0.485035984	0.467	0.027	0
Epithelial cell	ALDH1B1	0	0.484719908	0.393	0.014	0
Epithelial cell	ZDHHC3	0	0.484466894	0.554	0.061	0
Epithelial cell	TALDO1	0	0.484284274	0.756	0.253	0
Epithelial cell	CLCN3	0	0.484042592	0.526	0.05	0
Epithelial cell	MYDGF	0	0.482630897	0.741	0.258	0
Epithelial cell	KIAA1522	0	0.481601624	0.415	0.003	0
Epithelial cell	CHMP2B	0	0.480368683	0.645	0.133	0
Epithelial cell	SPR	0	0.480045966	0.442	0.008	0
Epithelial cell	MYO5B	0	0.480015012	0.412	0.005	0
Epithelial cell	PLEK2	0	0.479917709	0.414	0.006	0
Epithelial cell	SLC22A18	0	0.479257226	0.491	0.052	0
Epithelial cell	LAPTM4A	0	0.478894813	0.792	0.313	0
Epithelial cell	TRIP6	0	0.47858758	0.426	0.025	0
Epithelial cell	MRPL41	0	0.477915624	0.76	0.266	0
Epithelial cell	PDCD6	0	0.477757084	0.765	0.252	0
Epithelial cell	UCHL3	0	0.477410593	0.554	0.077	0
Epithelial cell	MYO1D	0	0.477334167	0.431	0.015	0
Epithelial cell	KCNK1	0	0.476692136	0.354	0.003	0
Epithelial cell	HSP90B1	0	0.475623069	0.856	0.448	0
Epithelial cell	EIF5A	0	0.475375066	0.721	0.313	0
Epithelial cell	DHRS11	0	0.47510634	0.336	0.015	0
Epithelial cell	PTRHD1	0	0.474453173	0.637	0.118	0
Epithelial cell	CEBPG	0	0.473857405	0.521	0.064	0
Epithelial cell	ACADS	0	0.471524474	0.49	0.048	0
Epithelial cell	RPN2	0	0.471044677	0.709	0.237	0
Epithelial cell	PDXK	0	0.470737535	0.545	0.107	0
Epithelial cell	LPP	0	0.470654327	0.665	0.121	0
Epithelial cell	CCT2	0	0.469298469	0.657	0.179	0
Epithelial cell	MPZL1	0	0.469169648	0.512	0.043	0
Epithelial cell	NME4	0	0.469038729	0.504	0.07	0
Epithelial cell	CDS1	0	0.468762866	0.39	0.003	0
Epithelial cell	MAPK3	0	0.468338584	0.474	0.048	0
Epithelial cell	PPP2R1A	0	0.468231671	0.68	0.158	0
Epithelial cell	PLEKHJ1	0	0.466675859	0.694	0.168	0
Epithelial cell	NXPE4	0	0.465651609	0.267	0.005	0
Epithelial cell	SAP18	0	0.465176526	0.862	0.434	0
Epithelial cell	NDUFV2	0	0.464786574	0.772	0.287	0
Epithelial cell	CTNNB1	0	0.464786346	0.718	0.217	0
Epithelial cell	EGLN3	0	0.464502941	0.291	0.003	0

Epithelial cell	PRELID3B	0	0.464381503	0.608	0.125	0
Epithelial cell	CHMP2A	0	0.463786889	0.797	0.274	0
Epithelial cell	RAB1A	0	0.463742719	0.725	0.2	0
Epithelial cell	UQCC3	0	0.463729318	0.594	0.092	0
Epithelial cell	CAPN8	0	0.462483909	0.315	0.004	0
Epithelial cell	PLP2	0	0.462202551	0.735	0.275	0
Epithelial cell	PDHA1	0	0.461348971	0.575	0.094	0
Epithelial cell	TMBIM1	0	0.460935914	0.498	0.082	0
Epithelial cell	ARHGEF35	0	0.460783594	0.415	0.01	0
Epithelial cell	NDUFV1	0	0.460639466	0.662	0.174	0
Epithelial cell	VEGFA	0	0.460415062	0.415	0.036	0
Epithelial cell	SMPDL3A	0	0.460231963	0.301	0.016	0
Epithelial cell	RARRES1	0	0.45989675	0.316	0.005	0
Epithelial cell	CDC42EP1	0	0.459550522	0.41	0.009	0
Epithelial cell	RAB4A	0	0.45944777	0.645	0.132	0
Epithelial cell	AKR7A3	0	0.458779381	0.384	0.003	0
Epithelial cell	TMBIM6	0	0.458365703	0.922	0.57	0
Epithelial cell	MT1X	0	0.458082349	0.587	0.157	0
Epithelial cell	C8orf33	0	0.457842656	0.47	0.089	0
Epithelial cell	TSTD1	0	0.457814691	0.803	0.295	0
Epithelial cell	MRPL57	0	0.457622519	0.757	0.252	0
Epithelial cell	FBLIM1	0	0.456929869	0.384	0.007	0
Epithelial cell	CBLC	0	0.456825924	0.405	0.003	0
Epithelial cell	NDRG2	0	0.455850066	0.45	0.026	0
Epithelial cell	COA4	0	0.455655046	0.614	0.107	0
Epithelial cell	FIS1	0	0.455307401	0.771	0.27	0
Epithelial cell	MRPL32	0	0.455036383	0.604	0.102	0
Epithelial cell	SH3D19	0	0.454549273	0.418	0.005	0
Epithelial cell	EDF1	0	0.453720582	0.935	0.608	0
Epithelial cell	FKBP4	0	0.453393453	0.536	0.073	0
Epithelial cell	TM4SF5	0	0.453243362	0.268	0.003	0
Epithelial cell	FAM13A	0	0.452693291	0.314	0.021	0
Epithelial cell	TNFSF10	0	0.452364513	0.577	0.101	0
Epithelial cell	ESRRA	0	0.45179961	0.516	0.051	0
Epithelial cell	ATP5F1A	0	0.45118166	0.772	0.373	0
Epithelial cell	TBX3	0	0.450370482	0.301	0.002	0
Epithelial cell	MRPS35	0	0.448589388	0.594	0.105	0
Epithelial cell	LRRC59	0	0.448115103	0.552	0.11	0
Epithelial cell	CAPNS1	0	0.447686441	0.662	0.14	0
Epithelial cell	MIR194-2HG	0	0.446804003	0.294	0.007	0
Epithelial cell	COMT	0	0.44490924	0.61	0.138	0
Epithelial cell	PTPRK	0	0.443937494	0.436	0.013	0
Epithelial cell	PLGRKT	0	0.44247209	0.587	0.097	0

Epithelial cell	CKMT1B	0	0.442453839	0.365	0.004	0
Epithelial cell	NEBL	0	0.442445183	0.368	0.004	0
Epithelial cell	HIGD2A	0	0.442110096	0.844	0.391	0
Epithelial cell	CLIC1	0	0.441846184	0.935	0.695	0
Epithelial cell	TOX3	0	0.441800645	0.379	0.002	0
Epithelial cell	AZGP1	0	0.441693004	0.255	0.003	0
Epithelial cell	GJB1	0	0.441555506	0.407	0.003	0
Epithelial cell	LGR4	0	0.441280347	0.374	0.004	0
Epithelial cell	LAMTOR5	0	0.441128714	0.82	0.332	0
Epithelial cell	FUCA1	0	0.44067768	0.533	0.077	0
Epithelial cell	IGF2BP2	0	0.439984714	0.372	0.008	0
Epithelial cell	ARFGEF3	0	0.43970856	0.37	0.003	0
Epithelial cell	GNA11	0	0.439391829	0.349	0.006	0
Epithelial cell	PDZD8	0	0.43929519	0.497	0.047	0
Epithelial cell	SYAP1	0	0.4382149	0.681	0.148	0
Epithelial cell	EPN1	0	0.437739247	0.578	0.086	0
Epithelial cell	WDR34	0	0.437174861	0.383	0.029	0
Epithelial cell	LTBR	0	0.435829013	0.438	0.025	0
Epithelial cell	CAPN5	0	0.435243449	0.345	0.006	0
Epithelial cell	POLR2I	0	0.434397484	0.705	0.186	0
Epithelial cell	PHPT1	0	0.432619649	0.772	0.281	0
Epithelial cell	DEGS2	0	0.43252346	0.41	0.01	0
Epithelial cell	F2RL1	0	0.43242444	0.319	0.004	0
Epithelial cell	SLC9A3R2	0	0.431954153	0.441	0.035	0
Epithelial cell	TMEM258	0	0.430844312	0.872	0.478	0
Epithelial cell	MRPL23	0	0.430514526	0.629	0.139	0
Epithelial cell	ARHGAP32	0	0.430239653	0.398	0.006	0
Epithelial cell	GTF3C6	0	0.429529944	0.676	0.175	0
Epithelial cell	POLR1D	0	0.42943746	0.754	0.279	0
Epithelial cell	MRPL15	0	0.429124062	0.544	0.08	0
Epithelial cell	GCSH	0	0.429078608	0.508	0.066	0
Epithelial cell	RGMB	0	0.428974841	0.298	0.009	0
Epithelial cell	MTMR11	0	0.428285143	0.348	0.017	0
Epithelial cell	RASEF	0	0.42823947	0.31	0.003	0
Epithelial cell	TP53I3	0	0.426966843	0.406	0.021	0
Epithelial cell	HOOK1	0	0.426544368	0.423	0.017	0
Epithelial cell	ABO	0	0.425982467	0.387	0.008	0
Epithelial cell	RAB5IF	0	0.425837391	0.691	0.245	0
Epithelial cell	KCNN4	0	0.425306253	0.469	0.045	0
Epithelial cell	TFRC	0	0.424484667	0.502	0.074	0
Epithelial cell	CDKN1A	0	0.424481131	0.392	0.075	0
Epithelial cell	PAICS	0	0.424472962	0.459	0.052	0
Epithelial cell	MYL6	0	0.424439537	0.977	0.838	0

Epithelial cell	AK3	0	0.424390761	0.611	0.115	0
Epithelial cell	ACSS2	0	0.423759304	0.348	0.015	0
Epithelial cell	ITGA2	0	0.423199416	0.366	0.005	0
Epithelial cell	HIST1H2AC	0	0.422910597	0.359	0.066	0
Epithelial cell	ALDH7A1	0	0.422831997	0.395	0.008	0
Epithelial cell	PDGFA	0	0.422814046	0.267	0.004	0
Epithelial cell	RNASE1	0	0.422577831	0.39	0.04	0
Epithelial cell	GNAQ	0	0.422367196	0.521	0.061	0
Epithelial cell	GLRX5	0	0.422302362	0.598	0.115	0
Epithelial cell	ATP5MG	0	0.421778046	0.931	0.771	0
Epithelial cell	ADAM15	0	0.421738607	0.428	0.035	0
Epithelial cell	PIGT	0	0.421710822	0.63	0.129	0
Epithelial cell	OCLN	0	0.421378968	0.368	0.011	0
Epithelial cell	LSM7	0	0.420573125	0.813	0.356	0
Epithelial cell	FOXA2	0	0.419913713	0.322	0.002	0
Epithelial cell	RPL8	0	0.419467447	0.975	0.958	0
Epithelial cell	BZW2	0	0.419082079	0.544	0.079	0
Epithelial cell	ZDHHC12	0	0.41900257	0.613	0.122	0
Epithelial cell	CRACR2B	0	0.418798454	0.443	0.04	0
Epithelial cell	YIF1A	0	0.418505974	0.647	0.155	0
Epithelial cell	TMEM125	0	0.418091082	0.376	0.004	0
Epithelial cell	TRAP1	0	0.417625929	0.47	0.05	0
Epithelial cell	TJP1	0	0.417393407	0.354	0.003	0
Epithelial cell	ICA1	0	0.417189985	0.466	0.029	0
Epithelial cell	PLXNB2	0	0.417006414	0.406	0.033	0
Epithelial cell	FUT2	0	0.416615457	0.375	0.006	0
Epithelial cell	SDHB	0	0.416495812	0.614	0.135	0
Epithelial cell	MRPL21	0	0.416289649	0.592	0.106	0
Epithelial cell	PLEKHA5	0	0.416028021	0.397	0.012	0
Epithelial cell	CDC42BPA	0	0.415356585	0.345	0.005	0
Epithelial cell	ATP9A	0	0.415108886	0.361	0.005	0
Epithelial cell	SSFA2	0	0.414831116	0.451	0.047	0
Epithelial cell	DGAT1	0	0.414505203	0.425	0.027	0
Epithelial cell	ENO1	0	0.41442949	0.827	0.517	0
Epithelial cell	FRYL	0	0.414328225	0.563	0.094	0
Epithelial cell	SNRPD1	0	0.413759164	0.725	0.269	0
Epithelial cell	MRPS16	0	0.413666713	0.63	0.134	0
Epithelial cell	RFK	0	0.413368012	0.49	0.075	0
Epithelial cell	PDIA4	0	0.412710708	0.707	0.216	0
Epithelial cell	CNPY2	0	0.412332024	0.702	0.205	0
Epithelial cell	NOX1	0	0.412130743	0.331	0.005	0
Epithelial cell	RNF7	0	0.411843717	0.757	0.245	0
Epithelial cell	MRPS34	0	0.411690087	0.727	0.243	0

Epithelial cell	CTTNBP2NL	0	0.410073069	0.373	0.017	0
Epithelial cell	CAPN1	0	0.410028197	0.635	0.135	0
Epithelial cell	ANAPC11	0	0.409885976	0.771	0.307	0
Epithelial cell	CHCHD3	0	0.409690325	0.523	0.075	0
Epithelial cell	BLOC1S1	0	0.409688996	0.855	0.38	0
Epithelial cell	RANBP1	0	0.409115273	0.722	0.32	0
Epithelial cell	NUDT4	0	0.408678602	0.654	0.16	0
Epithelial cell	ITGB1	0	0.408289465	0.733	0.181	0
Epithelial cell	ZNF511	0	0.408196308	0.579	0.106	0
Epithelial cell	NDFIP2	0	0.407958431	0.594	0.095	0
Epithelial cell	VSIG10	0	0.407752716	0.385	0.012	0
Epithelial cell	NDUFB1	0	0.407232492	0.86	0.434	0
Epithelial cell	PRELID2	0	0.407142478	0.373	0.006	0
Epithelial cell	DDOST	0	0.406879921	0.685	0.201	0
Epithelial cell	TMEM14C	0	0.406756687	0.746	0.243	0
Epithelial cell	PTP4A1	0	0.406575431	0.623	0.15	0
Epithelial cell	MRPS36	0	0.406352095	0.687	0.173	0
Epithelial cell	TMED9	0	0.406132468	0.766	0.298	0
Epithelial cell	HK2	0	0.405364095	0.31	0.015	0
Epithelial cell	GRN	0	0.404444224	0.793	0.208	0
Epithelial cell	TFG	0	0.404218637	0.583	0.107	0
Epithelial cell	AC008397.1	0	0.404033097	0.255	0.003	0
Epithelial cell	MEST	0	0.403874205	0.408	0.013	0
Epithelial cell	AAMDC	0	0.402841611	0.453	0.038	0
Epithelial cell	UQCR11	0	0.402697191	0.917	0.641	0
Epithelial cell	MRPL27	0	0.401881136	0.625	0.128	0
Epithelial cell	HOXB13	0	0.400859302	0.252	0.004	0
Epithelial cell	ATP2A2	0	0.400709802	0.515	0.066	0
Epithelial cell	TACC2	0	0.400652032	0.36	0.002	0
Epithelial cell	TWF1	0	0.400299057	0.517	0.061	0
Epithelial cell	DHCR24	0	0.399988032	0.338	0.01	0
Epithelial cell	CBR1	0	0.399321378	0.517	0.079	0
Epithelial cell	DYNLRB1	0	0.399256639	0.781	0.281	0
Epithelial cell	MORF4L2	0	0.399220923	0.677	0.193	0
Epithelial cell	POLR2H	0	0.398547244	0.584	0.101	0
Epithelial cell	PDIA6	0	0.397806147	0.797	0.376	0
Epithelial cell	SERF2	0	0.397801562	0.983	0.894	0
Epithelial cell	MPZL2	0	0.397482139	0.351	0.009	0
Epithelial cell	HDAC2	0	0.397209466	0.621	0.155	0
Epithelial cell	TOMM20	0	0.396884202	0.748	0.275	0
Epithelial cell	ETFA	0	0.39685412	0.681	0.189	0
Epithelial cell	STX3	0	0.396393213	0.399	0.028	0
Epithelial cell	MYO1C	0	0.396293138	0.446	0.04	0

Epithelial cell	CHDH	0	0.39617633	0.373	0.004	0
Epithelial cell	SLC1A5	0	0.395928159	0.443	0.074	0
Epithelial cell	EPB41L4B	0	0.395697888	0.348	0.003	0
Epithelial cell	FAM129B	0	0.395619669	0.376	0.012	0
Epithelial cell	SCCPDH	0	0.395390495	0.505	0.06	0
Epithelial cell	UACA	0	0.394436371	0.343	0.011	0
Epithelial cell	RPS2	0	0.393808806	0.961	0.974	0
Epithelial cell	AKR1A1	0	0.392489727	0.687	0.194	0
Epithelial cell	EBNA1BP2	0	0.392405926	0.528	0.089	0
Epithelial cell	ARHGEF5	0	0.392375251	0.329	0.008	0
Epithelial cell	MRPL51	0	0.391741988	0.742	0.276	0
Epithelial cell	PATJ	0	0.391498615	0.478	0.043	0
Epithelial cell	HACD3	0	0.391361454	0.472	0.07	0
Epithelial cell	TM9SF2	0	0.391267098	0.643	0.153	0
Epithelial cell	EIF4EBP1	0	0.390427252	0.513	0.095	0
Epithelial cell	WNK2	0	0.390151226	0.349	0.004	0
Epithelial cell	FKBP9	0	0.390137715	0.363	0.005	0
Epithelial cell	KIF9	0	0.389992709	0.383	0.011	0
Epithelial cell	DYNC1LI2	0	0.389579804	0.579	0.091	0
Epithelial cell	ARHGAP5	0	0.389467865	0.507	0.071	0
Epithelial cell	FDFT1	0	0.389392341	0.585	0.135	0
Epithelial cell	GOLGB1	0	0.389305183	0.663	0.166	0
Epithelial cell	YIPF3	0	0.389129251	0.58	0.118	0
Epithelial cell	ENAH	0	0.388932287	0.369	0.004	0
Epithelial cell	POLD2	0	0.385967998	0.508	0.083	0
Epithelial cell	TMEM63A	0	0.385277025	0.52	0.064	0
Epithelial cell	ERRFI1	0	0.385218239	0.262	0.01	0
Epithelial cell	TRAF4	0	0.384103511	0.461	0.042	0
Epithelial cell	KCNQ1	0	0.3834713	0.383	0.016	0
Epithelial cell	CDK4	0	0.383334532	0.513	0.098	0
Epithelial cell	ACP1	0	0.382979479	0.714	0.225	0
Epithelial cell	CLMN	0	0.382772813	0.372	0.022	0
Epithelial cell	MRPL37	0	0.382571553	0.497	0.062	0
Epithelial cell	PSMG1	0	0.382549804	0.465	0.06	0
Epithelial cell	TECR	0	0.382534934	0.685	0.211	0
Epithelial cell	RASSF7	0	0.382368091	0.574	0.095	0
Epithelial cell	FZD5	0	0.38207006	0.334	0.005	0
Epithelial cell	MRPS12	0	0.381981222	0.619	0.143	0
Epithelial cell	PAPSS2	0	0.381220316	0.344	0.016	0
Epithelial cell	AVPI1	0	0.380284666	0.373	0.025	0
Epithelial cell	NDUFA7	0	0.380044756	0.606	0.13	0
Epithelial cell	PNKD	0	0.380008816	0.651	0.157	0
Epithelial cell	PRUNE2	0	0.379834249	0.25	0.003	0

Epithelial cell	TNFRSF21	0	0.379822501	0.305	0.009	0
Epithelial cell	PTS	0	0.379655051	0.481	0.056	0
Epithelial cell	ATP1B3	0	0.379598009	0.708	0.224	0
Epithelial cell	RPS18	0	0.379491937	0.977	0.978	0
Epithelial cell	UBB	6.06E-264	0.379450952	0.952	0.752	1.41E-259
Epithelial cell	NORAD	0	0.379065324	0.637	0.145	0
Epithelial cell	GPRC5C	0	0.37854464	0.311	0.006	0
Epithelial cell	PSMB1	0	0.378338496	0.834	0.421	0
Epithelial cell	SH3PXD2A	0	0.377739875	0.371	0.017	0
Epithelial cell	SEMA3C	0	0.377470819	0.356	0.009	0
Epithelial cell	TDGF1	0	0.377066544	0.289	0.001	0
Epithelial cell	SCP2	0	0.377049681	0.818	0.351	0
Epithelial cell	TMEM219	0	0.37619394	0.756	0.251	0
Epithelial cell	GSKIP	0	0.375766018	0.509	0.082	0
Epithelial cell	INF2	0	0.375542713	0.367	0.022	0
Epithelial cell	PDIA3	0	0.375507468	0.823	0.459	0
Epithelial cell	ENY2	0	0.375447161	0.747	0.283	0
Epithelial cell	RALA	0	0.375372461	0.572	0.094	0
Epithelial cell	H2AFV	0	0.375302545	0.743	0.271	0
Epithelial cell	ACAT2	0	0.375266398	0.39	0.058	0
Epithelial cell	SF3B6	0	0.375131141	0.812	0.351	0
Epithelial cell	SIAE	0	0.37509121	0.35	0.008	0
Epithelial cell	CCL28	0	0.37493679	0.351	0.026	0
Epithelial cell	TMEM30B	0	0.374480324	0.361	0.008	0
Epithelial cell	HMGCS1	0	0.374414167	0.367	0.051	0
Epithelial cell	IRF6	0	0.374021677	0.312	0.003	0
Epithelial cell	BEX3	0	0.373831727	0.251	0.019	0
Epithelial cell	SEMA5A	0	0.373121419	0.311	0.002	0
Epithelial cell	TMEM205	0	0.372806163	0.567	0.118	0
Epithelial cell	GATA6	0	0.372538095	0.354	0.003	0
Epithelial cell	RTN3	0	0.372483455	0.627	0.146	0
Epithelial cell	CDCP1	0	0.37245741	0.336	0.007	0
Epithelial cell	ODC1	0	0.372085345	0.592	0.162	0
Epithelial cell	MTCH1	0	0.371732827	0.653	0.149	0
Epithelial cell	BAD	0	0.371420828	0.587	0.105	0
Epithelial cell	CARMIL1	0	0.371139325	0.38	0.026	0
Epithelial cell	SERBP1	0	0.370320285	0.832	0.478	0
Epithelial cell	FBXO32	0	0.370063102	0.278	0.028	0
Epithelial cell	CHPT1	0	0.369883518	0.608	0.129	0
Epithelial cell	IL18	0	0.369553859	0.346	0.025	0
Epithelial cell	ZNF593	0	0.369545763	0.536	0.09	0
Epithelial cell	DAP	0	0.369402473	0.489	0.073	0
Epithelial cell	CMTM4	0	0.369041056	0.36	0.005	0

Epithelial cell	ERO1A	0	0.368999776	0.447	0.103	0
Epithelial cell	MET	0	0.368896243	0.325	0.002	0
Epithelial cell	ARF5	0	0.368293385	0.723	0.22	0
Epithelial cell	PAK1	0	0.368111137	0.483	0.061	0
Epithelial cell	NR2F2	0	0.368053931	0.329	0.004	0
Epithelial cell	GIPC2	0	0.367958748	0.347	0.003	0
Epithelial cell	SEZ6L2	0	0.367919061	0.312	0.001	0
Epithelial cell	GNAS	0	0.366910934	0.846	0.415	0
Epithelial cell	MRPL11	0	0.366812632	0.614	0.142	0
Epithelial cell	CHCHD1	0	0.366582189	0.573	0.102	0
Epithelial cell	IP6K2	0	0.366219173	0.603	0.115	0
Epithelial cell	ANKRD22	0	0.365861195	0.329	0.006	0
Epithelial cell	RHBDL2	0	0.365690657	0.33	0.002	0
Epithelial cell	NHSL1	0	0.365677539	0.313	0.005	0
Epithelial cell	RAB40B	0	0.365268164	0.374	0.019	0
Epithelial cell	ARSE	0	0.364674737	0.336	0.002	0
Epithelial cell	BCAR1	0	0.364302118	0.334	0.004	0
Epithelial cell	MT-ND6	0	0.364190169	0.66	0.193	0
Epithelial cell	NDUFA9	0	0.363316344	0.595	0.142	0
Epithelial cell	EIF1AX	0	0.363256457	0.779	0.334	0
Epithelial cell	COMTD1	0	0.363140092	0.443	0.069	0
Epithelial cell	HMGB1	1.79E-119	0.361994402	0.927	0.812	4.15E-115
Epithelial cell	LAMTOR2	0	0.361763155	0.716	0.209	0
Epithelial cell	SH3RF1	0	0.361622564	0.343	0.008	0
Epithelial cell	TMEM123	0	0.361469893	0.786	0.367	0
Epithelial cell	IHH	0	0.361123277	0.311	0.002	0
Epithelial cell	PRELID1	0	0.361028687	0.824	0.418	0
Epithelial cell	BTF3	0	0.360579308	0.919	0.796	0
Epithelial cell	PTGES2	0	0.360302153	0.554	0.1	0
Epithelial cell	PSMB7	0	0.360231924	0.709	0.228	0
Epithelial cell	PDLIM5	0	0.35978699	0.476	0.06	0
Epithelial cell	NIPSNAP2	0	0.359242043	0.54	0.091	0
Epithelial cell	CARHSP1	0	0.358393993	0.639	0.167	0
Epithelial cell	ESD	0	0.358144297	0.694	0.232	0
Epithelial cell	DOK4	0	0.358014238	0.306	0.005	0
Epithelial cell	TRIOBP	0	0.357612506	0.453	0.043	0
Epithelial cell	RNPEP	0	0.357347179	0.49	0.068	0
Epithelial cell	MAP1LC3A	0	0.357075801	0.42	0.029	0
Epithelial cell	OAF	0	0.356449783	0.354	0.014	0
Epithelial cell	IL13RA1	0	0.356335856	0.417	0.047	0
Epithelial cell	EIF3I	0	0.356097361	0.73	0.289	0
Epithelial cell	CD320	0	0.355645037	0.54	0.101	0
Epithelial cell	SMS	0	0.355056085	0.643	0.164	0

Epithelial cell	GNB2	0	0.355019626	0.753	0.253	0
Epithelial cell	TMEM147	0	0.354600524	0.694	0.243	0
Epithelial cell	CCDC34	0	0.354376671	0.345	0.017	0
Epithelial cell	AK2	0	0.354235965	0.601	0.127	0
Epithelial cell	TSEN34	0	0.354072932	0.555	0.093	0
Epithelial cell	PIGZ	0	0.353979453	0.3	0.008	0
Epithelial cell	RNF128	0	0.353819133	0.327	0.001	0
Epithelial cell	ECH1	0	0.352205093	0.743	0.306	0
Epithelial cell	LGALS9	0	0.352010924	0.532	0.093	0
Epithelial cell	NDUFB11	0	0.351956505	0.836	0.431	0
Epithelial cell	REEP3	0	0.351661789	0.468	0.068	0
Epithelial cell	PPIB	0	0.351299468	0.838	0.5	0
Epithelial cell	TSPAN12	0	0.351297144	0.314	0.003	0
Epithelial cell	SVIL	0	0.351265982	0.366	0.013	0
Epithelial cell	ARL6IP1	0	0.351050637	0.768	0.328	0
Epithelial cell	DPM3	0	0.350984759	0.614	0.144	0
Epithelial cell	SLC25A4	0	0.350956447	0.442	0.046	0
Epithelial cell	SSBP1	0	0.350844858	0.762	0.311	0
Epithelial cell	DHRS3	0	0.350827122	0.403	0.039	0
Epithelial cell	ENTPD6	0	0.350493877	0.425	0.037	0
Epithelial cell	ARF1	0	0.350179205	0.835	0.377	0
Epithelial cell	CRNDE	0	0.35015608	0.256	0.004	0
Epithelial cell	ENTPD5	0	0.350008599	0.341	0.017	0
Epithelial cell	MMP15	0	0.349945682	0.322	0.005	0
Epithelial cell	CCDC47	0	0.349673418	0.552	0.112	0
Epithelial cell	RPS3	1.29E-298	0.34954414	0.973	0.96	3.01E-294
Epithelial cell	MRPL52	0	0.349401937	0.728	0.257	0
Epithelial cell	HOXB8	0	0.349336541	0.268	0.001	0
Epithelial cell	NOP16	0	0.349230368	0.409	0.04	0
Epithelial cell	FBP1	0	0.349126007	0.45	0.052	0
Epithelial cell	C1orf43	0	0.348844245	0.692	0.206	0
Epithelial cell	ARSD	0	0.348728687	0.372	0.017	0
Epithelial cell	NR4A1	0	0.348710139	0.559	0.147	0
Epithelial cell	CALR	0	0.348136484	0.861	0.458	0
Epithelial cell	MACC1	0	0.347852322	0.309	0.015	0
Epithelial cell	KIF1C	0	0.347340396	0.403	0.031	0
Epithelial cell	PBX1	0	0.347328952	0.327	0.007	0
Epithelial cell	EIF2S2	0	0.347119483	0.77	0.345	0
Epithelial cell	BRI3BP	0	0.347068192	0.445	0.054	0
Epithelial cell	RPL29	0	0.346805244	0.961	0.928	0
Epithelial cell	TFCP2L1	0	0.346459796	0.274	0.005	0
Epithelial cell	FAM114A1	0	0.345795366	0.305	0.015	0
Epithelial cell	DYNC1I2	0	0.345543847	0.695	0.193	0

Epithelial cell	MMAB	0	0.345175338	0.425	0.043	0
Epithelial cell	CNIH4	0	0.345088368	0.57	0.141	0
Epithelial cell	TOMM40	0	0.344926611	0.511	0.098	0
Epithelial cell	FKBP2	0	0.344832191	0.816	0.324	0
Epithelial cell	HOXA11-AS	0	0.344108817	0.305	0.003	0
Epithelial cell	PLCB3	0	0.343215539	0.347	0.016	0
Epithelial cell	PITX1	0	0.342386681	0.294	0.003	0
Epithelial cell	NIPAL1	0	0.34226297	0.317	0.002	0
Epithelial cell	SPIRE2	0	0.342161144	0.318	0.006	0
Epithelial cell	C19orf48	0	0.341749116	0.412	0.056	0
Epithelial cell	GSPT1	0	0.341620306	0.663	0.179	0
Epithelial cell	PSMB2	0	0.340811414	0.687	0.239	0
Epithelial cell	MZT2A	0	0.34061958	0.762	0.322	0
Epithelial cell	GADD45A	0	0.340577112	0.383	0.061	0
Epithelial cell	ARHGEF16	0	0.340324851	0.302	0.003	0
Epithelial cell	MRPL16	0	0.340117624	0.535	0.098	0
Epithelial cell	SERPINH1	0	0.339302326	0.369	0.022	0
Epithelial cell	AKR7A2	0	0.339122005	0.564	0.115	0
Epithelial cell	DPCD	0	0.338627011	0.341	0.014	0
Epithelial cell	PHB2	0	0.338319136	0.63	0.177	0
Epithelial cell	CRLS1	0	0.338306412	0.525	0.083	0
Epithelial cell	PLSCR1	0	0.338133872	0.584	0.111	0
Epithelial cell	ADAM9	0	0.337950632	0.367	0.026	0
Epithelial cell	SSBP3	0	0.337754597	0.419	0.034	0
Epithelial cell	FAM234A	0	0.337707019	0.397	0.027	0
Epithelial cell	YAP1	0	0.337614538	0.326	0.003	0
Epithelial cell	C1orf122	0	0.336817356	0.505	0.084	0
Epithelial cell	TOM1L1	0	0.336622842	0.335	0.003	0
Epithelial cell	NPTN	0	0.336428805	0.494	0.069	0
Epithelial cell	WEE1	0	0.33624875	0.335	0.036	0
Epithelial cell	C19orf70	0	0.335882532	0.73	0.275	0
Epithelial cell	RHEB	0	0.33534496	0.679	0.241	0
Epithelial cell	HYI	0	0.335167644	0.376	0.04	0
Epithelial cell	MRPL17	0	0.334850607	0.452	0.061	0
Epithelial cell	ADD3	0	0.334786306	0.64	0.153	0
Epithelial cell	MRPL18	0	0.333926755	0.616	0.144	0
Epithelial cell	GALNT12	0	0.333176641	0.297	0.011	0
Epithelial cell	ASAP2	0	0.332925302	0.297	0.005	0
Epithelial cell	NEO1	0	0.332793648	0.339	0.008	0
Epithelial cell	TMEM97	0	0.332359427	0.309	0.012	0
Epithelial cell	SBDS	0	0.332282667	0.695	0.205	0
Epithelial cell	THOC7	0	0.331485983	0.713	0.223	0
Epithelial cell	RAB32	0	0.331389871	0.407	0.049	0

Epithelial cell	METTL5	0	0.331234047	0.623	0.144	0
Epithelial cell	APEX1	0	0.331192934	0.617	0.183	0
Epithelial cell	RPN1	0	0.330494678	0.654	0.184	0
Epithelial cell	LSM5	0	0.330351178	0.742	0.314	0
Epithelial cell	RASSF6	0	0.329985724	0.351	0.035	0
Epithelial cell	SLC52A2	0	0.329897257	0.497	0.089	0
Epithelial cell	MYO1B	0	0.329594947	0.318	0.005	0
Epithelial cell	CCT7	0	0.329483608	0.644	0.194	0
Epithelial cell	MSMO1	0	0.329313196	0.354	0.053	0
Epithelial cell	CORO1B	0	0.329189686	0.69	0.174	0
Epithelial cell	UFC1	0	0.328961388	0.786	0.321	0
Epithelial cell	GSTO2	0	0.328743926	0.342	0.005	0
Epithelial cell	GRTP1	0	0.328431172	0.331	0.003	0
Epithelial cell	BDH1	0	0.327824075	0.364	0.014	0
Epithelial cell	SLC25A39	0	0.327751655	0.626	0.185	0
Epithelial cell	RPS7	7.25E-299	0.32691815	0.937	0.925	1.69E-294
Epithelial cell	CEBPA	0	0.32622643	0.339	0.02	0
Epithelial cell	BUD23	0	0.325968301	0.629	0.17	0
Epithelial cell	MRPS25	0	0.325741786	0.531	0.094	0
Epithelial cell	TP53I11	0	0.325610494	0.367	0.018	0
Epithelial cell	NAA20	0	0.325252055	0.559	0.147	0
Epithelial cell	DECR1	0	0.325209698	0.625	0.163	0
Epithelial cell	NOB1	0	0.324976983	0.482	0.077	0
Epithelial cell	DNPEP	0	0.324915662	0.567	0.114	0
Epithelial cell	MRPS15	0	0.324213613	0.61	0.155	0
Epithelial cell	ABHD2	0	0.323893387	0.425	0.053	0
Epithelial cell	GSS	0	0.323852073	0.428	0.051	0
Epithelial cell	PTK6	0	0.323786939	0.281	0.005	0
Epithelial cell	CGN	0	0.323786598	0.259	0.003	0
Epithelial cell	NDUFB5	0	0.323676959	0.745	0.292	0
Epithelial cell	ACVRL1	0	0.323259409	0.273	0.01	0
Epithelial cell	RBFOX2	0	0.323234862	0.331	0.005	0
Epithelial cell	RTN4	0	0.323026833	0.794	0.323	0
Epithelial cell	SORBS2	0	0.322854441	0.26	0.006	0
Epithelial cell	PAQR8	0	0.321943606	0.358	0.034	0
Epithelial cell	LSM3	0	0.321768584	0.75	0.309	0
Epithelial cell	B3GNT3	0	0.321698932	0.294	0.002	0
Epithelial cell	NDUFAF2	0	0.321661289	0.497	0.083	0
Epithelial cell	MAP3K20	0	0.321448436	0.328	0.029	0
Epithelial cell	CASP7	0	0.321395731	0.441	0.063	0
Epithelial cell	MTUS1	0	0.321232027	0.313	0.009	0
Epithelial cell	RPL26L1	0	0.320906913	0.495	0.082	0
Epithelial cell	NEK3	0	0.320717351	0.322	0.012	0

Epithelial cell	NSA2	0	0.32040756	0.731	0.259	0
Epithelial cell	MRPS18A	0	0.320334594	0.525	0.086	0
Epithelial cell	SRP9	0	0.320222714	0.815	0.411	0
Epithelial cell	GUCY2C	0	0.320109467	0.318	0.013	0
Epithelial cell	DACH1	0	0.319942976	0.268	0.001	0
Epithelial cell	ELOC	0	0.319885828	0.722	0.258	0
Epithelial cell	TNFRSF11A	0	0.319671572	0.309	0.006	0
Epithelial cell	VPS51	0	0.319360885	0.588	0.133	0
Epithelial cell	LXN	0	0.319312468	0.313	0.015	0
Epithelial cell	MCM7	0	0.319249086	0.339	0.043	0
Epithelial cell	SLC11A2	0	0.319143176	0.312	0.036	0
Epithelial cell	PIP5K1B	0	0.319052221	0.346	0.027	0
Epithelial cell	SORL1	0	0.318836203	0.501	0.083	0
Epithelial cell	C8orf82	0	0.318327611	0.441	0.049	0
Epithelial cell	PCDH1	0	0.318218947	0.267	0.002	0
Epithelial cell	RACK1	0	0.317536174	0.936	0.899	0
Epithelial cell	TLE1	0	0.316905661	0.389	0.026	0
Epithelial cell	EPHX2	0	0.316710562	0.349	0.022	0
Epithelial cell	ACOX1	0	0.316452333	0.373	0.039	0
Epithelial cell	HDAC1	0	0.316180681	0.679	0.189	0
Epithelial cell	MANBAL	0	0.315935239	0.416	0.039	0
Epithelial cell	ATOX1	0	0.315916794	0.636	0.163	0
Epithelial cell	NTPCR	0	0.315235323	0.432	0.038	0
Epithelial cell	F12	0	0.315197879	0.31	0.008	0
Epithelial cell	ADH5	0	0.31484721	0.593	0.137	0
Epithelial cell	RUVBL2	0	0.314646884	0.458	0.068	0
Epithelial cell	COQ9	0	0.314338492	0.441	0.048	0
Epithelial cell	ILVBL	0	0.314333529	0.419	0.044	0
Epithelial cell	COA6	0	0.3142332	0.487	0.069	0
Epithelial cell	RNF5	0	0.314145371	0.592	0.131	0
Epithelial cell	MORN2	0	0.313923293	0.344	0.014	0
Epithelial cell	EXOSC4	0	0.313374879	0.43	0.06	0
Epithelial cell	NFE2L3	0	0.313311136	0.345	0.028	0
Epithelial cell	B3GNT5	0	0.313060128	0.313	0.015	0
Epithelial cell	MXD1	0	0.312610199	0.309	0.062	0
Epithelial cell	HEBP1	0	0.312481975	0.421	0.047	0
Epithelial cell	NDUFS2	0	0.312215285	0.631	0.161	0
Epithelial cell	HOXA7	0	0.312068689	0.293	0.003	0
Epithelial cell	RETREG1	0	0.312019539	0.435	0.06	0
Epithelial cell	FAAH	0	0.311296605	0.341	0.009	0
Epithelial cell	EPPK1	0	0.31101111	0.264	0.002	0
Epithelial cell	HSD17B4	0	0.310861429	0.453	0.066	0
Epithelial cell	MLF2	0	0.310716109	0.601	0.137	0

Epithelial cell	EIF4G1	0	0.310596909	0.552	0.112	0
Epithelial cell	NDUFB8	0	0.31055836	0.778	0.37	0
Epithelial cell	MMP24OS	0	0.310223176	0.586	0.137	0
Epithelial cell	STAU1	0	0.309644877	0.625	0.157	0
Epithelial cell	RETSAT	0	0.309567871	0.316	0.019	0
Epithelial cell	GLCE	0	0.309394868	0.34	0.015	0
Epithelial cell	ZNF664	0	0.308983586	0.404	0.033	0
Epithelial cell	SLC25A1	0	0.308784189	0.426	0.055	0
Epithelial cell	CMTM8	0	0.308478636	0.399	0.027	0
Epithelial cell	NUTF2	0	0.307606553	0.597	0.149	0
Epithelial cell	EXPH5	0	0.307570405	0.286	0.003	0
Epithelial cell	SMIM26	0	0.307434874	0.741	0.247	0
Epithelial cell	EFNB1	0	0.307257103	0.304	0.009	0
Epithelial cell	CHMP5	0	0.307131631	0.665	0.195	0
Epithelial cell	NUDT8	0	0.307069258	0.338	0.026	0
Epithelial cell	SHTN1	0	0.306848672	0.358	0.029	0
Epithelial cell	MT-ND5	7.02E-223	0.306578136	0.965	0.863	1.63E-218
Epithelial cell	CLSTN1	0	0.306403963	0.486	0.08	0
Epithelial cell	ACOT11	0	0.306353219	0.292	0.006	0
Epithelial cell	ACOT7	0	0.306144063	0.355	0.036	0
Epithelial cell	MYL12B	7.13E-234	0.305871243	0.928	0.666	1.66E-229
Epithelial cell	PARVA	0	0.305577321	0.31	0.002	0
Epithelial cell	ANKS4B	0	0.30533324	0.284	0.002	0
Epithelial cell	ANKH	0	0.304822384	0.497	0.075	0
Epithelial cell	SLC39A7	0	0.304252921	0.453	0.07	0
Epithelial cell	GRHPR	0	0.30415033	0.553	0.104	0
Epithelial cell	HEXB	0	0.303964975	0.602	0.159	0
Epithelial cell	SLC5A1	0	0.303658629	0.291	0.002	0
Epithelial cell	ABHD12	0	0.303279692	0.409	0.056	0
Epithelial cell	HSD17B11	0	0.303269983	0.721	0.265	0
Epithelial cell	EIF4E2	0	0.302989485	0.592	0.127	0
Epithelial cell	BANF1	0	0.302980523	0.735	0.265	0
Epithelial cell	SELENOS	0	0.302935153	0.733	0.263	0
Epithelial cell	NDUFS8	0	0.30289676	0.74	0.301	0
Epithelial cell	LRP5	0	0.302392097	0.297	0.005	0
Epithelial cell	HMGN2	1.47E-101	0.302382575	0.842	0.592	3.42E-97
Epithelial cell	TOP1	0	0.302318249	0.622	0.179	0
Epithelial cell	SOWAHC	0	0.302210043	0.269	0.004	0
Epithelial cell	NPNT	0	0.301145209	0.266	0.001	0
Epithelial cell	AIMP2	0	0.301080092	0.395	0.036	0
Epithelial cell	YES1	0	0.300969387	0.391	0.027	0
Epithelial cell	LGMN	0	0.300952141	0.444	0.046	0
Epithelial cell	SDF4	0	0.300850361	0.661	0.188	0

Epithelial cell	KANK1	0	0.300756624	0.307	0.013	0
Epithelial cell	GHITM	0	0.300756023	0.778	0.346	0
Epithelial cell	MANSC1	0	0.300590315	0.315	0.004	0
Epithelial cell	PRKCSH	0	0.300383615	0.629	0.154	0
Epithelial cell	SDHC	0	0.30030604	0.698	0.222	0
Epithelial cell	CHKA	0	0.299625975	0.326	0.019	0
Epithelial cell	KCNE3	0	0.299532071	0.328	0.018	0
Epithelial cell	LACTB2	0	0.29941098	0.392	0.051	0
Epithelial cell	MKRN1	0	0.298693505	0.663	0.169	0
Epithelial cell	LURAP1L	0	0.2981413	0.269	0.003	0
Epithelial cell	ACO2	0	0.298130898	0.538	0.107	0
Epithelial cell	HNRNPA1	2.15E-186	0.29786055	0.908	0.843	5.00E-182
Epithelial cell	PTK2	0	0.296958373	0.359	0.021	0
Epithelial cell	GTF2I	0	0.296777172	0.636	0.2	0
Epithelial cell	FAM83H	0	0.296491771	0.31	0.007	0
Epithelial cell	HIBADH	0	0.296304774	0.409	0.045	0
Epithelial cell	TMEM183A	0	0.296204772	0.573	0.113	0
Epithelial cell	PRDX3	0	0.296177823	0.652	0.21	0
Epithelial cell	BCL10	0	0.295936213	0.441	0.074	0
Epithelial cell	MBOAT2	0	0.29555119	0.27	0.006	0
Epithelial cell	TMEM9	0	0.295475692	0.358	0.034	0
Epithelial cell	FH	0	0.295353056	0.413	0.055	0
Epithelial cell	MRPL4	0	0.295215078	0.528	0.102	0
Epithelial cell	NFIX	0	0.294533711	0.317	0.007	0
Epithelial cell	PRRG4	0	0.29373494	0.308	0.011	0
Epithelial cell	A1CF	0	0.29347691	0.254	0.001	0
Epithelial cell	FAM135A	0	0.293422195	0.308	0.014	0
Epithelial cell	MARVELD2	0	0.29327661	0.294	0.002	0
Epithelial cell	PINK1	0	0.29263427	0.306	0.028	0
Epithelial cell	RTRAF	0	0.292341136	0.782	0.365	0
Epithelial cell	MRPL36	0	0.292223854	0.562	0.136	0
Epithelial cell	RNF114	0	0.292153672	0.586	0.135	0
Epithelial cell	PGM1	0	0.292129755	0.406	0.052	0
Epithelial cell	KIAA1217	0	0.291828616	0.26	0.003	0
Epithelial cell	ZNF704	0	0.291799233	0.27	0.002	0
Epithelial cell	CUX1	0	0.291730097	0.451	0.063	0
Epithelial cell	LINC00668	0	0.29171442	0.252	0.001	0
Epithelial cell	TRAF7	0	0.291063778	0.406	0.046	0
Epithelial cell	SUGT1	0	0.290987859	0.574	0.134	0
Epithelial cell	IL17RE	0	0.29028777	0.297	0.008	0
Epithelial cell	RPL36	3.14E-262	0.289853305	0.977	0.952	7.30E-258
Epithelial cell	DAG1	0	0.289355871	0.334	0.014	0
Epithelial cell	GOLGA4	0	0.288902867	0.715	0.232	0

Epithelial cell	PLEKHA6	0	0.288832409	0.269	0.003	0
Epithelial cell	KIF13A	0	0.288581279	0.317	0.011	0
Epithelial cell	EIF3K	2.07E-306	0.288510946	0.849	0.573	4.81E-302
Epithelial cell	AFG3L2	0	0.288423073	0.477	0.074	0
Epithelial cell	BCL2L14	0	0.288049132	0.28	0.002	0
Epithelial cell	HOXA13	0	0.288018754	0.276	0.001	0
Epithelial cell	FGFR4	0	0.287845927	0.284	0.002	0
Epithelial cell	GNPNAT1	0	0.28777025	0.36	0.027	0
Epithelial cell	CYB561	0	0.287503349	0.337	0.018	0
Epithelial cell	DHCR7	0	0.287438932	0.283	0.017	0
Epithelial cell	TMEM238L	0	0.28737019	0.282	0.002	0
Epithelial cell	LAMTOR4	0	0.287270098	0.832	0.428	0
Epithelial cell	CLTC	0	0.287087484	0.592	0.128	0
Epithelial cell	ARL1	0	0.286593882	0.563	0.126	0
Epithelial cell	SMPDL3B	0	0.286219047	0.284	0.007	0
Epithelial cell	MLLT3	0	0.286159459	0.421	0.072	0
Epithelial cell	SRSF9	0	0.286048102	0.783	0.349	0
Epithelial cell	EPS8L2	0	0.28593019	0.455	0.07	0
Epithelial cell	CSNK2B	0	0.285604812	0.738	0.264	0
Epithelial cell	RNASE4	0	0.285002659	0.302	0.013	0
Epithelial cell	ALDOA	0	0.284961564	0.502	0.114	0
Epithelial cell	EIF3B	0	0.284930676	0.504	0.081	0
Epithelial cell	NOSTRIN	0	0.284523708	0.258	0.002	0
Epithelial cell	FOXA1	0	0.284374127	0.279	0.002	0
Epithelial cell	SEPHS2	0	0.284305455	0.619	0.17	0
Epithelial cell	LRRC1	0	0.284252706	0.304	0.018	0
Epithelial cell	C9orf3	0	0.28333048	0.276	0.009	0
Epithelial cell	TESC	0	0.283248133	0.268	0.065	0
Epithelial cell	SAMD5	0	0.283170822	0.266	0.001	0
Epithelial cell	CCT3	0	0.283001499	0.672	0.232	0
Epithelial cell	PLOD3	0	0.282974474	0.378	0.039	0
Epithelial cell	SLC39A8	0	0.282708807	0.387	0.059	0
Epithelial cell	CXCL16	0	0.282436451	0.366	0.045	0
Epithelial cell	RPL12	3.61E-280	0.282159977	0.973	0.954	8.40E-276
Epithelial cell	PSMA3	0	0.28197996	0.656	0.226	0
Epithelial cell	ARHGEF10L	0	0.281862065	0.307	0.013	0
Epithelial cell	ZDHHC9	0	0.281494689	0.307	0.007	0
Epithelial cell	POLR2J	0	0.281371771	0.721	0.239	0
Epithelial cell	SNRPC	0	0.280700517	0.65	0.189	0
Epithelial cell	C8orf59	0	0.280661725	0.728	0.277	0
Epithelial cell	ATF4	0	0.280654746	0.695	0.253	0
Epithelial cell	FARP1	0	0.279967166	0.255	0.005	0
Epithelial cell	FASN	0	0.279320571	0.299	0.016	0

Epithelial cell	EFNA4	0	0.278951802	0.346	0.022	0
Epithelial cell	TPT1	3.02E-148	0.278817633	0.988	0.958	7.02E-144
Epithelial cell	GOT1	0	0.278806676	0.36	0.032	0
Epithelial cell	SHROOM1	0	0.278690959	0.267	0.01	0
Epithelial cell	COX14	0	0.278383031	0.728	0.254	0
Epithelial cell	UBL5	2.74E-304	0.278281915	0.921	0.615	6.36E-300
Epithelial cell	TRIM28	0	0.278011998	0.48	0.081	0
Epithelial cell	NPM3	0	0.277982011	0.425	0.068	0
Epithelial cell	CASK	0	0.277880667	0.384	0.029	0
Epithelial cell	CDK2AP1	0	0.277764865	0.324	0.019	0
Epithelial cell	TTC39A	0	0.277696875	0.258	0.002	0
Epithelial cell	SORT1	0	0.276998328	0.304	0.013	0
Epithelial cell	AGRN	0	0.276828562	0.257	0.007	0
Epithelial cell	KLHL23	0	0.276823602	0.325	0.017	0
Epithelial cell	MRPL3	0	0.276509145	0.583	0.148	0
Epithelial cell	PCK2	0	0.276324761	0.349	0.033	0
Epithelial cell	PSMB4	0	0.276136914	0.585	0.142	0
Epithelial cell	GLO1	0	0.276087647	0.583	0.185	0
Epithelial cell	PHLDA1	0	0.27599211	0.352	0.07	0
Epithelial cell	PSME2	0	0.27557529	0.804	0.435	0
Epithelial cell	SH2D4A	0	0.275540087	0.277	0.002	0
Epithelial cell	IFNGR2	0	0.275493129	0.513	0.113	0
Epithelial cell	DNAJC22	0	0.275331457	0.274	0.001	0
Epithelial cell	PTPN3	0	0.275262985	0.258	0.002	0
Epithelial cell	POP7	0	0.274739194	0.482	0.078	0
Epithelial cell	DEK	0	0.274306502	0.778	0.359	0
Epithelial cell	WLS	0	0.273671033	0.298	0.009	0
Epithelial cell	PIGP	0	0.273565189	0.528	0.108	0
Epithelial cell	SRGAP1	0	0.272971145	0.267	0.013	0
Epithelial cell	DUS1L	0	0.272627226	0.426	0.059	0
Epithelial cell	DHX32	0	0.272430499	0.325	0.022	0
Epithelial cell	MCUR1	0	0.272247517	0.4	0.061	0
Epithelial cell	DNM2	0	0.27209059	0.532	0.12	0
Epithelial cell	PFDN4	0	0.272051364	0.604	0.156	0
Epithelial cell	SNHG19	0	0.271923348	0.347	0.039	0
Epithelial cell	RALY	0	0.27182958	0.728	0.262	0
Epithelial cell	MRPS26	0	0.271117279	0.559	0.125	0
Epithelial cell	LRP10	0	0.270949156	0.623	0.191	0
Epithelial cell	RHPN2	0	0.270906599	0.251	0.002	0
Epithelial cell	PODXL2	0	0.270629413	0.258	0.009	0
Epithelial cell	GPSM2	0	0.27054588	0.329	0.032	0
Epithelial cell	HMGCR	0	0.270444186	0.318	0.031	0
Epithelial cell	CDHR1	0	0.270194448	0.252	0.006	0

Epithelial cell	PPP2CB	0	0.269767856	0.362	0.04	0
Epithelial cell	NDUFS1	0	0.269760607	0.469	0.072	0
Epithelial cell	FBL	0	0.26972435	0.691	0.267	0
Epithelial cell	GSTM3	0	0.269557109	0.256	0.022	0
Epithelial cell	AP2S1	0	0.269389287	0.758	0.277	0
Epithelial cell	FNIP2	0	0.26923889	0.35	0.034	0
Epithelial cell	VILL	0	0.268888081	0.282	0.023	0
Epithelial cell	LRRC75A	0	0.268817336	0.257	0.02	0
Epithelial cell	FAM213B	0	0.268734272	0.308	0.019	0
Epithelial cell	SEC61B	0	0.268620676	0.848	0.447	0
Epithelial cell	EGFR	0	0.26837013	0.268	0.003	0
Epithelial cell	PTOV1	0	0.26827323	0.479	0.085	0
Epithelial cell	NRIP1	0	0.267415818	0.393	0.041	0
Epithelial cell	COX17	0	0.267169852	0.744	0.306	0
Epithelial cell	CTDSPL	0	0.266368029	0.278	0.005	0
Epithelial cell	ZNF326	0	0.266270475	0.452	0.069	0
Epithelial cell	MYO7B	0	0.265545141	0.257	0.009	0
Epithelial cell	ST5	0	0.265543675	0.258	0.003	0
Epithelial cell	FUCA2	0	0.265002955	0.453	0.077	0
Epithelial cell	RTCB	0	0.264879893	0.51	0.104	0
Epithelial cell	MRPL22	0	0.264769091	0.556	0.12	0
Epithelial cell	SMTN	0	0.264700426	0.276	0.01	0
Epithelial cell	FAM136A	0	0.264575046	0.521	0.106	0
Epithelial cell	CDC42BPB	0	0.264486361	0.303	0.012	0
Epithelial cell	ILF2	0	0.264339974	0.62	0.2	0
Epithelial cell	LINC00511	0	0.263805843	0.251	0.002	0
Epithelial cell	CCDC112	0	0.263631629	0.338	0.035	0
Epithelial cell	KIAA2013	0	0.26347462	0.4	0.054	0
Epithelial cell	LRRFIP2	0	0.26346482	0.436	0.063	0
Epithelial cell	CLDN15	0	0.263325302	0.286	0.021	0
Epithelial cell	SCML1	0	0.263032256	0.311	0.016	0
Epithelial cell	SAMD13	0	0.262580684	0.263	0.002	0
Epithelial cell	TPMT	0	0.262433793	0.477	0.092	0
Epithelial cell	THAP4	0	0.262417068	0.376	0.038	0
Epithelial cell	ZFYVE21	0	0.261896872	0.421	0.051	0
Epithelial cell	ATP5MC2	1.15E-252	0.261549486	0.886	0.694	2.68E-248
Epithelial cell	DLD	0	0.261489834	0.525	0.107	0
Epithelial cell	VDR	0	0.261441585	0.295	0.016	0
Epithelial cell	DAB2	0	0.260550231	0.327	0.032	0
Epithelial cell	ELMO3	0	0.260386461	0.284	0.007	0
Epithelial cell	PCNA	0	0.260242917	0.391	0.106	0
Epithelial cell	RIOK3	0	0.259597808	0.468	0.111	0
Epithelial cell	GALNT1	0	0.259417753	0.526	0.116	0

Epithelial cell	ARHGAP8	0	0.258960088	0.266	0.002	0
Epithelial cell	PANK3	0	0.258857498	0.379	0.041	0
Epithelial cell	BUD31	0	0.258760793	0.684	0.209	0
Epithelial cell	ARMC10	0	0.258637153	0.529	0.106	0
Epithelial cell	UGT8	0	0.258633548	0.295	0.012	0
Epithelial cell	ANXA11	0	0.25832658	0.765	0.307	0
Epithelial cell	MYO5C	0	0.25798199	0.268	0.005	0
Epithelial cell	MALSU1	0	0.257862092	0.455	0.064	0
Epithelial cell	RARS	0	0.257495296	0.485	0.085	0
Epithelial cell	GCAT	0	0.257472572	0.273	0.004	0
Epithelial cell	SDHA	0	0.257383015	0.58	0.148	0
Epithelial cell	GAR1	0	0.257325227	0.43	0.061	0
Epithelial cell	MIR4458HG	0	0.257141356	0.317	0.024	0
Epithelial cell	RRAS	0	0.256967667	0.293	0.022	0
Epithelial cell	MRPL24	0	0.25687794	0.518	0.104	0
Epithelial cell	DTYMK	0	0.256718292	0.34	0.055	0
Epithelial cell	PDAP1	0	0.25656283	0.647	0.186	0
Epithelial cell	RPP25	0	0.256304347	0.294	0.017	0
Epithelial cell	TM7SF2	0	0.256029053	0.293	0.028	0
Epithelial cell	ADGRA3	0	0.256025293	0.256	0.002	0
Epithelial cell	MTX2	0	0.256014933	0.468	0.074	0
Epithelial cell	EFHD2	0	0.255915796	0.623	0.15	0
Epithelial cell	DNTTIP1	0	0.255792728	0.397	0.059	0
Epithelial cell	COPRS	0	0.255596633	0.403	0.06	0
Epithelial cell	SLC39A14	0	0.25552934	0.287	0.014	0
Epithelial cell	JPT2	0	0.255298593	0.366	0.034	0
Epithelial cell	UBAC1	0	0.255164897	0.376	0.044	0
Epithelial cell	FAHD1	0	0.255041707	0.338	0.027	0
Epithelial cell	P4HA2	0	0.254815487	0.251	0.012	0
Epithelial cell	FAM84B	0	0.254482818	0.267	0.007	0
Epithelial cell	SYNJ2BP	0	0.254216644	0.503	0.085	0
Epithelial cell	NAXE	0	0.253907851	0.599	0.157	0
Epithelial cell	RTKN	0	0.253859159	0.275	0.004	0
Epithelial cell	STK24	0	0.25383455	0.512	0.102	0
Epithelial cell	NDUFA5	0	0.253723558	0.708	0.25	0
Epithelial cell	LRP11	0	0.253693187	0.273	0.007	0
Epithelial cell	POLR2F	0	0.253663022	0.661	0.21	0
Epithelial cell	SLC25A10	0	0.253617869	0.284	0.006	0
Epithelial cell	MAGI3	0	0.253187938	0.258	0.007	0
Epithelial cell	NEU1	0	0.252732871	0.414	0.094	0
Epithelial cell	KIF16B	0	0.252665223	0.29	0.018	0
Epithelial cell	ERGIC1	0	0.252566018	0.513	0.1	0
Epithelial cell	ALDH3A2	0	0.252419943	0.382	0.048	0

Epithelial cell	LIMK2	0	0.252268248	0.334	0.022	0
Epithelial cell	SYTL2	0	0.252090664	0.43	0.088	0
Epithelial cell	FUT4	0	0.251556362	0.275	0.01	0
Epithelial cell	YIPF2	0	0.251201142	0.426	0.067	0
Epithelial cell	HADHB	0	0.251119227	0.591	0.148	0
Epithelial cell	HINT2	0	0.251110326	0.596	0.16	0
Epithelial cell	PSMD7	0	0.251028342	0.654	0.209	0
Epithelial cell	CLINT1	0	0.250710419	0.529	0.125	0
Epithelial cell	NEK6	0	0.250663498	0.331	0.03	0
Epithelial cell	NUDT15	0	0.250469212	0.333	0.025	0
Epithelial cell	ARHGEF12	0	0.250252846	0.42	0.052	0
Epithelial cell	SLC35D2	0	0.250016599	0.345	0.027	0
Fibroblast	CFD	2.50E-151	6.342726594	0.835	0.183	5.82E-147
Fibroblast	DCN	0	6.279915035	0.89	0.017	0
Fibroblast	MGP	0	6.146506294	0.848	0.013	0
Fibroblast	IGFBP6	0	5.487930103	0.86	0.019	0
Fibroblast	COL1A2	0	5.417553859	0.909	0.006	0
Fibroblast	FBLN1	0	4.98148779	0.866	0.009	0
Fibroblast	COL3A1	0	4.883996854	0.835	0.005	0
Fibroblast	COL1A1	0	4.783373726	0.811	0.012	0
Fibroblast	CCDC80	0	4.63711454	0.841	0.007	0
Fibroblast	MFAP5	0	4.628772367	0.756	0.005	0
Fibroblast	TNXB	0	4.624397621	0.823	0.004	0
Fibroblast	GSN	6.49E-126	4.621392125	0.909	0.287	1.51E-121
Fibroblast	COL6A2	0	4.58223785	0.884	0.007	0
Fibroblast	PLAC9	0	4.353443273	0.823	0.003	0
Fibroblast	SFRP2	0	4.321148517	0.591	0.004	0
Fibroblast	TIMP3	0	4.161662153	0.817	0.015	0
Fibroblast	LUM	0	4.111301693	0.811	0.005	0
Fibroblast	C1R	0	4.092478854	0.829	0.009	0
Fibroblast	FSTL1	0	3.942938163	0.738	0.012	0
Fibroblast	FN1	0	3.942056958	0.799	0.026	0
Fibroblast	FBLN2	0	3.92919195	0.756	0.004	0
Fibroblast	MEG3	0	3.924265489	0.78	0.001	0
Fibroblast	C1S	0	3.907251246	0.872	0.004	0
Fibroblast	FBN1	0	3.808545798	0.701	0.002	0
Fibroblast	CLU	1.08E-281	3.725835761	0.695	0.059	2.51E-277
Fibroblast	IGFBP7	0	3.661635608	0.726	0.034	0
Fibroblast	LTBP4	2.33E-201	3.635996263	0.848	0.136	5.42E-197
Fibroblast	IGFBP5	0	3.623994231	0.652	0.001	0
Fibroblast	SPARC	0	3.558963506	0.774	0.004	0
Fibroblast	ADH1B	0	3.545948682	0.665	0.002	0
Fibroblast	COL6A1	0	3.506021848	0.793	0.008	0

Fibroblast	MFAP4	0	3.440112678	0.713	0.004	0
Fibroblast	MMP2	0	3.402239878	0.78	0.003	0
Fibroblast	C3	0	3.387408443	0.616	0.006	0
Fibroblast	SPARCL1	0	3.384825045	0.628	0.001	0
Fibroblast	CALD1	0	3.281268125	0.835	0.006	0
Fibroblast	SERPING1	0	3.271995928	0.75	0.008	0
Fibroblast	COL6A3	0	3.267423047	0.707	0.002	0
Fibroblast	EFEMP1	0	3.177469579	0.695	0.003	0
Fibroblast	PCOLCE	0	3.07673163	0.75	0.014	0
Fibroblast	DPT	0	3.051114303	0.573	0.004	0
Fibroblast	SFRP1	0	3.001316799	0.573	0.001	0
Fibroblast	TIMP2	0	2.962109874	0.744	0.05	0
Fibroblast	COL14A1	0	2.948283254	0.665	0.001	0
Fibroblast	PI16	0	2.943116044	0.579	0.001	0
Fibroblast	CD248	0	2.929617573	0.646	0.006	0
Fibroblast	RARRES2	3.83E-168	2.887085866	0.64	0.082	8.91E-164
Fibroblast	SERPINF1	0	2.869965794	0.646	0.009	0
Fibroblast	PLPP3	2.55E-232	2.803850787	0.591	0.05	5.94E-228
Fibroblast	PTN	0	2.761912959	0.402	0.001	0
Fibroblast	LGALS1	1.68E-96	2.698426062	0.902	0.324	3.91E-92
Fibroblast	ABCA8	0	2.661652207	0.567	0.001	0
Fibroblast	OGN	0	2.658651041	0.555	0.001	0
Fibroblast	GPX3	0	2.627723382	0.573	0.011	0
Fibroblast	PCOLCE2	0	2.587075969	0.561	0.002	0
Fibroblast	SLPI	7.57E-33	2.57996326	0.537	0.234	1.76E-28
Fibroblast	SCARA5	0	2.575840731	0.61	0.001	0
Fibroblast	GPNMB	0	2.566921131	0.659	0.016	0
Fibroblast	AEBP1	0	2.544390881	0.616	0.003	0
Fibroblast	ADIRF	3.07E-72	2.507756049	0.622	0.17	7.14E-68
Fibroblast	MFGE8	0	2.483256905	0.634	0.016	0
Fibroblast	SOD3	1.03E-80	2.477805053	0.634	0.166	2.38E-76
Fibroblast	CD55	1.97E-52	2.473107771	0.689	0.303	4.59E-48
Fibroblast	AHNAK	7.53E-77	2.454976693	0.774	0.288	1.75E-72
Fibroblast	IGFBP4	2.50E-58	2.449274815	0.61	0.21	5.81E-54
Fibroblast	THY1	0	2.438705107	0.598	0.001	0
Fibroblast	PMP22	0	2.389130998	0.634	0.038	0
Fibroblast	S100A4	9.38E-64	2.382129564	0.89	0.483	2.18E-59
Fibroblast	CFH	0	2.36792125	0.543	0.004	0
Fibroblast	CYBRD1	3.73E-263	2.338684396	0.573	0.041	8.67E-259
Fibroblast	SLIT3	0	2.327349324	0.524	0.001	0
Fibroblast	LRP1	7.55E-113	2.322228281	0.634	0.121	1.76E-108
Fibroblast	CLEC3B	0	2.314359145	0.494	0.002	0
Fibroblast	ISLR	0	2.31417334	0.537	0.001	0

Fibroblast	SERPINE2	9.22E-123	2.303401821	0.47	0.057	2.14E-118
Fibroblast	APOE	3.85E-131	2.296217369	0.329	0.025	8.94E-127
Fibroblast	CYR61	8.61E-115	2.289650949	0.372	0.038	2.00E-110
Fibroblast	MRC2	0	2.279439808	0.567	0.004	0
Fibroblast	CAVIN3	0	2.277661518	0.579	0.02	0
Fibroblast	CD81	9.82E-64	2.262855	0.707	0.277	2.28E-59
Fibroblast	VIM	3.96E-90	2.259731893	0.939	0.396	9.20E-86
Fibroblast	CEBPD	1.57E-50	2.256589079	0.689	0.303	3.66E-46
Fibroblast	CXCL12	0	2.24892922	0.573	0.004	0
Fibroblast	TIMP1	5.20E-79	2.248331549	0.848	0.37	1.21E-74
Fibroblast	TFPI	0	2.244722027	0.555	0.012	0
Fibroblast	APOD	3.75E-92	2.232065115	0.25	0.021	8.71E-88
Fibroblast	CILP	0	2.224093916	0.537	0.001	0
Fibroblast	IFITM3	3.09E-80	2.186798898	0.878	0.358	7.18E-76
Fibroblast	EMP3	2.23E-75	2.183179824	0.811	0.281	5.19E-71
Fibroblast	PTGIS	0	2.178350619	0.451	0.001	0
Fibroblast	CTSK	0	2.173257425	0.549	0.008	0
Fibroblast	CES1	0	2.159012274	0.439	0.011	0
Fibroblast	HTRA1	0	2.124798512	0.53	0.024	0
Fibroblast	VCAN	0	2.120817762	0.683	0.044	0
Fibroblast	CST3	3.57E-64	2.070741992	0.909	0.509	8.29E-60
Fibroblast	COX7A1	0	2.057134404	0.524	0.001	0
Fibroblast	KRT24	0	2.045083372	0.396	0.001	0
Fibroblast	ACKR3	0	2.039098655	0.47	0.002	0
Fibroblast	A2M	3.96E-206	2.038652628	0.287	0.012	9.20E-202
Fibroblast	PBX1	8.36E-78	2.024118975	0.567	0.134	1.94E-73
Fibroblast	PLTP	1.03E-173	2.023382195	0.47	0.041	2.40E-169
Fibroblast	S100A13	9.11E-46	2.018487365	0.604	0.252	2.12E-41
Fibroblast	FGFR1	0	2.017892607	0.463	0.005	0
Fibroblast	CAVIN1	0	1.995959644	0.524	0.019	0
Fibroblast	IL6ST	1.01E-87	1.993889965	0.579	0.122	2.35E-83
Fibroblast	PLA2G2A	6.33E-19	1.993202795	0.439	0.194	1.47E-14
Fibroblast	NOVA1	0	1.967060243	0.433	0.005	0
Fibroblast	PRELP	0	1.96575302	0.476	0.001	0
Fibroblast	ADAMTS1	0	1.954993196	0.402	0.001	0
Fibroblast	FGF7	0	1.954486247	0.445	0.002	0
Fibroblast	COL12A1	0	1.945115438	0.427	0	0
Fibroblast	NNMT	0	1.936556705	0.433	0.004	0
Fibroblast	MT2A	3.25E-17	1.901182026	0.561	0.337	7.56E-13
Fibroblast	UAP1	2.32E-24	1.890814835	0.488	0.247	5.39E-20
Fibroblast	WISP2	0	1.885763328	0.378	0.001	0
Fibroblast	LMNA	1.33E-35	1.88079038	0.671	0.379	3.10E-31
Fibroblast	GNG11	0	1.878602043	0.421	0.003	0

Fibroblast	TPPP3	4.67E-80	1.847416862	0.372	0.053	1.09E-75
Fibroblast	KLF2	4.66E-48	1.842388152	0.604	0.201	1.08E-43
Fibroblast	SDC2	0	1.839320153	0.463	0.007	0
Fibroblast	FILIP1L	8.94E-150	1.833042423	0.439	0.041	2.08E-145
Fibroblast	EGR1	7.68E-27	1.80788481	0.61	0.346	1.79E-22
Fibroblast	FXYD1	0	1.804421196	0.439	0.012	0
Fibroblast	PDGFRA	0	1.803414777	0.415	0.001	0
Fibroblast	FHL1	0	1.794709663	0.451	0.009	0
Fibroblast	DST	8.92E-51	1.770108945	0.634	0.239	2.07E-46
Fibroblast	COL5A1	0	1.765612361	0.433	0.001	0
Fibroblast	AC245595.1	1.63E-63	1.753713229	0.445	0.095	3.79E-59
Fibroblast	CREB5	1.85E-289	1.731565005	0.433	0.02	4.31E-285
Fibroblast	APP	3.00E-50	1.725082016	0.75	0.377	6.98E-46
Fibroblast	SEMA3C	2.12E-28	1.710730008	0.402	0.147	4.93E-24
Fibroblast	PPIC	8.74E-30	1.707122377	0.543	0.267	2.03E-25
Fibroblast	SPON2	7.57E-104	1.698841859	0.451	0.06	1.76E-99
Fibroblast	HSPG2	1.99E-73	1.69803188	0.433	0.078	4.62E-69
Fibroblast	CADM3	0	1.697434811	0.415	0	0
Fibroblast	C1QTNF3	7.76E-304	1.694489785	0.36	0.013	1.80E-299
Fibroblast	HSPB1	1.13E-44	1.692707804	0.805	0.442	2.63E-40
Fibroblast	ECM1	2.69E-196	1.690759989	0.451	0.033	6.26E-192
Fibroblast	CRYAB	0	1.684351993	0.335	0.002	0
Fibroblast	OLFML3	0	1.680440374	0.415	0.013	0
Fibroblast	NUPR1	4.07E-60	1.677722381	0.738	0.256	9.48E-56
Fibroblast	ADAMTSL4	0	1.673638635	0.47	0.008	0
Fibroblast	SHISA3	0	1.666641385	0.396	0.001	0
Fibroblast	TCEAL9	8.64E-37	1.663581324	0.512	0.198	2.01E-32
Fibroblast	ABI3BP	0	1.647032699	0.433	0.002	0
Fibroblast	LIMA1	3.89E-39	1.646818105	0.677	0.346	9.04E-35
Fibroblast	MMP14	4.32E-60	1.646488388	0.433	0.093	1.00E-55
Fibroblast	ELN	0	1.639102925	0.354	0.004	0
Fibroblast	WNT2B	9.56E-245	1.636686079	0.384	0.019	2.22E-240
Fibroblast	LTBP3	1.23E-49	1.63464434	0.463	0.123	2.85E-45
Fibroblast	ANXA5	2.24E-31	1.632811362	0.64	0.351	5.21E-27
Fibroblast	NFIA	1.69E-26	1.626361228	0.482	0.224	3.92E-22
Fibroblast	TUBA1A	2.67E-26	1.619295385	0.53	0.246	6.21E-22
Fibroblast	SRPX	0	1.603574061	0.335	0.007	0
Fibroblast	COL5A2	0	1.596857542	0.384	0.001	0
Fibroblast	EBF1	6.37E-280	1.596108859	0.415	0.019	1.48E-275
Fibroblast	DAB2	1.54E-45	1.580898653	0.488	0.15	3.57E-41
Fibroblast	LAMB2	1.65E-159	1.576169611	0.378	0.028	3.84E-155
Fibroblast	PAM	3.72E-92	1.568829189	0.476	0.076	8.65E-88
Fibroblast	ANXA1	3.57E-53	1.559525962	0.695	0.232	8.29E-49

Fibroblast	SELENOM	3.76E-39	1.558910577	0.537	0.195	8.74E-35
Fibroblast	SSC5D	0	1.554698228	0.384	0.003	0
Fibroblast	PLPP1	1.47E-45	1.546807167	0.451	0.125	3.42E-41
Fibroblast	S100A6	8.28E-46	1.537905273	0.951	0.824	1.93E-41
Fibroblast	MYL9	3.27E-120	1.533879084	0.384	0.038	7.61E-116
Fibroblast	TPM1	9.89E-28	1.530132857	0.677	0.395	2.30E-23
Fibroblast	CAV1	0	1.527231137	0.341	0.009	0
Fibroblast	JUND	2.03E-30	1.524906834	0.75	0.524	4.73E-26
Fibroblast	AXL	0	1.507988317	0.36	0.011	0
Fibroblast	BEX3	1.94E-46	1.504372179	0.427	0.111	4.51E-42
Fibroblast	PODN	0	1.501711169	0.341	0.001	0
Fibroblast	PROCR	4.61E-115	1.481678852	0.372	0.038	1.07E-110
Fibroblast	TNFAIP2	1.75E-81	1.48099823	0.335	0.041	4.06E-77
Fibroblast	SOCS3	3.38E-10	1.47919192	0.323	0.169	7.85E-06
Fibroblast	CD63	1.44E-39	1.473357101	0.866	0.713	3.34E-35
Fibroblast	DDR2	0	1.472661811	0.354	0.005	0
Fibroblast	ARHGAP29	6.13E-166	1.470248283	0.36	0.025	1.43E-161
Fibroblast	MARCKS	3.26E-14	1.468403966	0.555	0.405	7.57E-10
Fibroblast	EMILIN1	0	1.46753661	0.268	0.003	0
Fibroblast	CRISPLD2	3.94E-170	1.461692742	0.287	0.015	9.15E-166
Fibroblast	CPQ	4.83E-97	1.454910577	0.402	0.051	1.12E-92
Fibroblast	SLIT2	0	1.45285582	0.311	0.001	0
Fibroblast	F10	1.84E-279	1.450613396	0.317	0.011	4.27E-275
Fibroblast	PDGFRB	0	1.438388091	0.36	0.008	0
Fibroblast	HTRA3	0	1.435092315	0.256	0	0
Fibroblast	LAMA4	0	1.433981858	0.409	0.002	0
Fibroblast	SPTBN1	9.87E-21	1.419566952	0.518	0.302	2.30E-16
Fibroblast	SERPINH1	5.47E-26	1.408148283	0.409	0.161	1.27E-21
Fibroblast	CD99	1.26E-26	1.406162019	0.738	0.567	2.93E-22
Fibroblast	ITIH5	0	1.405663738	0.348	0.001	0
Fibroblast	BGN	0	1.401003646	0.28	0.001	0
Fibroblast	NFIC	8.22E-27	1.392877349	0.445	0.186	1.91E-22
Fibroblast	PSAP	6.12E-34	1.392434749	0.78	0.552	1.42E-29
Fibroblast	VKORC1	2.23E-17	1.391768528	0.482	0.284	5.18E-13
Fibroblast	CRABP2	0	1.391503877	0.305	0.004	0
Fibroblast	NFIX	3.62E-23	1.3856847	0.354	0.131	8.41E-19
Fibroblast	AKAP12	0	1.383444973	0.293	0.008	0
Fibroblast	REXO2	3.97E-18	1.38218524	0.524	0.33	9.23E-14
Fibroblast	TCF21	0	1.381840179	0.305	0.001	0
Fibroblast	DLC1	0	1.381667234	0.329	0.007	0
Fibroblast	TSHZ2	3.36E-90	1.372074686	0.28	0.026	7.82E-86
Fibroblast	OSR2	8.22E-151	1.371591996	0.329	0.023	1.91E-146
Fibroblast	EMP1	6.07E-58	1.360884759	0.433	0.091	1.41E-53

Fibroblast	PPL	1.04E-88	1.360483359	0.366	0.047	2.42E-84
Fibroblast	TSPAN4	1.18E-128	1.352337887	0.39	0.036	2.74E-124
Fibroblast	ZBTB20	1.56E-24	1.351601338	0.585	0.315	3.62E-20
Fibroblast	LAMC1	1.52E-45	1.341779046	0.317	0.063	3.55E-41
Fibroblast	PLD3	5.28E-19	1.337967523	0.445	0.226	1.23E-14
Fibroblast	QSOX1	3.47E-20	1.325733004	0.457	0.23	8.06E-16
Fibroblast	CD34	0	1.317494412	0.317	0.001	0
Fibroblast	EPB41L2	6.58E-21	1.309952222	0.457	0.224	1.53E-16
Fibroblast	NFIB	5.42E-14	1.305979076	0.366	0.196	1.26E-09
Fibroblast	LAPTM4A	7.93E-17	1.304911864	0.646	0.506	1.84E-12
Fibroblast	LHFPL6	2.96E-293	1.297411668	0.366	0.014	6.88E-289
Fibroblast	LRRC17	0	1.296122686	0.287	0.003	0
Fibroblast	WSB1	3.36E-24	1.293807238	0.683	0.455	7.82E-20
Fibroblast	COL8A1	0	1.28607603	0.274	0	0
Fibroblast	THBS3	1.45E-130	1.276217839	0.305	0.022	3.38E-126
Fibroblast	EID1	6.76E-17	1.269081146	0.634	0.497	1.57E-12
Fibroblast	ITGB5	1.29E-37	1.268581332	0.354	0.091	3.01E-33
Fibroblast	KLF4	2.59E-16	1.266620601	0.488	0.288	6.01E-12
Fibroblast	EFEMP2	0	1.258891629	0.305	0.004	0
Fibroblast	MEDAG	0	1.256049465	0.305	0	0
Fibroblast	SVEP1	0	1.25152225	0.305	0.002	0
Fibroblast	TNS2	4.80E-123	1.250630273	0.256	0.017	1.12E-118
Fibroblast	NR4A1	2.92E-05	1.249484645	0.384	0.313	0.677922379
Fibroblast	THBS2	0	1.24207223	0.305	0	0
Fibroblast	ITM2A	6.56E-28	1.240698188	0.537	0.216	1.53E-23
Fibroblast	TGFBR3	2.45E-74	1.236351192	0.323	0.042	5.69E-70
Fibroblast	TCEAL4	2.59E-15	1.234015851	0.39	0.201	6.02E-11
Fibroblast	CHRDL1	0	1.224921766	0.268	0	0
Fibroblast	LGALS3BP	9.46E-22	1.223415343	0.579	0.345	2.20E-17
Fibroblast	DPYSL2	1.16E-34	1.223317263	0.402	0.119	2.69E-30
Fibroblast	DCLK1	0	1.219280369	0.293	0	0
Fibroblast	DBN1	4.89E-68	1.218585085	0.366	0.057	1.14E-63
Fibroblast	C16orf89	0	1.218411285	0.268	0.006	0
Fibroblast	ABCA6	0	1.217105031	0.256	0.005	0
Fibroblast	TUBB6	0	1.210960116	0.305	0.009	0
Fibroblast	IGF1	2.31E-143	1.21075845	0.25	0.013	5.37E-139
Fibroblast	FBLN5	1.08E-180	1.206597805	0.299	0.015	2.50E-176
Fibroblast	SCPEP1	1.88E-36	1.20604732	0.396	0.11	4.36E-32
Fibroblast	PALM	0	1.205987937	0.305	0.002	0
Fibroblast	MEIS2	2.81E-229	1.197119422	0.293	0.011	6.54E-225
Fibroblast	TENT5A	1.73E-22	1.193979922	0.427	0.18	4.02E-18
Fibroblast	MXRA8	0	1.18763038	0.28	0	0
Fibroblast	DNM1	3.13E-209	1.185626997	0.256	0.009	7.29E-205

Fibroblast	PLXDC2	2.02E-40	1.184433147	0.323	0.071	4.69E-36
Fibroblast	VAT1	4.27E-48	1.179302798	0.317	0.06	9.93E-44
Fibroblast	CLDN11	0	1.17853388	0.293	0.003	0
Fibroblast	RSPO3	0	1.168002207	0.28	0	0
Fibroblast	AKR1C1	3.85E-251	1.166309112	0.305	0.011	8.95E-247
Fibroblast	RTL8C	1.17E-29	1.164384455	0.274	0.066	2.73E-25
Fibroblast	THBS1	8.78E-35	1.156669774	0.28	0.06	2.04E-30
Fibroblast	GAS6	2.37E-06	1.156073447	0.329	0.233	0.055199535
Fibroblast	CNN3	3.87E-07	1.15057593	0.317	0.208	0.009009075
Fibroblast	TCF4	1.73E-33	1.150528845	0.348	0.087	4.02E-29
Fibroblast	ENG	2.45E-68	1.149706981	0.287	0.036	5.69E-64
Fibroblast	HCFC1R1	4.20E-14	1.148796201	0.341	0.166	9.77E-10
Fibroblast	SEMA3B	1.63E-17	1.14057539	0.262	0.091	3.78E-13
Fibroblast	FKBP10	3.99E-252	1.140106653	0.268	0.009	9.28E-248
Fibroblast	TWIST2	0	1.127717683	0.262	0	0
Fibroblast	ANXA2	1.96E-28	1.123725069	0.799	0.543	4.57E-24
Fibroblast	NID1	0	1.119775399	0.256	0.003	0
Fibroblast	DDAH2	1.31E-14	1.116155265	0.512	0.336	3.05E-10
Fibroblast	YBX3	4.03E-14	1.113303448	0.518	0.355	9.37E-10
Fibroblast	KCNQ1OT1	9.98E-06	1.11165557	0.274	0.18	0.232191896
Fibroblast	CD59	8.26E-12	1.100179121	0.451	0.306	1.92E-07
Fibroblast	RHOB	6.11E-08	1.098775118	0.47	0.358	0.00142195
Fibroblast	GPC6	0	1.098177039	0.25	0	0
Fibroblast	PTMS	5.54E-11	1.094699616	0.488	0.347	1.29E-06
Fibroblast	SSPN	9.95E-31	1.087556404	0.293	0.072	2.31E-26
Fibroblast	GAS7	6.84E-119	1.085606442	0.268	0.018	1.59E-114
Fibroblast	MIR99AHG	0	1.083188808	0.268	0.004	0
Fibroblast	PPP1R12B	6.87E-13	1.07900551	0.287	0.127	1.60E-08
Fibroblast	ITM2B	1.50E-28	1.0773951	0.872	0.799	3.50E-24
Fibroblast	HSPB6	0	1.075386473	0.268	0.002	0
Fibroblast	ITGB1	2.11E-12	1.073593043	0.537	0.403	4.90E-08
Fibroblast	CALU	4.40E-13	1.06872487	0.36	0.191	1.02E-08
Fibroblast	ANGPTL2	0	1.064732219	0.262	0	0
Fibroblast	TGFBR2	2.45E-06	1.061214106	0.299	0.189	0.056929651
Fibroblast	SGCE	2.41E-198	1.056534379	0.256	0.01	5.61E-194
Fibroblast	ZNF385A	3.59E-93	1.04845539	0.256	0.021	8.35E-89
Fibroblast	MGST1	1.68E-12	1.04329768	0.518	0.332	3.91E-08
Fibroblast	FERMT2	0	1.041795398	0.256	0.001	0
Fibroblast	CERCAM	2.73E-235	1.039049798	0.28	0.01	6.36E-231
Fibroblast	S100A10	2.10E-27	1.036547304	0.872	0.67	4.89E-23
Fibroblast	FNDC3B	4.05E-10	1.032959922	0.329	0.188	9.43E-06
Fibroblast	CTSL	2.94E-43	1.032475004	0.348	0.075	6.84E-39
Fibroblast	CTSF	3.57E-81	1.031800708	0.256	0.024	8.30E-77

Fibroblast	TMEM176B	1.25E-08	1.025864914	0.439	0.313	0.000291612
Fibroblast	CRTAP	2.15E-08	1.014055739	0.317	0.193	0.000500608
Fibroblast	FLNA	1.93E-21	1.011005385	0.396	0.152	4.48E-17
Fibroblast	PRSS23	3.93E-06	1.008759724	0.323	0.212	0.091313558
Fibroblast	ATP2B4	4.49E-26	1.008346156	0.287	0.077	1.04E-21
Fibroblast	RBMS1	2.38E-25	1.000387098	0.378	0.12	5.53E-21
Fibroblast	PTGES	1.17E-103	0.999289434	0.25	0.019	2.71E-99
Fibroblast	ZFP36	3.33E-05	0.99871953	0.646	0.655	0.773417293
Fibroblast	CLEC11A	1.05E-37	0.997523032	0.274	0.055	2.45E-33
Fibroblast	TRIOBP	2.52E-08	0.991957214	0.329	0.208	0.000585697
Fibroblast	RAB34	6.12E-80	0.986374388	0.268	0.027	1.42E-75
Fibroblast	NEAT1	1.76E-27	0.985992079	0.909	0.776	4.10E-23
Fibroblast	METRNL	7.88E-21	0.982067081	0.317	0.111	1.83E-16
Fibroblast	CBX6	5.66E-15	0.980011775	0.299	0.115	1.32E-10
Fibroblast	MYADM	3.27E-10	0.97687261	0.409	0.252	7.59E-06
Fibroblast	CRIP1	4.08E-10	0.972944909	0.61	0.478	9.49E-06
Fibroblast	TAGLN2	1.46E-09	0.969320318	0.628	0.557	3.39E-05
Fibroblast	EPHX1	9.49E-12	0.968588228	0.25	0.107	2.21E-07
Fibroblast	LEPROT	4.70E-08	0.964249936	0.427	0.32	0.001093665
Fibroblast	FAM114A1	8.17E-19	0.953237431	0.329	0.131	1.90E-14
Fibroblast	PAMR1	0	0.94883348	0.268	0	0
Fibroblast	SPTAN1	1.04E-05	0.94824366	0.335	0.242	0.241004179
Fibroblast	RRBP1	1.53E-10	0.941194882	0.543	0.415	3.57E-06
Fibroblast	ADD3	1.09E-06	0.930796498	0.433	0.349	0.025277281
Fibroblast	SESTD1	3.05E-15	0.929380762	0.25	0.09	7.09E-11
Fibroblast	PLAGL1	2.85E-08	0.928903305	0.262	0.139	0.000662619
Fibroblast	P4HA2	1.01E-11	0.928326046	0.25	0.108	2.35E-07
Fibroblast	METTL7A	4.39E-10	0.89939436	0.299	0.153	1.02E-05
Fibroblast	SELENOW	1.36E-07	0.899154588	0.537	0.473	0.00316366
Fibroblast	APOL1	3.24E-23	0.896686263	0.262	0.072	7.54E-19
Fibroblast	TUBB	1.90E-09	0.896395309	0.598	0.51	4.42E-05
Fibroblast	LY6E	4.61E-13	0.895903865	0.5	0.283	1.07E-08
Fibroblast	LINC01133	1.14E-12	0.89457183	0.317	0.151	2.64E-08
Fibroblast	ZEB2	3.62E-18	0.890438563	0.354	0.126	8.41E-14
Fibroblast	TCEAL8	0.000150005	0.884261429	0.323	0.255	1
Fibroblast	GNAS	2.56E-08	0.87520461	0.634	0.589	0.000596055
Fibroblast	DDX17	7.20E-13	0.871050595	0.683	0.595	1.67E-08
Fibroblast	SPRY1	5.56E-05	0.870911132	0.287	0.192	1
Fibroblast	OAF	1.36E-10	0.860783152	0.293	0.15	3.17E-06
Fibroblast	SEC31A	8.67E-05	0.8581768	0.329	0.254	1
Fibroblast	NUCKS1	1.69E-09	0.857189665	0.591	0.499	3.92E-05
Fibroblast	SYNE1	6.73E-31	0.854349585	0.323	0.079	1.56E-26
Fibroblast	MT1E	1.99E-07	0.850408909	0.402	0.239	0.004628038

Fibroblast	LAMP1	3.91E-08	0.849034458	0.53	0.453	0.000910063
Fibroblast	CEBPB	6.91E-06	0.845803323	0.476	0.4	0.160639097
Fibroblast	GLG1	0.000958351	0.844550288	0.335	0.283	1
Fibroblast	RNH1	1.13E-09	0.83220087	0.524	0.422	2.63E-05
Fibroblast	RTN4	6.70E-08	0.821430619	0.567	0.513	0.001558451
Fibroblast	ARID5B	0.000327944	0.820913487	0.335	0.268	1
Fibroblast	SCARB2	6.03E-05	0.820197126	0.341	0.269	1
Fibroblast	FTX	2.39E-05	0.818559045	0.341	0.248	0.554747261
Fibroblast	NENF	2.57E-05	0.805927034	0.463	0.408	0.597493288
Fibroblast	TUBA1B	6.87E-13	0.791747479	0.689	0.57	1.60E-08
Fibroblast	SLC25A37	3.85E-07	0.787673546	0.317	0.199	0.008943494
Fibroblast	GRN	2.19E-06	0.775275672	0.512	0.444	0.050943331
Fibroblast	PID1	1.14E-11	0.775059804	0.256	0.111	2.64E-07
Fibroblast	TXNIP	1.69E-10	0.770620788	0.756	0.669	3.93E-06
Fibroblast	RBPJ	1.05E-05	0.769304261	0.409	0.318	0.244717849
Fibroblast	DSTN	1.38E-06	0.768870546	0.616	0.521	0.032078089
Fibroblast	SPAG9	0.007489574	0.765536283	0.256	0.206	1
Fibroblast	GSTM3	9.28E-11	0.760321559	0.256	0.115	2.16E-06
Fibroblast	UTRN	0.000113241	0.759997545	0.268	0.178	1
Fibroblast	TPM4	2.86E-08	0.755239481	0.561	0.47	0.000665323
Fibroblast	FCGRT	3.59E-06	0.754034354	0.47	0.396	0.083597285
Fibroblast	NPC2	1.41E-06	0.749167356	0.555	0.498	0.032753119
Fibroblast	ADD1	4.36E-06	0.745177989	0.348	0.243	0.101349389
Fibroblast	TNFRSF1A	0.000982687	0.738698029	0.317	0.256	1
Fibroblast	IFITM2	2.68E-10	0.734153867	0.61	0.457	6.22E-06
Fibroblast	CYB5R3	0.006720236	0.733495376	0.262	0.214	1
Fibroblast	NFE2L2	0.009371612	0.72830936	0.39	0.38	1
Fibroblast	SELENOP	2.68E-19	0.71769628	0.421	0.179	6.22E-15
Fibroblast	SDCBP	6.90E-06	0.715158391	0.53	0.472	0.160375655
Fibroblast	S100A11	6.83E-17	0.714739895	0.823	0.653	1.59E-12
Fibroblast	RPS27L	6.57E-07	0.703017393	0.646	0.632	0.015279348
Fibroblast	CAST	1.62E-05	0.702024658	0.53	0.505	0.3755693
Fibroblast	DYNLL1	3.71E-09	0.701671108	0.72	0.64	8.62E-05
Fibroblast	ATL3	0.000135655	0.694873721	0.256	0.174	1
Fibroblast	G0S2	2.08E-54	0.694421165	0.335	0.057	4.84E-50
Fibroblast	PRNP	1.80E-12	0.691364443	0.299	0.127	4.19E-08
Fibroblast	NKTR	9.66E-05	0.682608359	0.494	0.436	1
Fibroblast	DYNC1H1	0.000230095	0.67709806	0.402	0.344	1
Fibroblast	PLEC	0.004306711	0.676233689	0.274	0.222	1
Fibroblast	UBXN6	0.007741947	0.66906169	0.268	0.226	1
Fibroblast	PRCP	1.06E-06	0.668406233	0.262	0.151	0.024578897
Fibroblast	TRIM8	0.001922536	0.66103237	0.274	0.212	1
Fibroblast	CD151	0.002027368	0.657557976	0.427	0.402	1

Fibroblast	CAPNS1	0.004157554	0.655214315	0.372	0.35	1
Fibroblast	PIGT	0.003815292	0.651571873	0.354	0.331	1
Fibroblast	C1orf21	0.003854945	0.648160658	0.262	0.211	1
Fibroblast	RHOA	2.47E-06	0.640301434	0.671	0.686	0.05733209
Fibroblast	GOLIM4	0.000960145	0.637524248	0.396	0.339	1
Fibroblast	APLP2	7.55E-05	0.628903913	0.457	0.4	1
Fibroblast	CCPG1	0.003233567	0.617125139	0.299	0.246	1
Fibroblast	RABAC1	0.000750023	0.613760521	0.53	0.536	1
Fibroblast	TACC1	0.00895068	0.613051808	0.317	0.277	1
Fibroblast	HP1BP3	0.005296098	0.605680247	0.439	0.425	1
Fibroblast	NUCB2	0.007663175	0.600104596	0.348	0.307	1
Fibroblast	ZYX	0.001808187	0.597009675	0.28	0.215	1
Fibroblast	OAT	0.009029709	0.590232459	0.256	0.212	1
Fibroblast	MT-CYB	2.88E-16	0.572332295	0.982	0.985	6.70E-12
Fibroblast	N4BP2L2	2.87E-08	0.570761891	0.726	0.713	0.000666944
Fibroblast	ZFP36L1	0.000135891	0.559840166	0.61	0.587	1
Fibroblast	MT-ND4L	0.00850624	0.55662094	0.555	0.583	1
Fibroblast	PPIB	2.09E-06	0.553205841	0.677	0.636	0.048688272
Fibroblast	PRDX1	2.49E-07	0.553068258	0.671	0.568	0.005794024
Fibroblast	TAX1BP3	0.007804514	0.54944377	0.329	0.296	1
Fibroblast	FKBP2	0.009051981	0.53563179	0.5	0.522	1
Fibroblast	RAB13	0.003208761	0.52797398	0.14	0.271	1
Fibroblast	SEC62	0.002398692	0.521650365	0.561	0.618	1
Fibroblast	FUS	2.08E-05	0.520340278	0.634	0.643	0.484597758
Fibroblast	MALAT1	6.41E-18	0.517965991	1	0.996	1.49E-13
Fibroblast	NBL1	0.008133344	0.513654525	0.39	0.362	1
Fibroblast	HSPA1A	4.45E-06	0.490910868	0.372	0.252	0.103418622
Fibroblast	MGST3	0.000596963	0.4611565	0.524	0.473	1
Fibroblast	MACF1	0.009962572	0.452128972	0.329	0.28	1
Fibroblast	SMIM14	0.003276367	0.442728375	0.409	0.369	1
Fibroblast	TMSB10	4.94E-13	0.440381595	0.97	0.962	1.15E-08
Fibroblast	SERF2	3.33E-06	0.432282281	0.902	0.93	0.077331805
Fibroblast	POLR2L	0.001901577	0.410871041	0.646	0.684	1
Fibroblast	SFPQ	0.003045284	0.399322763	0.579	0.589	1
Fibroblast	CALM2	4.25E-05	0.375628647	0.823	0.819	0.989401201
Fibroblast	HSP90B1	0.000372557	0.36962346	0.64	0.613	1
Fibroblast	MT-ND3	2.35E-09	0.360314199	0.982	0.985	5.46E-05
Fibroblast	FABP5	5.74E-07	0.353905497	0.104	0.308	0.013348071
Fibroblast	MYL6	8.13E-05	0.345917513	0.835	0.894	1
Fibroblast	ACTG1	0.000103651	0.337415339	0.909	0.909	1
Fibroblast	UBC	0.000783033	0.32933017	0.835	0.843	1
Fibroblast	VSIR	0.006482314	0.321162727	0.256	0.187	1
Fibroblast	ATP5ME	0.000554496	0.318888465	0.75	0.706	1

Fibroblast	NDUFA8	0.00024111	0.314482692	0.159	0.329	1
Fibroblast	IRF3	0.004043837	0.312302819	0.165	0.294	1
Fibroblast	TMEM208	0.008489572	0.308734151	0.189	0.328	1
Fibroblast	MT-ND2	1.18E-09	0.307905316	0.988	0.989	2.75E-05
Follicular B cell	MS4A1	0	3.575337892	0.892	0.02	0
Follicular B cell	CD74	0	3.312219425	0.996	0.594	0
Follicular B cell	CD79B	0	3.260107447	0.855	0.055	0
Follicular B cell	CD79A	0	3.243038063	0.889	0.061	0
Follicular B cell	VPREB3	0	3.102454192	0.768	0.014	0
Follicular B cell	HLA-DRA	0	2.898989972	0.993	0.338	0
Follicular B cell	HLA-DQB1	0	2.771250938	0.886	0.151	0
Follicular B cell	CXCR4	0	2.724049847	0.92	0.28	0
Follicular B cell	BANK1	0	2.55591216	0.686	0.025	0
Follicular B cell	LTB	0	2.511831416	0.895	0.188	0
Follicular B cell	HLA-DPB1	0	2.457949608	0.949	0.262	0
Follicular B cell	TCL1A	0	2.379584349	0.372	0.01	0
Follicular B cell	HLA-DRB1	0	2.36364244	0.96	0.343	0
Follicular B cell	HLA-DQA1	0	2.362546097	0.749	0.058	0
Follicular B cell	MEF2C	0	2.266210106	0.699	0.108	0
Follicular B cell	HLA-DPA1	0	2.246167115	0.913	0.248	0
Follicular B cell	CD37	0	2.235364764	0.878	0.28	0
Follicular B cell	SMIM14	0	2.032764986	0.632	0.331	0
Follicular B cell	IGHD	0	1.868410553	0.303	0.003	0
Follicular B cell	TNFRSF13C	0	1.821171916	0.484	0.018	0
Follicular B cell	IGHM	0	1.786260375	0.587	0.119	0
Follicular B cell	CD22	0	1.778594035	0.422	0.007	0
Follicular B cell	HLA-DMA	0	1.763925744	0.649	0.211	0
Follicular B cell	HLA-DMB	0	1.745619815	0.544	0.092	0
Follicular B cell	ADAM28	0	1.656345905	0.455	0.068	0
Follicular B cell	POU2F2	0	1.637818097	0.483	0.075	0
Follicular B cell	LINC01781	0	1.614339917	0.286	0.008	0
Follicular B cell	IRF8	0	1.584467739	0.471	0.132	0
Follicular B cell	BCL11A	0	1.574907576	0.464	0.193	0
Follicular B cell	CD19	0	1.559678543	0.402	0.014	0
Follicular B cell	LY86	0	1.539494474	0.442	0.04	0
Follicular B cell	FCRLA	0	1.525404428	0.377	0.009	0
Follicular B cell	HVCN1	0	1.494102999	0.379	0.03	0
Follicular B cell	FCER2	0	1.470177275	0.295	0.009	0
Follicular B cell	LAPTM5	0	1.463228897	0.796	0.339	0
Follicular B cell	ORAI2	0	1.437259844	0.449	0.123	0
Follicular B cell	CD83	0	1.420372129	0.339	0.055	0
Follicular B cell	TCF4	0	1.419462895	0.377	0.046	0
Follicular B cell	SWAP70	0	1.416960255	0.4	0.112	0

Follicular B cell	GAPT	0	1.414753636	0.359	0.017	0
Follicular B cell	TLR10	0	1.41047379	0.349	0.007	0
Follicular B cell	LYL1	0	1.40410024	0.378	0.039	0
Follicular B cell	LINC00926	0	1.382142999	0.347	0.088	0
Follicular B cell	LINC02397	0	1.356896919	0.305	0.004	0
Follicular B cell	GPR183	0	1.34775638	0.424	0.124	0
Follicular B cell	ARHGAP24	0	1.30051067	0.316	0.039	0
Follicular B cell	SELL	0	1.2774107	0.319	0.038	0
Follicular B cell	DRAM2	0	1.275172273	0.491	0.289	0
Follicular B cell	RALGPS2	0	1.258125774	0.332	0.108	0
Follicular B cell	HLA-DOB	0	1.231634794	0.302	0.016	0
Follicular B cell	CD40	0	1.231221063	0.309	0.034	0
Follicular B cell	RIPOR2	0	1.218289841	0.41	0.083	0
Follicular B cell	CD53	0	1.218103288	0.672	0.291	0
Follicular B cell	STX7	8.20E-263	1.212523855	0.4	0.23	1.91E-258
Follicular B cell	NCF1	0	1.206151966	0.506	0.16	0
Follicular B cell	PLEKHF2	0	1.204162823	0.382	0.142	0
Follicular B cell	TMEM154	0	1.188464999	0.363	0.082	0
Follicular B cell	LIMD2	0	1.182084179	0.616	0.269	0
Follicular B cell	PHACTR1	0	1.181431922	0.281	0.05	0
Follicular B cell	FCMR	0	1.176815212	0.333	0.058	0
Follicular B cell	BLK	0	1.17640846	0.291	0.01	0
Follicular B cell	BTG1	0	1.173746325	0.958	0.831	0
Follicular B cell	TNFRSF13B	0	1.168833642	0.261	0.021	0
Follicular B cell	FCRL2	0	1.157150929	0.257	0.018	0
Follicular B cell	RPS27	0	1.156540789	0.988	0.982	0
Follicular B cell	BIRC3	0	1.153852034	0.431	0.207	0
Follicular B cell	BASP1	0	1.148319141	0.314	0.065	0
Follicular B cell	RNASE6	0	1.115134801	0.324	0.081	0
Follicular B cell	HHEX	0	1.109614172	0.286	0.029	0
Follicular B cell	CD52	0	1.104768872	0.957	0.478	0
Follicular B cell	SYPL1	4.96E-202	1.092333099	0.492	0.391	1.15E-197
Follicular B cell	CHI3L2	0	1.089655436	0.251	0.029	0
Follicular B cell	POU2AF1	0	1.081253505	0.281	0.041	0
Follicular B cell	REL	2.08E-263	1.072062882	0.488	0.31	4.83E-259
Follicular B cell	GNG7	0	1.046076952	0.272	0.042	0
Follicular B cell	SNX2	2.88E-190	1.027757405	0.415	0.282	6.70E-186
Follicular B cell	BLNK	0	1.012172803	0.272	0.098	0
Follicular B cell	TNFAIP8	0	1.004752393	0.497	0.237	0
Follicular B cell	PPM1K	0	1.001690155	0.362	0.13	0
Follicular B cell	BTK	0	0.998680051	0.257	0.032	0
Follicular B cell	MBD4	2.96E-158	0.997911959	0.368	0.242	6.87E-154
Follicular B cell	SPIB	0	0.989584711	0.278	0.029	0

Follicular B cell	SP110	0	0.988131757	0.42	0.204	0
Follicular B cell	QRSL1	2.04E-188	0.986756336	0.288	0.148	4.76E-184
Follicular B cell	LY9	0	0.982335178	0.253	0.058	0
Follicular B cell	RPS8	0	0.972640043	0.975	0.955	0
Follicular B cell	TMEM243	6.14E-192	0.971372275	0.43	0.294	1.43E-187
Follicular B cell	KLF2	3.85E-236	0.96693609	0.366	0.178	8.94E-232
Follicular B cell	SMARCB1	1.38E-105	0.963655656	0.395	0.331	3.21E-101
Follicular B cell	PRKCB	0	0.963145371	0.313	0.085	0
Follicular B cell	RPL17	0	0.961521243	0.893	0.818	0
Follicular B cell	CCR6	0	0.949256937	0.252	0.035	0
Follicular B cell	EVI2B	0	0.941742971	0.541	0.252	0
Follicular B cell	RPL18A	0	0.941358441	0.978	0.957	0
Follicular B cell	CD69	0	0.935306304	0.662	0.376	0
Follicular B cell	TSC22D3	0	0.927181473	0.643	0.361	0
Follicular B cell	RPL39	0	0.923830692	0.976	0.965	0
Follicular B cell	STK17A	0	0.921011525	0.668	0.397	0
Follicular B cell	TXNIP	8.67E-250	0.89289613	0.728	0.661	2.02E-245
Follicular B cell	RPL34	0	0.890638353	0.982	0.977	0
Follicular B cell	SNX3	1.50E-280	0.890361963	0.667	0.599	3.48E-276
Follicular B cell	RPL21	0	0.889858724	0.985	0.973	0
Follicular B cell	RCSD1	0	0.883271496	0.366	0.138	0
Follicular B cell	GPSM3	0	0.876689382	0.539	0.283	0
Follicular B cell	RHOH	0	0.872400276	0.408	0.168	0
Follicular B cell	LAT2	0	0.871996246	0.313	0.101	0
Follicular B cell	RASGRP2	0	0.870581192	0.259	0.047	0
Follicular B cell	RPLP2	0	0.870056272	0.985	0.973	0
Follicular B cell	FAU	0	0.861092238	0.97	0.949	0
Follicular B cell	UCP2	8.51E-149	0.854255286	0.4	0.27	1.98E-144
Follicular B cell	PKIG	6.98E-130	0.847713894	0.252	0.143	1.62E-125
Follicular B cell	CD27	0	0.847568226	0.305	0.093	0
Follicular B cell	SP140	0	0.846126176	0.275	0.068	0
Follicular B cell	RPL32	0	0.844469518	0.982	0.967	0
Follicular B cell	RPL23A	0	0.841630336	0.973	0.95	0
Follicular B cell	DCK	9.77E-196	0.838471603	0.379	0.216	2.27E-191
Follicular B cell	TMEM156	0	0.832610154	0.254	0.056	0
Follicular B cell	POLD4	1.15E-79	0.826922702	0.368	0.301	2.68E-75
Follicular B cell	RPS23	0	0.826280876	0.974	0.944	0
Follicular B cell	GGA2	7.92E-172	0.821986138	0.259	0.123	1.84E-167
Follicular B cell	IFI16	2.22E-261	0.820040556	0.572	0.371	5.17E-257
Follicular B cell	CCDC50	4.35E-73	0.818265544	0.296	0.223	1.01E-68
Follicular B cell	SEL1L3	5.30E-48	0.813076042	0.317	0.276	1.23E-43
Follicular B cell	HERPUD1	1.99E-118	0.811266682	0.56	0.513	4.62E-114
Follicular B cell	PARP1	6.87E-52	0.809425631	0.392	0.373	1.60E-47

Follicular B cell	INPP5D	2.59E-205	0.807407115	0.331	0.163	6.03E-201
Follicular B cell	RPS5	0	0.797237362	0.963	0.916	0
Follicular B cell	RPL10	0	0.791304831	0.992	0.981	0
Follicular B cell	RPS17	0	0.790123221	0.942	0.895	0
Follicular B cell	RPS25	0	0.786594635	0.966	0.946	0
Follicular B cell	RPL36A	0	0.78485256	0.924	0.872	0
Follicular B cell	PNISR	7.23E-269	0.782182352	0.73	0.688	1.68E-264
Follicular B cell	RPL11	0	0.780686565	0.985	0.968	0
Follicular B cell	AC004687.1	9.91E-232	0.773220772	0.296	0.12	2.30E-227
Follicular B cell	RPL13A	0	0.770307975	0.99	0.979	0
Follicular B cell	EIF2S3	5.29E-91	0.769099564	0.457	0.42	1.23E-86
Follicular B cell	SKIL	9.92E-50	0.766555953	0.343	0.305	2.31E-45
Follicular B cell	RPS15A	0	0.759510602	0.976	0.961	0
Follicular B cell	NAP1L1	2.66E-283	0.743589473	0.75	0.669	6.17E-279
Follicular B cell	RPS10	0	0.72544333	0.951	0.915	0
Follicular B cell	RPS28	0	0.723207575	0.974	0.965	0
Follicular B cell	ATM	1.38E-120	0.721783438	0.359	0.234	3.22E-116
Follicular B cell	ISG20	4.22E-73	0.720077024	0.384	0.293	9.82E-69
Follicular B cell	BCAS4	8.50E-180	0.715163465	0.262	0.117	1.98E-175
Follicular B cell	RGS19	4.74E-236	0.710212249	0.298	0.122	1.10E-231
Follicular B cell	RPL19	0	0.703775482	0.975	0.956	0
Follicular B cell	RPS27A	0	0.703101507	0.981	0.964	0
Follicular B cell	XIST	8.31E-175	0.690579249	0.346	0.176	1.93E-170
Follicular B cell	PNRC1	6.65E-226	0.684504784	0.72	0.641	1.55E-221
Follicular B cell	ANKRD44	3.13E-181	0.674321065	0.365	0.188	7.29E-177
Follicular B cell	CTSH	1.21E-32	0.670872062	0.297	0.272	2.82E-28
Follicular B cell	EIF3E	5.88E-272	0.661477598	0.812	0.72	1.37E-267
Follicular B cell	RPS11	0	0.660658174	0.958	0.926	0
Follicular B cell	NOP53	1.42E-249	0.660652178	0.803	0.756	3.30E-245
Follicular B cell	RPL27A	0	0.660011779	0.979	0.964	0
Follicular B cell	RPL26	0	0.659518407	0.972	0.954	0
Follicular B cell	SP100	2.29E-58	0.65816274	0.4	0.357	5.32E-54
Follicular B cell	CORO1A	0	0.653189138	0.808	0.465	0
Follicular B cell	RPS29	0	0.652593444	0.977	0.972	0
Follicular B cell	ATP6V1G1	2.52E-191	0.650756298	0.7	0.695	5.87E-187
Follicular B cell	ATP2B1	6.15E-30	0.649983885	0.342	0.33	1.43E-25
Follicular B cell	EEF1G	5.80E-27	0.644064805	0.356	0.367	1.35E-22
Follicular B cell	LYN	2.18E-54	0.643564462	0.255	0.189	5.08E-50
Follicular B cell	RPS21	0	0.642685194	0.941	0.902	0
Follicular B cell	MDM4	5.16E-28	0.64211628	0.366	0.357	1.20E-23
Follicular B cell	ARID5B	9.83E-20	0.641896373	0.278	0.267	2.29E-15
Follicular B cell	SMDT1	5.43E-93	0.640721023	0.554	0.573	1.26E-88
Follicular B cell	MYCBP2	1.08E-45	0.640071748	0.342	0.296	2.51E-41

Follicular B cell	TRIM38	2.64E-34	0.63715414	0.304	0.268	6.14E-30
Follicular B cell	RPS4X	0	0.636785553	0.979	0.953	0
Follicular B cell	EIF4A2	3.89E-250	0.635743571	0.786	0.756	9.04E-246
Follicular B cell	ITSN2	3.91E-42	0.634673217	0.362	0.324	9.10E-38
Follicular B cell	SEC62	2.00E-75	0.633676002	0.581	0.623	4.66E-71
Follicular B cell	RPSA	0	0.632001285	0.951	0.89	0
Follicular B cell	TRAF5	4.68E-61	0.631939196	0.251	0.175	1.09E-56
Follicular B cell	SKAP2	3.94E-20	0.626859574	0.313	0.315	9.16E-16
Follicular B cell	EEF2	0	0.62232313	0.902	0.832	0
Follicular B cell	RPL13	0	0.620472403	0.989	0.975	0
Follicular B cell	RPS15	0	0.61002075	0.981	0.969	0
Follicular B cell	RPL38	0	0.602267002	0.945	0.934	0
Follicular B cell	RPL35A	0	0.596560464	0.966	0.952	0
Follicular B cell	DUSP1	3.55E-107	0.593982636	0.695	0.672	8.25E-103
Follicular B cell	RPL15	0	0.590615945	0.974	0.95	0
Follicular B cell	EBLN3P	3.74E-10	0.588864934	0.27	0.281	8.70E-06
Follicular B cell	RPL9	0	0.588660359	0.966	0.943	0
Follicular B cell	GRB2	2.09E-23	0.587947473	0.349	0.352	4.87E-19
Follicular B cell	ANAPC16	6.27E-142	0.586187131	0.666	0.67	1.46E-137
Follicular B cell	EEF1B2	3.99E-235	0.585096189	0.876	0.817	9.29E-231
Follicular B cell	DEGS1	7.85E-76	0.584953768	0.271	0.177	1.83E-71
Follicular B cell	TCEA1	6.49E-18	0.58380319	0.431	0.497	1.51E-13
Follicular B cell	CNPY3	1.39E-25	0.580735339	0.312	0.295	3.24E-21
Follicular B cell	ANXA6	8.81E-120	0.579743222	0.333	0.196	2.05E-115
Follicular B cell	RPL12	0	0.574451014	0.968	0.961	0
Follicular B cell	RPL3	0	0.573627899	0.989	0.961	0
Follicular B cell	TAGAP	1.12E-143	0.572502676	0.25	0.115	2.60E-139
Follicular B cell	LYSMD2	2.68E-38	0.570534128	0.259	0.206	6.23E-34
Follicular B cell	ZBTB20	3.33E-18	0.566535767	0.32	0.315	7.75E-14
Follicular B cell	UBA52	0	0.559945185	0.947	0.934	0
Follicular B cell	AC114760.2	2.51E-132	0.559788504	0.279	0.14	5.83E-128
Follicular B cell	CYBA	1.92E-301	0.554192602	0.943	0.882	4.47E-297
Follicular B cell	CD48	4.72E-181	0.553871667	0.484	0.275	1.10E-176
Follicular B cell	ARHGDIB	4.59E-211	0.552091768	0.74	0.51	1.07E-206
Follicular B cell	SH3BGRL	4.19E-52	0.551908289	0.468	0.457	9.75E-48
Follicular B cell	RPL10A	0	0.550847103	0.97	0.924	0
Follicular B cell	CCNI	3.35E-163	0.549442865	0.762	0.759	7.79E-159
Follicular B cell	RPS20	0	0.549025171	0.954	0.933	0
Follicular B cell	RPL18	0	0.548860403	0.967	0.945	0
Follicular B cell	ARHGAP15	6.31E-156	0.547372144	0.288	0.136	1.47E-151
Follicular B cell	TNRC6B	4.79E-28	0.546652003	0.423	0.43	1.11E-23
Follicular B cell	ZNF791	7.61E-26	0.546360706	0.268	0.233	1.77E-21
Follicular B cell	PFDN5	9.20E-307	0.545857242	0.908	0.891	2.14E-302

Follicular B cell	RPL35	0	0.542526415	0.964	0.95	0
Follicular B cell	TOMM7	5.43E-175	0.54235962	0.777	0.775	1.26E-170
Follicular B cell	LBH	6.06E-157	0.536255663	0.294	0.138	1.41E-152
Follicular B cell	RPS14	0	0.532238084	0.977	0.97	0
Follicular B cell	DBNL	5.24E-10	0.527449502	0.32	0.347	1.22E-05
Follicular B cell	RAC2	7.50E-168	0.527018639	0.573	0.372	1.74E-163
Follicular B cell	NR4A2	3.55E-30	0.526756253	0.355	0.308	8.27E-26
Follicular B cell	SNAP23	4.67E-12	0.523359627	0.283	0.289	1.09E-07
Follicular B cell	BLOC1S2	3.77E-13	0.522400731	0.277	0.273	8.77E-09
Follicular B cell	YPEL5	1.18E-19	0.520858254	0.358	0.357	2.73E-15
Follicular B cell	CIRBP	3.18E-136	0.51784461	0.725	0.727	7.40E-132
Follicular B cell	SLC2A3	9.60E-189	0.516906193	0.352	0.167	2.23E-184
Follicular B cell	EEF1D	9.45E-266	0.515578961	0.923	0.901	2.20E-261
Follicular B cell	RPL41	0	0.513543166	0.991	0.988	0
Follicular B cell	RPL23	2.86E-258	0.510933227	0.922	0.877	6.64E-254
Follicular B cell	GDI2	7.34E-27	0.510652153	0.47	0.532	1.71E-22
Follicular B cell	PTPN6	1.82E-65	0.510615076	0.375	0.282	4.24E-61
Follicular B cell	RPL4	1.75E-276	0.510070947	0.933	0.881	4.07E-272
Follicular B cell	OGA	3.92E-17	0.508966617	0.381	0.4	9.12E-13
Follicular B cell	RPS16	0	0.508713731	0.967	0.945	0
Follicular B cell	CYBC1	1.92E-22	0.503370522	0.264	0.232	4.46E-18
Follicular B cell	RPS6	0	0.496307708	0.983	0.971	0
Follicular B cell	SNHG7	1.79E-06	0.494821	0.308	0.347	0.041616102
Follicular B cell	RPS13	0	0.494770581	0.962	0.937	0
Follicular B cell	RPS9	0	0.490793302	0.973	0.951	0
Follicular B cell	STK4	2.73E-50	0.48943764	0.493	0.453	6.35E-46
Follicular B cell	RPL31	0	0.487859897	0.966	0.947	0
Follicular B cell	RPL30	0	0.486485143	0.965	0.944	0
Follicular B cell	JMJD1C	4.34E-07	0.48451112	0.334	0.373	0.010103749
Follicular B cell	HLA-E	6.27E-214	0.479781862	0.877	0.862	1.46E-209
Follicular B cell	UPF2	1.92E-08	0.478795614	0.327	0.356	0.00044556
Follicular B cell	RPS12	0	0.478559774	0.975	0.963	0
Follicular B cell	RPL37	0	0.475890109	0.958	0.941	0
Follicular B cell	IL16	2.26E-112	0.474872967	0.265	0.137	5.24E-108
Follicular B cell	EIF1B	6.40E-10	0.474037812	0.369	0.418	1.49E-05
Follicular B cell	RPL36	0	0.473842069	0.967	0.961	0
Follicular B cell	EIF3L	1.85E-52	0.467045966	0.551	0.575	4.29E-48
Follicular B cell	HNRNPDL	1.71E-67	0.465154148	0.608	0.641	3.98E-63
Follicular B cell	RPL14	6.29E-297	0.464143224	0.962	0.927	1.46E-292
Follicular B cell	DDX5	4.91E-217	0.463415839	0.892	0.881	1.14E-212
Follicular B cell	IKZF1	4.33E-106	0.46228186	0.373	0.225	1.01E-101
Follicular B cell	CMTM6	1.98E-08	0.458199612	0.387	0.448	0.000460324
Follicular B cell	RPL27	7.77E-246	0.457981079	0.94	0.918	1.81E-241

Follicular B cell	RPL8	0	0.457958733	0.979	0.963	0
Follicular B cell	EEF1A1	0	0.457448661	0.992	0.979	0
Follicular B cell	RPL5	1.01E-246	0.455496572	0.949	0.908	2.34E-242
Follicular B cell	MOB1A	2.25E-09	0.455431521	0.389	0.447	5.23E-05
Follicular B cell	RACK1	2.91E-240	0.453001255	0.95	0.909	6.78E-236
Follicular B cell	UBE2N	1.51E-10	0.451276442	0.39	0.449	3.51E-06
Follicular B cell	ARL6IP5	2.35E-41	0.450328281	0.533	0.55	5.47E-37
Follicular B cell	IFT57	0.000381327	0.449487211	0.188	0.255	1
Follicular B cell	RNASET2	5.10E-27	0.441808145	0.507	0.559	1.19E-22
Follicular B cell	CD55	1.14E-08	0.441385356	0.295	0.306	0.000264756
Follicular B cell	SMCHD1	3.40E-12	0.439309993	0.332	0.336	7.90E-08
Follicular B cell	ATRX	1.48E-15	0.439293522	0.446	0.506	3.44E-11
Follicular B cell	TAF1D	2.56E-05	0.438675089	0.377	0.45	0.594333948
Follicular B cell	SCAF11	1.89E-12	0.434491627	0.461	0.54	4.40E-08
Follicular B cell	SF1	1.32E-12	0.433029554	0.45	0.524	3.07E-08
Follicular B cell	DOCK8	4.04E-68	0.431072994	0.305	0.201	9.40E-64
Follicular B cell	TSPAN13	0.000292179	0.43036802	0.249	0.345	1
Follicular B cell	RPS18	0	0.428258924	0.989	0.976	0
Follicular B cell	PSMB8-AS1	5.88E-41	0.424372072	0.269	0.2	1.37E-36
Follicular B cell	RPL29	1.56E-250	0.42374885	0.959	0.939	3.62E-246
Follicular B cell	RPS19	8.17E-245	0.423265669	0.983	0.975	1.90E-240
Follicular B cell	RPL22	1.18E-198	0.414968072	0.936	0.893	2.75E-194
Follicular B cell	VAMP2	5.13E-30	0.413910568	0.5	0.523	1.19E-25
Follicular B cell	RSRP1	1.84E-25	0.410106559	0.519	0.572	4.29E-21
Follicular B cell	RBMX	3.73E-17	0.407896677	0.468	0.532	8.67E-13
Follicular B cell	RPL28	0	0.40358904	0.973	0.969	0
Follicular B cell	NPM1	1.08E-130	0.396973738	0.862	0.822	2.52E-126
Follicular B cell	RPS2	0	0.396244816	0.986	0.966	0
Follicular B cell	ACTR3	1.01E-30	0.391120139	0.573	0.638	2.34E-26
Follicular B cell	SNHG8	0.000164192	0.385884586	0.4	0.485	1
Follicular B cell	RPL7	6.40E-245	0.384751938	0.974	0.953	1.49E-240
Follicular B cell	ICAM3	1.02E-21	0.381945924	0.36	0.335	2.37E-17
Follicular B cell	FOXP1	0.001249025	0.380833311	0.309	0.426	1
Follicular B cell	IKZF3	8.37E-97	0.380632641	0.307	0.173	1.95E-92
Follicular B cell	SELENOF	6.73E-08	0.379812538	0.452	0.554	0.001565473
Follicular B cell	ERP29	6.73E-20	0.376683864	0.521	0.607	1.57E-15
Follicular B cell	EIF4B	2.02E-15	0.374169659	0.481	0.557	4.69E-11
Follicular B cell	FXYD5	2.72E-34	0.372991352	0.476	0.451	6.34E-30
Follicular B cell	RSL24D1	4.39E-09	0.372196502	0.453	0.543	0.000101991
Follicular B cell	SRSF5	4.01E-77	0.369930436	0.705	0.747	9.33E-73
Follicular B cell	OAZ1	8.39E-125	0.365303318	0.875	0.893	1.95E-120
Follicular B cell	CLK1	0.000324407	0.359834509	0.338	0.376	1
Follicular B cell	GLIPR1	1.51E-57	0.35932735	0.31	0.209	3.50E-53

Follicular B cell	IDS	4.14E-11	0.35708478	0.332	0.325	9.64E-07
Follicular B cell	VOPP1	7.60E-07	0.354249621	0.21	0.292	0.017675022
Follicular B cell	EIF3F	4.11E-26	0.350348908	0.553	0.615	9.55E-22
Follicular B cell	FNBP1	1.24E-27	0.34793906	0.368	0.316	2.88E-23
Follicular B cell	PSIP1	0.001582121	0.346999446	0.256	0.27	1
Follicular B cell	POLR2J3.1	1.64E-05	0.345585726	0.415	0.479	0.38204995
Follicular B cell	MTF2	0.000325357	0.339964354	0.195	0.255	1
Follicular B cell	TTC14	0.001408716	0.339073814	0.273	0.29	1
Follicular B cell	RGS2	3.77E-12	0.335491273	0.267	0.234	8.76E-08
Follicular B cell	NT5C	0.000199349	0.335198621	0.266	0.365	1
Follicular B cell	FAM49B	0.000458767	0.333419141	0.387	0.436	1
Follicular B cell	RPL24	8.23E-133	0.33250085	0.932	0.905	1.91E-128
Follicular B cell	LTA4H	1.63E-05	0.331487524	0.207	0.283	0.378984504
Follicular B cell	GPBP1	0.000552331	0.328911767	0.428	0.521	1
Follicular B cell	CXXC5	2.63E-06	0.328442644	0.212	0.293	0.061247247
Follicular B cell	JUNB	3.34E-07	0.327643925	0.691	0.803	0.007776781
Follicular B cell	SINHCAF	1.02E-05	0.327139302	0.216	0.298	0.236176362
Follicular B cell	UBXN1	0.00048436	0.325853807	0.435	0.539	1
Follicular B cell	ASH1L	0.004498106	0.323895167	0.297	0.394	1
Follicular B cell	MARCKSL1	8.58E-07	0.323477608	0.367	0.452	0.019942633
Follicular B cell	RPLP1	2.04E-212	0.323258869	0.984	0.983	4.75E-208
Follicular B cell	PPP1CC	0.006289394	0.316736896	0.339	0.468	1
Follicular B cell	CNOT7	0.001390896	0.313665588	0.26	0.347	1
Follicular B cell	RNMT	0.002814332	0.31163278	0.256	0.334	1
Follicular B cell	HCLS1	3.62E-52	0.311414634	0.314	0.214	8.41E-48
Follicular B cell	NT5C3A	2.99E-11	0.310290165	0.217	0.32	6.95E-07
Follicular B cell	HMGN1	1.52E-10	0.309517881	0.673	0.727	3.55E-06
Follicular B cell	GYPC	3.00E-32	0.308575442	0.305	0.231	6.98E-28
Follicular B cell	PPP1R15A	0.001014733	0.305116569	0.471	0.564	1
Follicular B cell	CDC40	4.21E-06	0.302539332	0.186	0.253	0.097940642
Follicular B cell	ILF3-DT	3.30E-06	0.302228237	0.202	0.269	0.076716943
Follicular B cell	FAM96A	3.55E-07	0.301066049	0.251	0.358	0.008266118
Follicular B cell	RSL1D1	0.006184813	0.294640963	0.41	0.497	1
Follicular B cell	PABPC1	3.40E-92	0.294471441	0.905	0.863	7.91E-88
Follicular B cell	ACADM	7.56E-09	0.292194289	0.181	0.256	0.000175827
Follicular B cell	SNHG25	0.00010642	0.289682295	0.202	0.267	1
Follicular B cell	STK17B	4.38E-59	0.281341375	0.533	0.42	1.02E-54
Follicular B cell	THUMPD3-AS1	5.55E-05	0.279631397	0.263	0.353	1
Follicular B cell	H3F3B	3.98E-70	0.279358275	0.921	0.943	9.25E-66
Follicular B cell	TRAF3IP3	1.43E-53	0.278078234	0.34	0.232	3.33E-49
Follicular B cell	PPHLN1	3.69E-09	0.277392259	0.224	0.317	8.59E-05
Follicular B cell	PNRC2	0.000101885	0.277340093	0.307	0.423	1

Follicular B cell	METAP2	5.90E-26	0.275405909	0.254	0.415	1.37E-21
Follicular B cell	LSP1	1.65E-78	0.274484024	0.526	0.363	3.84E-74
Follicular B cell	COMMD6	9.91E-36	0.2737786	0.717	0.763	2.31E-31
Follicular B cell	ARPC3	4.87E-35	0.273191281	0.73	0.775	1.13E-30
Follicular B cell	LNPEP	0.000348323	0.26832566	0.199	0.253	1
Follicular B cell	RPS7	1.07E-107	0.268146773	0.954	0.926	2.49E-103
Follicular B cell	CHD2	0.000400195	0.264764142	0.212	0.273	1
Follicular B cell	EIF3H	3.86E-15	0.26465027	0.575	0.632	8.98E-11
Follicular B cell	IRF2	8.91E-06	0.253363474	0.196	0.261	0.207224287
Follicular B cell	HNRNPC	1.18E-11	0.25130127	0.581	0.672	2.75E-07
Follicular B cell	SLC38A1	2.26E-15	0.251203925	0.247	0.373	5.25E-11
Macrophage	S100A8	0	5.693393721	0.655	0.04	0
Macrophage	S100A9	0	5.657269488	0.738	0.076	0
Macrophage	CXCL8	0	4.974264161	0.425	0.072	0
Macrophage	LYZ	0	4.424731597	0.91	0.293	0
Macrophage	TYROBP	0	4.061657282	0.944	0.097	0
Macrophage	TIMP1	0	4.040529406	0.766	0.342	0
Macrophage	AIF1	0	3.92672198	0.842	0.025	0
Macrophage	IL1B	0	3.764954652	0.446	0.026	0
Macrophage	FCN1	0	3.755740472	0.657	0.01	0
Macrophage	MNDA	0	3.724508461	0.76	0.02	0
Macrophage	FCER1G	0	3.450948347	0.904	0.111	0
Macrophage	LST1	0	3.38299885	0.842	0.045	0
Macrophage	CD14	0	3.303274263	0.722	0.034	0
Macrophage	MS4A6A	0	3.295798069	0.727	0.01	0
Macrophage	C1QA	0	3.275477719	0.303	0.015	0
Macrophage	G0S2	0	3.264101072	0.259	0.043	0
Macrophage	NAMPT	0	3.132597307	0.749	0.281	0
Macrophage	S100A12	0	3.114471525	0.391	0.004	0
Macrophage	VCAN	0	3.10433583	0.561	0.008	0
Macrophage	CTSS	0	3.02291938	0.899	0.526	0
Macrophage	SOD2	0	3.001877476	0.66	0.283	0
Macrophage	SRGN	0	2.945742359	0.962	0.432	0
Macrophage	S100A4	0	2.834850441	0.906	0.453	0
Macrophage	CST3	0	2.808410887	0.896	0.481	0
Macrophage	TYMP	0	2.746688117	0.821	0.27	0
Macrophage	HLA-DRA	0	2.74566449	0.911	0.384	0
Macrophage	LGALS1	0	2.734240931	0.861	0.286	0
Macrophage	PSAP	0	2.710539359	0.88	0.528	0
Macrophage	HLA-DRB1	0	2.691673611	0.849	0.389	0
Macrophage	AC020656.1	0	2.647334295	0.468	0.096	0
Macrophage	CSTA	0	2.612985515	0.649	0.01	0
Macrophage	BCL2A1	0	2.596735382	0.511	0.086	0

Macrophage	FTL	0	2.582856475	0.988	0.937	0
Macrophage	SAT1	0	2.533894199	0.951	0.704	0
Macrophage	IL1RN	0	2.476653005	0.263	0.019	0
Macrophage	NFKBIA	0	2.458046468	0.719	0.422	0
Macrophage	PLAUR	0	2.449001946	0.564	0.124	0
Macrophage	RGS2	0	2.424081124	0.726	0.2	0
Macrophage	HLA-DPA1	0	2.407069956	0.745	0.302	0
Macrophage	AP1S2	0	2.400384212	0.698	0.13	0
Macrophage	HLA-DPB1	0	2.388409623	0.761	0.319	0
Macrophage	FGL2	0	2.37701946	0.681	0.037	0
Macrophage	CXCL2	3.55E-79	2.358250927	0.252	0.135	8.25E-75
Macrophage	VIM	0	2.337680915	0.884	0.362	0
Macrophage	CYBB	0	2.32755891	0.664	0.044	0
Macrophage	PTGS2	0	2.315053713	0.3	0.014	0
Macrophage	NPC2	0	2.262406849	0.807	0.475	0
Macrophage	CTSB	0	2.198088678	0.671	0.339	0
Macrophage	EMP3	0	2.191572204	0.775	0.246	0
Macrophage	CPVL	0	2.169444037	0.557	0.011	0
Macrophage	EREG	2.06E-295	2.144566002	0.262	0.066	4.79E-291
Macrophage	CCL3	0	2.09418307	0.281	0.063	0
Macrophage	CD68	0	2.085531123	0.605	0.118	0
Macrophage	MS4A7	0	2.084925886	0.571	0.01	0
Macrophage	FTH1	0	2.084330004	0.997	0.964	0
Macrophage	IFI30	0	2.08063981	0.565	0.036	0
Macrophage	RETN	0	2.077808484	0.437	0.003	0
Macrophage	ANXA1	0	2.053894587	0.749	0.195	0
Macrophage	CEBPB	0	2.005732546	0.712	0.377	0
Macrophage	HSPA1A	8.19E-123	2.001887698	0.412	0.24	1.90E-118
Macrophage	CFP	0	1.993733681	0.53	0.004	0
Macrophage	SPI1	0	1.97408202	0.627	0.033	0
Macrophage	ITGB2	0	1.967147345	0.667	0.102	0
Macrophage	C1orf162	0	1.954458505	0.604	0.042	0
Macrophage	GRN	0	1.915430068	0.749	0.421	0
Macrophage	IGSF6	0	1.898294885	0.519	0.006	0
Macrophage	PLEK	0	1.883480293	0.488	0.067	0
Macrophage	CLEC12A	0	1.875777932	0.478	0.006	0
Macrophage	CLEC7A	0	1.841940623	0.538	0.007	0
Macrophage	TNFSF13B	0	1.834227011	0.557	0.031	0
Macrophage	SLC2A3	0	1.818313191	0.483	0.169	0
Macrophage	CLEC10A	0	1.806468693	0.332	0.003	0
Macrophage	ZEB2	0	1.788970148	0.637	0.088	0
Macrophage	CD74	0	1.753082918	0.895	0.626	0
Macrophage	FCGR2A	0	1.741237853	0.473	0.014	0

Macrophage	SOCS3	0	1.722565807	0.484	0.146	0
Macrophage	SAMHD1	0	1.701984316	0.626	0.127	0
Macrophage	DUSP1	0	1.690492653	0.889	0.659	0
Macrophage	GLUL	1.00E-296	1.689949653	0.576	0.322	2.34E-292
Macrophage	NEAT1	0	1.660539131	0.958	0.763	0
Macrophage	HLA-DQA1	0	1.651170481	0.433	0.125	0
Macrophage	FPR1	0	1.650311263	0.408	0.002	0
Macrophage	CD300E	0	1.649323826	0.362	0.001	0
Macrophage	COTL1	0	1.64823362	0.784	0.475	0
Macrophage	CTSD	1.09E-140	1.633976519	0.635	0.523	2.53E-136
Macrophage	CD36	0	1.632231839	0.426	0.003	0
Macrophage	CSF3R	0	1.62882656	0.414	0.003	0
Macrophage	HLA-DMA	0	1.627181222	0.643	0.238	0
Macrophage	S100A11	0	1.612042404	0.915	0.634	0
Macrophage	LY96	0	1.605233924	0.53	0.051	0
Macrophage	ASAH1	0	1.598193063	0.638	0.351	0
Macrophage	CTSL	0	1.595217376	0.312	0.059	0
Macrophage	FOS	0	1.586927828	0.898	0.768	0
Macrophage	CEBPD	0	1.58286877	0.579	0.284	0
Macrophage	RNASE6	0	1.556606286	0.554	0.078	0
Macrophage	HLA-DQB1	0	1.554381441	0.591	0.219	0
Macrophage	FCGRT	0	1.554289926	0.687	0.374	0
Macrophage	CD44	1.82E-282	1.544519167	0.719	0.484	4.24E-278
Macrophage	ANXA5	0	1.534272936	0.662	0.329	0
Macrophage	SDCBP	0	1.533240329	0.75	0.451	0
Macrophage	SMIM25	0	1.529765931	0.343	0.009	0
Macrophage	MPEG1	0	1.528901263	0.482	0.015	0
Macrophage	RNF130	0	1.507717747	0.593	0.242	0
Macrophage	C5AR1	0	1.506156766	0.388	0.004	0
Macrophage	MT2A	1.96E-67	1.502999637	0.456	0.329	4.57E-63
Macrophage	NFKBIZ	1.61E-272	1.500227103	0.505	0.248	3.75E-268
Macrophage	SLC11A1	0	1.494513866	0.338	0.015	0
Macrophage	MAFB	0	1.467494079	0.372	0.03	0
Macrophage	CFD	0	1.456589869	0.588	0.155	0
Macrophage	KCTD12	0	1.446868693	0.485	0.082	0
Macrophage	NCF2	0	1.438300731	0.444	0.006	0
Macrophage	VSIG4	0	1.435109581	0.273	0.002	0
Macrophage	GCA	0	1.416554424	0.472	0.193	0
Macrophage	SGK1	0	1.415305774	0.328	0.063	0
Macrophage	CARD16	0	1.400658543	0.612	0.283	0
Macrophage	SERPINA1	0	1.381536651	0.616	0.148	0
Macrophage	ZFP36	0	1.370931896	0.805	0.643	0
Macrophage	PTPRE	0	1.345355347	0.501	0.145	0

Macrophage	CTSZ	5.39E-250	1.340242775	0.53	0.302	1.25E-245
Macrophage	CD86	0	1.325882123	0.438	0.019	0
Macrophage	PHACTR1	0	1.304384012	0.374	0.057	0
Macrophage	FCGR3A	0	1.298146808	0.277	0.026	0
Macrophage	CD302	0	1.297060517	0.424	0.052	0
Macrophage	MCL1	0	1.295668303	0.778	0.565	0
Macrophage	RAB31	0	1.293169351	0.421	0.017	0
Macrophage	IER3	9.82E-117	1.292541562	0.485	0.324	2.28E-112
Macrophage	GADD45B	3.97E-211	1.28758433	0.432	0.2	9.23E-207
Macrophage	CD55	4.17E-215	1.283190217	0.518	0.288	9.70E-211
Macrophage	PYCARD	0	1.279664637	0.645	0.389	0
Macrophage	VSIR	0	1.27601527	0.516	0.163	0
Macrophage	HCK	0	1.268360873	0.4	0.005	0
Macrophage	TREM1	0	1.261069163	0.265	0.002	0
Macrophage	MS4A4A	0	1.249436006	0.286	0.002	0
Macrophage	BLVRB	7.54E-213	1.236807574	0.531	0.327	1.75E-208
Macrophage	CLEC4A	0	1.225512684	0.351	0.006	0
Macrophage	HLA-DMB	0	1.221936914	0.513	0.122	0
Macrophage	CSF1R	0	1.220661796	0.361	0.002	0
Macrophage	APLP2	0	1.21563737	0.648	0.381	0
Macrophage	LIPA	0	1.209976581	0.402	0.132	0
Macrophage	STXBP2	2.16E-204	1.207565712	0.51	0.302	5.03E-200
Macrophage	VMP1	1.42E-154	1.206696373	0.652	0.483	3.31E-150
Macrophage	GABARAP	5.76E-285	1.199217593	0.645	0.414	1.34E-280
Macrophage	GLIPR1	0	1.198650769	0.618	0.192	0
Macrophage	CCDC88A	0	1.19692489	0.435	0.035	0
Macrophage	GMFG	0	1.195856384	0.738	0.324	0
Macrophage	CLEC4E	0	1.19371551	0.279	0.001	0
Macrophage	TSPO	5.52E-278	1.191848976	0.727	0.5	1.28E-273
Macrophage	CYBA	0	1.171349867	0.926	0.887	0
Macrophage	PPT1	0	1.161371828	0.509	0.206	0
Macrophage	NUP214	0	1.151656287	0.384	0.1	0
Macrophage	LITAF	1.22E-58	1.151381075	0.495	0.433	2.84E-54
Macrophage	KLF2	5.35E-280	1.150526097	0.463	0.182	1.25E-275
Macrophage	AP2S1	1.68E-285	1.142988142	0.665	0.456	3.91E-281
Macrophage	SLC7A7	0	1.139568896	0.374	0.019	0
Macrophage	ATP6V1B2	0	1.139425713	0.416	0.136	0
Macrophage	CD93	0	1.138367643	0.272	0.001	0
Macrophage	PLIN2	1.17E-32	1.132176651	0.27	0.201	2.72E-28
Macrophage	SERPINB9	1.35E-144	1.131036296	0.303	0.128	3.13E-140
Macrophage	TPP1	0	1.130388459	0.498	0.169	0
Macrophage	HBEGF	5.14E-158	1.128320004	0.277	0.113	1.20E-153
Macrophage	TNFAIP2	0	1.124358242	0.315	0.022	0

Macrophage	CD163	0	1.118927478	0.297	0.001	0
Macrophage	JAML	0	1.11861309	0.522	0.153	0
Macrophage	ATP6V0B	9.77E-260	1.115903413	0.68	0.473	2.27E-255
Macrophage	DUSP6	2.88E-191	1.111332858	0.375	0.172	6.70E-187
Macrophage	IFNGR1	1.97E-276	1.106577324	0.493	0.232	4.58E-272
Macrophage	AHNAK	5.60E-176	1.106512334	0.474	0.276	1.30E-171
Macrophage	EGR1	1.06E-49	1.102219689	0.426	0.342	2.46E-45
Macrophage	FGR	0	1.098487107	0.362	0.025	0
Macrophage	ARRB2	0	1.095651694	0.473	0.125	0
Macrophage	CALHM6	0	1.093211718	0.374	0.044	0
Macrophage	TKT	3.21E-159	1.089318498	0.59	0.413	7.46E-155
Macrophage	OAZ1	0	1.085984082	0.918	0.889	0
Macrophage	EVI2B	0	1.08537493	0.62	0.264	0
Macrophage	DPYSL2	0	1.085074248	0.408	0.099	0
Macrophage	PILRA	0	1.084551354	0.342	0.003	0
Macrophage	FCGR2B	0	1.083091459	0.288	0.037	0
Macrophage	ITM2B	0	1.080323441	0.868	0.794	0
Macrophage	RNF149	3.36E-174	1.078642624	0.467	0.264	7.82E-170
Macrophage	CD83	0	1.069938661	0.371	0.07	0
Macrophage	OGFRL1	0	1.065727203	0.34	0.034	0
Macrophage	GPR183	8.21E-258	1.058753737	0.409	0.143	1.91E-253
Macrophage	HIF1A	4.56E-90	1.057657546	0.373	0.235	1.06E-85
Macrophage	LILRB3	0	1.051790194	0.324	0.004	0
Macrophage	KYNU	0	1.047560618	0.327	0.02	0
Macrophage	SLC25A37	1.22E-31	1.046522742	0.261	0.194	2.84E-27
Macrophage	ADA2	0	1.041195741	0.378	0.038	0
Macrophage	NINJ1	1.07E-210	1.03922583	0.339	0.137	2.49E-206
Macrophage	TGFBI	0	1.035656625	0.508	0.209	0
Macrophage	ITGAM	0	1.030926942	0.297	0.007	0
Macrophage	GSTO1	1.17E-175	1.030467144	0.583	0.403	2.71E-171
Macrophage	TNFRSF1B	0	1.030255548	0.463	0.137	0
Macrophage	MFSD1	3.24E-294	1.024078493	0.414	0.159	7.54E-290
Macrophage	HMOX1	0	1.02243717	0.274	0.067	0
Macrophage	HSP90AA1	8.75E-14	1.020917864	0.798	0.808	2.03E-09
Macrophage	IVNS1ABP	1.34E-29	1.020515173	0.351	0.292	3.12E-25
Macrophage	LILRA5	0	1.017508549	0.275	0.001	0
Macrophage	GNAI2	0	1.016354022	0.526	0.221	0
Macrophage	SNX10	0	1.015521522	0.39	0.106	0
Macrophage	IER5	1.54E-80	1.015102979	0.306	0.178	3.59E-76
Macrophage	NCF1	4.47E-248	1.011945261	0.468	0.184	1.04E-243
Macrophage	MXD1	1.30E-85	1.003954869	0.281	0.153	3.03E-81
Macrophage	BNIP3L	6.65E-184	1.002579467	0.465	0.255	1.55E-179
Macrophage	QKI	0	0.999927148	0.36	0.034	0

Macrophage	RGS18	0	0.998320847	0.305	0.007	0
Macrophage	ARPC5	1.34E-268	0.993024315	0.761	0.621	3.11E-264
Macrophage	LCP1	0	0.990395751	0.701	0.273	0
Macrophage	CSF2RA	0	0.989107394	0.306	0.002	0
Macrophage	LILRB2	0	0.987148013	0.305	0.001	0
Macrophage	CRIP1	1.10E-85	0.984407897	0.593	0.47	2.56E-81
Macrophage	YBX3	1.37E-135	0.98353618	0.517	0.343	3.17E-131
Macrophage	VAMP8	2.84E-184	0.974734584	0.702	0.618	6.60E-180
Macrophage	LGALS2	1.35E-202	0.970132921	0.359	0.142	3.13E-198
Macrophage	MEF2C	0	0.969733358	0.543	0.157	0
Macrophage	TLR2	0	0.961433588	0.274	0.002	0
Macrophage	C9orf72	0	0.958786635	0.32	0.07	0
Macrophage	LPAR6	0	0.957493335	0.348	0.093	0
Macrophage	RBPJ	1.69E-167	0.957360365	0.504	0.304	3.92E-163
Macrophage	LYN	7.01E-250	0.954947316	0.428	0.18	1.63E-245
Macrophage	CASP1	2.06E-186	0.954862503	0.487	0.268	4.78E-182
Macrophage	MARCKS	6.83E-74	0.953592315	0.498	0.399	1.59E-69
Macrophage	CTSH	1.39E-216	0.951070256	0.484	0.259	3.23E-212
Macrophage	GLRX	3.86E-152	0.949662759	0.482	0.304	8.97E-148
Macrophage	RHOA	6.45E-268	0.946476584	0.792	0.677	1.50E-263
Macrophage	CPPED1	0	0.945218547	0.314	0.081	0
Macrophage	CHMP1B	2.20E-86	0.94405804	0.48	0.361	5.11E-82
Macrophage	CMTM6	1.90E-158	0.943227648	0.603	0.428	4.42E-154
Macrophage	CD4	0	0.943139669	0.328	0.017	0
Macrophage	SCPEP1	0	0.942672199	0.38	0.09	0
Macrophage	ATP2B1	4.26E-119	0.928827614	0.481	0.32	9.92E-115
Macrophage	PLXDC2	0	0.927184481	0.312	0.053	0
Macrophage	STX11	0	0.925147636	0.318	0.025	0
Macrophage	FKBP1A	4.42E-221	0.920334281	0.725	0.583	1.03E-216
Macrophage	LAPTM5	0	0.91685558	0.766	0.37	0
Macrophage	FOSL2	1.87E-76	0.915989839	0.324	0.205	4.36E-72
Macrophage	ARPC1B	2.37E-202	0.90636053	0.741	0.649	5.52E-198
Macrophage	ASGR1	0	0.904193336	0.272	0.012	0
Macrophage	NAGK	7.27E-248	0.903243863	0.404	0.167	1.69E-243
Macrophage	LRRC25	0	0.90315544	0.301	0.007	0
Macrophage	LY86	0	0.902716015	0.421	0.066	0
Macrophage	LCP2	1.41E-238	0.900556418	0.35	0.11	3.29E-234
Macrophage	RTN4	3.64E-191	0.899110964	0.679	0.5	8.46E-187
Macrophage	WSB1	6.24E-152	0.898306752	0.62	0.444	1.45E-147
Macrophage	CALM2	2.01E-213	0.895898426	0.839	0.818	4.66E-209
Macrophage	RNF13	2.18E-154	0.892421758	0.434	0.246	5.08E-150
Macrophage	ATP1B3	1.88E-88	0.891809145	0.533	0.411	4.37E-84
Macrophage	ACTB	0	0.88951374	0.983	0.978	0

Macrophage	TMEM167A	2.33E-112	0.882621377	0.518	0.38	5.42E-108
Macrophage	SAMSN1	7.78E-178	0.874479384	0.462	0.203	1.81E-173
Macrophage	RHOB	5.94E-60	0.871200943	0.448	0.352	1.38E-55
Macrophage	TALDO1	1.92E-104	0.870546662	0.574	0.447	4.46E-100
Macrophage	IFNGR2	1.98E-134	0.870147705	0.429	0.263	4.59E-130
Macrophage	RHOG	1.65E-205	0.867037872	0.5	0.26	3.84E-201
Macrophage	CCR1	0	0.866658427	0.262	0.015	0
Macrophage	TBXAS1	0	0.861786407	0.328	0.082	0
Macrophage	LYST	1.19E-267	0.858672974	0.324	0.098	2.76E-263
Macrophage	H2AFY	1.18E-170	0.857659107	0.636	0.464	2.74E-166
Macrophage	TIMP2	0	0.855371016	0.305	0.034	0
Macrophage	TNFAIP3	2.31E-82	0.852973318	0.342	0.189	5.37E-78
Macrophage	TLR4	0	0.852912088	0.269	0.058	0
Macrophage	ATP2B1-AS1	4.00E-290	0.851173001	0.308	0.085	9.29E-286
Macrophage	RAB32	3.85E-154	0.848937552	0.356	0.181	8.96E-150
Macrophage	FLNA	3.73E-307	0.84744519	0.411	0.134	8.68E-303
Macrophage	PLSCR1	1.13E-72	0.847016053	0.402	0.294	2.62E-68
Macrophage	NR4A2	2.17E-133	0.844408315	0.506	0.3	5.06E-129
Macrophage	HNMT	1.66E-111	0.84032471	0.381	0.238	3.86E-107
Macrophage	SLC43A2	5.62E-266	0.840300374	0.282	0.082	1.31E-261
Macrophage	ALOX5AP	1.84E-89	0.840187897	0.47	0.281	4.27E-85
Macrophage	THEMIS2	0	0.83895207	0.312	0.03	0
Macrophage	ATP6AP2	5.22E-135	0.829309238	0.555	0.398	1.21E-130
Macrophage	IER2	2.49E-58	0.82390131	0.696	0.665	5.79E-54
Macrophage	SH3BGRL	4.30E-140	0.820322977	0.603	0.448	1.00E-135
Macrophage	HACD4	0	0.820204509	0.345	0.052	0
Macrophage	LTA4H	2.57E-81	0.815913284	0.383	0.265	5.98E-77
Macrophage	TMSB10	0	0.815617437	0.966	0.962	0
Macrophage	C3AR1	0	0.813884958	0.265	0.02	0
Macrophage	CNPY3	8.90E-153	0.812345812	0.478	0.283	2.07E-148
Macrophage	DNAJB1	7.69E-15	0.810416738	0.26	0.385	1.79E-10
Macrophage	CAPZA2	1.23E-123	0.808425691	0.584	0.45	2.85E-119
Macrophage	ZFP36L1	5.16E-68	0.806696389	0.667	0.581	1.20E-63
Macrophage	YWHAH	2.04E-30	0.803084283	0.459	0.428	4.75E-26
Macrophage	UPP1	3.40E-141	0.799240446	0.292	0.125	7.91E-137
Macrophage	TUBA1A	1.24E-137	0.785677592	0.442	0.232	2.88E-133
Macrophage	KLF4	9.04E-86	0.781111287	0.418	0.279	2.10E-81
Macrophage	SLC31A2	0	0.780869345	0.261	0.025	0
Macrophage	PLD3	2.83E-51	0.780432049	0.31	0.221	6.59E-47
Macrophage	RNASET2	3.09E-110	0.778618752	0.638	0.546	7.18E-106
Macrophage	SLC16A3	7.10E-108	0.778185452	0.376	0.216	1.65E-103
Macrophage	SELL	1.76E-272	0.775559582	0.26	0.06	4.10E-268
Macrophage	JUNB	5.10E-174	0.772266298	0.845	0.785	1.19E-169

Macrophage	GNS	3.22E-152	0.771145412	0.269	0.11	7.48E-148
Macrophage	CREG1	2.01E-81	0.769827759	0.322	0.205	4.68E-77
Macrophage	GLIPR2	3.09E-230	0.769687816	0.366	0.131	7.18E-226
Macrophage	H3F3B	3.00E-199	0.768513843	0.943	0.94	6.99E-195
Macrophage	IFITM2	5.76E-11	0.763701953	0.494	0.455	1.34E-06
Macrophage	SULT1A1	7.02E-122	0.762346605	0.294	0.143	1.63E-117
Macrophage	NOP10	2.22E-94	0.762248198	0.657	0.566	5.17E-90
Macrophage	BACH1	1.63E-158	0.761929195	0.262	0.099	3.79E-154
Macrophage	PECAM1	0	0.760961605	0.279	0.039	0
Macrophage	RNH1	1.16E-100	0.760860326	0.526	0.414	2.70E-96
Macrophage	IQGAP1	4.13E-99	0.760583304	0.548	0.418	9.61E-95
Macrophage	GPCPD1	3.20E-78	0.75311313	0.266	0.145	7.44E-74
Macrophage	KDM6B	8.77E-94	0.752194074	0.291	0.153	2.04E-89
Macrophage	SH3BGRL3	1.37E-180	0.747975806	0.826	0.785	3.19E-176
Macrophage	VAMP5	5.35E-188	0.745778574	0.338	0.129	1.24E-183
Macrophage	ARPC3	3.70E-191	0.742303683	0.796	0.767	8.61E-187
Macrophage	PNRC1	1.29E-76	0.741432411	0.704	0.647	3.00E-72
Macrophage	WARS	1.12E-121	0.741050627	0.261	0.116	2.61E-117
Macrophage	CTSC	1.03E-66	0.740533659	0.479	0.356	2.40E-62
Macrophage	UBE2D1	1.37E-92	0.739485137	0.357	0.221	3.19E-88
Macrophage	EVI2A	1.48E-252	0.739369147	0.468	0.173	3.45E-248
Macrophage	LAMTOR4	2.89E-113	0.739303771	0.699	0.583	6.73E-109
Macrophage	LAMP2	1.14E-71	0.734655243	0.363	0.252	2.65E-67
Macrophage	EFHD2	3.04E-84	0.734378515	0.453	0.332	7.06E-80
Macrophage	ATP6V1F	6.68E-89	0.733895103	0.563	0.468	1.55E-84
Macrophage	LRP1	9.44E-145	0.733618417	0.267	0.113	2.20E-140
Macrophage	RILPL2	3.89E-220	0.731686843	0.322	0.11	9.04E-216
Macrophage	MSN	2.39E-246	0.728915582	0.499	0.196	5.56E-242
Macrophage	DPYD	0	0.726661319	0.257	0.03	0
Macrophage	SNX2	2.74E-118	0.723819453	0.454	0.287	6.37E-114
Macrophage	CORO1C	3.94E-107	0.719662011	0.286	0.147	9.17E-103
Macrophage	ATG3	1.32E-76	0.717958828	0.427	0.317	3.06E-72
Macrophage	BRI3	2.60E-96	0.716664806	0.492	0.353	6.06E-92
Macrophage	C20orf27	1.61E-121	0.716228394	0.331	0.174	3.74E-117
Macrophage	ZFAND5	1.32E-84	0.715857613	0.443	0.318	3.07E-80
Macrophage	TXNIP	7.77E-73	0.704048609	0.703	0.667	1.81E-68
Macrophage	FYB1	3.91E-265	0.702792823	0.596	0.238	9.09E-261
Macrophage	PRCP	3.09E-173	0.700858367	0.324	0.138	7.20E-169
Macrophage	FBXL5	7.15E-104	0.700851185	0.331	0.178	1.66E-99
Macrophage	PICALM	9.52E-100	0.694590618	0.324	0.18	2.21E-95
Macrophage	MGAT1	3.05E-89	0.690238786	0.399	0.259	7.10E-85
Macrophage	CXCL16	3.54E-76	0.687086268	0.281	0.166	8.24E-72
Macrophage	LRRFIP1	2.54E-97	0.684261637	0.619	0.505	5.91E-93

Macrophage	MAT2A	3.62E-47	0.683970747	0.34	0.255	8.42E-43
Macrophage	PFDN5	1.19E-206	0.68289217	0.89	0.894	2.76E-202
Macrophage	CDKN1A	3.39E-51	0.679043454	0.294	0.196	7.89E-47
Macrophage	KLF10	2.65E-39	0.676624225	0.255	0.176	6.16E-35
Macrophage	ATP5MPL	1.96E-112	0.676365689	0.797	0.708	4.55E-108
Macrophage	CD300A	0	0.674908606	0.254	0.021	0
Macrophage	PRNP	5.90E-109	0.671675626	0.268	0.117	1.37E-104
Macrophage	FXYD5	4.19E-140	0.671663933	0.641	0.44	9.74E-136
Macrophage	POMP	2.25E-85	0.67136088	0.718	0.62	5.24E-81
Macrophage	IL13RA1	2.75E-71	0.667460779	0.3	0.188	6.41E-67
Macrophage	HEXB	1.71E-54	0.663945298	0.414	0.332	3.98E-50
Macrophage	ANXA2	3.11E-104	0.663345077	0.696	0.532	7.22E-100
Macrophage	IFITM3	3.25E-67	0.662047932	0.502	0.35	7.57E-63
Macrophage	ATP6V0D1	1.02E-75	0.661268328	0.422	0.306	2.37E-71
Macrophage	NFE2L2	5.40E-44	0.658149529	0.444	0.375	1.26E-39
Macrophage	BST2	2.33E-138	0.655162629	0.518	0.288	5.42E-134
Macrophage	BLVRA	3.45E-212	0.654832807	0.256	0.08	8.02E-208
Macrophage	ACTR2	4.18E-105	0.652182763	0.664	0.565	9.72E-101
Macrophage	AGTRAP	5.26E-82	0.646136955	0.286	0.159	1.22E-77
Macrophage	LGALS9	3.11E-72	0.645484273	0.375	0.262	7.23E-68
Macrophage	LAP3	8.01E-54	0.644988483	0.326	0.233	1.86E-49
Macrophage	DOK2	2.98E-257	0.643079082	0.4	0.129	6.93E-253
Macrophage	PSMB3	1.22E-89	0.642689911	0.643	0.544	2.83E-85
Macrophage	SERPINB1	3.69E-44	0.641674886	0.555	0.465	8.59E-40
Macrophage	PLBD1	7.53E-36	0.641073413	0.322	0.251	1.75E-31
Macrophage	PCBP1	6.53E-96	0.639450752	0.683	0.615	1.52E-91
Macrophage	CAPG	3.39E-21	0.634646802	0.477	0.453	7.88E-17
Macrophage	ATOX1	5.09E-30	0.630137745	0.394	0.351	1.18E-25
Macrophage	PLEKHO1	0	0.629816645	0.32	0.079	0
Macrophage	HNRNPU	1.35E-52	0.625200615	0.703	0.659	3.14E-48
Macrophage	AC004687.1	4.19E-190	0.62503746	0.352	0.127	9.75E-186
Macrophage	CRTAP	1.23E-61	0.623900791	0.289	0.186	2.85E-57
Macrophage	MYO1F	3.44E-226	0.623655028	0.454	0.17	7.99E-222
Macrophage	CCNL1	2.04E-56	0.619755118	0.636	0.577	4.74E-52
Macrophage	LIMS1	1.18E-39	0.615356609	0.401	0.323	2.75E-35
Macrophage	JUND	9.06E-40	0.612031831	0.583	0.52	2.11E-35
Macrophage	SNX3	1.61E-100	0.610303382	0.667	0.603	3.74E-96
Macrophage	RGS10	3.97E-106	0.609278358	0.421	0.239	9.23E-102
Macrophage	LSP1	7.73E-132	0.607757632	0.622	0.366	1.80E-127
Macrophage	TNFSF10	6.44E-13	0.607146881	0.311	0.292	1.50E-08
Macrophage	NCOA4	5.09E-54	0.604653304	0.365	0.271	1.18E-49
Macrophage	SEC11A	2.75E-69	0.604534181	0.566	0.487	6.39E-65
Macrophage	SMAP2	1.58E-145	0.604518524	0.319	0.136	3.68E-141

Macrophage	PGK1	3.68E-73	0.604200536	0.654	0.571	8.56E-69
Macrophage	MYADM	5.48E-65	0.60204589	0.365	0.244	1.27E-60
Macrophage	CDC42	2.02E-90	0.600901351	0.744	0.689	4.70E-86
Macrophage	MOB1A	3.93E-63	0.591667949	0.525	0.433	9.14E-59
Macrophage	GPX4	1.63E-77	0.588798963	0.686	0.616	3.79E-73
Macrophage	NABP1	5.21E-89	0.586779705	0.299	0.149	1.21E-84
Macrophage	SMCO4	2.21E-38	0.584813265	0.323	0.249	5.14E-34
Macrophage	NCF4	4.98E-240	0.58428456	0.255	0.067	1.16E-235
Macrophage	ZYX	4.22E-73	0.578470926	0.333	0.206	9.81E-69
Macrophage	RAP2B	8.98E-110	0.574103949	0.271	0.125	2.09E-105
Macrophage	PRKCB	1.29E-205	0.571814765	0.312	0.099	2.99E-201
Macrophage	TAGLN2	2.39E-38	0.571595078	0.595	0.554	5.56E-34
Macrophage	NR4A1	2.22E-27	0.570544865	0.369	0.309	5.15E-23
Macrophage	FAM49B	4.52E-80	0.570141621	0.554	0.42	1.05E-75
Macrophage	CAP1	8.13E-78	0.569373675	0.664	0.604	1.89E-73
Macrophage	BID	1.65E-37	0.564951923	0.335	0.262	3.83E-33
Macrophage	CREM	5.80E-29	0.563326936	0.265	0.186	1.35E-24
Macrophage	LACTB	1.05E-69	0.554089198	0.255	0.146	2.45E-65
Macrophage	CFLAR	4.34E-20	0.553506371	0.397	0.356	1.01E-15
Macrophage	FAM45A	1.78E-17	0.550926608	0.263	0.222	4.13E-13
Macrophage	CLEC2B	5.48E-135	0.550534584	0.484	0.229	1.27E-130
Macrophage	SPG21	1.36E-45	0.548179946	0.411	0.334	3.17E-41
Macrophage	SKAP2	6.56E-50	0.545035095	0.403	0.308	1.53E-45
Macrophage	RNF181	1.01E-50	0.543945103	0.483	0.411	2.35E-46
Macrophage	NUMB	3.80E-25	0.542703261	0.285	0.225	8.83E-21
Macrophage	PGD	4.81E-34	0.541665699	0.261	0.194	1.12E-29
Macrophage	PGLS	3.54E-48	0.538995782	0.466	0.385	8.24E-44
Macrophage	USP15	2.26E-43	0.537947838	0.36	0.26	5.25E-39
Macrophage	OAZ2	6.57E-49	0.537457676	0.315	0.219	1.53E-44
Macrophage	AOAH	2.95E-195	0.536585625	0.28	0.089	6.87E-191
Macrophage	STAT1	2.13E-27	0.534680579	0.302	0.233	4.95E-23
Macrophage	VPS29	2.12E-40	0.533009543	0.485	0.427	4.94E-36
Macrophage	NCKAP1L	1.57E-235	0.531705373	0.319	0.093	3.66E-231
Macrophage	GNAQ	1.09E-24	0.531521292	0.294	0.243	2.53E-20
Macrophage	IFI6	7.25E-17	0.531171033	0.279	0.221	1.69E-12
Macrophage	IL10RA	2.05E-195	0.530896926	0.327	0.11	4.77E-191
Macrophage	HCLS1	1.82E-152	0.530398639	0.463	0.209	4.22E-148
Macrophage	RBM47	1.21E-18	0.528039979	0.333	0.299	2.82E-14
Macrophage	PPP1R15A	2.21E-24	0.52563679	0.572	0.551	5.14E-20
Macrophage	SUPT4H1	1.02E-49	0.525375878	0.416	0.321	2.38E-45
Macrophage	TMBIM4	6.28E-54	0.524136308	0.643	0.606	1.46E-49
Macrophage	TPM4	1.76E-19	0.523809124	0.485	0.47	4.10E-15
Macrophage	TGFB1	2.18E-168	0.523607561	0.344	0.133	5.07E-164

Macrophage	GRB2	3.08E-66	0.521068091	0.46	0.343	7.16E-62
Macrophage	CSTB	3.12E-56	0.517853105	0.666	0.558	7.25E-52
Macrophage	PLEKHB2	5.80E-29	0.517761473	0.342	0.28	1.35E-24
Macrophage	ATP6V0E1	7.81E-65	0.517368497	0.726	0.705	1.82E-60
Macrophage	TCIRG1	3.27E-64	0.516846886	0.318	0.197	7.59E-60
Macrophage	CTSA	2.70E-26	0.513358552	0.369	0.322	6.28E-22
Macrophage	ATP5F1E	3.35E-163	0.512732938	0.905	0.912	7.79E-159
Macrophage	GIMAP4	5.17E-179	0.511100124	0.407	0.157	1.20E-174
Macrophage	ATF3	5.92E-28	0.51042821	0.333	0.26	1.38E-23
Macrophage	EIF1	3.38E-170	0.505031579	0.946	0.958	7.86E-166
Macrophage	RTN3	1.22E-18	0.50434873	0.369	0.338	2.84E-14
Macrophage	RBMS1	3.59E-170	0.500677458	0.31	0.107	8.34E-166
Macrophage	HSBP1	3.59E-34	0.497741066	0.518	0.455	8.34E-30
Macrophage	NDUFB1	2.76E-52	0.497082793	0.671	0.601	6.42E-48
Macrophage	VPS35	3.28E-25	0.496579133	0.416	0.375	7.62E-21
Macrophage	PFN1	5.99E-172	0.495101522	0.917	0.904	1.39E-167
Macrophage	GPSM3	1.61E-105	0.492775221	0.527	0.3	3.75E-101
Macrophage	TNFRSF1A	1.86E-31	0.483629125	0.325	0.251	4.33E-27
Macrophage	BNIP2	2.46E-88	0.483254698	0.347	0.192	5.72E-84
Macrophage	ARPC2	1.27E-111	0.4822268	0.823	0.833	2.95E-107
Macrophage	SERF2	1.58E-171	0.48128391	0.931	0.929	3.67E-167
Macrophage	LAMTOR1	1.03E-32	0.481145411	0.506	0.469	2.39E-28
Macrophage	ZNF106	1.06E-26	0.480023532	0.31	0.25	2.46E-22
Macrophage	POLE4	1.87E-21	0.478130784	0.369	0.324	4.36E-17
Macrophage	LAMTOR2	1.50E-28	0.477871996	0.449	0.411	3.49E-24
Macrophage	XIST	4.31E-38	0.475300491	0.294	0.191	1.00E-33
Macrophage	RAB7A	3.64E-38	0.475180013	0.487	0.436	8.47E-34
Macrophage	ETS2	1.63E-08	0.467074292	0.35	0.319	0.000379456
Macrophage	WASHC4	1.93E-53	0.466515941	0.285	0.178	4.48E-49
Macrophage	CASP4	1.30E-42	0.466230392	0.387	0.285	3.01E-38
Macrophage	UCP2	2.36E-67	0.465754763	0.418	0.276	5.48E-63
Macrophage	CDC42SE1	1.68E-26	0.464535391	0.327	0.248	3.91E-22
Macrophage	RBX1	3.19E-38	0.463385914	0.579	0.537	7.41E-34
Macrophage	SYK	3.36E-61	0.46102027	0.262	0.156	7.81E-57
Macrophage	CD53	9.34E-132	0.460790127	0.6	0.32	2.17E-127
Macrophage	TUBA1B	3.53E-17	0.460703845	0.565	0.571	8.21E-13
Macrophage	S100A6	7.01E-180	0.460554574	0.944	0.815	1.63E-175
Macrophage	MCUB	4.40E-113	0.458377599	0.329	0.157	1.02E-108
Macrophage	PAK1	7.38E-20	0.456320083	0.27	0.229	1.72E-15
Macrophage	TMSB4X	1.03E-137	0.454383861	0.991	0.994	2.41E-133
Macrophage	LAPTM4A	1.05E-31	0.452203883	0.556	0.503	2.45E-27
Macrophage	MTPN	1.70E-36	0.450523765	0.403	0.332	3.96E-32
Macrophage	PKM	9.87E-44	0.449817016	0.623	0.556	2.30E-39

Macrophage	H3F3A	9.61E-99	0.44843008	0.927	0.909	2.23E-94
Macrophage	PET100	2.62E-22	0.446422436	0.543	0.524	6.10E-18
Macrophage	FAM96A	4.17E-26	0.44532199	0.39	0.34	9.70E-22
Macrophage	WAS	5.23E-166	0.445316948	0.336	0.122	1.22E-161
Macrophage	JMJD1C	3.95E-19	0.442860549	0.409	0.365	9.20E-15
Macrophage	UNC93B1	1.05E-27	0.441686334	0.253	0.191	2.44E-23
Macrophage	SFT2D1	2.37E-27	0.440968558	0.504	0.471	5.50E-23
Macrophage	PARVG	4.75E-223	0.436560484	0.293	0.083	1.10E-218
Macrophage	TMEM176B	0.003997861	0.436392017	0.303	0.315	1
Macrophage	MAP3K2	1.21E-12	0.434791523	0.25	0.215	2.82E-08
Macrophage	EIF4EBP1	5.95E-24	0.430165428	0.316	0.26	1.38E-19
Macrophage	FMNL1	5.91E-134	0.429072333	0.367	0.155	1.37E-129
Macrophage	CAT	7.31E-25	0.428705858	0.333	0.277	1.70E-20
Macrophage	DAZAP2	1.22E-30	0.426902074	0.606	0.572	2.84E-26
Macrophage	MYL6	3.79E-90	0.426664824	0.909	0.893	8.80E-86
Macrophage	PRELID1	8.68E-19	0.425049604	0.586	0.581	2.02E-14
Macrophage	MAP2K1	2.52E-43	0.423048804	0.298	0.203	5.86E-39
Macrophage	ENY2	1.80E-23	0.420268894	0.51	0.467	4.18E-19
Macrophage	ANP32A	9.90E-14	0.420009973	0.406	0.379	2.30E-09
Macrophage	TWF2	1.24E-47	0.419978022	0.339	0.235	2.88E-43
Macrophage	GNAI3	2.48E-21	0.41981132	0.35	0.298	5.78E-17
Macrophage	FERMT3	1.27E-88	0.41976508	0.315	0.16	2.95E-84
Macrophage	COMT	1.17E-27	0.419641955	0.384	0.324	2.72E-23
Macrophage	RAP1A	1.05E-33	0.418222299	0.53	0.481	2.43E-29
Macrophage	SERP1	4.41E-35	0.417641917	0.687	0.676	1.03E-30
Macrophage	STMP1	3.06E-19	0.41664047	0.426	0.391	7.11E-15
Macrophage	LEPROT	8.61E-18	0.416576171	0.352	0.318	2.00E-13
Macrophage	DHRS4L2	7.70E-18	0.41391967	0.273	0.232	1.79E-13
Macrophage	EIF4A3	6.72E-11	0.413080114	0.291	0.266	1.56E-06
Macrophage	NAAA	1.16E-47	0.412539819	0.264	0.164	2.69E-43
Macrophage	C15orf48	3.67E-12	0.412228655	0.333	0.414	8.53E-08
Macrophage	C4orf48	1.05E-24	0.408774696	0.496	0.425	2.44E-20
Macrophage	METTL9	0.000140121	0.408043047	0.402	0.427	1
Macrophage	DNAJA1	3.64E-06	0.407751228	0.443	0.456	0.08463381
Macrophage	M6PR	9.05E-36	0.402974654	0.412	0.333	2.10E-31
Macrophage	PPP1CB	1.55E-12	0.40213656	0.484	0.48	3.61E-08
Macrophage	PRDX1	2.58E-08	0.401331835	0.585	0.568	0.000600555
Macrophage	HSD17B11	9.77E-11	0.40084282	0.458	0.448	2.27E-06
Macrophage	RAB10	1.56E-13	0.400379395	0.394	0.38	3.63E-09
Macrophage	PSMA4	1.50E-22	0.400126211	0.565	0.54	3.49E-18
Macrophage	ATP6AP1	2.57E-08	0.396490223	0.269	0.256	0.000597764
Macrophage	B2M	5.16E-126	0.396081164	0.998	0.996	1.20E-121
Macrophage	REL	3.09E-28	0.395080008	0.406	0.328	7.20E-24

Macrophage	PTEN	1.09E-11	0.394966326	0.288	0.258	2.52E-07
Macrophage	RAC1	5.36E-59	0.393202893	0.743	0.62	1.25E-54
Macrophage	SSR1	3.36E-18	0.391266911	0.436	0.415	7.81E-14
Macrophage	PQLC3	7.61E-51	0.387871856	0.281	0.174	1.77E-46
Macrophage	CLTC	2.49E-05	0.387274461	0.31	0.316	0.578702871
Macrophage	CHD1	1.39E-10	0.382386847	0.31	0.282	3.24E-06
Macrophage	ARPC4	1.39E-21	0.382180644	0.504	0.478	3.23E-17
Macrophage	IFI16	3.37E-53	0.382085318	0.534	0.386	7.83E-49
Macrophage	TPM3	5.94E-38	0.381007775	0.663	0.64	1.38E-33
Macrophage	XRN2	7.84E-20	0.380564945	0.402	0.36	1.82E-15
Macrophage	OTULINL	1.44E-21	0.379007454	0.289	0.236	3.35E-17
Macrophage	ENO1	1.69E-34	0.377152996	0.676	0.64	3.93E-30
Macrophage	FABP5	0.000152574	0.375899035	0.257	0.311	1
Macrophage	UBE2B	0.000335564	0.372607486	0.417	0.437	1
Macrophage	STX10	3.40E-16	0.372225109	0.29	0.249	7.91E-12
Macrophage	C4orf3	6.42E-22	0.367023591	0.625	0.605	1.49E-17
Macrophage	GDI2	2.33E-22	0.366899349	0.541	0.522	5.42E-18
Macrophage	ADGRE5	6.37E-21	0.365586677	0.28	0.22	1.48E-16
Macrophage	ADPGK	8.27E-45	0.365410851	0.288	0.188	1.92E-40
Macrophage	DDX21	9.15E-09	0.362770273	0.415	0.405	0.000212856
Macrophage	IQGAP2	6.17E-18	0.362340655	0.256	0.203	1.43E-13
Macrophage	POU2F2	7.35E-121	0.362066632	0.288	0.115	1.71E-116
Macrophage	BAZ1A	4.57E-13	0.358541134	0.383	0.355	1.06E-08
Macrophage	ACAP2	1.03E-16	0.358493817	0.307	0.258	2.40E-12
Macrophage	CYTOR	8.94E-09	0.356376195	0.251	0.208	0.000207855
Macrophage	ATP6V1G1	1.57E-29	0.35530653	0.695	0.696	3.65E-25
Macrophage	SHKBP1	2.64E-27	0.350660879	0.297	0.222	6.14E-23
Macrophage	TLE4	3.15E-16	0.350367943	0.286	0.231	7.31E-12
Macrophage	PRKAR1A	6.21E-20	0.343430395	0.427	0.379	1.44E-15
Macrophage	TLN1	1.07E-22	0.342676452	0.374	0.31	2.50E-18
Macrophage	CAPZA1	2.54E-18	0.341491855	0.538	0.529	5.90E-14
Macrophage	UBE2R2	0.00018317	0.340457962	0.255	0.256	1
Macrophage	VMA21	8.97E-20	0.338395814	0.277	0.224	2.09E-15
Macrophage	VAPA	1.18E-12	0.338209132	0.54	0.537	2.75E-08
Macrophage	FOSB	3.62E-15	0.336413788	0.59	0.578	8.42E-11
Macrophage	NMI	8.63E-11	0.336237804	0.285	0.254	2.01E-06
Macrophage	OSBPL8	4.99E-12	0.334691374	0.311	0.27	1.16E-07
Macrophage	SAT2	7.84E-08	0.332230071	0.317	0.308	0.00182324
Macrophage	SNAP23	1.51E-12	0.329814223	0.322	0.285	3.52E-08
Macrophage	DNAJB6	1.86E-05	0.329251162	0.431	0.445	0.43352382
Macrophage	EIF3M	7.50E-09	0.327860039	0.504	0.509	0.000174347
Macrophage	LY6E	5.18E-19	0.327793561	0.363	0.279	1.21E-14
Macrophage	TRIM38	4.10E-16	0.327053364	0.322	0.269	9.52E-12

Macrophage	CKLF	2.73E-32	0.326612764	0.588	0.526	6.36E-28
Macrophage	TMEM50A	5.97E-13	0.326420495	0.525	0.525	1.39E-08
Macrophage	B4GALT1	1.51E-06	0.32456078	0.25	0.228	0.0352306
Macrophage	PRDX3	6.61E-12	0.322892601	0.407	0.387	1.54E-07
Macrophage	HERPUD1	2.17E-25	0.319678309	0.548	0.516	5.05E-21
Macrophage	RGS19	3.55E-99	0.319029813	0.3	0.133	8.25E-95
Macrophage	BTF3L4	7.71E-13	0.318757735	0.33	0.299	1.79E-08
Macrophage	IRF8	4.86E-40	0.317192286	0.269	0.168	1.13E-35
Macrophage	YBX1	8.74E-31	0.316910725	0.806	0.828	2.03E-26
Macrophage	MTHFD2	4.37E-13	0.313629701	0.263	0.225	1.02E-08
Macrophage	SSR3	5.81E-10	0.313548101	0.441	0.432	1.35E-05
Macrophage	LSM6	8.85E-06	0.31192431	0.387	0.388	0.205811499
Macrophage	ERP44	6.78E-12	0.309512193	0.397	0.386	1.58E-07
Macrophage	NDFIP1	1.63E-14	0.309070852	0.415	0.397	3.80E-10
Macrophage	DRAM2	5.51E-22	0.308823925	0.369	0.31	1.28E-17
Macrophage	UBE2D3	2.42E-18	0.306497323	0.664	0.69	5.63E-14
Macrophage	SERINC1	3.19E-14	0.304406353	0.302	0.26	7.41E-10
Macrophage	HMGN3	1.87E-12	0.303451716	0.436	0.418	4.36E-08
Macrophage	RAB5C	1.23E-09	0.302671233	0.403	0.394	2.85E-05
Macrophage	MIS18BP1	1.04E-20	0.300244922	0.294	0.232	2.42E-16
Macrophage	HNRNPH2	5.99E-08	0.299700402	0.33	0.311	0.001391817
Macrophage	ARHGDIB	1.05E-59	0.296562016	0.711	0.526	2.44E-55
Macrophage	ARL6IP5	1.22E-29	0.293875398	0.589	0.545	2.83E-25
Macrophage	NAP1L1	1.55E-16	0.293555748	0.671	0.68	3.60E-12
Macrophage	LAT2	4.34E-98	0.292967879	0.278	0.117	1.01E-93
Macrophage	C6orf62	3.15E-06	0.292659556	0.356	0.352	0.073360603
Macrophage	SPCS3	2.96E-08	0.291476099	0.38	0.373	0.000689361
Macrophage	HADHB	4.44E-05	0.288759217	0.323	0.327	1
Macrophage	STX7	5.52E-23	0.28691182	0.312	0.247	1.28E-18
Macrophage	CAPNS1	0.000319681	0.28615603	0.343	0.351	1
Macrophage	IFI27L2	4.32E-07	0.286015723	0.398	0.393	0.01005118
Macrophage	CD48	4.18E-58	0.285117529	0.475	0.289	9.73E-54
Macrophage	MAPK1	1.73E-07	0.282650249	0.279	0.256	0.004029906
Macrophage	UBA52	1.39E-69	0.27954697	0.91	0.938	3.23E-65
Macrophage	GNG5	1.00E-12	0.278925482	0.571	0.538	2.33E-08
Macrophage	PSMB10	1.20E-10	0.274925627	0.402	0.383	2.78E-06
Macrophage	VASP	1.49E-05	0.273529301	0.454	0.472	0.346814612
Macrophage	MGST2	7.48E-09	0.271580773	0.376	0.342	0.000173908
Macrophage	ARF3	0.001828889	0.270789412	0.266	0.268	1
Macrophage	DBNL	3.91E-12	0.270445313	0.371	0.341	9.10E-08
Macrophage	PAK2	1.78E-06	0.268085927	0.373	0.369	0.041479339
Macrophage	PTPN6	1.27E-49	0.264430628	0.431	0.284	2.95E-45
Macrophage	PSME2	0.000413027	0.26300793	0.558	0.586	1

Macrophage	ATP5MC2	1.35E-23	0.259074564	0.771	0.772	3.14E-19
Macrophage	NAA38	0.000251353	0.258746645	0.434	0.441	1
Macrophage	SUB1	6.48E-13	0.256806289	0.749	0.752	1.51E-08
Macrophage	RAP1B	1.80E-09	0.254958614	0.525	0.527	4.19E-05
Plasma B cell	JCHAIN	0	7.473543855	0.995	0.399	0
Plasma B cell	IGHA1	0	7.319467102	0.988	0.556	0
Plasma B cell	IGKC	0	7.285196483	0.977	0.644	0
Plasma B cell	IGLC2	0	7.120815811	0.863	0.405	0
Plasma B cell	IGLC3	0	6.982630197	0.577	0.163	0
Plasma B cell	IGHA2	0	6.527281838	0.933	0.303	0
Plasma B cell	IGHG1	4.41E-243	6.196116401	0.445	0.128	1.03E-238
Plasma B cell	IGHG3	3.19E-188	6.069593359	0.433	0.14	7.43E-184
Plasma B cell	IGLL5	0	4.976925955	0.326	0.013	0
Plasma B cell	IGHG4	6.89E-285	4.820715028	0.357	0.079	1.60E-280
Plasma B cell	IGHM	2.61E-96	4.4884215	0.412	0.169	6.08E-92
Plasma B cell	MZB1	0	3.794051213	0.985	0.069	0
Plasma B cell	SSR4	0	2.684168702	0.979	0.694	0
Plasma B cell	DERL3	0	2.636425295	0.912	0.048	0
Plasma B cell	HERPUD1	0	2.27859706	0.973	0.499	0
Plasma B cell	XBP1	0	2.232939357	0.947	0.453	0
Plasma B cell	FKBP11	0	2.080414795	0.93	0.284	0
Plasma B cell	TNFRSF17	0	2.066378867	0.86	0.015	0
Plasma B cell	SEC11C	0	1.563500848	0.879	0.345	0
Plasma B cell	UBE2J1	0	1.486018872	0.884	0.304	0
Plasma B cell	HSP90B1	0	1.449041202	0.958	0.598	0
Plasma B cell	SPINK2	0	1.379801899	0.414	0.04	0
Plasma B cell	PIM2	0	1.374736333	0.77	0.133	0
Plasma B cell	DNAJB9	0	1.354600443	0.84	0.21	0
Plasma B cell	JUN	1.29E-146	1.298072729	0.884	0.716	3.00E-142
Plasma B cell	FKBP2	0	1.284198821	0.929	0.505	0
Plasma B cell	PRDX4	0	1.24595756	0.844	0.329	0
Plasma B cell	SSR3	0	1.160465096	0.894	0.413	0
Plasma B cell	SPCS2	0	1.141193394	0.925	0.602	0
Plasma B cell	BTG2	7.83E-238	1.061282907	0.899	0.527	1.82E-233
Plasma B cell	TENT5C	0	1.048818548	0.767	0.112	0
Plasma B cell	Z93241.1	0	1.014106434	0.424	0.041	0
Plasma B cell	CD79A	0	1.006890387	0.865	0.137	0
Plasma B cell	FCRL5	0	1.001762658	0.643	0.013	0
Plasma B cell	CITED2	5.25E-220	0.994846371	0.62	0.249	1.22E-215
Plasma B cell	SPCS3	0	0.962410905	0.854	0.352	0
Plasma B cell	PABPC4	0	0.923981345	0.723	0.275	0
Plasma B cell	MANF	0	0.92203638	0.849	0.371	0
Plasma B cell	JSRP1	0	0.881447315	0.414	0.014	0

Plasma B cell	CD27	0	0.881022488	0.682	0.096	0
Plasma B cell	ANKRD28	0	0.874466717	0.643	0.163	0
Plasma B cell	PDIA4	5.99E-252	0.854469418	0.825	0.397	1.39E-247
Plasma B cell	SPCS1	4.90E-235	0.838579796	0.912	0.613	1.14E-230
Plasma B cell	TXNDC11	0	0.836281255	0.666	0.135	0
Plasma B cell	TXNDC5	0	0.834029907	0.57	0.038	0
Plasma B cell	SELENOS	2.20E-283	0.828893158	0.882	0.434	5.12E-279
Plasma B cell	WDR74	3.35E-205	0.828419847	0.533	0.206	7.80E-201
Plasma B cell	SDF2L1	1.07E-298	0.825828539	0.848	0.388	2.49E-294
Plasma B cell	PDK1	0	0.821781911	0.61	0.078	0
Plasma B cell	CRELD2	0	0.821447557	0.752	0.246	0
Plasma B cell	TXNDC15	0	0.791067185	0.697	0.144	0
Plasma B cell	DUSP5	0	0.782255469	0.523	0.053	0
Plasma B cell	EAF2	0	0.748182	0.584	0.069	0
Plasma B cell	SELENOK	9.86E-212	0.720355445	0.863	0.491	2.29E-207
Plasma B cell	PLPP5	0	0.704360307	0.717	0.223	0
Plasma B cell	ERLEC1	0	0.694569229	0.76	0.304	0
Plasma B cell	POU2AF1	0	0.687735702	0.621	0.048	0
Plasma B cell	SEC61B	6.62E-175	0.682216799	0.92	0.595	1.54E-170
Plasma B cell	GNG7	0	0.681077352	0.629	0.047	0
Plasma B cell	CCR10	0	0.677207985	0.452	0.003	0
Plasma B cell	TRAM1	3.94E-242	0.662341504	0.851	0.423	9.16E-238
Plasma B cell	CCPG1	0	0.655818528	0.7	0.227	0
Plasma B cell	SEL1L	0	0.638916055	0.628	0.175	0
Plasma B cell	HIST1H2BG	7.47E-227	0.623563919	0.315	0.074	1.74E-222
Plasma B cell	DNAJC3	3.22E-231	0.60936938	0.774	0.351	7.49E-227
Plasma B cell	CD38	0	0.606373239	0.582	0.054	0
Plasma B cell	LY96	0	0.596347624	0.605	0.062	0
Plasma B cell	MYDGF	1.28E-155	0.593716721	0.823	0.437	2.97E-151
Plasma B cell	MEF2C	0	0.579734023	0.787	0.158	0
Plasma B cell	SELENOM	0	0.57718995	0.701	0.174	0
Plasma B cell	ISG20	1.14E-296	0.573178211	0.813	0.283	2.64E-292
Plasma B cell	RASGRP3	0	0.572814871	0.416	0.036	0
Plasma B cell	ELL2	0	0.558539115	0.489	0.068	0
Plasma B cell	LMAN1	8.54E-227	0.553690617	0.717	0.307	1.99E-222
Plasma B cell	LINC02362	0	0.535850768	0.378	0.003	0
Plasma B cell	MEI1	0	0.528171744	0.521	0.029	0
Plasma B cell	SPATS2	0	0.523968916	0.572	0.173	0
Plasma B cell	TP53INP1	0	0.511899594	0.393	0.07	0
Plasma B cell	SDC1	1.53E-179	0.510495672	0.564	0.236	3.56E-175
Plasma B cell	HSPA5	1.04E-104	0.509917672	0.856	0.512	2.41E-100
Plasma B cell	CHPF	0	0.5090296	0.537	0.139	0
Plasma B cell	SPAG4	0	0.496155607	0.409	0.021	0

Plasma B cell	PDIA6	1.21E-109	0.485259035	0.875	0.531	2.82E-105
Plasma B cell	PTCH2	0	0.481936766	0.281	0.036	0
Plasma B cell	IRF4	0	0.481398643	0.315	0.02	0
Plasma B cell	FOSB	3.19E-60	0.47744729	0.819	0.568	7.41E-56
Plasma B cell	PRDM1	0	0.474649033	0.538	0.105	0
Plasma B cell	RRBP1	1.74E-115	0.466547992	0.767	0.401	4.05E-111
Plasma B cell	MANEA	0	0.465133926	0.435	0.059	0
Plasma B cell	KLF2	5.01E-164	0.450127082	0.519	0.189	1.17E-159
Plasma B cell	CREB3L2	0	0.439667974	0.505	0.126	0
Plasma B cell	AP001160.1	0	0.439442712	0.254	0.033	0
Plasma B cell	HSPA1B	3.13E-60	0.438910962	0.445	0.258	7.28E-56
Plasma B cell	SUB1	2.40E-68	0.428967896	0.925	0.744	5.59E-64
Plasma B cell	KRTCAP2	1.43E-100	0.426783078	0.856	0.533	3.34E-96
Plasma B cell	TSC22D3	2.13E-123	0.426176242	0.769	0.381	4.96E-119
Plasma B cell	SLAMF7	0	0.41713104	0.493	0.056	0
Plasma B cell	CXorf21	0	0.411766183	0.307	0.035	0
Plasma B cell	ZBP1	0	0.4035926	0.405	0.044	0
Plasma B cell	RAB30	0	0.403289676	0.376	0.052	0
Plasma B cell	AC007952.4	0	0.40153388	0.419	0.088	0
Plasma B cell	HM13	2.50E-162	0.39877807	0.696	0.321	5.82E-158
Plasma B cell	ERN1	1.19E-211	0.389400217	0.323	0.08	2.76E-207
Plasma B cell	GMPPB	3.71E-237	0.386249173	0.489	0.157	8.63E-233
Plasma B cell	FNDC3A	2.70E-200	0.379147965	0.508	0.181	6.29E-196
Plasma B cell	SEC62	8.79E-100	0.374889767	0.91	0.605	2.04E-95
Plasma B cell	ICAM2	0	0.370159875	0.553	0.075	0
Plasma B cell	DNAJC1	2.17E-182	0.367354343	0.655	0.28	5.04E-178
Plasma B cell	RGCC	0	0.365866268	0.424	0.092	0
Plasma B cell	TCF4	0	0.365822809	0.481	0.072	0
Plasma B cell	NUCB2	1.77E-184	0.364659786	0.689	0.291	4.13E-180
Plasma B cell	SIL1	2.94E-204	0.355837977	0.529	0.194	6.84E-200
Plasma B cell	ARMCX3	6.37E-213	0.353786365	0.6	0.221	1.48E-208
Plasma B cell	DNAJB11	1.58E-170	0.353625532	0.648	0.282	3.67E-166
Plasma B cell	TRIB1	3.47E-103	0.351820038	0.457	0.21	8.06E-99
Plasma B cell	SEL1L3	1.90E-189	0.350386851	0.644	0.266	4.42E-185
Plasma B cell	CYTOR	2.58E-176	0.34995167	0.553	0.196	6.00E-172
Plasma B cell	SERP1	3.79E-62	0.346704905	0.907	0.666	8.81E-58
Plasma B cell	HSPA13	0	0.34201917	0.391	0.063	0
Plasma B cell	TIFA	1.92E-305	0.341314808	0.4	0.088	4.47E-301
Plasma B cell	TNFRSF13B	0	0.340178383	0.369	0.038	0
Plasma B cell	CPEB4	2.27E-180	0.331463545	0.393	0.125	5.29E-176
Plasma B cell	ALG5	1.87E-132	0.33103587	0.664	0.318	4.35E-128
Plasma B cell	FBXW7	2.86E-241	0.33010389	0.457	0.133	6.66E-237
Plasma B cell	SRPRB	2.72E-190	0.327375892	0.566	0.219	6.32E-186

Plasma B cell	RPN1	1.92E-95	0.317608809	0.679	0.361	4.47E-91
Plasma B cell	SEC14L1	0	0.316903739	0.476	0.118	0
Plasma B cell	LMAN2	5.27E-81	0.312875487	0.802	0.472	1.23E-76
Plasma B cell	TMEM258	8.80E-39	0.312030461	0.888	0.626	2.05E-34
Plasma B cell	GLCCI1	2.27E-287	0.308339084	0.354	0.073	5.29E-283
Plasma B cell	CPNE5	0	0.305450981	0.298	0.024	0
Plasma B cell	IFNAR2	5.76E-306	0.304974631	0.548	0.149	1.34E-301
Plasma B cell	PPIB	3.43E-43	0.304820223	0.915	0.624	7.99E-39
Plasma B cell	HIST1H1C	1.43E-95	0.294186588	0.416	0.186	3.33E-91
Plasma B cell	UAP1	1.62E-120	0.289063662	0.514	0.237	3.76E-116
Plasma B cell	ARSA	6.05E-233	0.286412451	0.408	0.113	1.41E-228
Plasma B cell	UFM1	6.58E-105	0.283535027	0.727	0.381	1.53E-100
Plasma B cell	SRM	4.85E-134	0.281300281	0.618	0.28	1.13E-129
Plasma B cell	CASP10	6.31E-166	0.27710651	0.327	0.096	1.47E-161
Plasma B cell	Z93930.2	1.36E-208	0.270774786	0.261	0.056	3.16E-204
Plasma B cell	LMO4	1.27E-138	0.26891186	0.5	0.202	2.94E-134
Plasma B cell	AC103591.3	1.34E-63	0.268374197	0.279	0.12	3.12E-59
Plasma B cell	KCNN3	0	0.266600286	0.25	0.003	0
Plasma B cell	ERGIC2	1.03E-152	0.26642262	0.646	0.289	2.39E-148
Plasma B cell	SLC1A4	3.70E-235	0.259009784	0.285	0.06	8.60E-231
Plasma B cell	RPN2	4.65E-77	0.258871045	0.743	0.414	1.08E-72
Plasma B cell	PNOC	0	0.258545502	0.29	0.015	0
Plasma B cell	RABAC1	2.32E-65	0.258069582	0.823	0.523	5.39E-61
T cell	CCL5	0	5.041748355	0.819	0.103	0
T cell	GZMA	0	4.342383927	0.684	0.081	0
T cell	CD3D	0	3.71972741	0.861	0.052	0
T cell	CD7	0	3.554402511	0.776	0.052	0
T cell	KLRB1	0	3.503273191	0.561	0.032	0
T cell	TRAC	0	3.485619706	0.86	0.1	0
T cell	NKG7	0	3.464165235	0.645	0.048	0
T cell	TRBC1	0	2.984550118	0.546	0.03	0
T cell	CD2	0	2.973389853	0.723	0.028	0
T cell	CD3E	0	2.927893669	0.789	0.027	0
T cell	TRDC	0	2.917574759	0.297	0.018	0
T cell	CCL4	0	2.846475104	0.406	0.066	0
T cell	IL32	0	2.814827117	0.906	0.468	0
T cell	TRBC2	0	2.790521858	0.708	0.087	0
T cell	TRGC2	0	2.756938767	0.507	0.021	0
T cell	KLRD1	0	2.72262308	0.575	0.022	0
T cell	RGS1	0	2.656824515	0.68	0.193	0
T cell	CD3G	0	2.593625318	0.652	0.021	0
T cell	HCST	0	2.570416917	0.8	0.146	0
T cell	EVL	0	2.563847254	0.827	0.158	0

T cell	LINC01871	0	2.452783233	0.557	0.021	0
T cell	HOPX	0	2.414629626	0.554	0.047	0
T cell	LCK	0	2.237734718	0.653	0.065	0
T cell	CST7	0	2.202241883	0.516	0.027	0
T cell	PTPRC	0	2.083776021	0.882	0.265	0
T cell	ID2	0	2.072636737	0.732	0.505	0
T cell	ITGA1	0	2.058736881	0.462	0.124	0
T cell	IL7R	0	2.053469812	0.338	0.017	0
T cell	CD8A	0	2.007374538	0.397	0.015	0
T cell	CD160	0	2.002709642	0.397	0.014	0
T cell	CCL4L2	0	2.001599216	0.281	0.044	0
T cell	GZMB	0	1.996152562	0.313	0.035	0
T cell	CD96	0	1.994817871	0.505	0.024	0
T cell	CD247	0	1.98521872	0.468	0.019	0
T cell	CD69	0	1.983584933	0.777	0.215	0
T cell	FYB1	0	1.910080998	0.587	0.09	0
T cell	PRF1	0	1.854204234	0.383	0.012	0
T cell	ACAP1	0	1.836781112	0.657	0.148	0
T cell	CLEC2D	0	1.805536514	0.595	0.122	0
T cell	GIMAP7	0	1.762066399	0.468	0.041	0
T cell	ITM2A	0	1.748233443	0.476	0.079	0
T cell	CD8B	0	1.740897304	0.332	0.014	0
T cell	GZMM	0	1.715951686	0.423	0.015	0
T cell	RUNX3	0	1.694605827	0.446	0.052	0
T cell	CD52	0	1.694139398	0.898	0.346	0
T cell	CORO1A	0	1.672624551	0.837	0.332	0
T cell	CTSW	0	1.638781179	0.335	0.016	0
T cell	STK17B	0	1.628979484	0.659	0.313	0
T cell	RARRES3	0	1.625987038	0.59	0.258	0
T cell	CKLF	0	1.598069418	0.639	0.472	0
T cell	FYN	0	1.573755975	0.431	0.074	0
T cell	TIGIT	0	1.570657033	0.345	0.014	0
T cell	GPR171	0	1.5664728	0.362	0.009	0
T cell	RORA	0	1.546892788	0.346	0.048	0
T cell	ARHGDIB	0	1.531810711	0.794	0.402	0
T cell	PRKCH	0	1.515905584	0.388	0.028	0
T cell	ALOX5AP	0	1.511410841	0.582	0.139	0
T cell	RAC2	0	1.489557551	0.637	0.269	0
T cell	TMIGD2	0	1.486092266	0.338	0.014	0
T cell	PTPN7	0	1.463634559	0.441	0.091	0
T cell	IL2RG	0	1.449515738	0.552	0.263	0
T cell	XCL2	0	1.431503032	0.251	0.007	0
T cell	CLEC2B	0	1.425968926	0.491	0.116	0

T cell	IL2RB	0	1.42434754	0.318	0.013	0
T cell	GIMAP4	0	1.423633537	0.393	0.056	0
T cell	TBC1D10C	0	1.411595187	0.472	0.1	0
T cell	LAT	0	1.402447212	0.339	0.028	0
T cell	CYTIP	0	1.388092376	0.544	0.189	0
T cell	TMSB4X	0	1.386808061	0.993	0.994	0
T cell	PTPN22	0	1.386003409	0.366	0.056	0
T cell	STK17A	0	1.377207603	0.647	0.316	0
T cell	B2M	0	1.377039047	0.997	0.996	0
T cell	LSP1	0	1.352858926	0.632	0.25	0
T cell	TRAF3IP3	0	1.349050992	0.469	0.126	0
T cell	ABI3	0	1.339045202	0.397	0.076	0
T cell	PPP2R5C	0	1.337462456	0.517	0.437	0
T cell	DUSP2	0	1.320029006	0.331	0.171	0
T cell	SRSF7	0	1.315302031	0.725	0.658	0
T cell	BTG1	0	1.305273581	0.911	0.813	0
T cell	PYHIN1	0	1.267398894	0.302	0.025	0
T cell	TRG-AS1	0	1.266275535	0.28	0.016	0
T cell	LEPROTL1	0	1.258088614	0.446	0.322	0
T cell	SYTL3	0	1.256166178	0.295	0.023	0
T cell	SARAF	0	1.255023218	0.779	0.72	0
T cell	AAK1	0	1.247135626	0.38	0.212	0
T cell	JAML	0	1.244502806	0.372	0.075	0
T cell	SLFN5	0	1.228813667	0.311	0.078	0
T cell	KIR2DL4	0	1.227182777	0.25	0.009	0
T cell	TNFAIP3	0	1.222379368	0.33	0.13	0
T cell	DDX24	0	1.194975031	0.57	0.491	0
T cell	IKZF1	0	1.192595847	0.444	0.136	0
T cell	IKZF3	0	1.180465461	0.37	0.093	0
T cell	SPN	0	1.17579082	0.307	0.041	0
T cell	STK4	0	1.159655868	0.556	0.406	0
T cell	DDX5	0	1.155750386	0.915	0.864	0
T cell	IFITM2	0	1.150753264	0.536	0.415	0
T cell	SKAP1	0	1.144225228	0.3	0.043	0
T cell	PIP4K2A	0	1.141899766	0.358	0.124	0
T cell	GYPC	0	1.140941566	0.431	0.138	0
T cell	LCP1	0	1.134419283	0.533	0.179	0
T cell	BIN2	0	1.133981338	0.332	0.096	0
T cell	CD48	0	1.132183432	0.502	0.194	0
T cell	MYO1F	0	1.124151414	0.35	0.104	0
T cell	SAMSN1	0	1.121506277	0.408	0.121	0
T cell	HLA-E	0	1.105671784	0.887	0.851	0
T cell	WIPF1	0	1.102341519	0.403	0.134	0

T cell	FNBP1	0	1.097407655	0.453	0.252	0
T cell	GMFG	0	1.096677912	0.578	0.232	0
T cell	OSTF1	0	1.090497123	0.509	0.441	0
T cell	GPR65	0	1.087957622	0.332	0.08	0
T cell	ARL4C	0	1.079982775	0.299	0.104	0
T cell	APOBEC3G	0	1.078164116	0.279	0.065	0
T cell	TSC22D3	0	1.077985992	0.514	0.334	0
T cell	CHST12	0	1.072784439	0.321	0.181	0
T cell	PLEKHF1	0	1.06180543	0.257	0.029	0
T cell	ARHGAP30	0	1.059435082	0.363	0.107	0
T cell	MATK	0	1.057017232	0.257	0.016	0
T cell	MYL12A	0	1.056390068	0.871	0.858	0
T cell	CELF2	1.91E-287	1.051267244	0.443	0.359	4.44E-283
T cell	SUN2	0	1.039189739	0.345	0.196	0
T cell	LDLRAD4	8.23E-250	1.03646747	0.286	0.169	1.91E-245
T cell	PAXX	3.29E-229	1.031665175	0.445	0.413	7.66E-225
T cell	RBL2	6.11E-231	1.030766524	0.325	0.223	1.42E-226
T cell	DENND2D	1.91E-268	1.028791938	0.32	0.206	4.44E-264
T cell	RNF213	1.67E-242	1.020786483	0.444	0.385	3.88E-238
T cell	PPP1R18	0	1.019148853	0.368	0.13	0
T cell	STOM	0	1.016988291	0.285	0.083	0
T cell	APBB1IP	0	1.01333801	0.331	0.085	0
T cell	MBNL1	2.31E-288	1.0117131	0.509	0.469	5.36E-284
T cell	ACTB	0	1.008519774	0.98	0.977	0
T cell	PTPN4	0	1.008202112	0.299	0.159	0
T cell	DOK2	0	1.000101086	0.302	0.065	0
T cell	ARHGAP9	0	0.991950444	0.293	0.068	0
T cell	LYAR	5.02E-143	0.989628754	0.27	0.198	1.17E-138
T cell	CLDND1	3.15E-220	0.988377393	0.309	0.208	7.32E-216
T cell	AKNA	0	0.982641724	0.309	0.142	0
T cell	PIM1	0	0.963887612	0.264	0.123	0
T cell	AC245297.3	2.66E-244	0.95905734	0.345	0.237	6.19E-240
T cell	HLA-B	0	0.953512524	0.979	0.968	0
T cell	NR3C1	0	0.950412391	0.311	0.118	0
T cell	ARHGEF1	1.32E-229	0.948148528	0.343	0.242	3.07E-225
T cell	IRF1	1.67E-173	0.947787622	0.418	0.369	3.89E-169
T cell	BUB3	1.22E-203	0.945906006	0.443	0.417	2.84E-199
T cell	VAMP2	0	0.945902424	0.543	0.508	0
T cell	ETS1	0	0.945843923	0.281	0.105	0
T cell	S100A4	0	0.925436948	0.704	0.367	0
T cell	RASAL3	0	0.924781966	0.26	0.049	0
T cell	IDS	1.44E-211	0.919549465	0.385	0.294	3.34E-207
T cell	PIK3R1	2.58E-85	0.91658227	0.302	0.272	6.01E-81

T cell	C1orf56	1.52E-117	0.91338101	0.284	0.212	3.53E-113
T cell	SYNE2	2.57E-96	0.912485949	0.376	0.377	5.97E-92
T cell	ANKRD12	1.08E-222	0.909493111	0.519	0.515	2.52E-218
T cell	ITGB7	0	0.907985584	0.265	0.079	0
T cell	SRGN	0	0.907495432	0.843	0.268	0
T cell	CDC42SE2	6.42E-134	0.901008415	0.439	0.453	1.49E-129
T cell	CTSC	4.53E-204	0.900759084	0.408	0.342	1.05E-199
T cell	VIM	0	0.900392969	0.664	0.256	0
T cell	RASSF5	0	0.898700569	0.28	0.08	0
T cell	MBP	1.46E-143	0.885593206	0.347	0.292	3.40E-139
T cell	EVI2A	0	0.881543665	0.319	0.127	0
T cell	LBH	0	0.878088032	0.295	0.084	0
T cell	FMNL1	0	0.876285464	0.297	0.101	0
T cell	RGS10	6.53E-268	0.874930547	0.333	0.208	1.52E-263
T cell	HCLS1	0	0.873825161	0.369	0.15	0
T cell	CCDC85B	2.63E-159	0.872677458	0.422	0.403	6.11E-155
T cell	SRSF5	0	0.871501365	0.725	0.75	0
T cell	HLA-C	0	0.870467802	0.941	0.923	0
T cell	KIAA1551	1.89E-176	0.869351746	0.323	0.233	4.40E-172
T cell	ARPC2	0	0.863040374	0.831	0.832	0
T cell	LCP2	0	0.862704592	0.265	0.053	0
T cell	ITGAE	2.09E-76	0.861896955	0.287	0.257	4.85E-72
T cell	ARL6IP5	2.60E-246	0.860892521	0.55	0.547	6.05E-242
T cell	ICAM3	6.65E-225	0.856501932	0.399	0.306	1.55E-220
T cell	LY6E	0	0.855095782	0.388	0.229	0
T cell	JAK1	1.79E-156	0.852683387	0.483	0.514	4.16E-152
T cell	ANKRD44	0	0.851937716	0.332	0.145	0
T cell	PTPN6	1.52E-231	0.850316022	0.369	0.254	3.54E-227
T cell	RHOF	0	0.847818119	0.299	0.151	0
T cell	C9orf78	2.22E-82	0.845122731	0.387	0.412	5.17E-78
T cell	ARHGAP45	7.61E-206	0.842334821	0.323	0.221	1.77E-201
T cell	ARF6	6.28E-264	0.841220718	0.569	0.602	1.46E-259
T cell	EMB	0	0.841172157	0.285	0.135	0
T cell	AC114760.2	0	0.839255088	0.264	0.1	0
T cell	PFN1	0	0.838123325	0.909	0.902	0
T cell	PDCD4	2.92E-115	0.837829385	0.457	0.496	6.78E-111
T cell	PSMB9	1.09E-256	0.837296515	0.536	0.511	2.54E-252
T cell	ODF2L	1.20E-42	0.833739789	0.295	0.297	2.79E-38
T cell	PIK3IP1	7.30E-162	0.830989436	0.257	0.165	1.70E-157
T cell	APOBEC3C	2.03E-196	0.830943487	0.256	0.148	4.73E-192
T cell	CYTOR	8.32E-200	0.827321554	0.279	0.175	1.93E-195
T cell	N4BP2L2	0	0.823905309	0.69	0.726	0
T cell	MSN	0	0.822276104	0.338	0.153	0

T cell	TERF2IP	8.09E-133	0.821545874	0.415	0.399	1.88E-128
T cell	CCDC69	0	0.819902098	0.263	0.097	0
T cell	DOCK8	0	0.815369468	0.324	0.155	0
T cell	ZFP36L2	0	0.814754248	0.729	0.736	0
T cell	ELF1	1.68E-119	0.812169568	0.469	0.514	3.92E-115
T cell	CCL3L1	0	0.810331801	0.252	0.042	0
T cell	FUS	8.57E-262	0.810176588	0.605	0.664	1.99E-257
T cell	ABHD17A	7.43E-88	0.809844536	0.261	0.215	1.73E-83
T cell	LIMD2	0	0.802819733	0.465	0.232	0
T cell	DDX3X	6.52E-88	0.79859376	0.484	0.553	1.52E-83
T cell	NR4A2	2.21E-127	0.796205051	0.362	0.289	5.14E-123
T cell	RHOH	0	0.79579666	0.344	0.121	0
T cell	PHYKPL	3.04E-31	0.79364175	0.295	0.309	7.06E-27
T cell	POLR2J3.1	5.06E-79	0.783973797	0.441	0.487	1.18E-74
T cell	HLA-A	0	0.783583228	0.959	0.924	0
T cell	FGFR1OP2	2.49E-85	0.781461049	0.315	0.284	5.78E-81
T cell	PRPF38B	3.54E-75	0.773655128	0.445	0.513	8.24E-71
T cell	ANXA6	0	0.772163189	0.314	0.16	0
T cell	RAB27A	2.60E-171	0.770179081	0.258	0.164	6.05E-167
T cell	BTN3A2	4.99E-69	0.76917748	0.288	0.259	1.16E-64
T cell	ACTR3	1.06E-255	0.765607528	0.596	0.647	2.46E-251
T cell	SYNRG	1.61E-97	0.762123645	0.267	0.212	3.74E-93
T cell	ANXA1	3.94E-226	0.761866237	0.323	0.187	9.15E-222
T cell	DRAP1	3.42E-117	0.753352765	0.485	0.557	7.95E-113
T cell	SSBP4	1.77E-59	0.751754252	0.27	0.247	4.12E-55
T cell	CD53	0	0.747947155	0.515	0.244	0
T cell	IL16	0	0.74792613	0.263	0.095	0
T cell	GPSM3	0	0.746690569	0.427	0.256	0
T cell	CCND3	9.65E-88	0.745603596	0.288	0.241	2.24E-83
T cell	CRIP1	5.62E-297	0.744548623	0.547	0.442	1.31E-292
T cell	CMC1	3.83E-237	0.743426054	0.142	0.362	8.91E-233
T cell	MALAT1	0	0.739421349	0.999	0.995	0
T cell	ARHGAP15	0	0.738867379	0.266	0.096	0
T cell	CD99	1.28E-173	0.736490971	0.54	0.582	2.97E-169
T cell	EID1	1.61E-95	0.729996219	0.462	0.517	3.74E-91
T cell	RSRP1	7.45E-101	0.725347648	0.516	0.592	1.73E-96
T cell	G3BP2	1.46E-06	0.720863308	0.338	0.436	0.034043644
T cell	PCM1	1.81E-23	0.718410406	0.34	0.392	4.22E-19
T cell	CITED2	2.30E-19	0.718088252	0.26	0.266	5.36E-15
T cell	PNRC1	1.08E-296	0.713851954	0.644	0.655	2.50E-292
T cell	IFITM1	1.01E-140	0.713520005	0.43	0.381	2.36E-136
T cell	SNRPN	5.00E-136	0.713103246	0.277	0.199	1.16E-131
T cell	SON	5.06E-196	0.711599988	0.598	0.697	1.18E-191

T cell	NAP1L4	5.74E-15	0.710757246	0.305	0.359	1.34E-10
T cell	CALM1	0	0.70673182	0.742	0.748	0
T cell	MACF1	9.84E-28	0.706356566	0.276	0.283	2.29E-23
T cell	AKAP13	3.61E-38	0.703339174	0.38	0.43	8.41E-34
T cell	RAP1B	1.51E-76	0.696299262	0.475	0.555	3.51E-72
T cell	PPP1R2	1.37E-11	0.693713774	0.353	0.44	3.18E-07
T cell	PCSK7	3.27E-24	0.687902706	0.396	0.482	7.60E-20
T cell	PSMB8-AS1	4.34E-165	0.687458881	0.272	0.175	1.01E-160
T cell	JUNB	7.55E-153	0.68555155	0.749	0.811	1.75E-148
T cell	CDC42SE1	3.13E-15	0.677809441	0.248	0.257	7.28E-11
T cell	IFI16	4.74E-185	0.677683907	0.453	0.366	1.10E-180
T cell	PSMA3-AS1	9.78E-15	0.676352619	0.338	0.4	2.27E-10
T cell	SH3BGRL3	0	0.673181709	0.778	0.794	0
T cell	FAM49B	1.02E-89	0.669784664	0.426	0.431	2.36E-85
T cell	RAP1A	8.91E-39	0.667053287	0.425	0.516	2.07E-34
T cell	ROCK1	3.81E-28	0.666539622	0.354	0.401	8.87E-24
T cell	COMMD7	1.01E-10	0.66534246	0.283	0.331	2.34E-06
T cell	CLK1	3.72E-26	0.659163567	0.347	0.384	8.65E-22
T cell	LIMS1	2.87E-11	0.657564648	0.297	0.346	6.68E-07
T cell	TSC22D4	2.51E-18	0.647812096	0.277	0.301	5.83E-14
T cell	TTC14	2.10E-18	0.64776031	0.277	0.293	4.89E-14
T cell	NKTR	8.14E-11	0.640022256	0.374	0.471	1.89E-06
T cell	ARID4B	1.58E-17	0.639215954	0.368	0.444	3.66E-13
T cell	LAPTM5	0	0.635893007	0.619	0.278	0
T cell	ABRACL	6.24E-70	0.6351463	0.475	0.56	1.45E-65
T cell	ITM2B	0	0.632440866	0.8	0.799	0
T cell	HMOX2	1.53E-06	0.631319711	0.264	0.315	0.035559951
T cell	TNRC6B	1.77E-19	0.63080489	0.382	0.454	4.12E-15
T cell	DHRS7	2.06E-20	0.630053771	0.379	0.469	4.79E-16
T cell	CCND2	3.62E-09	0.627466226	0.289	0.335	8.43E-05
T cell	TRAPPC1	8.89E-72	0.624360935	0.481	0.578	2.07E-67
T cell	PRRC2C	1.68E-46	0.617060068	0.477	0.602	3.90E-42
T cell	POLR3GL	8.18E-12	0.615993837	0.268	0.299	1.90E-07
T cell	RPS27	0	0.615038331	0.982	0.983	0
T cell	HNRNPDL	1.07E-132	0.614932478	0.574	0.671	2.49E-128
T cell	ORMDL1	0.000579283	0.606926775	0.304	0.377	1
T cell	PNISR	1.70E-162	0.603249597	0.625	0.73	3.95E-158
T cell	ARPC1B	1.42E-215	0.602462827	0.633	0.668	3.29E-211
T cell	ARGLU1	3.23E-74	0.601396676	0.526	0.637	7.51E-70
T cell	CIRBP	7.67E-265	0.596525934	0.688	0.748	1.78E-260
T cell	TLN1	1.69E-08	0.589890708	0.286	0.331	0.000393843
T cell	EIF1	0	0.585475919	0.957	0.957	0
T cell	GLIPR1	7.23E-208	0.584636056	0.308	0.176	1.68E-203

T cell	FXYD5	1.98E-86	0.580643944	0.456	0.454	4.59E-82
T cell	PPP1CA	2.62E-95	0.576823507	0.543	0.664	6.08E-91
T cell	ATM	1.41E-15	0.573802332	0.249	0.25	3.28E-11
T cell	KMT2E	3.79E-29	0.569127123	0.468	0.601	8.82E-25
T cell	BIN1	3.08E-06	0.568463313	0.276	0.327	0.071735795
T cell	PRKACB	0.001027026	0.567864158	0.232	0.27	1
T cell	RPS29	0	0.561565249	0.974	0.972	0
T cell	COTL1	2.08E-122	0.558270995	0.51	0.49	4.83E-118
T cell	TAF7	6.32E-30	0.558123738	0.455	0.572	1.47E-25
T cell	MEAF6	0.006567618	0.546385474	0.337	0.44	1
T cell	EEF1D	0	0.54568466	0.897	0.908	0
T cell	MCL1	3.05E-51	0.544990515	0.515	0.616	7.10E-47
T cell	SELENOT	5.58E-19	0.54477547	0.43	0.552	1.30E-14
T cell	HLA-F	2.74E-08	0.544359359	0.356	0.437	0.000637798
T cell	TNFAIP8	8.58E-85	0.541475052	0.311	0.248	2.00E-80
T cell	ANAPC16	9.65E-119	0.541075487	0.588	0.714	2.24E-114
T cell	NCOR1	5.43E-07	0.537052979	0.406	0.548	0.012627998
T cell	PPP1R15A	1.83E-17	0.536798417	0.473	0.595	4.26E-13
T cell	GPBP1	1.64E-06	0.536248035	0.418	0.558	0.038155327
T cell	DDX17	2.76E-22	0.533481128	0.491	0.652	6.42E-18
T cell	LDHB	2.04E-85	0.531765409	0.454	0.425	4.75E-81
T cell	TUBA1A	1.14E-91	0.529034128	0.293	0.222	2.65E-87
T cell	LNPEP	0.002396644	0.527539711	0.226	0.257	1
T cell	ZFP36	3.36E-48	0.527419825	0.582	0.694	7.81E-44
T cell	C12orf57	9.82E-07	0.524548465	0.404	0.534	0.022826038
T cell	DHX36	0.005494395	0.52433625	0.292	0.404	1
T cell	CCSER2	0.006559456	0.521647352	0.205	0.268	1
T cell	APMAP	2.90E-09	0.521093902	0.207	0.292	6.74E-05
T cell	PSIP1	1.13E-05	0.520471182	0.249	0.279	0.262653877
T cell	KLF6	3.41E-25	0.519856055	0.592	0.696	7.94E-21
T cell	RCSD1	3.28E-227	0.518683856	0.252	0.121	7.62E-223
T cell	KANSL1	1.86E-08	0.518634972	0.236	0.329	0.000432946
T cell	CAP1	7.27E-62	0.51626511	0.53	0.65	1.69E-57
T cell	DDIT4	1.65E-13	0.513541869	0.297	0.316	3.84E-09
T cell	UBE2D3	2.63E-114	0.512741683	0.601	0.735	6.11E-110
T cell	RNF167	6.82E-08	0.511141758	0.234	0.331	0.001586419
T cell	DNAJB14	4.60E-05	0.510188293	0.225	0.303	1
T cell	C11orf58	1.22E-40	0.509151806	0.508	0.655	2.85E-36
T cell	STAT3	2.29E-07	0.508059424	0.3	0.437	0.005313953
T cell	PRMT2	7.00E-10	0.506391093	0.233	0.332	1.63E-05
T cell	CASP4	0.000627192	0.50405676	0.264	0.307	1
T cell	CAPG	0.000835913	0.499464275	0.38	0.496	1
T cell	SMCHD1	2.89E-07	0.495557892	0.306	0.351	0.006722642

T cell	EPB41	2.50E-06	0.494939404	0.189	0.252	0.058161569
T cell	DPP7	0.005301718	0.485889217	0.293	0.404	1
T cell	RPS19	0	0.484988821	0.98	0.975	0
T cell	SIGIRR	1.25E-21	0.480292355	0.246	0.382	2.91E-17
T cell	HNRNPH1	2.35E-07	0.479232254	0.404	0.593	0.00547018
T cell	CDKN1B	1.27E-10	0.473961144	0.22	0.312	2.96E-06
T cell	IK	3.72E-11	0.469737314	0.298	0.447	8.66E-07
T cell	TPR	3.32E-07	0.469683845	0.326	0.484	0.00771094
T cell	TRABD	1.05E-09	0.469335142	0.243	0.344	2.43E-05
T cell	PSME1	3.36E-92	0.468099395	0.613	0.742	7.81E-88
T cell	TTC19	3.02E-09	0.46789785	0.264	0.38	7.03E-05
T cell	SMARCA2	4.22E-05	0.467154049	0.206	0.274	0.981738302
T cell	UBAC2	3.39E-07	0.46595123	0.212	0.292	0.007892582
T cell	CAPZA1	7.30E-05	0.464252144	0.43	0.583	1
T cell	RBM39	2.76E-84	0.463025462	0.608	0.748	6.42E-80
T cell	SURF4	2.18E-25	0.461870437	0.241	0.38	5.08E-21
T cell	GSTK1	4.09E-25	0.458200987	0.5	0.634	9.51E-21
T cell	ZNF207	7.27E-10	0.453823417	0.33	0.497	1.69E-05
T cell	ADAR	4.58E-25	0.453137858	0.241	0.372	1.06E-20
T cell	THRAP3	0.000329906	0.449991149	0.337	0.49	1
T cell	CFLAR	1.41E-12	0.449705034	0.275	0.403	3.27E-08
T cell	CDC42	1.78E-60	0.448783532	0.605	0.74	4.13E-56
T cell	EVI2B	8.49E-202	0.448182217	0.385	0.237	1.97E-197
T cell	TPM3	4.77E-46	0.446983453	0.552	0.69	1.11E-41
T cell	ILF3-DT	7.20E-08	0.446623195	0.212	0.287	0.001673473
T cell	SMC3	2.54E-12	0.444166232	0.265	0.39	5.90E-08
T cell	TAP1	6.73E-15	0.443236233	0.234	0.342	1.57E-10
T cell	RPLP1	0	0.44321981	0.983	0.983	0
T cell	HNRNPK	7.68E-131	0.441911206	0.666	0.78	1.79E-126
T cell	M6PR	1.89E-09	0.44093371	0.265	0.379	4.39E-05
T cell	KMT2A	1.85E-18	0.440260569	0.205	0.301	4.31E-14
T cell	HP1BP3	5.19E-12	0.440018397	0.319	0.483	1.21E-07
T cell	EMP3	5.89E-189	0.439716278	0.369	0.237	1.37E-184
T cell	CIB1	2.16E-31	0.439209511	0.518	0.654	5.03E-27
T cell	CHD2	6.19E-14	0.436421082	0.208	0.296	1.44E-09
T cell	REX1BD	2.48E-06	0.436081249	0.337	0.5	0.057614652
T cell	CAPZB	8.23E-18	0.435567886	0.475	0.625	1.91E-13
T cell	SVIP	4.85E-32	0.434815773	0.267	0.432	1.13E-27
T cell	TRIM56	1.39E-25	0.433862488	0.24	0.371	3.23E-21
T cell	EIF4A2	3.70E-132	0.433095726	0.69	0.797	8.60E-128
T cell	GNPTAB	1.40E-26	0.432980074	0.194	0.298	3.25E-22
T cell	ARL2BP	2.97E-06	0.4326364	0.195	0.262	0.06907355
T cell	OTUD6B-AS1	6.60E-31	0.431285916	0.265	0.422	1.54E-26

T cell	IRF3	1.86E-22	0.430221032	0.22	0.333	4.33E-18
T cell	SS18L2	1.31E-11	0.42582487	0.262	0.38	3.06E-07
T cell	CCNDBP1	1.68E-10	0.423375684	0.244	0.345	3.91E-06
T cell	SF3B1	8.65E-11	0.423177443	0.342	0.517	2.01E-06
T cell	ERICH1	3.85E-11	0.421914364	0.203	0.282	8.95E-07
T cell	BCLAF1	8.57E-15	0.420659114	0.342	0.528	1.99E-10
T cell	CNBP	3.36E-50	0.419915046	0.56	0.703	7.81E-46
T cell	APOL6	1.15E-17	0.418651191	0.177	0.258	2.67E-13
T cell	MORF4L1	7.42E-35	0.417879242	0.529	0.68	1.73E-30
T cell	PPM1B	1.29E-15	0.414431812	0.198	0.287	3.01E-11
T cell	SP100	0.009538897	0.413726646	0.299	0.397	1
T cell	MAPK1	1.48E-22	0.412559206	0.194	0.292	3.43E-18
T cell	CDK2AP2	7.05E-27	0.412480424	0.264	0.414	1.64E-22
T cell	TBCB	1.02E-17	0.412084333	0.281	0.431	2.37E-13
T cell	HNRNPF	2.22E-23	0.409303439	0.502	0.65	5.17E-19
T cell	TXNIP	2.70E-78	0.408337007	0.623	0.695	6.28E-74
T cell	ZRANB2	6.92E-34	0.407719307	0.264	0.423	1.61E-29
T cell	AES	6.55E-13	0.40751549	0.329	0.502	1.52E-08
T cell	ANP32E	0.001435676	0.405870772	0.202	0.259	1
T cell	TMA7	8.47E-231	0.405464734	0.825	0.868	1.97E-226
T cell	SRSF2	1.13E-06	0.405382893	0.476	0.646	0.026171333
T cell	ASH1L	2.64E-32	0.404310644	0.277	0.439	6.13E-28
T cell	DAD1	8.37E-11	0.403135763	0.473	0.638	1.95E-06
T cell	TC2N	3.29E-65	0.401590286	0.147	0.265	7.66E-61
T cell	CRBN	7.86E-23	0.400154205	0.191	0.287	1.83E-18
T cell	TROVE2	2.30E-19	0.398414959	0.191	0.281	5.35E-15
T cell	EML4	1.22E-51	0.397787475	0.237	0.4	2.83E-47
T cell	ZMYM2	1.03E-35	0.396818508	0.202	0.32	2.40E-31
T cell	NSD1	1.89E-21	0.396791426	0.213	0.315	4.39E-17
T cell	TANK	1.38E-17	0.396510352	0.204	0.295	3.22E-13
T cell	ISG20	1.87E-81	0.396439065	0.346	0.283	4.34E-77
T cell	AKIRIN1	1.52E-21	0.396224612	0.198	0.297	3.52E-17
T cell	LSM14A	9.13E-22	0.395223801	0.266	0.411	2.12E-17
T cell	RPS4Y1	4.30E-41	0.391449619	0.492	0.488	1.00E-36
T cell	METTL23	1.58E-24	0.390332587	0.193	0.293	3.67E-20
T cell	DDX46	4.63E-13	0.38967273	0.338	0.517	1.08E-08
T cell	MAGED2	6.50E-22	0.387457904	0.18	0.271	1.51E-17
T cell	H3F3B	0	0.386870965	0.924	0.949	0
T cell	CNN2	4.41E-10	0.38650913	0.262	0.37	1.03E-05
T cell	HNRNPA0	0.001028535	0.386417015	0.439	0.604	1
T cell	SLC2A3	8.65E-234	0.383863364	0.285	0.14	2.01E-229
T cell	FNBP4	8.16E-28	0.383580473	0.224	0.343	1.90E-23
T cell	ZNF800	1.91E-27	0.383510016	0.18	0.275	4.43E-23

T cell	UBC	1.45E-177	0.383222225	0.8	0.866	3.36E-173
T cell	MIER1	1.22E-39	0.382252428	0.203	0.329	2.85E-35
T cell	PCNP	1.19E-18	0.380000842	0.315	0.489	2.76E-14
T cell	BBX	4.61E-25	0.379898846	0.229	0.346	1.07E-20
T cell	DNAJC8	4.23E-22	0.379857342	0.294	0.458	9.83E-18
T cell	UHMK1	2.88E-36	0.379318465	0.202	0.322	6.69E-32
T cell	KDM5A	3.37E-36	0.379276063	0.196	0.31	7.84E-32
T cell	SFPQ	0.001610497	0.378282497	0.474	0.652	1
T cell	LTB	1.91E-129	0.377649189	0.367	0.231	4.45E-125
T cell	SNRPB2	3.48E-05	0.377218406	0.381	0.567	0.808222801
T cell	RBPJ	9.57E-64	0.377103401	0.218	0.373	2.23E-59
T cell	SCAF11	0.00175627	0.376039257	0.406	0.597	1
T cell	PTPRA	5.28E-37	0.375921584	0.172	0.275	1.23E-32
T cell	CCDC107	7.26E-46	0.374257776	0.212	0.351	1.69E-41
T cell	RBM23	8.67E-26	0.370802287	0.196	0.295	2.02E-21
T cell	NUCB2	3.54E-25	0.369631707	0.229	0.349	8.23E-21
T cell	RSRC2	1.38E-24	0.369403343	0.333	0.525	3.21E-20
T cell	POLR2B	7.18E-40	0.369064167	0.186	0.301	1.67E-35
T cell	DR1	2.89E-23	0.36849366	0.177	0.265	6.72E-19
T cell	PIN1	1.35E-48	0.367135302	0.241	0.409	3.14E-44
T cell	COMMD6	2.92E-107	0.366709689	0.699	0.789	6.80E-103
T cell	OFD1	1.09E-30	0.365486663	0.253	0.393	2.53E-26
T cell	ARPC4	9.27E-07	0.36494845	0.366	0.541	0.021564727
T cell	WAPL	2.41E-29	0.364119402	0.198	0.305	5.60E-25
T cell	GABPB1-AS1	5.58E-29	0.362714112	0.179	0.27	1.30E-24
T cell	CCNI	6.97E-85	0.36057062	0.679	0.803	1.62E-80
T cell	SAR1A	1.31E-70	0.360446365	0.221	0.394	3.04E-66
T cell	DDX18	1.77E-19	0.359329969	0.341	0.536	4.11E-15
T cell	ARPC5L	1.34E-36	0.357154501	0.28	0.458	3.12E-32
T cell	IFI27L2	1.46E-34	0.35553055	0.281	0.454	3.40E-30
T cell	RPS27A	0	0.35549579	0.97	0.964	0
T cell	ANKRD11	7.59E-47	0.353878301	0.273	0.456	1.77E-42
T cell	SHISA5	1.41E-50	0.35343619	0.202	0.341	3.27E-46
T cell	HNRNPM	0.005427437	0.352285787	0.395	0.569	1
T cell	SH3KBP1	4.69E-32	0.352030525	0.278	0.439	1.09E-27
T cell	PNN	2.22E-16	0.351589472	0.337	0.515	5.17E-12
T cell	ARRDC3	3.79E-31	0.351262855	0.178	0.273	8.82E-27
T cell	MYCBP2	8.89E-18	0.35018475	0.235	0.339	2.07E-13
T cell	ATF6B	4.26E-55	0.349690872	0.238	0.403	9.91E-51
T cell	MED10	4.86E-44	0.348912139	0.221	0.363	1.13E-39
T cell	PCBP1	4.90E-09	0.348742689	0.501	0.685	0.000113874
T cell	HSPA8	2.83E-51	0.348724975	0.645	0.731	6.59E-47
T cell	ERGIC2	2.06E-54	0.347843966	0.209	0.355	4.79E-50

T cell	BDP1	8.92E-59	0.346980012	0.196	0.335	2.07E-54
T cell	USP15	7.24E-16	0.346946612	0.21	0.297	1.68E-11
T cell	MDM4	4.24E-29	0.346860679	0.268	0.406	9.86E-25
T cell	IAH1	2.20E-39	0.346732098	0.223	0.359	5.12E-35
T cell	REST	7.72E-50	0.346384413	0.172	0.29	1.80E-45
T cell	ARID4A	1.50E-25	0.346144559	0.176	0.265	3.49E-21
T cell	KLF13	3.42E-37	0.346020658	0.171	0.274	7.94E-33
T cell	PFDN5	7.37E-265	0.343336663	0.862	0.911	1.71E-260
T cell	LUC7L3	7.08E-22	0.342921256	0.359	0.565	1.65E-17
T cell	TRMT112	9.97E-07	0.342852594	0.486	0.655	0.023180628
T cell	GGNBP2	5.19E-45	0.342851385	0.269	0.446	1.21E-40
T cell	FNTA	2.20E-42	0.342362777	0.203	0.331	5.12E-38
T cell	PSMB10	1.39E-30	0.342216433	0.278	0.441	3.22E-26
T cell	RBMX	0.00023272	0.342084209	0.403	0.589	1
T cell	CYBC1	4.34E-07	0.341426226	0.196	0.257	0.010102402
T cell	SRSF8	1.67E-53	0.341351853	0.221	0.373	3.88E-49
T cell	GPATCH8	1.34E-38	0.340782313	0.172	0.276	3.13E-34
T cell	WTAP	1.07E-29	0.340385298	0.277	0.436	2.48E-25
T cell	RPS6KA3	3.21E-73	0.339652084	0.17	0.307	7.46E-69
T cell	H1FX	6.13E-67	0.338767168	0.22	0.385	1.43E-62
T cell	MED4	5.31E-54	0.338254997	0.25	0.428	1.24E-49
T cell	CHURC1	6.28E-15	0.338253599	0.368	0.575	1.46E-10
T cell	STAT1	6.04E-42	0.338157935	0.17	0.275	1.41E-37
T cell	PAK2	3.16E-46	0.336781607	0.258	0.429	7.35E-42
T cell	RBM8A	9.51E-12	0.33615148	0.373	0.576	2.21E-07
T cell	NAP1L1	7.90E-49	0.335824167	0.613	0.716	1.84E-44
T cell	CFAP97	2.12E-62	0.335404249	0.178	0.312	4.94E-58
T cell	DGCR6L	4.31E-70	0.335384751	0.187	0.335	1.00E-65
T cell	TLE4	5.35E-25	0.335044886	0.18	0.264	1.24E-20
T cell	IRF2	2.24E-23	0.334809285	0.192	0.285	5.22E-19
T cell	RPLP2	0	0.334677244	0.974	0.974	0
T cell	SLC38A1	2.07E-61	0.33381185	0.242	0.418	4.82E-57
T cell	RSBN1L	9.17E-69	0.333556604	0.222	0.391	2.13E-64
T cell	C19orf25	1.37E-36	0.33245859	0.169	0.267	3.20E-32
T cell	PTPN2	2.16E-67	0.331221723	0.183	0.322	5.03E-63
T cell	RPL17	2.90E-139	0.329106806	0.79	0.847	6.74E-135
T cell	RNF187	4.89E-55	0.328892272	0.204	0.349	1.14E-50
T cell	HSPB11	1.81E-52	0.328883515	0.198	0.334	4.21E-48
T cell	ZC3H13	8.97E-72	0.328296093	0.206	0.366	2.09E-67
T cell	GRAMD1A	1.70E-64	0.328095034	0.159	0.283	3.96E-60
T cell	RNPEPL1	1.32E-51	0.327731358	0.181	0.306	3.07E-47
T cell	DYNLT3	6.33E-43	0.327462846	0.163	0.267	1.47E-38
T cell	METTL9	1.40E-26	0.32696542	0.307	0.489	3.26E-22

T cell	THUMPD3-AS1	3.15E-68	0.326560711	0.232	0.401	7.33E-64
T cell	TAPBP	3.86E-28	0.326528341	0.351	0.566	8.98E-24
T cell	ZBTB38	3.10E-77	0.326527938	0.215	0.388	7.20E-73
T cell	SIPA1	2.05E-18	0.326293541	0.178	0.256	4.76E-14
T cell	GALM	1.74E-74	0.324690475	0.144	0.267	4.05E-70
T cell	CCNL1	0.001106405	0.324624243	0.449	0.653	1
T cell	MARK3	6.14E-64	0.323901392	0.162	0.286	1.43E-59
T cell	MYH9	5.19E-23	0.323620254	0.333	0.524	1.21E-18
T cell	ZBTB20	2.78E-32	0.323542857	0.235	0.36	6.46E-28
T cell	RNASEH2B	1.49E-41	0.323195479	0.213	0.343	3.47E-37
T cell	TMEM230	1.27E-20	0.320294793	0.34	0.535	2.96E-16
T cell	CTDSP1	2.97E-43	0.320195948	0.173	0.282	6.91E-39
T cell	YPEL3	1.83E-14	0.319549018	0.184	0.258	4.25E-10
T cell	PCF11	1.22E-49	0.31878336	0.211	0.346	2.84E-45
T cell	FAM173A	2.45E-84	0.316831748	0.209	0.385	5.69E-80
T cell	ISCU	6.36E-28	0.316778867	0.337	0.548	1.48E-23
T cell	BLOC1S4	2.90E-43	0.316550025	0.168	0.277	6.75E-39
T cell	NUTM2B-AS1	1.31E-57	0.315016806	0.163	0.28	3.06E-53
T cell	SRSF11	8.31E-30	0.314507991	0.353	0.572	1.93E-25
T cell	ADD1	1.27E-59	0.31422041	0.165	0.286	2.96E-55
T cell	TTC1	1.57E-76	0.314219293	0.216	0.39	3.66E-72
T cell	PJA2	7.72E-50	0.31404186	0.175	0.29	1.80E-45
T cell	RNF115	2.34E-65	0.313401254	0.14	0.251	5.43E-61
T cell	RBM25	1.25E-25	0.313267505	0.354	0.565	2.90E-21
T cell	MYADM	2.50E-66	0.313262056	0.17	0.297	5.82E-62
T cell	TMEM50A	2.58E-07	0.313132614	0.394	0.597	0.005997175
T cell	CDK13	5.42E-54	0.313060028	0.176	0.297	1.26E-49
T cell	GUK1	1.71E-26	0.312985161	0.571	0.717	3.98E-22
T cell	CCDC12	4.41E-60	0.312427723	0.275	0.477	1.02E-55
T cell	VPS13C	9.81E-52	0.312161849	0.182	0.302	2.28E-47
T cell	MFSD10	1.65E-68	0.312100812	0.205	0.363	3.83E-64
T cell	EXOC7	2.77E-51	0.31191206	0.161	0.271	6.43E-47
T cell	RNPS1	1.40E-34	0.310169765	0.315	0.515	3.25E-30
T cell	PPHLN1	2.51E-56	0.309791398	0.211	0.357	5.84E-52
T cell	NCL	1.45E-17	0.309654167	0.562	0.695	3.38E-13
T cell	ALKBH5	2.06E-52	0.308961552	0.164	0.279	4.79E-48
T cell	SNX17	4.64E-57	0.308382822	0.223	0.38	1.08E-52
T cell	UBE2L6	2.54E-24	0.307911233	0.189	0.284	5.90E-20
T cell	PPIG	6.67E-24	0.307898245	0.355	0.567	1.55E-19
T cell	INTS6	1.65E-73	0.307785314	0.167	0.298	3.83E-69
T cell	SF3B2	3.91E-46	0.307522418	0.306	0.518	9.09E-42
T cell	NSD3	4.12E-67	0.307357181	0.231	0.403	9.59E-63

T cell	HNRNPA2B1	1.27E-96	0.306258831	0.771	0.858	2.95E-92
T cell	CNOT4	7.91E-65	0.305952038	0.16	0.281	1.84E-60
T cell	RPS15A	0	0.30545897	0.969	0.959	0
T cell	DNTTIP2	8.08E-43	0.305381772	0.158	0.258	1.88E-38
T cell	SLTM	1.29E-61	0.305120409	0.273	0.473	3.00E-57
T cell	TAOK3	1.82E-58	0.304023667	0.272	0.461	4.23E-54
T cell	TMEM160	1.80E-47	0.303695481	0.271	0.454	4.18E-43
T cell	BAZ1B	3.73E-66	0.303659696	0.191	0.336	8.67E-62
T cell	CALM3	1.54E-20	0.303144035	0.346	0.543	3.57E-16
T cell	DNAJB1	1.74E-130	0.302334507	0.234	0.452	4.04E-126
T cell	LBR	5.64E-43	0.29940064	0.223	0.361	1.31E-38
T cell	SRSF4	2.02E-71	0.299091909	0.243	0.431	4.70E-67
T cell	TUBA4A	8.38E-53	0.298931066	0.199	0.332	1.95E-48
T cell	WDR33	2.21E-97	0.298930249	0.187	0.355	5.14E-93
T cell	NARF	4.99E-81	0.298768423	0.16	0.296	1.16E-76
T cell	RPL36AL	2.03E-73	0.298324101	0.739	0.84	4.71E-69
T cell	OSBPL8	2.14E-33	0.29829379	0.201	0.312	4.99E-29
T cell	HAX1	1.23E-88	0.297264618	0.227	0.419	2.85E-84
T cell	CGGBP1	7.47E-69	0.296996142	0.174	0.309	1.74E-64
T cell	PHIP	5.31E-80	0.296746907	0.237	0.423	1.24E-75
T cell	FYTTD1	1.61E-41	0.296725343	0.155	0.252	3.73E-37
T cell	DNAJC1	7.69E-106	0.296496469	0.182	0.356	1.79E-101
T cell	CLIP1	8.81E-54	0.295600951	0.148	0.254	2.05E-49
T cell	BAX	6.39E-33	0.2955144	0.337	0.541	1.49E-28
T cell	EXOSC1	5.03E-76	0.295324397	0.146	0.269	1.17E-71
T cell	ATG12	1.93E-70	0.295028197	0.205	0.36	4.48E-66
T cell	PAFAH1B1	1.76E-76	0.294683414	0.176	0.317	4.08E-72
T cell	TRA2B	2.00E-32	0.294563913	0.342	0.549	4.65E-28
T cell	TMEM9B	3.09E-63	0.294339151	0.252	0.438	7.19E-59
T cell	SDHAF2	3.13E-53	0.294025131	0.148	0.254	7.27E-49
T cell	FOSB	3.31E-09	0.293329415	0.463	0.641	7.69E-05
T cell	ATP6AP2	5.03E-37	0.293023952	0.293	0.472	1.17E-32
T cell	DERL1	1.93E-86	0.292587649	0.186	0.345	4.50E-82
T cell	FUBP1	4.84E-70	0.292303274	0.179	0.318	1.13E-65
T cell	PRPF4B	8.85E-91	0.290881996	0.23	0.423	2.06E-86
T cell	NEDD9	1.22E-55	0.290363673	0.164	0.279	2.84E-51
T cell	RABGAP1L	5.23E-63	0.290148035	0.161	0.28	1.22E-58
T cell	WDR82	1.89E-70	0.28977337	0.147	0.267	4.40E-66
T cell	ZNF655	2.12E-38	0.289338578	0.159	0.254	4.94E-34
T cell	SNRNP200	1.35E-90	0.289190886	0.153	0.292	3.14E-86
T cell	ERBIN	5.63E-39	0.288960763	0.174	0.275	1.31E-34
T cell	PPP1R10	5.73E-77	0.288684705	0.182	0.326	1.33E-72
T cell	TOMM7	1.59E-59	0.288595935	0.7	0.816	3.69E-55

T cell	MGAT1	2.75E-51	0.288317226	0.188	0.312	6.39E-47
T cell	SLK	1.94E-83	0.287818838	0.159	0.297	4.50E-79
T cell	TM2D3	2.30E-76	0.286609741	0.154	0.281	5.34E-72
T cell	THUMPD1	2.67E-94	0.285907894	0.165	0.315	6.22E-90
T cell	CTCF	4.92E-78	0.285724045	0.16	0.293	1.14E-73
T cell	RSF1	4.27E-88	0.285592076	0.235	0.43	9.94E-84
T cell	WBP11	2.47E-75	0.285553587	0.16	0.291	5.74E-71
T cell	NEMF	1.03E-79	0.28472483	0.227	0.404	2.39E-75
T cell	TWF2	6.71E-35	0.284705371	0.177	0.278	1.56E-30
T cell	SNX6	1.26E-71	0.283917413	0.242	0.428	2.93E-67
T cell	SHKBP1	2.87E-36	0.283913138	0.165	0.261	6.67E-32
T cell	ABT1	6.26E-71	0.283879005	0.162	0.293	1.46E-66
T cell	SAFB2	7.75E-77	0.283676708	0.167	0.303	1.80E-72
T cell	TIAL1	3.79E-103	0.283116866	0.191	0.367	8.81E-99
T cell	DAZAP2	4.92E-07	0.282092027	0.432	0.651	0.011431005
T cell	SIRT7	2.69E-62	0.281599867	0.151	0.266	6.25E-58
T cell	UBTF	7.60E-61	0.281416695	0.149	0.261	1.77E-56
T cell	TRAM1	6.82E-37	0.280305168	0.316	0.509	1.59E-32
T cell	PLA2G16	0.000287338	0.279399115	0.221	0.279	1
T cell	SMARCA5	1.55E-105	0.278887119	0.199	0.382	3.61E-101
T cell	USP47	5.12E-70	0.277573938	0.158	0.283	1.19E-65
T cell	ARPC5	5.97E-13	0.27650192	0.53	0.686	1.39E-08
T cell	KRR1	2.92E-92	0.27593947	0.144	0.277	6.78E-88
T cell	GTF3A	1.06E-14	0.274903722	0.367	0.554	2.47E-10
T cell	U2SURP	1.16E-91	0.274448405	0.253	0.466	2.70E-87
T cell	TRIR	0.009168864	0.274298747	0.506	0.708	1
T cell	WDR1	1.39E-29	0.274096122	0.319	0.505	3.22E-25
T cell	NOP53	7.43E-55	0.273737745	0.692	0.8	1.73E-50
T cell	HNRNPH2	2.22E-63	0.273093053	0.213	0.366	5.16E-59
T cell	TAF12	1.78E-53	0.272349643	0.153	0.26	4.15E-49
T cell	ACTR2	0.000250462	0.271831617	0.44	0.644	1
T cell	USP16	1.63E-107	0.271717205	0.261	0.496	3.79E-103
T cell	NSMCE1	1.43E-103	0.271290606	0.177	0.344	3.32E-99
T cell	BPTF	1.97E-79	0.270309068	0.247	0.441	4.58E-75
T cell	UBA52	3.13E-229	0.269151163	0.924	0.942	7.28E-225
T cell	FAM3C	6.79E-116	0.268793419	0.213	0.41	1.58E-111
T cell	CMC2	2.76E-88	0.268616887	0.161	0.305	6.42E-84
T cell	PPP4R3A	1.98E-83	0.268440459	0.166	0.306	4.61E-79
T cell	SRP19	9.81E-87	0.267765437	0.23	0.42	2.28E-82
T cell	TLK1	1.28E-113	0.267279981	0.176	0.346	2.99E-109
T cell	PNKP	1.27E-84	0.267269995	0.138	0.262	2.96E-80
T cell	ZNF638	8.07E-108	0.264660576	0.189	0.363	1.88E-103
T cell	YIPF5	6.69E-83	0.264464539	0.138	0.262	1.56E-78

T cell	CCDC115	3.86E-81	0.264328053	0.167	0.307	8.97E-77
T cell	ZNF292	2.02E-75	0.264227034	0.21	0.37	4.71E-71
T cell	EIF3G	2.54E-14	0.262452421	0.405	0.63	5.90E-10
T cell	POLR2G	9.82E-73	0.262414567	0.233	0.414	2.28E-68
T cell	PA2G4	0.000199182	0.262414564	0.41	0.571	1
T cell	MITD1	7.54E-67	0.262316863	0.157	0.277	1.75E-62
T cell	ZNF428	1.38E-90	0.262242368	0.182	0.342	3.21E-86
T cell	UGP2	1.16E-27	0.262116567	0.339	0.535	2.71E-23
T cell	RTF1	6.78E-78	0.260788396	0.287	0.515	1.58E-73
T cell	CASP1	3.62E-25	0.260650807	0.216	0.32	8.42E-21
T cell	OPTN	1.44E-63	0.259254237	0.17	0.296	3.34E-59
T cell	TADA3	8.89E-107	0.258900389	0.174	0.34	2.07E-102
T cell	LASP1	2.76E-100	0.258685767	0.21	0.396	6.42E-96
T cell	OTUB1	4.38E-114	0.257931894	0.178	0.35	1.02E-109
T cell	PYURF	1.37E-30	0.257903326	0.334	0.543	3.18E-26
T cell	MAT2B	4.42E-101	0.257800492	0.185	0.352	1.03E-96
T cell	SMC4	1.78E-49	0.257035153	0.163	0.269	4.14E-45
T cell	IMP3	9.09E-80	0.256866373	0.231	0.419	2.11E-75
T cell	BAZ1A	3.90E-67	0.256398555	0.244	0.418	9.06E-63
T cell	RIF1	1.15E-99	0.255747292	0.144	0.282	2.68E-95
T cell	BST2	3.61E-09	0.254813562	0.3	0.306	8.39E-05
T cell	COA1	1.19E-85	0.254630577	0.147	0.277	2.76E-81
T cell	SNW1	1.58E-91	0.254502854	0.191	0.357	3.67E-87
T cell	CFL1	2.36E-119	0.25376684	0.893	0.915	5.49E-115
T cell	TOX4	1.13E-115	0.253741401	0.161	0.323	2.64E-111
T cell	CCDC59	1.10E-127	0.253572558	0.184	0.37	2.55E-123
T cell	SETX	1.63E-36	0.25344157	0.17	0.266	3.79E-32
T cell	UPF2	1.49E-68	0.252700049	0.241	0.413	3.46E-64
T cell	ARPP19	1.83E-100	0.252179989	0.174	0.333	4.25E-96
T cell	CXCR4	3.43E-305	0.251279049	0.508	0.283	7.98E-301
T cell	CDC26	2.37E-101	0.251062445	0.187	0.359	5.51E-97
T cell	SNHG9	9.54E-73	0.250712518	0.143	0.259	2.22E-68
T cell	TMCO1	1.19E-45	0.250580049	0.344	0.579	2.78E-41
T cell	SKP1	2.58E-34	0.250252215	0.669	0.79	6.01E-30
T cell	CPNE1	2.89E-81	0.250195493	0.194	0.353	6.72E-77
