# Supplementary material for: Low expression of the dynamic network markers FOS/JUN in pre-deteriorated epithelial cells is associated with the progression of colorectal adenoma to carcinoma
Source: J Transl Med. 2023 Jan 25;21:45. doi: 10.1186/s12967-023-03890-5 (PMC9875500; doi:10.1186/s12967-023-03890-5)
Supplement: Supplementary file 9 — Additional file 9: Table S2. Signature genes for 7 clusters of epithelial cells. [file 12967_2023_3890_MOESM9_ESM.pdf]

Table S2. Signature genes for 7 clusters of epithelial cells

cluster	gene	p_val	avg_log2FC	pct.1	pct.2	p_val_adj
Benign	SELENBP1	0	1.826474877	0.931	0.609	0
Benign	REG1A	0	1.814560218	0.423	0.073	0
Benign	ASCL2	0	1.623827109	0.926	0.409	0
Benign	APCDD1	0	1.594973438	0.619	0.045	0
Benign	PCCA	0	1.538237197	0.708	0.288	0
Benign	DPEP1	0	1.514141375	0.71	0.217	0
Benign	ID3	2.56E-303	1.50972971	0.794	0.536	5.44E-299
Benign	NKD1	0	1.262245138	0.592	0.101	0
Benign	LEFTY1	0	1.197946451	0.927	0.513	0
Benign	SLC12A2	0	1.025988702	0.993	0.739	0
Benign	FERMT1	0	0.994844379	0.887	0.476	0
Benign	ID1	1.69E-184	0.942256291	0.82	0.656	3.59E-180
Benign	AXIN2	0	0.917578744	0.75	0.331	0
Benign	REPIN1	0	0.861659834	0.844	0.523	0
Benign	ID4	0	0.861015082	0.573	0.093	0
Benign	ITGA6	0	0.848705231	0.899	0.653	0
Benign	RNF43	0	0.824665817	0.848	0.511	0
Benign	GOLIM4	0	0.807870474	0.94	0.692	0
Benign	PRSS23	0	0.801521531	0.801	0.397	0
Benign	CXCL1	6.10E-157	0.794705783	0.441	0.191	1.30E-152
Benign	RPL26	0	0.785100319	1	0.944	0
Benign	ETS2	0	0.78507133	0.936	0.627	0
Benign	PRDX5	0	0.776738605	0.998	0.887	0
Benign	SMOC2	0	0.769525383	0.329	0.036	0
Benign	IMPDH2	0	0.768506053	0.907	0.608	0
Benign	CXCL2	2.12E-107	0.765828262	0.456	0.244	4.50E-103
Benign	RPS5	0	0.733640606	0.999	0.907	0
Benign	CD74	1.08E-106	0.732659901	0.773	0.564	2.29E-102
Benign	RGMB	0	0.72191085	0.612	0.216	0
Benign	GPX2	0	0.717300134	0.991	0.805	0
Benign	RPL3	0	0.715327049	1	0.953	0
Benign	CXCL3	3.55E-136	0.712253118	0.601	0.338	7.55E-132
Benign	RPLP0	0	0.700591899	1	0.913	0
Benign	NOP53	0	0.692160061	0.979	0.811	0
Benign	EEF1A1	0	0.688858941	1	0.969	0
Benign	EPHB3	0	0.688699951	0.687	0.256	0
Benign	RPS4X	0	0.685136627	1	0.931	0
Benign	EIF3L	0	0.675707627	0.925	0.689	0
Benign	EEF2	0	0.665694283	0.99	0.879	0
Benign	LCN2	2.78E-160	0.661919872	0.905	0.646	5.91E-156
Benign	NDUFA4	0	0.656597748	0.994	0.862	0

Benign	RACK1	0	0.652971389	1	0.917	0
Benign	RPS2	0	0.652157939	1	0.944	0
Benign	NPDC1	4.73E-288	0.648030391	0.908	0.593	1.01E-283
Benign	RPL4	0	0.645620376	0.998	0.866	0
Benign	CD44	9.19E-308	0.637425695	0.879	0.524	1.95E-303
Benign	CD9	3.13E-219	0.634199368	0.977	0.875	6.67E-215
Benign	RPL15	0	0.630795235	1	0.938	0
Benign	TSPAN8	1.05E-301	0.628942758	0.998	0.941	2.24E-297
Benign	RPL13	0	0.626630686	1	0.958	0
Benign	PRR15	3.39E-248	0.622350353	0.741	0.444	7.22E-244
Benign	RPL9	0	0.621242453	1	0.938	0
Benign	RPS16	0	0.618774035	1	0.932	0
Benign	EIF3D	3.66E-260	0.612686244	0.887	0.637	7.79E-256
Benign	RPL7A	0	0.609696093	1	0.923	0
Benign	RPS12	0	0.609586375	1	0.964	0
Benign	SLC40A1	5.90E-251	0.608771837	0.772	0.462	1.26E-246
Benign	RPL10A	0	0.607432533	1	0.901	0
Benign	CD99	2.18E-287	0.602963719	0.939	0.683	4.63E-283
Benign	TBX3	0	0.599491937	0.572	0.211	0
Benign	ITPR2	2.69E-255	0.595072114	0.53	0.215	5.72E-251
Benign	DAB2	0	0.591049568	0.61	0.253	0
Benign	SLC25A6	0	0.587533272	0.996	0.88	0
Benign	RPS9	0	0.587226006	1	0.947	0
Benign	GAS6	3.13E-241	0.579009483	0.765	0.468	6.66E-237
Benign	RPL5	0	0.577645752	1	0.895	0
Benign	ZFP36L1	9.93E-203	0.576441855	0.916	0.671	2.11E-198
Benign	RPS7	0	0.56726491	1	0.919	0
Benign	CCND2	1.75E-269	0.567248666	0.779	0.416	3.72E-265
Benign	RPL12	0	0.565543056	1	0.964	0
Benign	RPSA	2.04E-299	0.563290193	0.999	0.881	4.34E-295
Benign	HES1	9.88E-105	0.562849299	0.86	0.68	2.10E-100
Benign	TMEM230	1.25E-291	0.561353496	0.858	0.571	2.65E-287
Benign	LGR5	0	0.557856111	0.476	0.101	0
Benign	PPA1	3.93E-247	0.557068383	0.983	0.8	8.35E-243
Benign	ZFP36L2	2.35E-126	0.556779732	0.942	0.794	5.00E-122
Benign	RPL14	0	0.554130365	1	0.926	0
Benign	CDCA7	4.83E-230	0.55395826	0.695	0.37	1.03E-225
Benign	RPL23A	0	0.552883849	1	0.942	0
Benign	RPL29	0	0.549668255	1	0.951	0
Benign	RPS4Y1	1.07E-117	0.547931447	0.762	0.544	2.27E-113
Benign	TSC22D1	1.26E-157	0.543666361	0.925	0.757	2.68E-153
Benign	RPS3A	0	0.543442295	1	0.945	0
Benign	RPL34	0	0.5431496	1	0.976	0

Benign	RPL17	5.85E-261	0.538822791	0.989	0.824	1.24E-256
Benign	CCNI	3.18E-249	0.535780355	0.976	0.814	6.77E-245
Benign	EPHB2	4.05E-270	0.531038919	0.787	0.444	8.61E-266
Benign	RCN1	8.79E-233	0.529619516	0.827	0.525	1.87E-228
Benign	RPL23	5.93E-302	0.529199539	0.999	0.895	1.26E-297
Benign	RPS3	9.14E-297	0.528769824	1	0.965	1.94E-292
Benign	HNRNPA1	6.74E-244	0.52605443	0.998	0.88	1.43E-239
Benign	ZNF703	2.49E-236	0.519727582	0.786	0.486	5.29E-232
Benign	EIF3F	1.05E-243	0.516615745	0.956	0.742	2.23E-239
Benign	KRTCAP3	2.92E-251	0.513714401	0.901	0.623	6.21E-247
Benign	RPL24	0	0.511608692	0.999	0.912	0
Benign	L1TD1	0	0.510146173	0.566	0.161	0
Benign	RPL6	1.30E-264	0.508965271	1	0.907	2.76E-260
Benign	BACE2	2.05E-226	0.508081841	0.88	0.614	4.35E-222
Benign	ALDH1A1	3.28E-202	0.502774923	0.55	0.253	6.98E-198
Benign	RPL18	0	0.502419459	1	0.958	0
Benign	HLA-DRA	3.16E-42	0.500558297	0.474	0.353	6.73E-38
Benign	RPS8	5.18E-298	0.499920726	1	0.943	1.10E-293
Benign	ZKSCAN1	1.13E-187	0.495829043	0.846	0.613	2.40E-183
Benign	RPL18A	2.27E-283	0.494843347	1	0.941	4.82E-279
Benign	RPL35A	4.97E-308	0.490885741	1	0.946	1.06E-303
Benign	RPL13A	0	0.488559933	1	0.982	0
Benign	RPL19	1.14E-287	0.486336576	1	0.953	2.43E-283
Benign	NAP1L1	3.50E-214	0.484921781	0.933	0.66	7.44E-210
Benign	RPS23	0	0.482539933	1	0.929	0
Benign	PDZK1IP1	3.49E-260	0.479043922	0.883	0.551	7.41E-256
Benign	NOB1	2.92E-257	0.478388333	0.745	0.412	6.20E-253
Benign	GSTK1	4.75E-206	0.475813631	0.948	0.755	1.01E-201
Benign	NACA	3.54E-254	0.475316175	0.999	0.908	7.52E-250
Benign	RPS14	0	0.474820464	1	0.968	0
Benign	CYP4X1	0	0.469528338	0.429	0.063	0
Benign	SRPK1	1.00E-170	0.469090799	0.843	0.591	2.14E-166
Benign	IFITM3	1.09E-169	0.467671182	0.958	0.705	2.32E-165
Benign	ESD	6.82E-208	0.467635231	0.906	0.647	1.45E-203
Benign	RPL10	0	0.466741889	1	0.978	0
Benign	RPL11	0	0.466741524	1	0.97	0
Benign	GTF2I	2.03E-174	0.464632275	0.811	0.583	4.32E-170
Benign	SEMA5A	6.80E-253	0.463238203	0.556	0.242	1.45E-248
Benign	MSRB2	1.37E-193	0.462811244	0.852	0.622	2.91E-189
Benign	CD47	1.88E-210	0.461604879	0.837	0.562	4.00E-206
Benign	APIP	5.35E-223	0.461480607	0.785	0.463	1.14E-218
Benign	RPL31	1.77E-266	0.461247115	1	0.949	3.77E-262
Benign	MLEC	1.46E-186	0.461073082	0.978	0.766	3.09E-182

Benign	ALDH1B1	2.94E-232	0.460666059	0.646	0.322	6.26E-228
Benign	QPRT	1.23E-173	0.460649685	0.802	0.521	2.62E-169
Benign	TMEM9	2.79E-256	0.460041311	0.607	0.285	5.94E-252
Benign	QTRT1	1.19E-212	0.460014596	0.92	0.647	2.54E-208
Benign	COMMD6	2.93E-223	0.459703713	0.976	0.824	6.24E-219
Benign	TESC	2.57E-252	0.457536305	0.568	0.206	5.47E-248
Benign	RPS15A	3.07E-245	0.455801487	1	0.95	6.53E-241
Benign	SMAD9	0	0.454513373	0.487	0.132	0
Benign	NQO1	1.06E-143	0.451907989	0.844	0.595	2.25E-139
Benign	BTG2	1.97E-99	0.450195254	0.816	0.63	4.19E-95
Benign	MRPS33	1.03E-197	0.450131659	0.907	0.655	2.19E-193
Benign	BCL11A	1.44E-239	0.449894517	0.643	0.32	3.07E-235
Benign	PERP	1.78E-166	0.449138916	0.96	0.811	3.79E-162
Benign	PLCB4	3.42E-169	0.448468931	0.707	0.438	7.28E-165
Benign	RPL32	6.55E-267	0.447497707	1	0.954	1.39E-262
Benign	RPS27A	9.27E-272	0.447116951	1	0.955	1.97E-267
Benign	TGIF1	1.31E-185	0.446162066	0.823	0.549	2.78E-181
Benign	MYB	7.28E-234	0.445828837	0.693	0.378	1.55E-229
Benign	RPS17	9.50E-212	0.444756898	0.999	0.883	2.02E-207
Benign	BTF3	2.90E-255	0.442034087	0.997	0.904	6.17E-251
Benign	RPL21	1.97E-274	0.440452607	1	0.966	4.19E-270
Benign	RPS24	8.24E-266	0.439730271	1	0.943	1.75E-261
Benign	SH3YL1	8.95E-195	0.437654738	0.87	0.62	1.90E-190
Benign	SYPL1	2.05E-184	0.435174039	0.859	0.61	4.36E-180
Benign	C9orf16	3.49E-185	0.434394887	0.915	0.721	7.41E-181
Benign	HOXA9	1.24E-197	0.432875158	0.632	0.339	2.65E-193
Benign	PLPP2	9.80E-179	0.429264969	0.862	0.629	2.08E-174
Benign	RPS13	1.40E-254	0.428022714	1	0.921	2.98E-250
Benign	C6orf48	8.35E-179	0.42785507	0.895	0.61	1.78E-174
Benign	THRA	2.31E-227	0.427706737	0.567	0.271	4.91E-223
Benign	SORD	5.91E-242	0.425663977	0.678	0.343	1.26E-237
Benign	OLFM4	3.46E-168	0.424676455	0.892	0.564	7.36E-164
Benign	QARS	4.84E-191	0.420499374	0.871	0.625	1.03E-186
Benign	EBPL	2.48E-184	0.42009434	0.88	0.604	5.27E-180
Benign	RPL27	1.99E-213	0.419940488	1	0.914	4.22E-209
Benign	IFITM1	3.83E-174	0.4197205	0.829	0.5	8.15E-170
Benign	FBL	3.12E-177	0.419151316	0.906	0.639	6.63E-173
Benign	GDF15	2.72E-66	0.417844926	0.641	0.464	5.78E-62
Benign	TRIP6	1.65E-204	0.41620718	0.656	0.358	3.51E-200
Benign	C1QBP	1.55E-164	0.415662826	0.93	0.686	3.29E-160
Benign	MLXIP	3.47E-166	0.415471699	0.846	0.606	7.39E-162
Benign	ALDH2	8.54E-121	0.413410622	0.913	0.741	1.82E-116
Benign	ATP5F1A	1.90E-157	0.411595355	0.946	0.729	4.05E-153

Benign	ST13	1.79E-154	0.409753585	0.941	0.745	3.81E-150
Benign	RPL36	4.82E-219	0.409730633	1	0.97	1.02E-214
Benign	NIPSNAP2	6.04E-190	0.406870255	0.766	0.484	1.29E-185
Benign	MDH2	6.37E-150	0.406297768	0.962	0.768	1.35E-145
Benign	EIF3E	5.81E-142	0.404293293	0.967	0.777	1.24E-137
Benign	RPS18	9.00E-271	0.403715296	1	0.968	1.91E-266
Benign	RPS11	3.81E-239	0.402139828	0.999	0.936	8.10E-235
Benign	COX20	5.75E-147	0.400880153	0.893	0.675	1.22E-142
Benign	ZBTB38	8.12E-170	0.400813027	0.714	0.451	1.73E-165
Benign	TMEM147	3.30E-154	0.395118557	0.891	0.65	7.02E-150
Benign	EIF4B	5.94E-167	0.393859097	0.93	0.702	1.26E-162
Benign	RPS19	3.84E-208	0.39325695	1	0.969	8.17E-204
Benign	SEMA3C	2.12E-241	0.391305804	0.613	0.29	4.51E-237
Benign	H3F3A	8.90E-197	0.390923334	1	0.969	1.89E-192
Benign	RPL36A	5.30E-171	0.390735007	0.998	0.888	1.13E-166
Benign	RPS27	1.04E-218	0.389334387	1	0.985	2.21E-214
Benign	PEBP1	4.01E-145	0.388123511	0.983	0.786	8.52E-141
Benign	RSL1D1	8.86E-157	0.387837795	0.913	0.643	1.88E-152
Benign	RPL22	4.08E-175	0.387545609	0.998	0.889	8.67E-171
Benign	RUBCNL	7.51E-279	0.387530423	0.549	0.204	1.60E-274
Benign	RPS6	0	0.384662037	1	0.971	0
Benign	SERPINB1	3.37E-136	0.384462384	0.931	0.769	7.17E-132
Benign	TRIM2	1.08E-157	0.382684376	0.844	0.598	2.29E-153
Benign	IFT57	4.38E-176	0.381295138	0.696	0.419	9.32E-172
Benign	PROX1	3.60E-250	0.378795829	0.499	0.172	7.67E-246
Benign	EIF3G	1.60E-164	0.376301111	0.919	0.696	3.41E-160
Benign	FIS1	4.85E-149	0.375702026	0.928	0.739	1.03E-144
Benign	RPL41	4.62E-267	0.374437166	1	0.995	9.83E-263
Benign	EPB41L4A-AS1	8.46E-178	0.373628464	0.694	0.405	1.80E-173
Benign	RPL28	1.47E-221	0.368636748	1	0.975	3.13E-217
Benign	RPS28	3.25E-187	0.365270812	1	0.964	6.92E-183
Benign	C10orf99	1.71E-131	0.36314429	0.844	0.603	3.63E-127
Benign	RPL30	3.78E-156	0.360097754	1	0.941	8.04E-152
Benign	CD24	3.35E-109	0.360059394	0.997	0.858	7.13E-105
Benign	RPL7	5.63E-204	0.359971004	1	0.938	1.20E-199
Benign	HIBADH	1.28E-198	0.359434083	0.641	0.35	2.72E-194
Benign	ATOH8	0	0.35344743	0.353	0.05	0
Benign	RPL27A	3.40E-183	0.352907163	1	0.968	7.22E-179
Benign	RPLP1	1.97E-147	0.348947772	1	0.982	4.19E-143
Benign	TOMM7	7.32E-140	0.348877968	0.985	0.845	1.56E-135
Benign	PNRC1	1.77E-118	0.348101357	0.788	0.567	3.75E-114
Benign	APEX1	3.76E-138	0.348014248	0.827	0.563	8.00E-134

Benign	HLA-DRB1	1.06E-27	0.34781477	0.419	0.325	2.25E-23
Benign	RAB13	2.52E-146	0.347014037	0.828	0.591	5.35E-142
Benign	EIF3M	2.74E-139	0.344698128	0.921	0.684	5.83E-135
Benign	EIF2A	9.84E-140	0.342706811	0.799	0.553	2.09E-135
Benign	KCNN4	3.08E-165	0.342433883	0.709	0.416	6.55E-161
Benign	LDLRAD4	5.20E-256	0.342163231	0.446	0.142	1.11E-251
Benign	SSBP1	8.88E-122	0.341990299	0.926	0.721	1.89E-117
Benign	HDDC2	6.88E-142	0.340976224	0.713	0.471	1.46E-137
Benign	CRNDE	6.97E-177	0.340313572	0.446	0.185	1.48E-172
Benign	SLC44A3	4.79E-173	0.340108276	0.671	0.386	1.02E-168
Benign	CLDN2	1.60E-184	0.339216696	0.56	0.245	3.40E-180
Benign	CDX2	1.11E-91	0.338799985	0.875	0.701	2.36E-87
Benign	POLR1D	8.01E-139	0.338708715	0.922	0.717	1.70E-134
Benign	ARMC10	6.44E-148	0.338045026	0.732	0.477	1.37E-143
Benign	ZNRF3	2.08E-204	0.334729217	0.45	0.181	4.43E-200
Benign	ATP5MC2	5.06E-122	0.334609383	0.991	0.862	1.08E-117
Benign	EEF1B2	1.29E-114	0.334091899	0.997	0.87	2.75E-110
Benign	ANKRD22	2.42E-177	0.333487211	0.554	0.275	5.15E-173
Benign	GALNT6	9.63E-226	0.333467795	0.538	0.227	2.05E-221
Benign	RPLP2	5.03E-150	0.332932187	1	0.972	1.07E-145
Benign	IGBP1	4.64E-134	0.330877779	0.81	0.575	9.86E-130
Benign	HOXB9	6.37E-151	0.329218424	0.706	0.432	1.36E-146
Benign	NOTCH1	1.08E-207	0.327892347	0.479	0.199	2.30E-203
Benign	UPF3A	1.20E-143	0.326673513	0.692	0.44	2.56E-139
Benign	PSMA2	1.51E-122	0.323964065	0.948	0.741	3.21E-118
Benign	NDUFA5	2.08E-119	0.323855715	0.871	0.67	4.43E-115
Benign	TNRC6B	2.91E-143	0.322615001	0.707	0.437	6.18E-139
Benign	KIAA1324	3.33E-198	0.322544067	0.711	0.373	7.08E-194
Benign	CCT3	4.24E-122	0.322092549	0.865	0.625	9.02E-118
Benign	SAMD5	1.00E-195	0.321497735	0.48	0.204	2.13E-191
Benign	SNHG8	6.87E-123	0.321347136	0.916	0.689	1.46E-118
Benign	RPL22L1	2.21E-110	0.319786619	0.808	0.587	4.69E-106
Benign	CFTR	2.93E-143	0.319303951	0.654	0.383	6.23E-139
Benign	RAB32	1.00E-152	0.318883585	0.657	0.366	2.13E-148
Benign	EIF3I	1.99E-115	0.31851988	0.904	0.688	4.24E-111
Benign	MSX2	7.27E-169	0.318296856	0.301	0.096	1.55E-164
Benign	PMPCB	2.60E-144	0.318166604	0.705	0.453	5.53E-140
Benign	BSPRY	3.48E-163	0.317422255	0.656	0.377	7.39E-159
Benign	UFC1	2.30E-119	0.317420337	0.937	0.757	4.89E-115
Benign	ZBTB20	9.82E-136	0.316903927	0.626	0.362	2.09E-131
Benign	CD46	1.78E-120	0.316812333	0.902	0.689	3.78E-116
Benign	STMP1	3.50E-133	0.316239304	0.821	0.592	7.43E-129
Benign	TDGF1	9.02E-184	0.316082676	0.524	0.235	1.92E-179

Benign	CUTA	3.27E-112	0.315518576	0.956	0.76	6.95E-108
Benign	RPS25	1.29E-154	0.314970618	1	0.939	2.75E-150
Benign	TXNL1	4.80E-136	0.314618868	0.792	0.549	1.02E-131
Benign	CCDC88B	2.61E-177	0.314395588	0.543	0.265	5.55E-173
Benign	EIF3K	3.38E-97	0.313202419	0.982	0.82	7.20E-93
Benign	MPG	8.01E-137	0.312124151	0.757	0.514	1.70E-132
Benign	PLEKHA5	2.29E-133	0.311471463	0.58	0.342	4.87E-129
Benign	CERS6	2.46E-142	0.311308903	0.748	0.489	5.22E-138
Benign	EEPD1	2.30E-246	0.310353781	0.435	0.142	4.90E-242
Benign	SLC6A6	1.12E-203	0.307516342	0.434	0.16	2.39E-199
Benign	PFDN5	7.77E-141	0.306945348	0.991	0.907	1.65E-136
Benign	CXXC5	1.65E-115	0.306762023	0.653	0.421	3.51E-111
Benign	RPL37	5.53E-106	0.304778607	1	0.936	1.18E-101
Benign	RPL37A	2.26E-116	0.30366504	1	0.957	4.81E-112
Benign	SARAF	5.44E-117	0.301783763	0.911	0.744	1.16E-112
Benign	SNHG7	5.60E-113	0.301086052	0.74	0.503	1.19E-108
Benign	MET	1.25E-153	0.300242365	0.523	0.267	2.65E-149
Benign	TKT	2.86E-91	0.300206257	0.914	0.695	6.08E-87
Benign	JUN	3.60E-56	0.299468866	0.987	0.919	7.65E-52
Benign	ACAA2	2.36E-111	0.297998292	0.764	0.537	5.02E-107
Benign	NOXO1	2.62E-151	0.297856768	0.631	0.346	5.58E-147
Benign	UBE2H	7.56E-131	0.297563595	0.607	0.367	1.61E-126
Benign	DACH1	1.89E-159	0.296358543	0.458	0.207	4.01E-155
Benign	NPM1	5.03E-128	0.295934143	0.997	0.843	1.07E-123
Benign	CLNS1A	1.15E-115	0.295829037	0.875	0.625	2.44E-111
Benign	MARCKSL1	2.09E-117	0.294556175	0.988	0.844	4.44E-113
Benign	CNBP	4.65E-108	0.294020909	0.956	0.756	9.90E-104
Benign	MACROD1	4.57E-124	0.29329102	0.714	0.455	9.72E-120
Benign	SESN1	1.19E-148	0.293241856	0.464	0.218	2.52E-144
Benign	PTK7	9.75E-249	0.293130652	0.422	0.131	2.07E-244
Benign	PHLDA1	1.68E-208	0.292482753	0.611	0.267	3.57E-204
Benign	ZNF503	7.48E-174	0.291555764	0.412	0.162	1.59E-169
Benign	PHB2	1.87E-105	0.290066861	0.808	0.582	3.97E-101
Benign	UBXN1	4.73E-107	0.289074468	0.873	0.679	1.00E-102
Benign	APP	3.88E-100	0.287199392	0.966	0.831	8.25E-96
Benign	RPL38	1.04E-138	0.286498674	0.999	0.952	2.21E-134
Benign	CIRBP	9.85E-71	0.285329879	0.882	0.74	2.09E-66
Benign	ATP1A1	3.64E-86	0.285076128	0.976	0.803	7.74E-82
Benign	RBMX	9.55E-107	0.284084407	0.857	0.635	2.03E-102
Benign	DSTN	1.54E-116	0.283520902	0.994	0.898	3.28E-112
Benign	BUD23	1.78E-105	0.282209543	0.806	0.585	3.78E-101
Benign	PHF14	3.49E-129	0.28176932	0.792	0.546	7.43E-125
Benign	APRT	1.14E-80	0.28145322	0.969	0.767	2.43E-76

Benign	HSPA8	1.78E-101	0.281431631	0.967	0.809	3.79E-97
Benign	PCM1	6.00E-133	0.280736488	0.673	0.419	1.28E-128
Benign	NHP2	8.60E-97	0.279880737	0.92	0.689	1.83E-92
Benign	C7orf50	5.84E-108	0.279816172	0.729	0.489	1.24E-103
Benign	UQCRC2	6.65E-93	0.279538055	0.925	0.727	1.41E-88
Benign	SERBP1	2.06E-93	0.278492565	0.969	0.796	4.39E-89
Benign	HOXA7	1.21E-134	0.278299327	0.477	0.247	2.57E-130
Benign	GDI2	9.12E-93	0.276856548	0.903	0.704	1.94E-88
Benign	CDKN1B	1.37E-118	0.276706777	0.559	0.328	2.91E-114
Benign	NME4	7.03E-109	0.276042465	0.705	0.447	1.50E-104
Benign	CCL20	9.55E-55	0.2750002	0.349	0.198	2.03E-50
Benign	CTSB	7.45E-96	0.27463678	0.812	0.615	1.58E-91
Benign	KMT2E	2.46E-94	0.274201622	0.851	0.666	5.24E-90
Benign	MUC3A	8.85E-102	0.272792687	0.708	0.486	1.88E-97
Benign	ATRAID	1.92E-98	0.272438058	0.864	0.646	4.08E-94
Benign	GOPC	1.20E-103	0.271901172	0.614	0.402	2.55E-99
Benign	TMEM106B	7.82E-134	0.270308852	0.681	0.43	1.66E-129
Benign	ZNF277	1.03E-134	0.269977019	0.508	0.273	2.18E-130
Benign	ATP6V1F	4.78E-110	0.269697766	0.87	0.641	1.02E-105
Benign	TSPAN12	6.02E-138	0.269123143	0.52	0.27	1.28E-133
Benign	RPS26	1.49E-100	0.268714088	0.998	0.96	3.17E-96
Benign	RAC1	4.53E-94	0.267053548	0.992	0.922	9.63E-90
Benign	ZNF22	3.03E-133	0.266217273	0.651	0.392	6.45E-129
Benign	RPAIN	9.40E-121	0.266211792	0.65	0.409	2.00E-116
Benign	IFITM2	1.04E-123	0.264917804	0.653	0.378	2.22E-119
Benign	AP1S1	4.06E-87	0.264812352	0.848	0.636	8.62E-83
Benign	PABPC1	3.13E-115	0.264708584	0.997	0.888	6.65E-111
Benign	ARID5B	3.08E-105	0.264357899	0.601	0.362	6.55E-101
Benign	FOXQ1	1.32E-171	0.26264413	0.34	0.114	2.81E-167
Benign	ALDH3A2	2.73E-108	0.261313015	0.545	0.329	5.80E-104
Benign	PYURF	2.08E-102	0.260666867	0.861	0.632	4.43E-98
Benign	CAT	1.62E-109	0.260326183	0.681	0.448	3.45E-105
Benign	HIPK2	2.63E-121	0.259698951	0.524	0.291	5.59E-117
Benign	SYF2	1.39E-109	0.259519295	0.899	0.711	2.96E-105
Benign	NPM3	4.38E-107	0.259481286	0.605	0.37	9.32E-103
Benign	PSMB6	6.14E-72	0.259034151	0.86	0.68	1.31E-67
Benign	SEM1	1.05E-84	0.259004873	0.974	0.861	2.24E-80
Benign	NFIA	4.89E-103	0.258874515	0.722	0.485	1.04E-98
Benign	UBA52	9.99E-107	0.258188109	0.998	0.94	2.12E-102
Benign	TBPL1	4.97E-133	0.257592854	0.573	0.333	1.06E-128
Benign	HTATIP2	1.48E-92	0.256442965	0.805	0.605	3.15E-88
Benign	STXBP6	3.18E-113	0.256060894	0.687	0.433	6.76E-109
Benign	YBX3	7.91E-80	0.255091562	0.873	0.68	1.68E-75

Benign	CAPZA2	1.45E-97	0.255075068	0.844	0.65	3.08E-93
Benign	CAMLG	2.85E-103	0.254782215	0.704	0.475	6.06E-99
Benign	PDCD2	2.69E-103	0.25477902	0.737	0.506	5.71E-99
Benign	GGH	1.15E-65	0.254547956	0.732	0.526	2.45E-61
Benign	DNAJC15	1.25E-64	0.253961152	0.906	0.758	2.67E-60
Benign	TRAPPC6A	1.85E-111	0.253643734	0.714	0.469	3.93E-107
Benign	RNF186	4.04E-110	0.252884914	0.642	0.382	8.59E-106
Benign	TMEM63A	9.48E-114	0.252793463	0.717	0.47	2.02E-109
Benign	ABHD14B	1.28E-91	0.252636625	0.684	0.469	2.73E-87
Benign	RPS15	1.64E-128	0.252504358	1	0.971	3.49E-124
Benign	MRPL32	3.08E-95	0.252109895	0.791	0.565	6.56E-91
Benign	CRLS1	4.46E-105	0.251917381	0.712	0.488	9.47E-101
Benign	BIK	6.05E-100	0.251600837	0.654	0.427	1.29E-95
Benign	NTRK2	5.59E-148	0.251597863	0.266	0.08	1.19E-143
Benign	AC020916.1	1.85E-76	0.251594167	0.553	0.361	3.93E-72
Benign	SNU13	4.05E-81	0.250969076	0.887	0.687	8.61E-77
Benign	NFE2L3	5.96E-116	0.250777762	0.52	0.286	1.27E-111
Benign	SMIM19	7.95E-105	0.250678427	0.7	0.471	1.69E-100
Benign	ID2	6.77E-69	0.250023956	0.843	0.673	1.44E-64
Enterocyte	GUCA2A	0	5.077523685	0.518	0.136	0
Enterocyte	GUCA2B	0	4.463127433	0.389	0.065	0
Enterocyte	CA4	0	3.692852004	0.434	0.027	0
Enterocyte	MT1G	0	3.066480446	0.75	0.348	0
Enterocyte	CA2	0	2.986185717	0.831	0.238	0
Enterocyte	FABP1	0	2.955386584	0.824	0.477	0
Enterocyte	SLC26A3	0	2.832510153	0.38	0.039	0
Enterocyte	CLCA4	0	2.680914831	0.256	0.017	0
Enterocyte	CA1	0	2.468374913	0.372	0.022	0
Enterocyte	CEACAM7	0	2.444237079	0.405	0.061	0
Enterocyte	MT2A	0	2.367576631	0.813	0.512	0
Enterocyte	SLC26A2	0	2.260103306	0.66	0.2	0
Enterocyte	MT1H	0	2.193652415	0.401	0.06	0
Enterocyte	PHGR1	0	2.113667711	0.993	0.923	0
Enterocyte	SELENOP	3.69E-173	1.999708454	0.585	0.353	7.85E-169
Enterocyte	B3GNT7	0	1.98968147	0.575	0.155	0
Enterocyte	C15orf48	0	1.914497764	0.961	0.796	0
Enterocyte	MT1E	0	1.816490348	0.827	0.462	0
Enterocyte	ITM2C	0	1.72334966	0.963	0.816	0
Enterocyte	LYPD8	1.75E-188	1.698788006	0.353	0.126	3.72E-184
Enterocyte	ADH1C	0	1.693848715	0.691	0.328	0
Enterocyte	PIGR	0	1.69357294	0.946	0.638	0
Enterocyte	PLA2G2A	1.15E-134	1.635974717	0.581	0.389	2.45E-130
Enterocyte	FTH1	0	1.579309539	0.998	0.991	0

Enterocyte	MT1M	0	1.563209251	0.61	0.19	0
Enterocyte	MT1F	0	1.551366737	0.585	0.242	0
Enterocyte	MUC12	0	1.540626195	0.831	0.555	0
Enterocyte	MT1X	4.61E-196	1.458555468	0.714	0.564	9.80E-192
Enterocyte	LGALS2	0	1.452375176	0.621	0.231	0
Enterocyte	TSPAN1	0	1.45090232	0.918	0.694	0
Enterocyte	KRT20	3.41E-59	1.412578344	0.5	0.415	7.25E-55
Enterocyte	C19orf33	0	1.409723203	0.972	0.806	0
Enterocyte	MS4A12	0	1.357880433	0.317	0.021	0
Enterocyte	SDCBP2	0	1.34637649	0.647	0.274	0
Enterocyte	CDHR5	0	1.333134411	0.74	0.295	0
Enterocyte	PCK1	0	1.311349565	0.646	0.232	0
Enterocyte	PKIB	0	1.305419389	0.497	0.172	0
Enterocyte	AOC1	0	1.242046348	0.787	0.426	0
Enterocyte	HPGD	1.27E-307	1.233178014	0.5	0.175	2.70E-303
Enterocyte	CES2	0	1.228245186	0.815	0.449	0
Enterocyte	PLAC8	1.20E-162	1.22554493	0.607	0.365	2.55E-158
Enterocyte	FXYD3	0	1.211962961	0.99	0.964	0
Enterocyte	UGT2B17	0	1.190411813	0.519	0.145	0
Enterocyte	SRI	0	1.183899802	0.966	0.905	0
Enterocyte	GPA33	6.73E-254	1.093482336	0.723	0.48	1.43E-249
Enterocyte	VSIG2	0	1.09320676	0.81	0.528	0
Enterocyte	LGALS4	0	1.090410976	0.971	0.961	0
Enterocyte	KRT19	4.17E-270	1.077820563	0.943	0.902	8.87E-266
Enterocyte	CEACAM1	1.54E-15	1.072074686	0.358	0.335	3.28E-11
Enterocyte	HIST1H1C	9.71E-67	1.060058786	0.46	0.338	2.06E-62
Enterocyte	FKBP1A	5.34E-222	1.042707314	0.869	0.766	1.13E-217
Enterocyte	C10orf99	0	1.031935331	0.86	0.596	0
Enterocyte	LINC01133	2.03E-143	1.019528323	0.53	0.32	4.31E-139
Enterocyte	PRDX6	7.43E-298	1.018583507	0.843	0.741	1.58E-293
Enterocyte	B2M	3.45E-249	1.00723242	1	0.996	7.34E-245
Enterocyte	CHP2	0	1.006279276	0.632	0.22	0
Enterocyte	CDKN2B-AS1	0	0.992394933	0.391	0.039	0
Enterocyte	ETHE1	5.15E-244	0.968886451	0.811	0.753	1.10E-239
Enterocyte	DHRS11	0	0.967093852	0.613	0.257	0
Enterocyte	TMEM54	3.82E-307	0.948453342	0.925	0.841	8.12E-303
Enterocyte	LGALS3	0	0.939103362	0.988	0.956	0
Enterocyte	SPINK5	0	0.924244478	0.377	0.065	0
Enterocyte	SLC20A1	1.20E-167	0.915281053	0.468	0.252	2.56E-163
Enterocyte	CYSTM1	0	0.913144976	0.943	0.856	0
Enterocyte	CTSS	0	0.911595819	0.881	0.738	0
Enterocyte	C2orf88	0	0.897700486	0.37	0.081	0
Enterocyte	SLPI	1.51E-150	0.893003919	0.693	0.544	3.22E-146

Enterocyte	CKB	4.22E-136	0.878532489	0.882	0.773	8.97E-132
Enterocyte	AMN	0	0.870994009	0.765	0.483	0
Enterocyte	PLA2G10	0	0.858228693	0.604	0.239	0
Enterocyte	SULT1B1	0	0.84798793	0.616	0.28	0
Enterocyte	S100A14	1.03E-231	0.838121373	0.831	0.778	2.19E-227
Enterocyte	PPP1R14D	3.90E-241	0.836623078	0.708	0.47	8.28E-237
Enterocyte	ISG15	0.007705926	0.830022113	0.375	0.468	1
Enterocyte	CRIP1	7.89E-138	0.827509104	0.639	0.435	1.68E-133
Enterocyte	JUNB	3.82E-242	0.821795458	0.904	0.839	8.11E-238
Enterocyte	CCL15	1.29E-259	0.812671467	0.632	0.361	2.74E-255
Enterocyte	PADI2	0	0.810333127	0.544	0.133	0
Enterocyte	MT-ND4L	6.00E-218	0.808568737	0.921	0.864	1.27E-213
Enterocyte	MALL	4.22E-116	0.806369333	0.497	0.298	8.97E-112
Enterocyte	HMGCS2	5.33E-179	0.797174208	0.633	0.408	1.13E-174
Enterocyte	TST	1.17E-236	0.794582432	0.717	0.528	2.50E-232
Enterocyte	MT-CO1	0	0.792876024	0.996	0.993	0
Enterocyte	PDCD4	2.70E-267	0.791163038	0.772	0.596	5.73E-263
Enterocyte	HSD17B2	0	0.787456135	0.421	0.101	0
Enterocyte	DUSP1	1.02E-183	0.783820876	0.808	0.662	2.16E-179
Enterocyte	VAMP8	1.80E-218	0.783664909	0.914	0.861	3.83E-214
Enterocyte	GOLM1	2.99E-252	0.782545065	0.803	0.693	6.35E-248
Enterocyte	PRSS3	9.82E-243	0.780969551	0.904	0.709	2.09E-238
Enterocyte	CD177	2.20E-264	0.766329672	0.311	0.059	4.67E-260
Enterocyte	SLC4A4	0	0.765312844	0.477	0.08	0
Enterocyte	KRT8	1.59E-277	0.762857865	0.994	0.984	3.39E-273
Enterocyte	CLDN23	1.79E-163	0.755444695	0.334	0.125	3.81E-159
Enterocyte	GRN	7.33E-284	0.754725258	0.869	0.794	1.56E-279
Enterocyte	TRIM31	1.88E-07	0.753970093	0.378	0.376	0.003994423
Enterocyte	FABP5	8.05E-117	0.752838942	0.723	0.571	1.71E-112
Enterocyte	CDHR2	8.31E-167	0.748544621	0.266	0.075	1.77E-162
Enterocyte	NXPE4	4.31E-294	0.745999678	0.535	0.216	9.16E-290
Enterocyte	BLOC1S1	1.60E-254	0.728984018	0.912	0.859	3.39E-250
Enterocyte	CTSZ	6.27E-162	0.724520375	0.689	0.521	1.33E-157
Enterocyte	MT-ND5	6.87E-292	0.723879528	0.973	0.97	1.46E-287
Enterocyte	CA12	1.05E-220	0.721302861	0.652	0.425	2.23E-216
Enterocyte	CLDN7	2.50E-263	0.720365108	0.948	0.89	5.31E-259
Enterocyte	UGP2	7.78E-165	0.716831411	0.78	0.686	1.65E-160
Enterocyte	CFDP1	6.80E-33	0.71650742	0.728	0.705	1.45E-28
Enterocyte	KLF6	6.76E-137	0.715032241	0.866	0.802	1.44E-132
Enterocyte	AC008397.1	2.03E-287	0.713896701	0.514	0.204	4.31E-283
Enterocyte	RHOC	1.69E-60	0.712028563	0.864	0.811	3.59E-56
Enterocyte	S100A10	0	0.709440654	0.992	0.971	0
Enterocyte	CHCHD10	5.83E-169	0.707421554	0.825	0.835	1.24E-164

Enterocyte	TMEM59	2.02E-229	0.695107309	0.944	0.926	4.30E-225
Enterocyte	SAMD9	3.40E-62	0.688770142	0.264	0.142	7.23E-58
Enterocyte	SMPDL3A	5.60E-67	0.675169672	0.416	0.282	1.19E-62
Enterocyte	SULT1A1	2.68E-192	0.668712833	0.48	0.246	5.69E-188
Enterocyte	CTSD	3.85E-161	0.667856245	0.872	0.803	8.19E-157
Enterocyte	IER2	1.05E-192	0.66601727	0.897	0.84	2.24E-188
Enterocyte	ARL14	1.55E-76	0.663587416	0.417	0.272	3.30E-72
Enterocyte	HOXB13	2.81E-209	0.663218775	0.459	0.21	5.97E-205
Enterocyte	PTP4A1	6.28E-185	0.654486816	0.736	0.606	1.34E-180
Enterocyte	MISP	1.95E-99	0.651141245	0.76	0.63	4.16E-95
Enterocyte	CTSA	4.25E-100	0.647772208	0.66	0.548	9.04E-96
Enterocyte	SERINC2	5.75E-112	0.646733621	0.841	0.773	1.22E-107
Enterocyte	HIGD1A	3.28E-133	0.643886511	0.74	0.651	6.96E-129
Enterocyte	STAP2	4.84E-167	0.638503237	0.721	0.595	1.03E-162
Enterocyte	SLC44A4	4.43E-209	0.628536823	0.841	0.72	9.43E-205
Enterocyte	FABP2	5.59E-169	0.625604937	0.528	0.275	1.19E-164
Enterocyte	CDX1	3.76E-190	0.622954342	0.811	0.738	8.00E-186
Enterocyte	MEP1A	1.79E-243	0.620537623	0.364	0.106	3.80E-239
Enterocyte	JUND	1.21E-151	0.620120825	0.819	0.695	2.57E-147
Enterocyte	ZFP36	1.82E-118	0.611740987	0.836	0.768	3.87E-114
Enterocyte	FAM162A	9.53E-129	0.610651138	0.784	0.79	2.03E-124
Enterocyte	DST	7.97E-14	0.607874037	0.563	0.561	1.69E-09
Enterocyte	KLF4	9.68E-159	0.605213442	0.752	0.589	2.06E-154
Enterocyte	CCL28	1.42E-217	0.604758121	0.57	0.303	3.03E-213
Enterocyte	B3GALT5	0	0.602189862	0.394	0.077	0
Enterocyte	MAOA	4.04E-165	0.601918909	0.607	0.424	8.58E-161
Enterocyte	RNASE1	4.99E-25	0.600575263	0.493	0.393	1.06E-20
Enterocyte	FUCA1	6.43E-155	0.59880117	0.67	0.507	1.37E-150
Enterocyte	IL32	1.01E-29	0.596230122	0.569	0.753	2.14E-25
Enterocyte	CAMK2N1	4.01E-181	0.595532225	0.875	0.757	8.53E-177
Enterocyte	MIDN	1.07E-103	0.594466148	0.668	0.555	2.27E-99
Enterocyte	BSG	1.06E-132	0.593410976	0.917	0.869	2.25E-128
Enterocyte	HLA-B	7.35E-72	0.591722299	0.982	0.976	1.56E-67
Enterocyte	TDP2	4.71E-76	0.590326907	0.688	0.612	1.00E-71
Enterocyte	MT-ND3	1.28E-229	0.589888201	0.988	0.985	2.72E-225
Enterocyte	MVP	4.14E-140	0.589300714	0.758	0.648	8.80E-136
Enterocyte	LAMTOR4	8.01E-194	0.583927594	0.88	0.833	1.70E-189
Enterocyte	FRMD1	1.19E-130	0.580822908	0.293	0.114	2.54E-126
Enterocyte	GIPC1	9.05E-120	0.579638495	0.701	0.594	1.93E-115
Enterocyte	SLC22A18	2.42E-153	0.576474361	0.645	0.458	5.15E-149
Enterocyte	SLC39A5	8.79E-209	0.574458916	0.583	0.336	1.87E-204
Enterocyte	TSPAN3	1.56E-131	0.572209749	0.811	0.7	3.32E-127
Enterocyte	TMEM176B	3.28E-142	0.572101181	0.831	0.7	6.98E-138

Enterocyte	MRPL41	1.27E-127	0.569492539	0.785	0.766	2.71E-123
Enterocyte	HSD11B2	1.34E-102	0.567208064	0.565	0.429	2.85E-98
Enterocyte	MXD1	2.22E-96	0.565663708	0.446	0.273	4.73E-92
Enterocyte	MYL6	1.71E-235	0.563651422	0.99	0.98	3.63E-231
Enterocyte	SLC22A18AS	5.11E-281	0.563613275	0.438	0.144	1.09E-276
Enterocyte	ATP8B1	8.18E-148	0.560097769	0.69	0.564	1.74E-143
Enterocyte	RARRES2	2.32E-121	0.559552231	0.359	0.164	4.94E-117
Enterocyte	CLTB	2.13E-138	0.5571738	0.88	0.817	4.54E-134
Enterocyte	SPINT1	5.71E-82	0.555105538	0.771	0.687	1.21E-77
Enterocyte	CIB1	1.28E-131	0.554835863	0.875	0.806	2.71E-127
Enterocyte	PRAC1	1.32E-92	0.554338917	0.551	0.348	2.81E-88
Enterocyte	MIR194-2HG	3.91E-112	0.552447635	0.444	0.259	8.31E-108
Enterocyte	SELENOS	4.66E-141	0.548576992	0.805	0.725	9.90E-137
Enterocyte	SCIN	3.39E-123	0.546376214	0.322	0.135	7.22E-119
Enterocyte	TP53INP2	2.63E-144	0.541808217	0.307	0.11	5.60E-140
Enterocyte	CMBL	1.76E-118	0.541384677	0.672	0.582	3.74E-114
Enterocyte	TMPRSS2	9.06E-84	0.539825289	0.625	0.503	1.93E-79
Enterocyte	ENTPD5	4.25E-173	0.538970739	0.515	0.294	9.03E-169
Enterocyte	EGR1	8.17E-55	0.538737114	0.696	0.666	1.74E-50
Enterocyte	GNA11	8.00E-128	0.534863735	0.495	0.312	1.70E-123
Enterocyte	APOBR	7.99E-182	0.534083783	0.481	0.232	1.70E-177
Enterocyte	COX5B	1.08E-244	0.531833287	0.953	0.956	2.30E-240
Enterocyte	HSD17B11	3.79E-84	0.529114402	0.796	0.719	8.07E-80
Enterocyte	MUC13	1.47E-100	0.528451068	0.802	0.71	3.13E-96
Enterocyte	RIOK3	3.78E-77	0.528450697	0.565	0.443	8.03E-73
Enterocyte	PARM1	3.68E-131	0.526579006	0.578	0.389	7.83E-127
Enterocyte	CHMP2A	1.90E-111	0.525536335	0.85	0.805	4.04E-107
Enterocyte	TJP3	7.05E-88	0.523478462	0.534	0.392	1.50E-83
Enterocyte	HSPB1	5.25E-33	0.52316692	0.804	0.763	1.12E-28
Enterocyte	CISD3	5.17E-135	0.523149167	0.754	0.706	1.10E-130
Enterocyte	SATB2	1.24E-147	0.52111558	0.625	0.426	2.64E-143
Enterocyte	SFN	1.20E-51	0.51866449	0.694	0.602	2.55E-47
Enterocyte	HLA-C	1.98E-67	0.518594303	0.977	0.968	4.21E-63
Enterocyte	UQCR10	5.36E-139	0.516363847	0.877	0.896	1.14E-134
Enterocyte	AGPAT2	1.28E-52	0.515832878	0.729	0.684	2.71E-48
Enterocyte	SMIM22	2.96E-180	0.51299084	0.952	0.946	6.30E-176
Enterocyte	HK2	2.28E-165	0.512578763	0.498	0.261	4.84E-161
Enterocyte	UQCR11	2.01E-175	0.508223562	0.931	0.923	4.27E-171
Enterocyte	NFKBIA	1.25E-41	0.502787555	0.668	0.598	2.66E-37
Enterocyte	TAX1BP3	5.30E-49	0.499741899	0.702	0.633	1.13E-44
Enterocyte	GGT6	3.93E-123	0.497699621	0.578	0.411	8.35E-119
Enterocyte	GCNT3	2.77E-68	0.496916017	0.344	0.197	5.88E-64
Enterocyte	NANS	6.16E-77	0.496578107	0.73	0.736	1.31E-72

Enterocyte	EFNA1	7.23E-46	0.493269707	0.521	0.433	1.54E-41
Enterocyte	LGALS3BP	1.06E-138	0.492229595	0.872	0.795	2.25E-134
Enterocyte	TMEM45B	5.52E-88	0.489056907	0.701	0.567	1.17E-83
Enterocyte	CDKN2B	5.07E-213	0.488251201	0.32	0.088	1.08E-208
Enterocyte	AGR3	5.62E-102	0.486549819	0.833	0.791	1.19E-97
Enterocyte	EPCAM	5.76E-143	0.486403534	0.99	0.981	1.22E-138
Enterocyte	SPPL2A	4.90E-96	0.486046199	0.592	0.463	1.04E-91
Enterocyte	TNFRSF1A	9.32E-54	0.484823546	0.508	0.409	1.98E-49
Enterocyte	NDUFA1	1.45E-155	0.484202229	0.902	0.924	3.09E-151
Enterocyte	PRAP1	4.19E-09	0.483554315	0.266	0.365	8.92E-05
Enterocyte	SQOR	5.07E-101	0.482695258	0.672	0.593	1.08E-96
Enterocyte	ACADS	8.50E-96	0.482379401	0.592	0.474	1.81E-91
Enterocyte	NDUFB1	1.46E-116	0.481999913	0.86	0.868	3.11E-112
Enterocyte	NXPE1	8.68E-241	0.481775733	0.422	0.145	1.85E-236
Enterocyte	GPT	8.84E-275	0.480043164	0.405	0.125	1.88E-270
Enterocyte	UPP1	2.27E-55	0.479745884	0.298	0.183	4.83E-51
Enterocyte	STARD10	1.34E-88	0.478745436	0.776	0.828	2.86E-84
Enterocyte	RETSAT	3.43E-115	0.475426772	0.45	0.276	7.29E-111
Enterocyte	TNFSF10	3.04E-27	0.473982839	0.623	0.583	6.46E-23
Enterocyte	MT-CO3	9.09E-225	0.472898396	0.997	0.994	1.93E-220
Enterocyte	DSC2	2.69E-60	0.471930036	0.683	0.615	5.73E-56
Enterocyte	SLC9A3R1	5.50E-28	0.470742012	0.384	0.32	1.17E-23
Enterocyte	MYL12B	6.40E-92	0.469786559	0.952	0.935	1.36E-87
Enterocyte	C1orf21	3.53E-75	0.465421765	0.59	0.463	7.51E-71
Enterocyte	CYP3A5	7.32E-46	0.46510261	0.651	0.583	1.56E-41
Enterocyte	IL2RG	2.06E-48	0.464893973	0.334	0.221	4.39E-44
Enterocyte	MALAT1	1.22E-92	0.462007755	0.995	0.994	2.60E-88
Enterocyte	ACOX1	5.91E-44	0.460872126	0.436	0.356	1.26E-39
Enterocyte	LITAF	4.97E-58	0.459565118	0.708	0.655	1.06E-53
Enterocyte	ACVRL1	3.49E-142	0.459546973	0.451	0.241	7.42E-138
Enterocyte	RSRP1	6.31E-85	0.458764253	0.724	0.626	1.34E-80
Enterocyte	NDUFB7	2.75E-131	0.455233764	0.842	0.841	5.85E-127
Enterocyte	LRRC19	5.28E-150	0.454249345	0.385	0.177	1.12E-145
Enterocyte	TLCD2	4.84E-178	0.452704223	0.415	0.182	1.03E-173
Enterocyte	MT-ND1	2.36E-161	0.449924734	0.988	0.983	5.02E-157
Enterocyte	EDN3	3.56E-274	0.447698502	0.278	0.048	7.57E-270
Enterocyte	CHP1	7.27E-58	0.446511891	0.704	0.672	1.54E-53
Enterocyte	AVPI1	2.58E-75	0.446270196	0.486	0.355	5.49E-71
Enterocyte	PRSS8	7.84E-68	0.445974206	0.698	0.597	1.67E-63
Enterocyte	CDH17	1.83E-122	0.445290093	0.843	0.76	3.90E-118
Enterocyte	AHCYL2	2.99E-139	0.442079373	0.373	0.179	6.37E-135
Enterocyte	SCP2	3.01E-116	0.440697721	0.83	0.828	6.40E-112
Enterocyte	PFN1	2.52E-166	0.439408147	0.95	0.943	5.36E-162

Enterocyte	ITM2B	2.13E-108	0.436516003	0.915	0.91	4.52E-104
Enterocyte	UBB	6.65E-57	0.43640657	0.97	0.956	1.41E-52
Enterocyte	PAG1	1.22E-119	0.436400875	0.334	0.15	2.60E-115
Enterocyte	NDUFA13	5.75E-133	0.435071492	0.891	0.88	1.22E-128
Enterocyte	PLCE1	1.93E-195	0.434020602	0.412	0.172	4.10E-191
Enterocyte	TENT5A	3.74E-47	0.43294162	0.459	0.354	7.94E-43
Enterocyte	LIMA1	6.90E-103	0.432081454	0.804	0.777	1.47E-98
Enterocyte	FOS	2.25E-107	0.43187687	0.934	0.905	4.79E-103
Enterocyte	CCDC68	3.15E-113	0.430955935	0.371	0.183	6.71E-109
Enterocyte	CLDN3	6.36E-96	0.430336103	0.972	0.942	1.35E-91
Enterocyte	CD9	1.67E-112	0.429386498	0.93	0.888	3.55E-108
Enterocyte	POLD4	7.06E-55	0.429190623	0.545	0.445	1.50E-50
Enterocyte	PAPSS2	1.42E-96	0.428854868	0.478	0.323	3.02E-92
Enterocyte	NFKBIZ	4.53E-48	0.427082408	0.498	0.406	9.63E-44
Enterocyte	MYH14	8.33E-39	0.427079782	0.639	0.589	1.77E-34
Enterocyte	SLC44A1	2.43E-90	0.426165091	0.742	0.696	5.17E-86
Enterocyte	DEFB1	2.34E-165	0.42572681	0.268	0.079	4.99E-161
Enterocyte	CKMT1A	1.60E-94	0.42518545	0.533	0.372	3.41E-90
Enterocyte	CEACAM6	1.26E-05	0.423441347	0.485	0.469	0.267160122
Enterocyte	COX6A1	5.62E-157	0.423124335	0.945	0.94	1.20E-152
Enterocyte	RRBP1	3.03E-78	0.421907236	0.875	0.827	6.44E-74
Enterocyte	ANKRD12	4.21E-25	0.420904035	0.573	0.535	8.95E-21
Enterocyte	CCNL1	2.50E-72	0.42083079	0.77	0.721	5.32E-68
Enterocyte	PRKACB	2.30E-124	0.420280837	0.513	0.324	4.89E-120
Enterocyte	RBM47	4.18E-71	0.417908167	0.7	0.662	8.88E-67
Enterocyte	COX7B	9.62E-111	0.417783179	0.911	0.931	2.05E-106
Enterocyte	FOSB	8.49E-49	0.41770351	0.719	0.692	1.81E-44
Enterocyte	HHLA2	4.78E-94	0.416394406	0.252	0.106	1.02E-89
Enterocyte	TFCP2L1	1.39E-130	0.416173267	0.453	0.249	2.95E-126
Enterocyte	MIR22HG	3.25E-202	0.415720845	0.329	0.099	6.90E-198
Enterocyte	HLA-A	1.39E-35	0.415485091	0.984	0.978	2.96E-31
Enterocyte	ATP1B3	1.13E-05	0.415477256	0.716	0.724	0.239952074
Enterocyte	CDKN1A	4.44E-30	0.415300974	0.447	0.372	9.44E-26
Enterocyte	ACADVL	9.82E-98	0.413975448	0.785	0.696	2.09E-93
Enterocyte	GNPTAB	4.12E-73	0.413790148	0.501	0.378	8.76E-69
Enterocyte	CARD16	6.03E-52	0.412061637	0.486	0.358	1.28E-47
Enterocyte	ABCC3	3.44E-63	0.411692908	0.661	0.575	7.32E-59
Enterocyte	UGT2A3	1.25E-227	0.411403127	0.378	0.121	2.65E-223
Enterocyte	UGDH	3.01E-68	0.411251657	0.573	0.464	6.41E-64
Enterocyte	CAPN2	9.32E-60	0.408258321	0.616	0.513	1.98E-55
Enterocyte	UBC	2.72E-55	0.406843472	0.957	0.941	5.78E-51
Enterocyte	NDUFS7	1.46E-76	0.406578393	0.734	0.72	3.11E-72
Enterocyte	ATP5MC1	1.12E-44	0.406519933	0.725	0.804	2.39E-40

Enterocyte	TXNIP	5.32E-38	0.405917212	0.755	0.689	1.13E-33
Enterocyte	PLCD3	2.09E-131	0.405062713	0.39	0.197	4.44E-127
Enterocyte	MYO1A	3.99E-147	0.404186237	0.412	0.199	8.49E-143
Enterocyte	CASP7	7.29E-32	0.400572943	0.489	0.433	1.55E-27
Enterocyte	PLS1	2.08E-45	0.400406444	0.618	0.562	4.41E-41
Enterocyte	CPT1A	1.87E-79	0.399944651	0.451	0.316	3.98E-75
Enterocyte	CLDN8	0	0.398892676	0.286	0.034	0
Enterocyte	ATP5F1D	3.08E-92	0.396008392	0.87	0.881	6.55E-88
Enterocyte	IL18	1.69E-76	0.395602171	0.465	0.32	3.60E-72
Enterocyte	CPM	3.19E-226	0.395231386	0.371	0.114	6.79E-222
Enterocyte	ATP5ME	1.52E-94	0.394919135	0.907	0.924	3.24E-90
Enterocyte	PHLDA2	3.30E-53	0.391863061	0.686	0.607	7.02E-49
Enterocyte	FAM3C	2.50E-33	0.3909326	0.523	0.494	5.31E-29
Enterocyte	AKR1C3	1.81E-67	0.390739923	0.568	0.451	3.85E-63
Enterocyte	LINC00483	2.76E-107	0.389869749	0.306	0.139	5.86E-103
Enterocyte	MT-ND4	1.31E-175	0.389642587	0.992	0.991	2.78E-171
Enterocyte	LEFTY1	2.38E-17	0.389086249	0.622	0.599	5.05E-13
Enterocyte	PXMP2	3.33E-72	0.387997451	0.585	0.486	7.08E-68
Enterocyte	AC254629.1	2.53E-93	0.386240426	0.372	0.207	5.39E-89
Enterocyte	TCEA3	3.43E-74	0.382386383	0.654	0.553	7.29E-70
Enterocyte	KLF5	6.89E-80	0.381007092	0.834	0.828	1.47E-75
Enterocyte	ACAA1	2.32E-42	0.380928315	0.582	0.514	4.93E-38
Enterocyte	FCGRT	2.01E-55	0.380764984	0.793	0.743	4.26E-51
Enterocyte	SUCLG2	6.94E-57	0.3804104	0.699	0.706	1.48E-52
Enterocyte	ATP5MPL	2.25E-68	0.3790655	0.877	0.911	4.78E-64
Enterocyte	MT-ND2	2.31E-129	0.378600644	0.993	0.989	4.91E-125
Enterocyte	PINK1	8.54E-57	0.37853474	0.406	0.286	1.82E-52
Enterocyte	PI3	3.41E-37	0.378268488	0.289	0.181	7.24E-33
Enterocyte	LRP10	1.18E-36	0.377924189	0.673	0.62	2.51E-32
Enterocyte	MT-CO2	1.01E-146	0.377905041	0.995	0.992	2.14E-142
Enterocyte	ARF6	2.77E-77	0.377088653	0.739	0.677	5.89E-73
Enterocyte	SMCHD1	8.84E-18	0.376738462	0.383	0.343	1.88E-13
Enterocyte	MGAT4B	1.18E-50	0.376527582	0.617	0.547	2.51E-46
Enterocyte	CHMP4B	5.23E-41	0.375960851	0.864	0.831	1.11E-36
Enterocyte	MARCKS	3.11E-83	0.375873263	0.824	0.809	6.61E-79
Enterocyte	PDLIM1	1.68E-72	0.375386103	0.854	0.82	3.57E-68
Enterocyte	RNF186	5.92E-56	0.373066254	0.533	0.412	1.26E-51
Enterocyte	CD63	3.89E-134	0.372869332	0.972	0.961	8.26E-130
Enterocyte	ALKBH7	8.10E-72	0.372469521	0.76	0.691	1.72E-67
Enterocyte	CITED2	2.77E-66	0.371718479	0.391	0.258	5.90E-62
Enterocyte	NEAT1	2.94E-81	0.371448653	0.957	0.949	6.26E-77
Enterocyte	MPST	1.18E-68	0.371397682	0.839	0.796	2.52E-64
Enterocyte	PYCARD	2.19E-45	0.371169673	0.675	0.669	4.67E-41

Enterocyte	HSBP1L1	8.32E-79	0.370994931	0.57	0.447	1.77E-74
Enterocyte	PLK3	2.65E-78	0.370399592	0.312	0.169	5.63E-74
Enterocyte	ESPN	8.76E-85	0.368558838	0.281	0.137	1.86E-80
Enterocyte	VSIR	4.04E-56	0.368470322	0.265	0.151	8.59E-52
Enterocyte	MAX	0.001982059	0.368293099	0.358	0.37	1
Enterocyte	MGST3	1.01E-41	0.367829143	0.783	0.818	2.14E-37
Enterocyte	RAB11FIP1	1.78E-72	0.367596648	0.776	0.698	3.78E-68
Enterocyte	VAPA	1.08E-51	0.366982603	0.793	0.748	2.31E-47
Enterocyte	LAMB3	3.39E-18	0.366547454	0.406	0.355	7.21E-14
Enterocyte	AKR1B10	1.44E-72	0.366272827	0.289	0.15	3.06E-68
Enterocyte	MYL12A	5.55E-32	0.36572475	0.95	0.93	1.18E-27
Enterocyte	USP53	3.20E-63	0.365574726	0.571	0.454	6.81E-59
Enterocyte	ZBTB7A	1.89E-58	0.36553305	0.648	0.585	4.01E-54
Enterocyte	KLF3	1.78E-51	0.365518846	0.691	0.646	3.80E-47
Enterocyte	ANXA11	8.30E-63	0.365227671	0.805	0.77	1.76E-58
Enterocyte	FBLIM1	4.53E-40	0.364937117	0.453	0.369	9.64E-36
Enterocyte	LSR	1.34E-53	0.36303045	0.854	0.824	2.84E-49
Enterocyte	COX5A	8.13E-83	0.362511559	0.881	0.903	1.73E-78
Enterocyte	GBP3	1.43E-60	0.361785235	0.382	0.255	3.05E-56
Enterocyte	SERF2	4.44E-158	0.361056291	0.986	0.985	9.44E-154
Enterocyte	SELENOW	6.74E-39	0.361047447	0.825	0.828	1.43E-34
Enterocyte	UQCRC1	1.60E-72	0.359430375	0.798	0.802	3.40E-68
Enterocyte	NEU4	8.00E-173	0.359231473	0.327	0.113	1.70E-168
Enterocyte	SIAE	1.03E-85	0.359016472	0.471	0.326	2.18E-81
Enterocyte	S100A6	3.70E-112	0.357369995	0.998	0.995	7.87E-108
Enterocyte	TMEM98	2.99E-56	0.356432077	0.639	0.581	6.36E-52
Enterocyte	NAAA	2.47E-45	0.356040997	0.344	0.236	5.25E-41
Enterocyte	SLC25A5	1.48E-62	0.354864431	0.9	0.901	3.16E-58
Enterocyte	DOK4	6.29E-50	0.354705545	0.396	0.287	1.34E-45
Enterocyte	GPRC5C	7.73E-38	0.354426123	0.384	0.295	1.64E-33
Enterocyte	RILP	2.47E-80	0.354403049	0.377	0.228	5.25E-76
Enterocyte	METTL7A	1.57E-98	0.353858492	0.394	0.222	3.35E-94
Enterocyte	NR2F6	1.72E-53	0.353492454	0.52	0.423	3.66E-49
Enterocyte	UQCRQ	4.27E-78	0.352446501	0.939	0.931	9.09E-74
Enterocyte	SPINT1-AS1	2.75E-59	0.352248753	0.588	0.5	5.85E-55
Enterocyte	EPHX2	8.87E-94	0.350373882	0.485	0.322	1.89E-89
Enterocyte	LLGL2	4.44E-60	0.35034845	0.697	0.632	9.44E-56
Enterocyte	ASL	2.81E-58	0.350275082	0.648	0.596	5.97E-54
Enterocyte	SEMA6A	1.39E-129	0.347313702	0.37	0.168	2.95E-125
Enterocyte	CLDN4	1.68E-31	0.346405634	0.96	0.939	3.57E-27
Enterocyte	PKP2	9.81E-59	0.346170573	0.586	0.513	2.09E-54
Enterocyte	TMEM171	3.95E-84	0.345747524	0.353	0.201	8.40E-80
Enterocyte	EPS8	2.81E-30	0.345092592	0.638	0.584	5.98E-26

Enterocyte	PEX26	1.85E-17	0.343614907	0.305	0.258	3.94E-13
Enterocyte	SH3KBP1	7.20E-43	0.342621449	0.607	0.535	1.53E-38
Enterocyte	MPC1	4.66E-46	0.342535494	0.637	0.604	9.90E-42
Enterocyte	PRR13	6.03E-50	0.342287321	0.889	0.873	1.28E-45
Enterocyte	AHNAK	1.37E-45	0.341744922	0.586	0.5	2.92E-41
Enterocyte	SI	6.91E-186	0.340637636	0.252	0.062	1.47E-181
Enterocyte	BTG1	1.75E-73	0.340065736	0.816	0.776	3.71E-69
Enterocyte	MUC20-OT1	6.70E-55	0.339846199	0.622	0.549	1.42E-50
Enterocyte	TMBIM1	4.39E-27	0.339753553	0.551	0.492	9.33E-23
Enterocyte	PNKD	8.21E-50	0.339466689	0.666	0.659	1.75E-45
Enterocyte	LRRC1	7.85E-21	0.338346098	0.349	0.294	1.67E-16
Enterocyte	SPATS2L	5.09E-12	0.338216166	0.64	0.649	1.08E-07
Enterocyte	CAPN5	1.04E-74	0.337068558	0.47	0.323	2.21E-70
Enterocyte	FBXW5	2.87E-34	0.335769186	0.609	0.572	6.10E-30
Enterocyte	FAM3D	5.67E-66	0.335123769	0.884	0.877	1.21E-61
Enterocyte	PTTG1IP	5.62E-29	0.334523577	0.697	0.653	1.20E-24
Enterocyte	TMEM176A	8.94E-36	0.334096635	0.646	0.571	1.90E-31
Enterocyte	RARRES1	5.40E-106	0.333992617	0.466	0.27	1.15E-101
Enterocyte	FLNB	4.11E-29	0.333902852	0.519	0.458	8.74E-25
Enterocyte	SLC25A23	3.02E-46	0.333711212	0.327	0.224	6.43E-42
Enterocyte	VILL	2.49E-51	0.333654374	0.377	0.262	5.28E-47
Enterocyte	COX8A	2.39E-92	0.333457823	0.911	0.925	5.09E-88
Enterocyte	GSKIP	8.98E-32	0.332845435	0.562	0.503	1.91E-27
Enterocyte	DENND2A	2.23E-108	0.331950198	0.339	0.165	4.74E-104
Enterocyte	SULF2	1.69E-104	0.331223219	0.35	0.178	3.60E-100
Enterocyte	CALCOCO2	8.22E-50	0.330786354	0.523	0.431	1.75E-45
Enterocyte	SEMA4G	3.33E-103	0.32875598	0.363	0.194	7.09E-99
Enterocyte	ATP5PF	3.14E-69	0.327890761	0.905	0.915	6.68E-65
Enterocyte	CDC42BPA	7.54E-49	0.32694765	0.429	0.321	1.60E-44
Enterocyte	ERBIN	8.42E-09	0.326204037	0.349	0.336	0.000178946
Enterocyte	TMEM9B	9.28E-47	0.326147871	0.618	0.568	1.97E-42
Enterocyte	SIRT6	5.74E-23	0.325296064	0.325	0.265	1.22E-18
Enterocyte	LASP1	4.37E-29	0.325219301	0.581	0.543	9.29E-25
Enterocyte	ATP10B	1.42E-45	0.324905752	0.613	0.543	3.03E-41
Enterocyte	JUP	6.29E-26	0.323716925	0.645	0.613	1.34E-21
Enterocyte	LPCAT4	2.90E-85	0.321744165	0.44	0.29	6.16E-81
Enterocyte	TMSB4X	9.06E-106	0.321684623	1	0.998	1.93E-101
Enterocyte	ADTRP	6.11E-86	0.321088704	0.338	0.182	1.30E-81
Enterocyte	CYCS	8.77E-54	0.321059077	0.839	0.859	1.86E-49
Enterocyte	CHMP1B	1.12E-17	0.320694785	0.58	0.556	2.37E-13
Enterocyte	COX4I1	3.75E-104	0.319991	0.966	0.97	7.97E-100
Enterocyte	TOB1	1.34E-45	0.318813079	0.675	0.616	2.84E-41
Enterocyte	LPP	1.37E-49	0.315950532	0.698	0.668	2.91E-45

Enterocyte	MKNK2	1.59E-41	0.315864488	0.525	0.448	3.38E-37
Enterocyte	CHMP2B	4.09E-12	0.315505078	0.657	0.654	8.70E-08
Enterocyte	PCSK7	3.92E-57	0.315498934	0.686	0.608	8.33E-53
Enterocyte	MAPK3	5.81E-38	0.31525228	0.537	0.471	1.24E-33
Enterocyte	BCL10	3.06E-34	0.314578923	0.503	0.43	6.51E-30
Enterocyte	NR4A1	2.03E-24	0.314204726	0.583	0.56	4.32E-20
Enterocyte	H2AFJ	5.19E-28	0.313970686	0.831	0.815	1.10E-23
Enterocyte	NHSL1	1.07E-60	0.312891693	0.417	0.288	2.27E-56
Enterocyte	ST14	1.29E-16	0.312748174	0.834	0.828	2.74E-12
Enterocyte	ACAA2	1.01E-18	0.311587314	0.609	0.582	2.15E-14
Enterocyte	MYO1D	1.95E-46	0.310574728	0.501	0.419	4.15E-42
Enterocyte	GPRC5A	8.67E-09	0.31037805	0.614	0.615	0.000184416
Enterocyte	ELF3	3.46E-87	0.309812093	0.968	0.959	7.35E-83
Enterocyte	KTN1	3.81E-34	0.30962621	0.791	0.789	8.11E-30
Enterocyte	VIPR1	3.51E-54	0.309251058	0.279	0.162	7.47E-50
Enterocyte	PLOD2	4.97E-48	0.309174141	0.275	0.158	1.06E-43
Enterocyte	KRAS	2.57E-44	0.308945002	0.605	0.555	5.47E-40
Enterocyte	PPIC	7.15E-41	0.308860929	0.675	0.656	1.52E-36
Enterocyte	COX6B1	6.41E-84	0.308605914	0.939	0.953	1.36E-79
Enterocyte	RAPGEFL1	2.95E-93	0.308074305	0.353	0.195	6.28E-89
Enterocyte	ASAH1	3.36E-44	0.307681152	0.633	0.58	7.14E-40
Enterocyte	MIEN1	1.87E-37	0.306639537	0.681	0.646	3.97E-33
Enterocyte	ECH1	4.85E-45	0.306596713	0.75	0.754	1.03E-40
Enterocyte	LIPH	1.44E-66	0.306509578	0.637	0.505	3.07E-62
Enterocyte	ECE1	1.15E-43	0.306029948	0.28	0.178	2.46E-39
Enterocyte	PLXNB2	9.40E-18	0.305995513	0.436	0.395	2.00E-13
Enterocyte	SEPHS2	1.48E-36	0.30562463	0.663	0.616	3.15E-32
Enterocyte	NCOR1	1.02E-42	0.304982254	0.718	0.682	2.17E-38
Enterocyte	BMP2	5.83E-81	0.304972096	0.281	0.134	1.24E-76
Enterocyte	EPB41L4B	3.01E-51	0.304706574	0.433	0.329	6.39E-47
Enterocyte	WASL	7.76E-47	0.303415445	0.569	0.5	1.65E-42
Enterocyte	PDCD6IP	4.64E-27	0.303047556	0.598	0.567	9.87E-23
Enterocyte	AKAP9	4.26E-38	0.303005102	0.8	0.79	9.05E-34
Enterocyte	LGR4	4.23E-55	0.301450984	0.467	0.357	9.00E-51
Enterocyte	CYP4F12	6.65E-93	0.30005336	0.298	0.145	1.41E-88
Enterocyte	CES3	6.65E-85	0.300017467	0.352	0.199	1.42E-80
Enterocyte	PRR15L	2.63E-17	0.299406807	0.581	0.556	5.59E-13
Enterocyte	MAP2K2	1.69E-34	0.298679191	0.601	0.552	3.60E-30
Enterocyte	CYBA	2.57E-57	0.298099956	0.883	0.893	5.47E-53
Enterocyte	CGN	6.96E-25	0.297878144	0.313	0.245	1.48E-20
Enterocyte	FZD5	4.41E-48	0.297786939	0.423	0.319	9.37E-44
Enterocyte	INAVA	8.52E-27	0.29776793	0.434	0.361	1.81E-22
Enterocyte	MTMR11	3.41E-54	0.297607706	0.451	0.325	7.25E-50

Enterocyte	ATP6V0D1	1.12E-33	0.297217562	0.564	0.517	2.38E-29
Enterocyte	ABHD3	5.59E-08	0.295919303	0.444	0.437	0.001189283
Enterocyte	EPS8L2	4.52E-17	0.29517683	0.48	0.453	9.61E-13
Enterocyte	EPS8L3	1.09E-23	0.294467572	0.535	0.492	2.32E-19
Enterocyte	ENTPD8	1.82E-55	0.292716705	0.292	0.159	3.88E-51
Enterocyte	TAOK3	1.12E-34	0.292489729	0.607	0.564	2.38E-30
Enterocyte	NDUFA2	5.23E-47	0.292280087	0.812	0.807	1.11E-42
Enterocyte	GSDMD	4.69E-27	0.292106524	0.587	0.532	9.96E-23
Enterocyte	PIGZ	2.38E-48	0.291467669	0.4	0.283	5.06E-44
Enterocyte	ASS1	2.46E-08	0.290504944	0.591	0.566	0.000522904
Enterocyte	SRSF5	1.57E-47	0.290498418	0.815	0.781	3.33E-43
Enterocyte	MUC5B	3.24E-25	0.290282471	0.419	0.338	6.89E-21
Enterocyte	COX7A2	1.64E-76	0.28992824	0.935	0.957	3.49E-72
Enterocyte	NDUFB8	2.04E-37	0.287736221	0.743	0.795	4.34E-33
Enterocyte	PLLP	7.24E-72	0.287607778	0.273	0.145	1.54E-67
Enterocyte	GNG12	1.13E-22	0.28742804	0.519	0.484	2.41E-18
Enterocyte	SLC35A3	3.08E-40	0.287079774	0.668	0.633	6.56E-36
Enterocyte	ARHGDIA	1.04E-29	0.286441024	0.661	0.627	2.21E-25
Enterocyte	MCL1	1.45E-25	0.285551965	0.685	0.668	3.09E-21
Enterocyte	UQCRFS1	2.49E-31	0.284708863	0.754	0.806	5.30E-27
Enterocyte	RETREG1	1.09E-20	0.284030303	0.472	0.432	2.32E-16
Enterocyte	PPP1R15A	1.80E-23	0.283308226	0.661	0.628	3.83E-19
Enterocyte	NDUFA3	6.32E-48	0.282976273	0.827	0.859	1.34E-43
Enterocyte	MUC4	2.33E-67	0.282149511	0.654	0.526	4.96E-63
Enterocyte	A1CF	8.55E-49	0.280643549	0.341	0.231	1.82E-44
Enterocyte	EZR	2.66E-23	0.280172807	0.738	0.709	5.67E-19
Enterocyte	HERPUD1	1.09E-38	0.279105782	0.68	0.643	2.32E-34
Enterocyte	FOSL2	2.63E-24	0.278541051	0.435	0.378	5.59E-20
Enterocyte	ATP5MC3	3.76E-29	0.277799505	0.85	0.902	7.99E-25
Enterocyte	DNM2	4.75E-21	0.277624102	0.557	0.528	1.01E-16
Enterocyte	IVNS1ABP	3.07E-22	0.276861033	0.499	0.463	6.52E-18
Enterocyte	GLTP	9.50E-46	0.276515265	0.443	0.352	2.02E-41
Enterocyte	IRF1	1.93E-08	0.275964626	0.429	0.406	0.000409516
Enterocyte	KIAA2013	6.80E-16	0.275571121	0.428	0.395	1.45E-11
Enterocyte	TXNDC17	2.21E-36	0.275479892	0.799	0.814	4.71E-32
Enterocyte	PHYKPL	7.00E-44	0.275030196	0.462	0.366	1.49E-39
Enterocyte	EFHD2	5.19E-20	0.274434389	0.642	0.63	1.10E-15
Enterocyte	SMIM14	8.98E-73	0.274085204	0.684	0.588	1.91E-68
Enterocyte	ACAT1	2.04E-35	0.274068893	0.521	0.442	4.35E-31
Enterocyte	KIF1C	2.04E-30	0.274059028	0.456	0.391	4.33E-26
Enterocyte	BICDL2	1.11E-38	0.272572648	0.594	0.522	2.37E-34
Enterocyte	B4GALNT3	5.70E-80	0.272501679	0.29	0.147	1.21E-75
Enterocyte	PLEKHG6	1.65E-31	0.272412657	0.305	0.224	3.51E-27

Enterocyte	EDF1	5.72E-51	0.271887596	0.953	0.944	1.22E-46
Enterocyte	MT-CYB	2.64E-78	0.271277826	0.987	0.988	5.61E-74
Enterocyte	EIF4EBP2	6.08E-10	0.271217207	0.506	0.506	1.29E-05
Enterocyte	QSOX1	1.12E-57	0.271187445	0.588	0.477	2.37E-53
Enterocyte	CKMT1B	1.04E-38	0.2704527	0.448	0.358	2.20E-34
Enterocyte	ATF3	6.48E-28	0.26984742	0.583	0.51	1.38E-23
Enterocyte	CDC42EP5	3.64E-48	0.269563446	0.776	0.75	7.74E-44
Enterocyte	OPTN	4.28E-07	0.269388107	0.449	0.442	0.009107472
Enterocyte	S100A16	1.98E-36	0.269091811	0.726	0.737	4.21E-32
Enterocyte	MMP28	2.63E-173	0.268318576	0.265	0.073	5.58E-169
Enterocyte	GHITM	7.65E-37	0.267496761	0.787	0.792	1.63E-32
Enterocyte	GUCY2C	8.10E-26	0.266196109	0.375	0.307	1.72E-21
Enterocyte	NDUFC1	1.57E-36	0.265806832	0.78	0.825	3.34E-32
Enterocyte	SUCLG1	2.42E-24	0.264360257	0.7	0.727	5.15E-20
Enterocyte	TPMT	1.86E-18	0.264037049	0.509	0.473	3.95E-14
Enterocyte	CCL20	1.41E-11	0.263894449	0.172	0.25	3.01E-07
Enterocyte	KLC4	3.54E-52	0.263427371	0.361	0.245	7.53E-48
Enterocyte	SHD	1.48E-71	0.26329463	0.34	0.199	3.15E-67
Enterocyte	PPP1R14C	1.25E-19	0.262814813	0.274	0.213	2.65E-15
Enterocyte	IFI27	2.03E-08	0.262306743	0.972	0.969	0.000432092
Enterocyte	PRR34-AS1	1.21E-32	0.26219013	0.348	0.261	2.57E-28
Enterocyte	CFTR	5.67E-32	0.26189317	0.497	0.427	1.21E-27
Enterocyte	NDUFB2	2.31E-40	0.26048678	0.833	0.887	4.90E-36
Enterocyte	ERBB2	1.47E-27	0.260326936	0.503	0.453	3.13E-23
Enterocyte	KIF13B	7.26E-49	0.260228367	0.351	0.238	1.54E-44
Enterocyte	DAZAP2	2.83E-27	0.259644193	0.78	0.771	6.02E-23
Enterocyte	MAFF	1.41E-28	0.259063302	0.324	0.246	3.00E-24
Enterocyte	TP53I3	2.32E-30	0.258018125	0.46	0.395	4.94E-26
Enterocyte	C1orf210	2.39E-42	0.25596688	0.34	0.235	5.08E-38
Enterocyte	ATP5MD	6.63E-32	0.255480278	0.898	0.933	1.41E-27
Enterocyte	PLPP1	1.16E-23	0.255397526	0.283	0.214	2.46E-19
Enterocyte	PLEKHJ1	1.94E-24	0.255249516	0.718	0.698	4.13E-20
Enterocyte	TLE4	7.68E-28	0.254507288	0.366	0.282	1.63E-23
Enterocyte	C5orf30	6.43E-14	0.254328358	0.257	0.211	1.37E-09
Enterocyte	ARHGAP27	9.87E-32	0.254026363	0.385	0.306	2.10E-27
Enterocyte	RABAC1	9.02E-40	0.253898151	0.766	0.762	1.92E-35
Enterocyte	RFK	0.007626098	0.253823092	0.434	0.511	1
Enterocyte	BAIAP2L2	1.24E-43	0.25382239	0.299	0.195	2.63E-39
Enterocyte	DDX5	8.16E-48	0.253788079	0.877	0.849	1.73E-43
Enterocyte	MTUS1	1.43E-36	0.252621838	0.389	0.3	3.04E-32
Enterocyte	EPAS1	2.38E-41	0.252431047	0.327	0.224	5.07E-37
Enterocyte	ACTR2	7.34E-26	0.252264735	0.739	0.752	1.56E-21
Enterocyte	NDUFA6	1.33E-30	0.251467692	0.779	0.764	2.84E-26

Enterocyte	VPS4B	5.94E-27	0.250662592	0.546	0.49	1.26E-22
Enterocyte	SH3BGRL2	6.00E-29	0.250477952	0.532	0.479	1.28E-24
Enterocyte	TMBIM6	7.30E-52	0.250388984	0.93	0.934	1.55E-47
Enterocyte	SCAMP2	6.26E-25	0.250301285	0.51	0.464	1.33E-20
Enterocyte	C18orf32	3.49E-20	0.25025215	0.353	0.294	7.43E-16
Enteroendocrine	CHGA	0	6.021020916	0.418	0.01	0
Enteroendocrine	PCSK1N	1.56E-149	4.255455144	0.557	0.06	3.31E-145
Enteroendocrine	CRYBA2	0	3.507864956	0.373	0.005	0
Enteroendocrine	TTR	3.03E-154	3.226218422	0.5	0.046	6.44E-150
Enteroendocrine	TUBA1A	3.72E-135	3.084264162	0.842	0.187	7.91E-131
Enteroendocrine	HES6	2.79E-25	3.064550109	0.633	0.431	5.93E-21
Enteroendocrine	SOX4	3.36E-24	2.737277505	0.778	0.742	7.15E-20
Enteroendocrine	ANXA13	2.18E-39	2.699605068	0.557	0.219	4.63E-35
Enteroendocrine	SCGN	0	2.642089008	0.551	0.002	0
Enteroendocrine	MDK	3.57E-54	2.612087801	0.899	0.687	7.59E-50
Enteroendocrine	HEPACAM2	5.86E-98	2.575911907	0.677	0.14	1.25E-93
Enteroendocrine	PAX4	0	2.557197049	0.475	0.003	0
Enteroendocrine	SCG2	0	2.432293108	0.437	0.001	0
Enteroendocrine	RGS2	7.60E-108	2.428559241	0.538	0.079	1.62E-103
Enteroendocrine	KLK12	2.53E-98	2.388851249	0.551	0.088	5.37E-94
Enteroendocrine	CPE	5.79E-175	2.359593822	0.816	0.122	1.23E-170
Enteroendocrine	CADPS	5.62E-81	2.231219307	0.633	0.156	1.19E-76
Enteroendocrine	GADD45G	8.42E-46	2.200621566	0.525	0.165	1.79E-41
Enteroendocrine	SEC11C	1.81E-28	2.102339526	0.728	0.606	3.84E-24
Enteroendocrine	RGS16	3.49E-42	2.095411323	0.329	0.068	7.43E-38
Enteroendocrine	PAM	4.95E-108	2.067452103	0.69	0.141	1.05E-103
Enteroendocrine	NEUROG3	0	2.057327715	0.411	0.006	0
Enteroendocrine	INSM1	0	1.985866857	0.608	0.007	0
Enteroendocrine	RASD1	2.36E-35	1.833515183	0.443	0.136	5.01E-31
Enteroendocrine	BTG2	3.31E-27	1.768888728	0.797	0.669	7.03E-23
Enteroendocrine	CDKN1C	6.71E-59	1.768158959	0.411	0.079	1.43E-54
Enteroendocrine	SCG5	4.87E-77	1.714869667	0.589	0.13	1.04E-72
Enteroendocrine	MS4A8	5.15E-82	1.708365492	0.506	0.091	1.10E-77
Enteroendocrine	FEV	0	1.616919032	0.285	0.001	0
Enteroendocrine	KCNQ1OT1	2.10E-27	1.599362013	0.595	0.311	4.48E-23
Enteroendocrine	VPS13A	8.13E-46	1.578701196	0.759	0.436	1.73E-41
Enteroendocrine	CDKN1A	1.32E-13	1.567216725	0.551	0.387	2.80E-09
Enteroendocrine	TPPP3	3.51E-16	1.564657682	0.285	0.105	7.46E-12
Enteroendocrine	CACNA1A	5.44E-271	1.513079291	0.405	0.015	1.16E-266
Enteroendocrine	KCTD12	1.19E-46	1.506544107	0.538	0.173	2.53E-42
Enteroendocrine	HSPA2	1.19E-39	1.494929441	0.411	0.11	2.53E-35
Enteroendocrine	BTG1	7.65E-22	1.44479399	0.785	0.785	1.63E-17
Enteroendocrine	RAB26	6.16E-145	1.420511652	0.557	0.061	1.31E-140

Enteroendocrine	RASA4	1.08E-123	1.404272454	0.411	0.038	2.31E-119
Enteroendocrine	NPW	7.71E-39	1.400655528	0.38	0.093	1.64E-34
Enteroendocrine	DDC	4.17E-14	1.388779887	0.418	0.235	8.87E-10
Enteroendocrine	STMN1	6.13E-16	1.332781196	0.627	0.455	1.30E-11
Enteroendocrine	CHD7	1.32E-18	1.295814885	0.57	0.395	2.81E-14
Enteroendocrine	FAM241B	2.56E-47	1.295436395	0.449	0.116	5.44E-43
Enteroendocrine	PHLDA1	6.05E-08	1.293840998	0.437	0.342	0.001286225
Enteroendocrine	EGR1	2.26E-18	1.285040404	0.759	0.671	4.80E-14
Enteroendocrine	MALAT1	1.33E-50	1.283152305	1	0.994	2.83E-46
Enteroendocrine	VWA5B2	0	1.277918562	0.544	0.004	0
Enteroendocrine	RAB3B	6.24E-107	1.265973738	0.367	0.034	1.33E-102
Enteroendocrine	IER2	2.69E-18	1.259922518	0.829	0.853	5.72E-14
Enteroendocrine	IL13RA1	5.56E-12	1.244694449	0.519	0.415	1.18E-07
Enteroendocrine	SRP19	2.41E-10	1.223180792	0.576	0.514	5.12E-06
Enteroendocrine	MARCKS	8.03E-19	1.221376736	0.797	0.813	1.71E-14
Enteroendocrine	RHOBTB3	7.65E-20	1.22130411	0.665	0.53	1.63E-15
Enteroendocrine	SMIM24	4.88E-24	1.206854412	0.5	0.233	1.04E-19
Enteroendocrine	SMAD9	4.42E-28	1.191409161	0.494	0.207	9.39E-24
Enteroendocrine	DEPP1	9.06E-19	1.183470501	0.304	0.103	1.93E-14
Enteroendocrine	DNAJC12	1.63E-58	1.162273531	0.506	0.116	3.48E-54
Enteroendocrine	PCSK1	3.84E-22	1.147908499	0.304	0.091	8.16E-18
Enteroendocrine	TRIB1	1.56E-07	1.142081524	0.519	0.451	0.003314409
Enteroendocrine	CBFA2T2	3.55E-21	1.120346446	0.481	0.24	7.54E-17
Enteroendocrine	QDPR	7.81E-18	1.102716883	0.557	0.39	1.66E-13
Enteroendocrine	CDKN2A	4.38E-35	1.097342524	0.456	0.146	9.31E-31
Enteroendocrine	TUBA4A	2.31E-13	1.097127618	0.57	0.434	4.92E-09
Enteroendocrine	SLC29A4	1.11E-258	1.092553242	0.418	0.017	2.35E-254
Enteroendocrine	BAZ2B	1.45E-21	1.078390187	0.652	0.458	3.09E-17
Enteroendocrine	GNAS	9.50E-25	1.069237606	0.88	0.856	2.02E-20
Enteroendocrine	QPCT	9.46E-11	1.065692083	0.31	0.157	2.01E-06
Enteroendocrine	UCP2	5.75E-29	1.057153767	0.589	0.289	1.22E-24
Enteroendocrine	FXYD2	9.02E-252	1.056243253	0.291	0.007	1.92E-247
Enteroendocrine	SYT13	2.68E-23	1.055158714	0.456	0.197	5.70E-19
Enteroendocrine	HMGB3	1.44E-09	1.053986283	0.544	0.447	3.06E-05
Enteroendocrine	H3F3B	6.33E-21	1.05268335	0.93	0.966	1.35E-16
Enteroendocrine	RIMBP2	0	1.047641193	0.468	0.008	0
Enteroendocrine	MATN2	2.55E-22	1.032552672	0.424	0.179	5.43E-18
Enteroendocrine	MARCKSL1	9.23E-25	1.031699477	0.854	0.877	1.96E-20
Enteroendocrine	FOXA2	5.04E-18	1.031359326	0.532	0.318	1.07E-13
Enteroendocrine	MIR7-3HG	0	1.030560245	0.285	0	0
Enteroendocrine	PBXIP1	1.20E-29	1.024989769	0.481	0.194	2.55E-25
Enteroendocrine	PTP4A3	2.54E-17	1.007001996	0.354	0.149	5.40E-13
Enteroendocrine	FTX	2.26E-10	1.001642378	0.532	0.401	4.81E-06

Enteroendocrine	PLA2G12A	7.73E-12	1.001246425	0.449	0.304	1.64E-07
Enteroendocrine	STARD10	1.80E-17	0.999260775	0.804	0.816	3.82E-13
Enteroendocrine	C4orf48	1.59E-16	0.996456366	0.747	0.692	3.39E-12
Enteroendocrine	SSR4	4.33E-31	0.996420023	0.93	0.897	9.20E-27
Enteroendocrine	C2CD4B	1.40E-18	0.987688437	0.285	0.096	2.99E-14
Enteroendocrine	HNRNPA0	1.83E-12	0.985714007	0.684	0.727	3.89E-08
Enteroendocrine	CDKAL1	2.09E-22	0.983575857	0.323	0.106	4.45E-18
Enteroendocrine	GCH1	4.31E-24	0.968989097	0.399	0.153	9.16E-20
Enteroendocrine	ELL2	2.90E-43	0.967734411	0.456	0.126	6.18E-39
Enteroendocrine	TBPL1	7.59E-11	0.966080504	0.5	0.384	1.61E-06
Enteroendocrine	RFX3	7.37E-77	0.953836383	0.5	0.093	1.57E-72
Enteroendocrine	ENY2	2.91E-07	0.952278113	0.677	0.75	0.006185542
Enteroendocrine	ST18	0	0.942218141	0.392	0.007	0
Enteroendocrine	GADD45A	5.74E-12	0.926535516	0.525	0.368	1.22E-07
Enteroendocrine	ZNF706	5.69E-15	0.922919101	0.766	0.806	1.21E-10
Enteroendocrine	AQP3	2.16E-14	0.920258119	0.259	0.09	4.60E-10
Enteroendocrine	SPINK1	2.79E-07	0.91790408	0.886	0.818	0.005942671
Enteroendocrine	DUSP1	1.17E-09	0.911443299	0.741	0.695	2.48E-05
Enteroendocrine	IHH	3.59E-08	0.902886618	0.424	0.31	0.000763114
Enteroendocrine	PCBP4	8.45E-29	0.896967399	0.43	0.157	1.80E-24
Enteroendocrine	CDC42EP3	6.76E-19	0.891553198	0.456	0.222	1.44E-14
Enteroendocrine	JUNB	2.44E-07	0.890755091	0.823	0.855	0.005186099
Enteroendocrine	HMGN3	8.51E-17	0.887042556	0.696	0.615	1.81E-12
Enteroendocrine	KIAA1324	5.70E-20	0.882942166	0.658	0.445	1.21E-15
Enteroendocrine	BNIP3L	3.96E-14	0.877201776	0.563	0.409	8.42E-10
Enteroendocrine	NR2F1	5.09E-43	0.872861845	0.335	0.068	1.08E-38
Enteroendocrine	ATP2B1	1.57E-05	0.872437391	0.494	0.475	0.333866342
Enteroendocrine	RFX6	0	0.872200999	0.386	0.002	0
Enteroendocrine	TTC3	2.98E-14	0.870388356	0.728	0.733	6.33E-10
Enteroendocrine	DPP4	1.38E-33	0.865144416	0.405	0.119	2.93E-29
Enteroendocrine	XIST	3.26E-06	0.862476919	0.266	0.154	0.069393386
Enteroendocrine	EID1	5.19E-14	0.859117018	0.658	0.576	1.10E-09
Enteroendocrine	AMIGO2	8.10E-77	0.85883066	0.43	0.066	1.72E-72
Enteroendocrine	CRIP2	9.21E-157	0.857966671	0.253	0.01	1.96E-152
Enteroendocrine	CACUL1	2.07E-08	0.852626105	0.5	0.428	0.000440579
Enteroendocrine	IGFBP2	0.009275292	0.849829604	0.551	0.533	1
Enteroendocrine	FAM213A	1.18E-06	0.84479227	0.538	0.514	0.024993345
Enteroendocrine	AL450311.2	0	0.843107103	0.323	0.004	0
Enteroendocrine	DYNLT3	2.25E-14	0.843076029	0.519	0.357	4.79E-10
Enteroendocrine	REV3L	6.69E-18	0.841850934	0.475	0.26	1.42E-13
Enteroendocrine	BAMBI	1.87E-11	0.836796607	0.31	0.145	3.97E-07
Enteroendocrine	COLCA1	2.42E-30	0.833228188	0.392	0.118	5.14E-26
Enteroendocrine	NEURL1	3.02E-44	0.821722695	0.437	0.106	6.43E-40

Enteroendocrine	GSE1	2.40E-14	0.81556719	0.513	0.341	5.10E-10
Enteroendocrine	CYP2W1	3.05E-22	0.808094351	0.38	0.135	6.48E-18
Enteroendocrine	SELENOK	7.07E-14	0.804275408	0.753	0.745	1.50E-09
Enteroendocrine	BNIP3	4.79E-12	0.802961033	0.291	0.124	1.02E-07
Enteroendocrine	GABARAPL2	1.22E-11	0.80105936	0.728	0.751	2.60E-07
Enteroendocrine	RAMP1	5.77E-54	0.799093372	0.329	0.053	1.23E-49
Enteroendocrine	EXOC7	2.17E-12	0.797960457	0.5	0.351	4.62E-08
Enteroendocrine	PHIP	3.12E-09	0.791976722	0.582	0.501	6.64E-05
Enteroendocrine	C9orf16	6.86E-15	0.787681993	0.81	0.764	1.46E-10
Enteroendocrine	ETV1	1.65E-177	0.784362119	0.266	0.01	3.51E-173
Enteroendocrine	TM7SF2	4.05E-19	0.784138095	0.506	0.278	8.61E-15
Enteroendocrine	PTMS	3.20E-10	0.782926098	0.684	0.66	6.81E-06
Enteroendocrine	SYP	5.13E-80	0.779963329	0.392	0.053	1.09E-75
Enteroendocrine	DDX5	2.76E-14	0.779351222	0.804	0.856	5.88E-10
Enteroendocrine	STX1A	1.65E-48	0.778747888	0.259	0.037	3.50E-44
Enteroendocrine	AL450384.2	1.07E-20	0.778706214	0.291	0.091	2.27E-16
Enteroendocrine	ACTG1	7.51E-06	0.777953804	0.937	0.972	0.1598021
Enteroendocrine	A1CF	9.77E-13	0.774257585	0.437	0.254	2.08E-08
Enteroendocrine	ROCK1	1.56E-13	0.772450421	0.563	0.438	3.32E-09
Enteroendocrine	TMEM61	1.44E-51	0.771543511	0.361	0.065	3.07E-47
Enteroendocrine	AFF4	1.42E-12	0.76116266	0.538	0.401	3.02E-08
Enteroendocrine	MCOLN3	6.96E-65	0.760135328	0.285	0.034	1.48E-60
Enteroendocrine	ANK2	0	0.747657444	0.285	0	0
Enteroendocrine	RTN4	4.52E-11	0.747390873	0.753	0.805	9.61E-07
Enteroendocrine	SCAND1	5.95E-13	0.745702746	0.741	0.78	1.26E-08
Enteroendocrine	TDG	9.17E-06	0.744575453	0.475	0.452	0.195095004
Enteroendocrine	CBX6	1.41E-74	0.7440206	0.367	0.049	3.00E-70
Enteroendocrine	MYL6B	2.15E-14	0.739972284	0.5	0.317	4.57E-10
Enteroendocrine	ZNF428	8.47E-11	0.737382486	0.544	0.438	1.80E-06
Enteroendocrine	CARHSP1	1.55E-11	0.737019931	0.69	0.64	3.30E-07
Enteroendocrine	PRDX1	0.000690629	0.735092597	0.753	0.866	1
Enteroendocrine	ATF3	0.006300707	0.730251739	0.532	0.527	1
Enteroendocrine	DNPEP	1.58E-05	0.729698424	0.557	0.573	0.335180935
Enteroendocrine	TCEAL4	6.05E-10	0.726652255	0.487	0.362	1.29E-05
Enteroendocrine	HIST2H2BE	2.69E-20	0.724274638	0.342	0.121	5.72E-16
Enteroendocrine	OTULINL	3.71E-05	0.72065192	0.475	0.443	0.788097152
Enteroendocrine	MLXIPL	4.07E-17	0.720400882	0.443	0.22	8.64E-13
Enteroendocrine	F10	2.48E-130	0.718416532	0.285	0.016	5.28E-126
Enteroendocrine	RBPJ	5.09E-08	0.717765644	0.513	0.436	0.001082922
Enteroendocrine	ATP6V1G1	2.09E-12	0.717243689	0.753	0.849	4.44E-08
Enteroendocrine	IDS	3.19E-12	0.715969761	0.443	0.264	6.78E-08
Enteroendocrine	ARFGEF3	1.30E-12	0.715746033	0.519	0.365	2.77E-08
Enteroendocrine	TIMP1	0.002900714	0.71433847	0.551	0.545	1

Enteroendocrine	BMP4	4.70E-07	0.714243007	0.342	0.218	0.009987866
Enteroendocrine	TOX	5.74E-38	0.712685082	0.354	0.085	1.22E-33
Enteroendocrine	FFAR2	1.13E-260	0.711171883	0.335	0.01	2.39E-256
Enteroendocrine	RASSF6	1.35E-09	0.707158204	0.494	0.345	2.86E-05
Enteroendocrine	KCNH6	0	0.706952229	0.304	0.003	0
Enteroendocrine	DHRS7	0.000137676	0.706244802	0.544	0.602	1
Enteroendocrine	PRUNE2	3.44E-17	0.701196183	0.487	0.253	7.31E-13
Enteroendocrine	HDAC2	1.01E-05	0.697425109	0.595	0.623	0.214227355
Enteroendocrine	H3F3A	1.18E-14	0.693742123	0.956	0.976	2.52E-10
Enteroendocrine	CAST	4.20E-06	0.692510996	0.747	0.849	0.089314658
Enteroendocrine	ARID3A	9.27E-10	0.68765787	0.405	0.256	1.97E-05
Enteroendocrine	INSR	0.000497334	0.686541631	0.481	0.447	1
Enteroendocrine	HNRNPH1	0.002659593	0.682545888	0.646	0.694	1
Enteroendocrine	STIM2	1.48E-13	0.681615898	0.399	0.212	3.15E-09
Enteroendocrine	DSP	8.56E-08	0.675057364	0.709	0.73	0.001819421
Enteroendocrine	RANBP2	5.73E-09	0.671118162	0.513	0.415	0.000121924
Enteroendocrine	ATP5F1E	9.24E-24	0.670934811	0.962	0.96	1.96E-19
Enteroendocrine	H1FX	2.08E-07	0.670601815	0.563	0.504	0.004415103
Enteroendocrine	GCLC	8.59E-09	0.669849921	0.329	0.193	0.000182618
Enteroendocrine	POLR2J3.1	1.60E-08	0.666267562	0.595	0.548	0.000340599
Enteroendocrine	RSRC1	5.67E-08	0.65378606	0.475	0.368	0.001205399
Enteroendocrine	SINHCAF	4.86E-07	0.653186591	0.551	0.502	0.010331711
Enteroendocrine	PAIP2	8.43E-07	0.652985292	0.601	0.586	0.017924808
Enteroendocrine	SVBP	1.36E-12	0.650375642	0.481	0.324	2.89E-08
Enteroendocrine	ZNF608	1.11E-36	0.650059244	0.373	0.095	2.35E-32
Enteroendocrine	FKBP2	2.34E-10	0.648795624	0.81	0.825	4.98E-06
Enteroendocrine	PHF14	7.90E-05	0.647287175	0.576	0.601	1
Enteroendocrine	SELENOW	2.52E-09	0.646589982	0.848	0.827	5.35E-05
Enteroendocrine	CYP27A1	2.67E-16	0.642206657	0.342	0.145	5.68E-12
Enteroendocrine	PTPRN2	4.85E-13	0.64215923	0.259	0.1	1.03E-08
Enteroendocrine	ABCC5	1.99E-13	0.641469542	0.297	0.124	4.23E-09
Enteroendocrine	SELENOM	4.16E-17	0.64019608	0.456	0.214	8.85E-13
Enteroendocrine	PNISR	1.08E-08	0.637826714	0.759	0.772	0.000230236
Enteroendocrine	HSP90AA1	0.000113058	0.637553121	0.88	0.922	1
Enteroendocrine	FAM84B	0.001207009	0.635203634	0.31	0.257	1
Enteroendocrine	DLGAP4	7.26E-10	0.632731198	0.437	0.289	1.54E-05
Enteroendocrine	UBC	3.52E-08	0.629183954	0.918	0.945	0.00074752
Enteroendocrine	LMBR1L	1.55E-17	0.628777571	0.342	0.135	3.30E-13
Enteroendocrine	FBXL15	3.52E-07	0.628532471	0.494	0.422	0.00748062
Enteroendocrine	AES	1.11E-10	0.627733213	0.69	0.682	2.36E-06
Enteroendocrine	XRCC5	1.74E-06	0.627594875	0.639	0.684	0.037037716
Enteroendocrine	TOP1	9.54E-08	0.625260185	0.639	0.626	0.002028552
Enteroendocrine	OCIAD2	1.85E-09	0.623641622	0.785	0.82	3.93E-05

Enteroendocrine	TSPAN5	1.42E-16	0.622663292	0.367	0.158	3.02E-12
Enteroendocrine	FNBP1L	4.33E-05	0.619226797	0.5	0.489	0.919982113
Enteroendocrine	PROX1	7.47E-11	0.618872408	0.418	0.242	1.59E-06
Enteroendocrine	LRRFIP1	1.19E-08	0.616012478	0.69	0.686	0.000252308
Enteroendocrine	MTSS1	3.83E-16	0.615236028	0.38	0.173	8.14E-12
Enteroendocrine	PPP3CA	3.06E-06	0.612954476	0.532	0.482	0.065024551
Enteroendocrine	TRAF4	0.000113142	0.612069833	0.468	0.46	1
Enteroendocrine	CAMK2N1	3.32E-10	0.610045862	0.759	0.784	7.07E-06
Enteroendocrine	SCARB2	7.02E-09	0.608681651	0.614	0.56	0.000149194
Enteroendocrine	JUN	5.13E-08	0.607934136	0.918	0.934	0.001090946
Enteroendocrine	RSF1	8.30E-08	0.606852912	0.601	0.567	0.001765608
Enteroendocrine	KDM5B	2.77E-07	0.606170194	0.475	0.379	0.005889751
Enteroendocrine	GDAP1	4.27E-35	0.605186175	0.297	0.064	9.08E-31
Enteroendocrine	JMJD1C	6.29E-08	0.604196007	0.582	0.511	0.001337348
Enteroendocrine	UBE2E3	0.000226685	0.602707843	0.494	0.493	1
Enteroendocrine	CYTH2	1.51E-07	0.602596215	0.5	0.414	0.003202654
Enteroendocrine	RBM39	2.66E-11	0.602010042	0.791	0.836	5.66E-07
Enteroendocrine	DNAJA1	3.62E-08	0.601426185	0.709	0.713	0.000770745
Enteroendocrine	SKP1	3.90E-10	0.600676874	0.81	0.894	8.30E-06
Enteroendocrine	UBXN4	2.88E-11	0.600103512	0.772	0.788	6.12E-07
Enteroendocrine	CD200	1.70E-35	0.599935383	0.31	0.068	3.61E-31
Enteroendocrine	RASGEF1B	1.10E-05	0.595601941	0.253	0.151	0.234309296
Enteroendocrine	HOXA10	1.69E-05	0.595417726	0.494	0.453	0.358337113
Enteroendocrine	DBN1	6.19E-42	0.595327982	0.278	0.048	1.32E-37
Enteroendocrine	PHF20L1	4.71E-07	0.594722018	0.5	0.419	0.010008503
Enteroendocrine	ARID4B	1.06E-08	0.594161403	0.582	0.52	0.000225028
Enteroendocrine	TP53INP1	1.18E-19	0.593865393	0.329	0.11	2.51E-15
Enteroendocrine	N4BP2L2	7.18E-11	0.589088071	0.797	0.787	1.53E-06
Enteroendocrine	TPD52	1.50E-06	0.588339343	0.722	0.823	0.031890411
Enteroendocrine	HIST1H2BD	6.45E-07	0.585979537	0.38	0.241	0.013711396
Enteroendocrine	ARPC5	9.43E-09	0.583814435	0.734	0.779	0.000200626
Enteroendocrine	HIST1H2BG	8.77E-12	0.583746651	0.335	0.157	1.86E-07
Enteroendocrine	POLR2L	3.62E-05	0.583442928	0.848	0.877	0.769557513
Enteroendocrine	G3BP2	1.73E-05	0.581918125	0.563	0.572	0.367679738
Enteroendocrine	C1orf56	0.001367558	0.581359815	0.291	0.213	1
Enteroendocrine	GLS	2.33E-08	0.58088188	0.392	0.259	0.000496094
Enteroendocrine	PODXL2	5.37E-13	0.58051105	0.43	0.248	1.14E-08
Enteroendocrine	DDAH2	1.70E-06	0.579790373	0.646	0.643	0.036201234
Enteroendocrine	ZKSCAN1	2.96E-05	0.57788285	0.633	0.665	0.629748376
Enteroendocrine	KIAA0355	1.35E-10	0.576132308	0.386	0.219	2.88E-06
Enteroendocrine	TUBB	6.59E-05	0.573193851	0.677	0.683	1
Enteroendocrine	ATRX	2.31E-06	0.572194772	0.614	0.617	0.049214619
Enteroendocrine	C11orf58	1.53E-08	0.572172069	0.696	0.747	0.00324975

Enteroendocrine	LIMD2	3.33E-40	0.571421767	0.291	0.054	7.09E-36
Enteroendocrine	CISD1	0.001358016	0.569348373	0.595	0.639	1
Enteroendocrine	XBP1	0.00772835	0.569051816	0.62	0.728	1
Enteroendocrine	HIST1H2AC	3.28E-09	0.568996342	0.532	0.361	6.98E-05
Enteroendocrine	TRIM44	6.24E-06	0.566788784	0.5	0.461	0.132622905
Enteroendocrine	DACH1	3.09E-08	0.561204921	0.399	0.261	0.00065759
Enteroendocrine	ATP6V0B	1.45E-07	0.560179222	0.715	0.764	0.003091287
Enteroendocrine	NISCH	6.08E-12	0.557320442	0.31	0.141	1.29E-07
Enteroendocrine	TAF7	0.002231375	0.555081008	0.576	0.679	1
Enteroendocrine	NAPA	8.15E-07	0.553010082	0.519	0.456	0.017326112
Enteroendocrine	EMC10	1.34E-07	0.550136208	0.658	0.676	0.002854848
Enteroendocrine	ABHD13	6.41E-06	0.54901323	0.361	0.257	0.136338094
Enteroendocrine	ZNF638	7.73E-05	0.547853619	0.487	0.459	1
Enteroendocrine	TERF2IP	2.70E-06	0.546795478	0.475	0.417	0.057384277
Enteroendocrine	ARID1B	0.000444267	0.545681477	0.563	0.565	1
Enteroendocrine	ZNF326	5.62E-06	0.54497115	0.487	0.451	0.119506377
Enteroendocrine	HSPA8	0.005628037	0.54413628	0.747	0.846	1
Enteroendocrine	RCAN3	1.11E-09	0.543816218	0.405	0.252	2.36E-05
Enteroendocrine	GNAI3	2.57E-07	0.54290576	0.513	0.455	0.005466225
Enteroendocrine	SYT7	2.97E-21	0.541067819	0.323	0.105	6.32E-17
Enteroendocrine	CCDC14	0.000322685	0.537486018	0.544	0.535	1
Enteroendocrine	LUC7L3	6.82E-08	0.536614694	0.671	0.669	0.001449614
Enteroendocrine	BCL7A	7.11E-09	0.534772561	0.342	0.204	0.00015122
Enteroendocrine	RHOB	0.000455008	0.531891201	0.677	0.705	1
Enteroendocrine	RAP1GAP2	4.88E-08	0.530713306	0.259	0.132	0.0010387
Enteroendocrine	DYNLT1	5.94E-08	0.530047505	0.772	0.814	0.001262359
Enteroendocrine	CDC42SE1	0.001265876	0.529113909	0.348	0.284	1
Enteroendocrine	NREP	2.50E-10	0.528477007	0.285	0.134	5.32E-06
Enteroendocrine	JUND	3.88E-05	0.528112389	0.69	0.724	0.825505862
Enteroendocrine	KIDINS220	7.50E-06	0.527865307	0.392	0.305	0.159481816
Enteroendocrine	SAP30	1.43E-08	0.526481706	0.316	0.174	0.000304134
Enteroendocrine	TBCB	1.88E-07	0.525815119	0.608	0.567	0.004007906
Enteroendocrine	PPP1CB	0.001533577	0.525424752	0.652	0.727	1
Enteroendocrine	RNMT	3.19E-05	0.523136371	0.5	0.447	0.678978424
Enteroendocrine	RBFOX2	4.60E-05	0.523119471	0.392	0.326	0.978943647
Enteroendocrine	H2AFY2	7.53E-10	0.520403951	0.323	0.174	1.60E-05
Enteroendocrine	SRSF10	0.000211151	0.519526462	0.608	0.632	1
Enteroendocrine	LRP11	1.75E-05	0.518493756	0.361	0.271	0.373152559
Enteroendocrine	CPLX1	2.28E-57	0.517986383	0.278	0.036	4.84E-53
Enteroendocrine	DDX3X	0.00086539	0.516119434	0.582	0.603	1
Enteroendocrine	MICAL1	1.28E-11	0.515316996	0.297	0.135	2.71E-07
Enteroendocrine	PTK7	7.47E-09	0.514054545	0.335	0.194	0.000158855
Enteroendocrine	SMIM6	3.11E-20	0.513990794	0.361	0.128	6.62E-16

Enteroendocrine	MRFAP1	0.002506644	0.510953958	0.563	0.647	1
Enteroendocrine	SSBP3	0.001411868	0.510818565	0.443	0.419	1
Enteroendocrine	SI	1.05E-17	0.51022928	0.297	0.103	2.23E-13
Enteroendocrine	SORBS2	3.69E-07	0.509605183	0.38	0.255	0.007843202
Enteroendocrine	CSNK1A1	2.69E-05	0.509365261	0.671	0.766	0.571431525
Enteroendocrine	ANKS4B	3.02E-06	0.509301618	0.386	0.284	0.064123845
Enteroendocrine	NDFIP1	2.80E-06	0.507085499	0.614	0.621	0.05963955
Enteroendocrine	TROVE2	2.92E-05	0.506020567	0.418	0.344	0.621408098
Enteroendocrine	INPPL1	0.000118289	0.504820783	0.272	0.183	1
Enteroendocrine	RABGAP1L	0.004706771	0.504622233	0.367	0.342	1
Enteroendocrine	KCNB2	0	0.504393724	0.253	0	0
Enteroendocrine	ADH5	0.004341083	0.503324931	0.538	0.597	1
Enteroendocrine	HLA-A	1.62E-17	0.502777332	0.981	0.979	3.45E-13
Enteroendocrine	CLK1	4.29E-06	0.501170019	0.468	0.39	0.091290253
Enteroendocrine	SLC25A33	0.000141777	0.501148008	0.367	0.298	1
Enteroendocrine	CELF1	6.72E-05	0.499797034	0.544	0.531	1
Enteroendocrine	COX17	0.000128683	0.499524723	0.728	0.755	1
Enteroendocrine	EIF5B	8.05E-06	0.498738653	0.709	0.734	0.171260955
Enteroendocrine	UBE2I	0.000127377	0.498236344	0.652	0.71	1
Enteroendocrine	ZMYND8	1.44E-05	0.497671744	0.5	0.447	0.306910197
Enteroendocrine	GRAMD1A	0.002428239	0.497376926	0.405	0.385	1
Enteroendocrine	THAP7	0.003970282	0.496979789	0.297	0.249	1
Enteroendocrine	RPAIN	0.000366995	0.49613926	0.462	0.462	1
Enteroendocrine	PCLO	1.69E-05	0.494815274	0.278	0.176	0.359530098
Enteroendocrine	HOOK3	0.001677144	0.49401412	0.297	0.229	1
Enteroendocrine	DHX36	9.85E-05	0.493861624	0.506	0.487	1
Enteroendocrine	KLHL24	1.95E-06	0.493024109	0.424	0.315	0.041386376
Enteroendocrine	ZSCAN16-AS1	2.41E-13	0.491976157	0.259	0.1	5.13E-09
Enteroendocrine	HERPUD1	0.006685584	0.491200069	0.608	0.652	1
Enteroendocrine	FOS	8.52E-05	0.489853255	0.911	0.911	1
Enteroendocrine	GNAI2	1.48E-07	0.488877748	0.348	0.215	0.003150321
Enteroendocrine	CFLAR	4.14E-05	0.486903797	0.513	0.474	0.880093276
Enteroendocrine	SRP14	8.45E-08	0.486267823	0.88	0.936	0.001796819
Enteroendocrine	PCDHB14	4.95E-25	0.486074045	0.291	0.079	1.05E-20
Enteroendocrine	SON	6.94E-06	0.4824435	0.709	0.781	0.14759934
Enteroendocrine	MIF	1.11E-06	0.480729143	0.873	0.88	0.023499135
Enteroendocrine	MAP4K4	7.51E-06	0.479106803	0.342	0.231	0.159720094
Enteroendocrine	MAP1LC3A	0.003542417	0.478415111	0.443	0.424	1
Enteroendocrine	FAM133B	1.29E-05	0.477475104	0.601	0.612	0.274032599
Enteroendocrine	SRSF11	1.78E-07	0.477307118	0.696	0.696	0.003786226
Enteroendocrine	SEC61B	5.85E-06	0.477118218	0.797	0.857	0.124384871
Enteroendocrine	C3orf14	2.20E-10	0.475799277	0.297	0.147	4.67E-06

Enteroendocrine	SMARCA5	0.009484637	0.475524317	0.456	0.503	1
Enteroendocrine	SRP9	8.67E-07	0.47548204	0.747	0.822	0.018443711
Enteroendocrine	GCC2	5.68E-06	0.475441693	0.722	0.745	0.120750905
Enteroendocrine	LUC7L	8.91E-05	0.474596975	0.342	0.256	1
Enteroendocrine	GTF2I	0.000161559	0.474593192	0.627	0.634	1
Enteroendocrine	PTS	0.002854539	0.472079497	0.5	0.486	1
Enteroendocrine	SEC62	1.39E-09	0.470007704	0.785	0.789	2.95E-05
Enteroendocrine	ETNK1	0.001878351	0.469096435	0.468	0.444	1
Enteroendocrine	GRINA	3.30E-05	0.467342686	0.411	0.323	0.700821812
Enteroendocrine	DUSP10	4.23E-12	0.466955332	0.266	0.109	8.99E-08
Enteroendocrine	ZNF32	8.44E-07	0.465990086	0.392	0.292	0.017937824
Enteroendocrine	WSB1	0.000759272	0.465806387	0.595	0.62	1
Enteroendocrine	PSAP	2.27E-08	0.463452793	0.804	0.843	0.00048251
Enteroendocrine	DOCK8	1.02E-35	0.460840841	0.266	0.05	2.17E-31
Enteroendocrine	FTL	1.39E-08	0.460706465	0.968	0.965	0.000295028
Enteroendocrine	KLHL23	1.55E-05	0.46068907	0.405	0.318	0.32879945
Enteroendocrine	BAG6	0.002749692	0.460651837	0.462	0.455	1
Enteroendocrine	OAZ2	3.33E-06	0.458914041	0.43	0.337	0.070843434
Enteroendocrine	PLK2	2.05E-10	0.458822303	0.278	0.129	4.37E-06
Enteroendocrine	LFNG	2.13E-05	0.458547775	0.335	0.235	0.452531702
Enteroendocrine	VPS28	6.82E-07	0.457757761	0.709	0.786	0.0145035
Enteroendocrine	MAN1A1	5.48E-10	0.457495395	0.361	0.193	1.16E-05
Enteroendocrine	MBIP	0.005283018	0.455008932	0.354	0.315	1
Enteroendocrine	AKAP9	2.12E-06	0.453574425	0.734	0.793	0.045018349
Enteroendocrine	PNRC1	1.25E-06	0.452127261	0.652	0.615	0.026684064
Enteroendocrine	FUS	3.29E-06	0.451804598	0.766	0.763	0.0698943
Enteroendocrine	HIPK2	0.001165036	0.451659206	0.38	0.342	1
Enteroendocrine	FAM89B	0.000752148	0.450913809	0.348	0.29	1
Enteroendocrine	NDUFB4	8.74E-06	0.450126061	0.785	0.873	0.185830625
Enteroendocrine	DGKZ	0.001148966	0.449611107	0.342	0.287	1
Enteroendocrine	UBXN1	7.73E-05	0.448795534	0.677	0.722	1
Enteroendocrine	SSR2	5.24E-06	0.448442497	0.715	0.818	0.111327632
Enteroendocrine	NKTR	0.003690138	0.446199318	0.544	0.555	1
Enteroendocrine	LRRC42	0.001567268	0.4461363	0.253	0.184	1
Enteroendocrine	CHPF	8.95E-06	0.444929689	0.386	0.29	0.190332154
Enteroendocrine	SEZ6L2	0.002105549	0.444464492	0.354	0.301	1
Enteroendocrine	CHD6	7.11E-09	0.443542768	0.386	0.235	0.000151275
Enteroendocrine	CHD3	0.000111479	0.443322666	0.373	0.293	1
Enteroendocrine	AAK1	6.80E-05	0.442942173	0.354	0.265	1
Enteroendocrine	SCOC	0.002392584	0.441806674	0.487	0.495	1
Enteroendocrine	ACTR1A	0.000564295	0.440871866	0.361	0.301	1
Enteroendocrine	H2AFY	0.00023317	0.439871389	0.722	0.814	1
Enteroendocrine	HNRNPU	0.000167696	0.436594148	0.747	0.822	1

Enteroendocrine	EML4	0.007443673	0.436376063	0.487	0.53	1
Enteroendocrine	GUCY2C	0.004984053	0.434992043	0.361	0.322	1
Enteroendocrine	SERINC1	0.00215259	0.433109841	0.418	0.391	1
Enteroendocrine	RBM4	0.000145768	0.432408206	0.348	0.262	1
Enteroendocrine	CHD4	0.00056523	0.43138762	0.513	0.496	1
Enteroendocrine	MORF4L1	0.000653821	0.431341328	0.665	0.772	1
Enteroendocrine	PPL	1.06E-11	0.430563102	0.259	0.106	2.26E-07
Enteroendocrine	C5orf24	0.004743798	0.43001349	0.405	0.398	1
Enteroendocrine	ZNF891	8.24E-18	0.429901581	0.304	0.107	1.75E-13
Enteroendocrine	BTF3L4	0.000229638	0.429567741	0.487	0.466	1
Enteroendocrine	KDM7A	2.46E-05	0.429361488	0.443	0.358	0.522187283
Enteroendocrine	PLEKHB1	2.76E-11	0.428645872	0.323	0.151	5.86E-07
Enteroendocrine	MTF2	0.00090315	0.428524995	0.392	0.353	1
Enteroendocrine	UBR5	0.000169962	0.428002686	0.38	0.313	1
Enteroendocrine	BRK1	4.94E-06	0.427718904	0.766	0.805	0.105085981
Enteroendocrine	RPL7L1	0.000218295	0.426771623	0.551	0.556	1
Enteroendocrine	RNF114	0.004316128	0.426745558	0.563	0.593	1
Enteroendocrine	CNPY2	0.001398833	0.426173768	0.639	0.713	1
Enteroendocrine	OTUD6B-AS1	8.54E-05	0.425950987	0.513	0.521	1
Enteroendocrine	RAB2A	0.002492215	0.425147379	0.709	0.823	1
Enteroendocrine	TEAD1	0.000403878	0.424213874	0.291	0.216	1
Enteroendocrine	MEAF6	0.004563494	0.423318917	0.513	0.528	1
Enteroendocrine	MT-ND6	0.00448312	0.422587301	0.62	0.667	1
Enteroendocrine	AGO3	0.00031195	0.421624462	0.304	0.222	1
Enteroendocrine	PDZRN3	4.24E-25	0.42149214	0.272	0.069	9.03E-21
Enteroendocrine	HID1	1.38E-10	0.41896039	0.342	0.176	2.93E-06
Enteroendocrine	MID1	0.001816724	0.418467802	0.285	0.219	1
Enteroendocrine	ILF3	0.002837801	0.418394561	0.57	0.603	1
Enteroendocrine	COBLL1	4.98E-07	0.415809253	0.278	0.159	0.010594869
Enteroendocrine	SHROOM3	0.001925347	0.415061916	0.519	0.516	1
Enteroendocrine	NONO	0.00923626	0.414348054	0.557	0.619	1
Enteroendocrine	NIPSNAP1	4.32E-06	0.4141274	0.386	0.288	0.091849045
Enteroendocrine	TERF1	0.005455284	0.412625249	0.424	0.409	1
Enteroendocrine	CSNK2A1	0.000371439	0.409975153	0.525	0.528	1
Enteroendocrine	AC245297.3	5.67E-05	0.409748551	0.342	0.254	1
Enteroendocrine	MAGED2	9.21E-05	0.408526508	0.424	0.352	1
Enteroendocrine	MAD2L2	0.00026952	0.408062506	0.297	0.22	1
Enteroendocrine	NEAT1	0.001319687	0.407566515	0.911	0.951	1
Enteroendocrine	DYNLL1	4.62E-05	0.407381851	0.829	0.913	0.981885442
Enteroendocrine	WSB2	0.000169827	0.406934411	0.399	0.332	1
Enteroendocrine	SYNRG	0.000117207	0.403478852	0.316	0.23	1
Enteroendocrine	NPDC1	1.69E-06	0.403427437	0.759	0.662	0.035845993
Enteroendocrine	CCNL1	2.37E-05	0.40333802	0.709	0.733	0.503209772

Enteroendocrine	TPCN1	2.94E-06	0.402196207	0.31	0.194	0.062563976
Enteroendocrine	TULP4	1.50E-05	0.401816559	0.342	0.24	0.319904806
Enteroendocrine	HSD17B10	0.000694978	0.399857674	0.62	0.633	1
Enteroendocrine	TTC14	0.001939843	0.397820754	0.361	0.304	1
Enteroendocrine	CSDE1	0.00861826	0.395652407	0.671	0.788	1
Enteroendocrine	RSRC2	0.00191705	0.395511428	0.589	0.629	1
Enteroendocrine	GDI1	2.82E-05	0.393959908	0.304	0.203	0.599933285
Enteroendocrine	MCF2L	1.00E-09	0.393093333	0.278	0.13	2.13E-05
Enteroendocrine	PPP1R14C	0.006050178	0.392424308	0.285	0.226	1
Enteroendocrine	HBP1	0.000481864	0.391694772	0.354	0.284	1
Enteroendocrine	RAP2B	0.005029239	0.390482816	0.272	0.212	1
Enteroendocrine	RDH11	5.53E-05	0.38921157	0.43	0.364	1
Enteroendocrine	NSD1	0.004763	0.386937812	0.392	0.361	1
Enteroendocrine	FSD1L	3.30E-18	0.386127249	0.259	0.08	7.01E-14
Enteroendocrine	RHBDD2	0.000558753	0.385462489	0.411	0.367	1
Enteroendocrine	EIF4G2	2.03E-05	0.385281848	0.722	0.841	0.43149811
Enteroendocrine	POLR2K	0.00375937	0.384956976	0.614	0.669	1
Enteroendocrine	PLXNB1	0.001303724	0.384587675	0.304	0.234	1
Enteroendocrine	ATP6V0E1	0.000197361	0.383564802	0.759	0.844	1
Enteroendocrine	MORF4L2	0.000457656	0.383417727	0.62	0.684	1
Enteroendocrine	TBCC	0.00010708	0.382725804	0.323	0.242	1
Enteroendocrine	DNAJB6	0.0021626	0.38236117	0.582	0.659	1
Enteroendocrine	TBCA	4.78E-05	0.381885626	0.766	0.825	1
Enteroendocrine	JMY	1.99E-11	0.380517982	0.278	0.122	4.24E-07
Enteroendocrine	PRAC1	0.001976704	0.379278201	0.222	0.397	1
Enteroendocrine	SC5D	0.009560816	0.378857636	0.291	0.236	1
Enteroendocrine	NELFCD	0.008187999	0.377648444	0.424	0.415	1
Enteroendocrine	DNMT3A	1.46E-05	0.377599179	0.297	0.199	0.311044227
Enteroendocrine	RNF8	0.001583843	0.377556886	0.304	0.242	1
Enteroendocrine	DAAM1	0.007664812	0.376002661	0.437	0.42	1
Enteroendocrine	TMED4	0.003934346	0.375503059	0.57	0.621	1
Enteroendocrine	UBE2W	0.001198323	0.375396436	0.335	0.278	1
Enteroendocrine	CTNNBIP1	0.001501736	0.374950787	0.424	0.386	1
Enteroendocrine	CRYZ	3.29E-05	0.374438373	0.278	0.179	0.698594367
Enteroendocrine	SLC4A7	4.26E-05	0.37292247	0.291	0.194	0.905942478
Enteroendocrine	GCA	0.00031148	0.371148797	0.405	0.339	1
Enteroendocrine	RAB3D	0.000625284	0.369455155	0.297	0.23	1
Enteroendocrine	NAAA	2.61E-05	0.369160176	0.361	0.259	0.555753724
Enteroendocrine	DYNC1LI2	0.000713426	0.368715236	0.57	0.588	1
Enteroendocrine	GPBP1	0.000729279	0.36839581	0.582	0.633	1
Enteroendocrine	PHC2	0.002108694	0.365190257	0.304	0.242	1
Enteroendocrine	ARL3	4.91E-05	0.363396652	0.411	0.338	1
Enteroendocrine	TNPO1	0.008599314	0.359401583	0.418	0.407	1

Enteroendocrine	AZGP1	0.001513787	0.358380385	0.323	0.246	1
Enteroendocrine	AC097376.2	0.005615163	0.358078625	0.259	0.198	1
Enteroendocrine	MBOAT2	0.004242506	0.357459467	0.323	0.271	1
Enteroendocrine	TMEM134	0.001161468	0.357304062	0.544	0.542	1
Enteroendocrine	HNRNPDL	0.007754971	0.356089918	0.652	0.71	1
Enteroendocrine	CYSTM1	0.000188523	0.354496978	0.829	0.877	1
Enteroendocrine	SLC35E2B	8.84E-05	0.352837468	0.285	0.19	1
Enteroendocrine	SET	0.007526336	0.350088696	0.797	0.819	1
Enteroendocrine	YPEL5	0.001149775	0.349585719	0.437	0.385	1
Enteroendocrine	REEP3	0.004298402	0.346505167	0.468	0.468	1
Enteroendocrine	PHF6	0.008014056	0.344068586	0.316	0.27	1
Enteroendocrine	FAM174B	4.40E-17	0.342719777	0.278	0.091	9.36E-13
Enteroendocrine	BRAF	3.46E-05	0.342345366	0.31	0.208	0.734828479
Enteroendocrine	SGCB	0.007403822	0.34169924	0.285	0.241	1
Enteroendocrine	ABCA5	0.004039739	0.340701629	0.329	0.27	1
Enteroendocrine	YTHDC1	0.007047998	0.338924601	0.494	0.486	1
Enteroendocrine	FBXO32	0.000207577	0.335201325	0.38	0.285	1
Enteroendocrine	YPEL2	0.000283214	0.334069787	0.278	0.187	1
Enteroendocrine	MACF1	0.002769221	0.333361782	0.392	0.349	1
Enteroendocrine	PCGF3	0.001500645	0.332842335	0.285	0.211	1
Enteroendocrine	TRAK1	0.000446657	0.331414673	0.348	0.276	1
Enteroendocrine	RSPRY1	0.000419724	0.327106017	0.367	0.305	1
Enteroendocrine	FBXO21	0.00743322	0.323437848	0.354	0.322	1
Enteroendocrine	WDR6	3.93E-05	0.32342937	0.367	0.278	0.835364667
Enteroendocrine	RTL8A	0.000107203	0.321057791	0.342	0.244	1
Enteroendocrine	ITGB8	0.000100912	0.316167384	0.272	0.178	1
Enteroendocrine	MACO1	0.002879261	0.314610347	0.323	0.265	1
Enteroendocrine	SMAD5	0.000532364	0.312387126	0.411	0.348	1
Enteroendocrine	MRPS21	0.000435571	0.312330085	0.57	0.507	1
Enteroendocrine	GSTA4	1.85E-05	0.311546444	0.278	0.177	0.393408075
Enteroendocrine	RBM5	0.000940634	0.309957731	0.437	0.394	1
Enteroendocrine	DYNC1I2	0.00921678	0.30851746	0.627	0.703	1
Enteroendocrine	UFC1	0.004138247	0.307239298	0.709	0.798	1
Enteroendocrine	P4HA2	0.004828757	0.306701046	0.304	0.241	1
Enteroendocrine	ERV3-1	1.07E-06	0.305782786	0.285	0.165	0.022685489
Enteroendocrine	ZFAND5	0.001691012	0.30556111	0.551	0.553	1
Enteroendocrine	HNRNPK	0.00199171	0.305350345	0.785	0.865	1
Enteroendocrine	FAM172A	0.000464976	0.304087387	0.304	0.225	1
Enteroendocrine	COX6C	0.000766127	0.303684694	0.949	0.942	1
Enteroendocrine	ZSWIM6	0.000778366	0.302711302	0.259	0.179	1
Enteroendocrine	SLC44A1	0.007825448	0.301546108	0.646	0.707	1
Enteroendocrine	ZNF3	0.000302481	0.299886955	0.335	0.255	1
Enteroendocrine	WRB	0.000143846	0.299417264	0.297	0.21	1

Enteroendocrine	NDUFB11	0.000450088	0.294900194	0.791	0.843	1
Enteroendocrine	KLK11	4.46E-07	0.292508278	0.285	0.148	0.009477745
Enteroendocrine	MAPK8	0.000302143	0.288040932	0.272	0.188	1
Enteroendocrine	DIP2B	0.001544852	0.288025438	0.259	0.188	1
Enteroendocrine	WDR26	0.003305444	0.286304805	0.323	0.266	1
Enteroendocrine	KDM3A	0.001286571	0.286277594	0.253	0.176	1
Enteroendocrine	PDRG1	0.008172852	0.284252394	0.278	0.223	1
Enteroendocrine	ABI2	0.004358471	0.283750889	0.291	0.231	1
Enteroendocrine	UBALD1	0.005302951	0.282297691	0.259	0.199	1
Enteroendocrine	ABCB1	0.001145351	0.281225268	0.278	0.192	1
Enteroendocrine	CDKN2B	5.31E-06	0.28043099	0.259	0.14	0.112945903
Enteroendocrine	EIF4A2	0.000373052	0.270530318	0.81	0.852	1
Enteroendocrine	ATP8A1	0.00679291	0.269209377	0.259	0.201	1
Enteroendocrine	ARMC1	0.001494451	0.26681958	0.354	0.295	1
Enteroendocrine	SUMO1	0.00423112	0.256639392	0.684	0.767	1
Enteroendocrine	DYNC1LI1	0.009052942	0.251117217	0.323	0.267	1
Enteroendocrine	TUT4	0.003445307	0.250994627	0.266	0.203	1
Goblet	ZG16	0	7.632730207	0.971	0.242	0
Goblet	GSN	3.49E-306	4.813671537	0.979	0.56	7.41E-302
Goblet	ANKRD36C	0	4.527531408	0.916	0.268	0
Goblet	FCGBP	0	4.373432051	0.975	0.384	0
Goblet	MUC2	0	4.351143862	0.979	0.236	0
Goblet	MUC13	2.70E-257	3.157214655	0.967	0.721	5.74E-253
Goblet	MUC1	5.27E-274	3.028692788	0.961	0.586	1.12E-269
Goblet	TFF1	3.67E-95	2.809163488	0.69	0.41	7.81E-91
Goblet	CD177	0	2.725231439	0.801	0.088	0
Goblet	MALAT1	1.48E-246	2.661631725	1	0.994	3.14E-242
Goblet	HRASLS2	0	2.549537328	0.774	0.053	0
Goblet	TCIM	6.23E-178	2.50839686	0.737	0.305	1.32E-173
Goblet	SPATS2L	6.25E-237	2.444266844	0.943	0.634	1.33E-232
Goblet	ENTPD8	0	2.358714983	0.821	0.162	0
Goblet	LYPD8	0	2.323055696	0.848	0.149	0
Goblet	DST	9.42E-204	2.313071308	0.895	0.547	2.00E-199
Goblet	TSPAN1	2.41E-235	2.244247028	0.975	0.735	5.13E-231
Goblet	CEACAM5	1.60E-230	2.073388186	0.973	0.867	3.41E-226
Goblet	TENT5A	2.58E-172	2.03117722	0.774	0.361	5.49E-168
Goblet	ISG20	1.32E-236	1.985073671	0.717	0.192	2.80E-232
Goblet	MXD1	5.58E-199	1.966780788	0.768	0.293	1.19E-194
Goblet	LIPH	2.22E-143	1.908281423	0.805	0.523	4.73E-139
Goblet	SYTL2	1.54E-145	1.854611559	0.747	0.413	3.27E-141
Goblet	PLAC8	2.58E-180	1.843867594	0.86	0.401	5.50E-176
Goblet	CEACAM6	3.92E-161	1.838704374	0.864	0.456	8.33E-157
Goblet	HLA-E	3.46E-182	1.807013338	0.949	0.865	7.35E-178

Goblet	ISG15	1.07E-109	1.803591135	0.754	0.433	2.28E-105
Goblet	LMO7	1.63E-108	1.794432663	0.762	0.548	3.47E-104
Goblet	HLA-B	7.41E-202	1.772608274	0.994	0.977	1.58E-197
Goblet	QSOX1	1.48E-148	1.755476262	0.799	0.49	3.15E-144
Goblet	HLA-A	2.29E-216	1.732708401	0.994	0.978	4.86E-212
Goblet	LGALS9C	0	1.687984255	0.585	0.067	0
Goblet	TM4SF5	1.50E-117	1.679088624	0.61	0.265	3.20E-113
Goblet	MLLT3	1.50E-120	1.677263961	0.702	0.412	3.20E-116
Goblet	SCNN1A	8.23E-146	1.670975367	0.708	0.327	1.75E-141
Goblet	RASEF	5.42E-117	1.655992669	0.628	0.299	1.15E-112
Goblet	CDC42EP3	1.04E-162	1.655511537	0.62	0.208	2.22E-158
Goblet	IFI27	2.54E-179	1.649078092	0.988	0.969	5.40E-175
Goblet	HLA-C	7.21E-175	1.638482541	0.982	0.969	1.53E-170
Goblet	TRIM31	2.68E-122	1.638214399	0.725	0.362	5.69E-118
Goblet	DHRS9	0	1.63469412	0.6	0.055	0
Goblet	MINPP1	2.63E-156	1.612808536	0.567	0.173	5.59E-152
Goblet	TUBB2A	1.36E-136	1.604959506	0.573	0.197	2.89E-132
Goblet	LGALS9B	0	1.602705253	0.577	0.075	0
Goblet	SECTM1	0	1.597892146	0.546	0.066	0
Goblet	SDCBP2	3.03E-161	1.595942086	0.782	0.342	6.44E-157
Goblet	TPSG1	0	1.579111556	0.595	0.076	0
Goblet	SMIM6	1.06E-222	1.576429439	0.559	0.113	2.25E-218
Goblet	MUC4	8.98E-96	1.570407984	0.764	0.546	1.91E-91
Goblet	SMIM14	3.20E-103	1.55861776	0.762	0.603	6.81E-99
Goblet	REP15	1.72E-252	1.554492788	0.575	0.096	3.67E-248
Goblet	CDHR5	3.60E-135	1.539125781	0.762	0.382	7.65E-131
Goblet	TP53INP2	3.18E-161	1.52929732	0.54	0.139	6.77E-157
Goblet	RAB27A	1.61E-145	1.50862949	0.573	0.188	3.42E-141
Goblet	AREG	6.05E-127	1.507582292	0.836	0.466	1.29E-122
Goblet	LPIN2	8.63E-88	1.485228076	0.542	0.256	1.84E-83
Goblet	SAT1	1.05E-157	1.480223724	0.965	0.949	2.22E-153
Goblet	CDHR2	1.24E-273	1.478728446	0.602	0.098	2.64E-269
Goblet	CLDN4	3.01E-152	1.476744741	0.971	0.943	6.40E-148
Goblet	HLA-F	1.69E-79	1.47655949	0.708	0.536	3.59E-75
Goblet	SELENOP	5.26E-180	1.475882146	0.848	0.387	1.12E-175
Goblet	B2M	5.29E-190	1.471441012	0.998	0.997	1.13E-185
Goblet	SMIM5	0	1.461937583	0.503	0.039	0
Goblet	GDPD3	7.42E-196	1.458056014	0.499	0.099	1.58E-191
Goblet	PHGR1	2.32E-142	1.452445852	0.979	0.937	4.93E-138
Goblet	CAPN8	2.39E-112	1.446135694	0.634	0.31	5.08E-108
Goblet	BCAS1	4.37E-93	1.445030744	0.624	0.337	9.28E-89
Goblet	FBXO32	6.94E-105	1.43523027	0.6	0.272	1.48E-100
Goblet	SAMD9	9.32E-174	1.430291088	0.587	0.152	1.98E-169

Goblet	CFDP1	8.72E-123	1.423416464	0.844	0.704	1.86E-118
Goblet	JUND	6.42E-100	1.404793596	0.817	0.72	1.37E-95
Goblet	UBB	1.11E-126	1.399110515	0.949	0.96	2.35E-122
Goblet	NEDD4L	1.55E-76	1.395380097	0.616	0.391	3.31E-72
Goblet	PLAUR	7.78E-101	1.383473891	0.569	0.236	1.65E-96
Goblet	CCDC68	2.97E-118	1.367045831	0.565	0.212	6.31E-114
Goblet	HIST1H1C	2.57E-88	1.357051856	0.653	0.354	5.47E-84
Goblet	SH3BGRL3	2.15E-126	1.348387512	0.926	0.905	4.57E-122
Goblet	TMC5	1.57E-73	1.340358468	0.749	0.674	3.34E-69
Goblet	PARM1	3.56E-83	1.330267325	0.655	0.423	7.58E-79
Goblet	ZG16B	6.78E-57	1.322343396	0.647	0.533	1.44E-52
Goblet	ATF3	4.44E-80	1.318116454	0.745	0.518	9.45E-76
Goblet	ABHD3	1.12E-86	1.317374565	0.661	0.429	2.38E-82
Goblet	RARRES3	5.97E-97	1.31244617	0.628	0.292	1.27E-92
Goblet	IL3RA	0	1.310812786	0.45	0.024	0
Goblet	MLPH	8.05E-74	1.309351927	0.587	0.357	1.71E-69
Goblet	KLF4	2.11E-79	1.297255528	0.76	0.62	4.49E-75
Goblet	TFF3	3.35E-128	1.293438827	0.984	0.92	7.13E-124
Goblet	VSIG2	2.08E-89	1.291136671	0.774	0.585	4.42E-85
Goblet	MGLL	1.04E-83	1.288187102	0.737	0.596	2.22E-79
Goblet	POLD4	5.13E-63	1.285166744	0.628	0.461	1.09E-58
Goblet	NTHL1	1.62E-17	1.246084061	0.501	0.502	3.45E-13
Goblet	DDIT4	1.38E-18	1.244826561	0.435	0.356	2.93E-14
Goblet	CLDN7	7.96E-116	1.240890449	0.93	0.902	1.69E-111
Goblet	TXNIP	3.52E-75	1.238626034	0.793	0.701	7.48E-71
Goblet	CDKN1A	3.73E-64	1.225632712	0.602	0.38	7.93E-60
Goblet	TSPAN3	4.67E-81	1.204785496	0.774	0.724	9.92E-77
Goblet	DDIT3	1.55E-29	1.203891792	0.446	0.314	3.29E-25
Goblet	RIOK3	3.29E-68	1.202478181	0.647	0.463	6.99E-64
Goblet	FXYD3	7.04E-116	1.201447695	0.973	0.97	1.50E-111
Goblet	CYP3A5	2.42E-61	1.191471731	0.708	0.594	5.16E-57
Goblet	FFAR4	4.09E-110	1.188379341	0.47	0.146	8.70E-106
Goblet	CLDN23	3.86E-93	1.18179149	0.47	0.16	8.20E-89
Goblet	AMN	9.77E-69	1.170912759	0.702	0.541	2.08E-64
Goblet	TRANK1	2.27E-202	1.152658989	0.423	0.064	4.83E-198
Goblet	LINC00342	3.79E-163	1.148503018	0.388	0.068	8.07E-159
Goblet	INSR	4.46E-29	1.14782691	0.532	0.444	9.48E-25
Goblet	AC093277.1	0	1.145823244	0.351	0.002	0
Goblet	TCF7L2	3.56E-45	1.14499064	0.602	0.509	7.56E-41
Goblet	CREB3L1	1.39E-57	1.144425705	0.604	0.428	2.95E-53
Goblet	KCNK1	9.94E-56	1.144179382	0.546	0.354	2.11E-51
Goblet	WSB1	9.20E-46	1.141942147	0.663	0.618	1.96E-41
Goblet	F2RL1	8.67E-59	1.136212546	0.528	0.313	1.84E-54

Goblet	LINC01559	4.60E-130	1.126372239	0.441	0.106	9.79E-126
Goblet	LAMA3	3.39E-57	1.124334121	0.396	0.167	7.20E-53
Goblet	GPR153	2.80E-149	1.121262936	0.423	0.089	5.94E-145
Goblet	S100P	3.39E-42	1.113022817	0.737	0.639	7.22E-38
Goblet	RAB11FIP1	3.89E-57	1.108799624	0.747	0.715	8.27E-53
Goblet	ANKRD12	1.05E-48	1.108181511	0.639	0.54	2.23E-44
Goblet	KLF2	5.29E-58	1.108144772	0.355	0.134	1.13E-53
Goblet	MYO6	8.75E-36	1.104422082	0.639	0.638	1.86E-31
Goblet	SERPINA1	7.13E-153	1.100280726	0.772	0.283	1.52E-148
Goblet	UBC	6.84E-123	1.093460869	0.949	0.944	1.45E-118
Goblet	SYTL5	0	1.09319625	0.386	0.025	0
Goblet	HIST1H2AC	6.16E-52	1.089287345	0.556	0.355	1.31E-47
Goblet	GCNT3	8.68E-67	1.088346982	0.489	0.22	1.85E-62
Goblet	ATF4	6.33E-59	1.078307982	0.729	0.697	1.35E-54
Goblet	EREG	2.24E-55	1.076571068	0.37	0.141	4.76E-51
Goblet	NEAT1	2.10E-123	1.068938538	0.975	0.95	4.47E-119
Goblet	MT-ND4L	1.70E-83	1.055557496	0.899	0.876	3.62E-79
Goblet	MYO15B	2.50E-102	1.055276071	0.46	0.142	5.33E-98
Goblet	P2RX4	1.08E-48	1.053568124	0.478	0.28	2.30E-44
Goblet	SDR16C5	5.29E-80	1.05355781	0.38	0.123	1.12E-75
Goblet	RNF213	6.31E-16	1.050390835	0.476	0.465	1.34E-11
Goblet	GLUL	9.05E-40	1.050077934	0.684	0.695	1.92E-35
Goblet	TLE4	6.74E-32	1.04884687	0.441	0.295	1.43E-27
Goblet	ARL14	1.85E-52	1.045643219	0.513	0.297	3.93E-48
Goblet	RASD1	2.93E-64	1.03884725	0.374	0.13	6.23E-60
Goblet	HPGD	1.61E-71	1.03779272	0.522	0.238	3.41E-67
Goblet	AOC1	1.06E-63	1.035883213	0.69	0.501	2.26E-59
Goblet	GPA33	1.28E-53	1.035784786	0.663	0.53	2.73E-49
Goblet	BEST2	1.13E-155	1.032360113	0.312	0.043	2.39E-151
Goblet	TP53INP1	1.77E-115	1.025628833	0.405	0.101	3.77E-111
Goblet	YPEL3	5.23E-35	1.020582266	0.45	0.294	1.11E-30
Goblet	NAAA	2.07E-48	1.019308762	0.456	0.252	4.40E-44
Goblet	OPTN	2.17E-46	1.015442707	0.581	0.438	4.61E-42
Goblet	KLF6	4.86E-58	1.014246543	0.836	0.816	1.03E-53
Goblet	CDH1	8.53E-44	1.012411718	0.696	0.717	1.81E-39
Goblet	SMIM31	7.27E-35	1.000746608	0.526	0.402	1.55E-30
Goblet	B3GALT5	8.27E-75	0.994501286	0.405	0.139	1.76E-70
Goblet	CEACAM1	5.57E-37	0.983969829	0.509	0.333	1.18E-32
Goblet	DDX60	5.03E-56	0.977866238	0.343	0.129	1.07E-51
Goblet	EZR	5.46E-54	0.977713826	0.739	0.714	1.16E-49
Goblet	MUC12	3.04E-57	0.975357358	0.782	0.611	6.47E-53
Goblet	NT5C3A	6.37E-26	0.972732261	0.569	0.575	1.35E-21
Goblet	EIF2AK3	1.81E-91	0.970069451	0.361	0.099	3.84E-87

Goblet	AL121944.1	2.58E-17	0.969652662	0.306	0.2	5.49E-13
Goblet	FUT3	4.49E-30	0.969652456	0.53	0.454	9.54E-26
Goblet	ITLN1	6.59E-210	0.969479915	0.585	0.114	1.40E-205
Goblet	KRT20	1.52E-78	0.967487189	0.731	0.422	3.23E-74
Goblet	DEPP1	4.77E-54	0.965183436	0.296	0.098	1.01E-49
Goblet	UGCG	2.41E-35	0.9628597	0.409	0.243	5.13E-31
Goblet	LAMB3	7.54E-29	0.962610679	0.48	0.362	1.60E-24
Goblet	CTSE	8.14E-112	0.958930561	0.353	0.072	1.73E-107
Goblet	ACSS2	2.33E-25	0.950320597	0.446	0.344	4.96E-21
Goblet	PNPLA2	4.68E-40	0.936726458	0.499	0.341	9.94E-36
Goblet	MISP	6.61E-45	0.935438483	0.702	0.658	1.40E-40
Goblet	NUPR1	0.006627125	0.931317472	0.407	0.615	1
Goblet	CNKSR3	7.32E-41	0.930363937	0.363	0.175	1.56E-36
Goblet	SLC2A13	1.08E-57	0.929610126	0.366	0.144	2.29E-53
Goblet	LSR	2.14E-59	0.929409748	0.799	0.833	4.55E-55
Goblet	NEDD9	6.30E-21	0.924628216	0.464	0.395	1.34E-16
Goblet	CLDN8	5.98E-93	0.923888006	0.337	0.082	1.27E-88
Goblet	MT-CO1	1.33E-121	0.923783741	1	0.993	2.82E-117
Goblet	TBX10	9.69E-245	0.918467519	0.267	0.017	2.06E-240
Goblet	CPM	2.26E-46	0.914930506	0.368	0.164	4.80E-42
Goblet	PTPRH	5.36E-59	0.912896538	0.386	0.156	1.14E-54
Goblet	ASS1	1.70E-12	0.912026598	0.542	0.573	3.61E-08
Goblet	MTMR11	5.46E-32	0.910691613	0.478	0.349	1.16E-27
Goblet	DPYSL2	4.73E-56	0.905801965	0.38	0.157	1.01E-51
Goblet	SPDEF	3.46E-47	0.903024818	0.386	0.177	7.37E-43
Goblet	C19orf33	4.65E-76	0.899597009	0.899	0.842	9.90E-72
Goblet	N4BP2L2	2.02E-53	0.899372205	0.782	0.787	4.29E-49
Goblet	SPECC1	1.91E-38	0.899337374	0.39	0.213	4.06E-34
Goblet	SQSTM1	2.97E-37	0.898880444	0.737	0.796	6.32E-33
Goblet	PRSS8	9.35E-21	0.894197796	0.589	0.622	1.99E-16
Goblet	DNM2	1.96E-30	0.894061239	0.573	0.533	4.18E-26
Goblet	FOXA3	1.89E-19	0.889999817	0.474	0.43	4.03E-15
Goblet	SERINC2	1.13E-48	0.889432049	0.766	0.789	2.40E-44
Goblet	TMEM173	1.94E-12	0.88486748	0.339	0.274	4.13E-08
Goblet	CLIC5	9.84E-61	0.883846833	0.378	0.144	2.09E-56
Goblet	LRP10	7.65E-30	0.88004644	0.624	0.632	1.63E-25
Goblet	SHROOM3	9.57E-20	0.877621778	0.517	0.516	2.04E-15
Goblet	CLSTN1	4.02E-21	0.873664155	0.513	0.49	8.54E-17
Goblet	SLC7A11	2.10E-69	0.869727332	0.296	0.082	4.46E-65
Goblet	RAB3B	4.06E-216	0.869181021	0.302	0.027	8.63E-212
Goblet	IRF6	3.17E-28	0.868560968	0.435	0.313	6.75E-24
Goblet	TMEM59	1.24E-68	0.868035023	0.918	0.931	2.63E-64
Goblet	BDKRB2	1.00E-142	0.865049155	0.316	0.049	2.13E-138

Goblet	FAM107B	1.15E-31	0.863483743	0.378	0.22	2.45E-27
Goblet	MIER3	3.97E-44	0.860094801	0.343	0.149	8.45E-40
Goblet	INAVA	1.14E-29	0.856454798	0.487	0.373	2.42E-25
Goblet	AC015912.3	1.18E-26	0.853555689	0.292	0.149	2.51E-22
Goblet	ZNF292	4.44E-15	0.852669214	0.478	0.469	9.43E-11
Goblet	SUSD6	6.92E-30	0.851976142	0.345	0.192	1.47E-25
Goblet	RHOC	4.07E-59	0.848097055	0.819	0.823	8.66E-55
Goblet	RNF103	4.34E-21	0.847726504	0.388	0.284	9.23E-17
Goblet	PRSS3	2.40E-31	0.844585507	0.743	0.754	5.11E-27
Goblet	APOL1	2.86E-52	0.843898636	0.331	0.123	6.08E-48
Goblet	ACHE	1.13E-85	0.843761508	0.31	0.074	2.41E-81
Goblet	CBLB	7.21E-33	0.838104198	0.347	0.186	1.53E-28
Goblet	SMPDL3A	9.44E-31	0.838008436	0.452	0.307	2.01E-26
Goblet	SLC17A4	4.83E-81	0.834197918	0.347	0.095	1.03E-76
Goblet	FER1L6	0	0.833292664	0.281	0.007	0
Goblet	EPS8	1.08E-19	0.822602975	0.569	0.598	2.30E-15
Goblet	DYNLRB1	1.84E-23	0.814812878	0.68	0.8	3.92E-19
Goblet	DYRK2	3.74E-21	0.813553212	0.431	0.345	7.95E-17
Goblet	IRF7	9.51E-20	0.812829067	0.32	0.204	2.02E-15
Goblet	BBC3	1.16E-16	0.811711065	0.388	0.309	2.46E-12
Goblet	CTTNBP2NL	3.21E-13	0.809723656	0.411	0.373	6.83E-09
Goblet	RNF19A	1.92E-17	0.809252737	0.372	0.281	4.09E-13
Goblet	MIR194-2HG	5.23E-17	0.804089024	0.388	0.298	1.11E-12
Goblet	BAIAP2L1	5.61E-14	0.800536962	0.446	0.427	1.19E-09
Goblet	TMEM56	5.33E-22	0.799509681	0.353	0.231	1.13E-17
Goblet	NLN	2.82E-25	0.795028422	0.331	0.189	6.00E-21
Goblet	RAP2B	4.38E-16	0.793693397	0.308	0.209	9.31E-12
Goblet	VIPR1	3.69E-32	0.791146214	0.345	0.182	7.85E-28
Goblet	PLS1	1.43E-19	0.786774168	0.556	0.575	3.03E-15
Goblet	KLK1	5.84E-43	0.785700696	0.608	0.425	1.24E-38
Goblet	IL32	1.27E-45	0.781471313	0.786	0.707	2.70E-41
Goblet	PTGER4	2.72E-12	0.778845173	0.324	0.255	5.78E-08
Goblet	TNFRSF21	2.52E-13	0.776405151	0.366	0.301	5.36E-09
Goblet	OTUD1	1.29E-28	0.774178731	0.333	0.183	2.74E-24
Goblet	TNFRSF1A	8.54E-20	0.772562411	0.483	0.43	1.82E-15
Goblet	PLA2G10	3.44E-23	0.771689735	0.437	0.318	7.32E-19
Goblet	YPEL2	9.87E-21	0.770920764	0.304	0.183	2.10E-16
Goblet	RRBP1	7.60E-39	0.770104877	0.782	0.841	1.62E-34
Goblet	ADM	2.95E-30	0.766871216	0.405	0.236	6.28E-26
Goblet	VILL	2.42E-15	0.763521661	0.366	0.285	5.14E-11
Goblet	HK2	4.75E-12	0.757558129	0.374	0.313	1.01E-07
Goblet	BACH1	1.80E-23	0.754588209	0.296	0.164	3.83E-19
Goblet	LINC01133	1.84E-24	0.751807492	0.485	0.364	3.90E-20

Goblet	ITM2C	3.28E-56	0.750514979	0.889	0.848	6.97E-52
Goblet	RBP4	1.58E-17	0.750459236	0.318	0.197	3.36E-13
Goblet	DHDDS	4.96E-23	0.747770207	0.345	0.218	1.05E-18
Goblet	FAM177B	6.20E-177	0.740132951	0.259	0.024	1.32E-172
Goblet	TIMP2	1.32E-112	0.73809408	0.259	0.04	2.81E-108
Goblet	CTNND1	9.83E-17	0.736561468	0.585	0.67	2.09E-12
Goblet	ATP6V1G1	3.18E-33	0.732354817	0.762	0.852	6.77E-29
Goblet	EPS8L1	4.72E-16	0.732205755	0.335	0.238	1.00E-11
Goblet	PLXNB2	1.47E-11	0.731307788	0.421	0.404	3.13E-07
Goblet	DUSP1	5.84E-24	0.729204574	0.719	0.695	1.24E-19
Goblet	ABCA5	2.57E-18	0.72788491	0.368	0.266	5.47E-14
Goblet	GCC2	1.27E-23	0.726709318	0.674	0.748	2.71E-19
Goblet	MAN1A1	9.95E-21	0.72515727	0.312	0.19	2.12E-16
Goblet	GABARAPL1	2.43E-35	0.722481948	0.292	0.126	5.17E-31
Goblet	SLC16A9	3.51E-29	0.721183194	0.32	0.169	7.47E-25
Goblet	CYTH1	6.82E-21	0.718889162	0.3	0.178	1.45E-16
Goblet	USP53	3.14E-11	0.718531144	0.47	0.482	6.67E-07
Goblet	RFLNA	3.82E-44	0.717050679	0.294	0.113	8.13E-40
Goblet	SSFA2	2.70E-09	0.716573086	0.441	0.461	5.74E-05
Goblet	CDKN2B-AS1	9.35E-60	0.714821399	0.343	0.11	1.99E-55
Goblet	SLC44A4	1.33E-32	0.712925653	0.719	0.749	2.82E-28
Goblet	BTNL3	6.30E-42	0.712209462	0.308	0.124	1.34E-37
Goblet	MEF2D	2.15E-19	0.710798374	0.265	0.148	4.57E-15
Goblet	TICAM1	5.46E-20	0.710278118	0.259	0.139	1.16E-15
Goblet	HSPA2	6.60E-39	0.708400023	0.277	0.107	1.40E-34
Goblet	RETREG1	0.000386036	0.70196923	0.39	0.443	1
Goblet	TMEM63B	1.70E-12	0.697488104	0.32	0.245	3.61E-08
Goblet	RSRP1	5.15E-16	0.697348967	0.585	0.651	1.09E-11
Goblet	SPTBN1	5.50E-14	0.687443138	0.585	0.673	1.17E-09
Goblet	STX19	5.19E-19	0.687094539	0.304	0.188	1.10E-14
Goblet	PAQR8	2.06E-10	0.686394147	0.388	0.357	4.39E-06
Goblet	XRN1	1.02E-07	0.683489644	0.279	0.23	0.002162575
Goblet	CAPN5	7.07E-11	0.682517351	0.39	0.355	1.50E-06
Goblet	S100A6	2.47E-58	0.681962276	0.992	0.996	5.25E-54
Goblet	MT-ND5	1.91E-65	0.681600195	0.986	0.97	4.05E-61
Goblet	PDGFA	2.76E-16	0.678709739	0.361	0.262	5.86E-12
Goblet	MAPK3	1.20E-07	0.678078366	0.446	0.488	0.002546966
Goblet	DNPEP	1.86E-05	0.677423482	0.466	0.578	0.395363028
Goblet	SCGB2A1	3.84E-28	0.675798033	0.267	0.123	8.17E-24
Goblet	TMBIM1	1.60E-08	0.674077593	0.476	0.507	0.000341039
Goblet	ATOH1	1.53E-46	0.671162998	0.287	0.094	3.25E-42
Goblet	MAX	1.83E-07	0.669974167	0.374	0.367	0.003884528
Goblet	RBM47	4.47E-06	0.668333252	0.53	0.677	0.095112362

Goblet	SPINT1	2.24E-20	0.664440812	0.641	0.71	4.75E-16
Goblet	IL1R2	7.12E-29	0.663588147	0.265	0.119	1.51E-24
Goblet	APOL6	1.47E-07	0.662629402	0.366	0.358	0.003132826
Goblet	EPS8L3	2.39E-07	0.662555104	0.45	0.504	0.005087736
Goblet	GRN	6.35E-25	0.662310629	0.741	0.814	1.35E-20
Goblet	IL2RG	2.81E-13	0.661393736	0.335	0.243	5.97E-09
Goblet	TAGLN2	1.41E-24	0.658960813	0.727	0.809	3.00E-20
Goblet	OFD1	1.19E-05	0.658743806	0.431	0.497	0.252299751
Goblet	UACA	1.31E-05	0.653826636	0.343	0.344	0.279395913
Goblet	JOSD1	3.90E-11	0.650717113	0.368	0.323	8.29E-07
Goblet	SEMA6A	1.39E-18	0.649839724	0.322	0.21	2.95E-14
Goblet	ABCB1	8.94E-20	0.64127436	0.312	0.188	1.90E-15
Goblet	SLC16A3	9.01E-16	0.637935661	0.431	0.356	1.92E-11
Goblet	ABHD17C	1.15E-05	0.63714674	0.4	0.448	0.245404313
Goblet	RBCK1	8.16E-05	0.635749812	0.491	0.635	1
Goblet	FEM1C	5.43E-16	0.635714937	0.296	0.196	1.15E-11
Goblet	ERBIN	1.39E-06	0.63183956	0.351	0.338	0.029520528
Goblet	FAM214A	1.02E-21	0.626646903	0.269	0.144	2.17E-17
Goblet	CD2AP	0.000346651	0.626387125	0.474	0.613	1
Goblet	KCNK6	1.62E-08	0.621682246	0.349	0.316	0.000344586
Goblet	B4GALT1	1.08E-05	0.617307557	0.388	0.395	0.229886917
Goblet	SIPA1L3	1.85E-09	0.616941657	0.283	0.22	3.93E-05
Goblet	IRF1	0.001053472	0.616104062	0.38	0.412	1
Goblet	RNASE1	7.64E-12	0.607332919	0.464	0.414	1.62E-07
Goblet	CDKN2B	1.25E-26	0.604437182	0.283	0.135	2.66E-22
Goblet	STX3	1.84E-06	0.602815126	0.39	0.406	0.039187841
Goblet	SLC5A3	1.03E-06	0.602074984	0.273	0.23	0.021826795
Goblet	CLIC4	1.03E-22	0.601386296	0.271	0.142	2.18E-18
Goblet	SCIN	6.14E-27	0.600654897	0.329	0.172	1.30E-22
Goblet	MIR22HG	1.55E-22	0.596982078	0.279	0.147	3.29E-18
Goblet	SDC4	3.81E-07	0.596560622	0.468	0.532	0.008112014
Goblet	GALE	1.01E-07	0.596313698	0.468	0.531	0.002144628
Goblet	FAM3D	3.05E-38	0.594993037	0.893	0.878	6.48E-34
Goblet	IFNGR2	4.04E-05	0.594104413	0.446	0.522	0.859905151
Goblet	CLTB	5.29E-20	0.592382957	0.735	0.836	1.12E-15
Goblet	MYL12A	3.36E-42	0.591735398	0.875	0.937	7.15E-38
Goblet	APLP2	5.20E-16	0.590681094	0.655	0.758	1.11E-11
Goblet	VAMP8	1.76E-28	0.588805103	0.776	0.878	3.75E-24
Goblet	FAM114A1	3.33E-06	0.588438972	0.329	0.315	0.070918215
Goblet	CAMK2N1	3.45E-20	0.588253032	0.733	0.786	7.34E-16
Goblet	CACFD1	7.91E-13	0.583493469	0.267	0.179	1.68E-08
Goblet	TJP1	0.000700981	0.583244147	0.331	0.354	1
Goblet	HIST1H2BD	2.11E-06	0.582390116	0.285	0.241	0.044850593

Goblet	B3GNT5	9.24E-06	0.582312404	0.322	0.312	0.19642569
Goblet	CXCL16	0.006281926	0.582103858	0.333	0.37	1
Goblet	TMEM127	1.08E-08	0.580250302	0.287	0.232	0.000230572
Goblet	DHX32	2.76E-05	0.579271983	0.326	0.328	0.586085863
Goblet	MUC3A	0.005628077	0.578898141	0.431	0.54	1
Goblet	ATP2A3	4.27E-06	0.577723302	0.312	0.29	0.090726722
Goblet	GALNT5	4.73E-07	0.576522423	0.279	0.236	0.010068541
Goblet	SH3KBP1	1.22E-05	0.573200405	0.474	0.555	0.25861132
Goblet	AC254629.1	9.92E-08	0.572842898	0.292	0.243	0.002108593
Goblet	SELENOK	5.09E-10	0.570527413	0.604	0.752	1.08E-05
Goblet	ZNF655	0.000480981	0.568899933	0.318	0.33	1
Goblet	JMJD1C	0.001119642	0.568737168	0.423	0.516	1
Goblet	SPPL2A	0.001587953	0.568537478	0.413	0.496	1
Goblet	SNAP23	0.001013278	0.56610739	0.37	0.422	1
Goblet	MAST2	5.48E-15	0.565356128	0.271	0.172	1.17E-10
Goblet	TAPBP	1.79E-09	0.565336544	0.571	0.697	3.81E-05
Goblet	TNFSF10	1.54E-08	0.564892165	0.517	0.596	0.000328121
Goblet	KIF13B	2.70E-05	0.562499499	0.285	0.264	0.573150398
Goblet	GBP3	8.59E-06	0.552148291	0.306	0.283	0.182707681
Goblet	RASSF6	0.000103399	0.548264721	0.339	0.347	1
Goblet	FHL2	2.60E-10	0.545813562	0.563	0.604	5.52E-06
Goblet	TJP3	5.83E-05	0.541609621	0.39	0.426	1
Goblet	OGDH	8.11E-05	0.5378395	0.279	0.264	1
Goblet	YPEL5	0.000385432	0.537010369	0.355	0.387	1
Goblet	ELF3	1.75E-35	0.536017604	0.949	0.961	3.72E-31
Goblet	STXBP2	0.007006386	0.535008688	0.407	0.512	1
Goblet	PKIB	1.07E-29	0.532730304	0.417	0.24	2.27E-25
Goblet	JUP	0.00233762	0.532338443	0.476	0.626	1
Goblet	EPCAM	3.69E-35	0.530323968	0.971	0.984	7.85E-31
Goblet	UBE2B	6.41E-05	0.528673399	0.532	0.681	1
Goblet	APOBR	6.68E-05	0.528409088	0.304	0.289	1
Goblet	ST3GAL4	4.31E-06	0.52819236	0.269	0.223	0.091697021
Goblet	FURIN	1.47E-10	0.527836099	0.265	0.192	3.13E-06
Goblet	PDCD6IP	0.001417816	0.527011289	0.452	0.58	1
Goblet	BLOC1S1	2.80E-22	0.523638144	0.76	0.876	5.95E-18
Goblet	ZFP36	8.84E-11	0.5168749	0.68	0.788	1.88E-06
Goblet	CTNNA1	1.14E-05	0.515337357	0.548	0.705	0.242278217
Goblet	CAST	4.97E-15	0.515141309	0.704	0.854	1.06E-10
Goblet	COQ10B	9.75E-05	0.51491827	0.279	0.263	1
Goblet	CHMP2A	1.58E-12	0.513510532	0.663	0.822	3.37E-08
Goblet	DDR1	0.000523793	0.513451715	0.441	0.541	1
Goblet	SNX9	0.001353115	0.513150021	0.339	0.367	1
Goblet	PRUNE2	4.56E-06	0.508351397	0.292	0.254	0.096902532

Goblet	PPP1R15A	1.67E-06	0.503313015	0.536	0.64	0.035595098
Goblet	DAPK2	5.91E-05	0.501991015	0.281	0.262	1
Goblet	CCNL1	8.39E-06	0.499696789	0.585	0.739	0.178383509
Goblet	CHMP5	4.21E-06	0.495059348	0.536	0.681	0.089547175
Goblet	HEPACAM2	3.28E-30	0.493499043	0.322	0.14	6.98E-26
Goblet	CCNG2	0.000829235	0.484429877	0.253	0.237	1
Goblet	SRSF5	3.21E-10	0.472809243	0.651	0.795	6.83E-06
Goblet	ACTN4	2.75E-12	0.471073982	0.69	0.833	5.84E-08
Goblet	ZFAND2A	1.83E-06	0.471036383	0.251	0.201	0.038984112
Goblet	LLGL2	0.00228376	0.466805938	0.495	0.654	1
Goblet	ERBB3	0.00454206	0.460958038	0.522	0.696	1
Goblet	CTSS	6.80E-11	0.446331933	0.678	0.775	1.45E-06
Goblet	SMIM22	6.40E-22	0.436543625	0.869	0.951	1.36E-17
Goblet	TMEM54	2.08E-16	0.429421638	0.768	0.865	4.41E-12
Goblet	ST14	7.01E-07	0.412024667	0.667	0.836	0.014899304
Goblet	MARCKS	9.85E-08	0.41097873	0.665	0.819	0.00209359
Goblet	YWHAZ	3.23E-13	0.399489641	0.776	0.903	6.88E-09
Goblet	MT-ND3	5.15E-29	0.397674805	0.998	0.985	1.09E-24
Goblet	CIB1	7.18E-11	0.391804807	0.69	0.828	1.53E-06
Goblet	CD63	7.21E-22	0.374815442	0.916	0.966	1.53E-17
Goblet	ERRFI1	0.006463741	0.372848585	0.265	0.255	1
Goblet	PPP2CB	0.008207224	0.353660787	0.238	0.373	1
Goblet	SLC6A8	4.79E-05	0.352097561	0.292	0.249	1
Goblet	CLDN3	0.003043378	0.335052211	0.889	0.952	1
Goblet	CD151	0.000227727	0.331645184	0.682	0.855	1
Goblet	ATP10B	0.003865665	0.318152937	0.349	0.568	1
Goblet	GPBP1	0.001864016	0.314579633	0.386	0.643	1
Goblet	FAM120AOS	0.000501237	0.309853246	0.222	0.374	1
Goblet	H1FX	0.0073146	0.307278341	0.322	0.512	1
Goblet	GALNT3	7.10E-05	0.30343797	0.308	0.544	1
Goblet	ID3	1.29E-08	0.301766242	0.632	0.592	0.00027446
Goblet	SRI	3.15E-10	0.289344012	0.821	0.923	6.70E-06
Goblet	SLC38A2	0.00016958	0.286365221	0.269	0.46	1
Goblet	S100A13	0.002046176	0.285832393	0.378	0.623	1
Goblet	MYL12B	3.38E-12	0.282704328	0.885	0.941	7.19E-08
Goblet	ETV6	0.001905647	0.281949105	0.195	0.318	1
Goblet	CTSD	0.001988919	0.278408669	0.647	0.826	1
Goblet	VAMP2	0.000796688	0.274989334	0.324	0.547	1
Goblet	MYO7B	0.006507739	0.273902755	0.168	0.263	1
Goblet	TES	0.007062711	0.273699986	0.333	0.531	1
Goblet	ADAM9	0.005720636	0.271986148	0.24	0.379	1
Goblet	CHIC2	0.006140292	0.271041997	0.17	0.266	1
Goblet	TAOK3	0.000568079	0.270634759	0.343	0.584	1

Goblet	UBA1	0.000771275	0.270469096	0.255	0.426	1
Goblet	MYO5B	0.008653944	0.26735626	0.269	0.426	1
Goblet	MIA3	0.005097684	0.266538354	0.22	0.346	1
Goblet	OGFR	0.000747987	0.266400078	0.187	0.312	1
Goblet	SEL1L3	0.001804501	0.266041329	0.242	0.4	1
Goblet	MED13	0.000515434	0.265462343	0.193	0.324	1
Goblet	SPAG9	0.002311724	0.264967574	0.228	0.371	1
Goblet	CCDC107	3.87E-05	0.264807039	0.271	0.476	0.822178085
Goblet	ZNF91	5.71E-06	0.264313943	0.201	0.37	0.121504989
Goblet	MAP3K11	0.003226411	0.262725113	0.234	0.379	1
Goblet	NUMB	1.44E-05	0.255475052	0.22	0.397	0.307204157
Goblet	ARHGAP27	8.30E-05	0.253745871	0.189	0.331	1
Goblet	SLK	0.000124382	0.253141176	0.257	0.444	1
Goblet	ARHGAP26	0.004359365	0.252889365	0.201	0.318	1
Malignant	MMP7	7.59E-159	2.104364199	0.418	0.189	1.61E-154
Malignant	NDRG1	1.01E-119	1.2264625	0.606	0.431	2.15E-115
Malignant	CSTB	2.81E-278	1.1805842	0.929	0.839	5.98E-274
Malignant	LYZ	3.86E-87	1.166830192	0.744	0.568	8.20E-83
Malignant	NEAT1	1.02E-126	1.069341171	0.915	0.96	2.18E-122
Malignant	LDHA	1.13E-170	1.038621642	0.906	0.88	2.41E-166
Malignant	S100A11	6.09E-228	1.027244047	0.952	0.888	1.29E-223
Malignant	ZFAS1	4.32E-281	0.990596127	0.913	0.847	9.18E-277
Malignant	ERO1A	1.77E-149	0.985647712	0.617	0.401	3.77E-145
Malignant	TFF1	2.11E-68	0.962479293	0.556	0.387	4.48E-64
Malignant	IGLC2	7.30E-71	0.94328373	0.507	0.323	1.55E-66
Malignant	CST3	2.37E-113	0.940835096	0.941	0.979	5.03E-109
Malignant	VEGFA	1.25E-212	0.930423573	0.636	0.361	2.66E-208
Malignant	TNFRSF12A	1.37E-137	0.929505777	0.57	0.344	2.92E-133
Malignant	SCD	6.18E-127	0.927384458	0.576	0.354	1.31E-122
Malignant	C4orf3	6.34E-147	0.921329979	0.819	0.753	1.35E-142
Malignant	PLA2G16	7.09E-225	0.902490737	0.628	0.333	1.51E-220
Malignant	TIMP1	1.49E-159	0.881655186	0.715	0.502	3.17E-155
Malignant	C8orf33	4.98E-120	0.872672665	0.623	0.434	1.06E-115
Malignant	HSPA1A	0.00059582	0.864214382	0.405	0.414	1
Malignant	TM4SF1	2.48E-105	0.863625432	0.565	0.363	5.28E-101
Malignant	PRAP1	0	0.861975831	0.646	0.265	0
Malignant	PI3	3.82E-21	0.845999222	0.267	0.19	8.13E-17
Malignant	HSPA1B	6.85E-81	0.825826142	0.6	0.452	1.46E-76
Malignant	IGHA1	2.14E-53	0.81958045	0.628	0.473	4.56E-49
Malignant	FXYD5	0	0.798160086	0.721	0.317	0
Malignant	SOX4	8.08E-180	0.790620229	0.857	0.713	1.72E-175
Malignant	CA9	8.72E-175	0.786864074	0.457	0.191	1.85E-170
Malignant	PGK1	5.67E-86	0.753787745	0.816	0.755	1.21E-81

Malignant	C6orf48	1.68E-166	0.752900271	0.779	0.646	3.56E-162
Malignant	BST2	2.59E-159	0.752706467	0.46	0.197	5.51E-155
Malignant	ADIRF	1.44E-51	0.741332356	0.527	0.409	3.07E-47
Malignant	TPT1	3.25E-115	0.73971187	0.985	0.993	6.90E-111
Malignant	IGKC	7.05E-53	0.73858268	0.684	0.543	1.50E-48
Malignant	ATP1B1	7.16E-48	0.725809204	0.896	0.893	1.52E-43
Malignant	AREG	7.84E-44	0.720363149	0.586	0.455	1.67E-39
Malignant	IGHA2	1.03E-63	0.709342274	0.355	0.196	2.18E-59
Malignant	AZGP1	0	0.709207382	0.551	0.17	0
Malignant	TGFBI	1.07E-116	0.704877149	0.656	0.421	2.27E-112
Malignant	AC020656.1	2.05E-47	0.690506895	0.297	0.173	4.36E-43
Malignant	TMEM123	1.34E-126	0.688350217	0.858	0.78	2.86E-122
Malignant	TACSTD2	2.84E-83	0.676184291	0.369	0.19	6.04E-79
Malignant	FHL2	2.73E-33	0.665135075	0.636	0.593	5.81E-29
Malignant	SLC11A2	4.05E-81	0.643435233	0.437	0.27	8.62E-77
Malignant	RPS18	7.43E-156	0.635172684	0.997	0.97	1.58E-151
Malignant	PABPC1	4.93E-165	0.631532103	0.944	0.904	1.05E-160
Malignant	RPS6	1.77E-39	0.62294067	0.985	0.975	3.77E-35
Malignant	RBM39	6.24E-130	0.620446129	0.842	0.834	1.33E-125
Malignant	HSPB1	7.24E-80	0.620109278	0.839	0.755	1.54E-75
Malignant	PERP	1.99E-151	0.616428423	0.88	0.835	4.23E-147
Malignant	NPC2	6.69E-98	0.615107151	0.818	0.831	1.42E-93
Malignant	SLC6A8	5.43E-99	0.612584688	0.406	0.212	1.15E-94
Malignant	PMEPA1	2.16E-89	0.609353788	0.563	0.379	4.59E-85
Malignant	CFD	2.15E-36	0.600586185	0.367	0.255	4.57E-32
Malignant	MAL2	2.64E-77	0.596620012	0.757	0.699	5.62E-73
Malignant	CCND1	8.54E-76	0.596295512	0.651	0.522	1.82E-71
Malignant	FAM13A	3.12E-28	0.594824998	0.369	0.293	6.63E-24
Malignant	FTL	1.92E-85	0.585836346	0.968	0.964	4.08E-81
Malignant	RPS10	1.98E-17	0.578199386	0.943	0.924	4.20E-13
Malignant	EIF4A2	4.48E-41	0.577790162	0.825	0.858	9.52E-37
Malignant	CEACAM6	1.17E-86	0.568716844	0.633	0.432	2.50E-82
Malignant	CEBPB	2.24E-83	0.566237112	0.699	0.591	4.77E-79
Malignant	RPL7	7.35E-136	0.565134356	0.983	0.944	1.56E-131
Malignant	SMIM24	4.99E-216	0.562331961	0.471	0.177	1.06E-211
Malignant	RPS25	3.13E-155	0.53322594	0.977	0.946	6.67E-151
Malignant	RPL39	1.40E-124	0.532636765	0.984	0.958	2.97E-120
Malignant	RPS20	5.44E-219	0.527429847	0.968	0.943	1.16E-214
Malignant	SQLE	2.53E-51	0.527355417	0.502	0.362	5.38E-47
Malignant	UFM1	5.57E-57	0.52661513	0.668	0.622	1.19E-52
Malignant	GPRC5A	5.35E-55	0.524180367	0.692	0.596	1.14E-50
Malignant	CLIC1	3.99E-137	0.524161707	0.927	0.948	8.49E-133
Malignant	SORL1	3.61E-65	0.519402725	0.605	0.484	7.67E-61

Malignant	JCHAIN	3.08E-41	0.513813261	0.414	0.279	6.55E-37
Malignant	PLIN2	3.43E-95	0.512686062	0.528	0.338	7.30E-91
Malignant	RPL8	6.05E-162	0.510574016	0.989	0.972	1.29E-157
Malignant	SEC61G	9.11E-95	0.510423262	0.904	0.881	1.94E-90
Malignant	SAT1	8.07E-57	0.509170087	0.948	0.951	1.72E-52
Malignant	MIF	8.55E-90	0.509148674	0.917	0.871	1.82E-85
Malignant	P4HA1	3.76E-159	0.507824843	0.446	0.2	8.00E-155
Malignant	TMSB10	7.57E-150	0.507167189	0.992	0.98	1.61E-145
Malignant	HSPH1	9.10E-42	0.503046285	0.571	0.476	1.94E-37
Malignant	EIF3E	4.77E-71	0.500753769	0.842	0.813	1.01E-66
Malignant	CLTA	3.85E-67	0.499299206	0.852	0.862	8.18E-63
Malignant	HSP90AB1	6.82E-100	0.497908162	0.921	0.879	1.45E-95
Malignant	PRSS3	2.88E-83	0.497880787	0.788	0.745	6.13E-79
Malignant	NUPR1	1.07E-05	0.49783636	0.556	0.619	0.22808662
Malignant	RPS21	5.43E-190	0.497249107	0.966	0.918	1.15E-185
Malignant	SLPI	8.89E-05	0.493127031	0.552	0.585	1
Malignant	RPL30	1.97E-173	0.488865592	0.977	0.948	4.18E-169
Malignant	TSC22D1	3.05E-70	0.48570185	0.823	0.787	6.48E-66
Malignant	EEF1D	4.76E-112	0.485325511	0.921	0.913	1.01E-107
Malignant	IFI6	8.91E-14	0.484120295	0.406	0.338	1.90E-09
Malignant	EIF2S2	8.31E-76	0.48263571	0.823	0.769	1.77E-71
Malignant	DDAH2	8.89E-79	0.481575206	0.712	0.625	1.89E-74
Malignant	YWHAZ	1.85E-105	0.481308569	0.88	0.903	3.94E-101
Malignant	COMMD6	4.81E-56	0.478267407	0.876	0.853	1.02E-51
Malignant	EIF3H	9.40E-89	0.471988001	0.822	0.81	2.00E-84
Malignant	ZFAND5	5.87E-89	0.471339911	0.659	0.527	1.25E-84
Malignant	POLR1D	1.01E-71	0.470692672	0.795	0.755	2.14E-67
Malignant	DDIT4	4.83E-34	0.470127381	0.452	0.336	1.03E-29
Malignant	SLC2A1	9.47E-100	0.469905924	0.354	0.17	2.01E-95
Malignant	HMGA1	8.56E-27	0.469474781	0.744	0.707	1.82E-22
Malignant	NEU1	2.34E-98	0.468810726	0.555	0.375	4.98E-94
Malignant	POMP	5.52E-84	0.461027085	0.894	0.883	1.17E-79
Malignant	GDF15	2.67E-15	0.452872154	0.566	0.487	5.68E-11
Malignant	PPDPF	9.64E-124	0.451771253	0.952	0.973	2.05E-119
Malignant	C12orf75	3.73E-56	0.451463664	0.753	0.649	7.92E-52
Malignant	C4orf48	6.97E-47	0.450399899	0.753	0.677	1.48E-42
Malignant	KRT18	3.42E-77	0.449662259	0.968	0.985	7.27E-73
Malignant	CBWD5	1.53E-70	0.448848088	0.518	0.376	3.26E-66
Malignant	PRSS22	4.42E-108	0.445484552	0.326	0.141	9.40E-104
Malignant	ANXA3	3.24E-28	0.443297218	0.598	0.524	6.90E-24
Malignant	GTF3A	2.61E-69	0.441723676	0.726	0.63	5.56E-65
Malignant	BMP4	6.19E-59	0.44022513	0.334	0.191	1.32E-54
Malignant	PKM	1.11E-56	0.438786645	0.858	0.799	2.36E-52

Malignant	CD55	1.69E-06	0.436510765	0.47	0.444	0.035913305
Malignant	IFITM1	2.71E-14	0.435419077	0.617	0.562	5.76E-10
Malignant	TMSB4X	2.14E-33	0.434738447	0.994	0.999	4.56E-29
Malignant	RPS19	1.87E-109	0.431904995	0.994	0.971	3.98E-105
Malignant	RND3	1.36E-62	0.431301435	0.494	0.336	2.89E-58
Malignant	TRAM1	1.38E-54	0.42705684	0.648	0.568	2.94E-50
Malignant	PYGB	3.82E-39	0.425804374	0.532	0.449	8.13E-35
Malignant	SLC39A4	1.86E-63	0.421063382	0.657	0.565	3.96E-59
Malignant	SLC3A2	1.51E-38	0.420613664	0.541	0.445	3.22E-34
Malignant	S100A10	7.20E-47	0.420143599	0.966	0.979	1.53E-42
Malignant	GAPDH	5.94E-50	0.42007714	0.982	0.977	1.26E-45
Malignant	TRMT112	3.48E-72	0.418316192	0.806	0.73	7.40E-68
Malignant	ZNF706	1.50E-78	0.416907158	0.809	0.805	3.20E-74
Malignant	RPS27	5.68E-123	0.413660524	0.992	0.988	1.21E-118
Malignant	TMPRSS4	4.22E-85	0.413041346	0.583	0.404	8.96E-81
Malignant	LGALS3	2.03E-20	0.412811373	0.941	0.969	4.32E-16
Malignant	BCL2L1	3.71E-97	0.408071406	0.519	0.323	7.89E-93
Malignant	BNIP3	5.16E-109	0.403527396	0.26	0.092	1.10E-104
Malignant	ANXA2	6.16E-14	0.403516643	0.899	0.903	1.31E-09
Malignant	MLXIPL	3.95E-127	0.403309665	0.392	0.18	8.41E-123
Malignant	CDKN2A	6.02E-102	0.400767072	0.29	0.115	1.28E-97
Malignant	MTHFD2	1.17E-17	0.398778789	0.445	0.365	2.49E-13
Malignant	DUSP6	3.59E-84	0.394600028	0.461	0.269	7.62E-80
Malignant	RAC1	4.18E-88	0.394097162	0.927	0.94	8.90E-84
Malignant	S100A4	1.75E-22	0.391803733	0.356	0.262	3.72E-18
Malignant	ANXA4	7.88E-32	0.390619775	0.785	0.765	1.68E-27
Malignant	CXADR	2.30E-41	0.390127462	0.748	0.724	4.90E-37
Malignant	PHLDA1	1.55E-22	0.389999476	0.414	0.326	3.29E-18
Malignant	OSTF1	1.91E-67	0.389188718	0.596	0.478	4.06E-63
Malignant	ZFP36L1	1.23E-29	0.388640625	0.769	0.714	2.63E-25
Malignant	ADM	1.35E-39	0.386777429	0.345	0.217	2.88E-35
Malignant	ACTB	1.95E-23	0.385276706	0.975	0.989	4.15E-19
Malignant	KIF5B	1.82E-41	0.384644047	0.808	0.807	3.87E-37
Malignant	SERPINB6	2.97E-43	0.384334597	0.79	0.759	6.31E-39
Malignant	GUK1	1.96E-63	0.380150714	0.875	0.884	4.16E-59
Malignant	CEBPD	2.58E-38	0.378679169	0.643	0.557	5.48E-34
Malignant	MALL	2.98E-89	0.378554879	0.524	0.298	6.33E-85
Malignant	CPNE1	9.45E-72	0.377173736	0.567	0.422	2.01E-67
Malignant	SERPINE2	5.14E-179	0.376592501	0.276	0.071	1.09E-174
Malignant	EDN1	1.70E-43	0.373635229	0.408	0.269	3.61E-39
Malignant	BRD2	2.86E-26	0.372732362	0.604	0.573	6.08E-22
Malignant	TOMM20	1.32E-57	0.372604975	0.778	0.753	2.80E-53
Malignant	RPL27A	1.37E-105	0.369781125	0.984	0.973	2.91E-101

Malignant	CLDN2	2.96E-15	0.369776215	0.369	0.301	6.30E-11
Malignant	SULT2B1	6.92E-155	0.369646291	0.362	0.137	1.47E-150
Malignant	EIF1	2.64E-28	0.369468099	0.946	0.974	5.62E-24
Malignant	TAF7	1.44E-32	0.369306672	0.703	0.672	3.05E-28
Malignant	OCIAD2	1.34E-25	0.368475394	0.814	0.821	2.85E-21
Malignant	CDH17	6.30E-40	0.368132035	0.784	0.778	1.34E-35
Malignant	TAX1BP1	4.74E-41	0.362693863	0.814	0.814	1.01E-36
Malignant	PHKG1	2.72E-13	0.362639231	0.273	0.209	5.78E-09
Malignant	RPL10	2.15E-80	0.361742947	0.992	0.981	4.58E-76
Malignant	DDIT3	9.15E-33	0.361161212	0.41	0.296	1.95E-28
Malignant	BLCAP	1.39E-71	0.35915266	0.55	0.393	2.96E-67
Malignant	DYNLRB1	2.65E-53	0.358521697	0.798	0.794	5.64E-49
Malignant	NFIB	4.88E-40	0.354645288	0.548	0.472	1.04E-35
Malignant	KRT19	8.47E-53	0.354629821	0.909	0.912	1.80E-48
Malignant	HSP90AA1	3.90E-64	0.354080146	0.929	0.919	8.30E-60
Malignant	RPL37	2.55E-83	0.353703895	0.974	0.944	5.42E-79
Malignant	EGLN3	1.44E-68	0.353384846	0.418	0.252	3.06E-64
Malignant	ERRFI1	4.37E-16	0.352825177	0.311	0.242	9.30E-12
Malignant	RPS3	8.82E-75	0.352179923	0.986	0.97	1.88E-70
Malignant	SELENOH	2.77E-37	0.349468096	0.838	0.84	5.89E-33
Malignant	RPL21	2.29E-38	0.348549285	0.988	0.97	4.86E-34
Malignant	GSTP1	8.01E-47	0.348150935	0.937	0.924	1.70E-42
Malignant	HNF4A	1.76E-23	0.3478134	0.559	0.477	3.74E-19
Malignant	TINAGL1	4.31E-30	0.34692149	0.586	0.501	9.17E-26
Malignant	ETV4	8.36E-143	0.345830757	0.388	0.163	1.78E-138
Malignant	SAP18	1.81E-37	0.344609908	0.865	0.876	3.84E-33
Malignant	YWHAB	3.32E-46	0.344400984	0.856	0.867	7.06E-42
Malignant	EPB41L2	3.47E-28	0.343848424	0.583	0.476	7.38E-24
Malignant	STAU1	3.48E-52	0.343735358	0.691	0.615	7.40E-48
Malignant	LY6E	5.61E-57	0.342715894	0.345	0.194	1.19E-52
Malignant	ATP5F1E	1.53E-42	0.342713917	0.957	0.96	3.26E-38
Malignant	TAF1D	5.91E-36	0.342386218	0.729	0.66	1.26E-31
Malignant	TRIB3	2.89E-139	0.342175219	0.266	0.082	6.14E-135
Malignant	RPS13	8.86E-52	0.335907215	0.972	0.93	1.88E-47
Malignant	CD46	2.98E-42	0.334764559	0.789	0.723	6.33E-38
Malignant	FAM177A1	9.81E-32	0.334626344	0.747	0.753	2.09E-27
Malignant	HSPA5	3.00E-07	0.334370127	0.744	0.744	0.006387629
Malignant	PLAUR	2.16E-17	0.333607811	0.312	0.234	4.59E-13
Malignant	AP3S1	2.80E-32	0.331840096	0.705	0.641	5.96E-28
Malignant	CNIH4	2.18E-54	0.331147562	0.667	0.55	4.64E-50
Malignant	RPS15A	9.44E-86	0.331020272	0.984	0.956	2.01E-81
Malignant	ALDOA	7.60E-32	0.330412532	0.581	0.492	1.62E-27
Malignant	SERPINB1	2.06E-30	0.328105543	0.807	0.804	4.37E-26

Malignant	AQP5	1.16E-125	0.327795095	0.262	0.08	2.48E-121
Malignant	C8orf59	1.69E-49	0.327738118	0.786	0.725	3.59E-45
Malignant	MACC1	1.84E-51	0.327538304	0.416	0.27	3.91E-47
Malignant	NACA	1.68E-89	0.327150368	0.955	0.921	3.57E-85
Malignant	CTSH	2.48E-31	0.326948452	0.507	0.396	5.27E-27
Malignant	PHF20L1	1.22E-56	0.325477619	0.534	0.392	2.60E-52
Malignant	UBE2R2	2.74E-42	0.32485879	0.555	0.47	5.83E-38
Malignant	AL133453.1	8.41E-88	0.323714551	0.279	0.123	1.79E-83
Malignant	INSIG1	1.14E-09	0.322529976	0.414	0.371	2.43E-05
Malignant	GPX4	2.91E-12	0.322416711	0.802	0.814	6.19E-08
Malignant	VSNL1	2.72E-72	0.320424583	0.366	0.203	5.78E-68
Malignant	SDCBP	5.04E-25	0.320352831	0.724	0.699	1.07E-20
Malignant	CNN2	2.04E-56	0.320155426	0.544	0.398	4.33E-52
Malignant	EIF5	2.56E-17	0.319468152	0.716	0.727	5.44E-13
Malignant	PRELID3B	4.95E-33	0.318883835	0.656	0.6	1.05E-28
Malignant	PAPOLA	8.43E-28	0.318058615	0.715	0.724	1.79E-23
Malignant	PHLDA2	3.16E-24	0.317888582	0.694	0.607	6.71E-20
Malignant	RPL28	1.36E-81	0.317190722	0.983	0.98	2.90E-77
Malignant	MYC	1.43E-33	0.31605914	0.578	0.472	3.03E-29
Malignant	PRPF6	4.78E-53	0.313933829	0.555	0.439	1.02E-48
Malignant	ARHGAP5	2.70E-33	0.312405853	0.57	0.497	5.75E-29
Malignant	PTTG1IP	9.87E-32	0.311982689	0.694	0.655	2.10E-27
Malignant	N4BP2L2	2.06E-09	0.311324796	0.739	0.799	4.37E-05
Malignant	DGKH	8.03E-39	0.309816728	0.368	0.262	1.71E-34
Malignant	RNF43	5.75E-18	0.309003487	0.638	0.573	1.22E-13
Malignant	EZR	3.00E-19	0.308847015	0.731	0.712	6.39E-15
Malignant	EIF6	1.95E-46	0.306378301	0.765	0.749	4.15E-42
Malignant	LAPTM4B	5.76E-41	0.306136032	0.575	0.458	1.23E-36
Malignant	INSIG2	1.16E-61	0.305207561	0.412	0.258	2.46E-57
Malignant	PLEC	2.84E-45	0.304478957	0.537	0.402	6.05E-41
Malignant	MSMO1	7.96E-16	0.30330397	0.399	0.332	1.69E-11
Malignant	RPL31	8.03E-65	0.303115216	0.982	0.955	1.71E-60
Malignant	SUB1	1.40E-50	0.302091207	0.89	0.889	2.98E-46
Malignant	HILPDA	3.99E-28	0.300427185	0.337	0.238	8.49E-24
Malignant	GID8	1.02E-42	0.29980044	0.586	0.481	2.16E-38
Malignant	OLFM4	0.001429087	0.299579921	0.686	0.625	1
Malignant	RAB6A	3.83E-46	0.298695975	0.6	0.493	8.15E-42
Malignant	EIF2S3	3.42E-25	0.296298001	0.648	0.605	7.27E-21
Malignant	MPHOSPH8	2.32E-12	0.295720514	0.617	0.614	4.92E-08
Malignant	SOX9	5.31E-19	0.294985124	0.627	0.57	1.13E-14
Malignant	CAPG	1.55E-19	0.294056084	0.68	0.629	3.30E-15
Malignant	RPS29	5.74E-43	0.292260511	0.989	0.979	1.22E-38
Malignant	MTDH	8.32E-33	0.291965312	0.776	0.779	1.77E-28

Malignant	C6orf132	1.18E-32	0.290954092	0.504	0.41	2.51E-28
Malignant	GRINA	3.96E-80	0.290858164	0.47	0.287	8.41E-76
Malignant	PSMA7	1.22E-35	0.289273646	0.883	0.881	2.60E-31
Malignant	RAB5IF	1.62E-23	0.288138702	0.733	0.691	3.44E-19
Malignant	HOXB7	2.46E-24	0.286679919	0.59	0.549	5.22E-20
Malignant	FAM49B	2.69E-51	0.286594091	0.54	0.4	5.71E-47
Malignant	B4GALT1	4.55E-26	0.286495753	0.479	0.374	9.67E-22
Malignant	MAP3K20	2.61E-24	0.285468076	0.386	0.296	5.55E-20
Malignant	SPINK1	8.27E-22	0.285330463	0.665	0.858	1.76E-17
Malignant	APLP2	1.86E-21	0.284888913	0.765	0.751	3.96E-17
Malignant	INTS6	3.05E-19	0.2842356	0.441	0.368	6.48E-15
Malignant	ITGA2	1.71E-26	0.282791315	0.448	0.349	3.63E-22
Malignant	LACTB2	4.11E-44	0.282771895	0.494	0.365	8.74E-40
Malignant	RAB4A	1.08E-29	0.282056602	0.675	0.654	2.29E-25
Malignant	PRR13	1.27E-46	0.281874699	0.865	0.88	2.70E-42
Malignant	PFDN2	5.27E-24	0.281245188	0.718	0.669	1.12E-19
Malignant	EIF4B	2.92E-28	0.28091488	0.747	0.754	6.20E-24
Malignant	FLOT1	1.57E-33	0.280784605	0.643	0.595	3.35E-29
Malignant	KDM5B	1.88E-52	0.280330849	0.491	0.352	3.99E-48
Malignant	AZIN1	6.43E-38	0.278590459	0.566	0.452	1.37E-33
Malignant	PPARG	1.14E-14	0.278185761	0.442	0.388	2.42E-10
Malignant	PDXK	2.17E-12	0.278005635	0.577	0.541	4.62E-08
Malignant	TM7SF2	2.62E-75	0.277903054	0.422	0.246	5.58E-71
Malignant	ROMO1	1.26E-17	0.27670653	0.849	0.853	2.68E-13
Malignant	VDAC1	3.93E-14	0.276488603	0.839	0.84	8.35E-10
Malignant	AGO2	5.58E-56	0.275905957	0.471	0.315	1.19E-51
Malignant	TOP1	1.01E-29	0.275878645	0.681	0.612	2.15E-25
Malignant	FTH1	2.14E-60	0.275234551	0.989	0.994	4.54E-56
Malignant	PPP1R10	3.98E-18	0.274877585	0.458	0.383	8.46E-14
Malignant	EFNB2	7.97E-21	0.274790436	0.451	0.384	1.70E-16
Malignant	NORAD	1.62E-20	0.273786661	0.67	0.638	3.45E-16
Malignant	PTPN12	5.74E-66	0.273600196	0.453	0.289	1.22E-61
Malignant	MMP24OS	6.55E-33	0.273263359	0.636	0.58	1.39E-28
Malignant	COL17A1	4.34E-51	0.272550462	0.316	0.182	9.24E-47
Malignant	RSL1D1	1.48E-29	0.271802096	0.731	0.696	3.15E-25
Malignant	IL32	1.79E-52	0.271751819	0.814	0.684	3.81E-48
Malignant	FAU	5.93E-33	0.271309493	0.967	0.968	1.26E-28
Malignant	TMEM45B	1.81E-33	0.268752623	0.652	0.584	3.85E-29
Malignant	PHF14	1.73E-15	0.268690404	0.635	0.592	3.68E-11
Malignant	ASPH	3.82E-25	0.268144502	0.652	0.604	8.12E-21
Malignant	NUDT4	6.61E-23	0.267878835	0.692	0.659	1.41E-18
Malignant	PPP1R15A	1.48E-12	0.267782796	0.649	0.632	3.15E-08
Malignant	RNF114	1.02E-39	0.267177691	0.654	0.577	2.17E-35

Malignant	CYP2S1	5.26E-65	0.266769209	0.341	0.188	1.12E-60
Malignant	RPL13	2.25E-46	0.266614065	0.994	0.961	4.77E-42
Malignant	CEMIP2	1.29E-34	0.266226784	0.516	0.403	2.75E-30
Malignant	ZMYND8	1.94E-30	0.265857199	0.53	0.427	4.12E-26
Malignant	NENF	8.43E-29	0.263227368	0.771	0.74	1.79E-24
Malignant	ARPC1B	2.10E-18	0.263204429	0.766	0.727	4.47E-14
Malignant	ITGB1	1.69E-07	0.263015904	0.746	0.744	0.003588986
Malignant	LPGAT1	1.35E-43	0.262001888	0.495	0.367	2.87E-39
Malignant	HOXB8	2.03E-44	0.26178178	0.378	0.252	4.32E-40
Malignant	ERGIC3	4.84E-32	0.261711301	0.802	0.82	1.03E-27
Malignant	HIST1H2BG	2.32E-57	0.261306819	0.267	0.132	4.94E-53
Malignant	DNAJA1	2.38E-18	0.260531887	0.721	0.711	5.05E-14
Malignant	CCDC85B	1.36E-33	0.259052576	0.556	0.445	2.90E-29
Malignant	PFDN4	1.84E-24	0.258536759	0.665	0.592	3.91E-20
Malignant	TNRC18	3.09E-38	0.258523362	0.415	0.298	6.56E-34
Malignant	SNX3	2.87E-27	0.258338819	0.776	0.775	6.11E-23
Malignant	TPM4	3.71E-10	0.258078404	0.726	0.726	7.89E-06
Malignant	RPS15	1.71E-10	0.258038006	0.987	0.976	3.63E-06
Malignant	CHMP5	3.67E-18	0.257778673	0.681	0.674	7.80E-14
Malignant	PLOD2	2.15E-30	0.256524293	0.263	0.165	4.57E-26
Malignant	BNIP3L	8.36E-39	0.255152283	0.51	0.386	1.78E-34
Malignant	IFITM3	0.00039575	0.254117244	0.784	0.755	1
Malignant	SF1	1.23E-13	0.253939407	0.672	0.667	2.62E-09
Malignant	ELOC	2.36E-32	0.253662239	0.768	0.72	5.02E-28
Malignant	RPL36A	2.67E-09	0.253409442	0.955	0.902	5.68E-05
Malignant	ZFAND1	4.37E-32	0.253273302	0.498	0.397	9.29E-28
Malignant	MYO6	2.04E-25	0.252327279	0.686	0.626	4.35E-21
Malignant	EMP1	6.73E-60	0.251903694	0.296	0.151	1.43E-55
Malignant	ELF3	3.18E-07	0.251233517	0.91	0.974	0.006766696
Malignant	RSF1	9.43E-29	0.250982826	0.622	0.554	2.01E-24
Malignant	MISP	2.22E-06	0.250039332	0.639	0.665	0.047187401
SPINK4+ CLCA1+ DEFA6+ REG4+	SPINK4	0	5.807760454	0.844	0.223	0
SPINK4+ CLCA1+ DEFA6+ REG4+	CLCA1	0	5.088687172	0.732	0.097	0
SPINK4+ CLCA1+ DEFA6+ REG4+	DEFA6	9.08E-187	4.191121525	0.268	0.047	1.93E-182
SPINK4+ CLCA1+ DEFA6+ REG4+	REG4	0	3.779272352	0.802	0.215	0
SPINK4+ CLCA1+ DEFA6+ REG4+	ITLN1	0	3.322085356	0.602	0.083	0
SPINK4+ CLCA1+ DEFA6+ REG4+	TFF3	0	3.182551248	0.998	0.914	0

SPINK4+ CLCA1+ DEFA6+ REG4+	SERPINA1	0	2.771270298	0.84	0.245	0
SPINK4+ CLCA1+ DEFA6+ REG4+	WFDC2	0	2.722310104	0.863	0.328	0
SPINK4+ CLCA1+ DEFA6+ REG4+	KLK1	0	2.194866324	0.892	0.383	0
SPINK4+ CLCA1+ DEFA6+ REG4+	FCGBP	0	2.068451108	0.942	0.351	0
SPINK4+ CLCA1+ DEFA6+ REG4+	MUC2	0	2.050628606	0.87	0.202	0
SPINK4+ CLCA1+ DEFA6+ REG4+	LRRC26	0	1.990036768	0.883	0.18	0
SPINK4+ CLCA1+ DEFA6+ REG4+	IFI6	0	1.900615901	0.775	0.306	0
SPINK4+ CLCA1+ DEFA6+ REG4+	KLK12	0	1.838340504	0.676	0.031	0
SPINK4+ CLCA1+ DEFA6+ REG4+	ST6GALNAC1	0	1.771067268	0.821	0.438	0
SPINK4+ CLCA1+ DEFA6+ REG4+	AGR2	3.70E-175	1.724853653	0.962	0.94	7.88E-171
SPINK4+ CLCA1+ DEFA6+ REG4+	IGFBP2	6.48E-182	1.700793093	0.766	0.508	1.38E-177
SPINK4+ CLCA1+ DEFA6+ REG4+	SPINK1	0	1.666284764	0.988	0.801	0
SPINK4+ CLCA1+ DEFA6+ REG4+	REP15	0	1.601840452	0.693	0.053	0
SPINK4+ CLCA1+ DEFA6+ REG4+	SELENOM	0	1.503280388	0.682	0.166	0
SPINK4+ CLCA1+ DEFA6+ REG4+	PCSK1	0	1.467703303	0.413	0.059	0
SPINK4+ CLCA1+ DEFA6+ REG4+	HES6	2.01E-279	1.424320957	0.788	0.395	4.27E-275
SPINK4+ CLCA1+ DEFA6+ REG4+	ATOH1	0	1.419851877	0.687	0.039	0
SPINK4+ CLCA1+ DEFA6+ REG4+	HEPACAM2	0	1.410268465	0.71	0.086	0
SPINK4+ CLCA1+ DEFA6+ REG4+	XBP1	8.83E-283	1.409460958	0.892	0.708	1.88E-278
SPINK4+ CLCA1+ DEFA6+ REG4+	L1TD1	1.81E-198	1.263717404	0.57	0.217	3.86E-194
SPINK4+ CLCA1+ DEFA6+ REG4+	KLK11	0	1.203348437	0.654	0.095	0
SPINK4+ CLCA1+ DEFA6+ REG4+	CPE	1.66E-275	1.181618818	0.448	0.097	3.53E-271

SPINK4+ CLCA1+ DEFA6+ REG4+	KIAA1324	6.86E-271	1.172328763	0.793	0.41	1.46E-266
SPINK4+ CLCA1+ DEFA6+ REG4+	NUCB2	2.19E-171	1.168736757	0.695	0.433	4.65E-167
SPINK4+ CLCA1+ DEFA6+ REG4+	RNASE1	3.91E-142	1.164776701	0.661	0.389	8.31E-138
SPINK4+ CLCA1+ DEFA6+ REG4+	FKBP11	1.58E-176	1.155288349	0.648	0.37	3.37E-172
SPINK4+ CLCA1+ DEFA6+ REG4+	SH3BGRL3	2.12E-214	1.134578338	0.955	0.901	4.51E-210
SPINK4+ CLCA1+ DEFA6+ REG4+	SSR4	1.11E-245	1.076251504	0.965	0.89	2.37E-241
SPINK4+ CLCA1+ DEFA6+ REG4+	TPSG1	0	1.040176371	0.551	0.048	0
SPINK4+ CLCA1+ DEFA6+ REG4+	RASD1	0	0.996935575	0.534	0.097	0
SPINK4+ CLCA1+ DEFA6+ REG4+	ANXA13	3.41E-285	0.99078268	0.607	0.182	7.25E-281
SPINK4+ CLCA1+ DEFA6+ REG4+	GMDS	8.34E-192	0.921568222	0.885	0.715	1.77E-187
SPINK4+ CLCA1+ DEFA6+ REG4+	SPDEF	0	0.914919721	0.626	0.138	0
SPINK4+ CLCA1+ DEFA6+ REG4+	GALNT8	0	0.857817276	0.489	0.031	0
SPINK4+ CLCA1+ DEFA6+ REG4+	HPCAL1	4.21E-194	0.851448564	0.711	0.403	8.96E-190
SPINK4+ CLCA1+ DEFA6+ REG4+	FABP2	4.01E-131	0.824068166	0.604	0.304	8.52E-127
SPINK4+ CLCA1+ DEFA6+ REG4+	RAP1GAP	0	0.81835842	0.514	0.069	0
SPINK4+ CLCA1+ DEFA6+ REG4+	C12orf57	4.08E-160	0.803447075	0.851	0.667	8.68E-156
SPINK4+ CLCA1+ DEFA6+ REG4+	SEC11C	8.73E-149	0.79590085	0.777	0.589	1.86E-144
SPINK4+ CLCA1+ DEFA6+ REG4+	CREB3L1	4.40E-174	0.789895219	0.715	0.405	9.35E-170
SPINK4+ CLCA1+ DEFA6+ REG4+	S100P	1.07E-97	0.776337197	0.796	0.626	2.28E-93
SPINK4+ CLCA1+ DEFA6+ REG4+	FAM3D	1.24E-109	0.767764889	0.92	0.874	2.63E-105
SPINK4+ CLCA1+ DEFA6+ REG4+	B3GNT6	0	0.739591926	0.45	0.021	0
SPINK4+ CLCA1+ DEFA6+ REG4+	DNAJC12	0	0.730448728	0.507	0.08	0

SPINK4+ CLCA1+ DEFA6+ REG4+	TSPAN13	8.66E-123	0.714786686	0.827	0.702	1.84E-118
SPINK4+ CLCA1+ DEFA6+ REG4+	ASRGL1	3.21E-136	0.704730648	0.657	0.388	6.82E-132
SPINK4+ CLCA1+ DEFA6+ REG4+	PDIA3	2.60E-74	0.696392451	0.87	0.822	5.53E-70
SPINK4+ CLCA1+ DEFA6+ REG4+	TSTA3	7.90E-131	0.696092094	0.85	0.68	1.68E-126
SPINK4+ CLCA1+ DEFA6+ REG4+	SLC50A1	1.23E-90	0.690528677	0.585	0.405	2.61E-86
SPINK4+ CLCA1+ DEFA6+ REG4+	DYRK4	6.41E-181	0.686175672	0.513	0.199	1.36E-176
SPINK4+ CLCA1+ DEFA6+ REG4+	CD63	6.15E-144	0.681724443	0.977	0.962	1.31E-139
SPINK4+ CLCA1+ DEFA6+ REG4+	GNE	1.66E-215	0.676082895	0.533	0.177	3.52E-211
SPINK4+ CLCA1+ DEFA6+ REG4+	TPM1	2.10E-142	0.674958116	0.943	0.895	4.47E-138
SPINK4+ CLCA1+ DEFA6+ REG4+	RAB26	0	0.674598941	0.358	0.036	0
SPINK4+ CLCA1+ DEFA6+ REG4+	ST3GAL4	3.09E-204	0.668461944	0.552	0.189	6.56E-200
SPINK4+ CLCA1+ DEFA6+ REG4+	AQP3	0	0.666119236	0.429	0.056	0
SPINK4+ CLCA1+ DEFA6+ REG4+	NPDC1	1.05E-129	0.661049169	0.892	0.638	2.24E-125
SPINK4+ CLCA1+ DEFA6+ REG4+	RAB2A	1.60E-136	0.661019084	0.894	0.813	3.40E-132
SPINK4+ CLCA1+ DEFA6+ REG4+	ODC1	8.60E-39	0.655992616	0.668	0.591	1.83E-34
SPINK4+ CLCA1+ DEFA6+ REG4+	COLCA1	9.99E-236	0.648729493	0.408	0.091	2.13E-231
SPINK4+ CLCA1+ DEFA6+ REG4+	PRUNE2	7.30E-187	0.646836657	0.575	0.221	1.55E-182
SPINK4+ CLCA1+ DEFA6+ REG4+	KCNMA1	0	0.642719917	0.401	0.02	0
SPINK4+ CLCA1+ DEFA6+ REG4+	PLEKHB1	1.56E-241	0.639839615	0.467	0.119	3.32E-237
SPINK4+ CLCA1+ DEFA6+ REG4+	SMIM14	2.21E-133	0.633606034	0.806	0.589	4.69E-129
SPINK4+ CLCA1+ DEFA6+ REG4+	ALDH1A1	2.19E-132	0.629710757	0.598	0.288	4.66E-128
SPINK4+ CLCA1+ DEFA6+ REG4+	ARFGEF3	1.17E-137	0.627741265	0.617	0.339	2.49E-133

SPINK4+ CLCA1+ DEFA6+ REG4+	MB	3.28E-307	0.616575674	0.446	0.085	6.98E-303
SPINK4+ CLCA1+ DEFA6+ REG4+	BCAS1	3.82E-87	0.610372686	0.567	0.325	8.12E-83
SPINK4+ CLCA1+ DEFA6+ REG4+	ITM2A	0	0.610325514	0.301	0.019	0
SPINK4+ CLCA1+ DEFA6+ REG4+	ERLEC1	1.86E-82	0.607987037	0.664	0.526	3.96E-78
SPINK4+ CLCA1+ DEFA6+ REG4+	MT-ND2	9.78E-87	0.606169752	0.998	0.989	2.08E-82
SPINK4+ CLCA1+ DEFA6+ REG4+	TGFBI	9.52E-85	0.602584868	0.708	0.443	2.02E-80
SPINK4+ CLCA1+ DEFA6+ REG4+	WIPI1	1.10E-133	0.596937301	0.594	0.319	2.34E-129
SPINK4+ CLCA1+ DEFA6+ REG4+	SELENOK	7.05E-85	0.59491091	0.805	0.739	1.50E-80
SPINK4+ CLCA1+ DEFA6+ REG4+	TCEA3	3.70E-84	0.594416162	0.755	0.557	7.86E-80
SPINK4+ CLCA1+ DEFA6+ REG4+	LMAN1	2.94E-68	0.594279767	0.658	0.535	6.25E-64
SPINK4+ CLCA1+ DEFA6+ REG4+	COL16A1	4.80E-214	0.586815411	0.369	0.081	1.02E-209
SPINK4+ CLCA1+ DEFA6+ REG4+	PPIB	1.74E-69	0.585019217	0.906	0.837	3.70E-65
SPINK4+ CLCA1+ DEFA6+ REG4+	MUC4	2.84E-53	0.584731121	0.688	0.541	6.05E-49
SPINK4+ CLCA1+ DEFA6+ REG4+	B4GALNT2	0	0.580238438	0.358	0.028	0
SPINK4+ CLCA1+ DEFA6+ REG4+	NPW	1.74E-156	0.580018525	0.311	0.073	3.69E-152
SPINK4+ CLCA1+ DEFA6+ REG4+	DLL1	0	0.576883712	0.358	0.037	0
SPINK4+ CLCA1+ DEFA6+ REG4+	PDIA4	1.79E-56	0.572956415	0.764	0.704	3.80E-52
SPINK4+ CLCA1+ DEFA6+ REG4+	DNAJC10	5.84E-89	0.572132866	0.576	0.369	1.24E-84
SPINK4+ CLCA1+ DEFA6+ REG4+	FMOD	0	0.567376687	0.328	0.036	0
SPINK4+ CLCA1+ DEFA6+ REG4+	FKBP2	9.43E-101	0.564570611	0.899	0.817	2.01E-96
SPINK4+ CLCA1+ DEFA6+ REG4+	BEST2	0	0.55130178	0.335	0.024	0
SPINK4+ CLCA1+ DEFA6+ REG4+	NECTIN1	4.28E-128	0.547000522	0.485	0.214	9.10E-124

SPINK4+ CLCA1+ DEFA6+ REG4+	SOD3	1.83E-56	0.546384775	0.592	0.418	3.89E-52
SPINK4+ CLCA1+ DEFA6+ REG4+	LYZ	9.96E-103	0.546262449	0.868	0.575	2.12E-98
SPINK4+ CLCA1+ DEFA6+ REG4+	NEURL1	0	0.544798483	0.454	0.073	0
SPINK4+ CLCA1+ DEFA6+ REG4+	CKAP4	9.24E-80	0.536091946	0.658	0.481	1.97E-75
SPINK4+ CLCA1+ DEFA6+ REG4+	SCG5	4.02E-154	0.531862451	0.376	0.11	8.55E-150
SPINK4+ CLCA1+ DEFA6+ REG4+	RABAC1	3.19E-70	0.527879192	0.832	0.755	6.77E-66
SPINK4+ CLCA1+ DEFA6+ REG4+	SEC61B	3.81E-74	0.522979996	0.909	0.851	8.11E-70
SPINK4+ CLCA1+ DEFA6+ REG4+	HSPA2	1.15E-196	0.519806603	0.372	0.086	2.44E-192
SPINK4+ CLCA1+ DEFA6+ REG4+	ODF2L	3.55E-60	0.515522428	0.551	0.382	7.54E-56
SPINK4+ CLCA1+ DEFA6+ REG4+	STARD10	1.29E-98	0.514370737	0.922	0.805	2.75E-94
SPINK4+ CLCA1+ DEFA6+ REG4+	RPL36	3.83E-126	0.513072042	0.999	0.974	8.14E-122
SPINK4+ CLCA1+ DEFA6+ REG4+	AC005833.1	1.96E-147	0.510772934	0.39	0.129	4.16E-143
SPINK4+ CLCA1+ DEFA6+ REG4+	ERGIC1	1.47E-77	0.5062397	0.666	0.503	3.12E-73
SPINK4+ CLCA1+ DEFA6+ REG4+	SOX9	1.12E-45	0.505274756	0.679	0.571	2.39E-41
SPINK4+ CLCA1+ DEFA6+ REG4+	LCN2	0.000101474	0.504483937	0.619	0.713	1
SPINK4+ CLCA1+ DEFA6+ REG4+	KLF4	9.01E-58	0.504260661	0.749	0.613	1.92E-53
SPINK4+ CLCA1+ DEFA6+ REG4+	FFAR4	3.34E-159	0.504102384	0.426	0.131	7.09E-155
SPINK4+ CLCA1+ DEFA6+ REG4+	FOXA2	7.63E-69	0.501426995	0.496	0.302	1.62E-64
SPINK4+ CLCA1+ DEFA6+ REG4+	MYDGF	5.30E-44	0.501402137	0.79	0.746	1.13E-39
SPINK4+ CLCA1+ DEFA6+ REG4+	TMED3	4.07E-52	0.500465714	0.707	0.641	8.66E-48
SPINK4+ CLCA1+ DEFA6+ REG4+	FRYL	1.06E-57	0.500163287	0.676	0.554	2.26E-53
SPINK4+ CLCA1+ DEFA6+ REG4+	SLC12A2	4.82E-81	0.498739972	0.951	0.779	1.03E-76

SPINK4+ CLCA1+ DEFA6+ REG4+	HDLBP	5.55E-64	0.498297402	0.771	0.694	1.18E-59
SPINK4+ CLCA1+ DEFA6+ REG4+	APIP	9.14E-46	0.498129921	0.645	0.522	1.94E-41
SPINK4+ CLCA1+ DEFA6+ REG4+	CAPN12	1.14E-83	0.492825714	0.472	0.258	2.43E-79
SPINK4+ CLCA1+ DEFA6+ REG4+	TMEM61	0	0.491281307	0.39	0.034	0
SPINK4+ CLCA1+ DEFA6+ REG4+	HSP90B1	1.82E-57	0.48644381	0.926	0.859	3.87E-53
SPINK4+ CLCA1+ DEFA6+ REG4+	KDELR1	1.66E-72	0.482762975	0.821	0.757	3.54E-68
SPINK4+ CLCA1+ DEFA6+ REG4+	TMED10	3.00E-51	0.480712863	0.774	0.749	6.37E-47
SPINK4+ CLCA1+ DEFA6+ REG4+	IL13RA1	2.24E-63	0.480681894	0.563	0.401	4.77E-59
SPINK4+ CLCA1+ DEFA6+ REG4+	SLC9A3R2	2.51E-63	0.477347469	0.606	0.436	5.34E-59
SPINK4+ CLCA1+ DEFA6+ REG4+	SSR3	3.24E-55	0.476864345	0.773	0.711	6.89E-51
SPINK4+ CLCA1+ DEFA6+ REG4+	SDF2L1	2.65E-28	0.473832619	0.651	0.593	5.64E-24
SPINK4+ CLCA1+ DEFA6+ REG4+	CRACR2B	1.22E-62	0.472992568	0.6	0.438	2.60E-58
SPINK4+ CLCA1+ DEFA6+ REG4+	ANG	8.30E-59	0.472776552	0.609	0.463	1.76E-54
SPINK4+ CLCA1+ DEFA6+ REG4+	ASPH	5.92E-44	0.47199852	0.701	0.605	1.26E-39
SPINK4+ CLCA1+ DEFA6+ REG4+	HPN	0	0.470943329	0.27	0.004	0
SPINK4+ CLCA1+ DEFA6+ REG4+	SERF2	6.29E-104	0.470640733	0.997	0.984	1.34E-99
SPINK4+ CLCA1+ DEFA6+ REG4+	OSTC	9.80E-60	0.464735712	0.799	0.722	2.08E-55
SPINK4+ CLCA1+ DEFA6+ REG4+	FOXA3	6.70E-56	0.456074547	0.571	0.416	1.42E-51
SPINK4+ CLCA1+ DEFA6+ REG4+	MANF	8.60E-40	0.455519424	0.681	0.596	1.83E-35
SPINK4+ CLCA1+ DEFA6+ REG4+	HERPUD1	1.96E-39	0.454275426	0.718	0.644	4.18E-35
SPINK4+ CLCA1+ DEFA6+ REG4+	MUC5B	2.43E-41	0.448930999	0.488	0.343	5.16E-37
SPINK4+ CLCA1+ DEFA6+ REG4+	SCGB2A1	6.05E-116	0.447957574	0.333	0.106	1.29E-111

SPINK4+ CLCA1+ DEFA6+ REG4+	RPL22L1	1.13E-38	0.444377031	0.708	0.628	2.41E-34
SPINK4+ CLCA1+ DEFA6+ REG4+	GALNT7	3.15E-58	0.442592071	0.624	0.478	6.70E-54
SPINK4+ CLCA1+ DEFA6+ REG4+	ITPR2	8.71E-52	0.442360954	0.457	0.266	1.85E-47
SPINK4+ CLCA1+ DEFA6+ REG4+	TMEM258	1.24E-76	0.441840694	0.948	0.874	2.63E-72
SPINK4+ CLCA1+ DEFA6+ REG4+	MLPH	4.73E-83	0.437774665	0.57	0.345	1.01E-78
SPINK4+ CLCA1+ DEFA6+ REG4+	MT-CYB	1.64E-67	0.429014139	0.997	0.986	3.49E-63
SPINK4+ CLCA1+ DEFA6+ REG4+	FAM174B	2.46E-234	0.428664529	0.353	0.065	5.24E-230
SPINK4+ CLCA1+ DEFA6+ REG4+	CACNA2D2	0	0.427299139	0.287	0.011	0
SPINK4+ CLCA1+ DEFA6+ REG4+	AP002498.1	0	0.427202146	0.299	0.012	0
SPINK4+ CLCA1+ DEFA6+ REG4+	NR2F2	1.30E-36	0.426702246	0.444	0.322	2.77E-32
SPINK4+ CLCA1+ DEFA6+ REG4+	SEC62	5.49E-58	0.424644899	0.83	0.784	1.17E-53
SPINK4+ CLCA1+ DEFA6+ REG4+	SLC39A8	2.36E-66	0.423600885	0.573	0.383	5.03E-62
SPINK4+ CLCA1+ DEFA6+ REG4+	SEC61G	4.83E-59	0.420619631	0.933	0.88	1.03E-54
SPINK4+ CLCA1+ DEFA6+ REG4+	SOX4	8.04E-08	0.417401863	0.661	0.752	0.001709126
SPINK4+ CLCA1+ DEFA6+ REG4+	EHF	1.99E-40	0.41496407	0.674	0.572	4.23E-36
SPINK4+ CLCA1+ DEFA6+ REG4+	GPRIN3	5.22E-57	0.410369746	0.402	0.233	1.11E-52
SPINK4+ CLCA1+ DEFA6+ REG4+	ERN2	1.96E-53	0.409374807	0.544	0.374	4.16E-49
SPINK4+ CLCA1+ DEFA6+ REG4+	KCNQ1OT1	9.62E-26	0.4079899	0.417	0.303	2.05E-21
SPINK4+ CLCA1+ DEFA6+ REG4+	GSE1	6.54E-57	0.407675552	0.497	0.327	1.39E-52
SPINK4+ CLCA1+ DEFA6+ REG4+	MIA3	1.27E-44	0.407029195	0.473	0.326	2.70E-40
SPINK4+ CLCA1+ DEFA6+ REG4+	SMAD9	5.73E-97	0.406448196	0.434	0.187	1.22E-92
SPINK4+ CLCA1+ DEFA6+ REG4+	CDC25B	3.21E-108	0.403183522	0.364	0.133	6.83E-104

SPINK4+ CLCA1+ DEFA6+ REG4+	CREB3L4	1.76E-112	0.401179555	0.372	0.14	3.73E-108
SPINK4+ CLCA1+ DEFA6+ REG4+	CMIP	2.40E-32	0.396515419	0.451	0.343	5.09E-28
SPINK4+ CLCA1+ DEFA6+ REG4+	CRELD2	2.11E-27	0.388461592	0.502	0.413	4.49E-23
SPINK4+ CLCA1+ DEFA6+ REG4+	GLRX	1.11E-32	0.387150509	0.614	0.543	2.36E-28
SPINK4+ CLCA1+ DEFA6+ REG4+	HSD11B2	1.11E-41	0.386590732	0.584	0.447	2.35E-37
SPINK4+ CLCA1+ DEFA6+ REG4+	ANO7	0	0.38492288	0.29	0.026	0
SPINK4+ CLCA1+ DEFA6+ REG4+	IFI27L2	9.17E-45	0.38347785	0.652	0.532	1.95E-40
SPINK4+ CLCA1+ DEFA6+ REG4+	KDELR3	3.58E-67	0.383167728	0.395	0.207	7.62E-63
SPINK4+ CLCA1+ DEFA6+ REG4+	TCEAL8	6.37E-48	0.382351469	0.551	0.402	1.36E-43
SPINK4+ CLCA1+ DEFA6+ REG4+	HID1	1.06E-92	0.3816319	0.378	0.156	2.25E-88
SPINK4+ CLCA1+ DEFA6+ REG4+	RPS15	8.07E-71	0.38149654	1	0.975	1.72E-66
SPINK4+ CLCA1+ DEFA6+ REG4+	REXO2	3.13E-38	0.380157975	0.737	0.64	6.66E-34
SPINK4+ CLCA1+ DEFA6+ REG4+	TM9SF3	5.40E-50	0.37860768	0.863	0.802	1.15E-45
SPINK4+ CLCA1+ DEFA6+ REG4+	SPCS3	3.00E-47	0.376917921	0.638	0.53	6.39E-43
SPINK4+ CLCA1+ DEFA6+ REG4+	GADD45G	5.80E-55	0.375995775	0.318	0.154	1.23E-50
SPINK4+ CLCA1+ DEFA6+ REG4+	RAB15	1.23E-85	0.374024004	0.364	0.161	2.61E-81
SPINK4+ CLCA1+ DEFA6+ REG4+	PDXDC1	1.84E-46	0.37007357	0.688	0.579	3.91E-42
SPINK4+ CLCA1+ DEFA6+ REG4+	NEDD4L	1.14E-35	0.368533527	0.511	0.388	2.42E-31
SPINK4+ CLCA1+ DEFA6+ REG4+	TMED2	1.20E-46	0.367095663	0.847	0.81	2.54E-42
SPINK4+ CLCA1+ DEFA6+ REG4+	TRIM8	1.12E-44	0.366042284	0.484	0.336	2.39E-40
SPINK4+ CLCA1+ DEFA6+ REG4+	GALNT3	4.75E-39	0.365710776	0.621	0.524	1.01E-34
SPINK4+ CLCA1+ DEFA6+ REG4+	RBP4	3.44E-68	0.363090919	0.396	0.181	7.31E-64

SPINK4+ CLCA1+ DEFA6+ REG4+	GLUL	6.69E-48	0.362843657	0.79	0.685	1.42E-43
SPINK4+ CLCA1+ DEFA6+ REG4+	MT-ND3	1.43E-47	0.359579925	0.996	0.985	3.04E-43
SPINK4+ CLCA1+ DEFA6+ REG4+	SEC24D	1.11E-86	0.35945053	0.35	0.145	2.36E-82
SPINK4+ CLCA1+ DEFA6+ REG4+	MCTP2	3.71E-62	0.357837523	0.409	0.225	7.88E-58
SPINK4+ CLCA1+ DEFA6+ REG4+	RANBP2	3.46E-39	0.35759595	0.528	0.404	7.36E-35
SPINK4+ CLCA1+ DEFA6+ REG4+	CCDC174	6.59E-45	0.357575472	0.455	0.304	1.40E-40
SPINK4+ CLCA1+ DEFA6+ REG4+	TNFRSF11B	5.62E-113	0.356910232	0.259	0.068	1.20E-108
SPINK4+ CLCA1+ DEFA6+ REG4+	RPS11	1.69E-66	0.354565989	0.997	0.945	3.60E-62
SPINK4+ CLCA1+ DEFA6+ REG4+	CCDC14	2.14E-25	0.354236695	0.599	0.528	4.56E-21
SPINK4+ CLCA1+ DEFA6+ REG4+	SMIM31	9.56E-72	0.353975348	0.612	0.384	2.03E-67
SPINK4+ CLCA1+ DEFA6+ REG4+	CD74	1.05E-58	0.353150188	0.787	0.591	2.24E-54
SPINK4+ CLCA1+ DEFA6+ REG4+	RPS12	8.12E-75	0.351542078	0.999	0.969	1.73E-70
SPINK4+ CLCA1+ DEFA6+ REG4+	PLPP5	1.81E-38	0.35127293	0.541	0.421	3.85E-34
SPINK4+ CLCA1+ DEFA6+ REG4+	SERP1	2.02E-46	0.349034603	0.888	0.855	4.29E-42
SPINK4+ CLCA1+ DEFA6+ REG4+	FRA10AC1	2.57E-46	0.343411607	0.454	0.297	5.47E-42
SPINK4+ CLCA1+ DEFA6+ REG4+	CADPS	7.20E-94	0.342728045	0.361	0.141	1.53E-89
SPINK4+ CLCA1+ DEFA6+ REG4+	PPIC	5.23E-22	0.341849175	0.682	0.658	1.11E-17
SPINK4+ CLCA1+ DEFA6+ REG4+	SYNGR2	8.58E-36	0.34049718	0.8	0.726	1.83E-31
SPINK4+ CLCA1+ DEFA6+ REG4+	TC2N	8.70E-21	0.34016907	0.483	0.414	1.85E-16
SPINK4+ CLCA1+ DEFA6+ REG4+	PRDX4	3.01E-26	0.339533389	0.711	0.676	6.40E-22
SPINK4+ CLCA1+ DEFA6+ REG4+	KDELR2	5.23E-38	0.339490233	0.869	0.836	1.11E-33
SPINK4+ CLCA1+ DEFA6+ REG4+	SLC36A4	4.46E-129	0.339200841	0.305	0.084	9.49E-125

SPINK4+ CLCA1+ DEFA6+ REG4+	GCC2	8.44E-32	0.337778493	0.797	0.739	1.79E-27
SPINK4+ CLCA1+ DEFA6+ REG4+	FOXP1	6.85E-38	0.336645819	0.764	0.683	1.46E-33
SPINK4+ CLCA1+ DEFA6+ REG4+	HES2	1.08E-88	0.336105993	0.34	0.133	2.30E-84
SPINK4+ CLCA1+ DEFA6+ REG4+	SEC61A1	1.33E-30	0.335594727	0.563	0.478	2.82E-26
SPINK4+ CLCA1+ DEFA6+ REG4+	RAB27A	9.62E-67	0.335386642	0.381	0.185	2.05E-62
SPINK4+ CLCA1+ DEFA6+ REG4+	MBOAT2	3.68E-48	0.335327475	0.42	0.255	7.82E-44
SPINK4+ CLCA1+ DEFA6+ REG4+	POLR2L	4.61E-39	0.334575881	0.94	0.869	9.80E-35
SPINK4+ CLCA1+ DEFA6+ REG4+	OST4	1.82E-54	0.333431696	0.956	0.903	3.87E-50
SPINK4+ CLCA1+ DEFA6+ REG4+	RPL12	3.75E-50	0.330586453	0.997	0.969	7.97E-46
SPINK4+ CLCA1+ DEFA6+ REG4+	LXN	2.87E-38	0.330012946	0.442	0.308	6.11E-34
SPINK4+ CLCA1+ DEFA6+ REG4+	SSR2	7.54E-32	0.329849317	0.863	0.812	1.60E-27
SPINK4+ CLCA1+ DEFA6+ REG4+	OCIAD2	3.61E-43	0.328698035	0.881	0.813	7.68E-39
SPINK4+ CLCA1+ DEFA6+ REG4+	SHF	6.17E-162	0.328328833	0.262	0.051	1.31E-157
SPINK4+ CLCA1+ DEFA6+ REG4+	SLC4A7	1.60E-53	0.327725667	0.342	0.179	3.40E-49
SPINK4+ CLCA1+ DEFA6+ REG4+	AKAP9	1.29E-28	0.324686587	0.838	0.787	2.74E-24
SPINK4+ CLCA1+ DEFA6+ REG4+	ATF3	1.25E-05	0.324318275	0.549	0.525	0.266074102
SPINK4+ CLCA1+ DEFA6+ REG4+	URAD	2.05E-42	0.323610556	0.382	0.218	4.36E-38
SPINK4+ CLCA1+ DEFA6+ REG4+	AP3S1	1.11E-33	0.321854058	0.717	0.647	2.36E-29
SPINK4+ CLCA1+ DEFA6+ REG4+	SCNN1A	1.28E-74	0.318183492	0.551	0.32	2.71E-70
SPINK4+ CLCA1+ DEFA6+ REG4+	RPL36A	3.86E-40	0.317583905	0.986	0.904	8.22E-36
SPINK4+ CLCA1+ DEFA6+ REG4+	RPL34	7.82E-58	0.315903872	1	0.979	1.66E-53
SPINK4+ CLCA1+ DEFA6+ REG4+	SYTL1	7.20E-77	0.315264734	0.312	0.128	1.53E-72

SPINK4+ CLCA1+ DEFA6+ REG4+	CBFA2T2	5.80E-36	0.315211896	0.366	0.23	1.23E-31
SPINK4+ CLCA1+ DEFA6+ REG4+	SEL1L3	3.40E-31	0.314858459	0.486	0.383	7.23E-27
SPINK4+ CLCA1+ DEFA6+ REG4+	ABLIM1	3.71E-30	0.314801997	0.574	0.485	7.89E-26
SPINK4+ CLCA1+ DEFA6+ REG4+	CALR	1.71E-19	0.308753437	0.871	0.869	3.64E-15
SPINK4+ CLCA1+ DEFA6+ REG4+	PTPRN2	8.75E-90	0.308609863	0.264	0.084	1.86E-85
SPINK4+ CLCA1+ DEFA6+ REG4+	NTN4	1.55E-95	0.308247024	0.279	0.087	3.29E-91
SPINK4+ CLCA1+ DEFA6+ REG4+	S100A13	6.79E-33	0.307500376	0.689	0.605	1.44E-28
SPINK4+ CLCA1+ DEFA6+ REG4+	KCNE3	4.39E-22	0.307453752	0.408	0.321	9.33E-18
SPINK4+ CLCA1+ DEFA6+ REG4+	C4BPB	3.55E-63	0.307226813	0.282	0.118	7.55E-59
SPINK4+ CLCA1+ DEFA6+ REG4+	QSOX1	2.23E-57	0.306867858	0.673	0.484	4.75E-53
SPINK4+ CLCA1+ DEFA6+ REG4+	FOSB	1.26E-13	0.304770393	0.752	0.692	2.68E-09
SPINK4+ CLCA1+ DEFA6+ REG4+	SLC35A1	2.61E-39	0.304568838	0.462	0.322	5.55E-35
SPINK4+ CLCA1+ DEFA6+ REG4+	TRPM4	1.72E-12	0.304198364	0.57	0.533	3.66E-08
SPINK4+ CLCA1+ DEFA6+ REG4+	CDC42EP5	5.62E-21	0.302551664	0.784	0.753	1.19E-16
SPINK4+ CLCA1+ DEFA6+ REG4+	MSI2	2.32E-25	0.301358664	0.542	0.462	4.94E-21
SPINK4+ CLCA1+ DEFA6+ REG4+	ZNF511	2.16E-26	0.300827656	0.65	0.575	4.59E-22
SPINK4+ CLCA1+ DEFA6+ REG4+	B4GALT4	3.86E-38	0.300771521	0.43	0.289	8.22E-34
SPINK4+ CLCA1+ DEFA6+ REG4+	ITGA6	4.43E-24	0.297515506	0.766	0.701	9.41E-20
SPINK4+ CLCA1+ DEFA6+ REG4+	GALNT5	5.77E-43	0.297437972	0.376	0.222	1.23E-38
SPINK4+ CLCA1+ DEFA6+ REG4+	MAN1A1	2.65E-60	0.297389836	0.36	0.177	5.64E-56
SPINK4+ CLCA1+ DEFA6+ REG4+	ETS1	6.55E-96	0.296642634	0.279	0.087	1.39E-91
SPINK4+ CLCA1+ DEFA6+ REG4+	MAPKAPK5-AS1	8.63E-20	0.296266545	0.442	0.361	1.83E-15

SPINK4+ CLCA1+ DEFA6+ REG4+	BACE2	9.16E-20	0.295792785	0.707	0.67	1.95E-15
SPINK4+ CLCA1+ DEFA6+ REG4+	BTG2	9.78E-14	0.2957632	0.745	0.663	2.08E-09
SPINK4+ CLCA1+ DEFA6+ REG4+	CRACR2A	6.43E-78	0.292868303	0.289	0.108	1.37E-73
SPINK4+ CLCA1+ DEFA6+ REG4+	GFPT1	3.58E-24	0.292862004	0.723	0.663	7.61E-20
SPINK4+ CLCA1+ DEFA6+ REG4+	MGLL	7.94E-38	0.292591833	0.694	0.592	1.69E-33
SPINK4+ CLCA1+ DEFA6+ REG4+	RPL38	3.62E-47	0.291727746	0.995	0.959	7.70E-43
SPINK4+ CLCA1+ DEFA6+ REG4+	PDIA5	1.71E-47	0.291365461	0.352	0.2	3.63E-43
SPINK4+ CLCA1+ DEFA6+ REG4+	ARL1	6.53E-22	0.290189549	0.61	0.563	1.39E-17
SPINK4+ CLCA1+ DEFA6+ REG4+	HLA-A	6.49E-58	0.289854177	0.987	0.978	1.38E-53
SPINK4+ CLCA1+ DEFA6+ REG4+	IER3	1.05E-18	0.289311108	0.765	0.695	2.24E-14
SPINK4+ CLCA1+ DEFA6+ REG4+	FAM213A	2.59E-18	0.289300076	0.572	0.508	5.51E-14
SPINK4+ CLCA1+ DEFA6+ REG4+	DPM3	5.26E-20	0.287677654	0.651	0.624	1.12E-15
SPINK4+ CLCA1+ DEFA6+ REG4+	UCP2	4.73E-28	0.287070743	0.403	0.281	1.01E-23
SPINK4+ CLCA1+ DEFA6+ REG4+	INSR	1.17E-23	0.286286961	0.538	0.438	2.49E-19
SPINK4+ CLCA1+ DEFA6+ REG4+	ARF4	1.25E-24	0.28553307	0.771	0.738	2.67E-20
SPINK4+ CLCA1+ DEFA6+ REG4+	CHD9	1.40E-14	0.283929756	0.553	0.519	2.99E-10
SPINK4+ CLCA1+ DEFA6+ REG4+	ID2	3.17E-14	0.281285776	0.741	0.708	6.74E-10
SPINK4+ CLCA1+ DEFA6+ REG4+	HM13	1.17E-19	0.279093274	0.59	0.532	2.48E-15
SPINK4+ CLCA1+ DEFA6+ REG4+	ATP2A3	2.17E-31	0.277716939	0.412	0.278	4.62E-27
SPINK4+ CLCA1+ DEFA6+ REG4+	SIL1	6.94E-21	0.275748222	0.451	0.371	1.48E-16
SPINK4+ CLCA1+ DEFA6+ REG4+	EPHB3	9.22E-28	0.275725379	0.476	0.338	1.96E-23
SPINK4+ CLCA1+ DEFA6+ REG4+	RPL13	2.15E-38	0.275418131	0.999	0.964	4.58E-34

SPINK4+ CLCA1+ DEFA6+ REG4+	RPL31	2.17E-38	0.274791745	0.995	0.956	4.62E-34
SPINK4+ CLCA1+ DEFA6+ REG4+	MT-ATP6	4.86E-37	0.274392664	0.996	0.986	1.03E-32
SPINK4+ CLCA1+ DEFA6+ REG4+	ARFGAP3	1.86E-42	0.274272145	0.422	0.267	3.96E-38
SPINK4+ CLCA1+ DEFA6+ REG4+	HLA-E	7.98E-63	0.273440208	0.911	0.863	1.70E-58
SPINK4+ CLCA1+ DEFA6+ REG4+	PRIMPOL	7.65E-67	0.27200638	0.26	0.1	1.63E-62
SPINK4+ CLCA1+ DEFA6+ REG4+	CANT1	1.88E-20	0.271781348	0.582	0.531	4.01E-16
SPINK4+ CLCA1+ DEFA6+ REG4+	TMED9	2.34E-20	0.27151322	0.796	0.77	4.97E-16
SPINK4+ CLCA1+ DEFA6+ REG4+	TOMM7	2.91E-14	0.271478306	0.938	0.869	6.19E-10
SPINK4+ CLCA1+ DEFA6+ REG4+	RPL18	1.91E-25	0.271096691	0.997	0.964	4.07E-21
SPINK4+ CLCA1+ DEFA6+ REG4+	PROX1	7.84E-14	0.27037577	0.318	0.237	1.67E-09
SPINK4+ CLCA1+ DEFA6+ REG4+	TMEM263	3.07E-42	0.268870732	0.361	0.213	6.53E-38
SPINK4+ CLCA1+ DEFA6+ REG4+	TENT5C	6.51E-49	0.26832921	0.252	0.111	1.38E-44
SPINK4+ CLCA1+ DEFA6+ REG4+	RPL37	4.07E-26	0.268264945	0.993	0.945	8.66E-22
SPINK4+ CLCA1+ DEFA6+ REG4+	STXBP1	5.99E-96	0.266646052	0.26	0.077	1.27E-91
SPINK4+ CLCA1+ DEFA6+ REG4+	PLXDC2	3.45E-76	0.266610745	0.264	0.093	7.34E-72
SPINK4+ CLCA1+ DEFA6+ REG4+	SNHG18	2.69E-14	0.266566164	0.519	0.474	5.72E-10
SPINK4+ CLCA1+ DEFA6+ REG4+	RPL37A	4.10E-42	0.262555448	0.996	0.964	8.73E-38
SPINK4+ CLCA1+ DEFA6+ REG4+	HMG20B	8.89E-18	0.262409466	0.489	0.428	1.89E-13
SPINK4+ CLCA1+ DEFA6+ REG4+	FAM114A1	2.15E-29	0.260699701	0.426	0.304	4.58E-25
SPINK4+ CLCA1+ DEFA6+ REG4+	MCF2L	1.21E-67	0.258354681	0.287	0.115	2.58E-63
SPINK4+ CLCA1+ DEFA6+ REG4+	TBC1D2	1.36E-69	0.256963276	0.252	0.092	2.90E-65
SPINK4+ CLCA1+ DEFA6+ REG4+	IFITM2	6.40E-22	0.256316598	0.539	0.428	1.36E-17

SPINK4+ CLCA1+ DEFA6+ REG4+	KRT18	4.75E-22	0.253753447	0.994	0.981	1.01E-17
SPINK4+ CLCA1+ DEFA6+ REG4+	DNTTIP1	9.96E-20	0.252447094	0.464	0.39	2.12E-15
SPINK4+ CLCA1+ DEFA6+ REG4+	ENTPD8	2.34E-150	0.251982767	0.489	0.157	4.98E-146
SPINK4+ CLCA1+ DEFA6+ REG4+	ICA1	3.06E-15	0.251556245	0.532	0.469	6.51E-11
SPINK4+ CLCA1+ DEFA6+ REG4+	WNK2	3.16E-21	0.251274714	0.437	0.345	6.71E-17
SPINK4+ CLCA1+ DEFA6+ REG4+	SLC39A7	8.80E-16	0.250816043	0.5	0.448	1.87E-11
SPINK4+ CLCA1+ DEFA6+ REG4+	KCNK6	2.30E-28	0.250186761	0.428	0.305	4.89E-24
TUBA1B+ H2AFZ+ HMGB2+ HIST1H4C+	TUBA1B	0	2.13081014	0.991	0.742	0
TUBA1B+ H2AFZ+ HMGB2+ HIST1H4C+	H2AFZ	0	2.04166508	0.996	0.774	0
TUBA1B+ H2AFZ+ HMGB2+ HIST1H4C+	HMGB2	0	2.020781888	0.907	0.445	0
TUBA1B+ H2AFZ+ HMGB2+ HIST1H4C+	HIST1H4C	7.25E-288	2.012540123	0.895	0.659	1.54E-283
TUBA1B+ H2AFZ+ HMGB2+ HIST1H4C+	PTTG1	0	1.73256918	0.727	0.108	0
TUBA1B+ H2AFZ+ HMGB2+ HIST1H4C+	STMN1	0	1.692862253	0.938	0.343	0
TUBA1B+ H2AFZ+ HMGB2+ HIST1H4C+	HMGN2	0	1.561641182	0.986	0.806	0
TUBA1B+ H2AFZ+ HMGB2+ HIST1H4C+	PCLAF	0	1.546665662	0.77	0.098	0
TUBA1B+ H2AFZ+ HMGB2+ HIST1H4C+	UBE2C	0	1.481562858	0.541	0.025	0
TUBA1B+ H2AFZ+ HMGB2+ HIST1H4C+	TUBB	0	1.455595137	0.949	0.62	0
TUBA1B+ H2AFZ+ HMGB2+ HIST1H4C+	CKS2	0	1.308546852	0.855	0.413	0
TUBA1B+ H2AFZ+ HMGB2+ HIST1H4C+	HSPD1	0	1.270686678	0.982	0.766	0
TUBA1B+ H2AFZ+ HMGB2+ HIST1H4C+	RANBP1	0	1.267583545	0.954	0.665	0
TUBA1B+ H2AFZ+ HMGB2+ HIST1H4C+	CENPW	0	1.263989452	0.824	0.191	0
TUBA1B+ H2AFZ+ HMGB2+ HIST1H4C+	MKI67	0	1.259137611	0.67	0.038	0

TUBA1B+ H2AFZ+ HMGB2+ HIST1H4C+	OLFM4	4.73E-264	1.252022056	0.873	0.581	1.00E-259
TUBA1B+ H2AFZ+ HMGB2+ HIST1H4C+	HMGB1	0	1.250784919	0.999	0.909	0
TUBA1B+ H2AFZ+ HMGB2+ HIST1H4C+	RAN	0	1.203619635	0.989	0.797	0
TUBA1B+ H2AFZ+ HMGB2+ HIST1H4C+	LDHB	0	1.192645624	0.722	0.346	0
TUBA1B+ H2AFZ+ HMGB2+ HIST1H4C+	CKS1B	0	1.174910181	0.847	0.363	0
TUBA1B+ H2AFZ+ HMGB2+ HIST1H4C+	CENPF	0	1.145975253	0.586	0.046	0
TUBA1B+ H2AFZ+ HMGB2+ HIST1H4C+	RRM2	0	1.142957788	0.595	0.026	0
TUBA1B+ H2AFZ+ HMGB2+ HIST1H4C+	ENO1	0	1.133658281	0.983	0.796	0
TUBA1B+ H2AFZ+ HMGB2+ HIST1H4C+	TUBB4B	0	1.130446331	0.964	0.753	0
TUBA1B+ H2AFZ+ HMGB2+ HIST1H4C+	TOP2A	0	1.093235655	0.525	0.021	0
TUBA1B+ H2AFZ+ HMGB2+ HIST1H4C+	CCNB1	0	1.076320614	0.529	0.052	0
TUBA1B+ H2AFZ+ HMGB2+ HIST1H4C+	IDH2	0	1.074384347	0.948	0.646	0
TUBA1B+ H2AFZ+ HMGB2+ HIST1H4C+	PCNA	0	1.050694284	0.754	0.3	0
TUBA1B+ H2AFZ+ HMGB2+ HIST1H4C+	HSPE1	0	1.047372938	0.993	0.843	0
TUBA1B+ H2AFZ+ HMGB2+ HIST1H4C+	TK1	0	1.015971951	0.701	0.071	0
TUBA1B+ H2AFZ+ HMGB2+ HIST1H4C+	BIRC5	0	1.013744333	0.615	0.038	0
TUBA1B+ H2AFZ+ HMGB2+ HIST1H4C+	PTMA	0	1.006879044	1	0.982	0
TUBA1B+ H2AFZ+ HMGB2+ HIST1H4C+	SMC4	0	0.998753386	0.779	0.252	0
TUBA1B+ H2AFZ+ HMGB2+ HIST1H4C+	FABP5	0	0.971951209	0.894	0.538	0
TUBA1B+ H2AFZ+ HMGB2+ HIST1H4C+	MAD2L1	0	0.962268563	0.679	0.072	0
TUBA1B+ H2AFZ+ HMGB2+ HIST1H4C+	TYMS	0	0.961608624	0.666	0.092	0
TUBA1B+ H2AFZ+ HMGB2+ HIST1H4C+	DUT	0	0.958693091	0.85	0.499	0

TUBA1B+ H2AFZ+ HMGB2+ HIST1H4C+	CDKN3	0	0.951498833	0.622	0.056	0
TUBA1B+ H2AFZ+ HMGB2+ HIST1H4C+	HSP90AA1	0	0.926388173	0.994	0.904	0
TUBA1B+ H2AFZ+ HMGB2+ HIST1H4C+	ARL6IP1	1.73E-210	0.904944364	0.931	0.741	3.67E-206
TUBA1B+ H2AFZ+ HMGB2+ HIST1H4C+	SNRPG	0	0.890090733	0.986	0.82	0
TUBA1B+ H2AFZ+ HMGB2+ HIST1H4C+	CDK1	0	0.885264644	0.454	0.014	0
TUBA1B+ H2AFZ+ HMGB2+ HIST1H4C+	DBI	0	0.883966496	0.989	0.849	0
TUBA1B+ H2AFZ+ HMGB2+ HIST1H4C+	PA2G4	0	0.883546002	0.954	0.7	0
TUBA1B+ H2AFZ+ HMGB2+ HIST1H4C+	DEK	0	0.876900336	0.965	0.738	0
TUBA1B+ H2AFZ+ HMGB2+ HIST1H4C+	SNRPD1	0	0.875685648	0.945	0.675	0
TUBA1B+ H2AFZ+ HMGB2+ HIST1H4C+	SNRPB	0	0.873495994	0.967	0.736	0
TUBA1B+ H2AFZ+ HMGB2+ HIST1H4C+	DTYMK	0	0.872899372	0.784	0.227	0
TUBA1B+ H2AFZ+ HMGB2+ HIST1H4C+	NCL	0	0.871650122	0.957	0.753	0
TUBA1B+ H2AFZ+ HMGB2+ HIST1H4C+	NASP	0	0.870481375	0.822	0.373	0
TUBA1B+ H2AFZ+ HMGB2+ HIST1H4C+	NUSAP1	0	0.867774109	0.511	0.015	0
TUBA1B+ H2AFZ+ HMGB2+ HIST1H4C+	DHFR	0	0.85804533	0.721	0.135	0
TUBA1B+ H2AFZ+ HMGB2+ HIST1H4C+	NPM1	0	0.856162138	0.995	0.85	0
TUBA1B+ H2AFZ+ HMGB2+ HIST1H4C+	GGCT	0	0.854597419	0.925	0.571	0
TUBA1B+ H2AFZ+ HMGB2+ HIST1H4C+	CDC20	0	0.850864623	0.5	0.027	0
TUBA1B+ H2AFZ+ HMGB2+ HIST1H4C+	PGAM1	0	0.848568262	0.93	0.642	0
TUBA1B+ H2AFZ+ HMGB2+ HIST1H4C+	ZWINT	0	0.842537848	0.676	0.105	0
TUBA1B+ H2AFZ+ HMGB2+ HIST1H4C+	HNRNPA2B1	0	0.818433706	0.989	0.889	0
TUBA1B+ H2AFZ+ HMGB2+ HIST1H4C+	KPNA2	0	0.816574029	0.586	0.136	0

TUBA1B+ H2AFZ+ HMGB2+ HIST1H4C+	LSM4	0	0.814900872	0.946	0.692	0
TUBA1B+ H2AFZ+ HMGB2+ HIST1H4C+	PSMA7	0	0.812497479	0.979	0.858	0
TUBA1B+ H2AFZ+ HMGB2+ HIST1H4C+	SNRPF	0	0.811716132	0.976	0.753	0
TUBA1B+ H2AFZ+ HMGB2+ HIST1H4C+	TPI1	0	0.811561824	0.991	0.892	0
TUBA1B+ H2AFZ+ HMGB2+ HIST1H4C+	UBE2T	0	0.811430575	0.677	0.107	0
TUBA1B+ H2AFZ+ HMGB2+ HIST1H4C+	NUCKS1	0	0.810506488	0.936	0.679	0
TUBA1B+ H2AFZ+ HMGB2+ HIST1H4C+	UBE2S	0	0.800457321	0.651	0.208	0
TUBA1B+ H2AFZ+ HMGB2+ HIST1H4C+	NME1	0	0.79170732	0.88	0.551	0
TUBA1B+ H2AFZ+ HMGB2+ HIST1H4C+	ANP32B	0	0.79158921	0.96	0.699	0
TUBA1B+ H2AFZ+ HMGB2+ HIST1H4C+	RPA3	0	0.787686039	0.875	0.439	0
TUBA1B+ H2AFZ+ HMGB2+ HIST1H4C+	GLO1	0	0.787520562	0.876	0.512	0
TUBA1B+ H2AFZ+ HMGB2+ HIST1H4C+	H2AFV	0	0.776302935	0.952	0.697	0
TUBA1B+ H2AFZ+ HMGB2+ HIST1H4C+	LSM3	0	0.77568749	0.962	0.71	0
TUBA1B+ H2AFZ+ HMGB2+ HIST1H4C+	SMC2	0	0.77499721	0.701	0.157	0
TUBA1B+ H2AFZ+ HMGB2+ HIST1H4C+	YBX1	0	0.768584985	0.997	0.894	0
TUBA1B+ H2AFZ+ HMGB2+ HIST1H4C+	PRDX2	0	0.767734217	0.955	0.767	0
TUBA1B+ H2AFZ+ HMGB2+ HIST1H4C+	HNRNPA3	0	0.76426895	0.956	0.761	0
TUBA1B+ H2AFZ+ HMGB2+ HIST1H4C+	TUBA1C	6.47E-236	0.763153726	0.879	0.598	1.38E-231
TUBA1B+ H2AFZ+ HMGB2+ HIST1H4C+	MIF	0	0.76230676	0.983	0.856	0
TUBA1B+ H2AFZ+ HMGB2+ HIST1H4C+	CENPX	1.86E-300	0.762141258	0.88	0.584	3.96E-296
TUBA1B+ H2AFZ+ HMGB2+ HIST1H4C+	FDPS	9.37E-276	0.759497319	0.89	0.587	1.99E-271
TUBA1B+ H2AFZ+ HMGB2+ HIST1H4C+	EBP	0	0.756367739	0.881	0.536	0

TUBA1B+ H2AFZ+ HMGB2+ HIST1H4C+	GSTP1	0	0.752449068	0.996	0.91	0
TUBA1B+ H2AFZ+ HMGB2+ HIST1H4C+	CYC1	0	0.751084639	0.967	0.774	0
TUBA1B+ H2AFZ+ HMGB2+ HIST1H4C+	WDR34	0	0.749436786	0.766	0.284	0
TUBA1B+ H2AFZ+ HMGB2+ HIST1H4C+	SOD1	0	0.748468466	0.991	0.865	0
TUBA1B+ H2AFZ+ HMGB2+ HIST1H4C+	CCNB2	0	0.745568533	0.483	0.046	0
TUBA1B+ H2AFZ+ HMGB2+ HIST1H4C+	PTGES3	0	0.744359808	0.977	0.796	0
TUBA1B+ H2AFZ+ HMGB2+ HIST1H4C+	CACYBP	0	0.742349484	0.899	0.532	0
TUBA1B+ H2AFZ+ HMGB2+ HIST1H4C+	LSM5	0	0.741387337	0.953	0.697	0
TUBA1B+ H2AFZ+ HMGB2+ HIST1H4C+	MCM7	0	0.737478199	0.718	0.238	0
TUBA1B+ H2AFZ+ HMGB2+ HIST1H4C+	CENPM	0	0.737301971	0.619	0.06	0
TUBA1B+ H2AFZ+ HMGB2+ HIST1H4C+	TPX2	0	0.730394399	0.509	0.024	0
TUBA1B+ H2AFZ+ HMGB2+ HIST1H4C+	HSP90AB1	6.48E-284	0.728205667	0.991	0.863	1.38E-279
TUBA1B+ H2AFZ+ HMGB2+ HIST1H4C+	EIF5A	5.34E-183	0.718148701	0.891	0.674	1.14E-178
TUBA1B+ H2AFZ+ HMGB2+ HIST1H4C+	HMGN1	0	0.715768986	0.989	0.846	0
TUBA1B+ H2AFZ+ HMGB2+ HIST1H4C+	LMNB1	0	0.71476712	0.696	0.143	0
TUBA1B+ H2AFZ+ HMGB2+ HIST1H4C+	PKM	1.18E-258	0.713788536	0.968	0.774	2.50E-254
TUBA1B+ H2AFZ+ HMGB2+ HIST1H4C+	MZT2A	0	0.713186385	0.956	0.722	0
TUBA1B+ H2AFZ+ HMGB2+ HIST1H4C+	CCT5	0	0.713134122	0.911	0.625	0
TUBA1B+ H2AFZ+ HMGB2+ HIST1H4C+	ACAT2	0	0.710573391	0.735	0.301	0
TUBA1B+ H2AFZ+ HMGB2+ HIST1H4C+	HMGB3	0	0.707393105	0.809	0.363	0
TUBA1B+ H2AFZ+ HMGB2+ HIST1H4C+	PPIA	0	0.698770746	0.996	0.892	0
TUBA1B+ H2AFZ+ HMGB2+ HIST1H4C+	SIVA1	0	0.696198433	0.903	0.605	0

TUBA1B+ H2AFZ+ HMGB2+ HIST1H4C+	CKLF	0	0.680930217	0.862	0.473	0
TUBA1B+ H2AFZ+ HMGB2+ HIST1H4C+	MLEC	7.02E-276	0.679785414	0.97	0.776	1.49E-271
TUBA1B+ H2AFZ+ HMGB2+ HIST1H4C+	MGST1	3.44E-224	0.676209578	0.933	0.715	7.31E-220
TUBA1B+ H2AFZ+ HMGB2+ HIST1H4C+	GGH	3.16E-301	0.67486194	0.856	0.504	6.73E-297
TUBA1B+ H2AFZ+ HMGB2+ HIST1H4C+	ATP5MC3	0	0.67395221	0.99	0.866	0
TUBA1B+ H2AFZ+ HMGB2+ HIST1H4C+	NDUFAB1	0	0.672354345	0.962	0.764	0
TUBA1B+ H2AFZ+ HMGB2+ HIST1H4C+	GMNN	0	0.667297756	0.767	0.322	0
TUBA1B+ H2AFZ+ HMGB2+ HIST1H4C+	PSME2	1.30E-267	0.665511749	0.949	0.778	2.76E-263
TUBA1B+ H2AFZ+ HMGB2+ HIST1H4C+	TOMM40	0	0.661301477	0.835	0.442	0
TUBA1B+ H2AFZ+ HMGB2+ HIST1H4C+	RAD21	3.03E-223	0.657278667	0.782	0.491	6.44E-219
TUBA1B+ H2AFZ+ HMGB2+ HIST1H4C+	DNMT1	0	0.65436034	0.683	0.19	0
TUBA1B+ H2AFZ+ HMGB2+ HIST1H4C+	VDAC1	3.05E-221	0.65319939	0.969	0.809	6.49E-217
TUBA1B+ H2AFZ+ HMGB2+ HIST1H4C+	SET	1.30E-254	0.652184705	0.97	0.782	2.76E-250
TUBA1B+ H2AFZ+ HMGB2+ HIST1H4C+	GINS2	0	0.650180397	0.573	0.069	0
TUBA1B+ H2AFZ+ HMGB2+ HIST1H4C+	PLP2	7.17E-280	0.649440207	0.949	0.702	1.52E-275
TUBA1B+ H2AFZ+ HMGB2+ HIST1H4C+	SERBP1	0	0.645849683	0.971	0.802	0
TUBA1B+ H2AFZ+ HMGB2+ HIST1H4C+	HNRNPAB	1.06E-286	0.64174652	0.943	0.726	2.25E-282
TUBA1B+ H2AFZ+ HMGB2+ HIST1H4C+	MZT2B	0	0.639887078	0.978	0.833	0
TUBA1B+ H2AFZ+ HMGB2+ HIST1H4C+	PGP	0	0.638699355	0.767	0.279	0
TUBA1B+ H2AFZ+ HMGB2+ HIST1H4C+	BUB3	1.59E-271	0.635728236	0.803	0.451	3.39E-267
TUBA1B+ H2AFZ+ HMGB2+ HIST1H4C+	SRSF3	2.14E-281	0.634573229	0.969	0.795	4.54E-277
TUBA1B+ H2AFZ+ HMGB2+ HIST1H4C+	ATP5IF1	2.09E-293	0.633891161	0.98	0.859	4.45E-289

TUBA1B+ H2AFZ+ HMGB2+ HIST1H4C+	ERH	8.88E-281	0.633128444	0.943	0.755	1.89E-276
TUBA1B+ H2AFZ+ HMGB2+ HIST1H4C+	CCNA2	0	0.632454873	0.464	0.011	0
TUBA1B+ H2AFZ+ HMGB2+ HIST1H4C+	JPT1	1.14E-247	0.628168219	0.944	0.697	2.43E-243
TUBA1B+ H2AFZ+ HMGB2+ HIST1H4C+	TCP1	3.00E-293	0.627019631	0.868	0.557	6.38E-289
TUBA1B+ H2AFZ+ HMGB2+ HIST1H4C+	SLBP	2.39E-307	0.620192257	0.68	0.305	5.08E-303
TUBA1B+ H2AFZ+ HMGB2+ HIST1H4C+	PSMA4	7.67E-230	0.619513211	0.923	0.724	1.63E-225
TUBA1B+ H2AFZ+ HMGB2+ HIST1H4C+	CCT2	7.15E-286	0.619315208	0.899	0.605	1.52E-281
TUBA1B+ H2AFZ+ HMGB2+ HIST1H4C+	EIF2S2	4.74E-262	0.614230949	0.953	0.739	1.01E-257
TUBA1B+ H2AFZ+ HMGB2+ HIST1H4C+	MINOS1	0	0.609964199	0.99	0.865	0
TUBA1B+ H2AFZ+ HMGB2+ HIST1H4C+	MCM3	0	0.606668352	0.638	0.178	0
TUBA1B+ H2AFZ+ HMGB2+ HIST1H4C+	TXN	6.44E-305	0.603868569	0.994	0.909	1.37E-300
TUBA1B+ H2AFZ+ HMGB2+ HIST1H4C+	TKT	6.84E-251	0.603831561	0.948	0.695	1.45E-246
TUBA1B+ H2AFZ+ HMGB2+ HIST1H4C+	ATP5MC1	1.69E-214	0.603003358	0.957	0.745	3.60E-210
TUBA1B+ H2AFZ+ HMGB2+ HIST1H4C+	COTL1	2.55E-303	0.602702769	0.769	0.374	5.42E-299
TUBA1B+ H2AFZ+ HMGB2+ HIST1H4C+	NUDT1	0	0.602358994	0.735	0.204	0
TUBA1B+ H2AFZ+ HMGB2+ HIST1H4C+	CENPK	0	0.601563568	0.566	0.055	0
TUBA1B+ H2AFZ+ HMGB2+ HIST1H4C+	PSMB2	1.08E-290	0.601355434	0.927	0.638	2.29E-286
TUBA1B+ H2AFZ+ HMGB2+ HIST1H4C+	RAB5IF	9.06E-209	0.600081157	0.901	0.652	1.93E-204
TUBA1B+ H2AFZ+ HMGB2+ HIST1H4C+	SLIRP	4.98E-247	0.598872481	0.95	0.791	1.06E-242
TUBA1B+ H2AFZ+ HMGB2+ HIST1H4C+	HSPA8	1.57E-144	0.595492501	0.97	0.815	3.33E-140
TUBA1B+ H2AFZ+ HMGB2+ HIST1H4C+	HNRNPF	3.59E-275	0.594141768	0.944	0.728	7.64E-271
TUBA1B+ H2AFZ+ HMGB2+ HIST1H4C+	CA9	2.13E-140	0.593709472	0.451	0.196	4.53E-136

TUBA1B+ H2AFZ+ HMGB2+ HIST1H4C+	HMMR	0	0.593323887	0.393	0.015	0
TUBA1B+ H2AFZ+ HMGB2+ HIST1H4C+	SRSF2	2.81E-263	0.592740515	0.919	0.692	5.98E-259
TUBA1B+ H2AFZ+ HMGB2+ HIST1H4C+	PBK	0	0.5923109	0.461	0.012	0
TUBA1B+ H2AFZ+ HMGB2+ HIST1H4C+	ANP32E	0	0.59204176	0.632	0.206	0
TUBA1B+ H2AFZ+ HMGB2+ HIST1H4C+	PGK1	1.38E-191	0.591357097	0.937	0.728	2.94E-187
TUBA1B+ H2AFZ+ HMGB2+ HIST1H4C+	HNRNPD	1.10E-256	0.591200696	0.858	0.576	2.34E-252
TUBA1B+ H2AFZ+ HMGB2+ HIST1H4C+	TAGLN2	2.11E-278	0.589934709	0.966	0.767	4.49E-274
TUBA1B+ H2AFZ+ HMGB2+ HIST1H4C+	ATAD2	0	0.589832946	0.504	0.067	0
TUBA1B+ H2AFZ+ HMGB2+ HIST1H4C+	HNRNPR	2.77E-271	0.589532533	0.881	0.575	5.90E-267
TUBA1B+ H2AFZ+ HMGB2+ HIST1H4C+	SKA2	0	0.58573119	0.664	0.177	0
TUBA1B+ H2AFZ+ HMGB2+ HIST1H4C+	DNAJC9	0	0.584462299	0.677	0.203	0
TUBA1B+ H2AFZ+ HMGB2+ HIST1H4C+	HNRNPM	2.08E-230	0.58437823	0.885	0.638	4.42E-226
TUBA1B+ H2AFZ+ HMGB2+ HIST1H4C+	LDHA	2.43E-161	0.581721444	0.978	0.864	5.16E-157
TUBA1B+ H2AFZ+ HMGB2+ HIST1H4C+	KIF20B	0	0.581592852	0.53	0.068	0
TUBA1B+ H2AFZ+ HMGB2+ HIST1H4C+	MRPL51	4.97E-254	0.581129011	0.929	0.704	1.06E-249
TUBA1B+ H2AFZ+ HMGB2+ HIST1H4C+	PRKDC	6.10E-308	0.580956702	0.792	0.412	1.30E-303
TUBA1B+ H2AFZ+ HMGB2+ HIST1H4C+	AP2S1	1.34E-287	0.579332365	0.948	0.726	2.84E-283
TUBA1B+ H2AFZ+ HMGB2+ HIST1H4C+	SNRPE	4.78E-274	0.579110933	0.968	0.768	1.02E-269
TUBA1B+ H2AFZ+ HMGB2+ HIST1H4C+	HELLS	0	0.579050465	0.501	0.061	0
TUBA1B+ H2AFZ+ HMGB2+ HIST1H4C+	CCDC34	0	0.578780213	0.724	0.255	0
TUBA1B+ H2AFZ+ HMGB2+ HIST1H4C+	PRELID1	1.69E-236	0.577563746	0.957	0.809	3.58E-232
TUBA1B+ H2AFZ+ HMGB2+ HIST1H4C+	TALDO1	6.80E-267	0.577228055	0.946	0.72	1.45E-262

TUBA1B+ H2AFZ+ HMGB2+ HIST1H4C+	PARK7	4.02E-272	0.576426332	0.964	0.776	8.55E-268
TUBA1B+ H2AFZ+ HMGB2+ HIST1H4C+	IFITM1	4.57E-123	0.57313291	0.801	0.52	9.71E-119
TUBA1B+ H2AFZ+ HMGB2+ HIST1H4C+	MYBL2	0	0.571742468	0.461	0.014	0
TUBA1B+ H2AFZ+ HMGB2+ HIST1H4C+	HMGA1	3.19E-261	0.571270774	0.931	0.663	6.78E-257
TUBA1B+ H2AFZ+ HMGB2+ HIST1H4C+	CYCS	5.87E-224	0.57034284	0.982	0.824	1.25E-219
TUBA1B+ H2AFZ+ HMGB2+ HIST1H4C+	GCHFR	2.65E-283	0.569407531	0.743	0.347	5.64E-279
TUBA1B+ H2AFZ+ HMGB2+ HIST1H4C+	ATP5ME	3.32E-220	0.567701859	0.987	0.905	7.06E-216
TUBA1B+ H2AFZ+ HMGB2+ HIST1H4C+	GAPDH	2.64E-250	0.566146195	1	0.973	5.61E-246
TUBA1B+ H2AFZ+ HMGB2+ HIST1H4C+	PPM1G	8.20E-292	0.565099314	0.86	0.535	1.74E-287
TUBA1B+ H2AFZ+ HMGB2+ HIST1H4C+	C1QBP	8.76E-198	0.562961371	0.93	0.695	1.86E-193
TUBA1B+ H2AFZ+ HMGB2+ HIST1H4C+	ASPM	0	0.561580423	0.367	0.009	0
TUBA1B+ H2AFZ+ HMGB2+ HIST1H4C+	CXCL3	5.55E-96	0.561122126	0.597	0.349	1.18E-91
TUBA1B+ H2AFZ+ HMGB2+ HIST1H4C+	PDCD5	9.18E-268	0.559747546	0.93	0.661	1.95E-263
TUBA1B+ H2AFZ+ HMGB2+ HIST1H4C+	IFITM3	3.38E-119	0.559049652	0.929	0.721	7.19E-115
TUBA1B+ H2AFZ+ HMGB2+ HIST1H4C+	TMPO	0	0.558474793	0.754	0.355	0
TUBA1B+ H2AFZ+ HMGB2+ HIST1H4C+	SQLE	1.34E-196	0.557931402	0.662	0.326	2.86E-192
TUBA1B+ H2AFZ+ HMGB2+ HIST1H4C+	H2AFX	0	0.556316102	0.592	0.174	0
TUBA1B+ H2AFZ+ HMGB2+ HIST1H4C+	YWHAH	3.74E-257	0.555476115	0.895	0.608	7.95E-253
TUBA1B+ H2AFZ+ HMGB2+ HIST1H4C+	NHP2	6.71E-247	0.552258645	0.935	0.695	1.43E-242
TUBA1B+ H2AFZ+ HMGB2+ HIST1H4C+	CENPN	0	0.55222756	0.595	0.092	0
TUBA1B+ H2AFZ+ HMGB2+ HIST1H4C+	PRDX1	8.87E-190	0.548891204	0.974	0.838	1.89E-185
TUBA1B+ H2AFZ+ HMGB2+ HIST1H4C+	NUDC	1.63E-244	0.546900436	0.869	0.604	3.46E-240

TUBA1B+ H2AFZ+ HMGB2+ HIST1H4C+	UQCRH	1.77E-269	0.546653545	0.991	0.883	3.76E-265
TUBA1B+ H2AFZ+ HMGB2+ HIST1H4C+	PAICS	4.32E-279	0.546508298	0.743	0.381	9.19E-275
TUBA1B+ H2AFZ+ HMGB2+ HIST1H4C+	RAD51AP1	0	0.545788277	0.486	0.022	0
TUBA1B+ H2AFZ+ HMGB2+ HIST1H4C+	ILF2	8.06E-237	0.545775266	0.859	0.567	1.71E-232
TUBA1B+ H2AFZ+ HMGB2+ HIST1H4C+	CBX3	1.42E-240	0.545266067	0.946	0.7	3.02E-236
TUBA1B+ H2AFZ+ HMGB2+ HIST1H4C+	YWHAQ	2.97E-256	0.543799886	0.936	0.697	6.32E-252
TUBA1B+ H2AFZ+ HMGB2+ HIST1H4C+	TMA7	5.39E-304	0.539721047	0.994	0.931	1.15E-299
TUBA1B+ H2AFZ+ HMGB2+ HIST1H4C+	PSMA3	7.53E-238	0.539716026	0.88	0.607	1.60E-233
TUBA1B+ H2AFZ+ HMGB2+ HIST1H4C+	RRM1	0	0.539412327	0.615	0.155	0
TUBA1B+ H2AFZ+ HMGB2+ HIST1H4C+	TIMM13	9.48E-200	0.539377349	0.92	0.741	2.02E-195
TUBA1B+ H2AFZ+ HMGB2+ HIST1H4C+	HLA-DRA	2.63E-92	0.535325359	0.564	0.336	5.59E-88
TUBA1B+ H2AFZ+ HMGB2+ HIST1H4C+	FEN1	0	0.534007985	0.5	0.067	0
TUBA1B+ H2AFZ+ HMGB2+ HIST1H4C+	SRSF7	2.83E-225	0.533864993	0.902	0.661	6.02E-221
TUBA1B+ H2AFZ+ HMGB2+ HIST1H4C+	ELOC	1.04E-225	0.533766047	0.926	0.683	2.21E-221
TUBA1B+ H2AFZ+ HMGB2+ HIST1H4C+	SLC25A5	7.15E-223	0.533761249	0.987	0.88	1.52E-218
TUBA1B+ H2AFZ+ HMGB2+ HIST1H4C+	DDX39A	0	0.532291513	0.701	0.293	0
TUBA1B+ H2AFZ+ HMGB2+ HIST1H4C+	NDUFS8	5.71E-251	0.531866182	0.941	0.696	1.21E-246
TUBA1B+ H2AFZ+ HMGB2+ HIST1H4C+	GSTO1	7.98E-237	0.530776165	0.915	0.645	1.70E-232
TUBA1B+ H2AFZ+ HMGB2+ HIST1H4C+	USP1	2.98E-276	0.529270563	0.718	0.346	6.34E-272
TUBA1B+ H2AFZ+ HMGB2+ HIST1H4C+	CDK4	1.85E-263	0.527632999	0.8	0.445	3.94E-259
TUBA1B+ H2AFZ+ HMGB2+ HIST1H4C+	HNRNPC	1.38E-207	0.526273688	0.954	0.777	2.94E-203
TUBA1B+ H2AFZ+ HMGB2+ HIST1H4C+	ECHS1	6.65E-235	0.526086623	0.932	0.709	1.41E-230

TUBA1B+ H2AFZ+ HMGB2+ HIST1H4C+	PRMT1	1.73E-235	0.525408861	0.808	0.5	3.68E-231
TUBA1B+ H2AFZ+ HMGB2+ HIST1H4C+	CCT6A	2.81E-195	0.525251682	0.905	0.667	5.98E-191
TUBA1B+ H2AFZ+ HMGB2+ HIST1H4C+	SNRNP25	0	0.524829058	0.727	0.269	0
TUBA1B+ H2AFZ+ HMGB2+ HIST1H4C+	SLC25A39	3.53E-217	0.524466314	0.851	0.572	7.51E-213
TUBA1B+ H2AFZ+ HMGB2+ HIST1H4C+	CCT7	3.95E-215	0.523989303	0.867	0.595	8.40E-211
TUBA1B+ H2AFZ+ HMGB2+ HIST1H4C+	CNIH4	3.40E-218	0.523892383	0.832	0.512	7.23E-214
TUBA1B+ H2AFZ+ HMGB2+ HIST1H4C+	KPNB1	5.90E-235	0.52258586	0.825	0.53	1.25E-230
TUBA1B+ H2AFZ+ HMGB2+ HIST1H4C+	NOP56	2.59E-262	0.521300343	0.786	0.427	5.51E-258
TUBA1B+ H2AFZ+ HMGB2+ HIST1H4C+	AURKA	0	0.521294501	0.419	0.064	0
TUBA1B+ H2AFZ+ HMGB2+ HIST1H4C+	NUDT5	3.42E-291	0.521173467	0.819	0.449	7.26E-287
TUBA1B+ H2AFZ+ HMGB2+ HIST1H4C+	RBBP7	9.48E-280	0.520365695	0.834	0.479	2.02E-275
TUBA1B+ H2AFZ+ HMGB2+ HIST1H4C+	NDUFS6	1.52E-239	0.519261565	0.97	0.804	3.24E-235
TUBA1B+ H2AFZ+ HMGB2+ HIST1H4C+	AHCY	3.29E-215	0.518776476	0.812	0.507	7.00E-211
TUBA1B+ H2AFZ+ HMGB2+ HIST1H4C+	PSIP1	0	0.518523418	0.649	0.221	0
TUBA1B+ H2AFZ+ HMGB2+ HIST1H4C+	MRPL13	4.75E-230	0.517897345	0.892	0.611	1.01E-225
TUBA1B+ H2AFZ+ HMGB2+ HIST1H4C+	TMEM97	0	0.517126436	0.655	0.215	0
TUBA1B+ H2AFZ+ HMGB2+ HIST1H4C+	SYNCRIP	1.44E-229	0.516810472	0.853	0.568	3.07E-225
TUBA1B+ H2AFZ+ HMGB2+ HIST1H4C+	NDUFC2	1.16E-214	0.516084648	0.964	0.812	2.47E-210
TUBA1B+ H2AFZ+ HMGB2+ HIST1H4C+	DDX21	6.04E-217	0.515834085	0.879	0.597	1.28E-212
TUBA1B+ H2AFZ+ HMGB2+ HIST1H4C+	BRI3BP	5.76E-300	0.515475231	0.77	0.365	1.23E-295
TUBA1B+ H2AFZ+ HMGB2+ HIST1H4C+	UBE2N	5.69E-258	0.515114322	0.887	0.565	1.21E-253
TUBA1B+ H2AFZ+ HMGB2+ HIST1H4C+	PSMA5	5.50E-228	0.514268265	0.901	0.647	1.17E-223

TUBA1B+ H2AFZ+ HMGB2+ HIST1H4C+	POLD2	1.27E-270	0.512546523	0.805	0.44	2.71E-266
TUBA1B+ H2AFZ+ HMGB2+ HIST1H4C+	BANF1	3.50E-233	0.512345427	0.941	0.694	7.44E-229
TUBA1B+ H2AFZ+ HMGB2+ HIST1H4C+	CCT8	1.45E-193	0.511557344	0.897	0.652	3.09E-189
TUBA1B+ H2AFZ+ HMGB2+ HIST1H4C+	MZT1	0	0.51065234	0.677	0.269	0
TUBA1B+ H2AFZ+ HMGB2+ HIST1H4C+	SSRP1	4.50E-271	0.510374802	0.83	0.464	9.57E-267
TUBA1B+ H2AFZ+ HMGB2+ HIST1H4C+	PHB	9.82E-194	0.509289598	0.917	0.714	2.09E-189
TUBA1B+ H2AFZ+ HMGB2+ HIST1H4C+	NAP1L1	4.05E-180	0.508947889	0.941	0.669	8.61E-176
TUBA1B+ H2AFZ+ HMGB2+ HIST1H4C+	HDGF	5.43E-232	0.508826611	0.863	0.566	1.16E-227
TUBA1B+ H2AFZ+ HMGB2+ HIST1H4C+	TMEM106C	2.01E-194	0.508715207	0.929	0.697	4.28E-190
TUBA1B+ H2AFZ+ HMGB2+ HIST1H4C+	NDUFS5	1.10E-218	0.507981806	0.984	0.836	2.34E-214
TUBA1B+ H2AFZ+ HMGB2+ HIST1H4C+	HSPA9	2.45E-209	0.5077415	0.869	0.598	5.21E-205
TUBA1B+ H2AFZ+ HMGB2+ HIST1H4C+	EIF4EBP1	1.61E-210	0.505991986	0.802	0.455	3.42E-206
TUBA1B+ H2AFZ+ HMGB2+ HIST1H4C+	EIF1AX	5.77E-177	0.505724498	0.953	0.75	1.23E-172
TUBA1B+ H2AFZ+ HMGB2+ HIST1H4C+	DCTPP1	3.54E-209	0.505398301	0.875	0.602	7.54E-205
TUBA1B+ H2AFZ+ HMGB2+ HIST1H4C+	CSTB	3.10E-89	0.503895189	0.96	0.833	6.59E-85
TUBA1B+ H2AFZ+ HMGB2+ HIST1H4C+	NAA20	5.35E-152	0.502672785	0.794	0.507	1.14E-147
TUBA1B+ H2AFZ+ HMGB2+ HIST1H4C+	BOLA3	2.23E-261	0.502176397	0.877	0.522	4.75E-257
TUBA1B+ H2AFZ+ HMGB2+ HIST1H4C+	PDIA6	1.06E-197	0.502088561	0.956	0.77	2.26E-193
TUBA1B+ H2AFZ+ HMGB2+ HIST1H4C+	CHCHD2	8.07E-271	0.50002598	0.996	0.92	1.72E-266
TUBA1B+ H2AFZ+ HMGB2+ HIST1H4C+	TPRKB	7.31E-277	0.499346188	0.845	0.484	1.55E-272
TUBA1B+ H2AFZ+ HMGB2+ HIST1H4C+	YWHAB	2.14E-151	0.498546205	0.97	0.84	4.55E-147
TUBA1B+ H2AFZ+ HMGB2+ HIST1H4C+	ATP5F1B	9.99E-192	0.497481177	0.978	0.853	2.12E-187

TUBA1B+ H2AFZ+ HMGB2+ HIST1H4C+	CDT1	0	0.49287165	0.5	0.051	0
TUBA1B+ H2AFZ+ HMGB2+ HIST1H4C+	ARPC5L	8.79E-234	0.492222254	0.86	0.545	1.87E-229
TUBA1B+ H2AFZ+ HMGB2+ HIST1H4C+	CALR	3.01E-171	0.491805962	0.969	0.846	6.40E-167
TUBA1B+ H2AFZ+ HMGB2+ HIST1H4C+	ASF1B	0	0.490866593	0.449	0.015	0
TUBA1B+ H2AFZ+ HMGB2+ HIST1H4C+	MCM4	0	0.490603597	0.518	0.08	0
TUBA1B+ H2AFZ+ HMGB2+ HIST1H4C+	CKAP2	0	0.490414504	0.489	0.138	0
TUBA1B+ H2AFZ+ HMGB2+ HIST1H4C+	MDH1	5.48E-196	0.489522901	0.852	0.581	1.16E-191
TUBA1B+ H2AFZ+ HMGB2+ HIST1H4C+	MRPS34	6.70E-217	0.489477346	0.926	0.69	1.42E-212
TUBA1B+ H2AFZ+ HMGB2+ HIST1H4C+	LBR	2.72E-237	0.489202924	0.759	0.397	5.78E-233
TUBA1B+ H2AFZ+ HMGB2+ HIST1H4C+	PSMD14	8.47E-271	0.487747109	0.809	0.437	1.80E-266
TUBA1B+ H2AFZ+ HMGB2+ HIST1H4C+	TRMT112	6.38E-194	0.487167165	0.93	0.702	1.36E-189
TUBA1B+ H2AFZ+ HMGB2+ HIST1H4C+	PPIH	0	0.486187781	0.722	0.288	0
TUBA1B+ H2AFZ+ HMGB2+ HIST1H4C+	SPC25	0	0.485448821	0.383	0.005	0
TUBA1B+ H2AFZ+ HMGB2+ HIST1H4C+	PEBP1	1.48E-185	0.485244413	0.975	0.795	3.15E-181
TUBA1B+ H2AFZ+ HMGB2+ HIST1H4C+	CSE1L	0	0.482254881	0.617	0.2	0
TUBA1B+ H2AFZ+ HMGB2+ HIST1H4C+	PAFAH1B3	4.65E-237	0.482213848	0.831	0.47	9.90E-233
TUBA1B+ H2AFZ+ HMGB2+ HIST1H4C+	EIF6	1.05E-149	0.481862487	0.912	0.715	2.23E-145
TUBA1B+ H2AFZ+ HMGB2+ HIST1H4C+	PSMB3	3.33E-193	0.481047174	0.928	0.724	7.08E-189
TUBA1B+ H2AFZ+ HMGB2+ HIST1H4C+	SMS	1.38E-224	0.480871791	0.882	0.59	2.93E-220
TUBA1B+ H2AFZ+ HMGB2+ HIST1H4C+	HINT1	1.70E-224	0.480071659	0.995	0.906	3.61E-220
TUBA1B+ H2AFZ+ HMGB2+ HIST1H4C+	HNRNPA1	1.62E-165	0.480010411	0.998	0.884	3.44E-161
TUBA1B+ H2AFZ+ HMGB2+ HIST1H4C+	EBNA1BP2	4.91E-224	0.479863992	0.793	0.47	1.04E-219

TUBA1B+ H2AFZ+ HMGB2+ HIST1H4C+	CBX5	3.39E-296	0.478877551	0.702	0.304	7.20E-292
TUBA1B+ H2AFZ+ HMGB2+ HIST1H4C+	CCT4	5.50E-202	0.478115481	0.867	0.589	1.17E-197
TUBA1B+ H2AFZ+ HMGB2+ HIST1H4C+	AURKB	0	0.476578026	0.368	0.007	0
TUBA1B+ H2AFZ+ HMGB2+ HIST1H4C+	NAA38	3.92E-132	0.475925118	0.864	0.635	8.34E-128
TUBA1B+ H2AFZ+ HMGB2+ HIST1H4C+	ACTB	1.65E-255	0.474803181	1	0.983	3.51E-251
TUBA1B+ H2AFZ+ HMGB2+ HIST1H4C+	PARP1	8.91E-233	0.474172066	0.789	0.437	1.89E-228
TUBA1B+ H2AFZ+ HMGB2+ HIST1H4C+	SUB1	8.77E-207	0.473900474	0.979	0.868	1.87E-202
TUBA1B+ H2AFZ+ HMGB2+ HIST1H4C+	UQCRQ	1.11E-212	0.473481792	0.995	0.918	2.35E-208
TUBA1B+ H2AFZ+ HMGB2+ HIST1H4C+	ATP5MF	7.76E-215	0.473240621	0.984	0.877	1.65E-210
TUBA1B+ H2AFZ+ HMGB2+ HIST1H4C+	POLR2L	1.18E-196	0.471497373	0.981	0.852	2.51E-192
TUBA1B+ H2AFZ+ HMGB2+ HIST1H4C+	HADH	1.48E-223	0.467408381	0.834	0.5	3.14E-219
TUBA1B+ H2AFZ+ HMGB2+ HIST1H4C+	SAPCD2	0	0.466968259	0.499	0.094	0
TUBA1B+ H2AFZ+ HMGB2+ HIST1H4C+	IDI1	6.31E-180	0.466413266	0.72	0.384	1.34E-175
TUBA1B+ H2AFZ+ HMGB2+ HIST1H4C+	TUFM	4.48E-168	0.465962196	0.946	0.759	9.52E-164
TUBA1B+ H2AFZ+ HMGB2+ HIST1H4C+	NOP58	3.06E-242	0.464992539	0.78	0.423	6.51E-238
TUBA1B+ H2AFZ+ HMGB2+ HIST1H4C+	EIF3I	1.41E-175	0.464177767	0.917	0.693	3.00E-171
TUBA1B+ H2AFZ+ HMGB2+ HIST1H4C+	HSP90B1	1.26E-158	0.46393253	0.968	0.841	2.69E-154
TUBA1B+ H2AFZ+ HMGB2+ HIST1H4C+	SRP9	9.24E-198	0.462769467	0.961	0.788	1.97E-193
TUBA1B+ H2AFZ+ HMGB2+ HIST1H4C+	PAXX	4.52E-232	0.462143122	0.807	0.467	9.61E-228
TUBA1B+ H2AFZ+ HMGB2+ HIST1H4C+	MRPS15	2.12E-212	0.461581176	0.851	0.56	4.50E-208
TUBA1B+ H2AFZ+ HMGB2+ HIST1H4C+	CHEK1	0	0.459901264	0.54	0.085	0
TUBA1B+ H2AFZ+ HMGB2+ HIST1H4C+	CENPU	0	0.459894254	0.45	0.029	0

TUBA1B+ H2AFZ+ HMGB2+ HIST1H4C+	FDFT1	2.12E-149	0.458970272	0.815	0.536	4.51E-145
TUBA1B+ H2AFZ+ HMGB2+ HIST1H4C+	POLR3K	1.71E-297	0.458480425	0.666	0.269	3.63E-293
TUBA1B+ H2AFZ+ HMGB2+ HIST1H4C+	SLC25A3	3.57E-192	0.458323572	0.977	0.869	7.58E-188
TUBA1B+ H2AFZ+ HMGB2+ HIST1H4C+	PFN1	9.94E-180	0.458145043	0.996	0.933	2.11E-175
TUBA1B+ H2AFZ+ HMGB2+ HIST1H4C+	RPS21	1.85E-164	0.457286467	0.997	0.911	3.94E-160
TUBA1B+ H2AFZ+ HMGB2+ HIST1H4C+	SNRPA1	4.26E-235	0.457248927	0.728	0.382	9.07E-231
TUBA1B+ H2AFZ+ HMGB2+ HIST1H4C+	CALM3	8.89E-197	0.456538253	0.907	0.666	1.89E-192
TUBA1B+ H2AFZ+ HMGB2+ HIST1H4C+	DDT	6.30E-157	0.45639749	0.921	0.756	1.34E-152
TUBA1B+ H2AFZ+ HMGB2+ HIST1H4C+	TECR	2.28E-171	0.456168079	0.874	0.647	4.85E-167
TUBA1B+ H2AFZ+ HMGB2+ HIST1H4C+	SRSF9	4.49E-192	0.455782727	0.948	0.754	9.55E-188
TUBA1B+ H2AFZ+ HMGB2+ HIST1H4C+	RPSA	5.10E-176	0.455713541	0.999	0.885	1.09E-171
TUBA1B+ H2AFZ+ HMGB2+ HIST1H4C+	MTCH2	8.88E-205	0.455057882	0.89	0.622	1.89E-200
TUBA1B+ H2AFZ+ HMGB2+ HIST1H4C+	COX8A	6.37E-207	0.454213341	0.99	0.906	1.35E-202
TUBA1B+ H2AFZ+ HMGB2+ HIST1H4C+	ADRM1	3.74E-171	0.453484113	0.85	0.579	7.96E-167
TUBA1B+ H2AFZ+ HMGB2+ HIST1H4C+	VPS29	1.13E-192	0.453460622	0.907	0.672	2.41E-188
TUBA1B+ H2AFZ+ HMGB2+ HIST1H4C+	BCL2L12	0	0.453241386	0.608	0.168	0
TUBA1B+ H2AFZ+ HMGB2+ HIST1H4C+	PRC1	0	0.452452105	0.413	0.019	0
TUBA1B+ H2AFZ+ HMGB2+ HIST1H4C+	GCSH	4.40E-228	0.451513501	0.791	0.443	9.35E-224
TUBA1B+ H2AFZ+ HMGB2+ HIST1H4C+	ATP5MG	6.19E-181	0.45134893	0.994	0.92	1.32E-176
TUBA1B+ H2AFZ+ HMGB2+ HIST1H4C+	COX5A	4.89E-171	0.451147581	0.979	0.879	1.04E-166
TUBA1B+ H2AFZ+ HMGB2+ HIST1H4C+	FAM111B	0	0.450827973	0.386	0.021	0
TUBA1B+ H2AFZ+ HMGB2+ HIST1H4C+	XRCC6	7.86E-162	0.449605382	0.851	0.61	1.67E-157

TUBA1B+ H2AFZ+ HMGB2+ HIST1H4C+	ECT2	0	0.449580481	0.519	0.117	0
TUBA1B+ H2AFZ+ HMGB2+ HIST1H4C+	TFDP1	1.55E-264	0.449227336	0.69	0.312	3.30E-260
TUBA1B+ H2AFZ+ HMGB2+ HIST1H4C+	TRAP1	1.68E-232	0.449196387	0.754	0.408	3.58E-228
TUBA1B+ H2AFZ+ HMGB2+ HIST1H4C+	PSMB1	1.42E-185	0.448794344	0.965	0.814	3.01E-181
TUBA1B+ H2AFZ+ HMGB2+ HIST1H4C+	ATP5MD	8.42E-195	0.448216708	0.991	0.909	1.79E-190
TUBA1B+ H2AFZ+ HMGB2+ HIST1H4C+	HPRT1	1.09E-274	0.447936224	0.703	0.322	2.33E-270
TUBA1B+ H2AFZ+ HMGB2+ HIST1H4C+	PCBD1	9.07E-183	0.446764748	0.944	0.737	1.93E-178
TUBA1B+ H2AFZ+ HMGB2+ HIST1H4C+	MRPL17	5.95E-257	0.446638462	0.756	0.378	1.27E-252
TUBA1B+ H2AFZ+ HMGB2+ HIST1H4C+	CD74	3.93E-74	0.446427201	0.786	0.568	8.36E-70
TUBA1B+ H2AFZ+ HMGB2+ HIST1H4C+	TNFRSF12A	1.85E-175	0.445982298	0.66	0.326	3.94E-171
TUBA1B+ H2AFZ+ HMGB2+ HIST1H4C+	MPC2	1.16E-168	0.445975123	0.96	0.791	2.46E-164
TUBA1B+ H2AFZ+ HMGB2+ HIST1H4C+	MANF	3.41E-208	0.44593347	0.862	0.543	7.26E-204
TUBA1B+ H2AFZ+ HMGB2+ HIST1H4C+	ENSA	3.61E-211	0.445552227	0.925	0.685	7.67E-207
TUBA1B+ H2AFZ+ HMGB2+ HIST1H4C+	TIMM8B	9.41E-189	0.444935901	0.932	0.697	2.00E-184
TUBA1B+ H2AFZ+ HMGB2+ HIST1H4C+	MRPL12	1.51E-157	0.444832991	0.873	0.646	3.21E-153
TUBA1B+ H2AFZ+ HMGB2+ HIST1H4C+	LRRC59	2.30E-204	0.444041009	0.804	0.495	4.90E-200
TUBA1B+ H2AFZ+ HMGB2+ HIST1H4C+	EEF1B2	3.67E-168	0.443945387	0.993	0.876	7.80E-164
TUBA1B+ H2AFZ+ HMGB2+ HIST1H4C+	ATP5F1C	1.42E-176	0.443460696	0.971	0.807	3.03E-172
TUBA1B+ H2AFZ+ HMGB2+ HIST1H4C+	LYAR	0	0.442936331	0.577	0.196	0
TUBA1B+ H2AFZ+ HMGB2+ HIST1H4C+	SNRPD2	6.85E-179	0.442311937	0.984	0.834	1.46E-174
TUBA1B+ H2AFZ+ HMGB2+ HIST1H4C+	PPA1	1.06E-129	0.441482133	0.971	0.81	2.25E-125
TUBA1B+ H2AFZ+ HMGB2+ HIST1H4C+	MRPL11	4.61E-206	0.441408638	0.866	0.567	9.81E-202

TUBA1B+ H2AFZ+ HMGB2+ HIST1H4C+	SNRPD3	3.17E-197	0.441269129	0.876	0.619	6.73E-193
TUBA1B+ H2AFZ+ HMGB2+ HIST1H4C+	CHCHD3	1.74E-198	0.438959059	0.782	0.465	3.70E-194
TUBA1B+ H2AFZ+ HMGB2+ HIST1H4C+	ANP32A	4.03E-176	0.438200752	0.807	0.53	8.57E-172
TUBA1B+ H2AFZ+ HMGB2+ HIST1H4C+	H2AFY	1.27E-178	0.437965439	0.946	0.782	2.70E-174
TUBA1B+ H2AFZ+ HMGB2+ HIST1H4C+	SSB	6.97E-167	0.437923154	0.886	0.64	1.48E-162
TUBA1B+ H2AFZ+ HMGB2+ HIST1H4C+	LSM2	5.51E-199	0.4364694	0.863	0.552	1.17E-194
TUBA1B+ H2AFZ+ HMGB2+ HIST1H4C+	MRPL37	4.28E-242	0.435799514	0.789	0.433	9.09E-238
TUBA1B+ H2AFZ+ HMGB2+ HIST1H4C+	FBL	6.73E-171	0.435462364	0.908	0.649	1.43E-166
TUBA1B+ H2AFZ+ HMGB2+ HIST1H4C+	PRDX3	1.96E-177	0.435433588	0.871	0.602	4.17E-173
TUBA1B+ H2AFZ+ HMGB2+ HIST1H4C+	MYC	1.46E-111	0.434140472	0.707	0.443	3.11E-107
TUBA1B+ H2AFZ+ HMGB2+ HIST1H4C+	PSMC3	6.78E-198	0.43259627	0.841	0.555	1.44E-193
TUBA1B+ H2AFZ+ HMGB2+ HIST1H4C+	ITGB3BP	0	0.431980691	0.573	0.147	0
TUBA1B+ H2AFZ+ HMGB2+ HIST1H4C+	PSMA2	2.01E-167	0.43105709	0.955	0.748	4.28E-163
TUBA1B+ H2AFZ+ HMGB2+ HIST1H4C+	XRCC5	5.89E-182	0.429794731	0.892	0.634	1.25E-177
TUBA1B+ H2AFZ+ HMGB2+ HIST1H4C+	EXOSC8	1.71E-263	0.428569465	0.696	0.308	3.63E-259
TUBA1B+ H2AFZ+ HMGB2+ HIST1H4C+	EMP2	8.53E-191	0.42848706	0.882	0.581	1.81E-186
TUBA1B+ H2AFZ+ HMGB2+ HIST1H4C+	UQCRFS1	4.79E-154	0.427908177	0.946	0.758	1.02E-149
TUBA1B+ H2AFZ+ HMGB2+ HIST1H4C+	HDAC2	4.46E-184	0.427337152	0.846	0.569	9.49E-180
TUBA1B+ H2AFZ+ HMGB2+ HIST1H4C+	GTSE1	0	0.427286363	0.344	0.008	0
TUBA1B+ H2AFZ+ HMGB2+ HIST1H4C+	CEP55	0	0.426063192	0.396	0.012	0
TUBA1B+ H2AFZ+ HMGB2+ HIST1H4C+	CMSS1	1.65E-287	0.425564742	0.61	0.228	3.51E-283
TUBA1B+ H2AFZ+ HMGB2+ HIST1H4C+	GADD45GIP1	1.21E-149	0.425475583	0.86	0.668	2.57E-145

TUBA1B+ H2AFZ+ HMGB2+ HIST1H4C+	VRK1	0	0.425462762	0.507	0.084	0
TUBA1B+ H2AFZ+ HMGB2+ HIST1H4C+	POLR2E	9.98E-188	0.424803823	0.811	0.515	2.12E-183
TUBA1B+ H2AFZ+ HMGB2+ HIST1H4C+	EEF1D	1.69E-105	0.423668289	0.987	0.898	3.59E-101
TUBA1B+ H2AFZ+ HMGB2+ HIST1H4C+	ACOT7	8.85E-295	0.422270075	0.674	0.266	1.88E-290
TUBA1B+ H2AFZ+ HMGB2+ HIST1H4C+	CENPE	0	0.422170909	0.325	0.014	0
TUBA1B+ H2AFZ+ HMGB2+ HIST1H4C+	NDUFB9	1.51E-115	0.422110885	0.956	0.808	3.21E-111
TUBA1B+ H2AFZ+ HMGB2+ HIST1H4C+	RPL35	1.38E-222	0.421120736	0.999	0.94	2.93E-218
TUBA1B+ H2AFZ+ HMGB2+ HIST1H4C+	HACD3	1.28E-179	0.420686563	0.713	0.405	2.71E-175
TUBA1B+ H2AFZ+ HMGB2+ HIST1H4C+	CISD1	1.17E-171	0.419872001	0.86	0.586	2.49E-167
TUBA1B+ H2AFZ+ HMGB2+ HIST1H4C+	BST2	2.48E-115	0.418913229	0.446	0.203	5.27E-111
TUBA1B+ H2AFZ+ HMGB2+ HIST1H4C+	ANAPC11	1.34E-143	0.418586481	0.92	0.743	2.85E-139
TUBA1B+ H2AFZ+ HMGB2+ HIST1H4C+	CLSPN	0	0.418515886	0.377	0.012	0
TUBA1B+ H2AFZ+ HMGB2+ HIST1H4C+	DAZAP1	5.99E-207	0.417479143	0.775	0.454	1.27E-202
TUBA1B+ H2AFZ+ HMGB2+ HIST1H4C+	MIS18A	0	0.417209867	0.559	0.129	0
TUBA1B+ H2AFZ+ HMGB2+ HIST1H4C+	AURKAIP1	1.57E-177	0.417015211	0.963	0.825	3.33E-173
TUBA1B+ H2AFZ+ HMGB2+ HIST1H4C+	RPN2	2.03E-143	0.416426209	0.888	0.675	4.31E-139
TUBA1B+ H2AFZ+ HMGB2+ HIST1H4C+	MRPL18	1.68E-190	0.416035065	0.861	0.569	3.57E-186
TUBA1B+ H2AFZ+ HMGB2+ HIST1H4C+	MRPL20	9.03E-173	0.415401844	0.931	0.712	1.92E-168
TUBA1B+ H2AFZ+ HMGB2+ HIST1H4C+	DPM1	1.08E-168	0.414559311	0.755	0.455	2.30E-164
TUBA1B+ H2AFZ+ HMGB2+ HIST1H4C+	POMP	1.47E-150	0.414476061	0.981	0.863	3.13E-146
TUBA1B+ H2AFZ+ HMGB2+ HIST1H4C+	NDUFV1	1.34E-171	0.414461199	0.888	0.624	2.86E-167
TUBA1B+ H2AFZ+ HMGB2+ HIST1H4C+	CENPH	0	0.412523756	0.531	0.087	0

TUBA1B+ H2AFZ+ HMGB2+ HIST1H4C+	CDCA3	0	0.412289313	0.392	0.032	0
TUBA1B+ H2AFZ+ HMGB2+ HIST1H4C+	MTHFD2	9.75E-255	0.412119986	0.695	0.307	2.07E-250
TUBA1B+ H2AFZ+ HMGB2+ HIST1H4C+	PPA2	9.62E-172	0.411795648	0.87	0.603	2.05E-167
TUBA1B+ H2AFZ+ HMGB2+ HIST1H4C+	GRPEL1	3.38E-199	0.409582933	0.659	0.337	7.20E-195
TUBA1B+ H2AFZ+ HMGB2+ HIST1H4C+	P4HB	3.15E-118	0.409513232	0.941	0.81	6.70E-114
TUBA1B+ H2AFZ+ HMGB2+ HIST1H4C+	SSBP1	3.43E-153	0.408605679	0.938	0.726	7.29E-149
TUBA1B+ H2AFZ+ HMGB2+ HIST1H4C+	PDHA1	1.59E-189	0.408441067	0.828	0.524	3.38E-185
TUBA1B+ H2AFZ+ HMGB2+ HIST1H4C+	HNRNPDL	2.18E-150	0.408333812	0.895	0.665	4.63E-146
TUBA1B+ H2AFZ+ HMGB2+ HIST1H4C+	SUPT16H	1.19E-195	0.407027246	0.679	0.356	2.54E-191
TUBA1B+ H2AFZ+ HMGB2+ HIST1H4C+	NUTF2	5.17E-188	0.40665484	0.849	0.54	1.10E-183
TUBA1B+ H2AFZ+ HMGB2+ HIST1H4C+	PSMD7	2.37E-163	0.405996172	0.865	0.614	5.03E-159
TUBA1B+ H2AFZ+ HMGB2+ HIST1H4C+	SGO1	0	0.4052508	0.401	0.018	0
TUBA1B+ H2AFZ+ HMGB2+ HIST1H4C+	RUVBL2	6.47E-221	0.40519755	0.739	0.394	1.38E-216
TUBA1B+ H2AFZ+ HMGB2+ HIST1H4C+	ASRGL1	2.09E-238	0.405022992	0.736	0.339	4.45E-234
TUBA1B+ H2AFZ+ HMGB2+ HIST1H4C+	ATP5PF	1.19E-191	0.404972331	0.989	0.895	2.53E-187
TUBA1B+ H2AFZ+ HMGB2+ HIST1H4C+	PRDX4	1.14E-165	0.404876057	0.886	0.631	2.43E-161
TUBA1B+ H2AFZ+ HMGB2+ HIST1H4C+	PSMG1	5.02E-207	0.404550315	0.751	0.402	1.07E-202
TUBA1B+ H2AFZ+ HMGB2+ HIST1H4C+	RFC3	0	0.404005478	0.44	0.058	0
TUBA1B+ H2AFZ+ HMGB2+ HIST1H4C+	CENPA	0	0.403779384	0.309	0.007	0
TUBA1B+ H2AFZ+ HMGB2+ HIST1H4C+	ORC6	0	0.403060413	0.429	0.027	0
TUBA1B+ H2AFZ+ HMGB2+ HIST1H4C+	MAGOH	1.54E-184	0.402896749	0.844	0.55	3.27E-180
TUBA1B+ H2AFZ+ HMGB2+ HIST1H4C+	GLRX5	1.11E-158	0.402354241	0.806	0.554	2.36E-154

TUBA1B+ H2AFZ+ HMGB2+ HIST1H4C+	SDF2L1	5.54E-165	0.401817519	0.845	0.541	1.18E-160
TUBA1B+ H2AFZ+ HMGB2+ HIST1H4C+	MRPS12	1.70E-178	0.401750598	0.856	0.573	3.62E-174
TUBA1B+ H2AFZ+ HMGB2+ HIST1H4C+	RABL6	1.35E-147	0.400918216	0.808	0.546	2.87E-143
TUBA1B+ H2AFZ+ HMGB2+ HIST1H4C+	TACC3	0	0.400471599	0.396	0.025	0
TUBA1B+ H2AFZ+ HMGB2+ HIST1H4C+	PSMD8	1.01E-155	0.400316302	0.909	0.688	2.16E-151
TUBA1B+ H2AFZ+ HMGB2+ HIST1H4C+	PSMB5	9.21E-142	0.399328353	0.882	0.668	1.96E-137
TUBA1B+ H2AFZ+ HMGB2+ HIST1H4C+	CDC123	1.00E-196	0.399015527	0.753	0.432	2.13E-192
TUBA1B+ H2AFZ+ HMGB2+ HIST1H4C+	CARHSP1	7.99E-147	0.398798287	0.86	0.589	1.70E-142
TUBA1B+ H2AFZ+ HMGB2+ HIST1H4C+	TIMM10	2.17E-180	0.398499592	0.782	0.457	4.61E-176
TUBA1B+ H2AFZ+ HMGB2+ HIST1H4C+	GSPT1	5.83E-165	0.397928283	0.87	0.623	1.24E-160
TUBA1B+ H2AFZ+ HMGB2+ HIST1H4C+	NDUFA6	7.43E-145	0.397809459	0.914	0.733	1.58E-140
TUBA1B+ H2AFZ+ HMGB2+ HIST1H4C+	PLK1	0	0.397247208	0.294	0.013	0
TUBA1B+ H2AFZ+ HMGB2+ HIST1H4C+	GTF2A2	1.93E-161	0.397143933	0.842	0.583	4.11E-157
TUBA1B+ H2AFZ+ HMGB2+ HIST1H4C+	TBCA	4.65E-159	0.396758111	0.964	0.792	9.90E-155
TUBA1B+ H2AFZ+ HMGB2+ HIST1H4C+	UBE2V2	2.01E-156	0.396125243	0.801	0.512	4.28E-152
TUBA1B+ H2AFZ+ HMGB2+ HIST1H4C+	PPIB	1.19E-129	0.396111417	0.965	0.816	2.53E-125
TUBA1B+ H2AFZ+ HMGB2+ HIST1H4C+	HLA-DRB1	4.47E-111	0.395950488	0.558	0.295	9.50E-107
TUBA1B+ H2AFZ+ HMGB2+ HIST1H4C+	RPS2	5.49E-106	0.394199978	1	0.946	1.17E-101
TUBA1B+ H2AFZ+ HMGB2+ HIST1H4C+	PPIF	3.21E-267	0.394126538	0.694	0.3	6.82E-263
TUBA1B+ H2AFZ+ HMGB2+ HIST1H4C+	CD320	5.06E-168	0.393890686	0.788	0.49	1.08E-163
TUBA1B+ H2AFZ+ HMGB2+ HIST1H4C+	TOMM5	3.43E-211	0.393472698	0.759	0.396	7.30E-207
TUBA1B+ H2AFZ+ HMGB2+ HIST1H4C+	HNRNPK	2.35E-147	0.39334669	0.969	0.839	5.00E-143

TUBA1B+ H2AFZ+ HMGB2+ HIST1H4C+	RBM3	6.88E-134	0.39290897	0.938	0.755	1.46E-129
TUBA1B+ H2AFZ+ HMGB2+ HIST1H4C+	TOMM22	4.28E-138	0.392894282	0.85	0.63	9.10E-134
TUBA1B+ H2AFZ+ HMGB2+ HIST1H4C+	KIF22	5.68E-276	0.39144051	0.549	0.187	1.21E-271
TUBA1B+ H2AFZ+ HMGB2+ HIST1H4C+	MND1	0	0.390481847	0.388	0.02	0
TUBA1B+ H2AFZ+ HMGB2+ HIST1H4C+	CNN3	4.15E-188	0.389771603	0.779	0.438	8.82E-184
TUBA1B+ H2AFZ+ HMGB2+ HIST1H4C+	UBE2I	1.43E-151	0.389155667	0.906	0.663	3.03E-147
TUBA1B+ H2AFZ+ HMGB2+ HIST1H4C+	NDUFA12	5.18E-157	0.388711912	0.938	0.734	1.10E-152
TUBA1B+ H2AFZ+ HMGB2+ HIST1H4C+	STOML2	4.11E-143	0.388612365	0.899	0.664	8.75E-139
TUBA1B+ H2AFZ+ HMGB2+ HIST1H4C+	CFL1	1.08E-194	0.388452177	0.997	0.972	2.29E-190
TUBA1B+ H2AFZ+ HMGB2+ HIST1H4C+	NUF2	0	0.388062789	0.354	0.007	0
TUBA1B+ H2AFZ+ HMGB2+ HIST1H4C+	GLRX3	2.08E-178	0.387637177	0.825	0.52	4.42E-174
TUBA1B+ H2AFZ+ HMGB2+ HIST1H4C+	TROAP	0	0.387445857	0.416	0.033	0
TUBA1B+ H2AFZ+ HMGB2+ HIST1H4C+	PSMA1	1.40E-147	0.387148653	0.934	0.717	2.97E-143
TUBA1B+ H2AFZ+ HMGB2+ HIST1H4C+	CXCL1	5.12E-64	0.386692678	0.39	0.212	1.09E-59
TUBA1B+ H2AFZ+ HMGB2+ HIST1H4C+	EEF1E1	6.10E-215	0.386196667	0.748	0.388	1.30E-210
TUBA1B+ H2AFZ+ HMGB2+ HIST1H4C+	CMC2	3.64E-205	0.385949563	0.693	0.346	7.73E-201
TUBA1B+ H2AFZ+ HMGB2+ HIST1H4C+	SCD	2.49E-131	0.385720035	0.63	0.344	5.29E-127
TUBA1B+ H2AFZ+ HMGB2+ HIST1H4C+	BCL7C	2.58E-196	0.385175151	0.762	0.412	5.48E-192
TUBA1B+ H2AFZ+ HMGB2+ HIST1H4C+	MRPL16	3.61E-170	0.384287951	0.79	0.486	7.67E-166
TUBA1B+ H2AFZ+ HMGB2+ HIST1H4C+	OLA1	6.17E-153	0.384137958	0.862	0.591	1.31E-148
TUBA1B+ H2AFZ+ HMGB2+ HIST1H4C+	SF3B5	9.15E-143	0.383110232	0.934	0.756	1.95E-138
TUBA1B+ H2AFZ+ HMGB2+ HIST1H4C+	RBX1	4.79E-155	0.382946373	0.917	0.727	1.02E-150

TUBA1B+ H2AFZ+ HMGB2+ HIST1H4C+	ENY2	1.47E-117	0.382869684	0.893	0.715	3.13E-113
TUBA1B+ H2AFZ+ HMGB2+ HIST1H4C+	LYZ	2.83E-59	0.382726422	0.75	0.569	6.01E-55
TUBA1B+ H2AFZ+ HMGB2+ HIST1H4C+	MRFAP1	3.57E-121	0.382703932	0.824	0.604	7.60E-117
TUBA1B+ H2AFZ+ HMGB2+ HIST1H4C+	GPSM2	4.09E-201	0.382681428	0.588	0.255	8.71E-197
TUBA1B+ H2AFZ+ HMGB2+ HIST1H4C+	TFRC	1.95E-145	0.382109266	0.751	0.447	4.14E-141
TUBA1B+ H2AFZ+ HMGB2+ HIST1H4C+	FXYD5	8.96E-140	0.381935575	0.644	0.341	1.91E-135
TUBA1B+ H2AFZ+ HMGB2+ HIST1H4C+	LMNB2	0	0.381303036	0.539	0.149	0
TUBA1B+ H2AFZ+ HMGB2+ HIST1H4C+	TPM3	1.61E-155	0.381038589	0.957	0.778	3.42E-151
TUBA1B+ H2AFZ+ HMGB2+ HIST1H4C+	UQCC2	1.26E-165	0.380139993	0.775	0.485	2.68E-161
TUBA1B+ H2AFZ+ HMGB2+ HIST1H4C+	EIF5B	4.13E-154	0.38001521	0.916	0.691	8.79E-150
TUBA1B+ H2AFZ+ HMGB2+ HIST1H4C+	DLGAP5	0	0.379992776	0.312	0.006	0
TUBA1B+ H2AFZ+ HMGB2+ HIST1H4C+	MRPL36	4.57E-167	0.379689144	0.808	0.512	9.71E-163
TUBA1B+ H2AFZ+ HMGB2+ HIST1H4C+	DKC1	3.88E-184	0.379650843	0.725	0.4	8.25E-180
TUBA1B+ H2AFZ+ HMGB2+ HIST1H4C+	ATP5PB	9.67E-135	0.379263543	0.958	0.773	2.06E-130
TUBA1B+ H2AFZ+ HMGB2+ HIST1H4C+	DDX46	4.01E-154	0.378406292	0.847	0.58	8.53E-150
TUBA1B+ H2AFZ+ HMGB2+ HIST1H4C+	PSMD1	9.89E-192	0.377866756	0.723	0.388	2.10E-187
TUBA1B+ H2AFZ+ HMGB2+ HIST1H4C+	AK2	8.60E-177	0.377452034	0.852	0.552	1.83E-172
TUBA1B+ H2AFZ+ HMGB2+ HIST1H4C+	S100A11	2.78E-101	0.377008449	0.985	0.881	5.92E-97
TUBA1B+ H2AFZ+ HMGB2+ HIST1H4C+	RHOA	8.91E-149	0.376889036	0.966	0.835	1.89E-144
TUBA1B+ H2AFZ+ HMGB2+ HIST1H4C+	AHSA1	6.32E-147	0.376769275	0.736	0.467	1.34E-142
TUBA1B+ H2AFZ+ HMGB2+ HIST1H4C+	BRCA1	0	0.376395152	0.395	0.034	0
TUBA1B+ H2AFZ+ HMGB2+ HIST1H4C+	NUDT8	1.05E-215	0.375687477	0.617	0.272	2.23E-211

TUBA1B+ H2AFZ+ HMGB2+ HIST1H4C+	MCM5	0	0.375369128	0.437	0.099	0
TUBA1B+ H2AFZ+ HMGB2+ HIST1H4C+	SAC3D1	9.42E-301	0.375138245	0.544	0.175	2.00E-296
TUBA1B+ H2AFZ+ HMGB2+ HIST1H4C+	COPS9	5.84E-146	0.374962287	0.933	0.735	1.24E-141
TUBA1B+ H2AFZ+ HMGB2+ HIST1H4C+	MMAB	2.15E-228	0.374944691	0.74	0.358	4.58E-224
TUBA1B+ H2AFZ+ HMGB2+ HIST1H4C+	CCDC85B	3.96E-167	0.374533515	0.726	0.406	8.42E-163
TUBA1B+ H2AFZ+ HMGB2+ HIST1H4C+	CD81	9.78E-160	0.374104204	0.753	0.441	2.08E-155
TUBA1B+ H2AFZ+ HMGB2+ HIST1H4C+	NOP16	1.50E-191	0.374009941	0.684	0.349	3.19E-187
TUBA1B+ H2AFZ+ HMGB2+ HIST1H4C+	ACP1	3.48E-143	0.373876788	0.916	0.679	7.39E-139
TUBA1B+ H2AFZ+ HMGB2+ HIST1H4C+	TIMM17A	2.21E-180	0.373785124	0.775	0.454	4.70E-176
TUBA1B+ H2AFZ+ HMGB2+ HIST1H4C+	LSM6	1.90E-166	0.373306365	0.827	0.528	4.04E-162
TUBA1B+ H2AFZ+ HMGB2+ HIST1H4C+	METTL26	1.85E-159	0.372246651	0.862	0.586	3.94E-155
TUBA1B+ H2AFZ+ HMGB2+ HIST1H4C+	CALM1	1.15E-133	0.371617042	0.98	0.87	2.44E-129
TUBA1B+ H2AFZ+ HMGB2+ HIST1H4C+	CBX1	5.61E-211	0.371345658	0.642	0.292	1.19E-206
TUBA1B+ H2AFZ+ HMGB2+ HIST1H4C+	SUCLG1	2.99E-127	0.371124949	0.9	0.678	6.35E-123
TUBA1B+ H2AFZ+ HMGB2+ HIST1H4C+	C12orf75	4.63E-152	0.370743625	0.906	0.614	9.84E-148
TUBA1B+ H2AFZ+ HMGB2+ HIST1H4C+	NOLC1	1.18E-212	0.370663364	0.643	0.3	2.50E-208
TUBA1B+ H2AFZ+ HMGB2+ HIST1H4C+	LSM7	8.59E-137	0.370477481	0.956	0.787	1.83E-132
TUBA1B+ H2AFZ+ HMGB2+ HIST1H4C+	PTPMT1	2.48E-180	0.370113118	0.744	0.417	5.28E-176
TUBA1B+ H2AFZ+ HMGB2+ HIST1H4C+	UQCRC1	1.48E-128	0.369987242	0.944	0.767	3.15E-124
TUBA1B+ H2AFZ+ HMGB2+ HIST1H4C+	CYBA	5.11E-106	0.36977182	0.982	0.869	1.09E-101
TUBA1B+ H2AFZ+ HMGB2+ HIST1H4C+	SNRPC	7.93E-147	0.369742169	0.878	0.608	1.69E-142
TUBA1B+ H2AFZ+ HMGB2+ HIST1H4C+	MRPS26	2.86E-167	0.36962461	0.796	0.508	6.09E-163

TUBA1B+ H2AFZ+ HMGB2+ HIST1H4C+	SRM	8.08E-160	0.369244696	0.701	0.391	1.72E-155
TUBA1B+ H2AFZ+ HMGB2+ HIST1H4C+	TUBA4A	8.50E-186	0.369028792	0.718	0.369	1.81E-181
TUBA1B+ H2AFZ+ HMGB2+ HIST1H4C+	GPAA1	5.52E-159	0.368879272	0.763	0.464	1.17E-154
TUBA1B+ H2AFZ+ HMGB2+ HIST1H4C+	CALM2	1.73E-85	0.368819963	0.992	0.919	3.69E-81
TUBA1B+ H2AFZ+ HMGB2+ HIST1H4C+	SRPK1	3.79E-147	0.368728633	0.867	0.595	8.05E-143
TUBA1B+ H2AFZ+ HMGB2+ HIST1H4C+	GPI	1.83E-146	0.367978422	0.858	0.579	3.89E-142
TUBA1B+ H2AFZ+ HMGB2+ HIST1H4C+	POLE3	3.38E-256	0.367914488	0.662	0.277	7.19E-252
TUBA1B+ H2AFZ+ HMGB2+ HIST1H4C+	MGST2	1.91E-123	0.367832551	0.921	0.729	4.07E-119
TUBA1B+ H2AFZ+ HMGB2+ HIST1H4C+	EIF2S1	2.06E-186	0.367239674	0.688	0.362	4.37E-182
TUBA1B+ H2AFZ+ HMGB2+ HIST1H4C+	ISOC2	6.21E-151	0.367093512	0.824	0.54	1.32E-146
TUBA1B+ H2AFZ+ HMGB2+ HIST1H4C+	PDXK	6.20E-139	0.366761464	0.774	0.495	1.32E-134
TUBA1B+ H2AFZ+ HMGB2+ HIST1H4C+	PDIA3	8.79E-109	0.36611096	0.944	0.799	1.87E-104
TUBA1B+ H2AFZ+ HMGB2+ HIST1H4C+	THOC7	3.96E-150	0.365926489	0.911	0.679	8.41E-146
TUBA1B+ H2AFZ+ HMGB2+ HIST1H4C+	PUF60	3.33E-128	0.365518606	0.773	0.508	7.09E-124
TUBA1B+ H2AFZ+ HMGB2+ HIST1H4C+	PHF19	0	0.365095591	0.48	0.104	0
TUBA1B+ H2AFZ+ HMGB2+ HIST1H4C+	MRPS21	1.90E-162	0.364767478	0.784	0.442	4.04E-158
TUBA1B+ H2AFZ+ HMGB2+ HIST1H4C+	SMC1A	1.08E-217	0.364601085	0.634	0.283	2.30E-213
TUBA1B+ H2AFZ+ HMGB2+ HIST1H4C+	STIP1	3.50E-205	0.364298011	0.691	0.337	7.44E-201
TUBA1B+ H2AFZ+ HMGB2+ HIST1H4C+	EIF4A1	4.37E-110	0.363936683	0.871	0.658	9.30E-106
TUBA1B+ H2AFZ+ HMGB2+ HIST1H4C+	UBE2D2	1.82E-140	0.363680449	0.915	0.693	3.87E-136
TUBA1B+ H2AFZ+ HMGB2+ HIST1H4C+	NAA50	3.41E-202	0.36357354	0.737	0.384	7.24E-198
TUBA1B+ H2AFZ+ HMGB2+ HIST1H4C+	MRPL3	4.55E-150	0.362750978	0.805	0.535	9.67E-146

TUBA1B+ H2AFZ+ HMGB2+ HIST1H4C+	MRPL22	1.03E-170	0.36187591	0.813	0.502	2.19E-166
TUBA1B+ H2AFZ+ HMGB2+ HIST1H4C+	GMPS	1.29E-237	0.361807172	0.63	0.265	2.73E-233
TUBA1B+ H2AFZ+ HMGB2+ HIST1H4C+	ATG3	6.37E-163	0.361742232	0.8	0.496	1.35E-158
TUBA1B+ H2AFZ+ HMGB2+ HIST1H4C+	EI24	5.20E-136	0.361456894	0.911	0.676	1.11E-131
TUBA1B+ H2AFZ+ HMGB2+ HIST1H4C+	MTDH	8.15E-116	0.361300185	0.925	0.744	1.73E-111
TUBA1B+ H2AFZ+ HMGB2+ HIST1H4C+	PFDN2	2.98E-158	0.361021419	0.888	0.629	6.33E-154
TUBA1B+ H2AFZ+ HMGB2+ HIST1H4C+	ARHGAP11A	0	0.360740541	0.36	0.023	0
TUBA1B+ H2AFZ+ HMGB2+ HIST1H4C+	CDCA5	0	0.360710808	0.367	0.006	0
TUBA1B+ H2AFZ+ HMGB2+ HIST1H4C+	TSTA3	2.03E-109	0.360653257	0.886	0.651	4.32E-105
TUBA1B+ H2AFZ+ HMGB2+ HIST1H4C+	SUMO2	5.96E-102	0.360546086	0.972	0.852	1.27E-97
TUBA1B+ H2AFZ+ HMGB2+ HIST1H4C+	NDUFB6	2.81E-138	0.3602872	0.9	0.661	5.99E-134
TUBA1B+ H2AFZ+ HMGB2+ HIST1H4C+	TMEM141	2.44E-93	0.360151071	0.959	0.826	5.19E-89
TUBA1B+ H2AFZ+ HMGB2+ HIST1H4C+	NDUFAF8	8.14E-134	0.360112908	0.804	0.552	1.73E-129
TUBA1B+ H2AFZ+ HMGB2+ HIST1H4C+	UBE2L3	5.38E-130	0.360069159	0.87	0.66	1.14E-125
TUBA1B+ H2AFZ+ HMGB2+ HIST1H4C+	CISD2	7.88E-164	0.359892133	0.797	0.497	1.68E-159
TUBA1B+ H2AFZ+ HMGB2+ HIST1H4C+	COMMD4	4.09E-212	0.359753627	0.67	0.319	8.71E-208
TUBA1B+ H2AFZ+ HMGB2+ HIST1H4C+	SRRM1	1.75E-147	0.359686059	0.91	0.683	3.73E-143
TUBA1B+ H2AFZ+ HMGB2+ HIST1H4C+	SPCS1	2.28E-130	0.359469495	0.959	0.8	4.86E-126
TUBA1B+ H2AFZ+ HMGB2+ HIST1H4C+	MAGOHB	3.69E-252	0.359434959	0.635	0.26	7.86E-248
TUBA1B+ H2AFZ+ HMGB2+ HIST1H4C+	MRPS16	6.57E-159	0.359246788	0.867	0.584	1.40E-154
TUBA1B+ H2AFZ+ HMGB2+ HIST1H4C+	COX7B	9.31E-148	0.358913165	0.994	0.91	1.98E-143
TUBA1B+ H2AFZ+ HMGB2+ HIST1H4C+	SNU13	1.00E-104	0.358652875	0.887	0.695	2.13E-100

TUBA1B+ H2AFZ+ HMGB2+ HIST1H4C+	SAE1	1.60E-241	0.358650261	0.641	0.274	3.40E-237
TUBA1B+ H2AFZ+ HMGB2+ HIST1H4C+	GNL3	3.18E-151	0.35842121	0.792	0.499	6.76E-147
TUBA1B+ H2AFZ+ HMGB2+ HIST1H4C+	MRPL47	9.41E-162	0.358373142	0.78	0.481	2.00E-157
TUBA1B+ H2AFZ+ HMGB2+ HIST1H4C+	CTNNAL1	0	0.358005113	0.48	0.092	0
TUBA1B+ H2AFZ+ HMGB2+ HIST1H4C+	EPB41L2	3.41E-144	0.357630383	0.74	0.44	7.26E-140
TUBA1B+ H2AFZ+ HMGB2+ HIST1H4C+	ZDHHC12	1.54E-134	0.357483246	0.825	0.574	3.28E-130
TUBA1B+ H2AFZ+ HMGB2+ HIST1H4C+	MELK	0	0.357321855	0.374	0.014	0
TUBA1B+ H2AFZ+ HMGB2+ HIST1H4C+	LAPTM4B	1.23E-153	0.35655076	0.736	0.422	2.61E-149
TUBA1B+ H2AFZ+ HMGB2+ HIST1H4C+	CDCA8	0	0.356438555	0.366	0.028	0
TUBA1B+ H2AFZ+ HMGB2+ HIST1H4C+	SMC3	2.99E-168	0.356390682	0.742	0.425	6.36E-164
TUBA1B+ H2AFZ+ HMGB2+ HIST1H4C+	TUBG1	0	0.355944952	0.497	0.136	0
TUBA1B+ H2AFZ+ HMGB2+ HIST1H4C+	SFN	2.66E-171	0.355619116	0.871	0.565	5.65E-167
TUBA1B+ H2AFZ+ HMGB2+ HIST1H4C+	GSTM3	9.03E-231	0.355375469	0.524	0.183	1.92E-226
TUBA1B+ H2AFZ+ HMGB2+ HIST1H4C+	DYNLL1	2.47E-105	0.35528098	0.988	0.893	5.26E-101
TUBA1B+ H2AFZ+ HMGB2+ HIST1H4C+	AP2B1	6.77E-156	0.355183007	0.797	0.493	1.44E-151
TUBA1B+ H2AFZ+ HMGB2+ HIST1H4C+	NUDCD2	6.26E-152	0.354957106	0.754	0.47	1.33E-147
TUBA1B+ H2AFZ+ HMGB2+ HIST1H4C+	NUDT21	3.46E-177	0.354926113	0.783	0.46	7.35E-173
TUBA1B+ H2AFZ+ HMGB2+ HIST1H4C+	HAT1	1.05E-263	0.353700309	0.59	0.219	2.24E-259
TUBA1B+ H2AFZ+ HMGB2+ HIST1H4C+	FKBP4	1.34E-141	0.352866538	0.762	0.487	2.84E-137
TUBA1B+ H2AFZ+ HMGB2+ HIST1H4C+	NDUFV2	1.52E-125	0.352478159	0.925	0.748	3.22E-121
TUBA1B+ H2AFZ+ HMGB2+ HIST1H4C+	HSD17B10	2.17E-140	0.352453457	0.846	0.583	4.62E-136
TUBA1B+ H2AFZ+ HMGB2+ HIST1H4C+	ATP5PO	2.08E-124	0.352381199	0.979	0.832	4.42E-120

TUBA1B+ H2AFZ+ HMGB2+ HIST1H4C+	PSMD2	3.37E-176	0.351385507	0.758	0.433	7.17E-172
TUBA1B+ H2AFZ+ HMGB2+ HIST1H4C+	CDK2AP2	2.16E-127	0.351325993	0.784	0.486	4.58E-123
TUBA1B+ H2AFZ+ HMGB2+ HIST1H4C+	ODC1	9.46E-146	0.3501723	0.813	0.548	2.01E-141
TUBA1B+ H2AFZ+ HMGB2+ HIST1H4C+	NAA10	1.60E-151	0.350124118	0.827	0.544	3.41E-147
TUBA1B+ H2AFZ+ HMGB2+ HIST1H4C+	STRAP	1.15E-118	0.34966796	0.783	0.537	2.46E-114
TUBA1B+ H2AFZ+ HMGB2+ HIST1H4C+	NME4	4.10E-136	0.348799678	0.746	0.447	8.71E-132
TUBA1B+ H2AFZ+ HMGB2+ HIST1H4C+	RFC4	0	0.34752003	0.449	0.078	0
TUBA1B+ H2AFZ+ HMGB2+ HIST1H4C+	RHEB	6.97E-117	0.347338413	0.882	0.639	1.48E-112
TUBA1B+ H2AFZ+ HMGB2+ HIST1H4C+	RBM17	4.09E-159	0.347072559	0.832	0.533	8.69E-155
TUBA1B+ H2AFZ+ HMGB2+ HIST1H4C+	NDUFB3	8.03E-122	0.346922793	0.962	0.794	1.71E-117
TUBA1B+ H2AFZ+ HMGB2+ HIST1H4C+	EZH2	0	0.345857764	0.501	0.136	0
TUBA1B+ H2AFZ+ HMGB2+ HIST1H4C+	DHCR7	1.23E-191	0.345288129	0.53	0.216	2.62E-187
TUBA1B+ H2AFZ+ HMGB2+ HIST1H4C+	MDH2	7.24E-110	0.345224672	0.952	0.778	1.54E-105
TUBA1B+ H2AFZ+ HMGB2+ HIST1H4C+	DNPH1	4.18E-117	0.344906606	0.895	0.67	8.88E-113
TUBA1B+ H2AFZ+ HMGB2+ HIST1H4C+	RAD23A	2.18E-109	0.34431107	0.857	0.659	4.64E-105
TUBA1B+ H2AFZ+ HMGB2+ HIST1H4C+	RPL22L1	1.04E-47	0.34406505	0.785	0.6	2.20E-43
TUBA1B+ H2AFZ+ HMGB2+ HIST1H4C+	POLR2F	3.08E-116	0.34388832	0.834	0.624	6.54E-112
TUBA1B+ H2AFZ+ HMGB2+ HIST1H4C+	HNRNPA0	8.58E-131	0.343800997	0.91	0.682	1.82E-126
TUBA1B+ H2AFZ+ HMGB2+ HIST1H4C+	SDHB	2.76E-140	0.341675883	0.841	0.573	5.86E-136
TUBA1B+ H2AFZ+ HMGB2+ HIST1H4C+	HNRNPU	2.86E-120	0.341369192	0.946	0.792	6.09E-116
TUBA1B+ H2AFZ+ HMGB2+ HIST1H4C+	DMBT1	8.37E-89	0.340379881	0.361	0.164	1.78E-84
TUBA1B+ H2AFZ+ HMGB2+ HIST1H4C+	TFAM	2.10E-181	0.340179064	0.725	0.396	4.47E-177

TUBA1B+ H2AFZ+ HMGB2+ HIST1H4C+	SFPQ	5.85E-98	0.339876259	0.901	0.733	1.24E-93
TUBA1B+ H2AFZ+ HMGB2+ HIST1H4C+	UNG	3.55E-154	0.339843099	0.494	0.224	7.55E-150
TUBA1B+ H2AFZ+ HMGB2+ HIST1H4C+	SGO2	0	0.339521768	0.338	0.041	0
TUBA1B+ H2AFZ+ HMGB2+ HIST1H4C+	COQ2	4.99E-233	0.339239708	0.613	0.255	1.06E-228
TUBA1B+ H2AFZ+ HMGB2+ HIST1H4C+	MRPL4	6.61E-149	0.339110482	0.771	0.477	1.41E-144
TUBA1B+ H2AFZ+ HMGB2+ HIST1H4C+	RFC2	0	0.338579696	0.46	0.11	0
TUBA1B+ H2AFZ+ HMGB2+ HIST1H4C+	MRPL21	3.79E-149	0.338175546	0.826	0.548	8.06E-145
TUBA1B+ H2AFZ+ HMGB2+ HIST1H4C+	NEK2	0	0.337716146	0.316	0.007	0
TUBA1B+ H2AFZ+ HMGB2+ HIST1H4C+	RPS17	1.24E-94	0.33750757	0.996	0.888	2.63E-90
TUBA1B+ H2AFZ+ HMGB2+ HIST1H4C+	CCT3	7.64E-112	0.337356737	0.868	0.634	1.62E-107
TUBA1B+ H2AFZ+ HMGB2+ HIST1H4C+	PYCR1	3.10E-149	0.337172247	0.667	0.369	6.60E-145
TUBA1B+ H2AFZ+ HMGB2+ HIST1H4C+	EIF3K	1.10E-101	0.3370645	0.978	0.827	2.35E-97
TUBA1B+ H2AFZ+ HMGB2+ HIST1H4C+	CCND1	8.67E-61	0.336871438	0.708	0.51	1.84E-56
TUBA1B+ H2AFZ+ HMGB2+ HIST1H4C+	ATP5MPL	1.20E-129	0.336760154	0.986	0.883	2.56E-125
TUBA1B+ H2AFZ+ HMGB2+ HIST1H4C+	ILF3	4.74E-147	0.336737368	0.829	0.549	1.01E-142
TUBA1B+ H2AFZ+ HMGB2+ HIST1H4C+	MVD	7.43E-156	0.33671787	0.595	0.29	1.58E-151
TUBA1B+ H2AFZ+ HMGB2+ HIST1H4C+	RACGAP1	0	0.336435021	0.361	0.037	0
TUBA1B+ H2AFZ+ HMGB2+ HIST1H4C+	FH	3.69E-193	0.336214523	0.7	0.351	7.84E-189
TUBA1B+ H2AFZ+ HMGB2+ HIST1H4C+	ARPC1B	4.04E-91	0.335849056	0.908	0.694	8.59E-87
TUBA1B+ H2AFZ+ HMGB2+ HIST1H4C+	NEDD8	2.41E-116	0.335824144	0.943	0.777	5.12E-112
TUBA1B+ H2AFZ+ HMGB2+ HIST1H4C+	DCUN1D5	5.42E-200	0.33513797	0.619	0.282	1.15E-195
TUBA1B+ H2AFZ+ HMGB2+ HIST1H4C+	RPL26L1	4.71E-165	0.334447925	0.751	0.435	1.00E-160

TUBA1B+ H2AFZ+ HMGB2+ HIST1H4C+	DHCR24	7.02E-203	0.334177728	0.611	0.267	1.49E-198
TUBA1B+ H2AFZ+ HMGB2+ HIST1H4C+	LSM8	3.05E-124	0.333721768	0.882	0.628	6.48E-120
TUBA1B+ H2AFZ+ HMGB2+ HIST1H4C+	OAZ1	1.07E-137	0.333526122	0.99	0.941	2.29E-133
TUBA1B+ H2AFZ+ HMGB2+ HIST1H4C+	RUVBL1	3.24E-201	0.33319765	0.595	0.264	6.90E-197
TUBA1B+ H2AFZ+ HMGB2+ HIST1H4C+	GMDS	1.85E-112	0.333127773	0.913	0.688	3.94E-108
TUBA1B+ H2AFZ+ HMGB2+ HIST1H4C+	REXO2	2.98E-111	0.333084865	0.854	0.601	6.33E-107
TUBA1B+ H2AFZ+ HMGB2+ HIST1H4C+	SNRNP40	2.41E-212	0.332469628	0.655	0.298	5.13E-208
TUBA1B+ H2AFZ+ HMGB2+ HIST1H4C+	MAPRE1	2.54E-160	0.332223745	0.7	0.38	5.40E-156
TUBA1B+ H2AFZ+ HMGB2+ HIST1H4C+	POLR2G	1.42E-149	0.332140056	0.777	0.474	3.02E-145
TUBA1B+ H2AFZ+ HMGB2+ HIST1H4C+	HNRNPH3	1.06E-122	0.33074201	0.821	0.569	2.25E-118
TUBA1B+ H2AFZ+ HMGB2+ HIST1H4C+	TAF9	5.01E-136	0.330466805	0.782	0.515	1.07E-131
TUBA1B+ H2AFZ+ HMGB2+ HIST1H4C+	POLR2K	6.51E-129	0.330409631	0.862	0.622	1.38E-124
TUBA1B+ H2AFZ+ HMGB2+ HIST1H4C+	ATP6V0B	8.71E-117	0.330029402	0.914	0.728	1.85E-112
TUBA1B+ H2AFZ+ HMGB2+ HIST1H4C+	ACTG1	8.86E-78	0.32944297	0.997	0.966	1.88E-73
TUBA1B+ H2AFZ+ HMGB2+ HIST1H4C+	TMEM258	2.72E-127	0.329249772	0.982	0.857	5.79E-123
TUBA1B+ H2AFZ+ HMGB2+ HIST1H4C+	DNAJC8	9.98E-151	0.329225202	0.802	0.501	2.12E-146
TUBA1B+ H2AFZ+ HMGB2+ HIST1H4C+	NDUFB8	9.76E-114	0.328685518	0.926	0.75	2.08E-109
TUBA1B+ H2AFZ+ HMGB2+ HIST1H4C+	VDAC3	1.20E-121	0.328497052	0.821	0.553	2.56E-117
TUBA1B+ H2AFZ+ HMGB2+ HIST1H4C+	PPP1CA	4.39E-116	0.328308008	0.927	0.758	9.33E-112
TUBA1B+ H2AFZ+ HMGB2+ HIST1H4C+	DDX18	2.49E-134	0.328106431	0.868	0.609	5.30E-130
TUBA1B+ H2AFZ+ HMGB2+ HIST1H4C+	KIF4A	0	0.328065446	0.332	0.009	0
TUBA1B+ H2AFZ+ HMGB2+ HIST1H4C+	GOT2	2.17E-194	0.328006399	0.665	0.323	4.62E-190

TUBA1B+ H2AFZ+ HMGB2+ HIST1H4C+	KHDRBS1	1.58E-124	0.327996653	0.9	0.691	3.36E-120
TUBA1B+ H2AFZ+ HMGB2+ HIST1H4C+	TCOF1	8.18E-286	0.32775661	0.532	0.165	1.74E-281
TUBA1B+ H2AFZ+ HMGB2+ HIST1H4C+	FKBP3	1.48E-110	0.32689151	0.801	0.563	3.14E-106
TUBA1B+ H2AFZ+ HMGB2+ HIST1H4C+	CXCL2	4.28E-57	0.325151809	0.438	0.256	9.11E-53
TUBA1B+ H2AFZ+ HMGB2+ HIST1H4C+	NDUFA10	4.10E-120	0.325144808	0.891	0.641	8.72E-116
TUBA1B+ H2AFZ+ HMGB2+ HIST1H4C+	TMEM160	3.25E-148	0.324754561	0.807	0.51	6.92E-144
TUBA1B+ H2AFZ+ HMGB2+ HIST1H4C+	RPL8	1.42E-58	0.324118064	1	0.969	3.02E-54
TUBA1B+ H2AFZ+ HMGB2+ HIST1H4C+	SLC52A2	2.73E-111	0.324043525	0.709	0.445	5.81E-107
TUBA1B+ H2AFZ+ HMGB2+ HIST1H4C+	TRA2B	1.34E-137	0.323898028	0.837	0.577	2.86E-133
TUBA1B+ H2AFZ+ HMGB2+ HIST1H4C+	LAGE3	5.08E-153	0.323680516	0.731	0.417	1.08E-148
TUBA1B+ H2AFZ+ HMGB2+ HIST1H4C+	NDUFAF2	2.70E-153	0.323562035	0.765	0.446	5.74E-149
TUBA1B+ H2AFZ+ HMGB2+ HIST1H4C+	KIFC1	0	0.323538071	0.317	0.007	0
TUBA1B+ H2AFZ+ HMGB2+ HIST1H4C+	WDR1	1.27E-106	0.323377489	0.824	0.607	2.70E-102
TUBA1B+ H2AFZ+ HMGB2+ HIST1H4C+	MCM6	0	0.32184655	0.388	0.054	0
TUBA1B+ H2AFZ+ HMGB2+ HIST1H4C+	APRT	4.93E-92	0.321750423	0.952	0.779	1.05E-87
TUBA1B+ H2AFZ+ HMGB2+ HIST1H4C+	PHB2	1.51E-105	0.321175819	0.817	0.589	3.22E-101
TUBA1B+ H2AFZ+ HMGB2+ HIST1H4C+	PSMB6	2.18E-100	0.321048506	0.889	0.68	4.63E-96
TUBA1B+ H2AFZ+ HMGB2+ HIST1H4C+	CAMTA1	2.06E-123	0.321025867	0.926	0.703	4.38E-119
TUBA1B+ H2AFZ+ HMGB2+ HIST1H4C+	ETFB	1.29E-97	0.321004463	0.903	0.719	2.75E-93
TUBA1B+ H2AFZ+ HMGB2+ HIST1H4C+	HMGN3	5.97E-119	0.32098629	0.834	0.564	1.27E-114
TUBA1B+ H2AFZ+ HMGB2+ HIST1H4C+	MRTO4	1.11E-204	0.320727019	0.62	0.278	2.35E-200
TUBA1B+ H2AFZ+ HMGB2+ HIST1H4C+	CLDN2	4.43E-103	0.320520525	0.514	0.268	9.42E-99

TUBA1B+ H2AFZ+ HMGB2+ HIST1H4C+	MAZ	9.63E-138	0.31991722	0.762	0.471	2.05E-133
TUBA1B+ H2AFZ+ HMGB2+ HIST1H4C+	LYPLA1	7.46E-94	0.319706828	0.825	0.589	1.59E-89
TUBA1B+ H2AFZ+ HMGB2+ HIST1H4C+	CNBP	7.99E-102	0.319023774	0.939	0.768	1.70E-97
TUBA1B+ H2AFZ+ HMGB2+ HIST1H4C+	C19orf48	9.64E-161	0.317977649	0.662	0.339	2.05E-156
TUBA1B+ H2AFZ+ HMGB2+ HIST1H4C+	MORF4L2	3.64E-108	0.317769986	0.86	0.642	7.74E-104
TUBA1B+ H2AFZ+ HMGB2+ HIST1H4C+	BRCA2	0	0.317557655	0.324	0.025	0
TUBA1B+ H2AFZ+ HMGB2+ HIST1H4C+	TMED9	4.00E-97	0.317147919	0.912	0.74	8.51E-93
TUBA1B+ H2AFZ+ HMGB2+ HIST1H4C+	COPRS	2.18E-173	0.316930593	0.652	0.334	4.65E-169
TUBA1B+ H2AFZ+ HMGB2+ HIST1H4C+	PTMS	4.17E-57	0.314713255	0.826	0.621	8.87E-53
TUBA1B+ H2AFZ+ HMGB2+ HIST1H4C+	SNRPA	1.28E-200	0.31451629	0.672	0.317	2.73E-196
TUBA1B+ H2AFZ+ HMGB2+ HIST1H4C+	NPM3	6.95E-135	0.314492913	0.653	0.367	1.48E-130
TUBA1B+ H2AFZ+ HMGB2+ HIST1H4C+	FAM136A	3.43E-150	0.314418085	0.765	0.466	7.29E-146
TUBA1B+ H2AFZ+ HMGB2+ HIST1H4C+	PXMP2	1.80E-138	0.314405092	0.771	0.447	3.83E-134
TUBA1B+ H2AFZ+ HMGB2+ HIST1H4C+	ATAD5	0	0.314293824	0.372	0.043	0
TUBA1B+ H2AFZ+ HMGB2+ HIST1H4C+	RBM8A	6.12E-117	0.31388775	0.897	0.662	1.30E-112
TUBA1B+ H2AFZ+ HMGB2+ HIST1H4C+	PRPS2	1.05E-174	0.313596111	0.652	0.321	2.24E-170
TUBA1B+ H2AFZ+ HMGB2+ HIST1H4C+	SF3B6	4.74E-106	0.313567913	0.949	0.793	1.01E-101
TUBA1B+ H2AFZ+ HMGB2+ HIST1H4C+	MRPS25	2.71E-137	0.313282583	0.771	0.481	5.76E-133
TUBA1B+ H2AFZ+ HMGB2+ HIST1H4C+	NAP1L4	3.50E-153	0.312846376	0.728	0.413	7.45E-149
TUBA1B+ H2AFZ+ HMGB2+ HIST1H4C+	PKMYT1	0	0.312839997	0.333	0.01	0
TUBA1B+ H2AFZ+ HMGB2+ HIST1H4C+	BRIX1	5.10E-179	0.312820888	0.633	0.307	1.08E-174
TUBA1B+ H2AFZ+ HMGB2+ HIST1H4C+	NDUFS3	1.21E-116	0.312636675	0.872	0.631	2.57E-112

TUBA1B+ H2AFZ+ HMGB2+ HIST1H4C+	SMIM37	1.34E-137	0.312476186	0.802	0.503	2.84E-133
TUBA1B+ H2AFZ+ HMGB2+ HIST1H4C+	RPL27	3.43E-90	0.312397763	0.997	0.918	7.28E-86
TUBA1B+ H2AFZ+ HMGB2+ HIST1H4C+	PSMB7	2.23E-111	0.312112878	0.896	0.673	4.74E-107
TUBA1B+ H2AFZ+ HMGB2+ HIST1H4C+	TACSTD2	1.03E-76	0.311993014	0.383	0.189	2.20E-72
TUBA1B+ H2AFZ+ HMGB2+ HIST1H4C+	RNPS1	8.46E-124	0.311906308	0.874	0.618	1.80E-119
TUBA1B+ H2AFZ+ HMGB2+ HIST1H4C+	RPLP1	3.28E-89	0.311283159	1	0.983	6.98E-85
TUBA1B+ H2AFZ+ HMGB2+ HIST1H4C+	ACTL6A	2.23E-182	0.311255271	0.661	0.318	4.75E-178
TUBA1B+ H2AFZ+ HMGB2+ HIST1H4C+	JPT2	6.46E-199	0.310988491	0.65	0.297	1.37E-194
TUBA1B+ H2AFZ+ HMGB2+ HIST1H4C+	NUP37	2.08E-202	0.310981824	0.608	0.268	4.43E-198
TUBA1B+ H2AFZ+ HMGB2+ HIST1H4C+	PPP1R14B	2.63E-107	0.310666878	0.878	0.639	5.58E-103
TUBA1B+ H2AFZ+ HMGB2+ HIST1H4C+	PDAP1	6.30E-114	0.310360392	0.854	0.604	1.34E-109
TUBA1B+ H2AFZ+ HMGB2+ HIST1H4C+	IFRD2	3.19E-157	0.309344906	0.667	0.359	6.79E-153
TUBA1B+ H2AFZ+ HMGB2+ HIST1H4C+	EIF4A3	8.35E-131	0.309063628	0.655	0.383	1.78E-126
TUBA1B+ H2AFZ+ HMGB2+ HIST1H4C+	HDAC1	7.26E-116	0.308273595	0.872	0.639	1.54E-111
TUBA1B+ H2AFZ+ HMGB2+ HIST1H4C+	ALYREF	2.95E-227	0.307881885	0.536	0.204	6.27E-223
TUBA1B+ H2AFZ+ HMGB2+ HIST1H4C+	GAR1	1.12E-158	0.307707322	0.686	0.372	2.38E-154
TUBA1B+ H2AFZ+ HMGB2+ HIST1H4C+	CHAF1A	0	0.307474826	0.41	0.076	0
TUBA1B+ H2AFZ+ HMGB2+ HIST1H4C+	MRPS18C	4.46E-127	0.30740754	0.812	0.529	9.49E-123
TUBA1B+ H2AFZ+ HMGB2+ HIST1H4C+	EIF3J	2.92E-110	0.307355658	0.85	0.626	6.21E-106
TUBA1B+ H2AFZ+ HMGB2+ HIST1H4C+	PSMB9	1.31E-76	0.307071998	0.739	0.515	2.79E-72
TUBA1B+ H2AFZ+ HMGB2+ HIST1H4C+	BIN1	1.07E-118	0.307054163	0.671	0.391	2.27E-114
TUBA1B+ H2AFZ+ HMGB2+ HIST1H4C+	ATP5PD	2.50E-93	0.306910631	0.954	0.795	5.32E-89

TUBA1B+ H2AFZ+ HMGB2+ HIST1H4C+	BOP1	4.97E-164	0.30653311	0.536	0.245	1.06E-159
TUBA1B+ H2AFZ+ HMGB2+ HIST1H4C+	THOC3	5.99E-228	0.306342176	0.592	0.238	1.27E-223
TUBA1B+ H2AFZ+ HMGB2+ HIST1H4C+	NDUFB11	9.91E-105	0.306237663	0.959	0.815	2.11E-100
TUBA1B+ H2AFZ+ HMGB2+ HIST1H4C+	MRPL15	1.38E-111	0.306221655	0.76	0.5	2.93E-107
TUBA1B+ H2AFZ+ HMGB2+ HIST1H4C+	UBA2	4.10E-148	0.306049443	0.687	0.375	8.72E-144
TUBA1B+ H2AFZ+ HMGB2+ HIST1H4C+	NDUFB2	8.25E-94	0.305769635	0.983	0.849	1.75E-89
TUBA1B+ H2AFZ+ HMGB2+ HIST1H4C+	PRPF19	9.32E-176	0.303470435	0.694	0.358	1.98E-171
TUBA1B+ H2AFZ+ HMGB2+ HIST1H4C+	EIF4E	1.44E-123	0.303375161	0.791	0.515	3.05E-119
TUBA1B+ H2AFZ+ HMGB2+ HIST1H4C+	BZW1	3.38E-104	0.303042817	0.842	0.61	7.19E-100
TUBA1B+ H2AFZ+ HMGB2+ HIST1H4C+	PCMT1	3.43E-142	0.302670167	0.782	0.487	7.30E-138
TUBA1B+ H2AFZ+ HMGB2+ HIST1H4C+	HP1BP3	9.39E-93	0.302649118	0.799	0.554	2.00E-88
TUBA1B+ H2AFZ+ HMGB2+ HIST1H4C+	HSPB11	1.68E-139	0.302479554	0.661	0.355	3.58E-135
TUBA1B+ H2AFZ+ HMGB2+ HIST1H4C+	POLR2I	2.43E-109	0.302350676	0.884	0.671	5.17E-105
TUBA1B+ H2AFZ+ HMGB2+ HIST1H4C+	ATAD3A	7.48E-281	0.302192042	0.527	0.165	1.59E-276
TUBA1B+ H2AFZ+ HMGB2+ HIST1H4C+	CDC37	2.99E-109	0.302109428	0.876	0.663	6.37E-105
TUBA1B+ H2AFZ+ HMGB2+ HIST1H4C+	MRPS35	2.19E-110	0.301959112	0.802	0.555	4.66E-106
TUBA1B+ H2AFZ+ HMGB2+ HIST1H4C+	TMEM14B	4.70E-103	0.30195842	0.911	0.68	9.99E-99
TUBA1B+ H2AFZ+ HMGB2+ HIST1H4C+	F12	9.82E-196	0.301599013	0.577	0.246	2.09E-191
TUBA1B+ H2AFZ+ HMGB2+ HIST1H4C+	DRAP1	2.46E-100	0.301314165	0.878	0.636	5.22E-96
TUBA1B+ H2AFZ+ HMGB2+ HIST1H4C+	TAF15	3.73E-152	0.300615302	0.681	0.376	7.93E-148
TUBA1B+ H2AFZ+ HMGB2+ HIST1H4C+	KNSTRN	0	0.300546231	0.311	0.031	0
TUBA1B+ H2AFZ+ HMGB2+ HIST1H4C+	MRPL19	1.72E-135	0.300445442	0.73	0.434	3.66E-131

TUBA1B+ H2AFZ+ HMGB2+ HIST1H4C+	SIGMAR1	8.63E-203	0.300378504	0.595	0.252	1.84E-198
TUBA1B+ H2AFZ+ HMGB2+ HIST1H4C+	NDUFAF3	8.73E-111	0.30036872	0.85	0.579	1.86E-106
TUBA1B+ H2AFZ+ HMGB2+ HIST1H4C+	UHRF1	0	0.300117139	0.313	0.015	0
TUBA1B+ H2AFZ+ HMGB2+ HIST1H4C+	RNASEH2C	6.15E-145	0.300064384	0.696	0.389	1.31E-140
TUBA1B+ H2AFZ+ HMGB2+ HIST1H4C+	ANXA4	2.59E-55	0.299974857	0.9	0.739	5.50E-51
TUBA1B+ H2AFZ+ HMGB2+ HIST1H4C+	SRSF10	1.17E-125	0.299486917	0.84	0.582	2.49E-121
TUBA1B+ H2AFZ+ HMGB2+ HIST1H4C+	NUPR1	7.12E-143	0.299040939	0.866	0.545	1.51E-138
TUBA1B+ H2AFZ+ HMGB2+ HIST1H4C+	ANXA3	2.34E-92	0.298553444	0.748	0.49	4.99E-88
TUBA1B+ H2AFZ+ HMGB2+ HIST1H4C+	ELAVL1	2.04E-150	0.298524725	0.725	0.417	4.34E-146
TUBA1B+ H2AFZ+ HMGB2+ HIST1H4C+	PIH1D1	8.83E-144	0.29815305	0.764	0.468	1.88E-139
TUBA1B+ H2AFZ+ HMGB2+ HIST1H4C+	KNL1	0	0.297985147	0.31	0.015	0
TUBA1B+ H2AFZ+ HMGB2+ HIST1H4C+	RCC1	3.24E-260	0.297515707	0.528	0.174	6.89E-256
TUBA1B+ H2AFZ+ HMGB2+ HIST1H4C+	IMPA2	1.92E-130	0.296893363	0.727	0.433	4.09E-126
TUBA1B+ H2AFZ+ HMGB2+ HIST1H4C+	FOXM1	0	0.296208279	0.35	0.016	0
TUBA1B+ H2AFZ+ HMGB2+ HIST1H4C+	RPL4	1.41E-66	0.29612422	0.993	0.872	2.99E-62
TUBA1B+ H2AFZ+ HMGB2+ HIST1H4C+	COX7A2	3.39E-131	0.296123861	0.994	0.942	7.21E-127
TUBA1B+ H2AFZ+ HMGB2+ HIST1H4C+	TXNDC17	6.42E-105	0.295971065	0.949	0.777	1.37E-100
TUBA1B+ H2AFZ+ HMGB2+ HIST1H4C+	ARPC2	1.81E-83	0.295794179	0.972	0.869	3.85E-79
TUBA1B+ H2AFZ+ HMGB2+ HIST1H4C+	FANCI	0	0.295678446	0.348	0.023	0
TUBA1B+ H2AFZ+ HMGB2+ HIST1H4C+	IMPDH2	2.21E-86	0.295588062	0.876	0.627	4.69E-82
TUBA1B+ H2AFZ+ HMGB2+ HIST1H4C+	RCN2	1.32E-109	0.295475047	0.787	0.53	2.81E-105
TUBA1B+ H2AFZ+ HMGB2+ HIST1H4C+	PSMD13	3.10E-123	0.295474199	0.776	0.494	6.59E-119

TUBA1B+ H2AFZ+ HMGB2+ HIST1H4C+	NDUFA9	1.76E-98	0.295242272	0.8	0.554	3.73E-94
TUBA1B+ H2AFZ+ HMGB2+ HIST1H4C+	RPA2	3.77E-214	0.295192797	0.543	0.207	8.01E-210
TUBA1B+ H2AFZ+ HMGB2+ HIST1H4C+	MRPS23	2.06E-126	0.295099466	0.649	0.386	4.38E-122
TUBA1B+ H2AFZ+ HMGB2+ HIST1H4C+	ITGB1BP1	2.08E-159	0.294695927	0.666	0.339	4.43E-155
TUBA1B+ H2AFZ+ HMGB2+ HIST1H4C+	PSMC5	7.73E-102	0.293730623	0.786	0.553	1.64E-97
TUBA1B+ H2AFZ+ HMGB2+ HIST1H4C+	SUMO3	1.62E-120	0.29354372	0.81	0.537	3.45E-116
TUBA1B+ H2AFZ+ HMGB2+ HIST1H4C+	PFDN6	1.33E-117	0.29354002	0.801	0.525	2.82E-113
TUBA1B+ H2AFZ+ HMGB2+ HIST1H4C+	ANAPC15	1.42E-153	0.293182652	0.72	0.394	3.02E-149
TUBA1B+ H2AFZ+ HMGB2+ HIST1H4C+	MRPS2	2.56E-156	0.293006346	0.658	0.34	5.45E-152
TUBA1B+ H2AFZ+ HMGB2+ HIST1H4C+	COX6C	5.80E-91	0.293004381	0.992	0.931	1.23E-86
TUBA1B+ H2AFZ+ HMGB2+ HIST1H4C+	BZW2	3.55E-116	0.292895414	0.775	0.491	7.54E-112
TUBA1B+ H2AFZ+ HMGB2+ HIST1H4C+	NCAPD3	0	0.292624263	0.393	0.059	0
TUBA1B+ H2AFZ+ HMGB2+ HIST1H4C+	SRP72	2.99E-113	0.292290766	0.766	0.509	6.35E-109
TUBA1B+ H2AFZ+ HMGB2+ HIST1H4C+	VBP1	8.19E-134	0.29221108	0.73	0.428	1.74E-129
TUBA1B+ H2AFZ+ HMGB2+ HIST1H4C+	PPP2R1A	5.35E-93	0.292002158	0.867	0.646	1.14E-88
TUBA1B+ H2AFZ+ HMGB2+ HIST1H4C+	GHITM	9.22E-104	0.291522962	0.937	0.756	1.96E-99
TUBA1B+ H2AFZ+ HMGB2+ HIST1H4C+	MRPL50	5.21E-159	0.291416765	0.715	0.393	1.11E-154
TUBA1B+ H2AFZ+ HMGB2+ HIST1H4C+	PPP4C	7.31E-105	0.291305005	0.899	0.702	1.55E-100
TUBA1B+ H2AFZ+ HMGB2+ HIST1H4C+	PGD	5.82E-158	0.290983948	0.656	0.334	1.24E-153
TUBA1B+ H2AFZ+ HMGB2+ HIST1H4C+	THRAP3	4.36E-107	0.290345189	0.799	0.554	9.28E-103
TUBA1B+ H2AFZ+ HMGB2+ HIST1H4C+	RER1	3.03E-112	0.28988844	0.886	0.659	6.45E-108
TUBA1B+ H2AFZ+ HMGB2+ HIST1H4C+	TMEM70	5.64E-144	0.289858902	0.643	0.347	1.20E-139

TUBA1B+ H2AFZ+ HMGB2+ HIST1H4C+	CLPP	2.62E-144	0.289808908	0.717	0.415	5.58E-140
TUBA1B+ H2AFZ+ HMGB2+ HIST1H4C+	BID	8.07E-101	0.289628293	0.694	0.432	1.72E-96
TUBA1B+ H2AFZ+ HMGB2+ HIST1H4C+	MESD	6.65E-121	0.289231854	0.766	0.492	1.41E-116
TUBA1B+ H2AFZ+ HMGB2+ HIST1H4C+	SNRPB2	2.22E-112	0.289056719	0.884	0.648	4.72E-108
TUBA1B+ H2AFZ+ HMGB2+ HIST1H4C+	PARPBP	0	0.288958773	0.374	0.036	0
TUBA1B+ H2AFZ+ HMGB2+ HIST1H4C+	U2SURP	3.74E-125	0.28851466	0.822	0.547	7.96E-121
TUBA1B+ H2AFZ+ HMGB2+ HIST1H4C+	CDC6	0	0.288451334	0.324	0.022	0
TUBA1B+ H2AFZ+ HMGB2+ HIST1H4C+	LAP3	2.12E-125	0.288435006	0.648	0.364	4.52E-121
TUBA1B+ H2AFZ+ HMGB2+ HIST1H4C+	TMX2	1.32E-123	0.288078404	0.74	0.457	2.81E-119
TUBA1B+ H2AFZ+ HMGB2+ HIST1H4C+	RPL22	6.00E-81	0.287972472	0.997	0.893	1.27E-76
TUBA1B+ H2AFZ+ HMGB2+ HIST1H4C+	NSA2	2.88E-89	0.287947358	0.909	0.698	6.12E-85
TUBA1B+ H2AFZ+ HMGB2+ HIST1H4C+	SHMT2	1.09E-173	0.28793785	0.623	0.289	2.32E-169
TUBA1B+ H2AFZ+ HMGB2+ HIST1H4C+	NDC1	5.83E-301	0.287780519	0.442	0.109	1.24E-296
TUBA1B+ H2AFZ+ HMGB2+ HIST1H4C+	BARD1	7.34E-288	0.287747031	0.426	0.104	1.56E-283
TUBA1B+ H2AFZ+ HMGB2+ HIST1H4C+	E2F1	0	0.2874366	0.322	0.024	0
TUBA1B+ H2AFZ+ HMGB2+ HIST1H4C+	GNAS	3.76E-91	0.286903626	0.951	0.834	8.00E-87
TUBA1B+ H2AFZ+ HMGB2+ HIST1H4C+	IMMT	2.20E-144	0.286724682	0.717	0.398	4.68E-140
TUBA1B+ H2AFZ+ HMGB2+ HIST1H4C+	FAM96B	1.67E-108	0.28612171	0.917	0.692	3.56E-104
TUBA1B+ H2AFZ+ HMGB2+ HIST1H4C+	LACTB2	4.49E-142	0.286085602	0.639	0.332	9.54E-138
TUBA1B+ H2AFZ+ HMGB2+ HIST1H4C+	SNRNP70	1.30E-113	0.285929291	0.806	0.553	2.76E-109
TUBA1B+ H2AFZ+ HMGB2+ HIST1H4C+	DBF4	4.15E-214	0.285299829	0.493	0.177	8.82E-210
TUBA1B+ H2AFZ+ HMGB2+ HIST1H4C+	TXNDC12	2.23E-117	0.285126833	0.762	0.49	4.74E-113

TUBA1B+ H2AFZ+ HMGB2+ HIST1H4C+	MRPL52	1.32E-98	0.284201263	0.882	0.696	2.80E-94
TUBA1B+ H2AFZ+ HMGB2+ HIST1H4C+	INSIG1	5.78E-137	0.284063267	0.633	0.32	1.23E-132
TUBA1B+ H2AFZ+ HMGB2+ HIST1H4C+	NSD2	4.63E-213	0.284026396	0.55	0.212	9.85E-209
TUBA1B+ H2AFZ+ HMGB2+ HIST1H4C+	SMARCC1	3.69E-104	0.283816349	0.768	0.496	7.84E-100
TUBA1B+ H2AFZ+ HMGB2+ HIST1H4C+	NOL7	1.10E-98	0.283532441	0.832	0.59	2.33E-94
TUBA1B+ H2AFZ+ HMGB2+ HIST1H4C+	FASN	2.29E-175	0.282748771	0.542	0.233	4.87E-171
TUBA1B+ H2AFZ+ HMGB2+ HIST1H4C+	MTHFD1	9.86E-215	0.282720988	0.534	0.2	2.10E-210
TUBA1B+ H2AFZ+ HMGB2+ HIST1H4C+	PIN1	6.21E-131	0.28266201	0.783	0.493	1.32E-126
TUBA1B+ H2AFZ+ HMGB2+ HIST1H4C+	POC1A	0	0.282531009	0.362	0.021	0
TUBA1B+ H2AFZ+ HMGB2+ HIST1H4C+	POP7	8.65E-136	0.282432156	0.736	0.428	1.84E-131
TUBA1B+ H2AFZ+ HMGB2+ HIST1H4C+	C8orf59	3.52E-84	0.282243966	0.911	0.696	7.48E-80
TUBA1B+ H2AFZ+ HMGB2+ HIST1H4C+	LY6E	3.34E-32	0.281697105	0.316	0.202	7.10E-28
TUBA1B+ H2AFZ+ HMGB2+ HIST1H4C+	ANXA2	3.21E-76	0.281548666	0.975	0.885	6.82E-72
TUBA1B+ H2AFZ+ HMGB2+ HIST1H4C+	COX7C	3.47E-90	0.281397158	0.996	0.93	7.37E-86
TUBA1B+ H2AFZ+ HMGB2+ HIST1H4C+	UCHL3	2.45E-117	0.281318239	0.791	0.504	5.22E-113
TUBA1B+ H2AFZ+ HMGB2+ HIST1H4C+	HAUS1	3.15E-165	0.280500922	0.513	0.219	6.69E-161
TUBA1B+ H2AFZ+ HMGB2+ HIST1H4C+	NDUFA2	2.78E-95	0.279824566	0.941	0.777	5.91E-91
TUBA1B+ H2AFZ+ HMGB2+ HIST1H4C+	AKR7A2	1.39E-94	0.279760608	0.773	0.521	2.96E-90
TUBA1B+ H2AFZ+ HMGB2+ HIST1H4C+	RALY	6.22E-92	0.27975516	0.887	0.701	1.32E-87
TUBA1B+ H2AFZ+ HMGB2+ HIST1H4C+	NIFK	1.84E-113	0.27965414	0.713	0.427	3.91E-109
TUBA1B+ H2AFZ+ HMGB2+ HIST1H4C+	NAE1	2.70E-141	0.279232654	0.725	0.422	5.75E-137
TUBA1B+ H2AFZ+ HMGB2+ HIST1H4C+	RBMX	6.16E-80	0.279066124	0.867	0.641	1.31E-75

TUBA1B+ H2AFZ+ HMGB2+ HIST1H4C+	LIG1	0	0.278603725	0.403	0.07	0
TUBA1B+ H2AFZ+ HMGB2+ HIST1H4C+	KIF23	0	0.278353781	0.265	0.006	0
TUBA1B+ H2AFZ+ HMGB2+ HIST1H4C+	FUS	1.45E-95	0.27821947	0.915	0.727	3.09E-91
TUBA1B+ H2AFZ+ HMGB2+ HIST1H4C+	CENPS	0	0.278062364	0.443	0.104	0
TUBA1B+ H2AFZ+ HMGB2+ HIST1H4C+	SHMT1	1.87E-276	0.277791804	0.461	0.127	3.97E-272
TUBA1B+ H2AFZ+ HMGB2+ HIST1H4C+	COMT	2.84E-91	0.277773123	0.802	0.567	6.05E-87
TUBA1B+ H2AFZ+ HMGB2+ HIST1H4C+	DEPDC1	0	0.277631399	0.252	0.004	0
TUBA1B+ H2AFZ+ HMGB2+ HIST1H4C+	PTGES2	3.38E-108	0.276805173	0.782	0.509	7.18E-104
TUBA1B+ H2AFZ+ HMGB2+ HIST1H4C+	CDCA4	2.78E-284	0.276796173	0.377	0.083	5.91E-280
TUBA1B+ H2AFZ+ HMGB2+ HIST1H4C+	G3BP1	1.50E-122	0.276793293	0.784	0.496	3.20E-118
TUBA1B+ H2AFZ+ HMGB2+ HIST1H4C+	CSTF3	6.91E-198	0.276241124	0.588	0.244	1.47E-193
TUBA1B+ H2AFZ+ HMGB2+ HIST1H4C+	OXCT1	0	0.275599545	0.425	0.08	0
TUBA1B+ H2AFZ+ HMGB2+ HIST1H4C+	MYDGF	2.86E-88	0.275589305	0.903	0.714	6.08E-84
TUBA1B+ H2AFZ+ HMGB2+ HIST1H4C+	FAM96A	4.76E-113	0.274764917	0.787	0.516	1.01E-108
TUBA1B+ H2AFZ+ HMGB2+ HIST1H4C+	MTCH1	1.72E-98	0.2745919	0.848	0.619	3.67E-94
TUBA1B+ H2AFZ+ HMGB2+ HIST1H4C+	PLEK2	3.27E-155	0.274443325	0.673	0.356	6.95E-151
TUBA1B+ H2AFZ+ HMGB2+ HIST1H4C+	SLC38A5	5.91E-229	0.274253338	0.471	0.154	1.26E-224
TUBA1B+ H2AFZ+ HMGB2+ HIST1H4C+	ANLN	0	0.274194219	0.289	0.008	0
TUBA1B+ H2AFZ+ HMGB2+ HIST1H4C+	CTSH	1.26E-118	0.274151253	0.643	0.365	2.68E-114
TUBA1B+ H2AFZ+ HMGB2+ HIST1H4C+	YWHAE	2.58E-68	0.273858654	0.967	0.841	5.49E-64
TUBA1B+ H2AFZ+ HMGB2+ HIST1H4C+	CCDC124	1.89E-118	0.273743416	0.705	0.427	4.01E-114
TUBA1B+ H2AFZ+ HMGB2+ HIST1H4C+	GNPNAT1	4.70E-151	0.272336168	0.592	0.298	9.99E-147

TUBA1B+ H2AFZ+ HMGB2+ HIST1H4C+	BTG3	4.61E-166	0.272287536	0.586	0.267	9.81E-162
TUBA1B+ H2AFZ+ HMGB2+ HIST1H4C+	SRSF1	5.67E-126	0.27206304	0.771	0.485	1.21E-121
TUBA1B+ H2AFZ+ HMGB2+ HIST1H4C+	CANX	5.97E-77	0.272036635	0.933	0.755	1.27E-72
TUBA1B+ H2AFZ+ HMGB2+ HIST1H4C+	SPC24	0	0.271827679	0.293	0.008	0
TUBA1B+ H2AFZ+ HMGB2+ HIST1H4C+	AP2M1	1.61E-79	0.271666519	0.865	0.657	3.43E-75
TUBA1B+ H2AFZ+ HMGB2+ HIST1H4C+	RPS20	7.73E-70	0.271611175	0.998	0.936	1.64E-65
TUBA1B+ H2AFZ+ HMGB2+ HIST1H4C+	DENR	7.15E-115	0.27142729	0.738	0.457	1.52E-110
TUBA1B+ H2AFZ+ HMGB2+ HIST1H4C+	GOT1	8.13E-151	0.27103553	0.61	0.301	1.73E-146
TUBA1B+ H2AFZ+ HMGB2+ HIST1H4C+	BRK1	2.24E-89	0.270898289	0.943	0.772	4.77E-85
TUBA1B+ H2AFZ+ HMGB2+ HIST1H4C+	SPCS2	1.38E-76	0.270695911	0.916	0.752	2.94E-72
TUBA1B+ H2AFZ+ HMGB2+ HIST1H4C+	SERPINH1	8.62E-141	0.270652337	0.614	0.309	1.83E-136
TUBA1B+ H2AFZ+ HMGB2+ HIST1H4C+	PRPF40A	1.09E-100	0.270193114	0.839	0.595	2.31E-96
TUBA1B+ H2AFZ+ HMGB2+ HIST1H4C+	MCUB	4.99E-265	0.270099888	0.4	0.097	1.06E-260
TUBA1B+ H2AFZ+ HMGB2+ HIST1H4C+	DCXR	3.57E-97	0.269763954	0.815	0.574	7.59E-93
TUBA1B+ H2AFZ+ HMGB2+ HIST1H4C+	TOP1	7.94E-86	0.269452245	0.812	0.582	1.69E-81
TUBA1B+ H2AFZ+ HMGB2+ HIST1H4C+	DIAPH3	0	0.269098039	0.311	0.01	0
TUBA1B+ H2AFZ+ HMGB2+ HIST1H4C+	ESCO2	0	0.268996886	0.299	0.01	0
TUBA1B+ H2AFZ+ HMGB2+ HIST1H4C+	BCAP31	2.79E-90	0.268947071	0.903	0.719	5.94E-86
TUBA1B+ H2AFZ+ HMGB2+ HIST1H4C+	ZNF511	9.92E-103	0.267980923	0.792	0.532	2.11E-98
TUBA1B+ H2AFZ+ HMGB2+ HIST1H4C+	GDI2	8.72E-80	0.267644952	0.905	0.711	1.86E-75
TUBA1B+ H2AFZ+ HMGB2+ HIST1H4C+	PSMC4	8.44E-110	0.267602412	0.725	0.457	1.79E-105
TUBA1B+ H2AFZ+ HMGB2+ HIST1H4C+	HSBP1	1.19E-92	0.267311156	0.961	0.8	2.53E-88

TUBA1B+ H2AFZ+ HMGB2+ HIST1H4C+	HSD17B12	4.99E-70	0.267303252	0.828	0.623	1.06E-65
TUBA1B+ H2AFZ+ HMGB2+ HIST1H4C+	SERPINB6	1.38E-51	0.267212344	0.903	0.733	2.94E-47
TUBA1B+ H2AFZ+ HMGB2+ HIST1H4C+	HSPH1	2.95E-122	0.267047883	0.736	0.438	6.28E-118
TUBA1B+ H2AFZ+ HMGB2+ HIST1H4C+	CTSC	2.22E-155	0.266779032	0.69	0.342	4.72E-151
TUBA1B+ H2AFZ+ HMGB2+ HIST1H4C+	PSAT1	1.35E-221	0.266767405	0.322	0.073	2.88E-217
TUBA1B+ H2AFZ+ HMGB2+ HIST1H4C+	HPF1	4.50E-177	0.266254203	0.544	0.223	9.57E-173
TUBA1B+ H2AFZ+ HMGB2+ HIST1H4C+	MSMO1	4.05E-110	0.266193928	0.564	0.294	8.61E-106
TUBA1B+ H2AFZ+ HMGB2+ HIST1H4C+	POLR2H	7.80E-105	0.265994461	0.796	0.536	1.66E-100
TUBA1B+ H2AFZ+ HMGB2+ HIST1H4C+	MRPS7	1.54E-103	0.265660025	0.78	0.524	3.26E-99
TUBA1B+ H2AFZ+ HMGB2+ HIST1H4C+	DYNLT1	8.22E-95	0.265375601	0.948	0.781	1.75E-90
TUBA1B+ H2AFZ+ HMGB2+ HIST1H4C+	PGRMC1	1.27E-97	0.265018358	0.847	0.614	2.69E-93
TUBA1B+ H2AFZ+ HMGB2+ HIST1H4C+	KIF5B	6.37E-57	0.264961404	0.927	0.778	1.35E-52
TUBA1B+ H2AFZ+ HMGB2+ HIST1H4C+	MCRIP2	9.89E-80	0.264680649	0.853	0.643	2.10E-75
TUBA1B+ H2AFZ+ HMGB2+ HIST1H4C+	GTF3A	9.51E-81	0.264611956	0.862	0.599	2.02E-76
TUBA1B+ H2AFZ+ HMGB2+ HIST1H4C+	TRIM28	1.07E-111	0.26444982	0.707	0.426	2.27E-107
TUBA1B+ H2AFZ+ HMGB2+ HIST1H4C+	RTRAF	2.48E-69	0.264291268	0.913	0.757	5.27E-65
TUBA1B+ H2AFZ+ HMGB2+ HIST1H4C+	OIP5	0	0.264123625	0.334	0.01	0
TUBA1B+ H2AFZ+ HMGB2+ HIST1H4C+	IMP4	2.11E-110	0.263989529	0.708	0.426	4.50E-106
TUBA1B+ H2AFZ+ HMGB2+ HIST1H4C+	NDUFB1	1.04E-84	0.263649711	0.965	0.843	2.21E-80
TUBA1B+ H2AFZ+ HMGB2+ HIST1H4C+	EIF3A	1.81E-80	0.263568729	0.892	0.707	3.85E-76
TUBA1B+ H2AFZ+ HMGB2+ HIST1H4C+	DPY30	3.57E-92	0.263314541	0.856	0.632	7.58E-88
TUBA1B+ H2AFZ+ HMGB2+ HIST1H4C+	WDR43	6.30E-124	0.26290199	0.646	0.356	1.34E-119

TUBA1B+ H2AFZ+ HMGB2+ HIST1H4C+	TIMM50	8.60E-166	0.262731506	0.618	0.299	1.83E-161
TUBA1B+ H2AFZ+ HMGB2+ HIST1H4C+	GTPBP4	7.02E-128	0.262720391	0.605	0.324	1.49E-123
TUBA1B+ H2AFZ+ HMGB2+ HIST1H4C+	KIF11	0	0.262691816	0.273	0.011	0
TUBA1B+ H2AFZ+ HMGB2+ HIST1H4C+	ALDH7A1	3.98E-139	0.262608411	0.648	0.332	8.46E-135
TUBA1B+ H2AFZ+ HMGB2+ HIST1H4C+	ADAM15	1.06E-121	0.262396014	0.681	0.379	2.25E-117
TUBA1B+ H2AFZ+ HMGB2+ HIST1H4C+	RMI2	0	0.262341803	0.351	0.057	0
TUBA1B+ H2AFZ+ HMGB2+ HIST1H4C+	TSFM	4.64E-155	0.262307655	0.61	0.299	9.86E-151
TUBA1B+ H2AFZ+ HMGB2+ HIST1H4C+	SMAGP	8.40E-92	0.261897013	0.819	0.582	1.79E-87
TUBA1B+ H2AFZ+ HMGB2+ HIST1H4C+	EMC8	8.14E-156	0.261798386	0.609	0.296	1.73E-151
TUBA1B+ H2AFZ+ HMGB2+ HIST1H4C+	IDH3B	4.26E-107	0.261732015	0.74	0.46	9.06E-103
TUBA1B+ H2AFZ+ HMGB2+ HIST1H4C+	PSMD11	8.04E-110	0.261606775	0.73	0.472	1.71E-105
TUBA1B+ H2AFZ+ HMGB2+ HIST1H4C+	PITHD1	7.06E-148	0.260811245	0.646	0.328	1.50E-143
TUBA1B+ H2AFZ+ HMGB2+ HIST1H4C+	TMEM147	2.03E-76	0.260777331	0.875	0.663	4.32E-72
TUBA1B+ H2AFZ+ HMGB2+ HIST1H4C+	RHOBTB3	1.94E-107	0.260581993	0.768	0.476	4.12E-103
TUBA1B+ H2AFZ+ HMGB2+ HIST1H4C+	NOP10	3.29E-70	0.259991926	0.889	0.746	6.99E-66
TUBA1B+ H2AFZ+ HMGB2+ HIST1H4C+	AP1S1	5.25E-83	0.259929989	0.869	0.639	1.12E-78
TUBA1B+ H2AFZ+ HMGB2+ HIST1H4C+	METTL5	3.38E-92	0.259549252	0.829	0.585	7.19E-88
TUBA1B+ H2AFZ+ HMGB2+ HIST1H4C+	COX6A1	1.70E-92	0.259457493	0.993	0.929	3.61E-88
TUBA1B+ H2AFZ+ HMGB2+ HIST1H4C+	ARL2	9.75E-103	0.259049914	0.779	0.523	2.07E-98
TUBA1B+ H2AFZ+ HMGB2+ HIST1H4C+	BAX	1.20E-87	0.258999431	0.857	0.631	2.54E-83
TUBA1B+ H2AFZ+ HMGB2+ HIST1H4C+	ROMO1	1.23E-67	0.258970569	0.951	0.829	2.61E-63
TUBA1B+ H2AFZ+ HMGB2+ HIST1H4C+	EIF4G2	4.06E-64	0.258731613	0.953	0.812	8.64E-60

TUBA1B+ H2AFZ+ HMGB2+ HIST1H4C+	MSH6	4.17E-254	0.258542069	0.389	0.097	8.86E-250
TUBA1B+ H2AFZ+ HMGB2+ HIST1H4C+	UQCR10	3.05E-82	0.258082146	0.972	0.872	6.49E-78
TUBA1B+ H2AFZ+ HMGB2+ HIST1H4C+	NABP2	1.60E-159	0.257869468	0.631	0.305	3.41E-155
TUBA1B+ H2AFZ+ HMGB2+ HIST1H4C+	RPP25	1.00E-169	0.257599705	0.536	0.233	2.13E-165
TUBA1B+ H2AFZ+ HMGB2+ HIST1H4C+	CEP57	1.99E-118	0.257559556	0.655	0.359	4.23E-114
TUBA1B+ H2AFZ+ HMGB2+ HIST1H4C+	ENOPH1	2.98E-150	0.257206875	0.612	0.299	6.34E-146
TUBA1B+ H2AFZ+ HMGB2+ HIST1H4C+	POP5	2.32E-140	0.257116536	0.647	0.337	4.94E-136
TUBA1B+ H2AFZ+ HMGB2+ HIST1H4C+	CKAP4	9.36E-109	0.257051366	0.733	0.443	1.99E-104
TUBA1B+ H2AFZ+ HMGB2+ HIST1H4C+	WDR18	1.22E-129	0.257027859	0.621	0.335	2.58E-125
TUBA1B+ H2AFZ+ HMGB2+ HIST1H4C+	NCAPG	0	0.256826943	0.278	0.003	0
TUBA1B+ H2AFZ+ HMGB2+ HIST1H4C+	WDR77	6.14E-140	0.256810311	0.623	0.316	1.31E-135
TUBA1B+ H2AFZ+ HMGB2+ HIST1H4C+	HLA-DMA	4.25E-98	0.256773929	0.412	0.196	9.04E-94
TUBA1B+ H2AFZ+ HMGB2+ HIST1H4C+	AK6	6.36E-129	0.256599482	0.706	0.41	1.35E-124
TUBA1B+ H2AFZ+ HMGB2+ HIST1H4C+	RPL39L	0	0.256246341	0.302	0.035	0
TUBA1B+ H2AFZ+ HMGB2+ HIST1H4C+	THEM6	2.31E-151	0.256229332	0.489	0.211	4.91E-147
TUBA1B+ H2AFZ+ HMGB2+ HIST1H4C+	SLC2A4RG	5.66E-171	0.256166888	0.482	0.189	1.20E-166
TUBA1B+ H2AFZ+ HMGB2+ HIST1H4C+	PSMD12	8.48E-122	0.255268186	0.659	0.377	1.80E-117
TUBA1B+ H2AFZ+ HMGB2+ HIST1H4C+	EMC9	1.91E-210	0.254645057	0.471	0.161	4.05E-206
TUBA1B+ H2AFZ+ HMGB2+ HIST1H4C+	ABCE1	6.60E-133	0.254452258	0.652	0.35	1.40E-128
TUBA1B+ H2AFZ+ HMGB2+ HIST1H4C+	LCN2	3.56E-37	0.254415929	0.832	0.673	7.56E-33
TUBA1B+ H2AFZ+ HMGB2+ HIST1H4C+	NDUFAF4	3.96E-129	0.254223784	0.67	0.376	8.41E-125
TUBA1B+ H2AFZ+ HMGB2+ HIST1H4C+	PAK1IP1	2.24E-161	0.254098584	0.548	0.244	4.77E-157

TUBA1B+ H2AFZ+ HMGB2+ HIST1H4C+	VPS35	4.42E-86	0.253609571	0.844	0.602	9.40E-82
TUBA1B+ H2AFZ+ HMGB2+ HIST1H4C+	RPS7	7.36E-52	0.252866002	0.998	0.922	1.56E-47
TUBA1B+ H2AFZ+ HMGB2+ HIST1H4C+	UQCC3	6.93E-102	0.252765589	0.818	0.553	1.47E-97
TUBA1B+ H2AFZ+ HMGB2+ HIST1H4C+	RPS23	5.16E-44	0.252737929	0.999	0.932	1.10E-39
TUBA1B+ H2AFZ+ HMGB2+ HIST1H4C+	GNG5	4.42E-79	0.252721975	0.975	0.846	9.40E-75
TUBA1B+ H2AFZ+ HMGB2+ HIST1H4C+	CKAP5	1.01E-174	0.252720618	0.445	0.164	2.15E-170
TUBA1B+ H2AFZ+ HMGB2+ HIST1H4C+	ABRACL	3.00E-85	0.251984618	0.89	0.674	6.38E-81
TUBA1B+ H2AFZ+ HMGB2+ HIST1H4C+	TRIP13	0	0.251832583	0.323	0.017	0
TUBA1B+ H2AFZ+ HMGB2+ HIST1H4C+	CLTA	9.22E-95	0.251488092	0.968	0.834	1.96E-90
TUBA1B+ H2AFZ+ HMGB2+ HIST1H4C+	NDC80	0	0.251388822	0.26	0.006	0
TUBA1B+ H2AFZ+ HMGB2+ HIST1H4C+	NT5DC2	4.21E-304	0.250907678	0.373	0.075	8.94E-300
TUBA1B+ H2AFZ+ HMGB2+ HIST1H4C+	COX11	5.06E-112	0.250875285	0.656	0.384	1.08E-107
TUBA1B+ H2AFZ+ HMGB2+ HIST1H4C+	XRN2	1.37E-71	0.250385494	0.746	0.5	2.92E-67
TUBA1B+ H2AFZ+ HMGB2+ HIST1H4C+	ARL6IP4	1.36E-80	0.250365534	0.954	0.795	2.89E-76
SPINK4+ CLCA1+ DEFA6+ REG4+	RPL36A	3.86E-40	0.317583905	0.986	0.904	8.22E-36
SPINK4+ CLCA1+ DEFA6+ REG4+	RPL34	7.82E-58	0.315903872	1	0.979	1.66E-53
SPINK4+ CLCA1+ DEFA6+ REG4+	SYTL1	7.20E-77	0.315264734	0.312	0.128	1.53E-72
SPINK4+ CLCA1+ DEFA6+ REG4+	CBFA2T2	5.80E-36	0.315211896	0.366	0.23	1.23E-31
SPINK4+ CLCA1+ DEFA6+ REG4+	SEL1L3	3.40E-31	0.314858459	0.486	0.383	7.23E-27
SPINK4+ CLCA1+ DEFA6+ REG4+	ABLIM1	3.71E-30	0.314801997	0.574	0.485	7.89E-26
SPINK4+ CLCA1+ DEFA6+ REG4+	CALR	1.71E-19	0.308753437	0.871	0.869	3.64E-15
SPINK4+ CLCA1+ DEFA6+ REG4+	PTPRN2	8.75E-90	0.308609863	0.264	0.084	1.86E-85

SPINK4+ CLCA1+ DEFA6+ REG4+	NTN4	1.55E-95	0.308247024	0.279	0.087	3.29E-91
SPINK4+ CLCA1+ DEFA6+ REG4+	S100A13	6.79E-33	0.307500376	0.689	0.605	1.44E-28
SPINK4+ CLCA1+ DEFA6+ REG4+	KCNE3	4.39E-22	0.307453752	0.408	0.321	9.33E-18
SPINK4+ CLCA1+ DEFA6+ REG4+	C4BPB	3.55E-63	0.307226813	0.282	0.118	7.55E-59
SPINK4+ CLCA1+ DEFA6+ REG4+	QSOX1	2.23E-57	0.306867858	0.673	0.484	4.75E-53
SPINK4+ CLCA1+ DEFA6+ REG4+	FOSB	1.26E-13	0.304770393	0.752	0.692	2.68E-09
SPINK4+ CLCA1+ DEFA6+ REG4+	SLC35A1	2.61E-39	0.304568838	0.462	0.322	5.55E-35
SPINK4+ CLCA1+ DEFA6+ REG4+	TRPM4	1.72E-12	0.304198364	0.57	0.533	3.66E-08
SPINK4+ CLCA1+ DEFA6+ REG4+	CDC42EP5	5.62E-21	0.302551664	0.784	0.753	1.19E-16
SPINK4+ CLCA1+ DEFA6+ REG4+	MSI2	2.32E-25	0.301358664	0.542	0.462	4.94E-21
SPINK4+ CLCA1+ DEFA6+ REG4+	ZNF511	2.16E-26	0.300827656	0.65	0.575	4.59E-22
SPINK4+ CLCA1+ DEFA6+ REG4+	B4GALT4	3.86E-38	0.300771521	0.43	0.289	8.22E-34
SPINK4+ CLCA1+ DEFA6+ REG4+	ITGA6	4.43E-24	0.297515506	0.766	0.701	9.41E-20
SPINK4+ CLCA1+ DEFA6+ REG4+	GALNT5	5.77E-43	0.297437972	0.376	0.222	1.23E-38
SPINK4+ CLCA1+ DEFA6+ REG4+	MAN1A1	2.65E-60	0.297389836	0.36	0.177	5.64E-56
SPINK4+ CLCA1+ DEFA6+ REG4+	ETS1	6.55E-96	0.296642634	0.279	0.087	1.39E-91
SPINK4+ CLCA1+ DEFA6+ REG4+	MAPKAPK5-AS1	8.63E-20	0.296266545	0.442	0.361	1.83E-15
SPINK4+ CLCA1+ DEFA6+ REG4+	BACE2	9.16E-20	0.295792785	0.707	0.67	1.95E-15
SPINK4+ CLCA1+ DEFA6+ REG4+	BTG2	9.78E-14	0.2957632	0.745	0.663	2.08E-09
SPINK4+ CLCA1+ DEFA6+ REG4+	CRACR2A	6.43E-78	0.292868303	0.289	0.108	1.37E-73
SPINK4+ CLCA1+ DEFA6+ REG4+	GFPT1	3.58E-24	0.292862004	0.723	0.663	7.61E-20
SPINK4+ CLCA1+ DEFA6+ REG4+	MGLL	7.94E-38	0.292591833	0.694	0.592	1.69E-33

SPINK4+ CLCA1+ DEFA6+ REG4+	RPL38	3.62E-47	0.291727746	0.995	0.959	7.70E-43
SPINK4+ CLCA1+ DEFA6+ REG4+	PDIA5	1.71E-47	0.291365461	0.352	0.2	3.63E-43
SPINK4+ CLCA1+ DEFA6+ REG4+	ARL1	6.53E-22	0.290189549	0.61	0.563	1.39E-17
SPINK4+ CLCA1+ DEFA6+ REG4+	HLA-A	6.49E-58	0.289854177	0.987	0.978	1.38E-53
SPINK4+ CLCA1+ DEFA6+ REG4+	IER3	1.05E-18	0.289311108	0.765	0.695	2.24E-14
SPINK4+ CLCA1+ DEFA6+ REG4+	FAM213A	2.59E-18	0.289300076	0.572	0.508	5.51E-14
SPINK4+ CLCA1+ DEFA6+ REG4+	DPM3	5.26E-20	0.287677654	0.651	0.624	1.12E-15
SPINK4+ CLCA1+ DEFA6+ REG4+	UCP2	4.73E-28	0.287070743	0.403	0.281	1.01E-23
SPINK4+ CLCA1+ DEFA6+ REG4+	INSR	1.17E-23	0.286286961	0.538	0.438	2.49E-19
SPINK4+ CLCA1+ DEFA6+ REG4+	ARF4	1.25E-24	0.28553307	0.771	0.738	2.67E-20
SPINK4+ CLCA1+ DEFA6+ REG4+	CHD9	1.40E-14	0.283929756	0.553	0.519	2.99E-10
SPINK4+ CLCA1+ DEFA6+ REG4+	ID2	3.17E-14	0.281285776	0.741	0.708	6.74E-10
SPINK4+ CLCA1+ DEFA6+ REG4+	HM13	1.17E-19	0.279093274	0.59	0.532	2.48E-15
SPINK4+ CLCA1+ DEFA6+ REG4+	ATP2A3	2.17E-31	0.277716939	0.412	0.278	4.62E-27
SPINK4+ CLCA1+ DEFA6+ REG4+	SIL1	6.94E-21	0.275748222	0.451	0.371	1.48E-16
SPINK4+ CLCA1+ DEFA6+ REG4+	EPHB3	9.22E-28	0.275725379	0.476	0.338	1.96E-23
SPINK4+ CLCA1+ DEFA6+ REG4+	RPL13	2.15E-38	0.275418131	0.999	0.964	4.58E-34
SPINK4+ CLCA1+ DEFA6+ REG4+	RPL31	2.17E-38	0.274791745	0.995	0.956	4.62E-34
SPINK4+ CLCA1+ DEFA6+ REG4+	MT-ATP6	4.86E-37	0.274392664	0.996	0.986	1.03E-32
SPINK4+ CLCA1+ DEFA6+ REG4+	ARFGAP3	1.86E-42	0.274272145	0.422	0.267	3.96E-38
SPINK4+ CLCA1+ DEFA6+ REG4+	HLA-E	7.98E-63	0.273440208	0.911	0.863	1.70E-58
SPINK4+ CLCA1+ DEFA6+ REG4+	PRIMPOL	7.65E-67	0.27200638	0.26	0.1	1.63E-62

SPINK4+ CLCA1+ DEFA6+ REG4+	CANT1	1.88E-20	0.271781348	0.582	0.531	4.01E-16
SPINK4+ CLCA1+ DEFA6+ REG4+	TMED9	2.34E-20	0.27151322	0.796	0.77	4.97E-16
SPINK4+ CLCA1+ DEFA6+ REG4+	TOMM7	2.91E-14	0.271478306	0.938	0.869	6.19E-10
SPINK4+ CLCA1+ DEFA6+ REG4+	RPL18	1.91E-25	0.271096691	0.997	0.964	4.07E-21
SPINK4+ CLCA1+ DEFA6+ REG4+	PROX1	7.84E-14	0.27037577	0.318	0.237	1.67E-09
SPINK4+ CLCA1+ DEFA6+ REG4+	TMEM263	3.07E-42	0.268870732	0.361	0.213	6.53E-38
SPINK4+ CLCA1+ DEFA6+ REG4+	TENT5C	6.51E-49	0.26832921	0.252	0.111	1.38E-44
SPINK4+ CLCA1+ DEFA6+ REG4+	RPL37	4.07E-26	0.268264945	0.993	0.945	8.66E-22
SPINK4+ CLCA1+ DEFA6+ REG4+	STXBP1	5.99E-96	0.266646052	0.26	0.077	1.27E-91
SPINK4+ CLCA1+ DEFA6+ REG4+	PLXDC2	3.45E-76	0.266610745	0.264	0.093	7.34E-72
SPINK4+ CLCA1+ DEFA6+ REG4+	SNHG18	2.69E-14	0.266566164	0.519	0.474	5.72E-10
SPINK4+ CLCA1+ DEFA6+ REG4+	RPL37A	4.10E-42	0.262555448	0.996	0.964	8.73E-38
SPINK4+ CLCA1+ DEFA6+ REG4+	HMG20B	8.89E-18	0.262409466	0.489	0.428	1.89E-13
SPINK4+ CLCA1+ DEFA6+ REG4+	FAM114A1	2.15E-29	0.260699701	0.426	0.304	4.58E-25
SPINK4+ CLCA1+ DEFA6+ REG4+	MCF2L	1.21E-67	0.258354681	0.287	0.115	2.58E-63
SPINK4+ CLCA1+ DEFA6+ REG4+	TBC1D2	1.36E-69	0.256963276	0.252	0.092	2.90E-65
SPINK4+ CLCA1+ DEFA6+ REG4+	IFITM2	6.40E-22	0.256316598	0.539	0.428	1.36E-17
SPINK4+ CLCA1+ DEFA6+ REG4+	KRT18	4.75E-22	0.253753447	0.994	0.981	1.01E-17
SPINK4+ CLCA1+ DEFA6+ REG4+	DNTTIP1	9.96E-20	0.252447094	0.464	0.39	2.12E-15
SPINK4+ CLCA1+ DEFA6+ REG4+	ENTPD8	2.34E-150	0.251982767	0.489	0.157	4.98E-146
SPINK4+ CLCA1+ DEFA6+ REG4+	ICA1	3.06E-15	0.251556245	0.532	0.469	6.51E-11
SPINK4+ CLCA1+ DEFA6+ REG4+	WNK2	3.16E-21	0.251274714	0.437	0.345	6.71E-17

SPINK4+ CLCA1+ DEFA6+ REG4+	SLC39A7	8.80E-16	0.250816043	0.5	0.448	1.87E-11
SPINK4+ CLCA1+ DEFA6+ REG4+	KCNK6	2.30E-28	0.250186761	0.428	0.305	4.89E-24
TUBA1B+ H2AFZ+ HMGB2+ HIST1H4C+	TUBA1B	0	2.13081014	0.991	0.742	0
TUBA1B+ H2AFZ+ HMGB2+ HIST1H4C+	H2AFZ	0	2.04166508	0.996	0.774	0
TUBA1B+ H2AFZ+ HMGB2+ HIST1H4C+	HMGB2	0	2.020781888	0.907	0.445	0
TUBA1B+ H2AFZ+ HMGB2+ HIST1H4C+	HIST1H4C	7.25E-288	2.012540123	0.895	0.659	1.54E-283
TUBA1B+ H2AFZ+ HMGB2+ HIST1H4C+	PTTG1	0	1.73256918	0.727	0.108	0
TUBA1B+ H2AFZ+ HMGB2+ HIST1H4C+	STMN1	0	1.692862253	0.938	0.343	0
TUBA1B+ H2AFZ+ HMGB2+ HIST1H4C+	HMGN2	0	1.561641182	0.986	0.806	0
TUBA1B+ H2AFZ+ HMGB2+ HIST1H4C+	PCLAF	0	1.546665662	0.77	0.098	0
TUBA1B+ H2AFZ+ HMGB2+ HIST1H4C+	UBE2C	0	1.481562858	0.541	0.025	0
TUBA1B+ H2AFZ+ HMGB2+ HIST1H4C+	TUBB	0	1.455595137	0.949	0.62	0
TUBA1B+ H2AFZ+ HMGB2+ HIST1H4C+	CKS2	0	1.308546852	0.855	0.413	0
TUBA1B+ H2AFZ+ HMGB2+ HIST1H4C+	HSPD1	0	1.270686678	0.982	0.766	0
TUBA1B+ H2AFZ+ HMGB2+ HIST1H4C+	RANBP1	0	1.267583545	0.954	0.665	0
TUBA1B+ H2AFZ+ HMGB2+ HIST1H4C+	CENPW	0	1.263989452	0.824	0.191	0
TUBA1B+ H2AFZ+ HMGB2+ HIST1H4C+	MKI67	0	1.259137611	0.67	0.038	0
TUBA1B+ H2AFZ+ HMGB2+ HIST1H4C+	OLFM4	4.73E-264	1.252022056	0.873	0.581	1.00E-259
TUBA1B+ H2AFZ+ HMGB2+ HIST1H4C+	HMGB1	0	1.250784919	0.999	0.909	0
TUBA1B+ H2AFZ+ HMGB2+ HIST1H4C+	RAN	0	1.203619635	0.989	0.797	0
TUBA1B+ H2AFZ+ HMGB2+ HIST1H4C+	LDHB	0	1.192645624	0.722	0.346	0
TUBA1B+ H2AFZ+ HMGB2+ HIST1H4C+	CKS1B	0	1.174910181	0.847	0.363	0

TUBA1B+ H2AFZ+ HMGB2+ HIST1H4C+	CENPF	0	1.145975253	0.586	0.046	0
TUBA1B+ H2AFZ+ HMGB2+ HIST1H4C+	RRM2	0	1.142957788	0.595	0.026	0
TUBA1B+ H2AFZ+ HMGB2+ HIST1H4C+	ENO1	0	1.133658281	0.983	0.796	0
TUBA1B+ H2AFZ+ HMGB2+ HIST1H4C+	TUBB4B	0	1.130446331	0.964	0.753	0
TUBA1B+ H2AFZ+ HMGB2+ HIST1H4C+	TOP2A	0	1.093235655	0.525	0.021	0
TUBA1B+ H2AFZ+ HMGB2+ HIST1H4C+	CCNB1	0	1.076320614	0.529	0.052	0
TUBA1B+ H2AFZ+ HMGB2+ HIST1H4C+	IDH2	0	1.074384347	0.948	0.646	0
TUBA1B+ H2AFZ+ HMGB2+ HIST1H4C+	PCNA	0	1.050694284	0.754	0.3	0
TUBA1B+ H2AFZ+ HMGB2+ HIST1H4C+	HSPE1	0	1.047372938	0.993	0.843	0
TUBA1B+ H2AFZ+ HMGB2+ HIST1H4C+	TK1	0	1.015971951	0.701	0.071	0
TUBA1B+ H2AFZ+ HMGB2+ HIST1H4C+	BIRC5	0	1.013744333	0.615	0.038	0
TUBA1B+ H2AFZ+ HMGB2+ HIST1H4C+	PTMA	0	1.006879044	1	0.982	0
TUBA1B+ H2AFZ+ HMGB2+ HIST1H4C+	SMC4	0	0.998753386	0.779	0.252	0
TUBA1B+ H2AFZ+ HMGB2+ HIST1H4C+	FABP5	0	0.971951209	0.894	0.538	0
TUBA1B+ H2AFZ+ HMGB2+ HIST1H4C+	MAD2L1	0	0.962268563	0.679	0.072	0
TUBA1B+ H2AFZ+ HMGB2+ HIST1H4C+	TYMS	0	0.961608624	0.666	0.092	0
TUBA1B+ H2AFZ+ HMGB2+ HIST1H4C+	DUT	0	0.958693091	0.85	0.499	0
TUBA1B+ H2AFZ+ HMGB2+ HIST1H4C+	CDKN3	0	0.951498833	0.622	0.056	0
TUBA1B+ H2AFZ+ HMGB2+ HIST1H4C+	HSP90AA1	0	0.926388173	0.994	0.904	0
TUBA1B+ H2AFZ+ HMGB2+ HIST1H4C+	ARL6IP1	1.73E-210	0.904944364	0.931	0.741	3.67E-206
TUBA1B+ H2AFZ+ HMGB2+ HIST1H4C+	SNRPG	0	0.890090733	0.986	0.82	0
TUBA1B+ H2AFZ+ HMGB2+ HIST1H4C+	CDK1	0	0.885264644	0.454	0.014	0

TUBA1B+ H2AFZ+ HMGB2+ HIST1H4C+	DBI	0	0.883966496	0.989	0.849	0
TUBA1B+ H2AFZ+ HMGB2+ HIST1H4C+	PA2G4	0	0.883546002	0.954	0.7	0
TUBA1B+ H2AFZ+ HMGB2+ HIST1H4C+	DEK	0	0.876900336	0.965	0.738	0
TUBA1B+ H2AFZ+ HMGB2+ HIST1H4C+	SNRPD1	0	0.875685648	0.945	0.675	0
TUBA1B+ H2AFZ+ HMGB2+ HIST1H4C+	SNRPB	0	0.873495994	0.967	0.736	0
TUBA1B+ H2AFZ+ HMGB2+ HIST1H4C+	DTYMK	0	0.872899372	0.784	0.227	0
TUBA1B+ H2AFZ+ HMGB2+ HIST1H4C+	NCL	0	0.871650122	0.957	0.753	0
TUBA1B+ H2AFZ+ HMGB2+ HIST1H4C+	NASP	0	0.870481375	0.822	0.373	0
TUBA1B+ H2AFZ+ HMGB2+ HIST1H4C+	NUSAP1	0	0.867774109	0.511	0.015	0
TUBA1B+ H2AFZ+ HMGB2+ HIST1H4C+	DHFR	0	0.85804533	0.721	0.135	0
TUBA1B+ H2AFZ+ HMGB2+ HIST1H4C+	NPM1	0	0.856162138	0.995	0.85	0
TUBA1B+ H2AFZ+ HMGB2+ HIST1H4C+	GGCT	0	0.854597419	0.925	0.571	0
TUBA1B+ H2AFZ+ HMGB2+ HIST1H4C+	CDC20	0	0.850864623	0.5	0.027	0
TUBA1B+ H2AFZ+ HMGB2+ HIST1H4C+	PGAM1	0	0.848568262	0.93	0.642	0
TUBA1B+ H2AFZ+ HMGB2+ HIST1H4C+	ZWINT	0	0.842537848	0.676	0.105	0
TUBA1B+ H2AFZ+ HMGB2+ HIST1H4C+	HNRNPA2B1	0	0.818433706	0.989	0.889	0
TUBA1B+ H2AFZ+ HMGB2+ HIST1H4C+	KPNA2	0	0.816574029	0.586	0.136	0
TUBA1B+ H2AFZ+ HMGB2+ HIST1H4C+	LSM4	0	0.814900872	0.946	0.692	0
TUBA1B+ H2AFZ+ HMGB2+ HIST1H4C+	PSMA7	0	0.812497479	0.979	0.858	0
TUBA1B+ H2AFZ+ HMGB2+ HIST1H4C+	SNRPF	0	0.811716132	0.976	0.753	0
TUBA1B+ H2AFZ+ HMGB2+ HIST1H4C+	TPI1	0	0.811561824	0.991	0.892	0
TUBA1B+ H2AFZ+ HMGB2+ HIST1H4C+	UBE2T	0	0.811430575	0.677	0.107	0

TUBA1B+ H2AFZ+ HMGB2+ HIST1H4C+	NUCKS1	0	0.810506488	0.936	0.679	0
TUBA1B+ H2AFZ+ HMGB2+ HIST1H4C+	UBE2S	0	0.800457321	0.651	0.208	0
TUBA1B+ H2AFZ+ HMGB2+ HIST1H4C+	NME1	0	0.79170732	0.88	0.551	0
TUBA1B+ H2AFZ+ HMGB2+ HIST1H4C+	ANP32B	0	0.79158921	0.96	0.699	0
TUBA1B+ H2AFZ+ HMGB2+ HIST1H4C+	RPA3	0	0.787686039	0.875	0.439	0
TUBA1B+ H2AFZ+ HMGB2+ HIST1H4C+	GLO1	0	0.787520562	0.876	0.512	0
TUBA1B+ H2AFZ+ HMGB2+ HIST1H4C+	H2AFV	0	0.776302935	0.952	0.697	0
TUBA1B+ H2AFZ+ HMGB2+ HIST1H4C+	LSM3	0	0.77568749	0.962	0.71	0
TUBA1B+ H2AFZ+ HMGB2+ HIST1H4C+	SMC2	0	0.77499721	0.701	0.157	0
TUBA1B+ H2AFZ+ HMGB2+ HIST1H4C+	YBX1	0	0.768584985	0.997	0.894	0
TUBA1B+ H2AFZ+ HMGB2+ HIST1H4C+	PRDX2	0	0.767734217	0.955	0.767	0
TUBA1B+ H2AFZ+ HMGB2+ HIST1H4C+	HNRNPA3	0	0.76426895	0.956	0.761	0
TUBA1B+ H2AFZ+ HMGB2+ HIST1H4C+	TUBA1C	6.47E-236	0.763153726	0.879	0.598	1.38E-231
TUBA1B+ H2AFZ+ HMGB2+ HIST1H4C+	MIF	0	0.76230676	0.983	0.856	0
TUBA1B+ H2AFZ+ HMGB2+ HIST1H4C+	CENPX	1.86E-300	0.762141258	0.88	0.584	3.96E-296
TUBA1B+ H2AFZ+ HMGB2+ HIST1H4C+	FDPS	9.37E-276	0.759497319	0.89	0.587	1.99E-271
TUBA1B+ H2AFZ+ HMGB2+ HIST1H4C+	EBP	0	0.756367739	0.881	0.536	0
TUBA1B+ H2AFZ+ HMGB2+ HIST1H4C+	GSTP1	0	0.752449068	0.996	0.91	0
TUBA1B+ H2AFZ+ HMGB2+ HIST1H4C+	CYC1	0	0.751084639	0.967	0.774	0
TUBA1B+ H2AFZ+ HMGB2+ HIST1H4C+	WDR34	0	0.749436786	0.766	0.284	0
TUBA1B+ H2AFZ+ HMGB2+ HIST1H4C+	SOD1	0	0.748468466	0.991	0.865	0
TUBA1B+ H2AFZ+ HMGB2+ HIST1H4C+	CCNB2	0	0.745568533	0.483	0.046	0

TUBA1B+ H2AFZ+ HMGB2+ HIST1H4C+	PTGES3	0	0.744359808	0.977	0.796	0
TUBA1B+ H2AFZ+ HMGB2+ HIST1H4C+	CACYBP	0	0.742349484	0.899	0.532	0
TUBA1B+ H2AFZ+ HMGB2+ HIST1H4C+	LSM5	0	0.741387337	0.953	0.697	0
TUBA1B+ H2AFZ+ HMGB2+ HIST1H4C+	MCM7	0	0.737478199	0.718	0.238	0
TUBA1B+ H2AFZ+ HMGB2+ HIST1H4C+	CENPM	0	0.737301971	0.619	0.06	0
TUBA1B+ H2AFZ+ HMGB2+ HIST1H4C+	TPX2	0	0.730394399	0.509	0.024	0
TUBA1B+ H2AFZ+ HMGB2+ HIST1H4C+	HSP90AB1	6.48E-284	0.728205667	0.991	0.863	1.38E-279
TUBA1B+ H2AFZ+ HMGB2+ HIST1H4C+	EIF5A	5.34E-183	0.718148701	0.891	0.674	1.14E-178
TUBA1B+ H2AFZ+ HMGB2+ HIST1H4C+	HMGN1	0	0.715768986	0.989	0.846	0
TUBA1B+ H2AFZ+ HMGB2+ HIST1H4C+	LMNB1	0	0.71476712	0.696	0.143	0
TUBA1B+ H2AFZ+ HMGB2+ HIST1H4C+	PKM	1.18E-258	0.713788536	0.968	0.774	2.50E-254
TUBA1B+ H2AFZ+ HMGB2+ HIST1H4C+	MZT2A	0	0.713186385	0.956	0.722	0
TUBA1B+ H2AFZ+ HMGB2+ HIST1H4C+	CCT5	0	0.713134122	0.911	0.625	0
TUBA1B+ H2AFZ+ HMGB2+ HIST1H4C+	ACAT2	0	0.710573391	0.735	0.301	0
TUBA1B+ H2AFZ+ HMGB2+ HIST1H4C+	HMGB3	0	0.707393105	0.809	0.363	0
TUBA1B+ H2AFZ+ HMGB2+ HIST1H4C+	PPIA	0	0.698770746	0.996	0.892	0
TUBA1B+ H2AFZ+ HMGB2+ HIST1H4C+	SIVA1	0	0.696198433	0.903	0.605	0
TUBA1B+ H2AFZ+ HMGB2+ HIST1H4C+	CKLF	0	0.680930217	0.862	0.473	0
TUBA1B+ H2AFZ+ HMGB2+ HIST1H4C+	MLEC	7.02E-276	0.679785414	0.97	0.776	1.49E-271
TUBA1B+ H2AFZ+ HMGB2+ HIST1H4C+	MGST1	3.44E-224	0.676209578	0.933	0.715	7.31E-220
TUBA1B+ H2AFZ+ HMGB2+ HIST1H4C+	GGH	3.16E-301	0.67486194	0.856	0.504	6.73E-297
TUBA1B+ H2AFZ+ HMGB2+ HIST1H4C+	ATP5MC3	0	0.67395221	0.99	0.866	0

TUBA1B+ H2AFZ+ HMGB2+ HIST1H4C+	NDUFAB1	0	0.672354345	0.962	0.764	0
TUBA1B+ H2AFZ+ HMGB2+ HIST1H4C+	GMNN	0	0.667297756	0.767	0.322	0
TUBA1B+ H2AFZ+ HMGB2+ HIST1H4C+	PSME2	1.30E-267	0.665511749	0.949	0.778	2.76E-263
TUBA1B+ H2AFZ+ HMGB2+ HIST1H4C+	TOMM40	0	0.661301477	0.835	0.442	0
TUBA1B+ H2AFZ+ HMGB2+ HIST1H4C+	RAD21	3.03E-223	0.657278667	0.782	0.491	6.44E-219
TUBA1B+ H2AFZ+ HMGB2+ HIST1H4C+	DNMT1	0	0.65436034	0.683	0.19	0
TUBA1B+ H2AFZ+ HMGB2+ HIST1H4C+	VDAC1	3.05E-221	0.65319939	0.969	0.809	6.49E-217
TUBA1B+ H2AFZ+ HMGB2+ HIST1H4C+	SET	1.30E-254	0.652184705	0.97	0.782	2.76E-250
TUBA1B+ H2AFZ+ HMGB2+ HIST1H4C+	GINS2	0	0.650180397	0.573	0.069	0
TUBA1B+ H2AFZ+ HMGB2+ HIST1H4C+	PLP2	7.17E-280	0.649440207	0.949	0.702	1.52E-275
TUBA1B+ H2AFZ+ HMGB2+ HIST1H4C+	SERBP1	0	0.645849683	0.971	0.802	0
TUBA1B+ H2AFZ+ HMGB2+ HIST1H4C+	HNRNPAB	1.06E-286	0.64174652	0.943	0.726	2.25E-282
TUBA1B+ H2AFZ+ HMGB2+ HIST1H4C+	MZT2B	0	0.639887078	0.978	0.833	0
TUBA1B+ H2AFZ+ HMGB2+ HIST1H4C+	PGP	0	0.638699355	0.767	0.279	0
TUBA1B+ H2AFZ+ HMGB2+ HIST1H4C+	BUB3	1.59E-271	0.635728236	0.803	0.451	3.39E-267
TUBA1B+ H2AFZ+ HMGB2+ HIST1H4C+	SRSF3	2.14E-281	0.634573229	0.969	0.795	4.54E-277
TUBA1B+ H2AFZ+ HMGB2+ HIST1H4C+	ATP5IF1	2.09E-293	0.633891161	0.98	0.859	4.45E-289
TUBA1B+ H2AFZ+ HMGB2+ HIST1H4C+	ERH	8.88E-281	0.633128444	0.943	0.755	1.89E-276
TUBA1B+ H2AFZ+ HMGB2+ HIST1H4C+	CCNA2	0	0.632454873	0.464	0.011	0
TUBA1B+ H2AFZ+ HMGB2+ HIST1H4C+	JPT1	1.14E-247	0.628168219	0.944	0.697	2.43E-243
TUBA1B+ H2AFZ+ HMGB2+ HIST1H4C+	TCP1	3.00E-293	0.627019631	0.868	0.557	6.38E-289
TUBA1B+ H2AFZ+ HMGB2+ HIST1H4C+	SLBP	2.39E-307	0.620192257	0.68	0.305	5.08E-303

TUBA1B+ H2AFZ+ HMGB2+ HIST1H4C+	PSMA4	7.67E-230	0.619513211	0.923	0.724	1.63E-225
TUBA1B+ H2AFZ+ HMGB2+ HIST1H4C+	CCT2	7.15E-286	0.619315208	0.899	0.605	1.52E-281
TUBA1B+ H2AFZ+ HMGB2+ HIST1H4C+	EIF2S2	4.74E-262	0.614230949	0.953	0.739	1.01E-257
TUBA1B+ H2AFZ+ HMGB2+ HIST1H4C+	MINOS1	0	0.609964199	0.99	0.865	0
TUBA1B+ H2AFZ+ HMGB2+ HIST1H4C+	MCM3	0	0.606668352	0.638	0.178	0
TUBA1B+ H2AFZ+ HMGB2+ HIST1H4C+	TXN	6.44E-305	0.603868569	0.994	0.909	1.37E-300
TUBA1B+ H2AFZ+ HMGB2+ HIST1H4C+	TKT	6.84E-251	0.603831561	0.948	0.695	1.45E-246
TUBA1B+ H2AFZ+ HMGB2+ HIST1H4C+	ATP5MC1	1.69E-214	0.603003358	0.957	0.745	3.60E-210
TUBA1B+ H2AFZ+ HMGB2+ HIST1H4C+	COTL1	2.55E-303	0.602702769	0.769	0.374	5.42E-299
TUBA1B+ H2AFZ+ HMGB2+ HIST1H4C+	NUDT1	0	0.602358994	0.735	0.204	0
TUBA1B+ H2AFZ+ HMGB2+ HIST1H4C+	CENPK	0	0.601563568	0.566	0.055	0
TUBA1B+ H2AFZ+ HMGB2+ HIST1H4C+	PSMB2	1.08E-290	0.601355434	0.927	0.638	2.29E-286
TUBA1B+ H2AFZ+ HMGB2+ HIST1H4C+	RAB5IF	9.06E-209	0.600081157	0.901	0.652	1.93E-204
TUBA1B+ H2AFZ+ HMGB2+ HIST1H4C+	SLIRP	4.98E-247	0.598872481	0.95	0.791	1.06E-242
TUBA1B+ H2AFZ+ HMGB2+ HIST1H4C+	HSPA8	1.57E-144	0.595492501	0.97	0.815	3.33E-140
TUBA1B+ H2AFZ+ HMGB2+ HIST1H4C+	HNRNPF	3.59E-275	0.594141768	0.944	0.728	7.64E-271
TUBA1B+ H2AFZ+ HMGB2+ HIST1H4C+	CA9	2.13E-140	0.593709472	0.451	0.196	4.53E-136
TUBA1B+ H2AFZ+ HMGB2+ HIST1H4C+	HMMR	0	0.593323887	0.393	0.015	0
TUBA1B+ H2AFZ+ HMGB2+ HIST1H4C+	SRSF2	2.81E-263	0.592740515	0.919	0.692	5.98E-259
TUBA1B+ H2AFZ+ HMGB2+ HIST1H4C+	PBK	0	0.5923109	0.461	0.012	0
TUBA1B+ H2AFZ+ HMGB2+ HIST1H4C+	ANP32E	0	0.59204176	0.632	0.206	0
TUBA1B+ H2AFZ+ HMGB2+ HIST1H4C+	PGK1	1.38E-191	0.591357097	0.937	0.728	2.94E-187

TUBA1B+ H2AFZ+ HMGB2+ HIST1H4C+	HNRNPD	1.10E-256	0.591200696	0.858	0.576	2.34E-252
TUBA1B+ H2AFZ+ HMGB2+ HIST1H4C+	TAGLN2	2.11E-278	0.589934709	0.966	0.767	4.49E-274
TUBA1B+ H2AFZ+ HMGB2+ HIST1H4C+	ATAD2	0	0.589832946	0.504	0.067	0
TUBA1B+ H2AFZ+ HMGB2+ HIST1H4C+	HNRNPR	2.77E-271	0.589532533	0.881	0.575	5.90E-267
TUBA1B+ H2AFZ+ HMGB2+ HIST1H4C+	SKA2	0	0.58573119	0.664	0.177	0
TUBA1B+ H2AFZ+ HMGB2+ HIST1H4C+	DNAJC9	0	0.584462299	0.677	0.203	0
TUBA1B+ H2AFZ+ HMGB2+ HIST1H4C+	HNRNPM	2.08E-230	0.58437823	0.885	0.638	4.42E-226
TUBA1B+ H2AFZ+ HMGB2+ HIST1H4C+	LDHA	2.43E-161	0.581721444	0.978	0.864	5.16E-157
TUBA1B+ H2AFZ+ HMGB2+ HIST1H4C+	KIF20B	0	0.581592852	0.53	0.068	0
TUBA1B+ H2AFZ+ HMGB2+ HIST1H4C+	MRPL51	4.97E-254	0.581129011	0.929	0.704	1.06E-249
TUBA1B+ H2AFZ+ HMGB2+ HIST1H4C+	PRKDC	6.10E-308	0.580956702	0.792	0.412	1.30E-303
TUBA1B+ H2AFZ+ HMGB2+ HIST1H4C+	AP2S1	1.34E-287	0.579332365	0.948	0.726	2.84E-283
TUBA1B+ H2AFZ+ HMGB2+ HIST1H4C+	SNRPE	4.78E-274	0.579110933	0.968	0.768	1.02E-269
TUBA1B+ H2AFZ+ HMGB2+ HIST1H4C+	HELLS	0	0.579050465	0.501	0.061	0
TUBA1B+ H2AFZ+ HMGB2+ HIST1H4C+	CCDC34	0	0.578780213	0.724	0.255	0
TUBA1B+ H2AFZ+ HMGB2+ HIST1H4C+	PRELID1	1.69E-236	0.577563746	0.957	0.809	3.58E-232
TUBA1B+ H2AFZ+ HMGB2+ HIST1H4C+	TALDO1	6.80E-267	0.577228055	0.946	0.72	1.45E-262
TUBA1B+ H2AFZ+ HMGB2+ HIST1H4C+	PARK7	4.02E-272	0.576426332	0.964	0.776	8.55E-268
TUBA1B+ H2AFZ+ HMGB2+ HIST1H4C+	IFITM1	4.57E-123	0.57313291	0.801	0.52	9.71E-119
TUBA1B+ H2AFZ+ HMGB2+ HIST1H4C+	MYBL2	0	0.571742468	0.461	0.014	0
TUBA1B+ H2AFZ+ HMGB2+ HIST1H4C+	HMGA1	3.19E-261	0.571270774	0.931	0.663	6.78E-257
TUBA1B+ H2AFZ+ HMGB2+ HIST1H4C+	CYCS	5.87E-224	0.57034284	0.982	0.824	1.25E-219

TUBA1B+ H2AFZ+ HMGB2+ HIST1H4C+	GCHFR	2.65E-283	0.569407531	0.743	0.347	5.64E-279
TUBA1B+ H2AFZ+ HMGB2+ HIST1H4C+	ATP5ME	3.32E-220	0.567701859	0.987	0.905	7.06E-216
TUBA1B+ H2AFZ+ HMGB2+ HIST1H4C+	GAPDH	2.64E-250	0.566146195	1	0.973	5.61E-246
TUBA1B+ H2AFZ+ HMGB2+ HIST1H4C+	PPM1G	8.20E-292	0.565099314	0.86	0.535	1.74E-287
TUBA1B+ H2AFZ+ HMGB2+ HIST1H4C+	C1QBP	8.76E-198	0.562961371	0.93	0.695	1.86E-193
TUBA1B+ H2AFZ+ HMGB2+ HIST1H4C+	ASPM	0	0.561580423	0.367	0.009	0
TUBA1B+ H2AFZ+ HMGB2+ HIST1H4C+	CXCL3	5.55E-96	0.561122126	0.597	0.349	1.18E-91
TUBA1B+ H2AFZ+ HMGB2+ HIST1H4C+	PDCD5	9.18E-268	0.559747546	0.93	0.661	1.95E-263
TUBA1B+ H2AFZ+ HMGB2+ HIST1H4C+	IFITM3	3.38E-119	0.559049652	0.929	0.721	7.19E-115
TUBA1B+ H2AFZ+ HMGB2+ HIST1H4C+	TMPO	0	0.558474793	0.754	0.355	0
TUBA1B+ H2AFZ+ HMGB2+ HIST1H4C+	SQLE	1.34E-196	0.557931402	0.662	0.326	2.86E-192
TUBA1B+ H2AFZ+ HMGB2+ HIST1H4C+	H2AFX	0	0.556316102	0.592	0.174	0
TUBA1B+ H2AFZ+ HMGB2+ HIST1H4C+	YWHAH	3.74E-257	0.555476115	0.895	0.608	7.95E-253
TUBA1B+ H2AFZ+ HMGB2+ HIST1H4C+	NHP2	6.71E-247	0.552258645	0.935	0.695	1.43E-242
TUBA1B+ H2AFZ+ HMGB2+ HIST1H4C+	CENPN	0	0.55222756	0.595	0.092	0
TUBA1B+ H2AFZ+ HMGB2+ HIST1H4C+	PRDX1	8.87E-190	0.548891204	0.974	0.838	1.89E-185
TUBA1B+ H2AFZ+ HMGB2+ HIST1H4C+	NUDC	1.63E-244	0.546900436	0.869	0.604	3.46E-240
TUBA1B+ H2AFZ+ HMGB2+ HIST1H4C+	UQCRH	1.77E-269	0.546653545	0.991	0.883	3.76E-265
TUBA1B+ H2AFZ+ HMGB2+ HIST1H4C+	PAICS	4.32E-279	0.546508298	0.743	0.381	9.19E-275
TUBA1B+ H2AFZ+ HMGB2+ HIST1H4C+	RAD51AP1	0	0.545788277	0.486	0.022	0
TUBA1B+ H2AFZ+ HMGB2+ HIST1H4C+	ILF2	8.06E-237	0.545775266	0.859	0.567	1.71E-232
TUBA1B+ H2AFZ+ HMGB2+ HIST1H4C+	CBX3	1.42E-240	0.545266067	0.946	0.7	3.02E-236

TUBA1B+ H2AFZ+ HMGB2+ HIST1H4C+	YWHAQ	2.97E-256	0.543799886	0.936	0.697	6.32E-252
TUBA1B+ H2AFZ+ HMGB2+ HIST1H4C+	TMA7	5.39E-304	0.539721047	0.994	0.931	1.15E-299
TUBA1B+ H2AFZ+ HMGB2+ HIST1H4C+	PSMA3	7.53E-238	0.539716026	0.88	0.607	1.60E-233
TUBA1B+ H2AFZ+ HMGB2+ HIST1H4C+	RRM1	0	0.539412327	0.615	0.155	0
TUBA1B+ H2AFZ+ HMGB2+ HIST1H4C+	TIMM13	9.48E-200	0.539377349	0.92	0.741	2.02E-195
TUBA1B+ H2AFZ+ HMGB2+ HIST1H4C+	HLA-DRA	2.63E-92	0.535325359	0.564	0.336	5.59E-88
TUBA1B+ H2AFZ+ HMGB2+ HIST1H4C+	FEN1	0	0.534007985	0.5	0.067	0
TUBA1B+ H2AFZ+ HMGB2+ HIST1H4C+	SRSF7	2.83E-225	0.533864993	0.902	0.661	6.02E-221
TUBA1B+ H2AFZ+ HMGB2+ HIST1H4C+	ELOC	1.04E-225	0.533766047	0.926	0.683	2.21E-221
TUBA1B+ H2AFZ+ HMGB2+ HIST1H4C+	SLC25A5	7.15E-223	0.533761249	0.987	0.88	1.52E-218
TUBA1B+ H2AFZ+ HMGB2+ HIST1H4C+	DDX39A	0	0.532291513	0.701	0.293	0
TUBA1B+ H2AFZ+ HMGB2+ HIST1H4C+	NDUFS8	5.71E-251	0.531866182	0.941	0.696	1.21E-246
TUBA1B+ H2AFZ+ HMGB2+ HIST1H4C+	GSTO1	7.98E-237	0.530776165	0.915	0.645	1.70E-232
TUBA1B+ H2AFZ+ HMGB2+ HIST1H4C+	USP1	2.98E-276	0.529270563	0.718	0.346	6.34E-272
TUBA1B+ H2AFZ+ HMGB2+ HIST1H4C+	CDK4	1.85E-263	0.527632999	0.8	0.445	3.94E-259
TUBA1B+ H2AFZ+ HMGB2+ HIST1H4C+	HNRNPC	1.38E-207	0.526273688	0.954	0.777	2.94E-203
TUBA1B+ H2AFZ+ HMGB2+ HIST1H4C+	ECHS1	6.65E-235	0.526086623	0.932	0.709	1.41E-230
TUBA1B+ H2AFZ+ HMGB2+ HIST1H4C+	PRMT1	1.73E-235	0.525408861	0.808	0.5	3.68E-231
TUBA1B+ H2AFZ+ HMGB2+ HIST1H4C+	CCT6A	2.81E-195	0.525251682	0.905	0.667	5.98E-191
TUBA1B+ H2AFZ+ HMGB2+ HIST1H4C+	SNRNP25	0	0.524829058	0.727	0.269	0
TUBA1B+ H2AFZ+ HMGB2+ HIST1H4C+	SLC25A39	3.53E-217	0.524466314	0.851	0.572	7.51E-213
TUBA1B+ H2AFZ+ HMGB2+ HIST1H4C+	CCT7	3.95E-215	0.523989303	0.867	0.595	8.40E-211

TUBA1B+ H2AFZ+ HMGB2+ HIST1H4C+	CNIH4	3.40E-218	0.523892383	0.832	0.512	7.23E-214
TUBA1B+ H2AFZ+ HMGB2+ HIST1H4C+	KPNB1	5.90E-235	0.52258586	0.825	0.53	1.25E-230
TUBA1B+ H2AFZ+ HMGB2+ HIST1H4C+	NOP56	2.59E-262	0.521300343	0.786	0.427	5.51E-258
TUBA1B+ H2AFZ+ HMGB2+ HIST1H4C+	AURKA	0	0.521294501	0.419	0.064	0
TUBA1B+ H2AFZ+ HMGB2+ HIST1H4C+	NUDT5	3.42E-291	0.521173467	0.819	0.449	7.26E-287
TUBA1B+ H2AFZ+ HMGB2+ HIST1H4C+	RBBP7	9.48E-280	0.520365695	0.834	0.479	2.02E-275
TUBA1B+ H2AFZ+ HMGB2+ HIST1H4C+	NDUFS6	1.52E-239	0.519261565	0.97	0.804	3.24E-235
TUBA1B+ H2AFZ+ HMGB2+ HIST1H4C+	AHCY	3.29E-215	0.518776476	0.812	0.507	7.00E-211
TUBA1B+ H2AFZ+ HMGB2+ HIST1H4C+	PSIP1	0	0.518523418	0.649	0.221	0
TUBA1B+ H2AFZ+ HMGB2+ HIST1H4C+	MRPL13	4.75E-230	0.517897345	0.892	0.611	1.01E-225
TUBA1B+ H2AFZ+ HMGB2+ HIST1H4C+	TMEM97	0	0.517126436	0.655	0.215	0
TUBA1B+ H2AFZ+ HMGB2+ HIST1H4C+	SYNCRIP	1.44E-229	0.516810472	0.853	0.568	3.07E-225
TUBA1B+ H2AFZ+ HMGB2+ HIST1H4C+	NDUFC2	1.16E-214	0.516084648	0.964	0.812	2.47E-210
TUBA1B+ H2AFZ+ HMGB2+ HIST1H4C+	DDX21	6.04E-217	0.515834085	0.879	0.597	1.28E-212
TUBA1B+ H2AFZ+ HMGB2+ HIST1H4C+	BRI3BP	5.76E-300	0.515475231	0.77	0.365	1.23E-295
TUBA1B+ H2AFZ+ HMGB2+ HIST1H4C+	UBE2N	5.69E-258	0.515114322	0.887	0.565	1.21E-253
TUBA1B+ H2AFZ+ HMGB2+ HIST1H4C+	PSMA5	5.50E-228	0.514268265	0.901	0.647	1.17E-223
TUBA1B+ H2AFZ+ HMGB2+ HIST1H4C+	POLD2	1.27E-270	0.512546523	0.805	0.44	2.71E-266
TUBA1B+ H2AFZ+ HMGB2+ HIST1H4C+	BANF1	3.50E-233	0.512345427	0.941	0.694	7.44E-229
TUBA1B+ H2AFZ+ HMGB2+ HIST1H4C+	CCT8	1.45E-193	0.511557344	0.897	0.652	3.09E-189
TUBA1B+ H2AFZ+ HMGB2+ HIST1H4C+	MZT1	0	0.51065234	0.677	0.269	0
TUBA1B+ H2AFZ+ HMGB2+ HIST1H4C+	SSRP1	4.50E-271	0.510374802	0.83	0.464	9.57E-267

TUBA1B+ H2AFZ+ HMGB2+ HIST1H4C+	PHB	9.82E-194	0.509289598	0.917	0.714	2.09E-189
TUBA1B+ H2AFZ+ HMGB2+ HIST1H4C+	NAP1L1	4.05E-180	0.508947889	0.941	0.669	8.61E-176
TUBA1B+ H2AFZ+ HMGB2+ HIST1H4C+	HDGF	5.43E-232	0.508826611	0.863	0.566	1.16E-227
TUBA1B+ H2AFZ+ HMGB2+ HIST1H4C+	TMEM106C	2.01E-194	0.508715207	0.929	0.697	4.28E-190
TUBA1B+ H2AFZ+ HMGB2+ HIST1H4C+	NDUFS5	1.10E-218	0.507981806	0.984	0.836	2.34E-214
TUBA1B+ H2AFZ+ HMGB2+ HIST1H4C+	HSPA9	2.45E-209	0.5077415	0.869	0.598	5.21E-205
TUBA1B+ H2AFZ+ HMGB2+ HIST1H4C+	EIF4EBP1	1.61E-210	0.505991986	0.802	0.455	3.42E-206
TUBA1B+ H2AFZ+ HMGB2+ HIST1H4C+	EIF1AX	5.77E-177	0.505724498	0.953	0.75	1.23E-172
TUBA1B+ H2AFZ+ HMGB2+ HIST1H4C+	DCTPP1	3.54E-209	0.505398301	0.875	0.602	7.54E-205
TUBA1B+ H2AFZ+ HMGB2+ HIST1H4C+	CSTB	3.10E-89	0.503895189	0.96	0.833	6.59E-85
TUBA1B+ H2AFZ+ HMGB2+ HIST1H4C+	NAA20	5.35E-152	0.502672785	0.794	0.507	1.14E-147
TUBA1B+ H2AFZ+ HMGB2+ HIST1H4C+	BOLA3	2.23E-261	0.502176397	0.877	0.522	4.75E-257
TUBA1B+ H2AFZ+ HMGB2+ HIST1H4C+	PDIA6	1.06E-197	0.502088561	0.956	0.77	2.26E-193
TUBA1B+ H2AFZ+ HMGB2+ HIST1H4C+	CHCHD2	8.07E-271	0.50002598	0.996	0.92	1.72E-266
TUBA1B+ H2AFZ+ HMGB2+ HIST1H4C+	TPRKB	7.31E-277	0.499346188	0.845	0.484	1.55E-272
TUBA1B+ H2AFZ+ HMGB2+ HIST1H4C+	YWHAB	2.14E-151	0.498546205	0.97	0.84	4.55E-147
TUBA1B+ H2AFZ+ HMGB2+ HIST1H4C+	ATP5F1B	9.99E-192	0.497481177	0.978	0.853	2.12E-187
TUBA1B+ H2AFZ+ HMGB2+ HIST1H4C+	CDT1	0	0.49287165	0.5	0.051	0
TUBA1B+ H2AFZ+ HMGB2+ HIST1H4C+	ARPC5L	8.79E-234	0.492222254	0.86	0.545	1.87E-229
TUBA1B+ H2AFZ+ HMGB2+ HIST1H4C+	CALR	3.01E-171	0.491805962	0.969	0.846	6.40E-167
TUBA1B+ H2AFZ+ HMGB2+ HIST1H4C+	ASF1B	0	0.490866593	0.449	0.015	0
TUBA1B+ H2AFZ+ HMGB2+ HIST1H4C+	MCM4	0	0.490603597	0.518	0.08	0

TUBA1B+ H2AFZ+ HMGB2+ HIST1H4C+	CKAP2	0	0.490414504	0.489	0.138	0
TUBA1B+ H2AFZ+ HMGB2+ HIST1H4C+	MDH1	5.48E-196	0.489522901	0.852	0.581	1.16E-191
TUBA1B+ H2AFZ+ HMGB2+ HIST1H4C+	MRPS34	6.70E-217	0.489477346	0.926	0.69	1.42E-212
TUBA1B+ H2AFZ+ HMGB2+ HIST1H4C+	LBR	2.72E-237	0.489202924	0.759	0.397	5.78E-233
TUBA1B+ H2AFZ+ HMGB2+ HIST1H4C+	PSMD14	8.47E-271	0.487747109	0.809	0.437	1.80E-266
TUBA1B+ H2AFZ+ HMGB2+ HIST1H4C+	TRMT112	6.38E-194	0.487167165	0.93	0.702	1.36E-189
TUBA1B+ H2AFZ+ HMGB2+ HIST1H4C+	PPIH	0	0.486187781	0.722	0.288	0
TUBA1B+ H2AFZ+ HMGB2+ HIST1H4C+	SPC25	0	0.485448821	0.383	0.005	0
TUBA1B+ H2AFZ+ HMGB2+ HIST1H4C+	PEBP1	1.48E-185	0.485244413	0.975	0.795	3.15E-181
TUBA1B+ H2AFZ+ HMGB2+ HIST1H4C+	CSE1L	0	0.482254881	0.617	0.2	0
TUBA1B+ H2AFZ+ HMGB2+ HIST1H4C+	PAFAH1B3	4.65E-237	0.482213848	0.831	0.47	9.90E-233
TUBA1B+ H2AFZ+ HMGB2+ HIST1H4C+	EIF6	1.05E-149	0.481862487	0.912	0.715	2.23E-145
TUBA1B+ H2AFZ+ HMGB2+ HIST1H4C+	PSMB3	3.33E-193	0.481047174	0.928	0.724	7.08E-189
TUBA1B+ H2AFZ+ HMGB2+ HIST1H4C+	SMS	1.38E-224	0.480871791	0.882	0.59	2.93E-220
TUBA1B+ H2AFZ+ HMGB2+ HIST1H4C+	HINT1	1.70E-224	0.480071659	0.995	0.906	3.61E-220
TUBA1B+ H2AFZ+ HMGB2+ HIST1H4C+	HNRNPA1	1.62E-165	0.480010411	0.998	0.884	3.44E-161
TUBA1B+ H2AFZ+ HMGB2+ HIST1H4C+	EBNA1BP2	4.91E-224	0.479863992	0.793	0.47	1.04E-219
TUBA1B+ H2AFZ+ HMGB2+ HIST1H4C+	CBX5	3.39E-296	0.478877551	0.702	0.304	7.20E-292
TUBA1B+ H2AFZ+ HMGB2+ HIST1H4C+	CCT4	5.50E-202	0.478115481	0.867	0.589	1.17E-197
TUBA1B+ H2AFZ+ HMGB2+ HIST1H4C+	AURKB	0	0.476578026	0.368	0.007	0
TUBA1B+ H2AFZ+ HMGB2+ HIST1H4C+	NAA38	3.92E-132	0.475925118	0.864	0.635	8.34E-128
TUBA1B+ H2AFZ+ HMGB2+ HIST1H4C+	ACTB	1.65E-255	0.474803181	1	0.983	3.51E-251

TUBA1B+ H2AFZ+ HMGB2+ HIST1H4C+	PARP1	8.91E-233	0.474172066	0.789	0.437	1.89E-228
TUBA1B+ H2AFZ+ HMGB2+ HIST1H4C+	SUB1	8.77E-207	0.473900474	0.979	0.868	1.87E-202
TUBA1B+ H2AFZ+ HMGB2+ HIST1H4C+	UQCRQ	1.11E-212	0.473481792	0.995	0.918	2.35E-208
TUBA1B+ H2AFZ+ HMGB2+ HIST1H4C+	ATP5MF	7.76E-215	0.473240621	0.984	0.877	1.65E-210
TUBA1B+ H2AFZ+ HMGB2+ HIST1H4C+	POLR2L	1.18E-196	0.471497373	0.981	0.852	2.51E-192
TUBA1B+ H2AFZ+ HMGB2+ HIST1H4C+	HADH	1.48E-223	0.467408381	0.834	0.5	3.14E-219
TUBA1B+ H2AFZ+ HMGB2+ HIST1H4C+	SAPCD2	0	0.466968259	0.499	0.094	0
TUBA1B+ H2AFZ+ HMGB2+ HIST1H4C+	IDI1	6.31E-180	0.466413266	0.72	0.384	1.34E-175
TUBA1B+ H2AFZ+ HMGB2+ HIST1H4C+	TUFM	4.48E-168	0.465962196	0.946	0.759	9.52E-164
TUBA1B+ H2AFZ+ HMGB2+ HIST1H4C+	NOP58	3.06E-242	0.464992539	0.78	0.423	6.51E-238
TUBA1B+ H2AFZ+ HMGB2+ HIST1H4C+	EIF3I	1.41E-175	0.464177767	0.917	0.693	3.00E-171
TUBA1B+ H2AFZ+ HMGB2+ HIST1H4C+	HSP90B1	1.26E-158	0.46393253	0.968	0.841	2.69E-154
TUBA1B+ H2AFZ+ HMGB2+ HIST1H4C+	SRP9	9.24E-198	0.462769467	0.961	0.788	1.97E-193
TUBA1B+ H2AFZ+ HMGB2+ HIST1H4C+	PAXX	4.52E-232	0.462143122	0.807	0.467	9.61E-228
TUBA1B+ H2AFZ+ HMGB2+ HIST1H4C+	MRPS15	2.12E-212	0.461581176	0.851	0.56	4.50E-208
TUBA1B+ H2AFZ+ HMGB2+ HIST1H4C+	CHEK1	0	0.459901264	0.54	0.085	0
TUBA1B+ H2AFZ+ HMGB2+ HIST1H4C+	CENPU	0	0.459894254	0.45	0.029	0
TUBA1B+ H2AFZ+ HMGB2+ HIST1H4C+	FDFT1	2.12E-149	0.458970272	0.815	0.536	4.51E-145
TUBA1B+ H2AFZ+ HMGB2+ HIST1H4C+	POLR3K	1.71E-297	0.458480425	0.666	0.269	3.63E-293
TUBA1B+ H2AFZ+ HMGB2+ HIST1H4C+	SLC25A3	3.57E-192	0.458323572	0.977	0.869	7.58E-188
TUBA1B+ H2AFZ+ HMGB2+ HIST1H4C+	PFN1	9.94E-180	0.458145043	0.996	0.933	2.11E-175
TUBA1B+ H2AFZ+ HMGB2+ HIST1H4C+	RPS21	1.85E-164	0.457286467	0.997	0.911	3.94E-160

TUBA1B+ H2AFZ+ HMGB2+ HIST1H4C+	SNRPA1	4.26E-235	0.457248927	0.728	0.382	9.07E-231
TUBA1B+ H2AFZ+ HMGB2+ HIST1H4C+	CALM3	8.89E-197	0.456538253	0.907	0.666	1.89E-192
TUBA1B+ H2AFZ+ HMGB2+ HIST1H4C+	DDT	6.30E-157	0.45639749	0.921	0.756	1.34E-152
TUBA1B+ H2AFZ+ HMGB2+ HIST1H4C+	TECR	2.28E-171	0.456168079	0.874	0.647	4.85E-167
TUBA1B+ H2AFZ+ HMGB2+ HIST1H4C+	SRSF9	4.49E-192	0.455782727	0.948	0.754	9.55E-188
TUBA1B+ H2AFZ+ HMGB2+ HIST1H4C+	RPSA	5.10E-176	0.455713541	0.999	0.885	1.09E-171
TUBA1B+ H2AFZ+ HMGB2+ HIST1H4C+	MTCH2	8.88E-205	0.455057882	0.89	0.622	1.89E-200
TUBA1B+ H2AFZ+ HMGB2+ HIST1H4C+	COX8A	6.37E-207	0.454213341	0.99	0.906	1.35E-202
TUBA1B+ H2AFZ+ HMGB2+ HIST1H4C+	ADRM1	3.74E-171	0.453484113	0.85	0.579	7.96E-167
TUBA1B+ H2AFZ+ HMGB2+ HIST1H4C+	VPS29	1.13E-192	0.453460622	0.907	0.672	2.41E-188
TUBA1B+ H2AFZ+ HMGB2+ HIST1H4C+	BCL2L12	0	0.453241386	0.608	0.168	0
TUBA1B+ H2AFZ+ HMGB2+ HIST1H4C+	PRC1	0	0.452452105	0.413	0.019	0
TUBA1B+ H2AFZ+ HMGB2+ HIST1H4C+	GCSH	4.40E-228	0.451513501	0.791	0.443	9.35E-224
TUBA1B+ H2AFZ+ HMGB2+ HIST1H4C+	ATP5MG	6.19E-181	0.45134893	0.994	0.92	1.32E-176
TUBA1B+ H2AFZ+ HMGB2+ HIST1H4C+	COX5A	4.89E-171	0.451147581	0.979	0.879	1.04E-166
TUBA1B+ H2AFZ+ HMGB2+ HIST1H4C+	FAM111B	0	0.450827973	0.386	0.021	0
TUBA1B+ H2AFZ+ HMGB2+ HIST1H4C+	XRCC6	7.86E-162	0.449605382	0.851	0.61	1.67E-157
TUBA1B+ H2AFZ+ HMGB2+ HIST1H4C+	ECT2	0	0.449580481	0.519	0.117	0
TUBA1B+ H2AFZ+ HMGB2+ HIST1H4C+	TFDP1	1.55E-264	0.449227336	0.69	0.312	3.30E-260
TUBA1B+ H2AFZ+ HMGB2+ HIST1H4C+	TRAP1	1.68E-232	0.449196387	0.754	0.408	3.58E-228
TUBA1B+ H2AFZ+ HMGB2+ HIST1H4C+	PSMB1	1.42E-185	0.448794344	0.965	0.814	3.01E-181
TUBA1B+ H2AFZ+ HMGB2+ HIST1H4C+	ATP5MD	8.42E-195	0.448216708	0.991	0.909	1.79E-190

TUBA1B+ H2AFZ+ HMGB2+ HIST1H4C+	HPRT1	1.09E-274	0.447936224	0.703	0.322	2.33E-270
TUBA1B+ H2AFZ+ HMGB2+ HIST1H4C+	PCBD1	9.07E-183	0.446764748	0.944	0.737	1.93E-178
TUBA1B+ H2AFZ+ HMGB2+ HIST1H4C+	MRPL17	5.95E-257	0.446638462	0.756	0.378	1.27E-252
TUBA1B+ H2AFZ+ HMGB2+ HIST1H4C+	CD74	3.93E-74	0.446427201	0.786	0.568	8.36E-70
TUBA1B+ H2AFZ+ HMGB2+ HIST1H4C+	TNFRSF12A	1.85E-175	0.445982298	0.66	0.326	3.94E-171
TUBA1B+ H2AFZ+ HMGB2+ HIST1H4C+	MPC2	1.16E-168	0.445975123	0.96	0.791	2.46E-164
TUBA1B+ H2AFZ+ HMGB2+ HIST1H4C+	MANF	3.41E-208	0.44593347	0.862	0.543	7.26E-204
TUBA1B+ H2AFZ+ HMGB2+ HIST1H4C+	ENSA	3.61E-211	0.445552227	0.925	0.685	7.67E-207
TUBA1B+ H2AFZ+ HMGB2+ HIST1H4C+	TIMM8B	9.41E-189	0.444935901	0.932	0.697	2.00E-184
TUBA1B+ H2AFZ+ HMGB2+ HIST1H4C+	MRPL12	1.51E-157	0.444832991	0.873	0.646	3.21E-153
TUBA1B+ H2AFZ+ HMGB2+ HIST1H4C+	LRRC59	2.30E-204	0.444041009	0.804	0.495	4.90E-200
TUBA1B+ H2AFZ+ HMGB2+ HIST1H4C+	EEF1B2	3.67E-168	0.443945387	0.993	0.876	7.80E-164
TUBA1B+ H2AFZ+ HMGB2+ HIST1H4C+	ATP5F1C	1.42E-176	0.443460696	0.971	0.807	3.03E-172
TUBA1B+ H2AFZ+ HMGB2+ HIST1H4C+	LYAR	0	0.442936331	0.577	0.196	0
TUBA1B+ H2AFZ+ HMGB2+ HIST1H4C+	SNRPD2	6.85E-179	0.442311937	0.984	0.834	1.46E-174
TUBA1B+ H2AFZ+ HMGB2+ HIST1H4C+	PPA1	1.06E-129	0.441482133	0.971	0.81	2.25E-125
TUBA1B+ H2AFZ+ HMGB2+ HIST1H4C+	MRPL11	4.61E-206	0.441408638	0.866	0.567	9.81E-202
TUBA1B+ H2AFZ+ HMGB2+ HIST1H4C+	SNRPD3	3.17E-197	0.441269129	0.876	0.619	6.73E-193
TUBA1B+ H2AFZ+ HMGB2+ HIST1H4C+	CHCHD3	1.74E-198	0.438959059	0.782	0.465	3.70E-194
TUBA1B+ H2AFZ+ HMGB2+ HIST1H4C+	ANP32A	4.03E-176	0.438200752	0.807	0.53	8.57E-172
TUBA1B+ H2AFZ+ HMGB2+ HIST1H4C+	H2AFY	1.27E-178	0.437965439	0.946	0.782	2.70E-174
TUBA1B+ H2AFZ+ HMGB2+ HIST1H4C+	SSB	6.97E-167	0.437923154	0.886	0.64	1.48E-162

TUBA1B+ H2AFZ+ HMGB2+ HIST1H4C+	LSM2	5.51E-199	0.4364694	0.863	0.552	1.17E-194
TUBA1B+ H2AFZ+ HMGB2+ HIST1H4C+	MRPL37	4.28E-242	0.435799514	0.789	0.433	9.09E-238
TUBA1B+ H2AFZ+ HMGB2+ HIST1H4C+	FBL	6.73E-171	0.435462364	0.908	0.649	1.43E-166
TUBA1B+ H2AFZ+ HMGB2+ HIST1H4C+	PRDX3	1.96E-177	0.435433588	0.871	0.602	4.17E-173
TUBA1B+ H2AFZ+ HMGB2+ HIST1H4C+	MYC	1.46E-111	0.434140472	0.707	0.443	3.11E-107
TUBA1B+ H2AFZ+ HMGB2+ HIST1H4C+	PSMC3	6.78E-198	0.43259627	0.841	0.555	1.44E-193
TUBA1B+ H2AFZ+ HMGB2+ HIST1H4C+	ITGB3BP	0	0.431980691	0.573	0.147	0
TUBA1B+ H2AFZ+ HMGB2+ HIST1H4C+	PSMA2	2.01E-167	0.43105709	0.955	0.748	4.28E-163
TUBA1B+ H2AFZ+ HMGB2+ HIST1H4C+	XRCC5	5.89E-182	0.429794731	0.892	0.634	1.25E-177
TUBA1B+ H2AFZ+ HMGB2+ HIST1H4C+	EXOSC8	1.71E-263	0.428569465	0.696	0.308	3.63E-259
TUBA1B+ H2AFZ+ HMGB2+ HIST1H4C+	EMP2	8.53E-191	0.42848706	0.882	0.581	1.81E-186
TUBA1B+ H2AFZ+ HMGB2+ HIST1H4C+	UQCRFS1	4.79E-154	0.427908177	0.946	0.758	1.02E-149
TUBA1B+ H2AFZ+ HMGB2+ HIST1H4C+	HDAC2	4.46E-184	0.427337152	0.846	0.569	9.49E-180
TUBA1B+ H2AFZ+ HMGB2+ HIST1H4C+	GTSE1	0	0.427286363	0.344	0.008	0
TUBA1B+ H2AFZ+ HMGB2+ HIST1H4C+	CEP55	0	0.426063192	0.396	0.012	0
TUBA1B+ H2AFZ+ HMGB2+ HIST1H4C+	CMSS1	1.65E-287	0.425564742	0.61	0.228	3.51E-283
TUBA1B+ H2AFZ+ HMGB2+ HIST1H4C+	GADD45GIP1	1.21E-149	0.425475583	0.86	0.668	2.57E-145
TUBA1B+ H2AFZ+ HMGB2+ HIST1H4C+	VRK1	0	0.425462762	0.507	0.084	0
TUBA1B+ H2AFZ+ HMGB2+ HIST1H4C+	POLR2E	9.98E-188	0.424803823	0.811	0.515	2.12E-183
TUBA1B+ H2AFZ+ HMGB2+ HIST1H4C+	EEF1D	1.69E-105	0.423668289	0.987	0.898	3.59E-101
TUBA1B+ H2AFZ+ HMGB2+ HIST1H4C+	ACOT7	8.85E-295	0.422270075	0.674	0.266	1.88E-290
TUBA1B+ H2AFZ+ HMGB2+ HIST1H4C+	CENPE	0	0.422170909	0.325	0.014	0

TUBA1B+ H2AFZ+ HMGB2+ HIST1H4C+	NDUFB9	1.51E-115	0.422110885	0.956	0.808	3.21E-111
TUBA1B+ H2AFZ+ HMGB2+ HIST1H4C+	RPL35	1.38E-222	0.421120736	0.999	0.94	2.93E-218
TUBA1B+ H2AFZ+ HMGB2+ HIST1H4C+	HACD3	1.28E-179	0.420686563	0.713	0.405	2.71E-175
TUBA1B+ H2AFZ+ HMGB2+ HIST1H4C+	CISD1	1.17E-171	0.419872001	0.86	0.586	2.49E-167
TUBA1B+ H2AFZ+ HMGB2+ HIST1H4C+	BST2	2.48E-115	0.418913229	0.446	0.203	5.27E-111
TUBA1B+ H2AFZ+ HMGB2+ HIST1H4C+	ANAPC11	1.34E-143	0.418586481	0.92	0.743	2.85E-139
TUBA1B+ H2AFZ+ HMGB2+ HIST1H4C+	CLSPN	0	0.418515886	0.377	0.012	0
TUBA1B+ H2AFZ+ HMGB2+ HIST1H4C+	DAZAP1	5.99E-207	0.417479143	0.775	0.454	1.27E-202
TUBA1B+ H2AFZ+ HMGB2+ HIST1H4C+	MIS18A	0	0.417209867	0.559	0.129	0
TUBA1B+ H2AFZ+ HMGB2+ HIST1H4C+	AURKAIP1	1.57E-177	0.417015211	0.963	0.825	3.33E-173
TUBA1B+ H2AFZ+ HMGB2+ HIST1H4C+	RPN2	2.03E-143	0.416426209	0.888	0.675	4.31E-139
TUBA1B+ H2AFZ+ HMGB2+ HIST1H4C+	MRPL18	1.68E-190	0.416035065	0.861	0.569	3.57E-186
TUBA1B+ H2AFZ+ HMGB2+ HIST1H4C+	MRPL20	9.03E-173	0.415401844	0.931	0.712	1.92E-168
TUBA1B+ H2AFZ+ HMGB2+ HIST1H4C+	DPM1	1.08E-168	0.414559311	0.755	0.455	2.30E-164
TUBA1B+ H2AFZ+ HMGB2+ HIST1H4C+	POMP	1.47E-150	0.414476061	0.981	0.863	3.13E-146
TUBA1B+ H2AFZ+ HMGB2+ HIST1H4C+	NDUFV1	1.34E-171	0.414461199	0.888	0.624	2.86E-167
TUBA1B+ H2AFZ+ HMGB2+ HIST1H4C+	CENPH	0	0.412523756	0.531	0.087	0
TUBA1B+ H2AFZ+ HMGB2+ HIST1H4C+	CDCA3	0	0.412289313	0.392	0.032	0
TUBA1B+ H2AFZ+ HMGB2+ HIST1H4C+	MTHFD2	9.75E-255	0.412119986	0.695	0.307	2.07E-250
TUBA1B+ H2AFZ+ HMGB2+ HIST1H4C+	PPA2	9.62E-172	0.411795648	0.87	0.603	2.05E-167
TUBA1B+ H2AFZ+ HMGB2+ HIST1H4C+	GRPEL1	3.38E-199	0.409582933	0.659	0.337	7.20E-195
TUBA1B+ H2AFZ+ HMGB2+ HIST1H4C+	P4HB	3.15E-118	0.409513232	0.941	0.81	6.70E-114

TUBA1B+ H2AFZ+ HMGB2+ HIST1H4C+	SSBP1	3.43E-153	0.408605679	0.938	0.726	7.29E-149
TUBA1B+ H2AFZ+ HMGB2+ HIST1H4C+	PDHA1	1.59E-189	0.408441067	0.828	0.524	3.38E-185
TUBA1B+ H2AFZ+ HMGB2+ HIST1H4C+	HNRNPDL	2.18E-150	0.408333812	0.895	0.665	4.63E-146
TUBA1B+ H2AFZ+ HMGB2+ HIST1H4C+	SUPT16H	1.19E-195	0.407027246	0.679	0.356	2.54E-191
TUBA1B+ H2AFZ+ HMGB2+ HIST1H4C+	NUTF2	5.17E-188	0.40665484	0.849	0.54	1.10E-183
TUBA1B+ H2AFZ+ HMGB2+ HIST1H4C+	PSMD7	2.37E-163	0.405996172	0.865	0.614	5.03E-159
TUBA1B+ H2AFZ+ HMGB2+ HIST1H4C+	SGO1	0	0.4052508	0.401	0.018	0
TUBA1B+ H2AFZ+ HMGB2+ HIST1H4C+	RUVBL2	6.47E-221	0.40519755	0.739	0.394	1.38E-216
TUBA1B+ H2AFZ+ HMGB2+ HIST1H4C+	ASRGL1	2.09E-238	0.405022992	0.736	0.339	4.45E-234
TUBA1B+ H2AFZ+ HMGB2+ HIST1H4C+	ATP5PF	1.19E-191	0.404972331	0.989	0.895	2.53E-187
TUBA1B+ H2AFZ+ HMGB2+ HIST1H4C+	PRDX4	1.14E-165	0.404876057	0.886	0.631	2.43E-161
TUBA1B+ H2AFZ+ HMGB2+ HIST1H4C+	PSMG1	5.02E-207	0.404550315	0.751	0.402	1.07E-202
TUBA1B+ H2AFZ+ HMGB2+ HIST1H4C+	RFC3	0	0.404005478	0.44	0.058	0
TUBA1B+ H2AFZ+ HMGB2+ HIST1H4C+	CENPA	0	0.403779384	0.309	0.007	0
TUBA1B+ H2AFZ+ HMGB2+ HIST1H4C+	ORC6	0	0.403060413	0.429	0.027	0
TUBA1B+ H2AFZ+ HMGB2+ HIST1H4C+	MAGOH	1.54E-184	0.402896749	0.844	0.55	3.27E-180
TUBA1B+ H2AFZ+ HMGB2+ HIST1H4C+	GLRX5	1.11E-158	0.402354241	0.806	0.554	2.36E-154
TUBA1B+ H2AFZ+ HMGB2+ HIST1H4C+	SDF2L1	5.54E-165	0.401817519	0.845	0.541	1.18E-160
TUBA1B+ H2AFZ+ HMGB2+ HIST1H4C+	MRPS12	1.70E-178	0.401750598	0.856	0.573	3.62E-174
TUBA1B+ H2AFZ+ HMGB2+ HIST1H4C+	RABL6	1.35E-147	0.400918216	0.808	0.546	2.87E-143
TUBA1B+ H2AFZ+ HMGB2+ HIST1H4C+	TACC3	0	0.400471599	0.396	0.025	0
TUBA1B+ H2AFZ+ HMGB2+ HIST1H4C+	PSMD8	1.01E-155	0.400316302	0.909	0.688	2.16E-151

TUBA1B+ H2AFZ+ HMGB2+ HIST1H4C+	PSMB5	9.21E-142	0.399328353	0.882	0.668	1.96E-137
TUBA1B+ H2AFZ+ HMGB2+ HIST1H4C+	CDC123	1.00E-196	0.399015527	0.753	0.432	2.13E-192
TUBA1B+ H2AFZ+ HMGB2+ HIST1H4C+	CARHSP1	7.99E-147	0.398798287	0.86	0.589	1.70E-142
TUBA1B+ H2AFZ+ HMGB2+ HIST1H4C+	TIMM10	2.17E-180	0.398499592	0.782	0.457	4.61E-176
TUBA1B+ H2AFZ+ HMGB2+ HIST1H4C+	GSPT1	5.83E-165	0.397928283	0.87	0.623	1.24E-160
TUBA1B+ H2AFZ+ HMGB2+ HIST1H4C+	NDUFA6	7.43E-145	0.397809459	0.914	0.733	1.58E-140
TUBA1B+ H2AFZ+ HMGB2+ HIST1H4C+	PLK1	0	0.397247208	0.294	0.013	0
TUBA1B+ H2AFZ+ HMGB2+ HIST1H4C+	GTF2A2	1.93E-161	0.397143933	0.842	0.583	4.11E-157
TUBA1B+ H2AFZ+ HMGB2+ HIST1H4C+	TBCA	4.65E-159	0.396758111	0.964	0.792	9.90E-155
TUBA1B+ H2AFZ+ HMGB2+ HIST1H4C+	UBE2V2	2.01E-156	0.396125243	0.801	0.512	4.28E-152
TUBA1B+ H2AFZ+ HMGB2+ HIST1H4C+	PPIB	1.19E-129	0.396111417	0.965	0.816	2.53E-125
TUBA1B+ H2AFZ+ HMGB2+ HIST1H4C+	HLA-DRB1	4.47E-111	0.395950488	0.558	0.295	9.50E-107
TUBA1B+ H2AFZ+ HMGB2+ HIST1H4C+	RPS2	5.49E-106	0.394199978	1	0.946	1.17E-101
TUBA1B+ H2AFZ+ HMGB2+ HIST1H4C+	PPIF	3.21E-267	0.394126538	0.694	0.3	6.82E-263
TUBA1B+ H2AFZ+ HMGB2+ HIST1H4C+	CD320	5.06E-168	0.393890686	0.788	0.49	1.08E-163
TUBA1B+ H2AFZ+ HMGB2+ HIST1H4C+	TOMM5	3.43E-211	0.393472698	0.759	0.396	7.30E-207
TUBA1B+ H2AFZ+ HMGB2+ HIST1H4C+	HNRNPK	2.35E-147	0.39334669	0.969	0.839	5.00E-143
TUBA1B+ H2AFZ+ HMGB2+ HIST1H4C+	RBM3	6.88E-134	0.39290897	0.938	0.755	1.46E-129
TUBA1B+ H2AFZ+ HMGB2+ HIST1H4C+	TOMM22	4.28E-138	0.392894282	0.85	0.63	9.10E-134
TUBA1B+ H2AFZ+ HMGB2+ HIST1H4C+	KIF22	5.68E-276	0.39144051	0.549	0.187	1.21E-271
TUBA1B+ H2AFZ+ HMGB2+ HIST1H4C+	MND1	0	0.390481847	0.388	0.02	0
TUBA1B+ H2AFZ+ HMGB2+ HIST1H4C+	CNN3	4.15E-188	0.389771603	0.779	0.438	8.82E-184

TUBA1B+ H2AFZ+ HMGB2+ HIST1H4C+	UBE2I	1.43E-151	0.389155667	0.906	0.663	3.03E-147
TUBA1B+ H2AFZ+ HMGB2+ HIST1H4C+	NDUFA12	5.18E-157	0.388711912	0.938	0.734	1.10E-152
TUBA1B+ H2AFZ+ HMGB2+ HIST1H4C+	STOML2	4.11E-143	0.388612365	0.899	0.664	8.75E-139
TUBA1B+ H2AFZ+ HMGB2+ HIST1H4C+	CFL1	1.08E-194	0.388452177	0.997	0.972	2.29E-190
TUBA1B+ H2AFZ+ HMGB2+ HIST1H4C+	NUF2	0	0.388062789	0.354	0.007	0
TUBA1B+ H2AFZ+ HMGB2+ HIST1H4C+	GLRX3	2.08E-178	0.387637177	0.825	0.52	4.42E-174
TUBA1B+ H2AFZ+ HMGB2+ HIST1H4C+	TROAP	0	0.387445857	0.416	0.033	0
TUBA1B+ H2AFZ+ HMGB2+ HIST1H4C+	PSMA1	1.40E-147	0.387148653	0.934	0.717	2.97E-143
TUBA1B+ H2AFZ+ HMGB2+ HIST1H4C+	CXCL1	5.12E-64	0.386692678	0.39	0.212	1.09E-59
TUBA1B+ H2AFZ+ HMGB2+ HIST1H4C+	EEF1E1	6.10E-215	0.386196667	0.748	0.388	1.30E-210
TUBA1B+ H2AFZ+ HMGB2+ HIST1H4C+	CMC2	3.64E-205	0.385949563	0.693	0.346	7.73E-201
TUBA1B+ H2AFZ+ HMGB2+ HIST1H4C+	SCD	2.49E-131	0.385720035	0.63	0.344	5.29E-127
TUBA1B+ H2AFZ+ HMGB2+ HIST1H4C+	BCL7C	2.58E-196	0.385175151	0.762	0.412	5.48E-192
TUBA1B+ H2AFZ+ HMGB2+ HIST1H4C+	MRPL16	3.61E-170	0.384287951	0.79	0.486	7.67E-166
TUBA1B+ H2AFZ+ HMGB2+ HIST1H4C+	OLA1	6.17E-153	0.384137958	0.862	0.591	1.31E-148
TUBA1B+ H2AFZ+ HMGB2+ HIST1H4C+	SF3B5	9.15E-143	0.383110232	0.934	0.756	1.95E-138
TUBA1B+ H2AFZ+ HMGB2+ HIST1H4C+	RBX1	4.79E-155	0.382946373	0.917	0.727	1.02E-150
TUBA1B+ H2AFZ+ HMGB2+ HIST1H4C+	ENY2	1.47E-117	0.382869684	0.893	0.715	3.13E-113
TUBA1B+ H2AFZ+ HMGB2+ HIST1H4C+	LYZ	2.83E-59	0.382726422	0.75	0.569	6.01E-55
TUBA1B+ H2AFZ+ HMGB2+ HIST1H4C+	MRFAP1	3.57E-121	0.382703932	0.824	0.604	7.60E-117
TUBA1B+ H2AFZ+ HMGB2+ HIST1H4C+	GPSM2	4.09E-201	0.382681428	0.588	0.255	8.71E-197
TUBA1B+ H2AFZ+ HMGB2+ HIST1H4C+	TFRC	1.95E-145	0.382109266	0.751	0.447	4.14E-141

TUBA1B+ H2AFZ+ HMGB2+ HIST1H4C+	FXYD5	8.96E-140	0.381935575	0.644	0.341	1.91E-135
TUBA1B+ H2AFZ+ HMGB2+ HIST1H4C+	LMNB2	0	0.381303036	0.539	0.149	0
TUBA1B+ H2AFZ+ HMGB2+ HIST1H4C+	TPM3	1.61E-155	0.381038589	0.957	0.778	3.42E-151
TUBA1B+ H2AFZ+ HMGB2+ HIST1H4C+	UQCC2	1.26E-165	0.380139993	0.775	0.485	2.68E-161
TUBA1B+ H2AFZ+ HMGB2+ HIST1H4C+	EIF5B	4.13E-154	0.38001521	0.916	0.691	8.79E-150
TUBA1B+ H2AFZ+ HMGB2+ HIST1H4C+	DLGAP5	0	0.379992776	0.312	0.006	0
TUBA1B+ H2AFZ+ HMGB2+ HIST1H4C+	MRPL36	4.57E-167	0.379689144	0.808	0.512	9.71E-163
TUBA1B+ H2AFZ+ HMGB2+ HIST1H4C+	DKC1	3.88E-184	0.379650843	0.725	0.4	8.25E-180
TUBA1B+ H2AFZ+ HMGB2+ HIST1H4C+	ATP5PB	9.67E-135	0.379263543	0.958	0.773	2.06E-130
TUBA1B+ H2AFZ+ HMGB2+ HIST1H4C+	DDX46	4.01E-154	0.378406292	0.847	0.58	8.53E-150
TUBA1B+ H2AFZ+ HMGB2+ HIST1H4C+	PSMD1	9.89E-192	0.377866756	0.723	0.388	2.10E-187
TUBA1B+ H2AFZ+ HMGB2+ HIST1H4C+	AK2	8.60E-177	0.377452034	0.852	0.552	1.83E-172
TUBA1B+ H2AFZ+ HMGB2+ HIST1H4C+	S100A11	2.78E-101	0.377008449	0.985	0.881	5.92E-97
TUBA1B+ H2AFZ+ HMGB2+ HIST1H4C+	RHOA	8.91E-149	0.376889036	0.966	0.835	1.89E-144
TUBA1B+ H2AFZ+ HMGB2+ HIST1H4C+	AHSA1	6.32E-147	0.376769275	0.736	0.467	1.34E-142
TUBA1B+ H2AFZ+ HMGB2+ HIST1H4C+	BRCA1	0	0.376395152	0.395	0.034	0
TUBA1B+ H2AFZ+ HMGB2+ HIST1H4C+	NUDT8	1.05E-215	0.375687477	0.617	0.272	2.23E-211
TUBA1B+ H2AFZ+ HMGB2+ HIST1H4C+	MCM5	0	0.375369128	0.437	0.099	0
TUBA1B+ H2AFZ+ HMGB2+ HIST1H4C+	SAC3D1	9.42E-301	0.375138245	0.544	0.175	2.00E-296
TUBA1B+ H2AFZ+ HMGB2+ HIST1H4C+	COPS9	5.84E-146	0.374962287	0.933	0.735	1.24E-141
TUBA1B+ H2AFZ+ HMGB2+ HIST1H4C+	MMAB	2.15E-228	0.374944691	0.74	0.358	4.58E-224
TUBA1B+ H2AFZ+ HMGB2+ HIST1H4C+	CCDC85B	3.96E-167	0.374533515	0.726	0.406	8.42E-163

TUBA1B+ H2AFZ+ HMGB2+ HIST1H4C+	CD81	9.78E-160	0.374104204	0.753	0.441	2.08E-155
TUBA1B+ H2AFZ+ HMGB2+ HIST1H4C+	NOP16	1.50E-191	0.374009941	0.684	0.349	3.19E-187
TUBA1B+ H2AFZ+ HMGB2+ HIST1H4C+	ACP1	3.48E-143	0.373876788	0.916	0.679	7.39E-139
TUBA1B+ H2AFZ+ HMGB2+ HIST1H4C+	TIMM17A	2.21E-180	0.373785124	0.775	0.454	4.70E-176
TUBA1B+ H2AFZ+ HMGB2+ HIST1H4C+	LSM6	1.90E-166	0.373306365	0.827	0.528	4.04E-162
TUBA1B+ H2AFZ+ HMGB2+ HIST1H4C+	METTL26	1.85E-159	0.372246651	0.862	0.586	3.94E-155
TUBA1B+ H2AFZ+ HMGB2+ HIST1H4C+	CALM1	1.15E-133	0.371617042	0.98	0.87	2.44E-129
TUBA1B+ H2AFZ+ HMGB2+ HIST1H4C+	CBX1	5.61E-211	0.371345658	0.642	0.292	1.19E-206
TUBA1B+ H2AFZ+ HMGB2+ HIST1H4C+	SUCLG1	2.99E-127	0.371124949	0.9	0.678	6.35E-123
TUBA1B+ H2AFZ+ HMGB2+ HIST1H4C+	C12orf75	4.63E-152	0.370743625	0.906	0.614	9.84E-148
TUBA1B+ H2AFZ+ HMGB2+ HIST1H4C+	NOLC1	1.18E-212	0.370663364	0.643	0.3	2.50E-208
TUBA1B+ H2AFZ+ HMGB2+ HIST1H4C+	LSM7	8.59E-137	0.370477481	0.956	0.787	1.83E-132
TUBA1B+ H2AFZ+ HMGB2+ HIST1H4C+	PTPMT1	2.48E-180	0.370113118	0.744	0.417	5.28E-176
TUBA1B+ H2AFZ+ HMGB2+ HIST1H4C+	UQCRC1	1.48E-128	0.369987242	0.944	0.767	3.15E-124
TUBA1B+ H2AFZ+ HMGB2+ HIST1H4C+	CYBA	5.11E-106	0.36977182	0.982	0.869	1.09E-101
TUBA1B+ H2AFZ+ HMGB2+ HIST1H4C+	SNRPC	7.93E-147	0.369742169	0.878	0.608	1.69E-142
TUBA1B+ H2AFZ+ HMGB2+ HIST1H4C+	MRPS26	2.86E-167	0.36962461	0.796	0.508	6.09E-163
TUBA1B+ H2AFZ+ HMGB2+ HIST1H4C+	SRM	8.08E-160	0.369244696	0.701	0.391	1.72E-155
TUBA1B+ H2AFZ+ HMGB2+ HIST1H4C+	TUBA4A	8.50E-186	0.369028792	0.718	0.369	1.81E-181
TUBA1B+ H2AFZ+ HMGB2+ HIST1H4C+	GPAA1	5.52E-159	0.368879272	0.763	0.464	1.17E-154
TUBA1B+ H2AFZ+ HMGB2+ HIST1H4C+	CALM2	1.73E-85	0.368819963	0.992	0.919	3.69E-81
TUBA1B+ H2AFZ+ HMGB2+ HIST1H4C+	SRPK1	3.79E-147	0.368728633	0.867	0.595	8.05E-143

TUBA1B+ H2AFZ+ HMGB2+ HIST1H4C+	GPI	1.83E-146	0.367978422	0.858	0.579	3.89E-142
TUBA1B+ H2AFZ+ HMGB2+ HIST1H4C+	POLE3	3.38E-256	0.367914488	0.662	0.277	7.19E-252
TUBA1B+ H2AFZ+ HMGB2+ HIST1H4C+	MGST2	1.91E-123	0.367832551	0.921	0.729	4.07E-119
TUBA1B+ H2AFZ+ HMGB2+ HIST1H4C+	EIF2S1	2.06E-186	0.367239674	0.688	0.362	4.37E-182
TUBA1B+ H2AFZ+ HMGB2+ HIST1H4C+	ISOC2	6.21E-151	0.367093512	0.824	0.54	1.32E-146
TUBA1B+ H2AFZ+ HMGB2+ HIST1H4C+	PDXK	6.20E-139	0.366761464	0.774	0.495	1.32E-134
TUBA1B+ H2AFZ+ HMGB2+ HIST1H4C+	PDIA3	8.79E-109	0.36611096	0.944	0.799	1.87E-104
TUBA1B+ H2AFZ+ HMGB2+ HIST1H4C+	THOC7	3.96E-150	0.365926489	0.911	0.679	8.41E-146
TUBA1B+ H2AFZ+ HMGB2+ HIST1H4C+	PUF60	3.33E-128	0.365518606	0.773	0.508	7.09E-124
TUBA1B+ H2AFZ+ HMGB2+ HIST1H4C+	PHF19	0	0.365095591	0.48	0.104	0
TUBA1B+ H2AFZ+ HMGB2+ HIST1H4C+	MRPS21	1.90E-162	0.364767478	0.784	0.442	4.04E-158
TUBA1B+ H2AFZ+ HMGB2+ HIST1H4C+	SMC1A	1.08E-217	0.364601085	0.634	0.283	2.30E-213
TUBA1B+ H2AFZ+ HMGB2+ HIST1H4C+	STIP1	3.50E-205	0.364298011	0.691	0.337	7.44E-201
TUBA1B+ H2AFZ+ HMGB2+ HIST1H4C+	EIF4A1	4.37E-110	0.363936683	0.871	0.658	9.30E-106
TUBA1B+ H2AFZ+ HMGB2+ HIST1H4C+	UBE2D2	1.82E-140	0.363680449	0.915	0.693	3.87E-136
TUBA1B+ H2AFZ+ HMGB2+ HIST1H4C+	NAA50	3.41E-202	0.36357354	0.737	0.384	7.24E-198
TUBA1B+ H2AFZ+ HMGB2+ HIST1H4C+	MRPL3	4.55E-150	0.362750978	0.805	0.535	9.67E-146
TUBA1B+ H2AFZ+ HMGB2+ HIST1H4C+	MRPL22	1.03E-170	0.36187591	0.813	0.502	2.19E-166
TUBA1B+ H2AFZ+ HMGB2+ HIST1H4C+	GMPS	1.29E-237	0.361807172	0.63	0.265	2.73E-233
TUBA1B+ H2AFZ+ HMGB2+ HIST1H4C+	ATG3	6.37E-163	0.361742232	0.8	0.496	1.35E-158
TUBA1B+ H2AFZ+ HMGB2+ HIST1H4C+	EI24	5.20E-136	0.361456894	0.911	0.676	1.11E-131
TUBA1B+ H2AFZ+ HMGB2+ HIST1H4C+	MTDH	8.15E-116	0.361300185	0.925	0.744	1.73E-111

TUBA1B+ H2AFZ+ HMGB2+ HIST1H4C+	PFDN2	2.98E-158	0.361021419	0.888	0.629	6.33E-154
TUBA1B+ H2AFZ+ HMGB2+ HIST1H4C+	ARHGAP11A	0	0.360740541	0.36	0.023	0
TUBA1B+ H2AFZ+ HMGB2+ HIST1H4C+	CDCA5	0	0.360710808	0.367	0.006	0
TUBA1B+ H2AFZ+ HMGB2+ HIST1H4C+	TSTA3	2.03E-109	0.360653257	0.886	0.651	4.32E-105
TUBA1B+ H2AFZ+ HMGB2+ HIST1H4C+	SUMO2	5.96E-102	0.360546086	0.972	0.852	1.27E-97
TUBA1B+ H2AFZ+ HMGB2+ HIST1H4C+	NDUFB6	2.81E-138	0.3602872	0.9	0.661	5.99E-134
TUBA1B+ H2AFZ+ HMGB2+ HIST1H4C+	TMEM141	2.44E-93	0.360151071	0.959	0.826	5.19E-89
TUBA1B+ H2AFZ+ HMGB2+ HIST1H4C+	NDUFAF8	8.14E-134	0.360112908	0.804	0.552	1.73E-129
TUBA1B+ H2AFZ+ HMGB2+ HIST1H4C+	UBE2L3	5.38E-130	0.360069159	0.87	0.66	1.14E-125
TUBA1B+ H2AFZ+ HMGB2+ HIST1H4C+	CISD2	7.88E-164	0.359892133	0.797	0.497	1.68E-159
TUBA1B+ H2AFZ+ HMGB2+ HIST1H4C+	COMMD4	4.09E-212	0.359753627	0.67	0.319	8.71E-208
TUBA1B+ H2AFZ+ HMGB2+ HIST1H4C+	SRRM1	1.75E-147	0.359686059	0.91	0.683	3.73E-143
TUBA1B+ H2AFZ+ HMGB2+ HIST1H4C+	SPCS1	2.28E-130	0.359469495	0.959	0.8	4.86E-126
TUBA1B+ H2AFZ+ HMGB2+ HIST1H4C+	MAGOHB	3.69E-252	0.359434959	0.635	0.26	7.86E-248
TUBA1B+ H2AFZ+ HMGB2+ HIST1H4C+	MRPS16	6.57E-159	0.359246788	0.867	0.584	1.40E-154
TUBA1B+ H2AFZ+ HMGB2+ HIST1H4C+	COX7B	9.31E-148	0.358913165	0.994	0.91	1.98E-143
TUBA1B+ H2AFZ+ HMGB2+ HIST1H4C+	SNU13	1.00E-104	0.358652875	0.887	0.695	2.13E-100
TUBA1B+ H2AFZ+ HMGB2+ HIST1H4C+	SAE1	1.60E-241	0.358650261	0.641	0.274	3.40E-237
TUBA1B+ H2AFZ+ HMGB2+ HIST1H4C+	GNL3	3.18E-151	0.35842121	0.792	0.499	6.76E-147
TUBA1B+ H2AFZ+ HMGB2+ HIST1H4C+	MRPL47	9.41E-162	0.358373142	0.78	0.481	2.00E-157
TUBA1B+ H2AFZ+ HMGB2+ HIST1H4C+	CTNNAL1	0	0.358005113	0.48	0.092	0
TUBA1B+ H2AFZ+ HMGB2+ HIST1H4C+	EPB41L2	3.41E-144	0.357630383	0.74	0.44	7.26E-140

TUBA1B+ H2AFZ+ HMGB2+ HIST1H4C+	ZDHHC12	1.54E-134	0.357483246	0.825	0.574	3.28E-130
TUBA1B+ H2AFZ+ HMGB2+ HIST1H4C+	MELK	0	0.357321855	0.374	0.014	0
TUBA1B+ H2AFZ+ HMGB2+ HIST1H4C+	LAPTM4B	1.23E-153	0.35655076	0.736	0.422	2.61E-149
TUBA1B+ H2AFZ+ HMGB2+ HIST1H4C+	CDCA8	0	0.356438555	0.366	0.028	0
TUBA1B+ H2AFZ+ HMGB2+ HIST1H4C+	SMC3	2.99E-168	0.356390682	0.742	0.425	6.36E-164
TUBA1B+ H2AFZ+ HMGB2+ HIST1H4C+	TUBG1	0	0.355944952	0.497	0.136	0
TUBA1B+ H2AFZ+ HMGB2+ HIST1H4C+	SFN	2.66E-171	0.355619116	0.871	0.565	5.65E-167
TUBA1B+ H2AFZ+ HMGB2+ HIST1H4C+	GSTM3	9.03E-231	0.355375469	0.524	0.183	1.92E-226
TUBA1B+ H2AFZ+ HMGB2+ HIST1H4C+	DYNLL1	2.47E-105	0.35528098	0.988	0.893	5.26E-101
TUBA1B+ H2AFZ+ HMGB2+ HIST1H4C+	AP2B1	6.77E-156	0.355183007	0.797	0.493	1.44E-151
TUBA1B+ H2AFZ+ HMGB2+ HIST1H4C+	NUDCD2	6.26E-152	0.354957106	0.754	0.47	1.33E-147
TUBA1B+ H2AFZ+ HMGB2+ HIST1H4C+	NUDT21	3.46E-177	0.354926113	0.783	0.46	7.35E-173
TUBA1B+ H2AFZ+ HMGB2+ HIST1H4C+	HAT1	1.05E-263	0.353700309	0.59	0.219	2.24E-259
TUBA1B+ H2AFZ+ HMGB2+ HIST1H4C+	FKBP4	1.34E-141	0.352866538	0.762	0.487	2.84E-137
TUBA1B+ H2AFZ+ HMGB2+ HIST1H4C+	NDUFV2	1.52E-125	0.352478159	0.925	0.748	3.22E-121
TUBA1B+ H2AFZ+ HMGB2+ HIST1H4C+	HSD17B10	2.17E-140	0.352453457	0.846	0.583	4.62E-136
TUBA1B+ H2AFZ+ HMGB2+ HIST1H4C+	ATP5PO	2.08E-124	0.352381199	0.979	0.832	4.42E-120
TUBA1B+ H2AFZ+ HMGB2+ HIST1H4C+	PSMD2	3.37E-176	0.351385507	0.758	0.433	7.17E-172
TUBA1B+ H2AFZ+ HMGB2+ HIST1H4C+	CDK2AP2	2.16E-127	0.351325993	0.784	0.486	4.58E-123
TUBA1B+ H2AFZ+ HMGB2+ HIST1H4C+	ODC1	9.46E-146	0.3501723	0.813	0.548	2.01E-141
TUBA1B+ H2AFZ+ HMGB2+ HIST1H4C+	NAA10	1.60E-151	0.350124118	0.827	0.544	3.41E-147
TUBA1B+ H2AFZ+ HMGB2+ HIST1H4C+	STRAP	1.15E-118	0.34966796	0.783	0.537	2.46E-114

TUBA1B+ H2AFZ+ HMGB2+ HIST1H4C+	NME4	4.10E-136	0.348799678	0.746	0.447	8.71E-132
TUBA1B+ H2AFZ+ HMGB2+ HIST1H4C+	RFC4	0	0.34752003	0.449	0.078	0
TUBA1B+ H2AFZ+ HMGB2+ HIST1H4C+	RHEB	6.97E-117	0.347338413	0.882	0.639	1.48E-112
TUBA1B+ H2AFZ+ HMGB2+ HIST1H4C+	RBM17	4.09E-159	0.347072559	0.832	0.533	8.69E-155
TUBA1B+ H2AFZ+ HMGB2+ HIST1H4C+	NDUFB3	8.03E-122	0.346922793	0.962	0.794	1.71E-117
TUBA1B+ H2AFZ+ HMGB2+ HIST1H4C+	EZH2	0	0.345857764	0.501	0.136	0
TUBA1B+ H2AFZ+ HMGB2+ HIST1H4C+	DHCR7	1.23E-191	0.345288129	0.53	0.216	2.62E-187
TUBA1B+ H2AFZ+ HMGB2+ HIST1H4C+	MDH2	7.24E-110	0.345224672	0.952	0.778	1.54E-105
TUBA1B+ H2AFZ+ HMGB2+ HIST1H4C+	DNPH1	4.18E-117	0.344906606	0.895	0.67	8.88E-113
TUBA1B+ H2AFZ+ HMGB2+ HIST1H4C+	RAD23A	2.18E-109	0.34431107	0.857	0.659	4.64E-105
TUBA1B+ H2AFZ+ HMGB2+ HIST1H4C+	RPL22L1	1.04E-47	0.34406505	0.785	0.6	2.20E-43
TUBA1B+ H2AFZ+ HMGB2+ HIST1H4C+	POLR2F	3.08E-116	0.34388832	0.834	0.624	6.54E-112
TUBA1B+ H2AFZ+ HMGB2+ HIST1H4C+	HNRNPA0	8.58E-131	0.343800997	0.91	0.682	1.82E-126
TUBA1B+ H2AFZ+ HMGB2+ HIST1H4C+	SDHB	2.76E-140	0.341675883	0.841	0.573	5.86E-136
TUBA1B+ H2AFZ+ HMGB2+ HIST1H4C+	HNRNPU	2.86E-120	0.341369192	0.946	0.792	6.09E-116
TUBA1B+ H2AFZ+ HMGB2+ HIST1H4C+	DMBT1	8.37E-89	0.340379881	0.361	0.164	1.78E-84
TUBA1B+ H2AFZ+ HMGB2+ HIST1H4C+	TFAM	2.10E-181	0.340179064	0.725	0.396	4.47E-177
TUBA1B+ H2AFZ+ HMGB2+ HIST1H4C+	SFPQ	5.85E-98	0.339876259	0.901	0.733	1.24E-93
TUBA1B+ H2AFZ+ HMGB2+ HIST1H4C+	UNG	3.55E-154	0.339843099	0.494	0.224	7.55E-150
TUBA1B+ H2AFZ+ HMGB2+ HIST1H4C+	SGO2	0	0.339521768	0.338	0.041	0
TUBA1B+ H2AFZ+ HMGB2+ HIST1H4C+	COQ2	4.99E-233	0.339239708	0.613	0.255	1.06E-228
TUBA1B+ H2AFZ+ HMGB2+ HIST1H4C+	MRPL4	6.61E-149	0.339110482	0.771	0.477	1.41E-144

TUBA1B+ H2AFZ+ HMGB2+ HIST1H4C+	RFC2	0	0.338579696	0.46	0.11	0
TUBA1B+ H2AFZ+ HMGB2+ HIST1H4C+	MRPL21	3.79E-149	0.338175546	0.826	0.548	8.06E-145
TUBA1B+ H2AFZ+ HMGB2+ HIST1H4C+	NEK2	0	0.337716146	0.316	0.007	0
TUBA1B+ H2AFZ+ HMGB2+ HIST1H4C+	RPS17	1.24E-94	0.33750757	0.996	0.888	2.63E-90
TUBA1B+ H2AFZ+ HMGB2+ HIST1H4C+	CCT3	7.64E-112	0.337356737	0.868	0.634	1.62E-107
TUBA1B+ H2AFZ+ HMGB2+ HIST1H4C+	PYCR1	3.10E-149	0.337172247	0.667	0.369	6.60E-145
TUBA1B+ H2AFZ+ HMGB2+ HIST1H4C+	EIF3K	1.10E-101	0.3370645	0.978	0.827	2.35E-97
TUBA1B+ H2AFZ+ HMGB2+ HIST1H4C+	CCND1	8.67E-61	0.336871438	0.708	0.51	1.84E-56
TUBA1B+ H2AFZ+ HMGB2+ HIST1H4C+	ATP5MPL	1.20E-129	0.336760154	0.986	0.883	2.56E-125
TUBA1B+ H2AFZ+ HMGB2+ HIST1H4C+	ILF3	4.74E-147	0.336737368	0.829	0.549	1.01E-142
TUBA1B+ H2AFZ+ HMGB2+ HIST1H4C+	MVD	7.43E-156	0.33671787	0.595	0.29	1.58E-151
TUBA1B+ H2AFZ+ HMGB2+ HIST1H4C+	RACGAP1	0	0.336435021	0.361	0.037	0
TUBA1B+ H2AFZ+ HMGB2+ HIST1H4C+	FH	3.69E-193	0.336214523	0.7	0.351	7.84E-189
TUBA1B+ H2AFZ+ HMGB2+ HIST1H4C+	ARPC1B	4.04E-91	0.335849056	0.908	0.694	8.59E-87
TUBA1B+ H2AFZ+ HMGB2+ HIST1H4C+	NEDD8	2.41E-116	0.335824144	0.943	0.777	5.12E-112
TUBA1B+ H2AFZ+ HMGB2+ HIST1H4C+	DCUN1D5	5.42E-200	0.33513797	0.619	0.282	1.15E-195
TUBA1B+ H2AFZ+ HMGB2+ HIST1H4C+	RPL26L1	4.71E-165	0.334447925	0.751	0.435	1.00E-160
TUBA1B+ H2AFZ+ HMGB2+ HIST1H4C+	DHCR24	7.02E-203	0.334177728	0.611	0.267	1.49E-198
TUBA1B+ H2AFZ+ HMGB2+ HIST1H4C+	LSM8	3.05E-124	0.333721768	0.882	0.628	6.48E-120
TUBA1B+ H2AFZ+ HMGB2+ HIST1H4C+	OAZ1	1.07E-137	0.333526122	0.99	0.941	2.29E-133
TUBA1B+ H2AFZ+ HMGB2+ HIST1H4C+	RUVBL1	3.24E-201	0.33319765	0.595	0.264	6.90E-197
TUBA1B+ H2AFZ+ HMGB2+ HIST1H4C+	GMDS	1.85E-112	0.333127773	0.913	0.688	3.94E-108

TUBA1B+ H2AFZ+ HMGB2+ HIST1H4C+	REXO2	2.98E-111	0.333084865	0.854	0.601	6.33E-107
TUBA1B+ H2AFZ+ HMGB2+ HIST1H4C+	SNRNP40	2.41E-212	0.332469628	0.655	0.298	5.13E-208
TUBA1B+ H2AFZ+ HMGB2+ HIST1H4C+	MAPRE1	2.54E-160	0.332223745	0.7	0.38	5.40E-156
TUBA1B+ H2AFZ+ HMGB2+ HIST1H4C+	POLR2G	1.42E-149	0.332140056	0.777	0.474	3.02E-145
TUBA1B+ H2AFZ+ HMGB2+ HIST1H4C+	HNRNPH3	1.06E-122	0.33074201	0.821	0.569	2.25E-118
TUBA1B+ H2AFZ+ HMGB2+ HIST1H4C+	TAF9	5.01E-136	0.330466805	0.782	0.515	1.07E-131
TUBA1B+ H2AFZ+ HMGB2+ HIST1H4C+	POLR2K	6.51E-129	0.330409631	0.862	0.622	1.38E-124
TUBA1B+ H2AFZ+ HMGB2+ HIST1H4C+	ATP6V0B	8.71E-117	0.330029402	0.914	0.728	1.85E-112
TUBA1B+ H2AFZ+ HMGB2+ HIST1H4C+	ACTG1	8.86E-78	0.32944297	0.997	0.966	1.88E-73
TUBA1B+ H2AFZ+ HMGB2+ HIST1H4C+	TMEM258	2.72E-127	0.329249772	0.982	0.857	5.79E-123
TUBA1B+ H2AFZ+ HMGB2+ HIST1H4C+	DNAJC8	9.98E-151	0.329225202	0.802	0.501	2.12E-146
TUBA1B+ H2AFZ+ HMGB2+ HIST1H4C+	NDUFB8	9.76E-114	0.328685518	0.926	0.75	2.08E-109
TUBA1B+ H2AFZ+ HMGB2+ HIST1H4C+	VDAC3	1.20E-121	0.328497052	0.821	0.553	2.56E-117
TUBA1B+ H2AFZ+ HMGB2+ HIST1H4C+	PPP1CA	4.39E-116	0.328308008	0.927	0.758	9.33E-112
TUBA1B+ H2AFZ+ HMGB2+ HIST1H4C+	DDX18	2.49E-134	0.328106431	0.868	0.609	5.30E-130
TUBA1B+ H2AFZ+ HMGB2+ HIST1H4C+	KIF4A	0	0.328065446	0.332	0.009	0
TUBA1B+ H2AFZ+ HMGB2+ HIST1H4C+	GOT2	2.17E-194	0.328006399	0.665	0.323	4.62E-190
TUBA1B+ H2AFZ+ HMGB2+ HIST1H4C+	KHDRBS1	1.58E-124	0.327996653	0.9	0.691	3.36E-120
TUBA1B+ H2AFZ+ HMGB2+ HIST1H4C+	TCOF1	8.18E-286	0.32775661	0.532	0.165	1.74E-281
TUBA1B+ H2AFZ+ HMGB2+ HIST1H4C+	FKBP3	1.48E-110	0.32689151	0.801	0.563	3.14E-106
TUBA1B+ H2AFZ+ HMGB2+ HIST1H4C+	CXCL2	4.28E-57	0.325151809	0.438	0.256	9.11E-53
TUBA1B+ H2AFZ+ HMGB2+ HIST1H4C+	NDUFA10	4.10E-120	0.325144808	0.891	0.641	8.72E-116

TUBA1B+ H2AFZ+ HMGB2+ HIST1H4C+	TMEM160	3.25E-148	0.324754561	0.807	0.51	6.92E-144
TUBA1B+ H2AFZ+ HMGB2+ HIST1H4C+	RPL8	1.42E-58	0.324118064	1	0.969	3.02E-54
TUBA1B+ H2AFZ+ HMGB2+ HIST1H4C+	SLC52A2	2.73E-111	0.324043525	0.709	0.445	5.81E-107
TUBA1B+ H2AFZ+ HMGB2+ HIST1H4C+	TRA2B	1.34E-137	0.323898028	0.837	0.577	2.86E-133
TUBA1B+ H2AFZ+ HMGB2+ HIST1H4C+	LAGE3	5.08E-153	0.323680516	0.731	0.417	1.08E-148
TUBA1B+ H2AFZ+ HMGB2+ HIST1H4C+	NDUFAF2	2.70E-153	0.323562035	0.765	0.446	5.74E-149
TUBA1B+ H2AFZ+ HMGB2+ HIST1H4C+	KIFC1	0	0.323538071	0.317	0.007	0
TUBA1B+ H2AFZ+ HMGB2+ HIST1H4C+	WDR1	1.27E-106	0.323377489	0.824	0.607	2.70E-102
TUBA1B+ H2AFZ+ HMGB2+ HIST1H4C+	MCM6	0	0.32184655	0.388	0.054	0
TUBA1B+ H2AFZ+ HMGB2+ HIST1H4C+	APRT	4.93E-92	0.321750423	0.952	0.779	1.05E-87
TUBA1B+ H2AFZ+ HMGB2+ HIST1H4C+	PHB2	1.51E-105	0.321175819	0.817	0.589	3.22E-101
TUBA1B+ H2AFZ+ HMGB2+ HIST1H4C+	PSMB6	2.18E-100	0.321048506	0.889	0.68	4.63E-96
TUBA1B+ H2AFZ+ HMGB2+ HIST1H4C+	CAMTA1	2.06E-123	0.321025867	0.926	0.703	4.38E-119
TUBA1B+ H2AFZ+ HMGB2+ HIST1H4C+	ETFB	1.29E-97	0.321004463	0.903	0.719	2.75E-93
TUBA1B+ H2AFZ+ HMGB2+ HIST1H4C+	HMGN3	5.97E-119	0.32098629	0.834	0.564	1.27E-114
TUBA1B+ H2AFZ+ HMGB2+ HIST1H4C+	MRTO4	1.11E-204	0.320727019	0.62	0.278	2.35E-200
TUBA1B+ H2AFZ+ HMGB2+ HIST1H4C+	CLDN2	4.43E-103	0.320520525	0.514	0.268	9.42E-99
TUBA1B+ H2AFZ+ HMGB2+ HIST1H4C+	MAZ	9.63E-138	0.31991722	0.762	0.471	2.05E-133
TUBA1B+ H2AFZ+ HMGB2+ HIST1H4C+	LYPLA1	7.46E-94	0.319706828	0.825	0.589	1.59E-89
TUBA1B+ H2AFZ+ HMGB2+ HIST1H4C+	CNBP	7.99E-102	0.319023774	0.939	0.768	1.70E-97
TUBA1B+ H2AFZ+ HMGB2+ HIST1H4C+	C19orf48	9.64E-161	0.317977649	0.662	0.339	2.05E-156
TUBA1B+ H2AFZ+ HMGB2+ HIST1H4C+	MORF4L2	3.64E-108	0.317769986	0.86	0.642	7.74E-104

TUBA1B+ H2AFZ+ HMGB2+ HIST1H4C+	BRCA2	0	0.317557655	0.324	0.025	0
TUBA1B+ H2AFZ+ HMGB2+ HIST1H4C+	TMED9	4.00E-97	0.317147919	0.912	0.74	8.51E-93
TUBA1B+ H2AFZ+ HMGB2+ HIST1H4C+	COPRS	2.18E-173	0.316930593	0.652	0.334	4.65E-169
TUBA1B+ H2AFZ+ HMGB2+ HIST1H4C+	PTMS	4.17E-57	0.314713255	0.826	0.621	8.87E-53
TUBA1B+ H2AFZ+ HMGB2+ HIST1H4C+	SNRPA	1.28E-200	0.31451629	0.672	0.317	2.73E-196
TUBA1B+ H2AFZ+ HMGB2+ HIST1H4C+	NPM3	6.95E-135	0.314492913	0.653	0.367	1.48E-130
TUBA1B+ H2AFZ+ HMGB2+ HIST1H4C+	FAM136A	3.43E-150	0.314418085	0.765	0.466	7.29E-146
TUBA1B+ H2AFZ+ HMGB2+ HIST1H4C+	PXMP2	1.80E-138	0.314405092	0.771	0.447	3.83E-134
TUBA1B+ H2AFZ+ HMGB2+ HIST1H4C+	ATAD5	0	0.314293824	0.372	0.043	0
TUBA1B+ H2AFZ+ HMGB2+ HIST1H4C+	RBM8A	6.12E-117	0.31388775	0.897	0.662	1.30E-112
TUBA1B+ H2AFZ+ HMGB2+ HIST1H4C+	PRPS2	1.05E-174	0.313596111	0.652	0.321	2.24E-170
TUBA1B+ H2AFZ+ HMGB2+ HIST1H4C+	SF3B6	4.74E-106	0.313567913	0.949	0.793	1.01E-101
TUBA1B+ H2AFZ+ HMGB2+ HIST1H4C+	MRPS25	2.71E-137	0.313282583	0.771	0.481	5.76E-133
TUBA1B+ H2AFZ+ HMGB2+ HIST1H4C+	NAP1L4	3.50E-153	0.312846376	0.728	0.413	7.45E-149
TUBA1B+ H2AFZ+ HMGB2+ HIST1H4C+	PKMYT1	0	0.312839997	0.333	0.01	0
TUBA1B+ H2AFZ+ HMGB2+ HIST1H4C+	BRIX1	5.10E-179	0.312820888	0.633	0.307	1.08E-174
TUBA1B+ H2AFZ+ HMGB2+ HIST1H4C+	NDUFS3	1.21E-116	0.312636675	0.872	0.631	2.57E-112
TUBA1B+ H2AFZ+ HMGB2+ HIST1H4C+	SMIM37	1.34E-137	0.312476186	0.802	0.503	2.84E-133
TUBA1B+ H2AFZ+ HMGB2+ HIST1H4C+	RPL27	3.43E-90	0.312397763	0.997	0.918	7.28E-86
TUBA1B+ H2AFZ+ HMGB2+ HIST1H4C+	PSMB7	2.23E-111	0.312112878	0.896	0.673	4.74E-107
TUBA1B+ H2AFZ+ HMGB2+ HIST1H4C+	TACSTD2	1.03E-76	0.311993014	0.383	0.189	2.20E-72
TUBA1B+ H2AFZ+ HMGB2+ HIST1H4C+	RNPS1	8.46E-124	0.311906308	0.874	0.618	1.80E-119

TUBA1B+ H2AFZ+ HMGB2+ HIST1H4C+	RPLP1	3.28E-89	0.311283159	1	0.983	6.98E-85
TUBA1B+ H2AFZ+ HMGB2+ HIST1H4C+	ACTL6A	2.23E-182	0.311255271	0.661	0.318	4.75E-178
TUBA1B+ H2AFZ+ HMGB2+ HIST1H4C+	JPT2	6.46E-199	0.310988491	0.65	0.297	1.37E-194
TUBA1B+ H2AFZ+ HMGB2+ HIST1H4C+	NUP37	2.08E-202	0.310981824	0.608	0.268	4.43E-198
TUBA1B+ H2AFZ+ HMGB2+ HIST1H4C+	PPP1R14B	2.63E-107	0.310666878	0.878	0.639	5.58E-103
TUBA1B+ H2AFZ+ HMGB2+ HIST1H4C+	PDAP1	6.30E-114	0.310360392	0.854	0.604	1.34E-109
TUBA1B+ H2AFZ+ HMGB2+ HIST1H4C+	IFRD2	3.19E-157	0.309344906	0.667	0.359	6.79E-153
TUBA1B+ H2AFZ+ HMGB2+ HIST1H4C+	EIF4A3	8.35E-131	0.309063628	0.655	0.383	1.78E-126
TUBA1B+ H2AFZ+ HMGB2+ HIST1H4C+	HDAC1	7.26E-116	0.308273595	0.872	0.639	1.54E-111
TUBA1B+ H2AFZ+ HMGB2+ HIST1H4C+	ALYREF	2.95E-227	0.307881885	0.536	0.204	6.27E-223
TUBA1B+ H2AFZ+ HMGB2+ HIST1H4C+	GAR1	1.12E-158	0.307707322	0.686	0.372	2.38E-154
TUBA1B+ H2AFZ+ HMGB2+ HIST1H4C+	CHAF1A	0	0.307474826	0.41	0.076	0
TUBA1B+ H2AFZ+ HMGB2+ HIST1H4C+	MRPS18C	4.46E-127	0.30740754	0.812	0.529	9.49E-123
TUBA1B+ H2AFZ+ HMGB2+ HIST1H4C+	EIF3J	2.92E-110	0.307355658	0.85	0.626	6.21E-106
TUBA1B+ H2AFZ+ HMGB2+ HIST1H4C+	PSMB9	1.31E-76	0.307071998	0.739	0.515	2.79E-72
TUBA1B+ H2AFZ+ HMGB2+ HIST1H4C+	BIN1	1.07E-118	0.307054163	0.671	0.391	2.27E-114
TUBA1B+ H2AFZ+ HMGB2+ HIST1H4C+	ATP5PD	2.50E-93	0.306910631	0.954	0.795	5.32E-89
TUBA1B+ H2AFZ+ HMGB2+ HIST1H4C+	BOP1	4.97E-164	0.30653311	0.536	0.245	1.06E-159
TUBA1B+ H2AFZ+ HMGB2+ HIST1H4C+	THOC3	5.99E-228	0.306342176	0.592	0.238	1.27E-223
TUBA1B+ H2AFZ+ HMGB2+ HIST1H4C+	NDUFB11	9.91E-105	0.306237663	0.959	0.815	2.11E-100
TUBA1B+ H2AFZ+ HMGB2+ HIST1H4C+	MRPL15	1.38E-111	0.306221655	0.76	0.5	2.93E-107
TUBA1B+ H2AFZ+ HMGB2+ HIST1H4C+	UBA2	4.10E-148	0.306049443	0.687	0.375	8.72E-144

TUBA1B+ H2AFZ+ HMGB2+ HIST1H4C+	NDUFB2	8.25E-94	0.305769635	0.983	0.849	1.75E-89
TUBA1B+ H2AFZ+ HMGB2+ HIST1H4C+	PRPF19	9.32E-176	0.303470435	0.694	0.358	1.98E-171
TUBA1B+ H2AFZ+ HMGB2+ HIST1H4C+	EIF4E	1.44E-123	0.303375161	0.791	0.515	3.05E-119
TUBA1B+ H2AFZ+ HMGB2+ HIST1H4C+	BZW1	3.38E-104	0.303042817	0.842	0.61	7.19E-100
TUBA1B+ H2AFZ+ HMGB2+ HIST1H4C+	PCMT1	3.43E-142	0.302670167	0.782	0.487	7.30E-138
TUBA1B+ H2AFZ+ HMGB2+ HIST1H4C+	HP1BP3	9.39E-93	0.302649118	0.799	0.554	2.00E-88
TUBA1B+ H2AFZ+ HMGB2+ HIST1H4C+	HSPB11	1.68E-139	0.302479554	0.661	0.355	3.58E-135
TUBA1B+ H2AFZ+ HMGB2+ HIST1H4C+	POLR2I	2.43E-109	0.302350676	0.884	0.671	5.17E-105
TUBA1B+ H2AFZ+ HMGB2+ HIST1H4C+	ATAD3A	7.48E-281	0.302192042	0.527	0.165	1.59E-276
TUBA1B+ H2AFZ+ HMGB2+ HIST1H4C+	CDC37	2.99E-109	0.302109428	0.876	0.663	6.37E-105
TUBA1B+ H2AFZ+ HMGB2+ HIST1H4C+	MRPS35	2.19E-110	0.301959112	0.802	0.555	4.66E-106
TUBA1B+ H2AFZ+ HMGB2+ HIST1H4C+	TMEM14B	4.70E-103	0.30195842	0.911	0.68	9.99E-99
TUBA1B+ H2AFZ+ HMGB2+ HIST1H4C+	F12	9.82E-196	0.301599013	0.577	0.246	2.09E-191
TUBA1B+ H2AFZ+ HMGB2+ HIST1H4C+	DRAP1	2.46E-100	0.301314165	0.878	0.636	5.22E-96
TUBA1B+ H2AFZ+ HMGB2+ HIST1H4C+	TAF15	3.73E-152	0.300615302	0.681	0.376	7.93E-148
TUBA1B+ H2AFZ+ HMGB2+ HIST1H4C+	KNSTRN	0	0.300546231	0.311	0.031	0
TUBA1B+ H2AFZ+ HMGB2+ HIST1H4C+	MRPL19	1.72E-135	0.300445442	0.73	0.434	3.66E-131
TUBA1B+ H2AFZ+ HMGB2+ HIST1H4C+	SIGMAR1	8.63E-203	0.300378504	0.595	0.252	1.84E-198
TUBA1B+ H2AFZ+ HMGB2+ HIST1H4C+	NDUFAF3	8.73E-111	0.30036872	0.85	0.579	1.86E-106
TUBA1B+ H2AFZ+ HMGB2+ HIST1H4C+	UHRF1	0	0.300117139	0.313	0.015	0
TUBA1B+ H2AFZ+ HMGB2+ HIST1H4C+	RNASEH2C	6.15E-145	0.300064384	0.696	0.389	1.31E-140
TUBA1B+ H2AFZ+ HMGB2+ HIST1H4C+	ANXA4	2.59E-55	0.299974857	0.9	0.739	5.50E-51

TUBA1B+ H2AFZ+ HMGB2+ HIST1H4C+	SRSF10	1.17E-125	0.299486917	0.84	0.582	2.49E-121
TUBA1B+ H2AFZ+ HMGB2+ HIST1H4C+	NUPR1	7.12E-143	0.299040939	0.866	0.545	1.51E-138
TUBA1B+ H2AFZ+ HMGB2+ HIST1H4C+	ANXA3	2.34E-92	0.298553444	0.748	0.49	4.99E-88
TUBA1B+ H2AFZ+ HMGB2+ HIST1H4C+	ELAVL1	2.04E-150	0.298524725	0.725	0.417	4.34E-146
TUBA1B+ H2AFZ+ HMGB2+ HIST1H4C+	PIH1D1	8.83E-144	0.29815305	0.764	0.468	1.88E-139
TUBA1B+ H2AFZ+ HMGB2+ HIST1H4C+	KNL1	0	0.297985147	0.31	0.015	0
TUBA1B+ H2AFZ+ HMGB2+ HIST1H4C+	RCC1	3.24E-260	0.297515707	0.528	0.174	6.89E-256
TUBA1B+ H2AFZ+ HMGB2+ HIST1H4C+	IMPA2	1.92E-130	0.296893363	0.727	0.433	4.09E-126
TUBA1B+ H2AFZ+ HMGB2+ HIST1H4C+	FOXM1	0	0.296208279	0.35	0.016	0
TUBA1B+ H2AFZ+ HMGB2+ HIST1H4C+	RPL4	1.41E-66	0.29612422	0.993	0.872	2.99E-62
TUBA1B+ H2AFZ+ HMGB2+ HIST1H4C+	COX7A2	3.39E-131	0.296123861	0.994	0.942	7.21E-127
TUBA1B+ H2AFZ+ HMGB2+ HIST1H4C+	TXNDC17	6.42E-105	0.295971065	0.949	0.777	1.37E-100
TUBA1B+ H2AFZ+ HMGB2+ HIST1H4C+	ARPC2	1.81E-83	0.295794179	0.972	0.869	3.85E-79
TUBA1B+ H2AFZ+ HMGB2+ HIST1H4C+	FANCI	0	0.295678446	0.348	0.023	0
TUBA1B+ H2AFZ+ HMGB2+ HIST1H4C+	IMPDH2	2.21E-86	0.295588062	0.876	0.627	4.69E-82
TUBA1B+ H2AFZ+ HMGB2+ HIST1H4C+	RCN2	1.32E-109	0.295475047	0.787	0.53	2.81E-105
TUBA1B+ H2AFZ+ HMGB2+ HIST1H4C+	PSMD13	3.10E-123	0.295474199	0.776	0.494	6.59E-119
TUBA1B+ H2AFZ+ HMGB2+ HIST1H4C+	NDUFA9	1.76E-98	0.295242272	0.8	0.554	3.73E-94
TUBA1B+ H2AFZ+ HMGB2+ HIST1H4C+	RPA2	3.77E-214	0.295192797	0.543	0.207	8.01E-210
TUBA1B+ H2AFZ+ HMGB2+ HIST1H4C+	MRPS23	2.06E-126	0.295099466	0.649	0.386	4.38E-122
TUBA1B+ H2AFZ+ HMGB2+ HIST1H4C+	ITGB1BP1	2.08E-159	0.294695927	0.666	0.339	4.43E-155
TUBA1B+ H2AFZ+ HMGB2+ HIST1H4C+	PSMC5	7.73E-102	0.293730623	0.786	0.553	1.64E-97

TUBA1B+ H2AFZ+ HMGB2+ HIST1H4C+	SUMO3	1.62E-120	0.29354372	0.81	0.537	3.45E-116
TUBA1B+ H2AFZ+ HMGB2+ HIST1H4C+	PFDN6	1.33E-117	0.29354002	0.801	0.525	2.82E-113
TUBA1B+ H2AFZ+ HMGB2+ HIST1H4C+	ANAPC15	1.42E-153	0.293182652	0.72	0.394	3.02E-149
TUBA1B+ H2AFZ+ HMGB2+ HIST1H4C+	MRPS2	2.56E-156	0.293006346	0.658	0.34	5.45E-152
TUBA1B+ H2AFZ+ HMGB2+ HIST1H4C+	COX6C	5.80E-91	0.293004381	0.992	0.931	1.23E-86
TUBA1B+ H2AFZ+ HMGB2+ HIST1H4C+	BZW2	3.55E-116	0.292895414	0.775	0.491	7.54E-112
TUBA1B+ H2AFZ+ HMGB2+ HIST1H4C+	NCAPD3	0	0.292624263	0.393	0.059	0
TUBA1B+ H2AFZ+ HMGB2+ HIST1H4C+	SRP72	2.99E-113	0.292290766	0.766	0.509	6.35E-109
TUBA1B+ H2AFZ+ HMGB2+ HIST1H4C+	VBP1	8.19E-134	0.29221108	0.73	0.428	1.74E-129
TUBA1B+ H2AFZ+ HMGB2+ HIST1H4C+	PPP2R1A	5.35E-93	0.292002158	0.867	0.646	1.14E-88
TUBA1B+ H2AFZ+ HMGB2+ HIST1H4C+	GHITM	9.22E-104	0.291522962	0.937	0.756	1.96E-99
TUBA1B+ H2AFZ+ HMGB2+ HIST1H4C+	MRPL50	5.21E-159	0.291416765	0.715	0.393	1.11E-154
TUBA1B+ H2AFZ+ HMGB2+ HIST1H4C+	PPP4C	7.31E-105	0.291305005	0.899	0.702	1.55E-100
TUBA1B+ H2AFZ+ HMGB2+ HIST1H4C+	PGD	5.82E-158	0.290983948	0.656	0.334	1.24E-153
TUBA1B+ H2AFZ+ HMGB2+ HIST1H4C+	THRAP3	4.36E-107	0.290345189	0.799	0.554	9.28E-103
TUBA1B+ H2AFZ+ HMGB2+ HIST1H4C+	RER1	3.03E-112	0.28988844	0.886	0.659	6.45E-108
TUBA1B+ H2AFZ+ HMGB2+ HIST1H4C+	TMEM70	5.64E-144	0.289858902	0.643	0.347	1.20E-139
TUBA1B+ H2AFZ+ HMGB2+ HIST1H4C+	CLPP	2.62E-144	0.289808908	0.717	0.415	5.58E-140
TUBA1B+ H2AFZ+ HMGB2+ HIST1H4C+	BID	8.07E-101	0.289628293	0.694	0.432	1.72E-96
TUBA1B+ H2AFZ+ HMGB2+ HIST1H4C+	MESD	6.65E-121	0.289231854	0.766	0.492	1.41E-116
TUBA1B+ H2AFZ+ HMGB2+ HIST1H4C+	SNRPB2	2.22E-112	0.289056719	0.884	0.648	4.72E-108
TUBA1B+ H2AFZ+ HMGB2+ HIST1H4C+	PARPBP	0	0.288958773	0.374	0.036	0

TUBA1B+ H2AFZ+ HMGB2+ HIST1H4C+	U2SURP	3.74E-125	0.28851466	0.822	0.547	7.96E-121
TUBA1B+ H2AFZ+ HMGB2+ HIST1H4C+	CDC6	0	0.288451334	0.324	0.022	0
TUBA1B+ H2AFZ+ HMGB2+ HIST1H4C+	LAP3	2.12E-125	0.288435006	0.648	0.364	4.52E-121
TUBA1B+ H2AFZ+ HMGB2+ HIST1H4C+	TMX2	1.32E-123	0.288078404	0.74	0.457	2.81E-119
TUBA1B+ H2AFZ+ HMGB2+ HIST1H4C+	RPL22	6.00E-81	0.287972472	0.997	0.893	1.27E-76
TUBA1B+ H2AFZ+ HMGB2+ HIST1H4C+	NSA2	2.88E-89	0.287947358	0.909	0.698	6.12E-85
TUBA1B+ H2AFZ+ HMGB2+ HIST1H4C+	SHMT2	1.09E-173	0.28793785	0.623	0.289	2.32E-169
TUBA1B+ H2AFZ+ HMGB2+ HIST1H4C+	NDC1	5.83E-301	0.287780519	0.442	0.109	1.24E-296
TUBA1B+ H2AFZ+ HMGB2+ HIST1H4C+	BARD1	7.34E-288	0.287747031	0.426	0.104	1.56E-283
TUBA1B+ H2AFZ+ HMGB2+ HIST1H4C+	E2F1	0	0.2874366	0.322	0.024	0
TUBA1B+ H2AFZ+ HMGB2+ HIST1H4C+	GNAS	3.76E-91	0.286903626	0.951	0.834	8.00E-87
TUBA1B+ H2AFZ+ HMGB2+ HIST1H4C+	IMMT	2.20E-144	0.286724682	0.717	0.398	4.68E-140
TUBA1B+ H2AFZ+ HMGB2+ HIST1H4C+	FAM96B	1.67E-108	0.28612171	0.917	0.692	3.56E-104
TUBA1B+ H2AFZ+ HMGB2+ HIST1H4C+	LACTB2	4.49E-142	0.286085602	0.639	0.332	9.54E-138
TUBA1B+ H2AFZ+ HMGB2+ HIST1H4C+	SNRNP70	1.30E-113	0.285929291	0.806	0.553	2.76E-109
TUBA1B+ H2AFZ+ HMGB2+ HIST1H4C+	DBF4	4.15E-214	0.285299829	0.493	0.177	8.82E-210
TUBA1B+ H2AFZ+ HMGB2+ HIST1H4C+	TXNDC12	2.23E-117	0.285126833	0.762	0.49	4.74E-113
TUBA1B+ H2AFZ+ HMGB2+ HIST1H4C+	MRPL52	1.32E-98	0.284201263	0.882	0.696	2.80E-94
TUBA1B+ H2AFZ+ HMGB2+ HIST1H4C+	INSIG1	5.78E-137	0.284063267	0.633	0.32	1.23E-132
TUBA1B+ H2AFZ+ HMGB2+ HIST1H4C+	NSD2	4.63E-213	0.284026396	0.55	0.212	9.85E-209
TUBA1B+ H2AFZ+ HMGB2+ HIST1H4C+	SMARCC1	3.69E-104	0.283816349	0.768	0.496	7.84E-100
TUBA1B+ H2AFZ+ HMGB2+ HIST1H4C+	NOL7	1.10E-98	0.283532441	0.832	0.59	2.33E-94

TUBA1B+ H2AFZ+ HMGB2+ HIST1H4C+	FASN	2.29E-175	0.282748771	0.542	0.233	4.87E-171
TUBA1B+ H2AFZ+ HMGB2+ HIST1H4C+	MTHFD1	9.86E-215	0.282720988	0.534	0.2	2.10E-210
TUBA1B+ H2AFZ+ HMGB2+ HIST1H4C+	PIN1	6.21E-131	0.28266201	0.783	0.493	1.32E-126
TUBA1B+ H2AFZ+ HMGB2+ HIST1H4C+	POC1A	0	0.282531009	0.362	0.021	0
TUBA1B+ H2AFZ+ HMGB2+ HIST1H4C+	POP7	8.65E-136	0.282432156	0.736	0.428	1.84E-131
TUBA1B+ H2AFZ+ HMGB2+ HIST1H4C+	C8orf59	3.52E-84	0.282243966	0.911	0.696	7.48E-80
TUBA1B+ H2AFZ+ HMGB2+ HIST1H4C+	LY6E	3.34E-32	0.281697105	0.316	0.202	7.10E-28
TUBA1B+ H2AFZ+ HMGB2+ HIST1H4C+	ANXA2	3.21E-76	0.281548666	0.975	0.885	6.82E-72
TUBA1B+ H2AFZ+ HMGB2+ HIST1H4C+	COX7C	3.47E-90	0.281397158	0.996	0.93	7.37E-86
TUBA1B+ H2AFZ+ HMGB2+ HIST1H4C+	UCHL3	2.45E-117	0.281318239	0.791	0.504	5.22E-113
TUBA1B+ H2AFZ+ HMGB2+ HIST1H4C+	HAUS1	3.15E-165	0.280500922	0.513	0.219	6.69E-161
TUBA1B+ H2AFZ+ HMGB2+ HIST1H4C+	NDUFA2	2.78E-95	0.279824566	0.941	0.777	5.91E-91
TUBA1B+ H2AFZ+ HMGB2+ HIST1H4C+	AKR7A2	1.39E-94	0.279760608	0.773	0.521	2.96E-90
TUBA1B+ H2AFZ+ HMGB2+ HIST1H4C+	RALY	6.22E-92	0.27975516	0.887	0.701	1.32E-87
TUBA1B+ H2AFZ+ HMGB2+ HIST1H4C+	NIFK	1.84E-113	0.27965414	0.713	0.427	3.91E-109
TUBA1B+ H2AFZ+ HMGB2+ HIST1H4C+	NAE1	2.70E-141	0.279232654	0.725	0.422	5.75E-137
TUBA1B+ H2AFZ+ HMGB2+ HIST1H4C+	RBMX	6.16E-80	0.279066124	0.867	0.641	1.31E-75
TUBA1B+ H2AFZ+ HMGB2+ HIST1H4C+	LIG1	0	0.278603725	0.403	0.07	0
TUBA1B+ H2AFZ+ HMGB2+ HIST1H4C+	KIF23	0	0.278353781	0.265	0.006	0
TUBA1B+ H2AFZ+ HMGB2+ HIST1H4C+	FUS	1.45E-95	0.27821947	0.915	0.727	3.09E-91
TUBA1B+ H2AFZ+ HMGB2+ HIST1H4C+	CENPS	0	0.278062364	0.443	0.104	0
TUBA1B+ H2AFZ+ HMGB2+ HIST1H4C+	SHMT1	1.87E-276	0.277791804	0.461	0.127	3.97E-272

TUBA1B+ H2AFZ+ HMGB2+ HIST1H4C+	COMT	2.84E-91	0.277773123	0.802	0.567	6.05E-87
TUBA1B+ H2AFZ+ HMGB2+ HIST1H4C+	DEPDC1	0	0.277631399	0.252	0.004	0
TUBA1B+ H2AFZ+ HMGB2+ HIST1H4C+	PTGES2	3.38E-108	0.276805173	0.782	0.509	7.18E-104
TUBA1B+ H2AFZ+ HMGB2+ HIST1H4C+	CDCA4	2.78E-284	0.276796173	0.377	0.083	5.91E-280
TUBA1B+ H2AFZ+ HMGB2+ HIST1H4C+	G3BP1	1.50E-122	0.276793293	0.784	0.496	3.20E-118
TUBA1B+ H2AFZ+ HMGB2+ HIST1H4C+	CSTF3	6.91E-198	0.276241124	0.588	0.244	1.47E-193
TUBA1B+ H2AFZ+ HMGB2+ HIST1H4C+	OXCT1	0	0.275599545	0.425	0.08	0
TUBA1B+ H2AFZ+ HMGB2+ HIST1H4C+	MYDGF	2.86E-88	0.275589305	0.903	0.714	6.08E-84
TUBA1B+ H2AFZ+ HMGB2+ HIST1H4C+	FAM96A	4.76E-113	0.274764917	0.787	0.516	1.01E-108
TUBA1B+ H2AFZ+ HMGB2+ HIST1H4C+	MTCH1	1.72E-98	0.2745919	0.848	0.619	3.67E-94
TUBA1B+ H2AFZ+ HMGB2+ HIST1H4C+	PLEK2	3.27E-155	0.274443325	0.673	0.356	6.95E-151
TUBA1B+ H2AFZ+ HMGB2+ HIST1H4C+	SLC38A5	5.91E-229	0.274253338	0.471	0.154	1.26E-224
TUBA1B+ H2AFZ+ HMGB2+ HIST1H4C+	ANLN	0	0.274194219	0.289	0.008	0
TUBA1B+ H2AFZ+ HMGB2+ HIST1H4C+	CTSH	1.26E-118	0.274151253	0.643	0.365	2.68E-114
TUBA1B+ H2AFZ+ HMGB2+ HIST1H4C+	YWHAE	2.58E-68	0.273858654	0.967	0.841	5.49E-64
TUBA1B+ H2AFZ+ HMGB2+ HIST1H4C+	CCDC124	1.89E-118	0.273743416	0.705	0.427	4.01E-114
TUBA1B+ H2AFZ+ HMGB2+ HIST1H4C+	GNPNAT1	4.70E-151	0.272336168	0.592	0.298	9.99E-147
TUBA1B+ H2AFZ+ HMGB2+ HIST1H4C+	BTG3	4.61E-166	0.272287536	0.586	0.267	9.81E-162
TUBA1B+ H2AFZ+ HMGB2+ HIST1H4C+	SRSF1	5.67E-126	0.27206304	0.771	0.485	1.21E-121
TUBA1B+ H2AFZ+ HMGB2+ HIST1H4C+	CANX	5.97E-77	0.272036635	0.933	0.755	1.27E-72
TUBA1B+ H2AFZ+ HMGB2+ HIST1H4C+	SPC24	0	0.271827679	0.293	0.008	0
TUBA1B+ H2AFZ+ HMGB2+ HIST1H4C+	AP2M1	1.61E-79	0.271666519	0.865	0.657	3.43E-75

TUBA1B+ H2AFZ+ HMGB2+ HIST1H4C+	RPS20	7.73E-70	0.271611175	0.998	0.936	1.64E-65
TUBA1B+ H2AFZ+ HMGB2+ HIST1H4C+	DENR	7.15E-115	0.27142729	0.738	0.457	1.52E-110
TUBA1B+ H2AFZ+ HMGB2+ HIST1H4C+	GOT1	8.13E-151	0.27103553	0.61	0.301	1.73E-146
TUBA1B+ H2AFZ+ HMGB2+ HIST1H4C+	BRK1	2.24E-89	0.270898289	0.943	0.772	4.77E-85
TUBA1B+ H2AFZ+ HMGB2+ HIST1H4C+	SPCS2	1.38E-76	0.270695911	0.916	0.752	2.94E-72
TUBA1B+ H2AFZ+ HMGB2+ HIST1H4C+	SERPINH1	8.62E-141	0.270652337	0.614	0.309	1.83E-136
TUBA1B+ H2AFZ+ HMGB2+ HIST1H4C+	PRPF40A	1.09E-100	0.270193114	0.839	0.595	2.31E-96
TUBA1B+ H2AFZ+ HMGB2+ HIST1H4C+	MCUB	4.99E-265	0.270099888	0.4	0.097	1.06E-260
TUBA1B+ H2AFZ+ HMGB2+ HIST1H4C+	DCXR	3.57E-97	0.269763954	0.815	0.574	7.59E-93
TUBA1B+ H2AFZ+ HMGB2+ HIST1H4C+	TOP1	7.94E-86	0.269452245	0.812	0.582	1.69E-81
TUBA1B+ H2AFZ+ HMGB2+ HIST1H4C+	DIAPH3	0	0.269098039	0.311	0.01	0
TUBA1B+ H2AFZ+ HMGB2+ HIST1H4C+	ESCO2	0	0.268996886	0.299	0.01	0
TUBA1B+ H2AFZ+ HMGB2+ HIST1H4C+	BCAP31	2.79E-90	0.268947071	0.903	0.719	5.94E-86
TUBA1B+ H2AFZ+ HMGB2+ HIST1H4C+	ZNF511	9.92E-103	0.267980923	0.792	0.532	2.11E-98
TUBA1B+ H2AFZ+ HMGB2+ HIST1H4C+	GDI2	8.72E-80	0.267644952	0.905	0.711	1.86E-75
TUBA1B+ H2AFZ+ HMGB2+ HIST1H4C+	PSMC4	8.44E-110	0.267602412	0.725	0.457	1.79E-105
TUBA1B+ H2AFZ+ HMGB2+ HIST1H4C+	HSBP1	1.19E-92	0.267311156	0.961	0.8	2.53E-88
TUBA1B+ H2AFZ+ HMGB2+ HIST1H4C+	HSD17B12	4.99E-70	0.267303252	0.828	0.623	1.06E-65
TUBA1B+ H2AFZ+ HMGB2+ HIST1H4C+	SERPINB6	1.38E-51	0.267212344	0.903	0.733	2.94E-47
TUBA1B+ H2AFZ+ HMGB2+ HIST1H4C+	HSPH1	2.95E-122	0.267047883	0.736	0.438	6.28E-118
TUBA1B+ H2AFZ+ HMGB2+ HIST1H4C+	CTSC	2.22E-155	0.266779032	0.69	0.342	4.72E-151
TUBA1B+ H2AFZ+ HMGB2+ HIST1H4C+	PSAT1	1.35E-221	0.266767405	0.322	0.073	2.88E-217

TUBA1B+ H2AFZ+ HMGB2+ HIST1H4C+	HPF1	4.50E-177	0.266254203	0.544	0.223	9.57E-173
TUBA1B+ H2AFZ+ HMGB2+ HIST1H4C+	MSMO1	4.05E-110	0.266193928	0.564	0.294	8.61E-106
TUBA1B+ H2AFZ+ HMGB2+ HIST1H4C+	POLR2H	7.80E-105	0.265994461	0.796	0.536	1.66E-100
TUBA1B+ H2AFZ+ HMGB2+ HIST1H4C+	MRPS7	1.54E-103	0.265660025	0.78	0.524	3.26E-99
TUBA1B+ H2AFZ+ HMGB2+ HIST1H4C+	DYNLT1	8.22E-95	0.265375601	0.948	0.781	1.75E-90
TUBA1B+ H2AFZ+ HMGB2+ HIST1H4C+	PGRMC1	1.27E-97	0.265018358	0.847	0.614	2.69E-93
TUBA1B+ H2AFZ+ HMGB2+ HIST1H4C+	KIF5B	6.37E-57	0.264961404	0.927	0.778	1.35E-52
TUBA1B+ H2AFZ+ HMGB2+ HIST1H4C+	MCRIP2	9.89E-80	0.264680649	0.853	0.643	2.10E-75
TUBA1B+ H2AFZ+ HMGB2+ HIST1H4C+	GTF3A	9.51E-81	0.264611956	0.862	0.599	2.02E-76
TUBA1B+ H2AFZ+ HMGB2+ HIST1H4C+	TRIM28	1.07E-111	0.26444982	0.707	0.426	2.27E-107
TUBA1B+ H2AFZ+ HMGB2+ HIST1H4C+	RTRAF	2.48E-69	0.264291268	0.913	0.757	5.27E-65
TUBA1B+ H2AFZ+ HMGB2+ HIST1H4C+	OIP5	0	0.264123625	0.334	0.01	0
TUBA1B+ H2AFZ+ HMGB2+ HIST1H4C+	IMP4	2.11E-110	0.263989529	0.708	0.426	4.50E-106
TUBA1B+ H2AFZ+ HMGB2+ HIST1H4C+	NDUFB1	1.04E-84	0.263649711	0.965	0.843	2.21E-80
TUBA1B+ H2AFZ+ HMGB2+ HIST1H4C+	EIF3A	1.81E-80	0.263568729	0.892	0.707	3.85E-76
TUBA1B+ H2AFZ+ HMGB2+ HIST1H4C+	DPY30	3.57E-92	0.263314541	0.856	0.632	7.58E-88
TUBA1B+ H2AFZ+ HMGB2+ HIST1H4C+	WDR43	6.30E-124	0.26290199	0.646	0.356	1.34E-119
TUBA1B+ H2AFZ+ HMGB2+ HIST1H4C+	TIMM50	8.60E-166	0.262731506	0.618	0.299	1.83E-161
TUBA1B+ H2AFZ+ HMGB2+ HIST1H4C+	GTPBP4	7.02E-128	0.262720391	0.605	0.324	1.49E-123
TUBA1B+ H2AFZ+ HMGB2+ HIST1H4C+	KIF11	0	0.262691816	0.273	0.011	0
TUBA1B+ H2AFZ+ HMGB2+ HIST1H4C+	ALDH7A1	3.98E-139	0.262608411	0.648	0.332	8.46E-135
TUBA1B+ H2AFZ+ HMGB2+ HIST1H4C+	ADAM15	1.06E-121	0.262396014	0.681	0.379	2.25E-117

TUBA1B+ H2AFZ+ HMGB2+ HIST1H4C+	RMI2	0	0.262341803	0.351	0.057	0
TUBA1B+ H2AFZ+ HMGB2+ HIST1H4C+	TSFM	4.64E-155	0.262307655	0.61	0.299	9.86E-151
TUBA1B+ H2AFZ+ HMGB2+ HIST1H4C+	SMAGP	8.40E-92	0.261897013	0.819	0.582	1.79E-87
TUBA1B+ H2AFZ+ HMGB2+ HIST1H4C+	EMC8	8.14E-156	0.261798386	0.609	0.296	1.73E-151
TUBA1B+ H2AFZ+ HMGB2+ HIST1H4C+	IDH3B	4.26E-107	0.261732015	0.74	0.46	9.06E-103
TUBA1B+ H2AFZ+ HMGB2+ HIST1H4C+	PSMD11	8.04E-110	0.261606775	0.73	0.472	1.71E-105
TUBA1B+ H2AFZ+ HMGB2+ HIST1H4C+	PITHD1	7.06E-148	0.260811245	0.646	0.328	1.50E-143
TUBA1B+ H2AFZ+ HMGB2+ HIST1H4C+	TMEM147	2.03E-76	0.260777331	0.875	0.663	4.32E-72
TUBA1B+ H2AFZ+ HMGB2+ HIST1H4C+	RHOBTB3	1.94E-107	0.260581993	0.768	0.476	4.12E-103
TUBA1B+ H2AFZ+ HMGB2+ HIST1H4C+	NOP10	3.29E-70	0.259991926	0.889	0.746	6.99E-66
TUBA1B+ H2AFZ+ HMGB2+ HIST1H4C+	AP1S1	5.25E-83	0.259929989	0.869	0.639	1.12E-78
TUBA1B+ H2AFZ+ HMGB2+ HIST1H4C+	METTL5	3.38E-92	0.259549252	0.829	0.585	7.19E-88
TUBA1B+ H2AFZ+ HMGB2+ HIST1H4C+	COX6A1	1.70E-92	0.259457493	0.993	0.929	3.61E-88
TUBA1B+ H2AFZ+ HMGB2+ HIST1H4C+	ARL2	9.75E-103	0.259049914	0.779	0.523	2.07E-98
TUBA1B+ H2AFZ+ HMGB2+ HIST1H4C+	BAX	1.20E-87	0.258999431	0.857	0.631	2.54E-83
TUBA1B+ H2AFZ+ HMGB2+ HIST1H4C+	ROMO1	1.23E-67	0.258970569	0.951	0.829	2.61E-63
TUBA1B+ H2AFZ+ HMGB2+ HIST1H4C+	EIF4G2	4.06E-64	0.258731613	0.953	0.812	8.64E-60
TUBA1B+ H2AFZ+ HMGB2+ HIST1H4C+	MSH6	4.17E-254	0.258542069	0.389	0.097	8.86E-250
TUBA1B+ H2AFZ+ HMGB2+ HIST1H4C+	UQCR10	3.05E-82	0.258082146	0.972	0.872	6.49E-78
TUBA1B+ H2AFZ+ HMGB2+ HIST1H4C+	NABP2	1.60E-159	0.257869468	0.631	0.305	3.41E-155
TUBA1B+ H2AFZ+ HMGB2+ HIST1H4C+	RPP25	1.00E-169	0.257599705	0.536	0.233	2.13E-165
TUBA1B+ H2AFZ+ HMGB2+ HIST1H4C+	CEP57	1.99E-118	0.257559556	0.655	0.359	4.23E-114

TUBA1B+ H2AFZ+ HMGB2+ HIST1H4C+	ENOPH1	2.98E-150	0.257206875	0.612	0.299	6.34E-146
TUBA1B+ H2AFZ+ HMGB2+ HIST1H4C+	POP5	2.32E-140	0.257116536	0.647	0.337	4.94E-136
TUBA1B+ H2AFZ+ HMGB2+ HIST1H4C+	CKAP4	9.36E-109	0.257051366	0.733	0.443	1.99E-104
TUBA1B+ H2AFZ+ HMGB2+ HIST1H4C+	WDR18	1.22E-129	0.257027859	0.621	0.335	2.58E-125
TUBA1B+ H2AFZ+ HMGB2+ HIST1H4C+	NCAPG	0	0.256826943	0.278	0.003	0
TUBA1B+ H2AFZ+ HMGB2+ HIST1H4C+	WDR77	6.14E-140	0.256810311	0.623	0.316	1.31E-135
TUBA1B+ H2AFZ+ HMGB2+ HIST1H4C+	HLA-DMA	4.25E-98	0.256773929	0.412	0.196	9.04E-94
TUBA1B+ H2AFZ+ HMGB2+ HIST1H4C+	AK6	6.36E-129	0.256599482	0.706	0.41	1.35E-124
TUBA1B+ H2AFZ+ HMGB2+ HIST1H4C+	RPL39L	0	0.256246341	0.302	0.035	0
TUBA1B+ H2AFZ+ HMGB2+ HIST1H4C+	THEM6	2.31E-151	0.256229332	0.489	0.211	4.91E-147
TUBA1B+ H2AFZ+ HMGB2+ HIST1H4C+	SLC2A4RG	5.66E-171	0.256166888	0.482	0.189	1.20E-166
TUBA1B+ H2AFZ+ HMGB2+ HIST1H4C+	PSMD12	8.48E-122	0.255268186	0.659	0.377	1.80E-117
TUBA1B+ H2AFZ+ HMGB2+ HIST1H4C+	EMC9	1.91E-210	0.254645057	0.471	0.161	4.05E-206
TUBA1B+ H2AFZ+ HMGB2+ HIST1H4C+	ABCE1	6.60E-133	0.254452258	0.652	0.35	1.40E-128
TUBA1B+ H2AFZ+ HMGB2+ HIST1H4C+	LCN2	3.56E-37	0.254415929	0.832	0.673	7.56E-33
TUBA1B+ H2AFZ+ HMGB2+ HIST1H4C+	NDUFAF4	3.96E-129	0.254223784	0.67	0.376	8.41E-125
TUBA1B+ H2AFZ+ HMGB2+ HIST1H4C+	PAK1IP1	2.24E-161	0.254098584	0.548	0.244	4.77E-157
TUBA1B+ H2AFZ+ HMGB2+ HIST1H4C+	VPS35	4.42E-86	0.253609571	0.844	0.602	9.40E-82
TUBA1B+ H2AFZ+ HMGB2+ HIST1H4C+	RPS7	7.36E-52	0.252866002	0.998	0.922	1.56E-47
TUBA1B+ H2AFZ+ HMGB2+ HIST1H4C+	UQCC3	6.93E-102	0.252765589	0.818	0.553	1.47E-97
TUBA1B+ H2AFZ+ HMGB2+ HIST1H4C+	RPS23	5.16E-44	0.252737929	0.999	0.932	1.10E-39
TUBA1B+ H2AFZ+ HMGB2+ HIST1H4C+	GNG5	4.42E-79	0.252721975	0.975	0.846	9.40E-75

TUBA1B+ H2AFZ+ HMGB2+ HIST1H4C+	CKAP5	1.01E-174	0.252720618	0.445	0.164	2.15E-170
TUBA1B+ H2AFZ+ HMGB2+ HIST1H4C+	ABRACL	3.00E-85	0.251984618	0.89	0.674	6.38E-81
TUBA1B+ H2AFZ+ HMGB2+ HIST1H4C+	TRIP13	0	0.251832583	0.323	0.017	0
TUBA1B+ H2AFZ+ HMGB2+ HIST1H4C+	CLTA	9.22E-95	0.251488092	0.968	0.834	1.96E-90
TUBA1B+ H2AFZ+ HMGB2+ HIST1H4C+	NDC80	0	0.251388822	0.26	0.006	0
TUBA1B+ H2AFZ+ HMGB2+ HIST1H4C+	NT5DC2	4.21E-304	0.250907678	0.373	0.075	8.94E-300
TUBA1B+ H2AFZ+ HMGB2+ HIST1H4C+	COX11	5.06E-112	0.250875285	0.656	0.384	1.08E-107
TUBA1B+ H2AFZ+ HMGB2+ HIST1H4C+	XRN2	1.37E-71	0.250385494	0.746	0.5	2.92E-67
TUBA1B+ H2AFZ+ HMGB2+ HIST1H4C+	ARL6IP4	1.36E-80	0.250365534	0.954	0.795	2.89E-76
