# Supplementary material for: Low expression of the dynamic network markers FOS/JUN in pre-deteriorated epithelial cells is associated with the progression of colorectal adenoma to carcinoma
Source: J Transl Med. 2023 Jan 25;21:45. doi: 10.1186/s12967-023-03890-5 (PMC9875500; doi:10.1186/s12967-023-03890-5)
Supplement: Supplementary file 10 — Additional file 10: Table S3. Signature genes for 6 clusters of cells on pseudotime trajectory. [file 12967_2023_3890_MOESM10_ESM.pdf]

Table S3. Signature genes for 6 clusters of cells on pseudotime trajectory

Cluster 1					
**Gene symbol**	**p_val**	**avg_log2FC**	**pct.1**	**pct.2**	**p_val_adj**
REG1A	7.86E-167	2.419292682	0.648	0.21	1.48E-162
LEFTY1	5.13E-248	1.497439669	0.973	0.488	9.68E-244
SMOC2	1.54E-220	1.066439091	0.558	0.087	2.91E-216
PCCA	1.00E-153	1.013654755	0.911	0.511	1.89E-149
ASCL2	7.41E-138	1.004359614	0.974	0.848	1.40E-133
BTG2	7.10E-160	0.999755116	0.959	0.751	1.34E-155
ID3	5.53E-108	0.996205546	0.946	0.771	1.04E-103
TXNIP	2.07E-177	0.974776113	0.918	0.565	3.91E-173
RGMB	3.70E-191	0.946553063	0.809	0.374	6.99E-187
APCDD1	2.81E-172	0.939406083	0.834	0.311	5.30E-168
ZFP36L2	1.98E-147	0.907937192	0.993	0.927	3.74E-143
NOP53	1.86E-247	0.892243333	0.995	0.952	3.52E-243
DPEP1	1.70E-138	0.883493721	0.888	0.502	3.21E-134
RPL26	8.69E-308	0.867393205	0.999	0.995	1.64E-303
HES1	1.75E-63	0.822428522	0.902	0.807	3.31E-59
RPL17	2.82E-191	0.779772281	0.995	0.942	5.33E-187
NKD1	7.65E-131	0.77472181	0.828	0.394	1.44E-126
IGKC	3.63E-101	0.771728191	0.836	0.615	6.86E-97
RPS5	8.42E-246	0.761343866	0.999	0.986	1.59E-241
RPS4X	2.12E-302	0.759381719	0.999	0.993	4.01E-298
GAS6	4.51E-147	0.753536362	0.908	0.673	8.52E-143
FAM3D	2.08E-133	0.744901561	0.985	0.886	3.94E-129
MUC12	7.11E-105	0.744721832	0.875	0.61	1.34E-100
REPIN1	3.05E-147	0.73668656	0.952	0.783	5.75E-143
SELENBP1	7.28E-105	0.728245238	0.958	0.784	1.37E-100
ITPR2	1.98E-119	0.722986538	0.76	0.404	3.73E-115
PRDX5	6.40E-157	0.706567859	0.998	0.978	1.21E-152
ZKSCAN1	1.61E-134	0.704629632	0.958	0.796	3.05E-130
CD9	3.58E-142	0.691196321	0.996	0.953	6.75E-138
ID4	6.88E-117	0.687749991	0.731	0.321	1.30E-112
COMMD6	3.97E-183	0.685956641	0.998	0.957	7.50E-179
JUN	8.78E-124	0.683704172	0.996	0.963	1.66E-119
RPL13	0	0.65737743	0.999	1	0
EIF3L	5.52E-155	0.654345459	0.986	0.906	1.04E-150
C6orf48	3.21E-121	0.649912597	0.975	0.857	6.07E-117
RPL36A	1.65E-173	0.648825152	0.999	0.987	3.12E-169
C10orf99	5.17E-99	0.639171711	0.943	0.703	9.76E-95
LGR5	2.31E-126	0.629391584	0.673	0.29	4.37E-122
RPL34	5.59E-282	0.626288427	0.999	0.997	1.05E-277
PNRC1	3.51E-123	0.607759095	0.933	0.732	6.63E-119

FOSB	5.68E-82	0.605737161	0.884	0.702	1.07E-77
RPS27	4.43E-275	0.604968207	0.999	0.999	8.36E-271
RPS6	6.31E-272	0.60369293	0.999	0.996	1.19E-267
CDCA7	1.97E-90	0.598940596	0.849	0.631	3.72E-86
IGHA1	2.86E-81	0.593021702	0.752	0.52	5.41E-77
PRR15	1.17E-94	0.590081275	0.889	0.689	2.21E-90
DEFA5	7.90E-40	0.58982074	0.393	0.213	1.49E-35
RPL21	9.40E-247	0.585873316	0.999	0.996	1.78E-242
SMAD9	5.92E-113	0.574973607	0.662	0.304	1.12E-108
IMPDH2	3.10E-109	0.561709531	0.98	0.873	5.86E-105
RPS12	2.92E-242	0.559749851	0.999	0.998	5.51E-238
SLC25A6	7.34E-175	0.556019437	0.998	0.984	1.39E-170
QTRT1	1.46E-105	0.555612973	0.94	0.735	2.76E-101
RNF43	1.14E-104	0.553991635	0.967	0.836	2.16E-100
MT-CO3	1.90E-107	0.550384275	0.999	0.991	3.59E-103
FOS	7.31E-54	0.549953389	0.974	0.927	1.38E-49
EPHB3	2.24E-92	0.542094757	0.807	0.497	4.23E-88
ZNF703	1.06E-90	0.538279065	0.883	0.693	2.01E-86
FERMT1	3.58E-91	0.535537125	0.953	0.678	6.77E-87
EIF3D	2.25E-101	0.531994909	0.976	0.895	4.26E-97
RPL10A	5.49E-234	0.529499584	0.999	0.996	1.04E-229
ZBTB38	5.07E-81	0.525504291	0.82	0.628	9.58E-77
MUC3A	4.35E-85	0.524550738	0.827	0.63	8.22E-81
N4BP2L2	6.47E-98	0.522414165	0.964	0.859	1.22E-93
RPS18	2.49E-241	0.517778726	1	0.999	4.69E-237
EEF2	2.45E-159	0.51767407	0.997	0.983	4.62E-155
EEF1A1	9.74E-224	0.517420249	0.999	0.997	1.84E-219
KIAA1324	5.03E-91	0.515808186	0.849	0.58	9.50E-87
AXIN2	1.94E-80	0.508927233	0.905	0.714	3.66E-76
TOMM7	7.97E-131	0.508409396	0.995	0.954	1.51E-126
TCEA3	2.34E-115	0.505820907	0.771	0.387	4.42E-111
RPL10	5.27E-226	0.498158195	1	0.998	9.96E-222
TMEM230	2.43E-95	0.497654349	0.934	0.789	4.60E-91
RPL9	3.32E-211	0.495255805	0.998	0.993	6.28E-207
IGBP1	8.17E-93	0.493059665	0.909	0.785	1.54E-88
AC020916.1	3.17E-52	0.490399292	0.658	0.445	5.98E-48
ALDH1A1	2.79E-85	0.490048645	0.764	0.431	5.26E-81
RUBCNL	7.13E-102	0.489200145	0.669	0.332	1.35E-97
PROX1	4.90E-94	0.483884397	0.652	0.306	9.24E-90
MT-ND2	2.55E-93	0.483400461	1	0.985	4.82E-89
RPL15	2.47E-213	0.479818094	1	0.996	4.66E-209
SARAF	8.37E-90	0.47925406	0.961	0.871	1.58E-85
RPS15A	3.72E-229	0.478990255	1	0.995	7.02E-225

RPS9	1.02E-214	0.47741398	0.998	0.996	1.92E-210
TPT1	6.59E-147	0.475262941	1	0.997	1.24E-142
TNRC6B	7.22E-88	0.471775187	0.791	0.485	1.36E-83
RACK1	3.22E-180	0.469203189	0.998	0.994	6.09E-176
MUC5B	3.31E-93	0.46759769	0.622	0.279	6.26E-89
RPL3	4.73E-186	0.465800521	1	0.997	8.93E-182
RAB11FIP1	2.81E-64	0.465012989	0.884	0.74	5.31E-60
MT-ND3	1.31E-70	0.459167989	0.998	0.984	2.47E-66
NPDC1	1.34E-74	0.456801329	0.973	0.906	2.53E-70
ITGA6	4.04E-63	0.451883977	0.977	0.9	7.62E-59
CYP4X1	1.60E-95	0.450479255	0.616	0.281	3.02E-91
NDUFA5	8.53E-72	0.447377665	0.937	0.853	1.61E-67
RPL41	1.19E-238	0.446239355	1	0.999	2.26E-234
ID2	3.10E-40	0.442173074	0.908	0.801	5.84E-36
RPS3	3.71E-176	0.438968485	0.999	0.997	7.01E-172
ALDH1B1	1.40E-65	0.438689881	0.777	0.577	2.63E-61
CCNI	1.20E-87	0.43712372	0.994	0.962	2.26E-83
VPS51	4.91E-75	0.433876436	0.857	0.724	9.27E-71
HNMT	2.32E-72	0.431652511	0.799	0.619	4.37E-68
ESD	1.55E-69	0.43060443	0.964	0.859	2.93E-65
AGR3	2.29E-56	0.426394617	0.972	0.833	4.33E-52
VAMP2	2.48E-79	0.422380993	0.764	0.53	4.68E-75
MLXIP	1.01E-63	0.421807496	0.926	0.814	1.90E-59
THRA	3.90E-70	0.421706392	0.718	0.498	7.36E-66
CIRBP	5.51E-76	0.421516671	0.98	0.928	1.04E-71
KMT2E	2.45E-63	0.421444457	0.92	0.825	4.63E-59
RPL36	1.66E-180	0.420355847	0.999	0.998	3.14E-176
RPL31	6.49E-171	0.419048151	0.998	0.997	1.23E-166
MT-ATP6	8.99E-69	0.416806607	0.998	0.982	1.70E-64
MT-ND1	1.15E-76	0.416658725	0.999	0.981	2.17E-72
GTF2I	2.25E-58	0.415476184	0.937	0.84	4.24E-54
EIF3F	3.87E-79	0.409936982	0.986	0.928	7.31E-75
SMIM19	3.21E-74	0.409071312	0.799	0.595	6.06E-70
RPS8	8.48E-164	0.40603022	0.999	0.995	1.60E-159
BCL11A	1.61E-52	0.40560868	0.726	0.543	3.03E-48
RPS13	1.37E-144	0.405058569	0.999	0.991	2.58E-140
MT-ND4	3.81E-76	0.404472081	0.999	0.99	7.19E-72
MT-CYB	3.10E-60	0.403859468	1	0.983	5.85E-56
LAMTOR4	1.22E-67	0.403551311	0.979	0.927	2.31E-63
PDGFA	7.94E-49	0.403422829	0.494	0.279	1.50E-44
NIPSNAP2	2.97E-66	0.403376413	0.863	0.705	5.61E-62
ATP5MC2	9.96E-104	0.402292497	0.998	0.977	1.88E-99
BRI3	1.94E-64	0.401498977	0.96	0.859	3.66E-60

KRTCAP3	3.42E-63	0.400712406	0.947	0.825	6.45E-59
FARP1	6.89E-102	0.400344221	0.559	0.22	1.30E-97
YPEL3	1.42E-92	0.398383527	0.587	0.272	2.67E-88
L1TD1	4.32E-60	0.397168549	0.667	0.35	8.15E-56
TSTD1	4.43E-62	0.393090034	0.972	0.88	8.37E-58
ICA1	1.30E-69	0.392651062	0.759	0.558	2.46E-65
RPS16	1.96E-141	0.392555381	0.999	0.995	3.70E-137
RPLP0	7.44E-144	0.392115984	0.999	0.995	1.40E-139
GLUL	3.61E-50	0.391282367	0.887	0.797	6.81E-46
CDHR1	7.15E-77	0.390875156	0.584	0.314	1.35E-72
QARS	1.22E-62	0.389301928	0.924	0.831	2.31E-58
EFNA3	8.48E-74	0.38831848	0.528	0.256	1.60E-69
RPL14	1.04E-164	0.387221549	0.999	0.995	1.96E-160
AKAP9	8.44E-64	0.386726308	0.934	0.829	1.59E-59
RPL11	2.33E-191	0.386611839	0.999	0.998	4.40E-187
HOXA9	5.85E-45	0.385639809	0.737	0.595	1.10E-40
RPL18	1.01E-161	0.385504274	0.998	0.996	1.91E-157
RPL12	6.44E-143	0.384219839	1	0.997	1.22E-138
MRPS33	7.92E-61	0.382310128	0.952	0.866	1.50E-56
EIF2A	7.40E-57	0.382052541	0.882	0.76	1.40E-52
RPL27A	1.22E-162	0.381962028	0.999	0.995	2.30E-158
RPL32	4.62E-187	0.381516963	1	0.998	8.72E-183
RPL13A	2.84E-186	0.379194625	0.999	0.999	5.36E-182
RPS3A	1.00E-138	0.377932145	0.999	0.995	1.90E-134
UBL3	6.81E-66	0.373304615	0.702	0.485	1.29E-61
SNRPN	6.71E-87	0.372583504	0.468	0.169	1.27E-82
TSPAN12	2.53E-61	0.372493052	0.628	0.394	4.78E-57
DNAJC4	5.39E-61	0.371316626	0.679	0.481	1.02E-56
PFDN5	1.19E-92	0.369933342	0.997	0.98	2.25E-88
RPL23A	2.92E-152	0.366935151	1	0.994	5.52E-148
HSPA1A	5.78E-37	0.366459085	0.468	0.267	1.09E-32
POLR1D	3.48E-54	0.365895812	0.961	0.912	6.56E-50
SEMA3C	2.08E-65	0.363808782	0.736	0.512	3.94E-61
MACROD1	8.13E-48	0.363276229	0.771	0.645	1.53E-43
EBPL	3.07E-53	0.362342424	0.942	0.827	5.79E-49
UBXN1	1.94E-50	0.362151597	0.933	0.871	3.66E-46
EEPD1	4.34E-62	0.361979199	0.547	0.312	8.19E-58
GABARAP	1.18E-50	0.36188516	0.801	0.668	2.23E-46
CLDN3	1.22E-46	0.359136851	0.997	0.97	2.30E-42
UXT	8.78E-56	0.354625575	0.965	0.905	1.66E-51
RPL29	3.49E-152	0.354256696	0.999	0.996	6.58E-148
RPL5	2.85E-105	0.354220034	0.998	0.992	5.38E-101
EEF1G	5.21E-49	0.352883295	0.805	0.709	9.85E-45

KLHDC2	4.10E-51	0.352438689	0.829	0.722	7.75E-47
WNK2	6.81E-58	0.35138032	0.615	0.41	1.29E-53
NOTCH1	2.12E-55	0.35114998	0.589	0.38	3.99E-51
MPST	7.62E-60	0.350112743	0.985	0.919	1.44E-55
TMEM59	8.33E-67	0.347071776	0.989	0.961	1.57E-62
RPS2	4.05E-105	0.347014682	0.999	0.997	7.65E-101
NOB1	3.80E-49	0.346972441	0.834	0.711	7.17E-45
MPHOSPH8	1.41E-50	0.345081158	0.856	0.737	2.66E-46
ATP8B1	6.88E-53	0.344239091	0.819	0.68	1.30E-48
SH3YL1	1.57E-46	0.342973566	0.913	0.849	2.97E-42
SATB2	2.01E-56	0.340843762	0.654	0.411	3.80E-52
VAV3	1.68E-43	0.340621009	0.669	0.501	3.17E-39
RPL24	3.84E-117	0.33862577	0.999	0.992	7.25E-113
PFN2	7.53E-87	0.336497718	0.476	0.175	1.42E-82
CDX2	7.84E-34	0.336468266	0.937	0.81	1.48E-29
RPL35A	1.99E-141	0.336435542	0.999	0.996	3.76E-137
ETS2	1.32E-45	0.335782563	0.979	0.915	2.49E-41
ATOH8	1.68E-61	0.335357475	0.465	0.206	3.17E-57
SMDT1	1.61E-47	0.332358308	0.952	0.878	3.04E-43
AIFM3	2.98E-72	0.327574063	0.531	0.249	5.63E-68
IGLC2	7.35E-34	0.326824155	0.416	0.248	1.39E-29
GOLIM4	5.96E-28	0.32516696	0.961	0.871	1.13E-23
FIS1	5.58E-47	0.325165274	0.963	0.911	1.05E-42
HIBADH	1.33E-48	0.324565519	0.756	0.606	2.52E-44
ADH1C	6.78E-39	0.321889365	0.62	0.378	1.28E-34
TSPAN8	2.01E-50	0.320777176	0.995	0.993	3.80E-46
FOXP1	5.90E-42	0.32015875	0.871	0.763	1.11E-37
CUTA	8.13E-49	0.319352737	0.979	0.924	1.54E-44
RNF186	6.61E-52	0.319156337	0.783	0.568	1.25E-47
CD99	7.54E-36	0.318032738	0.96	0.892	1.42E-31
TDGF1	5.62E-47	0.31777687	0.594	0.401	1.06E-42
RPS28	1.54E-141	0.317697022	1	0.996	2.91E-137
C9orf152	4.61E-44	0.317321556	0.715	0.549	8.71E-40
BASP1	7.04E-58	0.317223356	0.342	0.119	1.33E-53
RPS14	5.92E-133	0.316908316	0.999	0.997	1.12E-128
RPS25	9.23E-117	0.3151915	0.999	0.995	1.74E-112
RAB13	9.08E-44	0.315143108	0.889	0.772	1.71E-39
EPHX2	2.61E-55	0.31400994	0.611	0.38	4.94E-51
NHLRC3	1.46E-50	0.313775639	0.673	0.471	2.75E-46
ID1	4.50E-21	0.31349968	0.962	0.915	8.49E-17
ZNF503	1.75E-41	0.313302821	0.536	0.337	3.31E-37
EPB41L4A-AS1	5.87E-38	0.312966469	0.788	0.667	1.11E-33
SRI	2.35E-40	0.312929124	0.993	0.96	4.44E-36

PMPCB	5.84E-46	0.311208834	0.789	0.672	1.10E-41
TBPL1	1.50E-41	0.311008908	0.686	0.551	2.82E-37
ZBTB20	1.04E-43	0.310887928	0.696	0.491	1.96E-39
HIPK2	9.00E-42	0.310424938	0.652	0.485	1.70E-37
CDKN1B	1.05E-43	0.30981899	0.734	0.565	1.99E-39
ATP5F1A	4.23E-41	0.308870338	0.973	0.903	7.99E-37
SLC44A3	5.01E-47	0.308048355	0.749	0.61	9.46E-43
RPL7A	1.80E-94	0.307943749	0.998	0.995	3.40E-90
RPS24	3.28E-131	0.30601439	0.999	0.997	6.19E-127
LETMD1	1.58E-49	0.303446477	0.637	0.458	2.98E-45
EIF4B	8.07E-45	0.303281331	0.975	0.912	1.52E-40
ARSD	2.14E-47	0.302106852	0.631	0.455	4.04E-43
SYF2	8.44E-46	0.30207686	0.943	0.872	1.59E-41
PCLO	7.32E-54	0.301280367	0.389	0.169	1.38E-49
NSD3	2.58E-43	0.30118909	0.736	0.599	4.88E-39
PLCB4	3.26E-25	0.301086854	0.742	0.653	6.16E-21
PNISR	2.68E-42	0.300727383	0.952	0.87	5.05E-38
MYB	4.97E-39	0.298354571	0.786	0.659	9.39E-35
GSTK1	6.60E-38	0.298173671	0.962	0.908	1.25E-33
VPS36	1.06E-38	0.297388254	0.797	0.68	1.99E-34
HOXA11-AS	5.61E-45	0.297366519	0.577	0.382	1.06E-40
RPS17	5.62E-60	0.296935489	0.998	0.985	1.06E-55
TSC22D1	1.67E-40	0.296888105	0.977	0.918	3.15E-36
MT-CO2	1.99E-44	0.296674521	0.999	0.989	3.76E-40
HOXB6	1.19E-38	0.296262982	0.653	0.493	2.26E-34
MYADM	2.65E-50	0.29623688	0.565	0.324	5.01E-46
MKRN1	4.64E-40	0.296126499	0.893	0.816	8.76E-36
RPL18A	6.85E-116	0.295787894	1	0.997	1.29E-111
DAB2	1.17E-30	0.293317495	0.659	0.5	2.21E-26
TESC	4.32E-61	0.292999279	0.587	0.27	8.16E-57
ST13	1.83E-40	0.292924212	0.985	0.945	3.46E-36
SLC39A8	2.36E-39	0.291242069	0.604	0.414	4.46E-35
BACE2	6.72E-37	0.291192357	0.939	0.843	1.27E-32
ALKBH7	3.62E-40	0.290601972	0.939	0.855	6.83E-36
ACADM	2.86E-38	0.289693756	0.632	0.467	5.40E-34
RPL38	1.09E-85	0.288777549	0.999	0.993	2.05E-81
ATF3	9.54E-13	0.288659755	0.564	0.488	1.80E-08
GATAD1	4.73E-35	0.287884292	0.614	0.485	8.93E-31
PTEN	2.11E-50	0.287435949	0.679	0.486	3.98E-46
ZNF277	5.93E-37	0.287068788	0.607	0.462	1.12E-32
PCM1	1.04E-32	0.284047894	0.738	0.634	1.97E-28
PYCARD	2.00E-35	0.28371945	0.846	0.676	3.78E-31
SNHG7	8.83E-31	0.283658344	0.8	0.723	1.67E-26

RPS26	4.46E-90	0.283337813	0.999	0.993	8.41E-86
TRIM56	1.52E-31	0.282455427	0.72	0.607	2.87E-27
RSL1D1	3.85E-34	0.281720907	0.956	0.893	7.27E-30
STMP1	4.18E-35	0.280009375	0.895	0.825	7.90E-31
DACH1	1.37E-33	0.279081178	0.577	0.416	2.60E-29
HSBP1L1	2.62E-37	0.278232908	0.717	0.581	4.96E-33
CRNDE	2.86E-30	0.277698042	0.62	0.463	5.40E-26
RPL19	9.89E-93	0.276628858	1	0.998	1.87E-88
ZNF655	5.68E-54	0.276535295	0.547	0.301	1.07E-49
ARFIP1	2.05E-33	0.276413135	0.639	0.522	3.87E-29
FGGY	9.17E-45	0.275307634	0.501	0.307	1.73E-40
MPG	2.22E-34	0.274394735	0.808	0.732	4.20E-30
KLF4	1.97E-20	0.273066269	0.678	0.598	3.72E-16
TMEM219	3.11E-36	0.271064534	0.94	0.874	5.88E-32
CDCA7L	8.54E-37	0.270803144	0.484	0.304	1.61E-32
RPL39	4.25E-69	0.269867812	0.999	0.996	8.02E-65
NACA	1.24E-67	0.269369501	0.999	0.991	2.35E-63
OARD1	9.16E-35	0.266438251	0.681	0.553	1.73E-30
PIAS2	6.82E-33	0.263553561	0.529	0.365	1.29E-28
TMEM106B	1.06E-36	0.263385194	0.768	0.644	2.00E-32
UPF3A	1.28E-34	0.26231484	0.803	0.685	2.41E-30
RPSA	1.36E-48	0.262152709	1	0.987	2.57E-44
RPS19	3.35E-100	0.26169018	0.999	0.999	6.33E-96
TP53TG1	7.65E-36	0.261164069	0.579	0.421	1.45E-31
RPS7	1.74E-68	0.260786439	0.998	0.995	3.29E-64
GALNT6	9.71E-34	0.258817103	0.589	0.43	1.83E-29
GPX2	4.81E-28	0.256706308	0.997	0.954	9.08E-24
FAU	1.54E-79	0.256539506	0.999	0.993	2.92E-75
POLR2J3.1	4.97E-28	0.256210404	0.781	0.702	9.38E-24
CCDC28A	1.78E-37	0.256114833	0.496	0.328	3.36E-33
EGR1	9.44E-12	0.255618141	0.741	0.694	1.78E-07
ARFGEF3	1.10E-27	0.255120562	0.615	0.498	2.08E-23
TMEM63A	6.34E-32	0.255034418	0.802	0.706	1.20E-27
CCND2	8.54E-39	0.25488687	0.847	0.575	1.61E-34
FRA10AC1	1.61E-31	0.254540032	0.511	0.358	3.04E-27
SAT2	7.98E-33	0.252546674	0.755	0.644	1.51E-28
NTHL1	2.51E-30	0.251935728	0.696	0.594	4.74E-26
RPS11	6.75E-94	0.251215902	0.999	0.994	1.27E-89
RPS27A	3.02E-81	0.250242676	1	0.994	5.70E-77
SESN1	6.73E-33	0.250032313	0.622	0.445	1.27E-28
LMNA	2.57E-11	-0.250031871	0.877	0.893	4.85E-07
CXCL2	0.000849005	-0.25064905	0.396	0.454	1
PRR15L	1.35E-16	-0.250657293	0.588	0.709	2.55E-12

PPP2CA	2.34E-29	-0.250678386	0.507	0.712	4.41E-25
TRIR	4.44E-29	-0.250714576	0.944	0.944	8.39E-25
CEMIP	6.68E-15	-0.250871332	0.334	0.481	1.26E-10
GSPT1	1.58E-22	-0.251006813	0.772	0.842	2.98E-18
PODXL2	1.57E-43	-0.251215622	0.14	0.387	2.96E-39
SRA1	1.26E-34	-0.251456676	0.368	0.612	2.37E-30
TPRKB	1.23E-22	-0.251460313	0.593	0.743	2.32E-18
RBM3	6.06E-25	-0.251496613	0.939	0.936	1.14E-20
AMD1	7.92E-25	-0.251622284	0.476	0.669	1.50E-20
PITX1	3.35E-31	-0.251710191	0.329	0.574	6.32E-27
NDUFAF3	1.68E-23	-0.251818052	0.641	0.784	3.18E-19
TMEM92	6.76E-40	-0.251993531	0.067	0.272	1.28E-35
SLC38A5	3.19E-52	-0.252076857	0.071	0.317	6.02E-48
SPTBN1	1.19E-16	-0.252517294	0.753	0.836	2.24E-12
NORAD	1.91E-13	-0.252819095	0.748	0.822	3.60E-09
DUSP6	1.39E-30	-0.253214993	0.204	0.423	2.63E-26
ARF3	1.04E-33	-0.253229613	0.377	0.631	1.97E-29
BID	1.12E-20	-0.253265603	0.673	0.78	2.12E-16
HNRNPK	5.98E-30	-0.253600589	0.977	0.965	1.13E-25
YARS	3.04E-49	-0.253666741	0.181	0.466	5.74E-45
FAM107B	1.34E-54	-0.25369905	0.043	0.29	2.53E-50
ANP32E	4.33E-23	-0.254252032	0.293	0.486	8.17E-19
AURKAIP1	6.47E-26	-0.254257369	0.934	0.937	1.22E-21
CTSZ	9.30E-18	-0.254636124	0.511	0.674	1.76E-13
HDAC2	1.70E-18	-0.254737157	0.761	0.824	3.21E-14
CPNE1	1.02E-18	-0.254877999	0.531	0.695	1.93E-14
GNPNAT1	4.82E-36	-0.254941522	0.323	0.568	9.10E-32
SEC61A1	3.42E-29	-0.254969578	0.399	0.631	6.46E-25
EMC2	3.81E-34	-0.255214895	0.335	0.57	7.20E-30
POLE4	2.12E-26	-0.25537391	0.504	0.701	4.00E-22
TPD52L2	2.00E-32	-0.255376139	0.299	0.535	3.78E-28
DPCD	1.90E-38	-0.255412988	0.293	0.556	3.58E-34
HDAC1	1.82E-20	-0.25549944	0.785	0.849	3.43E-16
VCL	4.13E-30	-0.25583232	0.262	0.486	7.81E-26
PPIA	4.82E-38	-0.255907225	0.999	0.99	9.11E-34
LAMC2	9.62E-18	-0.256390433	0.188	0.343	1.82E-13
ATP6V1G1	3.62E-22	-0.256527226	0.931	0.945	6.84E-18
UCHL3	6.42E-27	-0.257052272	0.616	0.771	1.21E-22
NECTIN2	1.71E-27	-0.25726326	0.432	0.635	3.22E-23
GHITM	1.41E-23	-0.257288703	0.88	0.913	2.65E-19
RPS27L	4.75E-17	-0.257648996	0.978	0.969	8.97E-13
SHARPIN	3.46E-28	-0.257969836	0.506	0.697	6.53E-24
NDUFB8	7.21E-28	-0.258158863	0.907	0.918	1.36E-23

PCNA	6.68E-07	-0.258222107	0.438	0.528	0.01261084
PAIP2	5.79E-18	-0.258307315	0.672	0.795	1.09E-13
CTNNA1	1.12E-21	-0.258356347	0.733	0.849	2.11E-17
TNFRSF21	1.19E-29	-0.258410462	0.225	0.442	2.26E-25
DAZAP1	7.83E-26	-0.258496045	0.554	0.719	1.48E-21
STAU1	6.81E-16	-0.258502182	0.728	0.825	1.29E-11
LMNB1	1.01E-23	-0.259265941	0.208	0.385	1.91E-19
NAA50	5.26E-27	-0.259428026	0.453	0.649	9.94E-23
ATP5F1B	6.60E-23	-0.260005685	0.986	0.967	1.25E-18
NANS	1.04E-22	-0.260010686	0.814	0.868	1.96E-18
PPP1CA	8.48E-28	-0.260078224	0.896	0.915	1.60E-23
POLR2F	1.33E-23	-0.260084342	0.816	0.871	2.52E-19
RARRES1	5.09E-08	-0.260811832	0.264	0.364	0.000961906
PSMD2	6.62E-30	-0.261583795	0.504	0.705	1.25E-25
PRDX4	4.79E-23	-0.261790349	0.825	0.862	9.05E-19
ETF1	2.75E-38	-0.261850774	0.389	0.645	5.19E-34
PARK7	4.57E-28	-0.261910675	0.971	0.942	8.63E-24
KDELR2	6.52E-27	-0.262136592	0.92	0.937	1.23E-22
NDUFB6	4.84E-23	-0.262292209	0.766	0.85	9.14E-19
BCL2L1	1.00E-21	-0.26252127	0.426	0.621	1.89E-17
WEE1	2.16E-25	-0.26366954	0.299	0.492	4.08E-21
NT5DC2	2.56E-61	-0.263706435	0.045	0.31	4.83E-57
SLC3A2	0.02861386	-0.264460953	0.668	0.695	1
SOD3	8.32E-11	-0.264990385	0.206	0.326	1.57E-06
MALAT1	0.000808774	-0.265027872	1	0.992	1
PSMD12	1.11E-33	-0.265074274	0.434	0.659	2.10E-29
JUND	2.00E-12	-0.265205088	0.689	0.783	3.78E-08
SAR1B	1.53E-33	-0.266244417	0.334	0.583	2.90E-29
PAM	2.53E-63	-0.266347861	0.029	0.296	4.78E-59
XRCC5	5.51E-22	-0.266355061	0.811	0.871	1.04E-17
HYOU1	3.19E-48	-0.267265443	0.141	0.413	6.03E-44
GINS2	7.97E-36	-0.26798898	0.108	0.315	1.51E-31
POLR2K	2.66E-23	-0.26865573	0.776	0.85	5.02E-19
PRDX6	4.55E-13	-0.268860242	0.851	0.874	8.59E-09
ODC1	4.94E-21	-0.26906882	0.668	0.771	9.33E-17
GGH	3.72E-10	-0.269343696	0.789	0.805	7.02E-06
HK2	3.41E-40	-0.269385251	0.119	0.35	6.44E-36
MT1E	4.57E-15	-0.269700018	0.164	0.308	8.63E-11
OCIAD2	3.73E-16	-0.269712046	0.963	0.954	7.05E-12
GID8	9.51E-20	-0.269803182	0.568	0.705	1.80E-15
NDUFA2	3.36E-25	-0.270007182	0.884	0.909	6.35E-21
TMPRSS4	1.65E-42	-0.270091118	0.228	0.495	3.11E-38
MRPL47	9.88E-30	-0.270128733	0.532	0.723	1.87E-25

TMEM123	2.24E-12	-0.270216379	0.965	0.953	4.23E-08
PLA2G16	3.73E-08	-0.270433335	0.402	0.504	0.000703824
LAPTM4B	8.19E-18	-0.271049043	0.6	0.709	1.55E-13
NDUFB3	1.24E-29	-0.271236628	0.911	0.926	2.34E-25
ATP5MG	5.19E-40	-0.271590424	0.994	0.986	9.79E-36
PGRMC1	1.27E-23	-0.271604976	0.737	0.833	2.40E-19
ARF6	2.43E-17	-0.272111562	0.713	0.818	4.58E-13
PKP3	2.43E-23	-0.272468566	0.662	0.79	4.59E-19
SPCS3	7.69E-30	-0.272562184	0.528	0.718	1.45E-25
SH3GLB1	1.64E-18	-0.273022022	0.697	0.808	3.10E-14
OTULINL	5.51E-26	-0.274010349	0.374	0.574	1.04E-21
BHLHE40	4.93E-24	-0.274111682	0.204	0.393	9.30E-20
MRPL51	9.47E-28	-0.274196866	0.887	0.915	1.79E-23
MMAB	1.64E-32	-0.274227873	0.362	0.585	3.10E-28
PSMD8	1.79E-32	-0.274559544	0.855	0.901	3.38E-28
GARS	1.90E-28	-0.275005711	0.383	0.6	3.59E-24
CCT7	6.17E-23	-0.275122861	0.819	0.851	1.17E-18
DDX5	2.02E-24	-0.275524146	0.946	0.956	3.81E-20
PYCR1	9.10E-30	-0.275697123	0.489	0.678	1.72E-25
PPA1	1.18E-33	-0.275957829	0.988	0.98	2.23E-29
B4GALT1	2.33E-21	-0.276037404	0.313	0.502	4.40E-17
PSME1	5.95E-25	-0.276159577	0.961	0.946	1.12E-20
LAP3	1.40E-24	-0.276180544	0.497	0.68	2.64E-20
DIAPH1	8.03E-52	-0.276261229	0.236	0.529	1.52E-47
NUTF2	1.09E-29	-0.276676323	0.701	0.829	2.06E-25
UQCR10	5.89E-33	-0.276775685	0.979	0.967	1.11E-28
CDV3	4.45E-24	-0.277174195	0.673	0.79	8.41E-20
ATAD2	7.31E-32	-0.277291472	0.095	0.279	1.38E-27
THEM6	4.27E-39	-0.277507736	0.251	0.492	8.07E-35
11-Sep	1.82E-33	-0.277583249	0.477	0.687	3.43E-29
BOLA3	4.47E-26	-0.27808181	0.614	0.748	8.44E-22
GRINA	3.77E-30	-0.278091804	0.345	0.564	7.12E-26
EBNA1BP2	1.84E-25	-0.279307646	0.609	0.74	3.48E-21
UBE2I	4.36E-27	-0.279359456	0.809	0.898	8.22E-23
MVB12A	2.07E-54	-0.279588515	0.211	0.51	3.91E-50
EPHA2	5.22E-38	-0.279875355	0.1	0.312	9.86E-34
LIPH	1.35E-16	-0.280177612	0.367	0.531	2.55E-12
PET100	2.40E-26	-0.280185752	0.771	0.882	4.52E-22
FUT4	3.09E-47	-0.280490019	0.176	0.447	5.83E-43
DDX39A	8.99E-34	-0.280658427	0.322	0.558	1.70E-29
ACTR2	1.39E-32	-0.281713274	0.776	0.875	2.62E-28
CLINT1	4.95E-26	-0.281807789	0.506	0.681	9.35E-22
SRSF9	2.57E-32	-0.282153837	0.939	0.939	4.85E-28

SLC9A3R1	2.04E-47	-0.282491789	0.172	0.441	3.86E-43
SAPCD2	1.17E-46	-0.282552538	0.111	0.359	2.20E-42
FDX1	4.67E-24	-0.28276999	0.598	0.736	8.82E-20
PCBP1	1.37E-32	-0.282984003	0.835	0.9	2.58E-28
TAF7	1.43E-10	-0.283062055	0.819	0.854	2.70E-06
TMEM54	6.24E-19	-0.283212834	0.949	0.934	1.18E-14
NDUFC1	6.39E-30	-0.283268217	0.902	0.928	1.21E-25
SDHB	1.23E-27	-0.283286033	0.666	0.796	2.32E-23
EPCAM	7.38E-24	-0.283666574	0.998	0.994	1.39E-19
DNMT1	6.35E-32	-0.283723356	0.241	0.458	1.20E-27
NOP58	9.10E-26	-0.283900058	0.538	0.703	1.72E-21
SET	3.82E-21	-0.284797755	0.973	0.956	7.21E-17
SEPHS2	1.12E-14	-0.284836933	0.716	0.771	2.11E-10
BRI3BP	3.34E-30	-0.285325807	0.418	0.625	6.30E-26
MTMR11	4.96E-51	-0.286258258	0.153	0.435	9.37E-47
ETFB	1.10E-23	-0.286571101	0.879	0.908	2.07E-19
HLA-F	5.03E-21	-0.286783262	0.361	0.537	9.50E-17
HSBP1	3.80E-35	-0.287458834	0.914	0.931	7.18E-31
ATP6V0E1	1.87E-30	-0.287529448	0.959	0.955	3.53E-26
PSMB1	9.50E-35	-0.287979669	0.971	0.956	1.79E-30
SRP19	8.45E-30	-0.288082218	0.516	0.708	1.60E-25
RALY	5.30E-26	-0.288088623	0.827	0.885	1.00E-21
CCDC85B	1.71E-26	-0.288111636	0.506	0.688	3.23E-22
VDAC2	1.75E-10	-0.288411408	0.966	0.944	3.30E-06
UQCRC1	6.77E-28	-0.288504988	0.878	0.914	1.28E-23
ZFAS1	0.188264484	-0.28865465	0.996	0.973	1
TAP1	8.13E-21	-0.288743134	0.4	0.587	1.53E-16
ACTR3	2.82E-26	-0.289263913	0.754	0.852	5.33E-22
COX8A	5.73E-32	-0.289684154	0.987	0.976	1.08E-27
AHSA1	2.07E-25	-0.289751546	0.614	0.745	3.90E-21
NDUFA12	1.11E-34	-0.290143114	0.881	0.919	2.09E-30
GGCT	1.63E-18	-0.290286875	0.798	0.844	3.08E-14
NASP	3.05E-12	-0.290388187	0.573	0.65	5.76E-08
LAMTOR5	4.16E-39	-0.291370814	0.94	0.951	7.86E-35
GCSH	2.89E-27	-0.291848213	0.583	0.732	5.45E-23
DCUN1D5	5.13E-46	-0.292201352	0.294	0.56	9.70E-42
ERRFI1	3.83E-09	-0.292527034	0.286	0.4	7.23E-05
SPCS2	2.69E-26	-0.292649874	0.899	0.915	5.08E-22
RPL26L1	1.53E-37	-0.293032021	0.499	0.725	2.89E-33
RAB11A	1.63E-28	-0.293742762	0.9	0.931	3.07E-24
DERL1	3.39E-40	-0.293776697	0.369	0.621	6.40E-36
PRMT1	3.06E-23	-0.293917172	0.732	0.802	5.77E-19
FAM84B	2.96E-37	-0.293936875	0.264	0.502	5.59E-33

GSDMD	2.43E-37	-0.294020195	0.439	0.669	4.59E-33
PPP4C	7.47E-37	-0.294082105	0.813	0.893	1.41E-32
ECHS1	2.34E-33	-0.294141066	0.891	0.916	4.41E-29
NMI	7.18E-40	-0.294210391	0.377	0.627	1.36E-35
SLC25A39	1.88E-24	-0.29438142	0.763	0.836	3.54E-20
HLA-B	0.037090615	-0.295761839	0.996	0.98	1
PHF20L1	2.14E-26	-0.295885068	0.465	0.65	4.04E-22
MCM7	1.77E-19	-0.296172331	0.378	0.53	3.35E-15
RTN4	8.81E-25	-0.296381796	0.918	0.934	1.66E-20
LETM1	5.31E-39	-0.296403845	0.318	0.575	1.00E-34
CDC37	2.03E-33	-0.296425184	0.772	0.862	3.82E-29
HKDC1	7.46E-69	-0.296447465	0.024	0.306	1.41E-64
PDIA4	3.19E-27	-0.29803755	0.807	0.878	6.02E-23
PPARG	3.89E-18	-0.298352504	0.491	0.642	7.35E-14
MRPS34	2.24E-28	-0.298842179	0.824	0.874	4.22E-24
CASP1	2.85E-19	-0.299199348	0.353	0.52	5.39E-15
MDK	8.12E-12	-0.299503683	0.647	0.78	1.53E-07
MYH9	4.63E-23	-0.299521904	0.807	0.874	8.74E-19
FAT1	1.55E-30	-0.300138597	0.485	0.694	2.92E-26
TUBA4A	4.04E-34	-0.301004843	0.288	0.524	7.62E-30
EIF4G1	2.34E-34	-0.301085337	0.554	0.74	4.41E-30
MCM3	4.82E-34	-0.301802294	0.232	0.45	9.10E-30
CTNNB1	6.20E-13	-0.302076013	0.815	0.892	1.17E-08
GFPT1	1.99E-21	-0.30220423	0.618	0.761	3.76E-17
CALM3	4.58E-24	-0.302628872	0.766	0.836	8.65E-20
ASRGL1	1.47E-37	-0.302891833	0.297	0.54	2.77E-33
RNASET2	1.29E-23	-0.303093016	0.866	0.896	2.43E-19
FASN	1.38E-49	-0.303145628	0.215	0.496	2.60E-45
MYO6	2.09E-25	-0.303186958	0.645	0.813	3.95E-21
TFG	2.65E-33	-0.303576734	0.572	0.765	5.00E-29
POLR2E	1.25E-34	-0.303587773	0.677	0.81	2.35E-30
AZIN1	4.97E-29	-0.303620258	0.496	0.694	9.39E-25
AP3S1	2.43E-24	-0.304556193	0.697	0.811	4.59E-20
PLCB3	5.60E-42	-0.304817794	0.295	0.551	1.06E-37
CSE1L	5.67E-40	-0.304831678	0.239	0.486	1.07E-35
KIF5B	5.10E-19	-0.305116722	0.966	0.947	9.62E-15
CTTN	1.33E-27	-0.305131822	0.678	0.833	2.51E-23
ERG28	1.33E-25	-0.305172614	0.556	0.71	2.51E-21
NDUFA3	9.43E-35	-0.306311324	0.919	0.95	1.78E-30
CENPM	1.73E-52	-0.306512929	0.054	0.294	3.26E-48
RPL36AL	1.49E-34	-0.306862727	0.987	0.98	2.82E-30
PLEC	8.23E-24	-0.307136422	0.342	0.542	1.55E-19
SSR3	2.35E-29	-0.307323722	0.812	0.878	4.44E-25

DAD1	1.34E-37	-0.307491762	0.916	0.933	2.52E-33
LYN	2.09E-59	-0.307555068	0.188	0.496	3.94E-55
HMGCR	2.64E-39	-0.308192914	0.225	0.47	4.99E-35
UBE2N	1.91E-31	-0.30935292	0.678	0.808	3.61E-27
HMGA1	3.01E-19	-0.309758155	0.9	0.902	5.69E-15
DPP7	2.13E-40	-0.311333234	0.421	0.678	4.02E-36
CDS1	2.79E-39	-0.311648312	0.315	0.56	5.27E-35
SEC13	9.07E-46	-0.311699955	0.385	0.647	1.71E-41
HSPA1B	4.38E-06	-0.312008187	0.543	0.628	0.08264318
SNRPD3	4.67E-35	-0.312035399	0.786	0.867	8.81E-31
GADD45GIP1	7.59E-34	-0.312190351	0.866	0.903	1.43E-29
NOP56	3.10E-27	-0.312708082	0.54	0.704	5.84E-23
MZT2A	1.79E-33	-0.312768953	0.928	0.937	3.39E-29
NUDC	3.64E-29	-0.313815026	0.788	0.861	6.88E-25
BLCAP	1.73E-31	-0.314112971	0.372	0.58	3.27E-27
SMIM31	2.17E-26	-0.314386054	0.303	0.5	4.09E-22
GSTM4	5.84E-57	-0.314553489	0.14	0.431	1.10E-52
ATP5F1C	2.60E-38	-0.315024625	0.967	0.958	4.90E-34
SKP1	3.59E-32	-0.315226741	0.986	0.969	6.79E-28
CKLF	4.48E-27	-0.31541718	0.596	0.751	8.46E-23
GSTO1	3.75E-28	-0.315713434	0.836	0.886	7.09E-24
CFDP1	7.11E-22	-0.315898045	0.691	0.809	1.34E-17
SLC25A1	1.53E-42	-0.31712245	0.322	0.579	2.90E-38
MARCKS	1.37E-20	-0.317150751	0.819	0.879	2.59E-16
SLC16A1	7.62E-77	-0.318046035	0.043	0.351	1.44E-72
FAM96B	2.37E-41	-0.318201984	0.785	0.87	4.47E-37
USP53	1.13E-16	-0.318325955	0.389	0.564	2.13E-12
TOMM5	2.04E-43	-0.318361355	0.352	0.611	3.85E-39
ORMDL2	3.91E-45	-0.318545185	0.418	0.692	7.39E-41
CISD1	3.37E-29	-0.318685121	0.764	0.841	6.36E-25
PLP2	2.68E-23	-0.319285424	0.848	0.892	5.06E-19
PTP4A1	4.74E-33	-0.3200862	0.486	0.709	8.96E-29
REEP3	5.39E-41	-0.320254014	0.427	0.681	1.02E-36
DUOX2	4.96E-09	-0.320316118	0.255	0.35	9.37E-05
CHMP2B	6.46E-28	-0.32037062	0.739	0.843	1.22E-23
CANX	1.60E-29	-0.320437329	0.934	0.938	3.02E-25
MRPL17	5.75E-42	-0.320694094	0.433	0.674	1.09E-37
HSPH1	1.18E-23	-0.320807951	0.505	0.676	2.23E-19
COX7C	1.37E-65	-0.32115656	0.996	0.992	2.60E-61
UBE2T	7.02E-42	-0.321636072	0.129	0.362	1.32E-37
SPINT2	8.81E-36	-0.321738644	0.995	0.981	1.66E-31
RBX1	3.13E-44	-0.322673915	0.891	0.916	5.92E-40
MAP1LC3B	1.42E-23	-0.322749012	0.623	0.779	2.68E-19

ATP2B1	1.31E-32	-0.322795095	0.431	0.648	2.48E-28
ARPC4	3.51E-38	-0.323453487	0.635	0.802	6.64E-34
EIF5B	1.45E-35	-0.323690163	0.794	0.877	2.74E-31
DYNLL1	1.89E-31	-0.324088336	0.989	0.973	3.57E-27
AGO2	4.73E-41	-0.324240513	0.327	0.584	8.93E-37
STRAP	5.72E-32	-0.324787375	0.724	0.824	1.08E-27
ELOB	9.78E-59	-0.324863337	0.995	0.983	1.85E-54
RABAC1	5.98E-26	-0.325145297	0.657	0.799	1.13E-21
NAPRT	3.50E-31	-0.325171364	0.615	0.778	6.61E-27
CISD2	4.92E-32	-0.325208044	0.642	0.785	9.30E-28
DYNC1I2	7.98E-26	-0.325254531	0.782	0.884	1.51E-21
ACSL3	9.87E-45	-0.325395993	0.309	0.583	1.86E-40
MTCH2	9.02E-39	-0.325694937	0.783	0.874	1.70E-34
MRPL14	2.15E-41	-0.32663237	0.834	0.892	4.05E-37
PSMD14	4.29E-38	-0.326695311	0.555	0.742	8.10E-34
DNAJA1	2.24E-24	-0.326809311	0.744	0.839	4.22E-20
KLF6	1.52E-08	-0.326831789	0.833	0.857	0.000286943
HNRNPH1	2.96E-15	-0.326924525	0.738	0.843	5.58E-11
COMT	7.14E-29	-0.327043247	0.762	0.861	1.35E-24
RAB1A	4.48E-37	-0.328326363	0.789	0.887	8.45E-33
HNRNPC	1.01E-31	-0.329024454	0.966	0.962	1.91E-27
WDR34	6.40E-30	-0.329170443	0.364	0.562	1.21E-25
TMPRSS2	2.38E-28	-0.329255187	0.489	0.679	4.50E-24
CTSV	1.42E-51	-0.329563795	0.058	0.304	2.68E-47
CDKN1A	6.74E-30	-0.329647581	0.283	0.5	1.27E-25
MPC2	7.30E-32	-0.329809791	0.939	0.934	1.38E-27
SLBP	2.35E-22	-0.330562974	0.462	0.63	4.44E-18
HES4	1.28E-57	-0.330842968	0.082	0.352	2.42E-53
NDUFA6	2.02E-37	-0.331012991	0.886	0.92	3.82E-33
VPS28	4.00E-30	-0.33112254	0.86	0.913	7.55E-26
SLC25A3	2.87E-50	-0.331258222	0.988	0.967	5.43E-46
CYP2S1	6.02E-43	-0.331864293	0.159	0.403	1.14E-38
ME1	2.60E-73	-0.332107559	0.062	0.378	4.92E-69
CDH1	4.53E-25	-0.332937842	0.759	0.858	8.55E-21
SSR4	2.58E-49	-0.334034058	0.975	0.971	4.87E-45
H2AFY2	1.51E-92	-0.334784157	0.016	0.363	2.86E-88
RNF114	1.13E-37	-0.335011273	0.613	0.788	2.14E-33
BAX	4.12E-35	-0.335203397	0.748	0.856	7.78E-31
TARS	1.77E-47	-0.335400329	0.333	0.607	3.34E-43
HSPA4	1.30E-52	-0.335840336	0.318	0.614	2.46E-48
SMC4	5.06E-23	-0.335902335	0.314	0.487	9.55E-19
ACTG1	4.49E-23	-0.33614892	0.999	0.995	8.49E-19
ATP2A2	4.85E-48	-0.336172514	0.422	0.687	9.15E-44

ZMYND8	1.06E-33	-0.336303161	0.391	0.628	2.01E-29
ZWINT	5.12E-34	-0.337118527	0.142	0.347	9.67E-30
HINT1	2.01E-60	-0.337579901	0.997	0.987	3.79E-56
SERPINH1	1.48E-48	-0.339501939	0.282	0.562	2.80E-44
UGDH	6.11E-28	-0.339945903	0.507	0.69	1.15E-23
CEACAM1	1.17E-17	-0.340438368	0.258	0.413	2.20E-13
GSS	2.28E-46	-0.342094543	0.342	0.6	4.30E-42
IFNGR1	5.69E-34	-0.342216348	0.413	0.636	1.07E-29
HNRNPD	5.23E-31	-0.342663452	0.726	0.827	9.87E-27
PRKDC	2.99E-31	-0.34302902	0.572	0.726	5.65E-27
HNRNPF	1.76E-39	-0.343032875	0.898	0.929	3.32E-35
COPS9	5.69E-41	-0.343106895	0.832	0.902	1.08E-36
CD81	4.29E-32	-0.34310791	0.612	0.753	8.10E-28
C1orf21	3.25E-58	-0.343460697	0.238	0.551	6.13E-54
CDKN3	8.96E-50	-0.344347391	0.044	0.273	1.69E-45
PTBP1	1.51E-47	-0.344689342	0.527	0.768	2.86E-43
TMEM167A	3.95E-44	-0.344862832	0.58	0.783	7.45E-40
UBE2S	2.43E-37	-0.345222511	0.223	0.461	4.59E-33
IQGAP2	8.81E-54	-0.346031709	0.172	0.45	1.66E-49
NDUFV2	1.19E-47	-0.346144201	0.904	0.93	2.25E-43
TMEM97	1.85E-42	-0.347326179	0.289	0.537	3.48E-38
CD63	2.19E-52	-0.348797841	0.998	0.982	4.13E-48
P4HA1	1.11E-39	-0.348910175	0.195	0.435	2.10E-35
MZT2B	1.50E-43	-0.348974259	0.978	0.96	2.83E-39
CEBPG	2.43E-34	-0.349169706	0.48	0.699	4.58E-30
CENPX	2.78E-23	-0.349940405	0.678	0.781	5.26E-19
CLTA	4.36E-52	-0.350833232	0.959	0.96	8.23E-48
LMO4	2.27E-55	-0.350896945	0.12	0.394	4.29E-51
GTF2A2	8.48E-36	-0.350943019	0.701	0.829	1.60E-31
HSPA9	2.53E-30	-0.351520597	0.784	0.863	4.77E-26
HDGF	3.19E-37	-0.351584528	0.699	0.819	6.02E-33
TALDO1	1.20E-40	-0.352762748	0.884	0.917	2.27E-36
RPN1	2.74E-43	-0.352763971	0.647	0.817	5.18E-39
ATP5MC1	1.39E-26	-0.353146603	0.946	0.902	2.62E-22
CCT2	1.62E-36	-0.353988393	0.799	0.863	3.05E-32
MRFAP1	1.82E-35	-0.354103916	0.843	0.873	3.43E-31
NDUFS5	1.02E-50	-0.354462067	0.985	0.974	1.93E-46
CCL20	2.34E-06	-0.354808949	0.289	0.377	0.044221741
PSMD1	9.59E-49	-0.355656046	0.444	0.692	1.81E-44
SSB	1.75E-37	-0.356066743	0.833	0.883	3.30E-33
SURF4	7.60E-55	-0.357791666	0.412	0.685	1.43E-50
ANP32A	4.19E-38	-0.358921771	0.686	0.82	7.91E-34
RABL6	8.35E-36	-0.358998195	0.695	0.81	1.58E-31

EGLN3	3.85E-39	-0.359448604	0.212	0.454	7.27E-35
H2AFJ	4.08E-43	-0.360707137	0.894	0.932	7.71E-39
SERPINB6	9.85E-29	-0.360896707	0.952	0.94	1.86E-24
VCP	9.63E-41	-0.360921118	0.582	0.772	1.82E-36
F3	1.94E-26	-0.361605629	0.202	0.4	3.67E-22
PSMC4	8.40E-49	-0.362560018	0.52	0.753	1.59E-44
UBE2V2	5.14E-37	-0.363312475	0.642	0.787	9.71E-33
HNRNPDL	5.61E-35	-0.363518692	0.794	0.881	1.06E-30
DTYMK	2.43E-31	-0.363680395	0.333	0.532	4.58E-27
ADM	6.89E-10	-0.363925935	0.18	0.28	1.30E-05
PLEK2	1.42E-52	-0.364734887	0.36	0.633	2.67E-48
ARL6IP1	8.35E-10	-0.365699084	0.874	0.886	1.58E-05
TMEM258	1.27E-60	-0.366315782	0.969	0.968	2.39E-56
GNG5	1.17E-45	-0.366433743	0.943	0.957	2.20E-41
AKAP7	5.58E-45	-0.366603709	0.089	0.32	1.05E-40
TPM1	2.96E-34	-0.366778356	0.946	0.958	5.58E-30
GPI	7.74E-37	-0.366869693	0.74	0.852	1.46E-32
DDT	2.10E-46	-0.367231268	0.945	0.939	3.96E-42
SRSF7	6.44E-28	-0.367791104	0.802	0.857	1.22E-23
PDCD5	7.83E-45	-0.367966972	0.837	0.887	1.48E-40
MTDH	8.19E-41	-0.368112702	0.94	0.923	1.55E-36
GALE	3.34E-54	-0.368776342	0.365	0.662	6.30E-50
PFKP	1.11E-55	-0.368811121	0.233	0.526	2.10E-51
NEDD8	1.77E-52	-0.369708588	0.936	0.951	3.34E-48
SNRPA1	2.69E-41	-0.369900819	0.477	0.686	5.07E-37
PSMB9	1.68E-09	-0.369917137	0.5	0.585	3.18E-05
PGP	5.54E-70	-0.372074588	0.196	0.528	1.05E-65
SRSF2	1.45E-36	-0.372180502	0.813	0.891	2.75E-32
ALDOA	1.48E-42	-0.372309671	0.438	0.664	2.80E-38
KRT10	9.84E-40	-0.373357615	0.706	0.835	1.86E-35
TBCA	2.38E-54	-0.373558634	0.942	0.95	4.50E-50
ENSA	1.72E-48	-0.373988578	0.781	0.869	3.25E-44
CDKN2A	3.91E-85	-0.376645344	0.035	0.369	7.38E-81
RHOA	1.31E-52	-0.377548726	0.943	0.95	2.47E-48
MVD	5.47E-30	-0.377742451	0.317	0.522	1.03E-25
TMEM141	1.24E-34	-0.378782242	0.959	0.944	2.35E-30
PLIN2	6.06E-32	-0.379521689	0.382	0.603	1.14E-27
PSMD7	6.41E-48	-0.379853127	0.724	0.85	1.21E-43
CALM2	1.93E-34	-0.38004462	0.992	0.978	3.65E-30
HNF4A	6.88E-26	-0.380433925	0.442	0.647	1.30E-21
LRRFIP1	5.02E-42	-0.380729229	0.672	0.835	9.47E-38
PTGES3	9.88E-39	-0.38101911	0.965	0.962	1.87E-34
RRBP1	1.94E-32	-0.381052215	0.827	0.885	3.67E-28

FAM49B	6.84E-48	-0.381346025	0.426	0.684	1.29E-43
SRSF3	4.33E-40	-0.381643727	0.946	0.939	8.18E-36
MRPL36	5.60E-51	-0.381972966	0.591	0.788	1.06E-46
TKT	3.72E-41	-0.382146734	0.895	0.932	7.02E-37
ARPC5	9.47E-44	-0.38246928	0.856	0.905	1.79E-39
ARPC1B	2.39E-33	-0.382856771	0.849	0.896	4.50E-29
GIPC1	6.95E-48	-0.383071464	0.629	0.791	1.31E-43
PSMB3	2.29E-45	-0.383423921	0.888	0.911	4.33E-41
PPP2R1A	1.10E-44	-0.383700652	0.759	0.874	2.08E-40
IFITM1	3.75E-11	-0.384859816	0.78	0.8	7.08E-07
CTSD	6.71E-11	-0.385759417	0.854	0.894	1.27E-06
RDH11	2.30E-69	-0.386306487	0.288	0.631	4.35E-65
FUOM	1.19E-77	-0.38761862	0.135	0.477	2.24E-73
CAPZB	2.95E-52	-0.388188457	0.828	0.91	5.57E-48
CEACAM5	2.80E-17	-0.388322752	0.92	0.88	5.29E-13
TMBIM1	1.69E-32	-0.388413778	0.42	0.641	3.19E-28
GLRX	2.76E-25	-0.389109531	0.473	0.65	5.21E-21
DYNLRB1	9.69E-37	-0.390269484	0.847	0.911	1.83E-32
HSD17B12	1.62E-31	-0.39029874	0.793	0.865	3.06E-27
CDK2AP2	2.20E-42	-0.391737051	0.443	0.683	4.15E-38
DNAJB11	9.75E-59	-0.392268518	0.384	0.668	1.84E-54
HLA-C	6.17E-22	-0.392598055	0.991	0.983	1.17E-17
TCP1	1.14E-36	-0.393241064	0.724	0.824	2.15E-32
SPATS2L	6.27E-43	-0.39396014	0.519	0.752	1.18E-38
MYL12A	2.24E-50	-0.394462549	0.987	0.98	4.23E-46
DHFR	1.39E-61	-0.395312761	0.106	0.396	2.62E-57
PSMA4	8.46E-42	-0.395681502	0.931	0.934	1.60E-37
MISP	1.84E-05	-0.395875416	0.805	0.827	0.346497292
ATP5MPL	2.66E-68	-0.396560629	0.987	0.976	5.02E-64
RND3	3.76E-38	-0.397364289	0.217	0.454	7.10E-34
MAPRE1	2.67E-53	-0.397653591	0.389	0.659	5.05E-49
MRPL52	5.59E-56	-0.397679081	0.783	0.886	1.06E-51
MAD2L1	1.08E-46	-0.398240154	0.081	0.309	2.05E-42
LSM3	3.12E-39	-0.398679084	0.828	0.899	5.89E-35
RBM39	4.38E-30	-0.399037175	0.924	0.948	8.27E-26
PRSS22	1.18E-38	-0.400108183	0.143	0.369	2.22E-34
DPM1	7.54E-42	-0.400232693	0.536	0.721	1.42E-37
ETHE1	2.16E-40	-0.400241847	0.803	0.87	4.09E-36
GPAA1	9.10E-49	-0.400375844	0.473	0.704	1.72E-44
BCAP31	5.53E-52	-0.401097898	0.812	0.892	1.04E-47
GRPEL1	1.84E-55	-0.401400782	0.392	0.665	3.48E-51
SDCBP2	1.05E-51	-0.402225449	0.104	0.362	1.99E-47
CTSH	1.91E-36	-0.402859242	0.553	0.747	3.61E-32

HM13	4.35E-50	-0.4037709	0.558	0.772	8.21E-46
SYNCRIP	4.06E-45	-0.404382507	0.692	0.837	7.66E-41
TPM4	1.79E-40	-0.404976494	0.786	0.878	3.39E-36
DHCR24	1.62E-75	-0.405266587	0.157	0.499	3.07E-71
PSMB5	2.48E-62	-0.405364981	0.867	0.917	4.69E-58
EXOSC4	1.32E-61	-0.406813152	0.361	0.656	2.49E-57
HIGD1A	8.78E-46	-0.407103862	0.586	0.783	1.66E-41
GSN	4.60E-23	-0.410773338	0.462	0.651	8.69E-19
SERF2	1.65E-108	-0.411179585	0.998	0.996	3.11E-104
PMEPA1	9.18E-10	-0.411899992	0.609	0.697	1.73E-05
MT2A	3.61E-07	-0.412210082	0.343	0.447	0.006823144
MT-ND6	1.18E-25	-0.412753239	0.604	0.758	2.23E-21
BIRC5	1.26E-49	-0.41279781	0.032	0.253	2.37E-45
COX17	4.19E-43	-0.412836788	0.64	0.812	7.91E-39
RHOBTB3	1.69E-45	-0.413431421	0.496	0.727	3.19E-41
RAD21	6.02E-28	-0.413481649	0.596	0.753	1.14E-23
LINC01133	9.82E-48	-0.413846011	0.162	0.42	1.85E-43
YWHAH	6.20E-41	-0.413947572	0.664	0.823	1.17E-36
XBP1	2.17E-36	-0.414020864	0.777	0.867	4.10E-32
VEGFA	6.49E-32	-0.414915018	0.333	0.557	1.23E-27
MGST3	5.50E-46	-0.415673273	0.948	0.949	1.04E-41
LGALS3BP	1.18E-37	-0.416182057	0.907	0.919	2.24E-33
ATP5PF	1.50E-73	-0.416411882	0.985	0.968	2.84E-69
HNRNPM	1.28E-43	-0.416556447	0.768	0.858	2.41E-39
TRMT112	3.97E-51	-0.41659629	0.876	0.928	7.51E-47
DDX21	3.88E-47	-0.417100658	0.733	0.837	7.34E-43
ATP1B3	8.28E-46	-0.419803086	0.808	0.877	1.56E-41
YBX1	7.41E-64	-0.41991624	0.998	0.991	1.40E-59
MGST1	6.41E-43	-0.420678626	0.962	0.945	1.21E-38
FKBP2	3.22E-52	-0.420794128	0.853	0.93	6.08E-48
LACTB2	1.29E-54	-0.422041331	0.347	0.615	2.43E-50
TYMS	3.82E-59	-0.422390481	0.091	0.367	7.21E-55
LSM4	3.62E-39	-0.424384824	0.865	0.89	6.83E-35
HNRNPAB	2.64E-46	-0.425667245	0.852	0.899	4.99E-42
TIMM17A	4.23E-65	-0.426457248	0.457	0.721	7.99E-61
DHCR7	2.63E-53	-0.427559967	0.214	0.491	4.97E-49
SLC2A1	1.44E-59	-0.428556854	0.079	0.352	2.71E-55
ANXA3	1.31E-31	-0.429096286	0.699	0.825	2.46E-27
ERH	3.21E-59	-0.429604557	0.948	0.942	6.05E-55
ARF4	5.37E-54	-0.429649199	0.738	0.871	1.01E-49
CAP1	7.90E-51	-0.429865849	0.761	0.881	1.49E-46
C19orf33	0.00049153	-0.430626058	0.813	0.789	1
SLC6A8	2.73E-47	-0.430879932	0.085	0.328	5.16E-43

SMS	5.00E-61	-0.431422439	0.687	0.85	9.44E-57
COX6A1	1.32E-86	-0.4325499	0.994	0.985	2.48E-82
CKS1B	4.61E-29	-0.432599152	0.465	0.648	8.71E-25
HMGCS1	2.28E-35	-0.433658095	0.274	0.493	4.31E-31
PSAT1	5.12E-78	-0.433731773	0.031	0.343	9.66E-74
FLNB	2.17E-41	-0.43404068	0.375	0.63	4.10E-37
B2M	1.35E-13	-0.435092863	1	0.997	2.55E-09
NPM1	1.13E-42	-0.436024822	0.998	0.983	2.13E-38
SELENOS	2.76E-42	-0.436469948	0.751	0.858	5.21E-38
MORF4L2	4.28E-58	-0.436951208	0.702	0.866	8.09E-54
NCL	7.23E-28	-0.437874058	0.963	0.932	1.37E-23
PFDN2	3.66E-60	-0.43838246	0.732	0.868	6.90E-56
CDC42	9.65E-44	-0.440230885	0.938	0.957	1.82E-39
CACYBP	1.16E-40	-0.441135799	0.678	0.802	2.19E-36
TK1	2.04E-64	-0.441556775	0.042	0.317	3.86E-60
NAA20	4.45E-15	-0.442056152	0.675	0.786	8.40E-11
TSPAN3	4.21E-45	-0.44209664	0.746	0.851	7.95E-41
COX6C	1.61E-78	-0.443672708	0.999	0.988	3.05E-74
REEP5	9.88E-61	-0.443973939	0.733	0.877	1.87E-56
BZW1	2.34E-61	-0.444780387	0.691	0.866	4.42E-57
MGST2	5.82E-56	-0.445938433	0.867	0.917	1.10E-51
RNASE1	1.11E-55	-0.447810699	0.061	0.313	2.09E-51
HMGB3	3.01E-51	-0.4488307	0.392	0.668	5.68E-47
SNRPB	2.22E-51	-0.45026441	0.924	0.93	4.19E-47
MINOS1	3.17E-69	-0.45066396	0.981	0.97	5.98E-65
COPE	1.89E-74	-0.450718751	0.851	0.92	3.57E-70
NDUFS6	2.11E-67	-0.451745486	0.943	0.94	3.99E-63
H2AFY	1.24E-60	-0.452299083	0.934	0.946	2.35E-56
LSR	1.52E-59	-0.453439612	0.84	0.914	2.87E-55
ARF1	2.72E-68	-0.453924168	0.881	0.932	5.13E-64
FCGBP	1.37E-22	-0.454448957	0.169	0.346	2.59E-18
ARPC3	5.41E-95	-0.45459821	0.982	0.974	1.02E-90
SCAND1	2.31E-56	-0.455117561	0.815	0.906	4.37E-52
CLDN2	7.26E-17	-0.455224504	0.512	0.619	1.37E-12
ABRACL	9.12E-59	-0.455429604	0.762	0.882	1.72E-54
BMP4	6.00E-17	-0.455567094	0.347	0.495	1.13E-12
SLC1A5	1.35E-61	-0.456198677	0.257	0.558	2.56E-57
EEF1D	3.48E-49	-0.457123464	0.998	0.985	6.58E-45
KPNA2	1.63E-60	-0.457459734	0.124	0.418	3.07E-56
CEBPD	1.61E-27	-0.459544656	0.691	0.798	3.03E-23
GCNT3	1.30E-67	-0.46073987	0.016	0.285	2.46E-63
CRIP1	1.33E-30	-0.461482406	0.144	0.332	2.51E-26
LRPAP1	3.37E-60	-0.461497184	0.601	0.807	6.37E-56

SEC61B	4.11E-81	-0.463002278	0.944	0.96	7.76E-77
GTF3C6	2.25E-66	-0.463709761	0.681	0.847	4.25E-62
CHMP4B	1.06E-50	-0.467485823	0.906	0.934	2.00E-46
COX7A2	2.70E-106	-0.467583537	0.993	0.987	5.10E-102
GPRC5A	6.58E-17	-0.469242846	0.698	0.791	1.24E-12
HNRNPA3	1.16E-54	-0.469589902	0.914	0.93	2.18E-50
MKI67	3.02E-48	-0.471728322	0.034	0.251	5.70E-44
AP2S1	2.39E-71	-0.473213117	0.817	0.907	4.51E-67
NOP10	3.98E-76	-0.473810713	0.872	0.925	7.52E-72
PRR13	1.95E-70	-0.474045539	0.898	0.947	3.68E-66
EIF4G2	3.68E-68	-0.474138263	0.919	0.955	6.95E-64
INSIG1	9.43E-39	-0.474361781	0.274	0.519	1.78E-34
DYNLT1	1.26E-73	-0.474743788	0.859	0.926	2.39E-69
NDUFAB1	8.43E-65	-0.475717794	0.929	0.936	1.59E-60
ANXA5	9.09E-59	-0.475720591	0.337	0.633	1.72E-54
PSMB2	1.60E-67	-0.476569811	0.786	0.894	3.03E-63
IDH1	4.50E-59	-0.477443409	0.556	0.798	8.51E-55
PUF60	5.89E-63	-0.477616961	0.568	0.794	1.11E-58
CEBPB	1.63E-20	-0.478371036	0.703	0.785	3.08E-16
SDF2L1	6.28E-46	-0.478783346	0.609	0.773	1.19E-41
EMP2	1.13E-59	-0.47938376	0.499	0.744	2.13E-55
ACOT7	6.75E-99	-0.479576611	0.132	0.525	1.27E-94
HMGB2	0.004903749	-0.479694767	0.658	0.57	1
POLR2L	8.37E-68	-0.483483028	0.939	0.948	1.58E-63
ASPH	4.66E-57	-0.485307016	0.643	0.825	8.79E-53
MRPL13	6.88E-62	-0.486133206	0.686	0.86	1.30E-57
UBE2L3	1.84E-76	-0.487220452	0.743	0.875	3.47E-72
PRSS8	3.32E-41	-0.487837514	0.508	0.711	6.27E-37
LDLR	2.37E-80	-0.487984535	0.16	0.514	4.48E-76
TMBIM6	8.22E-87	-0.490818971	0.981	0.971	1.55E-82
ADRM1	1.10E-56	-0.490948545	0.695	0.843	2.08E-52
TMED10	2.03E-88	-0.491387771	0.768	0.897	3.83E-84
EPB41L2	4.73E-59	-0.49275248	0.528	0.771	8.94E-55
TMEM45B	6.45E-50	-0.493008815	0.582	0.777	1.22E-45
PRELID3B	5.32E-54	-0.49349386	0.643	0.817	1.00E-49
SLC52A2	9.89E-71	-0.495320376	0.468	0.737	1.87E-66
TCEAL9	3.74E-62	-0.495415276	0.256	0.574	7.07E-58
PSMA5	1.15E-78	-0.496927616	0.748	0.885	2.16E-74
NPC2	2.08E-60	-0.498046866	0.906	0.946	3.92E-56
ATP5MC3	2.00E-78	-0.499825238	0.996	0.976	3.78E-74
NDUFB9	7.84E-68	-0.500903787	0.971	0.963	1.48E-63
HIST1H4C	0.141436995	-0.50092411	0.834	0.805	1
PTGR1	3.18E-67	-0.500929018	0.342	0.651	6.00E-63

CES2	3.55E-43	-0.500995911	0.313	0.556	6.70E-39
SNRPD1	6.18E-58	-0.501976148	0.872	0.905	1.17E-53
PFN1	1.37E-71	-0.503850186	0.996	0.982	2.58E-67
PA2G4	3.96E-57	-0.505237334	0.9	0.915	7.47E-53
PSMA3	1.30E-72	-0.506155988	0.74	0.882	2.45E-68
AKR1B10	4.67E-83	-0.506742708	0.018	0.336	8.81E-79
FAM162A	1.25E-69	-0.510582124	0.829	0.911	2.36E-65
ARPC5L	1.12E-76	-0.51118775	0.519	0.778	2.11E-72
NDUFC2	3.27E-76	-0.511656236	0.951	0.953	6.17E-72
SAT1	1.21E-22	-0.512889385	0.973	0.977	2.28E-18
IGFBP2	4.47E-43	-0.515317941	0.897	0.92	8.44E-39
POMP	5.48E-90	-0.516048833	0.983	0.973	1.03E-85
TMA7	1.26E-102	-0.517587641	0.998	0.985	2.38E-98
S100A16	3.46E-40	-0.519309769	0.719	0.817	6.54E-36
GCHFR	1.07E-48	-0.522680746	0.354	0.591	2.03E-44
CKS2	2.28E-29	-0.523062248	0.576	0.726	4.30E-25
COX7B	1.04E-95	-0.524328874	0.985	0.978	1.96E-91
MYDGF	2.63E-80	-0.52449315	0.845	0.917	4.96E-76
CTSC	4.16E-116	-0.524629792	0.089	0.512	7.86E-112
SNRPF	2.11E-74	-0.525247536	0.903	0.93	3.99E-70
LIMA1	5.44E-64	-0.526493194	0.854	0.901	1.03E-59
ADIRF	3.19E-25	-0.527797631	0.149	0.313	6.02E-21
SLC25A5	9.68E-79	-0.528018788	0.982	0.975	1.83E-74
PDIA6	4.75E-70	-0.5295345	0.939	0.949	8.97E-66
TPD52	9.94E-43	-0.529782728	0.926	0.933	1.88E-38
SDCBP	1.78E-50	-0.530349537	0.789	0.875	3.37E-46
HNRNPA2B1	2.87E-56	-0.530434161	0.986	0.969	5.42E-52
CENPW	2.83E-39	-0.530920915	0.283	0.501	5.35E-35
CXCL3	2.33E-09	-0.533144127	0.533	0.623	4.40E-05
MRLN	3.46E-71	-0.534704215	0.003	0.277	6.54E-67
CCT5	6.41E-72	-0.535409307	0.765	0.882	1.21E-67
MSMO1	1.18E-51	-0.537516244	0.26	0.522	2.23E-47
FAM13A	9.51E-22	-0.539674077	0.285	0.459	1.80E-17
PRDX1	5.68E-78	-0.542137072	0.984	0.97	1.07E-73
GUK1	1.19E-87	-0.54271506	0.926	0.944	2.24E-83
EMP1	4.00E-65	-0.544502536	0.042	0.32	7.55E-61
SUB1	1.19E-103	-0.546027557	0.976	0.973	2.24E-99
GLO1	3.77E-45	-0.548922803	0.725	0.829	7.11E-41
TSTA3	5.92E-65	-0.55095828	0.704	0.852	1.12E-60
UBL5	5.62E-121	-0.55106976	0.978	0.973	1.06E-116
ANXA13	4.25E-55	-0.551481138	0.125	0.388	8.02E-51
PTMA	2.18E-110	-0.552673599	1	0.997	4.11E-106
RHOC	8.36E-65	-0.552783875	0.844	0.922	1.58E-60

NME1	5.46E-57	-0.554410251	0.766	0.84	1.03E-52
TXNDC17	8.18E-81	-0.559360231	0.848	0.914	1.55E-76
LRRC59	9.50E-99	-0.559443981	0.396	0.736	1.79E-94
C15orf48	6.54E-19	-0.565042388	0.853	0.872	1.23E-14
HMGN2	1.13E-09	-0.567859985	0.981	0.942	2.13E-05
SLC11A2	1.70E-38	-0.569093605	0.307	0.547	3.22E-34
CPS1	1.16E-68	-0.570341241	0.017	0.29	2.20E-64
ASS1	2.29E-23	-0.574371703	0.515	0.652	4.33E-19
IFITM3	1.87E-41	-0.575936499	0.927	0.93	3.53E-37
AZGP1	8.65E-88	-0.576824551	0.066	0.416	1.63E-83
CALM1	5.19E-103	-0.577092653	0.975	0.973	9.80E-99
EIF5A	1.78E-50	-0.584393812	0.915	0.92	3.36E-46
CD55	1.27E-24	-0.586200223	0.398	0.57	2.40E-20
GMDS	3.19E-92	-0.586871641	0.812	0.92	6.01E-88
TOP1	7.97E-79	-0.587793719	0.554	0.796	1.51E-74
TIMM8B	7.44E-92	-0.589571247	0.705	0.869	1.40E-87
PRELID1	3.05E-80	-0.589644637	0.955	0.951	5.76E-76
TFRC	3.02E-81	-0.59112494	0.434	0.732	5.71E-77
SPINK4	1.37E-16	-0.592693512	0.135	0.274	2.59E-12
FTL	8.06E-63	-0.593821067	0.999	0.995	1.52E-58
CYC1	5.05E-77	-0.596403854	0.956	0.952	9.54E-73
HSP90AB1	2.96E-74	-0.596703517	0.994	0.98	5.59E-70
IDI1	2.90E-49	-0.597506732	0.357	0.607	5.47E-45
ATP6V0B	1.13E-97	-0.597545152	0.757	0.897	2.12E-93
CYP2W1	1.09E-67	-0.600295758	0.051	0.333	2.06E-63
TPM3	2.65E-94	-0.600497315	0.789	0.906	5.00E-90
CXCL1	3.28E-13	-0.600586895	0.355	0.485	6.20E-09
SEC61G	1.95E-106	-0.601958994	0.953	0.965	3.68E-102
RANBP1	1.68E-39	-0.605602571	0.892	0.899	3.18E-35
PLAUR	1.40E-53	-0.608221043	0.14	0.41	2.65E-49
TRAM1	1.38E-64	-0.612789827	0.588	0.789	2.60E-60
SMIM24	1.37E-76	-0.613671107	0.045	0.353	2.58E-72
TACSTD2	2.93E-62	-0.615703767	0.026	0.284	5.54E-58
DDIT4	1.68E-18	-0.620704254	0.455	0.606	3.17E-14
PRSS3	1.71E-71	-0.621147418	0.769	0.885	3.23E-67
TXN	1.42E-147	-0.621363122	0.997	0.991	2.68E-143
MYL6	4.18E-144	-0.621493874	0.997	0.992	7.89E-140
ZNF706	1.75E-73	-0.625261114	0.904	0.932	3.30E-69
P4HB	1.14E-88	-0.626712583	0.938	0.959	2.16E-84
SLC39A4	1.60E-74	-0.629415684	0.575	0.797	3.03E-70
GSTM3	6.90E-117	-0.629971903	0.046	0.462	1.30E-112
SLC16A3	7.17E-75	-0.632799622	0.168	0.495	1.35E-70
ATP5F1E	3.27E-117	-0.636099848	0.997	0.99	6.17E-113

EIF6	4.02E-81	-0.636309681	0.841	0.914	7.60E-77
WDR1	4.71E-97	-0.644074378	0.646	0.858	8.90E-93
TMED9	5.87E-98	-0.64994832	0.827	0.914	1.11E-93
GNAS	7.38E-96	-0.65047015	0.906	0.954	1.39E-91
PDXK	2.33E-82	-0.650598828	0.553	0.788	4.40E-78
TUBA1C	1.58E-64	-0.652099597	0.609	0.818	2.98E-60
CNIH4	3.60E-87	-0.652846243	0.543	0.795	6.79E-83
TSPAN1	9.35E-38	-0.656495801	0.471	0.678	1.77E-33
ACAT2	2.11E-62	-0.66126725	0.314	0.59	3.99E-58
MT1X	1.24E-15	-0.667973813	0.513	0.641	2.35E-11
SLIRP	1.57E-112	-0.675142438	0.922	0.949	2.97E-108
FBXO2	4.04E-85	-0.676094772	0.017	0.341	7.64E-81
EZR	1.39E-71	-0.677417859	0.598	0.818	2.63E-67
CLTB	2.63E-83	-0.678540396	0.834	0.91	4.97E-79
MANF	1.08E-101	-0.679591622	0.504	0.798	2.04E-97
OAZ1	6.61E-156	-0.67998993	0.995	0.985	1.25E-151
LGALS4	2.88E-57	-0.683670358	0.998	0.986	5.43E-53
ENY2	1.65E-95	-0.687109777	0.838	0.92	3.11E-91
MYL12B	6.43E-137	-0.689667909	0.994	0.98	1.21E-132
GAPDH	1.11E-138	-0.690247862	0.999	0.997	2.10E-134
APLP2	7.67E-84	-0.692017614	0.621	0.842	1.45E-79
ELOC	6.41E-118	-0.693067034	0.775	0.905	1.21E-113
ANXA4	1.64E-75	-0.695021604	0.824	0.905	3.11E-71
ARPC2	6.02E-126	-0.698773587	0.94	0.963	1.14E-121
SOD1	7.25E-131	-0.701244267	0.986	0.979	1.37E-126
CYSTM1	6.31E-78	-0.704199294	0.912	0.955	1.19E-73
ATP5IF1	6.75E-119	-0.705758668	0.924	0.953	1.28E-114
PPIB	8.96E-130	-0.708369859	0.955	0.963	1.69E-125
RPN2	5.75E-106	-0.708390919	0.753	0.897	1.09E-101
PRDX2	1.18E-96	-0.710411681	0.971	0.956	2.23E-92
S100P	6.03E-07	-0.720816216	0.639	0.602	0.011382111
TUBB	5.81E-50	-0.721839661	0.774	0.882	1.10E-45
MTHFD2	2.84E-91	-0.72259506	0.237	0.607	5.36E-87
OLFM4	4.03E-12	-0.725146231	0.903	0.898	7.61E-08
ATP5ME	1.06E-131	-0.72647212	0.984	0.981	2.00E-127
YWHAZ	5.78E-110	-0.729942178	0.96	0.968	1.09E-105
MRPS21	5.66E-140	-0.731032948	0.133	0.606	1.07E-135
COTL1	1.00E-94	-0.73201103	0.348	0.674	1.89E-90
UQCRQ	1.78E-165	-0.732703483	0.992	0.985	3.36E-161
ATP5MD	8.36E-170	-0.741575178	0.985	0.982	1.58E-165
C4orf3	2.55E-66	-0.744483357	0.878	0.935	4.81E-62
CIB1	7.80E-118	-0.744844604	0.768	0.919	1.47E-113
BSG	2.61E-138	-0.747880622	0.945	0.964	4.92E-134

CEACAM6	1.11E-41	-0.74854358	0.136	0.384	2.10E-37
ATP1B1	2.73E-48	-0.749440994	0.987	0.979	5.16E-44
MAL2	2.51E-83	-0.750519054	0.731	0.873	4.74E-79
TM4SF1	1.23E-12	-0.765418048	0.638	0.708	2.32E-08
CALR	3.65E-119	-0.768255293	0.919	0.958	6.89E-115
FHL2	3.35E-61	-0.789253951	0.721	0.877	6.32E-57
EBP	2.87E-87	-0.794257838	0.552	0.778	5.41E-83
C12orf75	1.73E-78	-0.795639656	0.554	0.803	3.27E-74
SNRPG	1.78E-149	-0.798399465	0.912	0.955	3.37E-145
HSPA5	7.06E-89	-0.799487411	0.732	0.888	1.33E-84
TUBB4B	1.20E-61	-0.800184404	0.789	0.883	2.27E-57
PCLAF	4.31E-67	-0.801930315	0.089	0.384	8.14E-63
YWHAB	1.12E-120	-0.803626502	0.942	0.961	2.12E-116
CAPG	1.13E-136	-0.813340017	0.438	0.806	2.13E-132
TMSB4X	5.74E-129	-0.814102081	1	0.998	1.08E-124
CFL1	2.14E-198	-0.81832448	0.998	0.993	4.04E-194
FXYD5	2.23E-130	-0.819982665	0.195	0.667	4.21E-126
HSPB1	4.51E-17	-0.825770197	0.769	0.813	8.52E-13
TAGLN2	3.35E-131	-0.836432961	0.679	0.899	6.33E-127
PTTG1	9.36E-60	-0.839299724	0.082	0.357	1.77E-55
AC020656.1	1.12E-55	-0.844223763	0.114	0.389	2.11E-51
VDAC1	1.59E-142	-0.844955792	0.956	0.974	3.00E-138
PRAP1	6.44E-101	-0.855140527	0.213	0.602	1.22E-96
ROMO1	3.36E-149	-0.855584125	0.866	0.95	6.34E-145
SH3BGRL3	2.92E-106	-0.866737722	0.843	0.938	5.51E-102
HSP90AA1	6.41E-122	-0.873739238	0.997	0.984	1.21E-117
PSME2	5.77E-125	-0.88301131	0.867	0.928	1.09E-120
LGALS2	1.46E-88	-0.887226381	0.043	0.385	2.76E-84
CFD	9.50E-80	-0.887884935	0.157	0.497	1.79E-75
EIF2S2	6.19E-122	-0.889264488	0.814	0.927	1.17E-117
GSTP1	2.26E-204	-0.891198691	0.999	0.989	4.26E-200
HSPD1	7.03E-103	-0.892321014	0.968	0.961	1.33E-98
C8orf33	1.86E-71	-0.892725186	0.416	0.698	3.50E-67
RAN	1.99E-158	-0.897787383	0.962	0.971	3.75E-154
HLA-A	5.66E-81	-0.901714215	0.992	0.986	1.07E-76
PDIA3	1.75E-156	-0.910541443	0.914	0.958	3.31E-152
ISG15	1.07E-75	-0.916322869	0.235	0.57	2.02E-71
NEAT1	2.15E-55	-0.935597979	0.978	0.947	4.07E-51
LDHB	4.71E-104	-0.938289343	0.174	0.634	8.89E-100
HSP90B1	1.23E-155	-0.939659437	0.932	0.961	2.33E-151
SFN	6.75E-120	-0.939681939	0.415	0.77	1.27E-115
HSPE1	1.69E-155	-0.94636936	0.982	0.976	3.19E-151
ERO1A	1.69E-82	-0.949140933	0.42	0.723	3.18E-78

FABP5	4.15E-83	-0.952671436	0.44	0.709	7.84E-79
RAB5IF	9.35E-141	-0.963132357	0.681	0.885	1.76E-136
PSMA7	8.55E-175	-0.967352796	0.98	0.975	1.61E-170
KRT18	2.12E-133	-0.972278154	0.999	0.993	4.01E-129
DBI	1.72E-138	-0.974963984	0.954	0.961	3.24E-134
SQLE	4.54E-91	-0.979443062	0.262	0.607	8.58E-87
TPI1	2.49E-175	-0.989643746	0.997	0.984	4.71E-171
PHLDA2	1.40E-116	-0.995369127	0.378	0.748	2.65E-112
STMN1	1.20E-70	-1.007581938	0.381	0.671	2.26E-66
MALL	5.28E-132	-1.011285689	0.018	0.467	9.98E-128
LCN2	1.42E-05	-1.034288332	0.873	0.798	0.267407537
C4orf48	6.65E-161	-1.064290434	0.634	0.903	1.25E-156
ACTB	9.61E-191	-1.068231191	0.996	0.995	1.81E-186
TIMP1	9.26E-162	-1.077686817	0.496	0.867	1.75E-157
ANXA2	1.44E-168	-1.079084103	0.973	0.986	2.72E-164
IFI27	8.49E-120	-1.089450013	0.997	0.971	1.60E-115
FDPS	7.34E-106	-1.09125798	0.613	0.84	1.39E-101
LGALS3	6.24E-95	-1.091764126	0.998	0.987	1.18E-90
BST2	1.37E-118	-1.111371405	0.013	0.426	2.58E-114
KRT8	5.09E-168	-1.112596171	0.999	0.995	9.62E-164
JPT1	1.05E-181	-1.112989455	0.602	0.897	1.98E-177
IDH2	5.94E-146	-1.113466876	0.756	0.908	1.12E-141
TGFBI	5.24E-137	-1.115282221	0.333	0.76	9.90E-133
REG4	1.56E-74	-1.120407332	0.107	0.428	2.94E-70
CD74	3.42E-25	-1.123058329	0.558	0.678	6.46E-21
PGAM1	1.17E-178	-1.123644054	0.645	0.897	2.21E-174
CCND1	1.39E-140	-1.128283001	0.486	0.827	2.62E-136
IL32	3.24E-117	-1.140473569	0.704	0.912	6.11E-113
MIF	1.03E-226	-1.151545646	0.964	0.979	1.95E-222
SERPINA1	6.53E-55	-1.161083358	0.143	0.418	1.23E-50
IFI6	1.79E-61	-1.162175975	0.217	0.511	3.39E-57
S100A10	2.14E-216	-1.184052167	0.998	0.994	4.04E-212
CA9	7.28E-114	-1.186393347	0.08	0.49	1.37E-109
TUBA1B	1.90E-50	-1.189057221	0.869	0.9	3.59E-46
AGR2	6.29E-143	-1.211220613	0.976	0.984	1.19E-138
SPINK1	6.68E-84	-1.214787355	0.934	0.971	1.26E-79
NDRG1	3.06E-55	-1.21901123	0.347	0.599	5.77E-51
TMSB10	3.39E-245	-1.226996545	0.999	0.998	6.40E-241
ENO1	5.87E-189	-1.237820385	0.935	0.973	1.11E-184
PGK1	9.00E-155	-1.245107266	0.794	0.93	1.70E-150
TFF3	1.11E-06	-1.258981752	0.995	0.99	0.021003308
TNFRSF12A	3.39E-139	-1.262744747	0.272	0.698	6.39E-135
PKM	6.37E-225	-1.294881221	0.907	0.974	1.20E-220

S100A6	7.17E-159	-1.305734107	0.998	0.998	1.35E-154
CST3	8.47E-179	-1.357543165	0.968	0.982	1.60E-174
H2AFZ	2.20E-138	-1.374923173	0.883	0.954	4.16E-134
KRT19	7.19E-126	-1.385958853	0.96	0.966	1.36E-121
AREG	3.96E-56	-1.394390147	0.299	0.576	7.47E-52
SCD	3.99E-160	-1.410602905	0.191	0.678	7.53E-156
ANPEP	1.53E-91	-1.429157909	0.004	0.34	2.89E-87
KRT20	1.11E-153	-1.431525894	0.184	0.66	2.09E-149
S100A11	1.08E-262	-1.44940927	0.989	0.988	2.05E-258
FXYD3	5.18E-116	-1.452492758	0.968	0.978	9.79E-112
LDHA	6.00E-174	-1.510261762	0.957	0.976	1.13E-169
CSTB	1.28E-156	-1.540344042	0.949	0.986	2.42E-152
PLAC8	2.19E-117	-1.569084082	0.112	0.524	4.13E-113
PIGR	9.57E-15	-1.575587337	0.22	0.332	1.81E-10
PLA2G2A	3.30E-82	-1.939405608	0.196	0.545	6.23E-78
NUPR1	4.40E-136	-2.048183945	0.276	0.709	8.30E-132
TFF1	2.09E-75	-2.067322731	0.178	0.505	3.95E-71
LYZ	1.03E-125	-2.124497366	0.628	0.875	1.94E-121
CKB	5.28E-161	-2.607248161	0.744	0.927	9.97E-157
MMP7	6.42E-159	-2.867809181	0.171	0.657	1.21E-154
FABP1	2.91E-146	-3.461164303	0.134	0.607	5.49E-142
**Cluster 2**					
**Gene symbol**	**p_val**	**avg_log2FC**	**pct.1**	**pct.2**	**p_val_adj**
SELENBP1	2.10E-67	1.261641329	0.958	0.811	3.96E-63
APCDD1	1.70E-74	0.973576753	0.82	0.393	3.21E-70
DPEP1	1.65E-71	0.923008129	0.884	0.562	3.12E-67
ID3	1.39E-42	0.873510812	0.933	0.8	2.62E-38
PCCA	5.82E-60	0.853800694	0.884	0.577	1.10E-55
NKD1	7.36E-46	0.702328788	0.796	0.466	1.39E-41
NOTUM	6.94E-27	0.605739389	0.389	0.18	1.31E-22
FERMT1	2.57E-57	0.596609497	0.969	0.718	4.86E-53
CD9	9.57E-55	0.580497506	0.993	0.96	1.81E-50
ID4	2.29E-41	0.541576747	0.711	0.387	4.32E-37
LEFTY1	2.38E-33	0.5286039	0.913	0.571	4.50E-29
PRR15	6.09E-37	0.502031325	0.896	0.719	1.15E-32
SLC40A1	5.94E-28	0.49314436	0.898	0.75	1.12E-23
PHGR1	6.35E-35	0.487226558	1	0.975	1.20E-30
TSC22D1	4.04E-34	0.482853091	0.98	0.927	7.63E-30
ASCL2	7.18E-28	0.474382801	0.958	0.869	1.36E-23
REPIN1	1.41E-44	0.473563971	0.953	0.809	2.66E-40
AXIN2	1.83E-31	0.465663349	0.891	0.746	3.46E-27
PRSS23	8.30E-20	0.464662864	0.896	0.816	1.57E-15
ALDH1A1	6.12E-31	0.455690398	0.738	0.486	1.16E-26

RPL26	2.99E-48	0.453755942	1	0.995	5.64E-44
HSPA1A	6.63E-19	0.450307901	0.504	0.293	1.25E-14
CCND2	1.10E-36	0.444808442	0.889	0.611	2.08E-32
TMEM230	1.39E-44	0.442291973	0.942	0.81	2.63E-40
TESC	8.83E-44	0.436116039	0.644	0.311	1.67E-39
QTRT1	6.46E-41	0.435835983	0.947	0.766	1.22E-36
ITPR2	5.00E-31	0.428842013	0.744	0.461	9.44E-27
SLPI	2.14E-22	0.408210718	0.753	0.542	4.05E-18
CDX2	3.16E-26	0.40815203	0.933	0.83	5.97E-22
ID1	3.41E-15	0.40712697	0.962	0.922	6.44E-11
GSTK1	4.26E-39	0.40469418	0.962	0.917	8.05E-35
AGR3	7.53E-26	0.40428639	0.953	0.857	1.42E-21
RPS4X	9.75E-55	0.396026525	1	0.994	1.84E-50
TPT1	6.97E-58	0.387583403	1	0.998	1.32E-53
RNF43	3.85E-30	0.386526444	0.964	0.857	7.26E-26
RAB32	3.11E-42	0.382197485	0.809	0.466	5.88E-38
CLDN3	1.72E-27	0.381382977	0.989	0.975	3.25E-23
FAM3D	4.18E-25	0.380048895	0.973	0.903	7.90E-21
RPS5	8.29E-39	0.377909191	1	0.988	1.57E-34
CD99	3.00E-27	0.371378349	0.976	0.9	5.67E-23
PYCARD	1.06E-30	0.367318468	0.882	0.697	2.00E-26
COMMD6	3.11E-41	0.364584713	0.993	0.964	5.88E-37
GOLIM4	2.42E-17	0.364476359	0.973	0.883	4.56E-13
SMAD9	6.18E-38	0.363204914	0.662	0.359	1.17E-33
C10orf99	8.22E-26	0.360395834	0.944	0.74	1.55E-21
NPDC1	3.01E-26	0.359704591	0.958	0.918	5.68E-22
RAB11FIP1	9.17E-27	0.358052577	0.909	0.758	1.73E-22
HOXB9	3.18E-35	0.356240595	0.842	0.615	6.01E-31
TBX3	1.46E-14	0.354239861	0.707	0.605	2.77E-10
NDUFA4	4.99E-34	0.34850045	0.996	0.984	9.43E-30
MSRB2	1.32E-22	0.348172044	0.924	0.833	2.49E-18
SLC12A2	2.20E-16	0.344598922	0.989	0.961	4.15E-12
LAMTOR4	5.17E-32	0.342108024	0.989	0.934	9.77E-28
GAS6	1.13E-26	0.338878906	0.876	0.714	2.14E-22
MT-CO3	1.09E-21	0.335591319	1	0.992	2.05E-17
SRI	1.80E-28	0.332155802	0.989	0.965	3.40E-24
BTG2	8.99E-21	0.326571852	0.936	0.786	1.70E-16
SEMA3C	4.04E-29	0.325491781	0.773	0.541	7.62E-25
IGKC	5.79E-16	0.324445102	0.798	0.655	1.09E-11
KRTCAP3	9.46E-28	0.32165316	0.944	0.844	1.79E-23
ITGA6	7.29E-18	0.32022562	0.969	0.913	1.38E-13
SEMA5A	3.52E-26	0.319982345	0.758	0.541	6.65E-22
ZNF703	1.32E-23	0.317992915	0.88	0.723	2.49E-19

RPL17	2.45E-26	0.317124682	0.996	0.95	4.63E-22
NTRK2	5.78E-25	0.312388685	0.387	0.186	1.09E-20
MUC3A	3.14E-25	0.310279252	0.836	0.66	5.93E-21
TRIM31	2.82E-23	0.310187668	0.584	0.347	5.32E-19
EIF3L	4.72E-30	0.309917716	0.98	0.919	8.92E-26
FIS1	4.60E-26	0.30827621	0.976	0.917	8.69E-22
TNRC6B	2.27E-27	0.307532913	0.8	0.531	4.29E-23
GPX2	1.13E-19	0.306717758	0.998	0.961	2.14E-15
RPL21	4.11E-43	0.306715249	1	0.996	7.76E-39
ID2	1.11E-10	0.306606016	0.889	0.82	2.09E-06
PRDX5	4.84E-23	0.305888283	0.998	0.981	9.14E-19
AC020916.1	7.10E-18	0.304769317	0.649	0.479	1.34E-13
TMEM9	2.38E-22	0.304750557	0.751	0.571	4.49E-18
BACE2	6.08E-20	0.303924439	0.944	0.857	1.15E-15
NIPSNAP2	1.31E-27	0.300538586	0.887	0.726	2.48E-23
TSTD1	6.80E-28	0.299927322	0.964	0.895	1.28E-23
S100A14	9.30E-28	0.29885594	0.971	0.864	1.76E-23
EEF1A1	3.61E-44	0.297028623	1	0.997	6.81E-40
HTATIP2	1.31E-25	0.296076134	0.911	0.79	2.47E-21
MUC12	1.61E-10	0.295926757	0.776	0.665	3.04E-06
GTF2I	1.36E-22	0.294793716	0.942	0.854	2.56E-18
ACAA2	5.66E-18	0.294278012	0.893	0.785	1.07E-13
IFT57	1.10E-22	0.29344829	0.8	0.651	2.08E-18
ALDH2	5.10E-14	0.287503705	0.964	0.91	9.63E-10
H3F3A	2.09E-24	0.285521185	1	0.994	3.95E-20
TRAPPC1	1.55E-24	0.28521599	0.907	0.796	2.93E-20
ATP5F1A	9.49E-22	0.284768937	0.978	0.913	1.79E-17
EBPL	1.60E-21	0.28241021	0.933	0.846	3.03E-17
MUC5B	8.29E-28	0.282030512	0.6	0.335	1.57E-23
ESD	2.07E-20	0.281901912	0.971	0.875	3.90E-16
THRA	1.78E-21	0.281383823	0.711	0.533	3.37E-17
RPS6	1.28E-33	0.281290968	1	0.996	2.43E-29
GALNT6	7.47E-25	0.279265357	0.667	0.443	1.41E-20
C7orf50	2.31E-18	0.278030627	0.842	0.712	4.37E-14
N4BP2L2	5.92E-24	0.278013782	0.958	0.876	1.12E-19
HIPK2	2.82E-18	0.277390873	0.671	0.508	5.32E-14
HOXA9	4.06E-16	0.276908937	0.753	0.614	7.66E-12
C9orf152	1.92E-24	0.276501279	0.771	0.566	3.62E-20
NOP53	7.87E-26	0.274919031	0.991	0.959	1.49E-21
SMIM19	1.78E-25	0.271553738	0.816	0.624	3.36E-21
SRPK1	7.38E-13	0.270961887	0.942	0.859	1.39E-08
CYBA	6.52E-21	0.267417223	0.987	0.958	1.23E-16
ARGLU1	4.83E-18	0.264022	0.951	0.863	9.12E-14

TXNIP	6.12E-16	0.263549921	0.824	0.632	1.16E-11
IGHA1	3.15E-12	0.261156308	0.707	0.562	5.95E-08
RPL10A	1.84E-34	0.259532753	1	0.996	3.47E-30
SARAF	1.35E-21	0.259105218	0.969	0.884	2.55E-17
POLR2J3.1	4.36E-18	0.257783748	0.849	0.705	8.24E-14
NQO1	3.17E-12	0.257214044	0.909	0.82	5.99E-08
RPL3	1.05E-32	0.256475873	1	0.998	1.98E-28
MT-CO2	5.81E-17	0.256435833	1	0.99	1.10E-12
GTF3A	9.67E-14	0.255375265	0.918	0.858	1.83E-09
BRI3	4.10E-17	0.254171623	0.949	0.876	7.74E-13
DNAJC15	4.29E-17	0.253804791	0.982	0.931	8.11E-13
IFITM2	7.16E-19	0.252575955	0.724	0.545	1.35E-14
RPL15	2.25E-38	0.251395204	1	0.996	4.25E-34
MRPS33	2.65E-19	0.251368812	0.96	0.878	5.01E-15
UBE2H	1.60E-13	0.250955542	0.729	0.626	3.02E-09
HIBADH	1.81E-22	0.250701833	0.8	0.623	3.42E-18
CD44	1.33E-12	0.250636674	0.964	0.891	2.50E-08
FUOM	2.10E-13	-0.25006601	0.258	0.407	3.96E-09
UQCRQ	9.13E-11	-0.25080103	1	0.985	1.72E-06
HIGD1A	1.29E-08	-0.251039319	0.7	0.737	0.000242762
IER2	4.13E-05	-0.251295559	0.907	0.901	0.779205885
DSC2	6.52E-08	-0.251504542	0.713	0.767	0.001231401
TIMM17A	2.49E-11	-0.251733962	0.587	0.662	4.70E-07
ARPC5L	8.60E-11	-0.251856493	0.656	0.719	1.62E-06
LINC01133	9.88E-09	-0.252102033	0.247	0.368	0.00018662
PLIN2	1.02E-06	-0.252536886	0.456	0.558	0.01926598
SLC52A2	7.56E-07	-0.252683946	0.653	0.67	0.014277119
WEE1	5.83E-06	-0.254204617	0.36	0.454	0.110147222
SNRPG	3.45E-06	-0.255427154	0.971	0.94	0.065220075
KIF5B	4.44E-09	-0.255731672	0.971	0.949	8.39E-05
SMIM31	6.56E-10	-0.25658187	0.333	0.465	1.24E-05
MYL6	1.32E-12	-0.257124835	1	0.992	2.49E-08
GRPEL1	2.33E-10	-0.257986508	0.527	0.604	4.40E-06
DYNLRB1	3.51E-08	-0.258033821	0.896	0.894	0.000663146
SEPHS2	6.21E-08	-0.258452026	0.727	0.761	0.001172485
SLIRP	2.96E-08	-0.259900045	0.967	0.939	0.000558872
PHF20L1	2.41E-11	-0.259958276	0.511	0.615	4.56E-07
LACTB2	1.70E-09	-0.260181351	0.46	0.558	3.21E-05
CFL1	5.64E-09	-0.260226414	1	0.993	0.000106545
TSPAN3	7.53E-08	-0.26031942	0.816	0.825	0.001422253
PRELID3B	6.31E-08	-0.260767361	0.753	0.775	0.001190531
IER3	0.000153004	-0.260946106	0.602	0.67	1
GFPT1	2.32E-10	-0.262826448	0.66	0.733	4.37E-06

F3	0.000135915	-0.262904297	0.278	0.359	1
MAD2L1	5.36E-09	-0.263192329	0.149	0.264	0.000101282
PPP2R1A	3.22E-11	-0.264096425	0.833	0.846	6.08E-07
ALDOA	2.42E-10	-0.264516083	0.52	0.618	4.57E-06
CKS2	0.015584154	-0.264707576	0.713	0.684	1
CLDN2	0.003262774	-0.266312206	0.558	0.596	1
P4HB	2.55E-06	-0.267156895	0.982	0.949	0.048101852
HSPA1B	0.001316692	-0.267615018	0.542	0.615	1
LIMA1	1.48E-08	-0.267878483	0.909	0.886	0.000279008
PPIB	2.07E-09	-0.267911861	0.993	0.956	3.92E-05
IQGAP2	6.02E-18	-0.268186031	0.2	0.403	1.14E-13
LRPAP1	1.71E-09	-0.269074129	0.742	0.755	3.22E-05
USP53	0.000189446	-0.269079005	0.471	0.526	1
TRABD2A	1.41E-14	-0.270027084	0.413	0.578	2.66E-10
EGLN3	6.73E-10	-0.270421653	0.278	0.407	1.27E-05
MTDH	9.71E-12	-0.2717522	0.936	0.926	1.83E-07
SLC1A5	4.80E-13	-0.272004446	0.342	0.499	9.07E-09
MYC	2.34E-06	-0.27223835	0.622	0.687	0.044271268
TAF7	3.63E-08	-0.272930592	0.844	0.845	0.000686028
C1orf21	9.92E-18	-0.273382147	0.293	0.495	1.87E-13
NPM1	4.25E-09	-0.275671169	0.998	0.985	8.02E-05
ACOT7	5.89E-13	-0.276543546	0.291	0.442	1.11E-08
SELENOS	7.34E-09	-0.27673202	0.813	0.833	0.000138583
CTSV	5.64E-17	-0.277277236	0.087	0.262	1.07E-12
CALM2	1.56E-09	-0.279302859	0.996	0.98	2.95E-05
EXOSC4	4.65E-14	-0.279793927	0.48	0.594	8.79E-10
HSP90AB1	6.25E-11	-0.280612906	1	0.981	1.18E-06
KPNA2	9.46E-08	-0.280761029	0.242	0.356	0.001787053
FAM49B	1.56E-11	-0.281149981	0.531	0.63	2.95E-07
PSAT1	5.33E-19	-0.281255415	0.089	0.286	1.01E-14
CACYBP	7.24E-09	-0.281547975	0.751	0.773	0.000136618
LMO4	4.22E-19	-0.282924341	0.142	0.349	7.97E-15
MT1E	2.44E-06	-0.283408492	0.176	0.284	0.046099461
MZT2B	2.62E-16	-0.283426266	0.978	0.963	4.96E-12
RHOC	3.66E-07	-0.283493331	0.922	0.899	0.006913208
S100A6	0.622517039	-0.284323834	1	0.998	1
AKAP7	3.77E-15	-0.285637295	0.113	0.281	7.11E-11
TFRC	3.77E-06	-0.286201847	0.651	0.656	0.071179682
MARCKS	9.45E-09	-0.28631318	0.853	0.865	0.000178475
IDI1	0.001897018	-0.286377376	0.52	0.545	1
CYSTM1	0.00235188	-0.286685685	0.964	0.941	1
TK1	1.74E-12	-0.287087672	0.12	0.264	3.28E-08
MYL12B	1.25E-09	-0.287139366	0.991	0.983	2.37E-05

CYP2S1	3.20E-17	-0.287207236	0.169	0.364	6.04E-13
CFDP1	6.46E-11	-0.289268678	0.72	0.787	1.22E-06
FCGBP	0.053234207	-0.29013672	0.262	0.306	1
SCAND1	1.23E-12	-0.290436841	0.882	0.882	2.33E-08
MCL1	3.02E-12	-0.290770105	0.613	0.723	5.70E-08
UGDH	3.83E-10	-0.291266157	0.58	0.651	7.23E-06
TMA7	4.40E-18	-0.291668949	0.996	0.988	8.31E-14
DPM1	1.85E-15	-0.293086076	0.56	0.689	3.49E-11
RBM39	6.35E-10	-0.29342911	0.964	0.939	1.20E-05
PDIA6	3.84E-11	-0.29470154	0.964	0.944	7.26E-07
ADIRF	0.004244421	-0.296919733	0.222	0.278	1
PFKP	3.16E-17	-0.298131694	0.291	0.472	5.96E-13
PUF60	1.02E-10	-0.298409677	0.731	0.736	1.93E-06
MSMO1	1.33E-06	-0.299824511	0.369	0.467	0.025068357
KRT10	3.38E-15	-0.300068552	0.753	0.809	6.39E-11
TSTA3	4.91E-09	-0.300608787	0.831	0.812	9.26E-05
KLF6	6.65E-05	-0.303066007	0.871	0.848	1
GSTP1	5.23E-10	-0.303290313	0.998	0.991	9.87E-06
PRELID1	2.67E-09	-0.305351964	0.973	0.949	5.04E-05
TIMM8B	1.57E-11	-0.306321638	0.844	0.824	2.97E-07
VEGFA	8.66E-08	-0.306911875	0.396	0.513	0.001634419
FCGRT	4.96E-08	-0.308232037	0.893	0.854	0.000936354
TAGLN2	1.00E-07	-0.3083806	0.856	0.841	0.001897291
TPM1	3.67E-17	-0.308410141	0.951	0.956	6.92E-13
AGO2	1.87E-17	-0.310838638	0.371	0.538	3.53E-13
CES2	2.34E-08	-0.315115097	0.391	0.507	0.000441592
P4HA1	1.28E-15	-0.316334256	0.218	0.395	2.41E-11
CTSC	1.65E-27	-0.316775873	0.171	0.435	3.11E-23
SDCBP	1.57E-09	-0.316824946	0.851	0.854	2.96E-05
SLC2A1	6.81E-12	-0.318250579	0.158	0.299	1.29E-07
PTGR1	4.60E-11	-0.32187651	0.476	0.585	8.68E-07
UBE2V2	5.91E-16	-0.322912899	0.689	0.758	1.12E-11
NDUFB9	7.63E-17	-0.323396973	0.993	0.961	1.44E-12
ADM	0.000471469	-0.323583277	0.193	0.263	1
ATP5MD	7.63E-19	-0.325751075	0.989	0.982	1.44E-14
RPN2	5.03E-10	-0.326543383	0.873	0.858	9.49E-06
PRSS22	1.90E-11	-0.328676844	0.187	0.328	3.58E-07
ACAT2	3.15E-07	-0.3298009	0.444	0.529	0.005951907
ATP5F1E	1.10E-14	-0.330291367	0.998	0.991	2.08E-10
GAPDH	1.06E-09	-0.330622099	1	0.997	2.00E-05
HSPB1	0.568041257	-0.330630098	0.851	0.795	1
FTL	2.73E-12	-0.333002217	1	0.995	5.15E-08
LGALS4	0.000170654	-0.334700563	0.998	0.988	1

SLC6A8	8.41E-12	-0.335739381	0.136	0.284	1.59E-07
CEACAM1	7.41E-08	-0.339571599	0.278	0.386	0.001399577
ANXA5	1.29E-17	-0.341006987	0.387	0.58	2.44E-13
ARL6IP1	4.52E-07	-0.341086827	0.902	0.88	0.008536225
ACTB	1.20E-09	-0.341408458	1	0.994	2.27E-05
WDR1	4.91E-12	-0.342262281	0.789	0.806	9.28E-08
RAD21	2.75E-12	-0.344403996	0.633	0.724	5.20E-08
VDAC2	6.14E-14	-0.347600149	0.967	0.948	1.16E-09
KRT18	9.07E-07	-0.34997224	0.993	0.995	0.017129948
EMP1	2.45E-06	-0.350199512	0.167	0.26	0.046220342
TMED9	9.10E-13	-0.353075494	0.9	0.89	1.72E-08
RAN	1.09E-09	-0.354455022	0.991	0.965	2.05E-05
ASPH	2.19E-14	-0.356177441	0.742	0.784	4.13E-10
BSG	2.04E-13	-0.358996373	0.973	0.957	3.85E-09
GLO1	5.51E-09	-0.361871266	0.809	0.801	0.000104012
CEBPD	8.95E-10	-0.363187163	0.718	0.778	1.69E-05
IL32	0.062928866	-0.363648042	0.896	0.854	1
EZR	4.86E-08	-0.365047709	0.733	0.765	0.000917265
CAPG	9.14E-13	-0.366764693	0.644	0.72	1.72E-08
FHL2	0.002008045	-0.372891229	0.869	0.832	1
TUBA1C	2.19E-09	-0.374191339	0.742	0.767	4.13E-05
ATP5ME	9.21E-21	-0.374806238	1	0.979	1.74E-16
PDXK	9.09E-10	-0.375070027	0.724	0.728	1.72E-05
TUBB	2.08E-06	-0.379843702	0.86	0.853	0.03921526
SLC11A2	0.001210948	-0.379917703	0.449	0.49	1
NPC2	8.95E-21	-0.381592014	0.94	0.935	1.69E-16
PDIA3	5.71E-12	-0.381712766	0.98	0.942	1.08E-07
CD55	0.004630889	-0.382575603	0.489	0.531	1
EEF1D	2.98E-23	-0.383002436	1	0.987	5.62E-19
AZGP1	3.56E-22	-0.385138657	0.127	0.354	6.72E-18
PRSS8	8.10E-13	-0.385762436	0.569	0.671	1.53E-08
CEACAM6	1.87E-12	-0.38638243	0.167	0.341	3.53E-08
TOP1	2.74E-16	-0.3882984	0.667	0.743	5.18E-12
PRSS3	2.27E-13	-0.390174855	0.844	0.857	4.29E-09
TSPAN1	0.00239335	-0.391888884	0.631	0.624	1
ATP5IF1	3.46E-20	-0.394773727	0.956	0.944	6.53E-16
ELOC	3.31E-17	-0.395390709	0.869	0.872	6.26E-13
GPRC5A	5.70E-07	-0.395892609	0.738	0.771	0.010753338
ZNF706	2.40E-12	-0.396067055	0.933	0.924	4.53E-08
CEACAM5	2.28E-13	-0.397410794	0.856	0.895	4.30E-09
PSMA7	3.90E-10	-0.40008321	0.991	0.974	7.36E-06
XBP1	4.76E-20	-0.400934663	0.804	0.85	8.99E-16
OAZ1	4.44E-29	-0.401125061	0.991	0.987	8.39E-25

AKR1B10	1.86E-26	-0.402513189	0.051	0.282	3.51E-22
MANF	2.59E-16	-0.40332249	0.66	0.731	4.90E-12
YWHAB	8.34E-13	-0.404086807	0.984	0.952	1.57E-08
FABP5	0.00146439	-0.404159444	0.658	0.637	1
KRT8	2.10E-06	-0.404315546	0.998	0.996	0.039671177
PLAUR	2.41E-05	-0.404996068	0.264	0.351	0.45481918
ANXA4	4.26E-12	-0.405265783	0.9	0.882	8.04E-08
ZFAS1	2.12E-15	-0.407804699	0.993	0.977	4.01E-11
TGFBI	0.00430562	-0.408208163	0.656	0.649	1
CYC1	5.21E-19	-0.408255722	0.971	0.95	9.84E-15
CEBPB	1.06E-12	-0.414080265	0.707	0.771	1.99E-08
SOD1	1.72E-22	-0.41697351	0.991	0.979	3.26E-18
GNAS	3.41E-20	-0.418719967	0.942	0.942	6.43E-16
SAT1	1.44E-08	-0.420963415	0.987	0.974	0.000271456
IFI27	0.005689443	-0.423217079	0.993	0.975	1
GCHFR	1.40E-15	-0.425015572	0.402	0.547	2.65E-11
VDAC1	1.77E-15	-0.428403753	0.98	0.968	3.34E-11
MRPS21	7.23E-34	-0.429221192	0.213	0.522	1.37E-29
CNIH4	4.82E-18	-0.431138972	0.667	0.739	9.10E-14
YWHAZ	3.14E-16	-0.431713817	0.984	0.964	5.93E-12
TCEAL9	2.61E-28	-0.433257082	0.253	0.525	4.93E-24
DDIT4	0.003529129	-0.439163183	0.524	0.573	1
SLC39A4	4.04E-17	-0.439175796	0.68	0.748	7.64E-13
CIB1	2.91E-17	-0.439526226	0.889	0.879	5.50E-13
HMGB2	0.01197656	-0.441974026	0.609	0.59	1
MT1X	0.001188334	-0.444607791	0.587	0.611	1
HSPE1	2.01E-14	-0.447390877	0.989	0.976	3.80E-10
SFN	7.39E-12	-0.449589416	0.611	0.688	1.39E-07
AGR2	1.44E-05	-0.449641217	0.991	0.981	0.271957426
EBP	8.45E-12	-0.450660606	0.698	0.723	1.59E-07
ANXA13	3.02E-31	-0.451182418	0.08	0.354	5.70E-27
HMGN2	4.24E-07	-0.45166342	0.969	0.95	0.008009406
MT2A	2.80E-06	-0.453356589	0.336	0.432	0.052811069
HSPA5	6.24E-11	-0.46274155	0.878	0.843	1.18E-06
HSP90AA1	9.86E-17	-0.463983664	0.998	0.986	1.86E-12
NEAT1	0.00050971	-0.466856131	0.984	0.951	1
ROMO1	5.14E-19	-0.467106833	0.94	0.926	9.71E-15
MTHFD2	4.93E-19	-0.470434101	0.356	0.533	9.31E-15
HSP90B1	7.47E-19	-0.478667919	0.956	0.953	1.41E-14
C4orf3	3.79E-09	-0.479460612	0.951	0.916	7.15E-05
TIMP1	7.23E-12	-0.482616634	0.742	0.776	1.36E-07
COTL1	4.36E-19	-0.484477005	0.464	0.607	8.24E-15
PRAP1	2.19E-11	-0.485600079	0.387	0.518	4.13E-07

TPD52	1.22E-23	-0.487641289	0.931	0.931	2.30E-19
DBI	6.64E-14	-0.489914457	0.982	0.956	1.25E-09
SMIM24	3.43E-26	-0.491757003	0.069	0.302	6.47E-22
CYP2W1	4.31E-25	-0.492289913	0.062	0.288	8.14E-21
ENY2	2.27E-28	-0.492982253	0.902	0.899	4.29E-24
PKM	1.36E-11	-0.493305869	0.973	0.955	2.56E-07
TRAM1	8.82E-23	-0.49341075	0.647	0.75	1.67E-18
FAM13A	6.23E-09	-0.493833736	0.311	0.428	0.00011765
HSPD1	4.04E-12	-0.500464528	0.987	0.959	7.63E-08
APLP2	1.83E-21	-0.500571456	0.727	0.793	3.46E-17
LGALS3	0.026253093	-0.501954917	0.996	0.989	1
MAL2	9.61E-17	-0.503326018	0.811	0.84	1.81E-12
EIF6	1.13E-26	-0.504101713	0.887	0.897	2.14E-22
PTTG1	5.93E-10	-0.504851144	0.171	0.302	1.12E-05
SLC16A3	6.66E-21	-0.504893707	0.233	0.436	1.26E-16
PRDX2	3.46E-26	-0.512503156	0.964	0.959	6.53E-22
GSTM3	1.20E-40	-0.518916766	0.078	0.393	2.27E-36
C12orf75	1.14E-16	-0.526177638	0.651	0.751	2.16E-12
TUBB4B	6.81E-13	-0.529687864	0.833	0.863	1.29E-08
TPI1	6.30E-22	-0.549746089	0.998	0.985	1.19E-17
PCLAF	5.15E-12	-0.552670456	0.182	0.326	9.72E-08
AC020656.1	3.96E-06	-0.557405578	0.231	0.33	0.074710376
FBXO2	3.46E-32	-0.563955219	0.029	0.289	6.54E-28
JPT1	6.52E-21	-0.572338545	0.811	0.822	1.23E-16
FDPS	1.88E-08	-0.573554856	0.789	0.78	0.000354407
MIF	2.79E-30	-0.578309503	0.98	0.975	5.27E-26
ENO1	1.04E-14	-0.582239687	0.987	0.96	1.97E-10
PGAM1	2.50E-18	-0.590517456	0.858	0.829	4.71E-14
FXYD5	4.88E-35	-0.591166345	0.291	0.581	9.22E-31
CFD	3.63E-11	-0.600432137	0.293	0.426	6.84E-07
TM4SF1	4.05E-06	-0.605468708	0.651	0.695	0.076450967
RAB5IF	4.17E-22	-0.609980302	0.816	0.835	7.87E-18
EIF2S2	2.35E-29	-0.610089072	0.896	0.898	4.44E-25
SQLE	1.36E-13	-0.613687204	0.402	0.535	2.56E-09
REG4	7.17E-07	-0.618065005	0.249	0.359	0.013531395
TUBA1B	0.000382519	-0.619516111	0.907	0.89	1
PHLDA2	6.55E-18	-0.624337769	0.544	0.667	1.24E-13
S100A10	4.01E-24	-0.634637042	0.998	0.994	7.57E-20
CCND1	3.17E-17	-0.63956188	0.716	0.742	5.99E-13
KRT19	1.88E-07	-0.642366673	0.98	0.962	0.003542254
C4orf48	2.79E-28	-0.66263785	0.8	0.838	5.26E-24
LDHB	3.79E-31	-0.674778869	0.222	0.556	7.16E-27
ERO1A	4.50E-12	-0.697343336	0.573	0.655	8.50E-08

ISG15	1.51E-15	-0.697413633	0.342	0.504	2.86E-11
S100A11	5.87E-22	-0.710602064	0.989	0.989	1.11E-17
C8orf33	1.03E-15	-0.733405359	0.544	0.636	1.95E-11
CST3	1.91E-12	-0.736341722	0.993	0.977	3.60E-08
LGALS2	1.74E-29	-0.751342482	0.073	0.328	3.29E-25
STMN1	4.61E-25	-0.765765545	0.411	0.622	8.71E-21
IFI6	3.98E-11	-0.774849261	0.309	0.452	7.52E-07
PGK1	6.23E-23	-0.788955672	0.9	0.894	1.18E-18
H2AFZ	9.44E-19	-0.789267707	0.947	0.934	1.78E-14
IDH2	3.34E-34	-0.792958401	0.851	0.871	6.31E-30
LDHA	1.38E-14	-0.807086038	0.987	0.969	2.61E-10
SERPINA1	6.09E-12	-0.832007339	0.204	0.367	1.15E-07
MALL	1.01E-41	-0.841246388	0.069	0.39	1.91E-37
TMSB10	5.92E-65	-0.858284002	1	0.998	1.12E-60
KRT20	1.13E-13	-0.859427882	0.458	0.548	2.14E-09
TNFRSF12A	1.70E-24	-0.860638681	0.436	0.61	3.21E-20
OLFM4	1.68E-15	-0.872262528	0.858	0.905	3.17E-11
PLA2G2A	0.000146183	-0.8723046	0.398	0.463	1
BST2	7.26E-37	-0.902734223	0.06	0.355	1.37E-32
SCD	2.31E-20	-0.904485701	0.407	0.572	4.36E-16
SPINK1	8.04E-24	-0.930530485	0.942	0.964	1.52E-19
PIGR	0.012042474	-0.944299531	0.271	0.308	1
CA9	1.31E-29	-0.967017833	0.169	0.414	2.48E-25
AREG	1.40E-14	-1.012655069	0.369	0.523	2.63E-10
FXYD3	3.10E-19	-1.014096469	0.982	0.975	5.86E-15
CSTB	2.48E-20	-1.047796885	0.993	0.975	4.68E-16
NDRG1	5.47E-23	-1.106961976	0.387	0.555	1.03E-18
ANPEP	7.43E-32	-1.193889875	0.027	0.285	1.40E-27
PLAC8	1.13E-33	-1.245150974	0.173	0.452	2.14E-29
TFF3	7.22E-14	-1.27834179	0.998	0.99	1.36E-09
LYZ	2.91E-13	-1.302653034	0.802	0.813	5.49E-09
CKB	1.25E-20	-1.632183908	0.86	0.883	2.36E-16
TFF1	6.34E-11	-1.6550314	0.307	0.436	1.20E-06
NUPR1	3.90E-53	-1.671462086	0.278	0.642	7.37E-49
MMP7	3.22E-29	-2.158419169	0.34	0.559	6.09E-25
FABP1	2.97E-36	-2.695378799	0.238	0.52	5.61E-32
**Cluster 3**					
**Gene symbol**	**p_val**	**avg_log2FC**	**pct.1**	**pct.2**	**p_val_adj**
PIGR	1.35E-127	2.752026113	0.638	0.232	2.54E-123
CD74	5.42E-114	1.526595048	0.865	0.6	1.02E-109
SPINK4	6.77E-46	1.376870144	0.443	0.194	1.28E-41
LCN2	3.33E-85	1.329052681	0.909	0.797	6.28E-81
PRAC1	1.06E-125	0.938575962	0.529	0.118	2.00E-121

CXCL2	5.14E-31	0.856708228	0.603	0.404	9.70E-27
PLA2G2A	1.32E-50	0.841360561	0.677	0.408	2.49E-46
HLA-B	2.35E-94	0.809603868	0.974	0.987	4.44E-90
CLCA1	2.10E-66	0.797279458	0.271	0.056	3.96E-62
S100P	4.47E-76	0.781796498	0.851	0.56	8.45E-72
HLA-A	2.40E-72	0.751697264	0.977	0.99	4.53E-68
L1TD1	1.20E-61	0.718550162	0.694	0.375	2.27E-57
UGT2B17	5.92E-103	0.705919206	0.371	0.073	1.12E-98
MT-ND5	3.19E-61	0.686282838	0.971	0.973	6.02E-57
PSMB9	8.08E-61	0.674789026	0.755	0.522	1.53E-56
HLA-DPB1	3.04E-116	0.67213934	0.411	0.08	5.75E-112
MT1E	2.16E-46	0.666682125	0.488	0.224	4.08E-42
MT-CO2	1.80E-84	0.665256289	0.995	0.99	3.40E-80
MT-CYB	1.37E-55	0.664436383	0.986	0.987	2.59E-51
B2M	6.37E-79	0.662591814	0.995	0.999	1.20E-74
CXCL3	5.50E-25	0.659977495	0.738	0.57	1.04E-20
MT-ND2	5.95E-51	0.641132136	0.988	0.989	1.12E-46
HLA-DPA1	5.12E-123	0.63459632	0.438	0.086	9.68E-119
SOD3	3.53E-71	0.631128991	0.578	0.235	6.67E-67
MT-CO3	1.69E-71	0.63042997	0.994	0.993	3.18E-67
BEX3	1.49E-115	0.625910014	0.482	0.109	2.81E-111
MT-ATP6	1.06E-62	0.6226468	0.982	0.987	2.00E-58
APIP	5.83E-44	0.57479571	0.791	0.678	1.10E-39
MT-ND3	3.28E-32	0.562161776	0.989	0.987	6.20E-28
LDHB	6.12E-22	0.55817873	0.629	0.491	1.16E-17
RNASE1	6.95E-58	0.549911016	0.474	0.199	1.31E-53
MT-CO1	4.56E-52	0.54246504	0.991	0.993	8.60E-48
FABP5	1.50E-36	0.53733579	0.803	0.604	2.83E-32
C10orf99	1.98E-34	0.531692496	0.869	0.743	3.74E-30
CXCL1	1.99E-21	0.528223052	0.6	0.42	3.76E-17
HLA-C	5.23E-57	0.526321362	0.972	0.987	9.87E-53
CASP1	1.32E-41	0.495879241	0.665	0.436	2.50E-37
HLA-DMA	9.11E-80	0.487738788	0.468	0.153	1.72E-75
MUC4	8.15E-58	0.487679401	0.642	0.323	1.54E-53
SELENBP1	1.10E-27	0.487527962	0.915	0.81	2.08E-23
ADH1C	3.93E-16	0.486197895	0.54	0.419	7.41E-12
MT-ND6	1.07E-33	0.484095395	0.828	0.695	2.03E-29
ATP5MC1	2.60E-48	0.480582617	0.935	0.909	4.90E-44
FCGBP	1.10E-13	0.458349412	0.415	0.276	2.08E-09
PSMB8	3.28E-47	0.45575774	0.898	0.839	6.19E-43
MUC2	3.19E-19	0.450224174	0.275	0.136	6.03E-15
MT2A	1.34E-18	0.443921709	0.552	0.392	2.53E-14
PSME2	5.46E-34	0.443176737	0.931	0.908	1.03E-29

IER3	2.30E-22	0.434794083	0.78	0.636	4.35E-18
RPS27L	2.25E-39	0.43359185	0.954	0.975	4.25E-35
MT1G	1.27E-12	0.424040196	0.3	0.185	2.40E-08
HLA-F	2.08E-39	0.423843694	0.669	0.454	3.92E-35
ITM2C	4.60E-15	0.423274724	0.794	0.765	8.68E-11
FAM3D	3.30E-31	0.423084808	0.92	0.91	6.23E-27
GOLIM4	5.47E-30	0.421228077	0.934	0.885	1.03E-25
C1QBP	8.96E-41	0.420051178	0.934	0.9	1.69E-36
MUC1	1.31E-43	0.414862369	0.708	0.441	2.48E-39
IFI16	1.20E-62	0.409206546	0.398	0.132	2.27E-58
NAA38	5.78E-36	0.408452833	0.862	0.803	1.09E-31
CKMT1A	7.69E-54	0.403660995	0.594	0.32	1.45E-49
GPX2	3.37E-34	0.398749014	0.974	0.963	6.37E-30
HLA-E	1.43E-31	0.396366453	0.923	0.91	2.70E-27
PFN1	4.43E-42	0.395985431	0.974	0.988	8.36E-38
FERMT1	1.39E-28	0.394143006	0.838	0.73	2.62E-24
MT-ND4	2.58E-38	0.393490119	0.991	0.993	4.86E-34
CTSS	1.26E-22	0.387440051	0.818	0.757	2.37E-18
STAT1	4.41E-21	0.382734145	0.54	0.381	8.33E-17
VSIG2	1.58E-42	0.380050693	0.623	0.348	2.98E-38
ACADVL	1.61E-26	0.372759267	0.882	0.836	3.05E-22
CCL20	5.69E-09	0.368839876	0.448	0.335	0.000107423
CARD16	2.97E-52	0.368676141	0.511	0.234	5.60E-48
AC103702.2	1.43E-26	0.368140856	0.82	0.698	2.70E-22
PSMB6	3.44E-27	0.363316206	0.889	0.857	6.49E-23
RARRES3	1.51E-43	0.361256333	0.331	0.12	2.85E-39
ASRGL1	2.16E-35	0.358478631	0.652	0.44	4.08E-31
MT-ND4L	2.69E-24	0.357056837	0.883	0.853	5.08E-20
TAP1	8.80E-24	0.355664773	0.668	0.511	1.66E-19
GGH	3.24E-19	0.35109583	0.846	0.792	6.11E-15
TCIM	3.11E-17	0.349364629	0.469	0.316	5.87E-13
COX5A	6.78E-41	0.347394063	0.965	0.978	1.28E-36
UQCRC1	9.31E-31	0.344664136	0.917	0.902	1.76E-26
RPL22L1	1.43E-21	0.344419683	0.886	0.84	2.70E-17
C2	4.66E-81	0.342793428	0.386	0.099	8.79E-77
URAD	9.14E-75	0.342653797	0.375	0.1	1.73E-70
ATP5F1A	2.62E-29	0.341327973	0.94	0.917	4.94E-25
IRF1	1.50E-08	0.341067265	0.508	0.431	0.000283293
LDLRAD4	1.84E-35	0.336951844	0.558	0.317	3.48E-31
HOXB9	8.28E-29	0.335390724	0.771	0.615	1.56E-24
PSME1	2.34E-26	0.334054574	0.945	0.951	4.41E-22
DUOX2	4.72E-23	0.333699177	0.486	0.291	8.91E-19
NFKBIA	9.74E-11	0.329399737	0.789	0.749	1.84E-06

GMDS	4.68E-27	0.32939697	0.922	0.886	8.84E-23
S100A6	4.55E-27	0.327381946	0.998	0.998	8.59E-23
SERF2	2.24E-61	0.326405785	0.992	0.997	4.24E-57
C15orf48	5.43E-08	0.324789867	0.889	0.862	0.001025897
DAB2	1.25E-16	0.323028635	0.654	0.516	2.36E-12
WFDC2	2.60E-21	0.316069677	0.252	0.115	4.90E-17
GLRX	4.25E-23	0.311866398	0.718	0.58	8.03E-19
COX7B	1.38E-35	0.310942232	0.965	0.983	2.60E-31
SOD2	2.17E-15	0.310237335	0.72	0.63	4.10E-11
MRPL12	2.31E-18	0.309438824	0.866	0.821	4.36E-14
CYBA	1.55E-28	0.308348553	0.957	0.963	2.93E-24
CHCHD10	5.48E-23	0.307356405	0.943	0.953	1.04E-18
NXPE4	1.51E-58	0.306805809	0.378	0.122	2.86E-54
COX5B	1.80E-42	0.305950557	0.98	0.994	3.40E-38
ZNF814	6.62E-49	0.304683518	0.272	0.079	1.25E-44
CCND2	1.15E-23	0.302949178	0.814	0.609	2.18E-19
TAGLN2	8.97E-19	0.302304041	0.903	0.829	1.69E-14
DMAC1	7.69E-26	0.301695167	0.878	0.837	1.45E-21
EPHB3	2.38E-18	0.300629916	0.698	0.551	4.49E-14
NQO1	2.64E-14	0.295296744	0.871	0.823	4.98E-10
PARM1	1.56E-32	0.293660554	0.571	0.345	2.95E-28
PRDX5	1.57E-19	0.293139105	0.977	0.985	2.96E-15
TCEAL9	2.09E-11	0.290942332	0.574	0.474	3.94E-07
ALDH2	1.23E-16	0.289110686	0.908	0.918	2.33E-12
RAB32	6.69E-29	0.287085268	0.695	0.468	1.26E-24
NUCB2	1.10E-14	0.285471883	0.595	0.472	2.08E-10
EIF4A1	8.54E-19	0.284203464	0.872	0.832	1.61E-14
CALR	1.00E-19	0.28105909	0.945	0.948	1.89E-15
COA3	1.12E-23	0.27940489	0.917	0.899	2.11E-19
CYB5A	8.56E-17	0.279182064	0.894	0.873	1.62E-12
MT-ND1	2.50E-21	0.278732374	0.986	0.985	4.72E-17
SATB2	1.26E-22	0.278544632	0.618	0.442	2.38E-18
TUFM	2.15E-24	0.27825925	0.934	0.934	4.06E-20
YWHAE	1.45E-23	0.276362859	0.962	0.967	2.74E-19
SLC12A2	2.00E-15	0.273582624	0.968	0.964	3.78E-11
SELENOW	1.64E-15	0.270885087	0.891	0.871	3.09E-11
SYNGR2	3.46E-21	0.27069748	0.888	0.871	6.54E-17
SLPI	4.03E-06	0.270106928	0.632	0.554	0.076087476
MRPL23	1.68E-18	0.269536259	0.768	0.672	3.17E-14
CNDP2	2.31E-18	0.269492524	0.706	0.578	4.35E-14
S100A13	8.72E-19	0.269359506	0.743	0.6	1.65E-14
UBE2L6	7.91E-15	0.267580554	0.557	0.43	1.49E-10
PTMS	1.06E-07	0.265046903	0.812	0.795	0.002000995

H2AFJ	4.84E-14	0.263658063	0.918	0.923	9.14E-10
IFITM3	8.74E-25	0.262813577	0.943	0.926	1.65E-20
MRPS21	2.66E-09	0.261926372	0.551	0.47	5.02E-05
NDUFA1	7.19E-26	0.25838096	0.963	0.978	1.36E-21
CYCS	5.67E-18	0.255656437	0.94	0.952	1.07E-13
PYCARD	9.88E-16	0.25426511	0.815	0.699	1.87E-11
SLC25A5	3.65E-19	0.253455248	0.96	0.98	6.89E-15
PDHA1	5.43E-13	0.252960666	0.803	0.728	1.02E-08
ART3	2.27E-46	0.250401269	0.265	0.078	4.28E-42
EBP	0.001526784	-0.25002643	0.708	0.723	1
TMEM176A	2.51E-12	-0.251069187	0.518	0.62	4.74E-08
CFDP1	6.23E-14	-0.251652523	0.725	0.79	1.18E-09
KIF5B	9.00E-16	-0.253092616	0.898	0.963	1.70E-11
BNIP3L	2.18E-19	-0.255889496	0.446	0.608	4.12E-15
SOX4	9.01E-16	-0.256176388	0.931	0.961	1.70E-11
SLC3A2	5.77E-12	-0.256573428	0.603	0.706	1.09E-07
TSTA3	5.86E-14	-0.257173655	0.763	0.825	1.11E-09
MFSD10	3.13E-23	-0.257486438	0.665	0.777	5.90E-19
LMO7	6.88E-14	-0.257963923	0.597	0.716	1.30E-09
WEE1	9.85E-14	-0.259403096	0.326	0.467	1.86E-09
RHEB	1.79E-24	-0.261018853	0.792	0.913	3.37E-20
CTSV	2.74E-24	-0.261195394	0.086	0.273	5.17E-20
LACTB2	1.47E-16	-0.261332618	0.434	0.57	2.77E-12
F3	1.33E-10	-0.261485936	0.246	0.371	2.51E-06
SLC6A8	9.60E-10	-0.261653204	0.177	0.284	1.81E-05
CEBPD	1.69E-09	-0.261774053	0.726	0.78	3.19E-05
MPST	2.07E-20	-0.262176513	0.889	0.946	3.92E-16
PPP1R16A	3.34E-21	-0.263332649	0.586	0.735	6.30E-17
CXADR	4.01E-15	-0.264131972	0.84	0.915	7.56E-11
ENY2	7.22E-10	-0.264980862	0.882	0.903	1.36E-05
TXNIP	0.000926688	-0.266112399	0.651	0.657	1
BCL2L1	1.91E-25	-0.26645328	0.414	0.604	3.60E-21
CRNDE	4.88E-25	-0.266464538	0.317	0.543	9.22E-21
RAD21	3.68E-10	-0.268009675	0.686	0.719	6.96E-06
EPCAM	1.40E-16	-0.269050311	0.989	0.996	2.64E-12
ROMO1	3.38E-05	-0.269816348	0.911	0.932	0.638759455
RPS21	2.19E-31	-0.270545705	0.982	0.996	4.14E-27
PRSS3	3.46E-09	-0.270878181	0.834	0.86	6.54E-05
PTTG1	0.032056469	-0.272807685	0.263	0.291	1
GNAS	7.54E-11	-0.273146564	0.92	0.947	1.42E-06
PRR15L	6.02E-22	-0.274014233	0.563	0.702	1.14E-17
DHRS7	2.70E-17	-0.274065614	0.745	0.825	5.09E-13
PCLAF	0.004500594	-0.274539543	0.275	0.315	1

INSIG2	2.67E-20	-0.275607922	0.328	0.495	5.04E-16
VEGFA	2.65E-10	-0.276448633	0.411	0.518	5.01E-06
NAA20	0.07935374	-0.276544886	0.76	0.757	1
GCHFR	3.92E-15	-0.277119307	0.429	0.551	7.40E-11
ACTG1	4.40E-12	-0.277122011	0.985	0.998	8.30E-08
RPS20	6.95E-48	-0.277926561	0.989	0.996	1.31E-43
EEF2	3.07E-22	-0.278901815	0.972	0.99	5.81E-18
CDKN2A	1.33E-37	-0.280132453	0.075	0.328	2.52E-33
GID8	4.84E-22	-0.28035183	0.558	0.693	9.14E-18
SDCBP	4.46E-12	-0.280775175	0.8	0.865	8.43E-08
TSPAN8	1.47E-15	-0.281948616	0.985	0.995	2.77E-11
ADD3	2.29E-22	-0.28304508	0.763	0.858	4.33E-18
CCNI	6.88E-24	-0.283381272	0.945	0.975	1.30E-19
EIF6	1.95E-12	-0.283400263	0.878	0.899	3.67E-08
ANXA3	6.36E-12	-0.284724657	0.746	0.802	1.20E-07
RPS16	8.81E-39	-0.285458491	0.989	0.998	1.66E-34
IGHA1	6.67E-32	-0.285637052	0.355	0.628	1.26E-27
PSAT1	5.54E-19	-0.286042481	0.128	0.291	1.05E-14
PHLDA1	5.86E-16	-0.286181671	0.503	0.645	1.11E-11
PNRC2	8.01E-29	-0.286188483	0.592	0.762	1.51E-24
CLTB	3.34E-11	-0.286584239	0.858	0.897	6.31E-07
STMN1	9.06E-08	-0.287066205	0.54	0.609	0.001711026
AZIN1	5.13E-25	-0.28812969	0.509	0.671	9.69E-21
CYP2S1	2.11E-20	-0.288360929	0.198	0.371	3.98E-16
CIB1	6.57E-08	-0.288915368	0.869	0.882	0.001241064
HSP90AA1	2.30E-05	-0.289610458	0.969	0.991	0.433891039
FDPS	0.008746075	-0.293289174	0.783	0.781	1
IDI1	1.04E-05	-0.29436345	0.505	0.55	0.196047668
PMEPA1	1.44E-13	-0.295225795	0.578	0.695	2.72E-09
KLF6	9.51E-08	-0.296131252	0.792	0.863	0.0017965
KLF5	1.96E-20	-0.296977564	0.889	0.953	3.69E-16
DDAH2	1.47E-27	-0.298030855	0.692	0.849	2.77E-23
CD55	0.006356054	-0.299072956	0.5	0.531	1
SORL1	1.45E-13	-0.299098917	0.514	0.625	2.73E-09
SERPINB6	1.70E-18	-0.299743891	0.889	0.955	3.22E-14
MSMO1	2.66E-12	-0.300178321	0.352	0.477	5.02E-08
SMIM31	4.59E-22	-0.30053526	0.291	0.483	8.66E-18
SFN	0.000162319	-0.301742493	0.691	0.676	1
PTTG1IP	7.04E-17	-0.302857935	0.652	0.772	1.33E-12
PDGFA	1.50E-26	-0.303410318	0.158	0.372	2.82E-22
SLC39A4	1.29E-13	-0.304594218	0.694	0.75	2.43E-09
FTH1	4.34E-30	-0.304790756	0.997	0.998	8.19E-26
EGLN3	2.02E-18	-0.305119383	0.257	0.42	3.82E-14

PDXK	1.43E-14	-0.305625539	0.672	0.739	2.70E-10
TAF7	7.89E-21	-0.306887293	0.78	0.859	1.49E-16
RPS4Y1	9.92E-16	-0.307171965	0.842	0.839	1.87E-11
ADM	2.59E-11	-0.308375541	0.158	0.275	4.88E-07
CCNG1	3.23E-30	-0.308843479	0.729	0.84	6.10E-26
NOP53	1.40E-13	-0.310134544	0.954	0.965	2.64E-09
TRIB1	8.73E-18	-0.312284747	0.465	0.615	1.65E-13
VDAC1	6.90E-10	-0.312626529	0.946	0.974	1.30E-05
AZGP1	1.06E-16	-0.314019777	0.189	0.355	1.99E-12
CLDN15	8.81E-32	-0.316472962	0.286	0.526	1.66E-27
MYC	6.32E-10	-0.317261214	0.611	0.693	1.19E-05
SLC16A3	1.36E-08	-0.318033964	0.345	0.425	0.000257601
IGKC	1.64E-26	-0.318799947	0.465	0.717	3.10E-22
PNRC1	4.07E-17	-0.319970516	0.703	0.801	7.69E-13
ITPR2	1.15E-12	-0.322621118	0.382	0.52	2.16E-08
PSMA7	5.33E-07	-0.322734809	0.943	0.983	0.010065143
GPRC5A	2.62E-14	-0.322741527	0.709	0.779	4.95E-10
ERRFI1	4.54E-15	-0.324019148	0.242	0.399	8.58E-11
CHMP4B	1.78E-24	-0.326423758	0.877	0.938	3.35E-20
ANXA4	1.85E-18	-0.329053853	0.838	0.894	3.49E-14
SLC2A1	9.96E-19	-0.329176844	0.151	0.31	1.88E-14
TMEM123	2.30E-20	-0.335361758	0.912	0.965	4.33E-16
EIF3H	1.53E-38	-0.337887989	0.938	0.972	2.88E-34
CES2	2.55E-10	-0.340244445	0.418	0.509	4.82E-06
RBM39	6.32E-22	-0.342265698	0.9	0.951	1.19E-17
PGAM1	5.72E-07	-0.342455827	0.832	0.832	0.010792784
PRSS8	2.82E-17	-0.342823487	0.582	0.675	5.33E-13
CDH17	1.14E-24	-0.342855276	0.812	0.908	2.16E-20
DNAJB1	6.95E-19	-0.343244352	0.666	0.776	1.31E-14
ZFP36L2	9.36E-14	-0.343420977	0.935	0.946	1.77E-09
ANXA13	1.92E-34	-0.34724114	0.112	0.365	3.63E-30
S100A11	0.000131359	-0.349407313	0.975	0.991	1
JUN	1.66E-19	-0.349739955	0.952	0.976	3.13E-15
PRSS22	1.86E-34	-0.34996156	0.115	0.352	3.52E-30
P4HA1	8.72E-25	-0.350194134	0.215	0.407	1.65E-20
CYSTM1	1.39E-17	-0.350734373	0.9	0.953	2.63E-13
APLP2	8.46E-16	-0.351184764	0.748	0.793	1.60E-11
COTL1	1.06E-18	-0.354543303	0.491	0.611	2.00E-14
ATF4	1.61E-22	-0.357051882	0.766	0.855	3.03E-18
NEU1	1.35E-31	-0.357251235	0.434	0.639	2.56E-27
ZFP36L1	1.97E-21	-0.357302896	0.877	0.934	3.72E-17
AGR2	4.82E-06	-0.358386048	0.969	0.985	0.090959556
H2AFZ	0.119302444	-0.358611267	0.925	0.938	1

S100A10	2.17E-05	-0.359454747	0.985	0.997	0.408838069
PLBD1	3.61E-28	-0.361533588	0.712	0.833	6.81E-24
VDAC2	9.31E-22	-0.362357321	0.908	0.959	1.76E-17
RPL30	3.36E-85	-0.366724511	0.992	0.998	6.34E-81
TSPAN3	2.92E-29	-0.369960509	0.731	0.844	5.51E-25
YWHAB	3.00E-14	-0.371970046	0.923	0.963	5.66E-10
CD164	1.36E-32	-0.374327706	0.895	0.961	2.57E-28
C4orf48	1.35E-13	-0.378711721	0.842	0.832	2.56E-09
RAB5IF	2.98E-10	-0.379615612	0.818	0.836	5.62E-06
CNIH4	1.07E-22	-0.379770328	0.672	0.742	2.02E-18
EDN1	9.30E-16	-0.38440881	0.255	0.417	1.76E-11
TMEM45B	4.03E-23	-0.387199309	0.638	0.746	7.60E-19
PLAUR	2.27E-09	-0.387356212	0.249	0.36	4.28E-05
SMIM24	2.91E-24	-0.38838118	0.115	0.308	5.50E-20
EZR	1.84E-18	-0.388635509	0.698	0.775	3.47E-14
EEF1D	2.15E-35	-0.390947074	0.962	0.994	4.05E-31
SPINK1	0.451944238	-0.399044082	0.957	0.962	1
PERP	3.18E-41	-0.400210159	0.912	0.982	6.01E-37
CST3	1.85E-05	-0.400491529	0.974	0.98	0.348591257
CYP2W1	1.74E-30	-0.406076989	0.078	0.299	3.28E-26
EIF1	1.03E-33	-0.409726105	0.98	0.997	1.95E-29
SERPINA1	0.472554134	-0.413383649	0.371	0.342	1
RPL8	1.94E-82	-0.415317825	0.997	0.999	3.66E-78
EIF2S2	1.58E-14	-0.416925886	0.877	0.903	2.99E-10
CEACAM6	0.032356852	-0.421045759	0.303	0.324	1
CCND1	5.04E-10	-0.421209013	0.715	0.744	9.52E-06
GLO1	9.98E-24	-0.421420877	0.725	0.819	1.88E-19
ISG15	2.04E-07	-0.421722772	0.423	0.497	0.00384449
CEBPB	6.40E-20	-0.422544871	0.663	0.785	1.21E-15
FTL	2.68E-50	-0.431231296	0.986	0.998	5.05E-46
TRAM1	1.62E-26	-0.440805549	0.666	0.752	3.06E-22
SLC11A2	1.21E-18	-0.442909397	0.355	0.513	2.28E-14
FBXO2	2.23E-40	-0.444414342	0.048	0.302	4.22E-36
PPDPF	9.86E-55	-0.447812059	0.978	0.995	1.86E-50
GPX4	4.91E-48	-0.457078115	0.897	0.972	9.26E-44
C12orf75	2.41E-17	-0.459693247	0.692	0.749	4.56E-13
HSD17B11	6.96E-47	-0.461768202	0.798	0.906	1.31E-42
DDIT4	7.80E-07	-0.461844554	0.508	0.579	0.014736585
YWHAZ	6.89E-30	-0.466606943	0.928	0.974	1.30E-25
CLDN4	3.76E-33	-0.471204297	0.946	0.976	7.10E-29
TPD52	3.41E-40	-0.476360617	0.875	0.943	6.44E-36
PHLDA2	9.10E-14	-0.481637377	0.582	0.668	1.72E-09
NUPR1	0.185158946	-0.49214967	0.626	0.592	1

C4orf3	2.96E-19	-0.497319185	0.872	0.931	5.60E-15
LGALS2	2.88E-20	-0.505988259	0.154	0.327	5.44E-16
AC020656.1	1.55E-09	-0.516416731	0.229	0.337	2.92E-05
ZNF706	4.08E-48	-0.521279707	0.868	0.937	7.71E-44
C6orf48	1.47E-43	-0.523466175	0.8	0.906	2.78E-39
MISP	8.91E-26	-0.530090522	0.752	0.836	1.68E-21
PABPC1	3.16E-84	-0.530312418	0.977	0.998	5.97E-80
FAM13A	1.09E-18	-0.532578847	0.289	0.441	2.06E-14
FCGRT	2.65E-53	-0.532707672	0.735	0.885	5.00E-49
EIF4A2	8.95E-43	-0.534122642	0.923	0.972	1.69E-38
FXYD3	0.065950316	-0.535021714	0.966	0.978	1
MAL2	6.22E-33	-0.53683656	0.757	0.853	1.18E-28
FXYD5	6.45E-33	-0.542689718	0.392	0.578	1.22E-28
PRAP1	1.82E-23	-0.546036273	0.354	0.534	3.43E-19
EIF3E	5.25E-68	-0.551995563	0.934	0.979	9.92E-64
FHL2	2.00E-15	-0.552327	0.803	0.844	3.78E-11
LGALS3	8.79E-19	-0.576771323	0.971	0.994	1.66E-14
LDHA	2.70E-06	-0.592083839	0.962	0.973	0.050957846
GDF15	7.93E-14	-0.595512534	0.623	0.738	1.50E-09
CFD	1.14E-28	-0.597047939	0.238	0.446	2.15E-24
HSPA1B	1.95E-39	-0.604671517	0.415	0.647	3.68E-35
SQLE	4.39E-17	-0.616052628	0.414	0.541	8.29E-13
CAMK2N1	2.64E-64	-0.631592222	0.672	0.89	4.99E-60
KRT20	1.81E-25	-0.649806421	0.378	0.571	3.43E-21
PGK1	1.45E-13	-0.652395335	0.871	0.9	2.73E-09
SCD	8.20E-18	-0.682252535	0.455	0.573	1.55E-13
ZFAS1	2.62E-67	-0.720532883	0.937	0.988	4.94E-63
C8orf33	7.09E-32	-0.732884346	0.485	0.655	1.34E-27
HSPB1	4.02E-38	-0.746382798	0.638	0.837	7.59E-34
ERO1A	4.14E-27	-0.752051071	0.537	0.668	7.82E-23
BST2	1.45E-24	-0.757967302	0.169	0.352	2.73E-20
REG1A	0.080365892	-0.762239831	0.315	0.325	1
MALL	7.22E-46	-0.76443032	0.122	0.4	1.36E-41
KRT19	3.00E-42	-0.814130416	0.909	0.976	5.67E-38
TM4SF1	1.29E-46	-0.903548075	0.494	0.732	2.44E-42
CA9	4.39E-39	-0.907234665	0.178	0.429	8.28E-35
PLAC8	6.32E-10	-0.939208254	0.348	0.433	1.19E-05
TNFRSF12A	1.12E-44	-0.94194626	0.409	0.627	2.11E-40
TFF3	6.67E-45	-0.95392731	0.972	0.995	1.26E-40
LYZ	6.23E-11	-0.972987477	0.789	0.816	1.18E-06
CSTB	7.51E-41	-1.062274581	0.958	0.981	1.42E-36
CKB	0.107141764	-1.072867386	0.875	0.881	1
ANPEP	1.19E-36	-1.07932523	0.058	0.295	2.24E-32

NDRG1	2.38E-49	-1.105392311	0.312	0.582	4.49E-45
AREG	6.18E-35	-1.186978125	0.326	0.542	1.17E-30
TFF1	1.93E-16	-1.332117605	0.298	0.447	3.64E-12
MMP7	5.36E-39	-1.776447313	0.346	0.572	1.01E-34
FABP1	2.21E-33	-1.873285922	0.306	0.524	4.17E-29
**Cluster 4**					
**Gene symbol**	**p_val**	**avg_log2FC**	**pct.1**	**pct.2**	**p_val_adj**
FABP5	3.90E-66	1.02342557	0.842	0.611	7.37E-62
S100P	4.27E-12	0.956663592	0.627	0.609	8.07E-08
PLA2G2A	2.65E-27	0.892654926	0.653	0.428	5.01E-23
TUBA1B	1.53E-30	0.834321914	0.918	0.889	2.89E-26
S100A6	1.55E-25	0.774445913	0.991	0.999	2.93E-21
H2AFZ	1.03E-34	0.769622821	0.951	0.934	1.95E-30
REG4	1.18E-19	0.731929717	0.527	0.32	2.23E-15
LDHB	1.11E-41	0.730284983	0.771	0.48	2.10E-37
ASS1	7.64E-19	0.728256189	0.722	0.602	1.44E-14
CD74	3.89E-09	0.718700841	0.698	0.64	7.35E-05
LCN2	0.008372444	0.707450078	0.778	0.822	1
PSME2	3.83E-66	0.702963316	0.956	0.906	7.22E-62
CXCL1	1.04E-05	0.668785918	0.516	0.443	0.196160713
PTTG1	8.06E-25	0.647922257	0.478	0.26	1.52E-20
TUBB4B	1.35E-35	0.629644802	0.913	0.851	2.56E-31
PCLAF	3.97E-33	0.61782497	0.544	0.275	7.50E-29
TUBB	1.49E-26	0.604926291	0.911	0.846	2.80E-22
SFN	2.85E-42	0.599845319	0.86	0.653	5.38E-38
ENO1	1.21E-40	0.59878535	0.967	0.963	2.29E-36
RPL22L1	2.06E-15	0.593379173	0.873	0.845	3.90E-11
HSPE1	3.90E-45	0.585713626	0.976	0.977	7.36E-41
RANBP1	8.39E-31	0.58512934	0.929	0.893	1.58E-26
MT2A	2.82E-10	0.583149909	0.549	0.402	5.32E-06
PA2G4	3.11E-52	0.582594873	0.938	0.908	5.88E-48
RAN	2.47E-50	0.579735344	0.964	0.969	4.66E-46
PI3	5.66E-19	0.57792156	0.271	0.118	1.07E-14
EIF5A	1.05E-26	0.557949803	0.938	0.916	1.98E-22
TAGLN2	9.77E-42	0.556647748	0.916	0.832	1.84E-37
PFN1	2.07E-42	0.555496027	0.967	0.989	3.91E-38
NME1	2.92E-41	0.548395277	0.911	0.809	5.52E-37
PKM	1.76E-40	0.542176157	0.969	0.955	3.32E-36
SLIRP	3.33E-49	0.539536389	0.944	0.942	6.29E-45
KRT18	3.79E-31	0.531652381	0.991	0.995	7.15E-27
HIST1H4C	0.004180547	0.528910238	0.807	0.813	1
CXCL8	2.65E-11	0.524690102	0.253	0.138	5.00E-07
HSPD1	1.32E-25	0.519052799	0.969	0.962	2.49E-21

ATP5ME	5.62E-43	0.518567049	0.973	0.983	1.06E-38
SNRPG	3.91E-44	0.5144681	0.94	0.944	7.38E-40
KRT8	2.79E-31	0.511182649	0.993	0.997	5.26E-27
SNRPD1	2.48E-36	0.507699265	0.924	0.892	4.68E-32
HSP90AA1	1.13E-28	0.503380308	0.976	0.989	2.14E-24
STMN1	2.86E-17	0.499442407	0.753	0.575	5.40E-13
SOD3	1.66E-12	0.496001323	0.429	0.277	3.13E-08
CXCL3	2.07E-05	0.494015536	0.656	0.592	0.39139668
ATP5IF1	6.51E-44	0.491737678	0.96	0.944	1.23E-39
SNRPF	5.33E-47	0.49069034	0.942	0.92	1.01E-42
NCL	1.68E-23	0.481616701	0.931	0.941	3.18E-19
SPINK4	5.43E-13	0.477191996	0.369	0.22	1.03E-08
HMGN2	7.44E-10	0.470585436	0.944	0.953	1.41E-05
PTMA	1.01E-52	0.46905499	0.993	0.999	1.91E-48
SRSF7	4.87E-30	0.464686077	0.9	0.835	9.19E-26
CENPW	2.01E-27	0.464624796	0.647	0.417	3.80E-23
SDF2L1	1.35E-32	0.461992263	0.858	0.713	2.54E-28
S100A11	4.25E-40	0.461437461	0.987	0.989	8.03E-36
LRRC59	3.70E-36	0.460251405	0.824	0.624	6.98E-32
ATP5MD	8.59E-48	0.458276543	0.973	0.984	1.62E-43
TUBA1C	4.77E-25	0.45821138	0.884	0.748	9.01E-21
DBI	7.51E-31	0.457819469	0.953	0.96	1.42E-26
PSMB9	4.85E-15	0.456850798	0.68	0.547	9.15E-11
PIGR	1.08E-12	0.455017103	0.442	0.284	2.03E-08
ANPEP	4.61E-11	0.453103216	0.38	0.236	8.70E-07
C15orf48	2.77E-15	0.4499112	0.902	0.862	5.22E-11
KPNA2	8.85E-30	0.448567484	0.573	0.31	1.67E-25
MT1E	2.08E-16	0.445841043	0.433	0.248	3.93E-12
S100A16	1.94E-16	0.444836169	0.833	0.786	3.67E-12
DUOXA2	4.32E-12	0.443911379	0.304	0.18	8.16E-08
HMGB2	0.000923485	0.442606676	0.627	0.588	1
DUOX2	6.01E-08	0.441829859	0.409	0.314	0.001134188
CCT5	1.99E-34	0.440681027	0.918	0.843	3.76E-30
HMGA1	7.75E-26	0.439893783	0.922	0.899	1.46E-21
CACYBP	8.74E-23	0.439453774	0.853	0.758	1.65E-18
ATP5MC3	4.56E-40	0.438073742	0.967	0.983	8.60E-36
JPT1	1.14E-33	0.437768661	0.922	0.807	2.15E-29
PSMA3	5.30E-33	0.434275077	0.9	0.838	1.00E-28
CKS2	5.01E-13	0.43412136	0.773	0.675	9.46E-09
CYCS	2.76E-26	0.433615938	0.949	0.95	5.21E-22
CALR	5.99E-28	0.433416197	0.942	0.948	1.13E-23
PGAM1	3.38E-31	0.432905525	0.909	0.822	6.38E-27
PPIB	1.96E-33	0.425624247	0.953	0.962	3.70E-29

ANXA2	5.43E-26	0.425125824	0.987	0.982	1.03E-21
TIMM8B	9.77E-36	0.421313417	0.902	0.816	1.85E-31
HNRNPA2B1	5.37E-27	0.421181299	0.958	0.976	1.01E-22
MRPS21	1.11E-32	0.420467109	0.713	0.452	2.10E-28
HNRNPA3	2.71E-28	0.416405506	0.929	0.925	5.11E-24
S100A2	1.45E-16	0.415933637	0.278	0.134	2.74E-12
TXN	2.81E-41	0.415478342	0.987	0.993	5.30E-37
CCNB1	2.94E-21	0.412842864	0.358	0.174	5.55E-17
PRDX1	1.36E-29	0.412366228	0.958	0.976	2.57E-25
PSMA7	3.60E-34	0.411199896	0.967	0.977	6.81E-30
SRSF2	2.02E-27	0.406281424	0.904	0.866	3.82E-23
CASP1	1.90E-17	0.401818077	0.618	0.457	3.59E-13
NPM1	3.52E-23	0.401686989	0.978	0.988	6.64E-19
ANXA1	1.39E-23	0.400842471	0.342	0.15	2.63E-19
GSTP1	7.07E-39	0.400433919	0.982	0.993	1.34E-34
BUB3	3.60E-08	0.400316548	0.767	0.663	0.000680461
AP2S1	6.09E-29	0.398891251	0.916	0.879	1.15E-24
ATP5PF	7.51E-46	0.395955071	0.962	0.974	1.42E-41
SRSF3	2.78E-26	0.39557884	0.942	0.941	5.24E-22
MUC2	2.67E-19	0.394478009	0.304	0.14	5.04E-15
NDUFS6	1.54E-32	0.393035177	0.931	0.942	2.91E-28
TOMM40	6.15E-29	0.392273532	0.796	0.636	1.16E-24
PRDX2	4.63E-21	0.392040325	0.949	0.961	8.73E-17
TYMS	3.77E-29	0.392019924	0.516	0.265	7.12E-25
PSMB3	1.07E-30	0.389667752	0.927	0.902	2.02E-26
WARS	9.03E-15	0.388917401	0.449	0.271	1.71E-10
PSMB2	1.65E-28	0.388361793	0.907	0.861	3.11E-24
LSM4	1.79E-24	0.388133691	0.918	0.879	3.38E-20
BIRC5	3.87E-32	0.386984259	0.402	0.168	7.31E-28
PCNA	3.81E-16	0.386271649	0.642	0.485	7.18E-12
RRM2	1.92E-32	0.385881361	0.384	0.149	3.62E-28
TPM3	1.56E-24	0.385425894	0.916	0.87	2.95E-20
IER3	2.00E-08	0.385253886	0.749	0.649	0.000378514
COX7B	4.71E-37	0.38516165	0.964	0.982	8.89E-33
HMGB1	1.52E-16	0.384142247	0.973	0.988	2.87E-12
OLFM4	0.001095144	0.378363136	0.902	0.899	1
ACTB	3.63E-31	0.377617499	0.996	0.995	6.85E-27
CEACAM6	8.42E-33	0.37760566	0.576	0.284	1.59E-28
RAB5IF	9.30E-27	0.377310926	0.913	0.821	1.76E-22
HNRNPAB	3.52E-21	0.377019421	0.911	0.883	6.65E-17
CENPX	6.28E-20	0.373615049	0.842	0.742	1.19E-15
ERH	4.84E-29	0.373134118	0.938	0.945	9.13E-25
ATP5MPL	9.38E-38	0.370141013	0.967	0.981	1.77E-33

SLC25A5	2.43E-26	0.369453105	0.962	0.979	4.59E-22
MRPL52	3.84E-30	0.368806582	0.913	0.852	7.24E-26
UQCRQ	5.63E-35	0.368217532	0.976	0.988	1.06E-30
MAD2L1	3.73E-31	0.367738168	0.469	0.22	7.05E-27
NDUFAB1	5.80E-26	0.36643766	0.916	0.937	1.10E-21
FABP1	2.16E-24	0.366160277	0.722	0.452	4.07E-20
CENPF	2.98E-20	0.365549907	0.336	0.159	5.63E-16
PSMA4	2.85E-25	0.364640552	0.938	0.932	5.39E-21
UBE2C	1.38E-17	0.363917041	0.302	0.142	2.61E-13
EIF6	1.62E-21	0.360302603	0.92	0.892	3.06E-17
YBX1	4.35E-28	0.35931791	0.989	0.993	8.21E-24
HNRNPM	9.36E-25	0.359279494	0.893	0.827	1.77E-20
CYC1	4.99E-19	0.356801298	0.931	0.956	9.43E-15
ODC1	1.61E-21	0.356541248	0.842	0.731	3.04E-17
TMA7	9.87E-34	0.355719806	0.973	0.991	1.86E-29
ATP5MC1	2.35E-25	0.355446355	0.936	0.911	4.44E-21
IDH2	4.15E-20	0.355166092	0.929	0.86	7.84E-16
HSP90AB1	1.02E-17	0.35299251	0.967	0.986	1.93E-13
ASRGL1	1.69E-27	0.351749292	0.687	0.448	3.19E-23
WDR34	6.29E-22	0.351473249	0.702	0.485	1.19E-17
NASP	5.77E-18	0.351035267	0.756	0.613	1.09E-13
CCT2	1.60E-23	0.35084231	0.891	0.84	3.02E-19
TCP1	3.87E-17	0.349829542	0.876	0.788	7.31E-13
POLR2L	2.00E-29	0.349216596	0.944	0.946	3.77E-25
TMSB4X	9.78E-20	0.349194387	1	0.998	1.85E-15
HMGB3	9.62E-18	0.349036718	0.756	0.574	1.82E-13
CFD	9.66E-06	0.348526708	0.533	0.392	0.182470555
LSM3	1.24E-18	0.348344064	0.922	0.875	2.34E-14
UBE2L3	5.95E-28	0.34774271	0.9	0.833	1.12E-23
CALM1	4.38E-27	0.346862686	0.962	0.975	8.27E-23
BAX	1.46E-20	0.346251257	0.891	0.82	2.76E-16
POMP	3.59E-28	0.342515425	0.958	0.978	6.77E-24
NOP16	7.37E-25	0.341960753	0.736	0.536	1.39E-20
SNRPB	1.84E-20	0.341356456	0.931	0.928	3.47E-16
APOL1	4.90E-18	0.340573337	0.258	0.117	9.26E-14
HMGCS2	6.43E-20	0.33874543	0.642	0.415	1.21E-15
ETHE1	1.30E-19	0.338669	0.873	0.85	2.45E-15
EIF4A1	1.36E-18	0.338077205	0.873	0.834	2.57E-14
S100A10	3.16E-30	0.337714705	0.991	0.995	5.97E-26
TK1	2.06E-33	0.337180933	0.473	0.215	3.89E-29
IL32	7.84E-07	0.336237125	0.907	0.852	0.01480034
TIMM13	5.88E-27	0.335314085	0.949	0.937	1.11E-22
ARPC5L	2.17E-22	0.332752187	0.844	0.693	4.09E-18

EBNA1BP2	3.70E-22	0.332336973	0.809	0.692	6.99E-18
SOD1	3.45E-26	0.332242214	0.973	0.982	6.51E-22
CPS1	6.64E-07	0.331978716	0.298	0.209	0.01254391
BOLA3	3.17E-22	0.33182274	0.809	0.7	5.99E-18
COPE	1.35E-26	0.331316523	0.936	0.897	2.55E-22
EMP2	6.10E-21	0.330666762	0.802	0.664	1.15E-16
RNASE1	1.69E-23	0.330503367	0.433	0.222	3.18E-19
DTYMK	3.55E-16	0.33004254	0.618	0.462	6.70E-12
TPI1	2.54E-29	0.329479985	0.978	0.988	4.80E-25
PSMC3	7.54E-20	0.329286807	0.844	0.735	1.42E-15
ECHS1	1.25E-20	0.325509463	0.92	0.908	2.36E-16
MKI67	4.94E-22	0.325440934	0.367	0.171	9.33E-18
TIMM10	5.45E-25	0.324055001	0.771	0.589	1.03E-20
ACOT7	6.15E-32	0.323382341	0.66	0.391	1.16E-27
LMNB1	2.37E-20	0.322175166	0.518	0.315	4.48E-16
MRPL13	3.80E-17	0.318965781	0.882	0.806	7.17E-13
MUC1	7.45E-05	0.318575102	0.52	0.484	1
NOP56	1.61E-18	0.318178638	0.78	0.645	3.03E-14
ENY2	2.18E-13	0.317912599	0.933	0.894	4.11E-09
LGALS1	5.86E-25	0.317739211	0.287	0.112	1.11E-20
CFL1	1.61E-29	0.317519688	0.984	0.995	3.03E-25
MDH1	7.81E-14	0.317274237	0.869	0.826	1.48E-09
CDKN3	5.55E-27	0.316195207	0.404	0.187	1.05E-22
HSP90B1	2.06E-18	0.31579852	0.947	0.955	3.88E-14
PSMA5	1.85E-21	0.315782701	0.878	0.846	3.50E-17
TMED10	2.62E-24	0.314178486	0.918	0.857	4.94E-20
PRELID1	7.30E-17	0.313753944	0.94	0.954	1.38E-12
MCM3	1.09E-24	0.313311659	0.6	0.365	2.06E-20
NUDC	7.61E-19	0.312568433	0.889	0.836	1.44E-14
ATP6V0B	1.76E-14	0.312086636	0.893	0.856	3.33E-10
VDAC1	2.18E-13	0.311722141	0.964	0.97	4.11E-09
MRPS34	3.62E-21	0.311292548	0.887	0.858	6.83E-17
VCP	2.37E-15	0.310604839	0.833	0.708	4.48E-11
ROMO1	3.85E-19	0.310478391	0.956	0.924	7.27E-15
MRPL47	2.56E-24	0.309662157	0.813	0.654	4.84E-20
TXNDC17	1.47E-25	0.309464527	0.918	0.894	2.78E-21
PARK7	1.61E-22	0.309160942	0.938	0.951	3.03E-18
ELOC	2.15E-18	0.309027283	0.929	0.863	4.06E-14
TOMM5	7.23E-22	0.308511634	0.722	0.52	1.36E-17
UBE2N	4.61E-24	0.306489465	0.871	0.761	8.70E-20
MRPS15	3.74E-20	0.305896564	0.838	0.767	7.06E-16
MANF	2.91E-19	0.304782522	0.847	0.705	5.50E-15
GLRX3	2.09E-16	0.304260215	0.827	0.742	3.95E-12

SEC61B	3.57E-24	0.303828411	0.953	0.956	6.74E-20
TIMM17A	3.30E-25	0.303619748	0.791	0.633	6.22E-21
PDIA3	2.94E-17	0.303273907	0.942	0.947	5.56E-13
SSB	2.18E-18	0.303244765	0.898	0.866	4.12E-14
UBL5	4.31E-29	0.30311397	0.96	0.976	8.14E-25
MRPL12	5.43E-16	0.302499776	0.873	0.823	1.02E-11
GADD45GIP1	3.37E-26	0.302025002	0.924	0.889	6.37E-22
DDX39A	5.46E-21	0.301615278	0.662	0.475	1.03E-16
TSTA3	2.70E-13	0.300756373	0.876	0.805	5.10E-09
UBE2T	7.54E-22	0.299434717	0.487	0.276	1.42E-17
GSTO1	3.14E-20	0.299275302	0.909	0.868	5.92E-16
CDC37	8.30E-21	0.299146762	0.889	0.831	1.57E-16
MT-ND6	1.34E-07	0.299039809	0.771	0.711	0.002530391
CDK1	4.68E-21	0.298986875	0.264	0.104	8.84E-17
NEDD8	1.21E-24	0.297901281	0.969	0.944	2.29E-20
ZWINT	6.73E-22	0.297663544	0.487	0.267	1.27E-17
MRPL41	3.81E-21	0.297477017	0.902	0.867	7.20E-17
SH3BGRL3	3.64E-12	0.297458258	0.927	0.911	6.88E-08
PHLDA2	4.74E-21	0.295858742	0.84	0.626	8.95E-17
FDPS	1.05E-15	0.295027052	0.871	0.769	1.98E-11
CALM3	1.67E-14	0.294968669	0.873	0.81	3.16E-10
TALDO1	4.95E-18	0.294925154	0.918	0.908	9.35E-14
GINS2	7.61E-26	0.294189377	0.464	0.233	1.44E-21
SYNCRIP	1.09E-15	0.293700454	0.864	0.791	2.06E-11
POLR2F	9.35E-20	0.293385594	0.909	0.85	1.77E-15
MYDGF	2.86E-15	0.29133896	0.913	0.897	5.40E-11
AURKAIP1	4.12E-22	0.291228867	0.936	0.936	7.78E-18
TMSB10	3.10E-28	0.28929821	0.996	0.999	5.84E-24
PGP	1.31E-28	0.288905001	0.664	0.412	2.47E-24
AKR1B10	6.61E-20	0.28881637	0.427	0.23	1.25E-15
GMDS	1.54E-17	0.288429829	0.931	0.887	2.91E-13
NDUFV2	1.31E-23	0.288246952	0.927	0.923	2.48E-19
ATP5F1E	2.11E-15	0.287710175	0.98	0.993	3.99E-11
YWHAB	7.14E-19	0.287671564	0.96	0.956	1.35E-14
PDCD5	3.37E-20	0.285421245	0.913	0.869	6.36E-16
CCT7	1.73E-15	0.285346955	0.882	0.838	3.26E-11
NDUFB6	2.82E-14	0.284013331	0.882	0.821	5.32E-10
NDUFB8	2.53E-21	0.283476414	0.929	0.914	4.77E-17
C4orf48	6.15E-16	0.28227472	0.924	0.821	1.16E-11
HNRNPF	6.22E-16	0.28189451	0.92	0.921	1.17E-11
MTCH2	8.50E-20	0.281675878	0.896	0.845	1.60E-15
SRM	1.98E-18	0.280956041	0.74	0.595	3.73E-14
NDUFA6	3.10E-19	0.280923992	0.909	0.912	5.86E-15

ATP5F1B	5.90E-16	0.280289704	0.956	0.975	1.11E-11
NDUFB1	2.04E-21	0.280115292	0.94	0.948	3.86E-17
GGCT	3.82E-14	0.279585163	0.876	0.826	7.21E-10
FCGBP	3.28E-15	0.278805499	0.46	0.278	6.19E-11
COX6C	5.97E-14	0.27868812	0.976	0.993	1.13E-09
MGST2	1.85E-16	0.277984813	0.918	0.902	3.49E-12
CCNB2	1.12E-20	0.277075891	0.309	0.14	2.12E-16
GTF2A2	1.10E-14	0.27638061	0.869	0.786	2.08E-10
NDUFC2	4.17E-15	0.276158737	0.951	0.952	7.87E-11
DDX21	3.58E-14	0.275581384	0.867	0.803	6.77E-10
C1QBP	2.60E-14	0.275056319	0.907	0.906	4.92E-10
ATP5MF	1.09E-20	0.274901611	0.964	0.977	2.05E-16
SMC4	9.19E-11	0.274625829	0.58	0.423	1.74E-06
SNRPA1	5.93E-10	0.274598341	0.751	0.616	1.12E-05
MRPS12	7.40E-19	0.274281272	0.849	0.773	1.40E-14
LGALS4	3.60E-08	0.274100687	0.978	0.991	0.000680452
COPS9	5.59E-17	0.27341387	0.902	0.881	1.06E-12
MRPL36	2.39E-18	0.27337052	0.831	0.724	4.52E-14
EIF5B	8.65E-16	0.272968106	0.882	0.852	1.63E-11
POLR2K	1.45E-12	0.27257137	0.896	0.821	2.74E-08
EXOSC4	1.54E-16	0.272496376	0.738	0.558	2.91E-12
DHFR	1.44E-22	0.271894771	0.522	0.293	2.72E-18
NOP58	5.87E-15	0.271678057	0.78	0.644	1.11E-10
MCM7	2.04E-11	0.271231504	0.613	0.473	3.85E-07
UQCRC1	2.87E-16	0.271160878	0.913	0.904	5.42E-12
SLBP	4.12E-08	0.271067648	0.66	0.577	0.000777295
EIF4G2	1.38E-12	0.270719371	0.962	0.943	2.61E-08
PSMB5	3.58E-18	0.270700893	0.907	0.904	6.77E-14
SMS	7.31E-14	0.270381886	0.884	0.797	1.38E-09
GLRX	4.67E-06	0.269839038	0.676	0.594	0.088194395
GTF3C6	6.84E-15	0.269620323	0.898	0.792	1.29E-10
UQCRFS1	7.38E-18	0.269312605	0.9	0.933	1.39E-13
MRPL20	9.48E-18	0.269310748	0.911	0.888	1.79E-13
DUT	0.000977447	0.268941934	0.813	0.785	1
CENPM	1.54E-29	0.268529058	0.438	0.203	2.90E-25
PRMT1	6.57E-17	0.267562636	0.836	0.777	1.24E-12
SNRPE	2.21E-17	0.267561608	0.927	0.954	4.18E-13
NOP10	2.54E-15	0.266985165	0.918	0.91	4.80E-11
LYAR	3.77E-17	0.266506252	0.604	0.407	7.11E-13
PSME1	3.67E-17	0.265623316	0.942	0.951	6.92E-13
PSMB8	7.15E-09	0.265602203	0.842	0.851	0.000135062
NDUFA13	3.26E-22	0.26520221	0.967	0.967	6.16E-18
HSBP1	2.73E-18	0.264986922	0.933	0.926	5.15E-14

NDUFS5	1.08E-17	0.264503005	0.969	0.978	2.04E-13
IFITM3	5.34E-10	0.264273866	0.933	0.928	1.01E-05
OAZ1	7.31E-22	0.263859296	0.98	0.989	1.38E-17
PTGES3	1.97E-10	0.263736012	0.949	0.964	3.73E-06
CSE1L	3.56E-16	0.263226553	0.6	0.398	6.71E-12
TPM4	2.69E-11	0.262219504	0.882	0.85	5.09E-07
ANXA4	2.75E-09	0.26173603	0.913	0.88	5.20E-05
SCAND1	1.29E-13	0.261272597	0.929	0.876	2.44E-09
SMIM24	9.82E-07	0.261247376	0.364	0.261	0.018545076
SLC25A39	2.57E-14	0.261029194	0.871	0.81	4.85E-10
AHSA1	2.10E-12	0.261023436	0.784	0.701	3.97E-08
MRPL51	3.37E-15	0.260917969	0.918	0.907	6.36E-11
GHITM	9.74E-18	0.260827736	0.904	0.905	1.84E-13
MINOS1	7.18E-19	0.260288608	0.949	0.976	1.36E-14
HSPA4	1.39E-17	0.26020357	0.713	0.514	2.62E-13
CST3	2.63E-06	0.259878309	0.971	0.98	0.049571912
CYP2W1	0.169908926	0.25954091	0.276	0.258	1
NHP2	6.57E-15	0.259320409	0.929	0.905	1.24E-10
SOD2	1.01E-06	0.259252304	0.711	0.637	0.019138405
HSPA9	5.86E-09	0.259243211	0.873	0.838	0.000110557
TRAP1	2.60E-14	0.259229659	0.778	0.668	4.92E-10
RUVBL1	4.27E-19	0.259210101	0.691	0.484	8.06E-15
VPS29	2.03E-14	0.259131488	0.884	0.836	3.84E-10
DNMT1	1.64E-19	0.258979826	0.591	0.376	3.09E-15
CAPG	3.50E-17	0.258826713	0.853	0.691	6.61E-13
UQCR10	1.33E-18	0.258154437	0.958	0.972	2.51E-14
RNF213	0.036624747	0.257965086	0.631	0.589	1
LMNA	7.20E-06	0.257820921	0.853	0.894	0.135949642
COX17	5.54E-15	0.257784123	0.862	0.754	1.05E-10
EIF3I	4.89E-17	0.25743016	0.913	0.926	9.23E-13
MIF	1.74E-21	0.256975909	0.971	0.976	3.29E-17
LGALS3	1.23E-21	0.256880746	0.984	0.99	2.32E-17
GCHFR	3.11E-09	0.256773277	0.664	0.511	5.86E-05
RPL36AL	5.85E-22	0.256110351	0.962	0.985	1.11E-17
GUK1	7.23E-20	0.255757241	0.947	0.939	1.37E-15
TUBA4A	3.16E-16	0.255720744	0.636	0.439	5.97E-12
PHB	9.86E-15	0.255207401	0.924	0.928	1.86E-10
CCT4	5.08E-12	0.254888469	0.871	0.838	9.60E-08
ARHGDIA	2.41E-12	0.254528766	0.809	0.715	4.56E-08
ISOC2	1.45E-16	0.254369108	0.82	0.7	2.73E-12
CRIP1	2.88E-14	0.254223164	0.433	0.263	5.44E-10
SRSF1	2.23E-15	0.254168195	0.811	0.674	4.21E-11
NDUFV1	1.20E-14	0.253443172	0.878	0.813	2.27E-10

KRT19	1.59E-15	0.253318048	0.962	0.964	3.00E-11
EBP	4.86E-14	0.253255102	0.818	0.706	9.18E-10
AGR2	6.39E-08	0.253055972	0.982	0.982	0.001207015
UBE2S	4.84E-17	0.252764179	0.578	0.375	9.14E-13
RER1	1.44E-13	0.252337325	0.871	0.822	2.72E-09
ANP32A	4.24E-12	0.252051561	0.88	0.773	8.00E-08
CCDC124	7.43E-15	0.25160309	0.769	0.626	1.40E-10
GRPEL1	2.57E-15	0.250694714	0.749	0.574	4.86E-11
EIF5AL1	6.16E-14	0.250591694	0.564	0.383	1.16E-09
TUFM	1.90E-17	0.250495439	0.936	0.934	3.58E-13
NDFIP2	1.68E-13	-0.250140762	0.74	0.809	3.18E-09
DNAJC4	6.32E-16	-0.250190573	0.409	0.549	1.19E-11
ZBTB38	8.20E-08	-0.250722793	0.636	0.683	0.001548371
VAMP2	7.62E-15	-0.251669091	0.48	0.606	1.44E-10
GATAD1	1.13E-12	-0.251694297	0.44	0.529	2.14E-08
TFF3	0.000266149	-0.252148349	0.987	0.992	1
TSPAN12	1.50E-13	-0.253504707	0.34	0.47	2.83E-09
PDLIM1	2.64E-14	-0.254940421	0.862	0.916	4.98E-10
MT-ND3	6.39E-07	-0.255592712	0.96	0.992	0.012062401
RPL9	1.04E-27	-0.25594393	0.987	0.995	1.96E-23
ELF1	3.49E-16	-0.255997293	0.682	0.769	6.59E-12
RPS3	1.91E-28	-0.258787509	0.998	0.998	3.60E-24
CYP4X1	1.92E-19	-0.258901609	0.189	0.392	3.63E-15
FOXP1	1.52E-13	-0.25921995	0.74	0.797	2.88E-09
DDAH2	7.65E-16	-0.259540622	0.778	0.827	1.44E-11
TCEA3	1.83E-09	-0.262481558	0.4	0.498	3.45E-05
INSIG2	2.79E-11	-0.263741884	0.364	0.48	5.27E-07
QPRT	5.04E-13	-0.26413391	0.778	0.841	9.52E-09
C4orf3	0.071692754	-0.264434174	0.907	0.922	1
SLC6A6	4.49E-12	-0.26481124	0.344	0.464	8.49E-08
SLC11A2	0.012186951	-0.265642741	0.496	0.484	1
HSD17B11	5.12E-18	-0.265964933	0.811	0.897	9.67E-14
TBPL1	2.26E-19	-0.266229722	0.449	0.605	4.28E-15
MKRN1	4.00E-16	-0.266249689	0.782	0.843	7.56E-12
SRI	1.78E-12	-0.266399537	0.933	0.973	3.36E-08
THRA	3.02E-13	-0.268148047	0.451	0.569	5.71E-09
TAX1BP1	9.78E-15	-0.26834898	0.898	0.946	1.85E-10
RPL3	1.67E-31	-0.26864085	0.993	0.998	3.15E-27
MUC3A	1.03E-09	-0.27103645	0.638	0.687	1.95E-05
AC020656.1	0.758332279	-0.273535583	0.324	0.317	1
KLHDC2	5.39E-19	-0.274131745	0.664	0.762	1.02E-14
RPL41	3.20E-41	-0.274557533	0.998	1	6.05E-37
HOXA9	2.58E-10	-0.276003936	0.576	0.639	4.87E-06

ITGA6	1.51E-11	-0.276826936	0.869	0.926	2.86E-07
RPS12	6.79E-29	-0.279393538	0.996	0.999	1.28E-24
EIF3D	1.41E-14	-0.279509427	0.873	0.922	2.66E-10
SAT1	9.56E-06	-0.279982306	0.964	0.977	0.180547667
EPHB3	2.04E-10	-0.280604935	0.496	0.588	3.86E-06
TBX3	2.16E-10	-0.280809259	0.536	0.629	4.08E-06
EPB41L4A-AS1	6.64E-13	-0.28321565	0.633	0.707	1.25E-08
MUC5B	2.99E-10	-0.283542636	0.271	0.38	5.64E-06
RPS15A	1.65E-35	-0.284272799	0.991	0.997	3.12E-31
HNMT	3.78E-17	-0.284682754	0.58	0.677	7.14E-13
GLUL	2.27E-13	-0.285862309	0.771	0.827	4.29E-09
TES	1.05E-15	-0.28618081	0.627	0.697	1.98E-11
ATP5MC2	1.70E-24	-0.287998305	0.953	0.986	3.22E-20
RPL21	5.67E-26	-0.288311303	0.987	0.998	1.07E-21
RPL15	2.46E-36	-0.292585921	0.991	0.998	4.64E-32
TMEM230	2.86E-15	-0.293498822	0.784	0.832	5.40E-11
POLR1D	2.57E-18	-0.297263196	0.88	0.931	4.85E-14
SORL1	2.09E-15	-0.298767347	0.516	0.618	3.94E-11
TOMM7	3.42E-20	-0.299750558	0.936	0.969	6.45E-16
PROX1	1.71E-13	-0.301518397	0.26	0.414	3.22E-09
RPL17	2.87E-11	-0.302027203	0.907	0.962	5.42E-07
PERP	2.15E-18	-0.302355991	0.944	0.973	4.07E-14
ZFP36L1	3.09E-12	-0.302439035	0.876	0.931	5.84E-08
MPST	4.64E-20	-0.302591577	0.891	0.942	8.77E-16
ALDH1A1	3.56E-14	-0.302599602	0.373	0.537	6.72E-10
ATF3	1.95E-09	-0.303086877	0.413	0.521	3.67E-05
DEFA5	4.67E-08	-0.30360767	0.167	0.272	0.000882644
RPL10A	2.03E-34	-0.30374508	0.991	0.998	3.84E-30
PSAP	3.66E-21	-0.304314856	0.889	0.945	6.92E-17
QTRT1	1.90E-10	-0.305879642	0.787	0.788	3.59E-06
MLXIP	8.19E-15	-0.306728305	0.787	0.851	1.55E-10
SARAF	7.01E-18	-0.307473657	0.831	0.903	1.32E-13
GTF2I	5.25E-17	-0.310327397	0.844	0.868	9.91E-13
CDCA7	3.18E-10	-0.310422217	0.642	0.693	6.00E-06
ZBTB20	1.07E-17	-0.311980001	0.393	0.564	2.03E-13
KLF4	1.03E-12	-0.315660794	0.536	0.63	1.95E-08
SEMA3C	8.79E-20	-0.317410995	0.433	0.588	1.66E-15
RPS18	4.92E-42	-0.323432625	0.998	0.999	9.29E-38
CRNDE	2.40E-23	-0.324319992	0.304	0.531	4.52E-19
SLC25A6	1.02E-29	-0.328498316	0.978	0.989	1.92E-25
KLF5	2.04E-20	-0.32858301	0.9	0.948	3.86E-16
CAMK2N1	6.65E-15	-0.331720628	0.773	0.863	1.26E-10
IGHA1	2.38E-19	-0.331912357	0.416	0.603	4.50E-15

ZFAS1	7.21E-13	-0.332783302	0.956	0.982	1.36E-08
CD9	1.01E-14	-0.333091263	0.942	0.967	1.91E-10
IGBP1	8.24E-20	-0.334441193	0.742	0.827	1.55E-15
FHL2	0.021550075	-0.334610198	0.824	0.838	1
KIAA1324	1.95E-15	-0.335673424	0.56	0.662	3.68E-11
DDIT4	0.175515074	-0.338493501	0.573	0.566	1
RPL36A	1.56E-18	-0.339708045	0.978	0.992	2.95E-14
RNF186	5.44E-15	-0.341378738	0.504	0.64	1.03E-10
RPS6	2.39E-37	-0.341958869	0.989	0.998	4.52E-33
RPS5	9.56E-21	-0.342226617	0.98	0.991	1.80E-16
IRF2BP2	7.88E-25	-0.344303208	0.827	0.898	1.49E-20
RPL10	2.02E-44	-0.344738803	0.996	0.999	3.81E-40
TSPAN8	1.54E-22	-0.345259683	0.984	0.994	2.90E-18
EIF3L	4.71E-20	-0.348319214	0.891	0.932	8.89E-16
SOX4	1.46E-26	-0.349242072	0.904	0.962	2.75E-22
NPDC1	2.43E-19	-0.352011827	0.887	0.928	4.59E-15
AGR3	1.79E-14	-0.352621158	0.8	0.878	3.39E-10
KMT2E	3.39E-24	-0.356623593	0.776	0.86	6.39E-20
CLDN3	6.31E-17	-0.359437957	0.944	0.981	1.19E-12
LGR5	4.92E-19	-0.364893808	0.22	0.412	9.28E-15
TM4SF1	1.76E-06	-0.365667785	0.644	0.696	0.03324714
SMAD9	7.27E-19	-0.366502837	0.244	0.418	1.37E-14
AXIN2	8.55E-14	-0.369993296	0.7	0.772	1.61E-09
AKAP9	3.89E-24	-0.371883199	0.771	0.868	7.35E-20
RPL34	1.22E-43	-0.372274524	0.991	0.998	2.31E-39
EEF1A1	2.83E-49	-0.375505236	0.991	0.998	5.35E-45
ETS2	1.97E-24	-0.378660714	0.869	0.94	3.72E-20
NUPR1	0.005741021	-0.380131635	0.747	0.577	1
IGKC	1.31E-19	-0.383722086	0.544	0.69	2.48E-15
BRI3	1.22E-24	-0.384014651	0.796	0.897	2.31E-20
DUSP1	2.19E-12	-0.384784573	0.673	0.771	4.13E-08
EIF4A2	1.94E-19	-0.386962941	0.942	0.966	3.66E-15
CXADR	3.43E-28	-0.387983993	0.84	0.911	6.47E-24
GDF15	3.52E-08	-0.388657131	0.636	0.729	0.000665132
CLDN4	7.01E-26	-0.389531049	0.92	0.978	1.32E-21
HSPB1	5.43E-06	-0.392835801	0.762	0.807	0.102450848
PRDX5	2.59E-16	-0.399660754	0.96	0.987	4.90E-12
FAM13A	3.85E-08	-0.402707074	0.333	0.425	0.000727219
MUC12	1.55E-09	-0.403901671	0.62	0.687	2.92E-05
RPL13	3.39E-52	-0.406723642	0.998	1	6.40E-48
RNF43	1.08E-22	-0.409837194	0.8	0.88	2.05E-18
TPT1	4.00E-41	-0.410553212	0.993	0.999	7.56E-37
FOS	9.95E-17	-0.41218167	0.902	0.944	1.88E-12

RPL26	6.18E-26	-0.41224809	0.989	0.997	1.17E-21
FOSB	1.18E-20	-0.422056434	0.644	0.764	2.23E-16
RPS27	3.33E-61	-0.425623996	0.998	0.999	6.29E-57
HES1	4.92E-08	-0.435395934	0.784	0.838	0.000928414
CCNI	1.77E-38	-0.436957552	0.929	0.976	3.34E-34
N4BP2L2	1.79E-30	-0.442096528	0.818	0.896	3.38E-26
CA9	0.061831504	-0.448860529	0.391	0.384	1
ERO1A	0.496035516	-0.450291356	0.718	0.635	1
ZNF703	1.37E-27	-0.45660752	0.604	0.761	2.58E-23
RPS4X	4.06E-44	-0.46327566	0.987	0.996	7.67E-40
REPIN1	4.98E-22	-0.465238544	0.749	0.837	9.40E-18
AREG	0.592657451	-0.467952885	0.569	0.495	1
ID4	4.53E-13	-0.468871627	0.309	0.443	8.56E-09
ITPR2	6.61E-22	-0.472241625	0.32	0.52	1.25E-17
PCCA	3.59E-09	-0.472357463	0.536	0.625	6.79E-05
GAS6	1.84E-24	-0.474678271	0.627	0.748	3.47E-20
COMMD6	2.32E-37	-0.475735772	0.942	0.971	4.38E-33
TSC22D1	1.10E-28	-0.484701402	0.862	0.943	2.09E-24
TMEM59	1.41E-43	-0.486005538	0.924	0.974	2.66E-39
BTG1	5.33E-32	-0.491400995	0.784	0.889	1.01E-27
MMP7	2.98E-12	-0.50411653	0.773	0.498	5.63E-08
PRR15	2.06E-29	-0.51845896	0.584	0.763	3.88E-25
TXNIP	5.01E-18	-0.551729664	0.553	0.67	9.45E-14
NOP53	5.95E-40	-0.552151051	0.942	0.966	1.12E-35
ASCL2	2.17E-13	-0.562508202	0.869	0.882	4.09E-09
RGMB	3.66E-29	-0.565175527	0.287	0.514	6.92E-25
DPEP1	3.67E-15	-0.567487912	0.509	0.615	6.93E-11
FAM3D	2.97E-24	-0.568483198	0.867	0.918	5.62E-20
PNRC1	3.48E-46	-0.576722581	0.602	0.809	6.56E-42
ZKSCAN1	3.02E-38	-0.586165057	0.722	0.854	5.71E-34
JUN	4.93E-43	-0.623479551	0.933	0.977	9.30E-39
NKD1	6.90E-23	-0.640001329	0.316	0.533	1.30E-18
ZFP36L2	1.63E-37	-0.68691454	0.893	0.951	3.08E-33
BTG2	5.74E-30	-0.686974671	0.696	0.82	1.08E-25
APCDD1	4.04E-17	-0.69455349	0.298	0.466	7.64E-13
ID3	2.51E-10	-0.697761155	0.78	0.821	4.74E-06
NDRG1	0.000243712	-0.704557829	0.531	0.535	1
SELENBP1	8.75E-10	-0.710325432	0.824	0.829	1.65E-05
C6orf48	3.59E-62	-0.724629709	0.74	0.908	6.78E-58
REG1A	1.95E-11	-1.048781614	0.207	0.339	3.68E-07
LEFTY1	1.32E-33	-1.192747396	0.433	0.638	2.49E-29
**Cluster 5**					
**Gene symbol**	**p_val**	**avg_log2FC**	**pct.1**	**pct.2**	**p_val_adj**

LYZ	1.16E-45	1.027442091	0.94	0.793	2.19E-41
CST3	4.11E-39	0.907462401	0.978	0.979	7.76E-35
STMN1	9.19E-76	0.88265213	0.907	0.553	1.74E-71
CYP2W1	4.54E-107	0.816710485	0.671	0.203	8.58E-103
TUBA1B	9.59E-13	0.813579402	0.916	0.889	1.81E-08
H2AFZ	2.89E-37	0.811008968	0.976	0.93	5.46E-33
AGR2	6.71E-58	0.764940909	0.993	0.981	1.27E-53
PTTG1	1.27E-29	0.763938711	0.496	0.257	2.39E-25
SPINK1	6.08E-37	0.749795951	0.993	0.957	1.15E-32
BST2	6.14E-77	0.738538166	0.693	0.267	1.16E-72
HSPD1	6.82E-51	0.735052145	0.971	0.961	1.29E-46
ANPEP	7.64E-65	0.72037653	0.593	0.206	1.44E-60
IDH2	3.96E-53	0.698626021	0.944	0.858	7.48E-49
CPS1	5.48E-83	0.684147149	0.562	0.172	1.04E-78
CCND1	1.80E-56	0.683779497	0.942	0.711	3.39E-52
PSMA7	5.61E-52	0.678531343	0.993	0.974	1.06E-47
YWHAB	2.16E-63	0.669943597	0.98	0.953	4.08E-59
UBE2C	7.77E-28	0.66871459	0.34	0.137	1.47E-23
AC020656.1	4.30E-28	0.665225628	0.547	0.287	8.11E-24
OLFM4	2.06E-10	0.657599831	0.929	0.896	3.89E-06
HSP90AA1	2.73E-53	0.653520509	0.993	0.986	5.16E-49
C4orf48	1.90E-70	0.651377435	0.976	0.814	3.60E-66
REG4	4.16E-27	0.644774036	0.569	0.314	7.85E-23
ENY2	1.79E-70	0.639259251	0.964	0.89	3.38E-66
HSPE1	1.50E-47	0.635877847	0.984	0.976	2.84E-43
FTL	6.36E-95	0.635364045	1	0.995	1.20E-90
ANXA13	1.81E-92	0.633377384	0.736	0.263	3.41E-88
HMGN2	2.90E-18	0.631913153	0.967	0.95	5.48E-14
TUBB4B	2.69E-34	0.6318242	0.942	0.847	5.08E-30
SQLE	1.37E-52	0.629483515	0.802	0.479	2.58E-48
EIF6	1.14E-64	0.62728776	0.964	0.886	2.15E-60
TSTA3	1.32E-65	0.623059758	0.947	0.796	2.49E-61
RAN	2.35E-55	0.623044435	0.991	0.965	4.44E-51
ZFAS1	2.96E-35	0.618647693	0.998	0.976	5.59E-31
EEF1D	1.91E-89	0.616647559	0.998	0.987	3.61E-85
TGFBI	3.23E-35	0.611695347	0.856	0.621	6.11E-31
HSP90AB1	1.52E-56	0.602327648	0.991	0.982	2.88E-52
ATP5F1E	6.87E-65	0.598578634	1	0.99	1.30E-60
NAA20	5.78E-13	0.587490784	0.847	0.745	1.09E-08
PCLAF	2.06E-27	0.569982091	0.511	0.28	3.89E-23
PRDX2	3.72E-53	0.565045666	0.978	0.957	7.02E-49
BSG	1.24E-63	0.564908372	0.991	0.955	2.34E-59
RAB5IF	4.51E-47	0.562893134	0.949	0.817	8.52E-43

TPD52	3.61E-61	0.561776435	0.98	0.925	6.81E-57
NPM1	2.85E-47	0.556287439	0.993	0.986	5.38E-43
JPT1	1.79E-42	0.55627773	0.96	0.802	3.38E-38
TUBB	2.79E-30	0.555751767	0.956	0.84	5.26E-26
SMIM24	8.11E-74	0.555216257	0.629	0.225	1.53E-69
CCNB1	1.15E-28	0.551707563	0.384	0.17	2.16E-24
AREG	2.03E-40	0.54398801	0.778	0.466	3.84E-36
VDAC1	1.85E-52	0.535627221	0.991	0.966	3.49E-48
NDUFB9	1.23E-57	0.533343474	0.98	0.963	2.32E-53
PABPC1	6.30E-65	0.531659304	1	0.994	1.19E-60
FCGRT	1.14E-42	0.531342583	0.942	0.847	2.16E-38
PRSS2	2.39E-40	0.530217612	0.253	0.063	4.51E-36
PUF60	1.60E-60	0.528688051	0.911	0.711	3.02E-56
GLO1	1.60E-36	0.523014154	0.907	0.788	3.03E-32
FDPS	2.53E-31	0.518870547	0.909	0.763	4.78E-27
CYC1	1.72E-40	0.518835139	0.982	0.949	3.24E-36
CKS2	3.29E-28	0.50559172	0.833	0.667	6.21E-24
GSTM3	7.86E-68	0.504574973	0.7	0.307	1.48E-63
EIF2S2	4.33E-46	0.502745992	0.973	0.888	8.18E-42
GNAS	6.04E-48	0.498618487	0.976	0.937	1.14E-43
MKI67	5.89E-30	0.494940027	0.389	0.168	1.11E-25
MMP7	3.65E-60	0.490470025	0.896	0.481	6.88E-56
LGALS4	2.17E-19	0.489213538	0.993	0.989	4.09E-15
ROMO1	2.73E-43	0.487009615	0.98	0.921	5.15E-39
DPM1	1.53E-41	0.485652874	0.844	0.65	2.90E-37
CLDN2	3.93E-15	0.4802442	0.722	0.573	7.43E-11
YWHAZ	4.86E-57	0.478452038	0.996	0.962	9.18E-53
COTL1	2.96E-46	0.474426253	0.84	0.555	5.58E-42
AZGP1	9.48E-57	0.471636259	0.662	0.279	1.79E-52
TUBA1C	2.70E-25	0.470600007	0.887	0.747	5.09E-21
MYC	1.94E-21	0.469001679	0.807	0.661	3.66E-17
PRAP1	6.25E-54	0.468330856	0.829	0.456	1.18E-49
KRT19	8.50E-33	0.467641066	0.991	0.96	1.61E-28
BMP4	1.30E-16	0.466580163	0.624	0.433	2.46E-12
UGDH	4.62E-42	0.462326211	0.851	0.614	8.72E-38
TAF7	3.94E-35	0.460211825	0.938	0.832	7.44E-31
HMGB2	0.532045121	0.459764668	0.553	0.598	1
EIF3E	5.23E-47	0.459715296	0.98	0.97	9.87E-43
RBP2	1.48E-66	0.45727736	0.424	0.112	2.79E-62
EBP	1.96E-29	0.457230994	0.84	0.703	3.70E-25
RPL8	3.14E-71	0.45194157	1	0.999	5.94E-67
SLC39A4	2.20E-40	0.451209077	0.896	0.718	4.15E-36
NDUFC2	7.60E-42	0.450093003	0.971	0.95	1.44E-37

ADRM1	7.06E-30	0.449774543	0.909	0.791	1.33E-25
RBM39	3.94E-36	0.44876331	0.987	0.936	7.43E-32
GCHFR	5.61E-33	0.448249841	0.733	0.501	1.06E-28
NCL	2.21E-25	0.447676439	0.964	0.936	4.18E-21
RANBP1	2.05E-17	0.446485213	0.927	0.893	3.87E-13
SOD1	4.96E-33	0.444752832	0.989	0.98	9.36E-29
TIMP1	2.00E-28	0.443448925	0.947	0.747	3.78E-24
KPNA2	2.27E-37	0.442253549	0.607	0.306	4.29E-33
PDIA3	6.98E-31	0.442245184	0.976	0.943	1.32E-26
MAP3K20	6.99E-31	0.439610083	0.784	0.549	1.32E-26
ACAT2	3.66E-23	0.438366803	0.691	0.495	6.91E-19
TOP1	1.12E-43	0.438061907	0.904	0.71	2.12E-39
CDKN3	1.81E-31	0.437510783	0.413	0.186	3.42E-27
ZNF706	3.80E-53	0.436759928	0.978	0.918	7.17E-49
MRPL13	2.23E-41	0.436356347	0.936	0.799	4.21E-37
FABP1	7.08E-53	0.436065203	0.849	0.435	1.34E-48
SLIRP	1.49E-36	0.434936177	0.973	0.938	2.82E-32
CCT5	8.12E-33	0.434790901	0.933	0.841	1.53E-28
LDHB	6.80E-34	0.433913759	0.838	0.47	1.28E-29
PGAM1	2.70E-27	0.433717533	0.933	0.818	5.10E-23
HES6	2.13E-18	0.432370792	0.749	0.567	4.03E-14
CEBPB	5.52E-29	0.431390103	0.898	0.745	1.04E-24
RPS21	3.04E-58	0.430164987	1	0.993	5.74E-54
ATP5IF1	5.89E-38	0.428684625	0.962	0.943	1.11E-33
PIK3R1	1.66E-28	0.427586299	0.791	0.601	3.13E-24
TPI1	2.27E-30	0.427378191	0.993	0.986	4.28E-26
RPN2	4.08E-30	0.42567742	0.913	0.852	7.71E-26
TMED9	2.70E-37	0.425657824	0.956	0.883	5.09E-33
MIF	4.03E-39	0.422744531	0.989	0.973	7.61E-35
CDKN2A	2.79E-85	0.422730466	0.68	0.228	5.27E-81
PTGR1	7.44E-44	0.422181342	0.813	0.538	1.41E-39
ANP32A	8.54E-35	0.41893528	0.882	0.772	1.61E-30
TMEM97	7.72E-42	0.418637977	0.736	0.436	1.46E-37
ELOC	6.54E-41	0.418405443	0.938	0.862	1.23E-36
DBI	9.75E-18	0.417490135	0.96	0.959	1.84E-13
ENO1	5.65E-25	0.41724283	0.976	0.961	1.07E-20
SCD	2.55E-61	0.417080841	0.896	0.504	4.81E-57
RAD21	1.85E-26	0.416408455	0.858	0.693	3.48E-22
IDH1	1.21E-40	0.41532791	0.893	0.713	2.29E-36
EIF3H	5.24E-53	0.41452136	0.993	0.963	9.89E-49
AKR1B10	9.19E-44	0.413245635	0.518	0.217	1.73E-39
TMSB10	1.49E-43	0.408391049	0.998	0.998	2.82E-39
HNRNPA2B1	3.29E-20	0.408194834	0.993	0.971	6.21E-16

MTDH	2.04E-37	0.408088131	0.971	0.922	3.86E-33
RPS20	2.28E-82	0.407450996	1	0.994	4.30E-78
ATP5ME	8.06E-33	0.404911265	0.991	0.98	1.52E-28
BIN1	1.26E-35	0.404198632	0.842	0.665	2.39E-31
FXYD5	6.82E-46	0.402383748	0.889	0.498	1.29E-41
MARCKSL1	3.16E-23	0.398532988	0.989	0.97	5.96E-19
PHLDA2	1.27E-29	0.397730051	0.88	0.621	2.40E-25
CFL1	2.34E-37	0.397152794	0.998	0.994	4.41E-33
THEM6	1.85E-55	0.396831575	0.733	0.387	3.50E-51
OAZ1	9.70E-46	0.396778079	0.998	0.986	1.83E-41
CTSC	8.49E-60	0.394562386	0.74	0.356	1.60E-55
FBXO2	7.10E-69	0.394428754	0.622	0.207	1.34E-64
CTSH	1.13E-33	0.392215677	0.869	0.673	2.14E-29
GPAA1	4.32E-33	0.392067073	0.838	0.617	8.16E-29
TCP1	1.83E-27	0.391807675	0.884	0.786	3.46E-23
GSTA1	1.60E-69	0.391482305	0.396	0.095	3.03E-65
MRFAP1	6.96E-34	0.390815227	0.924	0.857	1.31E-29
ANXA4	1.06E-29	0.390392191	0.942	0.876	2.00E-25
APLP2	1.45E-37	0.389773259	0.927	0.765	2.74E-33
SULT1E1	9.58E-58	0.389547544	0.32	0.074	1.81E-53
OTULINL	8.29E-39	0.388708023	0.756	0.49	1.57E-34
CPNE1	2.14E-30	0.388479554	0.824	0.629	4.03E-26
TRAM1	1.44E-45	0.388169859	0.916	0.712	2.72E-41
PHF20L1	1.09E-36	0.386841656	0.822	0.572	2.06E-32
DEPTOR	4.66E-50	0.386587199	0.638	0.304	8.81E-46
IDI1	6.72E-20	0.38647803	0.702	0.52	1.27E-15
SLC52A2	2.63E-34	0.386223912	0.844	0.643	4.97E-30
SYNCRIP	3.32E-34	0.385811616	0.927	0.782	6.28E-30
PRELID3B	8.35E-32	0.384612161	0.904	0.754	1.58E-27
TTR	1.74E-52	0.384013188	0.351	0.095	3.29E-48
YBX1	7.19E-35	0.381416456	0.993	0.993	1.36E-30
RPS4Y1	1.93E-20	0.380405268	0.856	0.837	3.64E-16
MGST2	4.84E-28	0.380155993	0.94	0.899	9.13E-24
CALM2	2.33E-28	0.379503639	0.989	0.981	4.41E-24
PRDX1	3.71E-28	0.379279428	0.991	0.971	7.00E-24
H2AFY	3.73E-35	0.378433866	0.976	0.939	7.03E-31
XBP1	2.32E-30	0.377650775	0.94	0.831	4.38E-26
TRMT112	5.95E-34	0.377233056	0.964	0.908	1.12E-29
FUOM	2.07E-50	0.376515696	0.687	0.348	3.90E-46
CNIH4	7.51E-34	0.376112481	0.882	0.709	1.42E-29
PPP2R1A	3.31E-35	0.375330158	0.94	0.831	6.24E-31
RABL6	6.67E-28	0.374608284	0.887	0.766	1.26E-23
HMGB3	7.30E-29	0.373589794	0.784	0.57	1.38E-24

AURKA	8.79E-35	0.371119148	0.4	0.162	1.66E-30
IFI6	1.95E-06	0.370795891	0.531	0.422	0.036739681
GRPEL1	1.27E-29	0.36949816	0.784	0.569	2.40E-25
CTSV	1.40E-59	0.368165225	0.549	0.197	2.63E-55
AHCY	7.63E-26	0.368082282	0.878	0.783	1.44E-21
MAL2	4.01E-38	0.36751897	0.949	0.821	7.57E-34
KIF5B	1.08E-28	0.366994315	0.982	0.947	2.04E-24
PRELID1	3.48E-21	0.366223156	0.962	0.951	6.57E-17
NAPRT	1.56E-29	0.361836238	0.88	0.716	2.94E-25
LACTB2	1.02E-31	0.361052116	0.767	0.515	1.93E-27
SOX4	0.004422637	0.3608799	0.958	0.955	1
CES2	3.26E-20	0.360267068	0.68	0.467	6.16E-16
GID8	5.93E-30	0.35926921	0.844	0.645	1.12E-25
HSPA1B	9.23E-26	0.358404038	0.809	0.578	1.74E-21
SUB1	4.42E-31	0.3578453	0.993	0.971	8.34E-27
UBE2V2	8.80E-37	0.357027443	0.891	0.73	1.66E-32
NPC2	4.81E-35	0.356735012	0.978	0.93	9.08E-31
KRT20	4.70E-36	0.355702891	0.824	0.497	8.88E-32
COMT	1.53E-34	0.354147467	0.94	0.821	2.89E-30
CDC20	2.38E-32	0.35285162	0.322	0.116	4.49E-28
BIRC5	1.47E-19	0.351371542	0.349	0.175	2.78E-15
NME1	6.99E-18	0.348980157	0.876	0.813	1.32E-13
ERH	1.10E-26	0.348212678	0.98	0.939	2.08E-22
GSTP1	6.07E-33	0.347058658	0.996	0.991	1.15E-28
SEPHS2	7.03E-25	0.34663889	0.856	0.743	1.33E-20
PSAT1	6.33E-39	0.346228374	0.522	0.226	1.19E-34
MRPS21	1.65E-43	0.346089878	0.813	0.439	3.12E-39
DCAF13	1.76E-33	0.345938827	0.767	0.542	3.33E-29
EXOSC4	7.05E-36	0.345712572	0.8	0.549	1.33E-31
PGK1	2.31E-24	0.34493233	0.944	0.888	4.36E-20
RPL7	8.64E-59	0.344775956	1	0.997	1.63E-54
MAD2L1	9.86E-22	0.343893048	0.422	0.226	1.86E-17
CACYBP	2.69E-23	0.342687368	0.869	0.756	5.08E-19
ARL6IP1	1.37E-06	0.341794058	0.902	0.88	0.025939457
MANF	2.85E-27	0.341711187	0.884	0.7	5.38E-23
NUPR1	3.48E-45	0.341614709	0.902	0.555	6.57E-41
H2AFY2	5.99E-67	0.339771399	0.611	0.227	1.13E-62
NDUFAB1	5.86E-22	0.338913343	0.953	0.931	1.11E-17
FABP6	1.94E-83	0.338640246	0.553	0.158	3.66E-79
HACD3	3.58E-22	0.337723055	0.82	0.721	6.76E-18
NUDC	1.04E-22	0.335625937	0.913	0.833	1.97E-18
DCUN1D5	3.57E-36	0.335608633	0.722	0.46	6.74E-32
HSP90B1	5.08E-26	0.335211779	0.987	0.949	9.59E-22

CISD1	2.31E-27	0.334836214	0.893	0.811	4.36E-23
PDIA6	1.40E-22	0.334678509	0.967	0.944	2.64E-18
PSMB5	3.70E-33	0.334435216	0.953	0.897	6.99E-29
SNRPF	2.18E-23	0.334078811	0.956	0.918	4.12E-19
RRM2	3.81E-25	0.333743212	0.356	0.153	7.20E-21
ISG15	1.93E-16	0.333207746	0.676	0.457	3.64E-12
SCAND1	7.34E-25	0.332648856	0.958	0.872	1.39E-20
ODC1	4.76E-20	0.3322446	0.842	0.731	9.00E-16
CENPF	2.85E-20	0.331961399	0.333	0.159	5.38E-16
PA2G4	6.12E-20	0.331852217	0.949	0.906	1.16E-15
TMA7	3.56E-29	0.331395629	0.998	0.987	6.72E-25
CCT2	8.79E-24	0.331243995	0.918	0.836	1.66E-19
MPC2	2.57E-26	0.331169274	0.951	0.933	4.85E-22
DYNC1I2	6.89E-29	0.330898391	0.938	0.847	1.30E-24
NORAD	8.66E-20	0.33074778	0.898	0.79	1.64E-15
LRPAP1	5.24E-30	0.33060679	0.898	0.734	9.90E-26
BAG2	5.14E-80	0.330421504	0.547	0.16	9.70E-76
CBX1	2.73E-29	0.330062607	0.736	0.511	5.15E-25
HSD17B12	4.00E-25	0.329998154	0.916	0.837	7.55E-21
MALL	1.58E-45	0.329606081	0.684	0.305	2.98E-41
GCSH	9.47E-27	0.329528385	0.849	0.672	1.79E-22
COX6C	6.65E-29	0.326564134	0.996	0.99	1.26E-24
REEP5	3.20E-26	0.325424377	0.92	0.828	6.04E-22
ACOT7	2.84E-33	0.325083264	0.669	0.389	5.36E-29
HNRNPM	5.45E-20	0.324000051	0.898	0.826	1.03E-15
SFN	3.30E-19	0.32300788	0.842	0.656	6.23E-15
PDCD5	1.32E-28	0.32227268	0.929	0.867	2.50E-24
ANXA5	4.36E-29	0.322172584	0.804	0.522	8.24E-25
KRT10	1.95E-34	0.321945951	0.942	0.782	3.67E-30
PSMA4	3.13E-23	0.321694681	0.958	0.929	5.90E-19
SNRPG	8.75E-21	0.321577779	0.976	0.939	1.65E-16
SLC16A1	3.44E-62	0.321401734	0.593	0.227	6.50E-58
MTHFD2	4.05E-17	0.321335587	0.709	0.484	7.65E-13
SRSF7	2.58E-18	0.321190157	0.911	0.834	4.87E-14
KRT8	3.50E-16	0.320504079	0.998	0.996	6.60E-12
SKP1	2.01E-21	0.3197401	0.993	0.971	3.79E-17
LSM4	8.42E-17	0.319724046	0.938	0.876	1.59E-12
STRAP	1.36E-22	0.319662307	0.891	0.785	2.56E-18
EREG	1.11E-32	0.319405631	0.364	0.136	2.10E-28
NT5DC2	3.41E-64	0.317730753	0.553	0.198	6.44E-60
PRKDC	5.27E-21	0.317008969	0.807	0.67	9.95E-17
SET	1.15E-20	0.316442177	0.982	0.957	2.18E-16
CA9	1.59E-46	0.315912239	0.724	0.337	3.00E-42

HM13	3.51E-29	0.315081644	0.884	0.693	6.63E-25
RNF114	1.27E-32	0.314813621	0.918	0.719	2.40E-28
WDR1	1.86E-24	0.314619932	0.92	0.788	3.51E-20
SMC4	1.06E-15	0.314003192	0.596	0.421	2.00E-11
NUSAP1	2.56E-21	0.313335518	0.293	0.124	4.84E-17
AHSA1	1.07E-24	0.313313556	0.856	0.691	2.02E-20
FKBP4	4.59E-25	0.313030068	0.836	0.705	8.67E-21
AGO2	1.83E-36	0.312127378	0.773	0.482	3.46E-32
VPS28	7.97E-25	0.312101507	0.962	0.89	1.51E-20
CCNB2	1.14E-24	0.31194775	0.324	0.137	2.15E-20
EIF5	5.73E-18	0.311551628	0.944	0.877	1.08E-13
TRABD2A	2.95E-28	0.310429479	0.767	0.529	5.57E-24
SSB	7.27E-22	0.310145097	0.936	0.861	1.37E-17
RHOBTB3	7.72E-23	0.309824895	0.829	0.645	1.46E-18
TXN	1.07E-34	0.309533546	0.996	0.992	2.02E-30
TIMM8B	3.63E-24	0.308988011	0.918	0.814	6.86E-20
ASPH	2.57E-21	0.307211175	0.907	0.761	4.85E-17
EEF1B2	5.01E-26	0.307181556	0.993	0.986	9.46E-22
LGALS3	4.51E-14	0.306867023	0.993	0.989	8.52E-10
HINT1	4.19E-35	0.306350073	0.996	0.989	7.92E-31
LYAR	1.70E-22	0.305751887	0.616	0.405	3.22E-18
LGALS2	4.31E-42	0.305504301	0.587	0.256	8.13E-38
DPP7	5.44E-33	0.305130848	0.838	0.581	1.03E-28
UBE2S	3.78E-19	0.304797521	0.582	0.375	7.14E-15
TM7SF2	9.43E-24	0.303874281	0.687	0.447	1.78E-19
CSE1L	6.24E-27	0.303145401	0.636	0.393	1.18E-22
LAPTM4B	3.23E-22	0.302976912	0.82	0.661	6.11E-18
NDUFS5	5.98E-27	0.302201596	0.991	0.975	1.13E-22
MYBL2	4.70E-28	0.301488447	0.344	0.142	8.88E-24
GSS	1.55E-32	0.30145041	0.753	0.503	2.93E-28
CFD	1.38E-24	0.301252437	0.64	0.377	2.60E-20
DDX5	1.62E-23	0.300804442	0.973	0.951	3.07E-19
PAFAH1B3	2.01E-26	0.300389613	0.889	0.756	3.80E-22
RAE1	1.19E-28	0.300307551	0.611	0.351	2.25E-24
CYP27A1	9.14E-73	0.29992613	0.538	0.163	1.73E-68
PTGES3	2.32E-17	0.299408464	0.976	0.961	4.39E-13
LBR	1.52E-22	0.298987172	0.767	0.57	2.87E-18
UBE2L3	6.79E-24	0.298324549	0.938	0.827	1.28E-19
SNRPD1	1.80E-16	0.297426851	0.931	0.891	3.39E-12
ATP6V0B	1.99E-21	0.2972605	0.927	0.851	3.76E-17
CDK4	7.11E-23	0.296265546	0.853	0.718	1.34E-18
LDHA	3.82E-11	0.295553815	0.962	0.973	7.21E-07
NIPSNAP1	2.31E-29	0.295474893	0.669	0.413	4.36E-25

EIF4G2	2.41E-18	0.295295735	0.969	0.942	4.55E-14
HNRNPC	9.91E-20	0.294503415	0.978	0.961	1.87E-15
TBCA	2.59E-25	0.293875166	0.982	0.943	4.89E-21
SAPCD2	2.60E-35	0.293118977	0.54	0.261	4.90E-31
ETFB	9.98E-20	0.292365728	0.94	0.895	1.89E-15
HSPA8	8.30E-09	0.292105118	0.969	0.962	0.000156679
ARF1	1.20E-20	0.29113413	0.969	0.912	2.27E-16
CCT8	1.09E-19	0.291090961	0.911	0.888	2.05E-15
MAPRE1	2.37E-28	0.290949577	0.802	0.56	4.48E-24
MISP	0.010010894	0.290666787	0.864	0.815	1
TATDN1	1.75E-23	0.290560629	0.864	0.695	3.31E-19
S100A10	1.77E-24	0.290301931	0.996	0.995	3.34E-20
NELFCD	7.27E-29	0.289752248	0.798	0.586	1.37E-24
FAM84B	1.54E-34	0.289547963	0.702	0.404	2.90E-30
HNRNPA3	3.78E-15	0.289200216	0.96	0.921	7.14E-11
PSMB2	2.52E-21	0.288381352	0.947	0.855	4.75E-17
FDX1	1.46E-22	0.288350369	0.818	0.684	2.76E-18
SMIM31	3.48E-28	0.287625964	0.698	0.415	6.58E-24
RPL30	3.50E-39	0.287271873	1	0.996	6.62E-35
S100A11	1.17E-25	0.286914884	0.996	0.988	2.21E-21
TOP2A	5.33E-21	0.286853128	0.291	0.124	1.01E-16
SLBP	2.92E-14	0.286760137	0.713	0.569	5.52E-10
TMED10	7.95E-25	0.285824864	0.942	0.853	1.50E-20
SDCBP	3.03E-13	0.285784178	0.929	0.843	5.73E-09
EPB41L2	8.79E-17	0.285290868	0.851	0.688	1.66E-12
ABRACL	7.38E-23	0.285201046	0.9	0.845	1.39E-18
HIST1H4C	0.339919247	0.284799303	0.844	0.808	1
PSMA5	3.02E-22	0.284281891	0.933	0.838	5.71E-18
ACSL3	4.47E-32	0.283350359	0.76	0.478	8.45E-28
MSMO1	4.02E-20	0.283338405	0.656	0.427	7.60E-16
HES4	3.45E-31	0.283056159	0.516	0.25	6.51E-27
SNRPE	7.55E-24	0.282564343	0.967	0.949	1.43E-19
PDRG1	1.08E-40	0.282432882	0.613	0.313	2.05E-36
TSPAN3	2.81E-27	0.281775838	0.933	0.809	5.30E-23
PLCB3	4.00E-32	0.280268805	0.722	0.452	7.56E-28
ARPC5L	6.70E-21	0.279977059	0.849	0.692	1.27E-16
SUMO3	5.39E-20	0.279928234	0.836	0.697	1.02E-15
MRPL36	3.56E-20	0.279554867	0.847	0.722	6.73E-16
QDPR	8.73E-25	0.279350647	0.778	0.562	1.65E-20
PRR15L	7.73E-26	0.279206182	0.869	0.651	1.46E-21
EMP2	5.65E-18	0.278542728	0.824	0.661	1.07E-13
C8orf59	1.19E-18	0.278480068	0.947	0.89	2.25E-14
MAF1	1.77E-22	0.278227865	0.882	0.762	3.35E-18

IRF8	7.38E-43	0.278207736	0.564	0.258	1.39E-38
IQGAP2	1.20E-30	0.278063433	0.616	0.345	2.27E-26
ANP32B	4.29E-13	0.277448614	0.969	0.926	8.11E-09
HSPA4	1.77E-26	0.277088011	0.74	0.51	3.34E-22
TPX2	8.00E-23	0.277018331	0.316	0.137	1.51E-18
HSPA9	8.27E-15	0.277003455	0.891	0.836	1.56E-10
PRMT1	7.30E-19	0.276945507	0.882	0.77	1.38E-14
DDIT4	2.66E-09	0.27680863	0.707	0.547	5.02E-05
HNRNPA0	1.24E-16	0.276680843	0.949	0.899	2.34E-12
AKIRIN1	5.33E-26	0.275866983	0.744	0.504	1.01E-21
HDAC2	2.71E-20	0.275279929	0.88	0.798	5.12E-16
GRINA	4.44E-25	0.274795456	0.72	0.478	8.38E-21
TNFRSF12A	5.81E-45	0.273842472	0.896	0.546	1.10E-40
CENPW	5.98E-05	0.273817279	0.507	0.436	1
CANX	5.42E-17	0.273796492	0.956	0.935	1.02E-12
EMC2	9.96E-29	0.273769517	0.733	0.479	1.88E-24
STIP1	1.27E-21	0.272722097	0.738	0.52	2.40E-17
PRSS3	5.85E-17	0.272702671	0.938	0.844	1.10E-12
ZWINT	5.69E-13	0.27246866	0.427	0.276	1.07E-08
SBSPON	6.86E-34	0.272192667	0.382	0.149	1.30E-29
AC020656.2	4.51E-40	0.27204737	0.324	0.103	8.51E-36
PTMA	1.42E-14	0.271948482	0.998	0.998	2.69E-10
P4HB	8.39E-14	0.271570706	0.969	0.951	1.58E-09
TMEM70	7.62E-23	0.271485757	0.744	0.54	1.44E-18
MGST1	7.21E-15	0.271026471	0.967	0.947	1.36E-10
TLE2	2.42E-45	0.270681338	0.629	0.293	4.57E-41
ANP32E	9.24E-16	0.270437021	0.596	0.414	1.74E-11
MARCKS	1.84E-19	0.270155747	0.933	0.854	3.47E-15
DHRS7	5.73E-14	0.269936181	0.887	0.8	1.08E-09
SRSF3	1.66E-14	0.269097636	0.962	0.938	3.13E-10
NXF3	1.81E-58	0.268774765	0.36	0.093	3.42E-54
MORF4L2	6.88E-20	0.268028046	0.911	0.812	1.30E-15
NOP10	9.15E-26	0.268012092	0.971	0.903	1.73E-21
ARPC2	1.85E-20	0.267473488	0.982	0.954	3.49E-16
FAM49B	3.03E-28	0.267431719	0.827	0.589	5.73E-24
TIMM17A	4.50E-20	0.26643218	0.804	0.632	8.49E-16
GPX4	1.72E-19	0.264890636	0.982	0.956	3.25E-15
IER2	7.55E-08	0.264816276	0.951	0.895	0.001426222
SNRPA1	5.24E-16	0.262898331	0.756	0.615	9.90E-12
PAIP2	3.53E-20	0.262828937	0.884	0.746	6.67E-16
MFSD10	5.74E-19	0.262790594	0.873	0.742	1.08E-14
DHCR7	6.97E-22	0.262466011	0.613	0.393	1.32E-17
C19orf48	1.12E-15	0.262131181	0.791	0.609	2.11E-11

CLDN15	5.07E-22	0.262044423	0.682	0.456	9.57E-18
ATP1B3	4.00E-17	0.261357716	0.913	0.852	7.55E-13
CENPM	1.68E-20	0.260920487	0.4	0.208	3.18E-16
SUPT4H1	5.16E-18	0.260838129	0.824	0.652	9.75E-14
CACNA1D	3.19E-37	0.260686632	0.484	0.21	6.03E-33
DDX21	2.91E-16	0.260361865	0.878	0.801	5.49E-12
TSPAN7	3.30E-61	0.259932665	0.527	0.179	6.24E-57
DNAJA1	4.88E-14	0.259423732	0.884	0.805	9.22E-10
BOP1	2.31E-21	0.25918649	0.682	0.472	4.36E-17
PKM	4.51E-14	0.258936373	0.984	0.953	8.52E-10
CCT6A	2.66E-17	0.258332799	0.951	0.91	5.02E-13
PACSIN3	2.74E-61	0.258005017	0.438	0.131	5.17E-57
DMKN	2.02E-27	0.257945021	0.411	0.188	3.81E-23
CNPY2	5.75E-16	0.257149892	0.911	0.863	1.09E-11
MBIP	3.44E-22	0.25706926	0.689	0.459	6.49E-18
TYMS	4.52E-16	0.256986376	0.453	0.274	8.54E-12
BLOC1S4	1.12E-24	0.256735736	0.778	0.556	2.12E-20
CKB	1.88E-14	0.256155359	0.951	0.87	3.55E-10
NDUFA2	4.91E-14	0.256113363	0.947	0.897	9.27E-10
HNRNPF	4.09E-16	0.255732095	0.951	0.917	7.73E-12
UBL5	4.93E-25	0.255715038	0.989	0.972	9.32E-21
MORF4L1	3.86E-18	0.255611622	0.958	0.927	7.28E-14
CCNG1	1.35E-18	0.255506281	0.902	0.809	2.56E-14
LSM3	1.64E-12	0.255466739	0.927	0.874	3.09E-08
PPIB	6.56E-17	0.255423161	0.976	0.959	1.24E-12
LRRC59	6.80E-22	0.255356661	0.816	0.625	1.28E-17
HNRNPU	2.04E-19	0.254766205	0.969	0.944	3.86E-15
FUS	2.69E-16	0.254521258	0.947	0.872	5.08E-12
ELAVL1	1.90E-19	0.254012897	0.809	0.648	3.59E-15
LMNB1	4.66E-17	0.253452691	0.5	0.317	8.80E-13
NOP56	1.04E-17	0.253357943	0.798	0.643	1.95E-13
PODXL2	6.50E-35	0.253304565	0.571	0.289	1.23E-30
BZW1	2.17E-17	0.253190836	0.922	0.807	4.09E-13
TCEA1	1.61E-13	0.25243801	0.936	0.875	3.04E-09
TPRKB	1.43E-18	0.252403126	0.836	0.686	2.70E-14
SLC25A39	1.75E-12	0.251458377	0.86	0.812	3.30E-08
HMGB1	6.15E-05	0.251397151	0.982	0.987	1
UQCRQ	6.99E-17	0.250953747	0.996	0.985	1.32E-12
REEP6	4.52E-39	0.250787755	0.602	0.294	8.54E-35
ME1	7.04E-39	0.250772238	0.567	0.259	1.33E-34
KPNB1	1.00E-14	0.250769717	0.873	0.768	1.89E-10
PPP1R1B	1.32E-13	0.250506809	0.92	0.921	2.50E-09
DTYMK	1.53E-09	0.250496241	0.587	0.466	2.89E-05

CCT4	1.97E-15	0.250456952	0.904	0.833	3.73E-11
TUBA4A	6.45E-22	0.250144048	0.667	0.435	1.22E-17
THRA	3.64E-08	-0.250052517	0.522	0.559	0.00068776
CD59	5.15E-08	-0.250293141	0.813	0.824	0.000971709
SQOR	2.69E-12	-0.250382212	0.687	0.727	5.08E-08
ANKRD22	1.41E-12	-0.251162795	0.429	0.538	2.66E-08
EFNA3	1.26E-18	-0.252827819	0.16	0.349	2.37E-14
NFKBIZ	4.51E-10	-0.253192522	0.407	0.492	8.51E-06
PLCB4	3.95E-07	-0.253391635	0.653	0.679	0.007463485
CTSB	1.44E-12	-0.253728626	0.802	0.84	2.73E-08
HIBADH	4.51E-15	-0.254123949	0.564	0.656	8.51E-11
FOXP1	5.40E-11	-0.25442887	0.767	0.794	1.02E-06
TFCP2L1	6.97E-34	-0.255965551	0.013	0.28	1.32E-29
CYP4X1	3.18E-11	-0.257590228	0.26	0.382	6.00E-07
MET	2.30E-11	-0.258197792	0.498	0.58	4.33E-07
SERPINB1	8.56E-14	-0.258790485	0.942	0.94	1.62E-09
PFN2	1.65E-31	-0.259244906	0.027	0.284	3.12E-27
YPEL3	3.34E-23	-0.260906277	0.158	0.38	6.30E-19
LGR5	4.25E-11	-0.26101633	0.258	0.407	8.02E-07
NPDC1	1.28E-09	-0.263782011	0.938	0.921	2.41E-05
RPL21	1.87E-26	-0.264056381	1	0.996	3.53E-22
MRPS33	1.81E-11	-0.264149993	0.889	0.888	3.42E-07
RAC1	2.74E-18	-0.264234085	0.991	0.988	5.18E-14
ZNHIT1	2.79E-14	-0.264704	0.918	0.928	5.27E-10
PTEN	2.40E-19	-0.2664816	0.373	0.558	4.53E-15
NCOR1	2.91E-14	-0.266887952	0.778	0.819	5.49E-10
PPP1R14D	1.27E-12	-0.268705254	0.58	0.666	2.40E-08
YWHAE	9.72E-18	-0.269189268	0.958	0.967	1.83E-13
UBL3	1.04E-15	-0.26948438	0.424	0.557	1.97E-11
MVP	1.34E-13	-0.270897012	0.642	0.724	2.53E-09
MLXIP	2.04E-11	-0.271083227	0.822	0.846	3.86E-07
GABARAP	1.49E-14	-0.271341223	0.653	0.709	2.81E-10
TMEM219	3.39E-17	-0.274300154	0.889	0.891	6.40E-13
IFT172	8.19E-16	-0.2747086	0.491	0.62	1.55E-11
MYADM	9.34E-26	-0.275968719	0.16	0.418	1.76E-21
HSBP1L1	3.56E-17	-0.276689907	0.502	0.632	6.73E-13
ETS2	6.41E-14	-0.27717626	0.911	0.934	1.21E-09
AIFM3	9.65E-29	-0.278340326	0.1	0.352	1.82E-24
CEACAM5	0.000771285	-0.278409282	0.869	0.893	1
IMPDH2	9.28E-12	-0.278653696	0.896	0.901	1.75E-07
CYBA	3.39E-14	-0.279353291	0.951	0.963	6.41E-10
NUDT4	3.15E-14	-0.279620268	0.809	0.851	5.95E-10
EEPD1	1.03E-21	-0.282256603	0.193	0.397	1.94E-17

RPS6	1.30E-30	-0.282421209	1	0.996	2.46E-26
EPHX2	3.15E-23	-0.283848551	0.251	0.465	5.94E-19
RPL34	5.12E-27	-0.283867768	1	0.997	9.67E-23
CHCHD10	1.27E-12	-0.284121177	0.938	0.953	2.41E-08
NEDD9	1.70E-18	-0.285553549	0.327	0.501	3.21E-14
TOMM7	4.62E-20	-0.285603721	0.971	0.964	8.73E-16
CCDC88B	2.08E-16	-0.285617403	0.382	0.518	3.93E-12
NOXO1	2.69E-19	-0.285627709	0.356	0.546	5.08E-15
RAB13	2.24E-15	-0.288753727	0.767	0.807	4.23E-11
CYP3A5	6.11E-12	-0.28989724	0.691	0.727	1.15E-07
CDC42EP5	3.06E-16	-0.292115203	0.818	0.867	5.79E-12
ASAH1	2.53E-16	-0.293723517	0.687	0.731	4.77E-12
AKAP9	5.50E-15	-0.294572592	0.816	0.862	1.04E-10
SNRPN	5.22E-34	-0.295956802	0.013	0.279	9.85E-30
PROX1	5.95E-23	-0.297819547	0.18	0.425	1.12E-18
SEMA3C	2.83E-21	-0.297921008	0.416	0.591	5.34E-17
CLRN3	1.67E-16	-0.299719491	0.464	0.59	3.15E-12
HTATIP2	6.16E-15	-0.303214157	0.771	0.809	1.16E-10
ATF3	8.05E-09	-0.3035046	0.418	0.52	0.000151938
SMDT1	2.27E-19	-0.303596275	0.884	0.899	4.28E-15
BIK	3.62E-18	-0.304133225	0.54	0.682	6.83E-14
PIAS2	6.63E-26	-0.305142467	0.211	0.435	1.25E-21
SOD2	4.13E-11	-0.305430965	0.611	0.65	7.80E-07
ZKSCAN1	1.25E-11	-0.306631565	0.804	0.842	2.36E-07
PDLIM1	7.01E-20	-0.307092869	0.887	0.912	1.32E-15
S100A13	2.08E-19	-0.307600305	0.52	0.64	3.92E-15
TRAPPC1	4.43E-17	-0.308231342	0.776	0.815	8.37E-13
PNRC1	1.47E-10	-0.30980712	0.76	0.787	2.77E-06
LLGL2	1.72E-18	-0.31007774	0.698	0.768	3.25E-14
ID2	1.61E-08	-0.310556721	0.798	0.833	0.000303361
FOS	5.06E-09	-0.312275082	0.936	0.939	9.56E-05
NSD3	6.62E-23	-0.314801052	0.516	0.651	1.25E-18
HNMT	1.39E-16	-0.315812329	0.58	0.677	2.62E-12
ATOH8	4.76E-34	-0.31768924	0.029	0.306	8.99E-30
TSPAN12	7.47E-27	-0.321858757	0.249	0.483	1.41E-22
KLF4	1.89E-15	-0.322377347	0.491	0.636	3.57E-11
ACTN4	7.79E-20	-0.323605467	0.876	0.905	1.47E-15
SULT1B1	1.22E-28	-0.323655519	0.162	0.42	2.30E-24
SLC44A4	9.34E-19	-0.324271203	0.733	0.809	1.76E-14
AHNAK	4.38E-25	-0.326316134	0.329	0.553	8.27E-21
N4BP2L2	4.40E-16	-0.328008448	0.88	0.887	8.30E-12
GALNT6	3.32E-26	-0.328288247	0.282	0.497	6.27E-22
LAMTOR4	5.01E-19	-0.328743669	0.951	0.939	9.46E-15

NIPSNAP2	4.72E-19	-0.329428872	0.682	0.754	8.92E-15
KMT2E	1.08E-19	-0.331148278	0.816	0.855	2.04E-15
ITPR2	6.85E-10	-0.331538033	0.404	0.508	1.29E-05
ADH1C	6.59E-08	-0.33171424	0.347	0.453	0.00124393
NAA38	3.59E-17	-0.333977725	0.782	0.817	6.78E-13
ZNF655	1.01E-39	-0.33534227	0.091	0.403	1.90E-35
ATP8B1	4.71E-21	-0.335397755	0.644	0.726	8.89E-17
ABHD11	1.48E-19	-0.335830907	0.658	0.742	2.79E-15
AC103702.2	2.38E-18	-0.336554945	0.624	0.733	4.49E-14
NOP53	1.76E-15	-0.337898361	0.962	0.963	3.32E-11
FAM84A	6.65E-17	-0.337971271	0.756	0.821	1.26E-12
FARP1	6.26E-42	-0.339228565	0.027	0.346	1.18E-37
SEMA5A	9.34E-18	-0.340994527	0.447	0.585	1.76E-13
LDLRAD4	5.67E-30	-0.342782512	0.131	0.391	1.07E-25
CLDN7	7.50E-22	-0.343218594	0.94	0.952	1.42E-17
IGHA1	5.30E-05	-0.344550558	0.587	0.579	1
TFF1	3.97E-05	-0.345314156	0.542	0.404	0.749327245
DSTN	1.75E-31	-0.347292656	0.989	0.98	3.30E-27
HOXB9	1.48E-19	-0.348393989	0.542	0.657	2.79E-15
IFITM2	6.73E-20	-0.352299204	0.42	0.587	1.27E-15
NQO1	2.37E-13	-0.35517601	0.791	0.837	4.47E-09
S100A6	2.67E-07	-0.355360511	1	0.998	0.005044905
TRIM31	1.35E-27	-0.361408719	0.138	0.41	2.55E-23
MUC3A	5.31E-19	-0.363754336	0.578	0.695	1.00E-14
SRI	1.83E-22	-0.367397512	0.971	0.968	3.46E-18
C19orf33	4.22E-34	-0.367553575	0.627	0.819	7.98E-30
BRI3	3.13E-20	-0.371573765	0.882	0.885	5.91E-16
TSC22D1	3.74E-16	-0.375200435	0.938	0.933	7.07E-12
SLC39A8	1.45E-31	-0.37556891	0.242	0.494	2.73E-27
TBX3	1.15E-13	-0.377503561	0.536	0.629	2.18E-09
CHMP2A	1.46E-27	-0.380652959	0.824	0.868	2.75E-23
TPT1	3.37E-47	-0.385788811	1	0.998	6.36E-43
FOSB	4.04E-13	-0.385997867	0.676	0.759	7.63E-09
SMIM19	7.03E-29	-0.386296755	0.533	0.663	1.33E-24
CD47	2.38E-22	-0.387414395	0.753	0.834	4.50E-18
MUC13	1.59E-14	-0.391760954	0.793	0.81	3.01E-10
SERINC2	6.22E-26	-0.394271829	0.82	0.864	1.17E-21
RUBCNL	8.62E-35	-0.39443855	0.164	0.454	1.63E-30
MT-ND4L	6.87E-18	-0.394975629	0.831	0.862	1.30E-13
CDX2	3.27E-14	-0.395807563	0.824	0.846	6.18E-10
MGLL	4.84E-28	-0.3981506	0.513	0.689	9.14E-24
RNF43	1.05E-20	-0.40055455	0.853	0.872	1.98E-16
ZBTB38	1.77E-21	-0.401139901	0.591	0.689	3.34E-17

VSIG2	5.27E-37	-0.40580331	0.116	0.436	9.95E-33
HLA-C	1.08E-26	-0.406023968	0.993	0.984	2.04E-22
KRTCAP3	2.90E-27	-0.41018513	0.807	0.863	5.47E-23
SOD3	6.14E-14	-0.414775226	0.158	0.314	1.16E-09
PSMB9	1.82E-14	-0.416603817	0.453	0.579	3.44E-10
VAMP2	1.78E-35	-0.418230985	0.387	0.619	3.35E-31
S100A14	1.81E-30	-0.422453381	0.782	0.891	3.41E-26
ATP5F1A	1.57E-26	-0.425412171	0.909	0.923	2.96E-22
BTG2	6.84E-09	-0.427153135	0.773	0.809	0.000129198
SATB2	1.83E-39	-0.42871944	0.216	0.509	3.45E-35
ZNF703	1.22E-24	-0.429901946	0.633	0.757	2.30E-20
RPS4X	1.37E-40	-0.430734367	0.998	0.994	2.59E-36
COMMD6	1.10E-30	-0.431249423	0.973	0.966	2.07E-26
AC020916.1	7.26E-18	-0.431349234	0.349	0.521	1.37E-13
GOLIM4	7.01E-14	-0.432176444	0.884	0.895	1.32E-09
ASS1	2.62E-12	-0.432767026	0.527	0.629	4.95E-08
NFKBIA	1.45E-12	-0.432967937	0.718	0.761	2.73E-08
MT2A	0.029206069	-0.434307311	0.404	0.422	1
H1F0	3.25E-30	-0.435466135	0.476	0.721	6.13E-26
GAS6	2.99E-16	-0.436581424	0.704	0.737	5.65E-12
SARAF	2.30E-34	-0.446032246	0.862	0.898	4.34E-30
HLA-E	4.24E-30	-0.446172526	0.871	0.918	8.01E-26
EPHB3	2.99E-23	-0.449024908	0.413	0.6	5.65E-19
DUOX2	3.25E-23	-0.452002208	0.131	0.353	6.13E-19
SMAD9	5.54E-32	-0.453533144	0.162	0.429	1.05E-27
MT-ND5	2.68E-14	-0.463442276	0.962	0.974	5.07E-10
GSTK1	6.80E-34	-0.46783185	0.92	0.922	1.28E-29
CD99	5.47E-32	-0.475470487	0.889	0.912	1.03E-27
AXIN2	7.49E-21	-0.483775074	0.716	0.77	1.41E-16
ACADVL	2.76E-34	-0.484339373	0.784	0.852	5.22E-30
TMEM230	7.59E-35	-0.484680227	0.756	0.836	1.43E-30
TSTD1	5.07E-39	-0.484689408	0.86	0.91	9.57E-35
MT1E	0.000311257	-0.48729331	0.22	0.278	1
ITM2C	7.69E-16	-0.496669187	0.769	0.771	1.45E-11
RPL36A	3.92E-46	-0.498907817	0.993	0.99	7.41E-42
MUC4	3.59E-48	-0.501269227	0.064	0.422	6.78E-44
MT1X	0.000903507	-0.501441201	0.598	0.609	1
CXCL3	8.04E-05	-0.503676476	0.547	0.607	1
DAB2	1.69E-30	-0.504008873	0.336	0.569	3.19E-26
MUC5B	3.94E-54	-0.507130184	0.033	0.414	7.44E-50
MT-ND1	1.87E-40	-0.512442865	0.978	0.986	3.54E-36
PYCARD	3.90E-35	-0.514313933	0.569	0.741	7.36E-31
RNF186	9.08E-27	-0.520285124	0.447	0.648	1.71E-22

IGKC	4.30E-11	-0.521146598	0.656	0.674	8.12E-07
GPX2	9.14E-34	-0.542649652	0.942	0.968	1.73E-29
TSPAN1	7.78E-19	-0.544774361	0.496	0.643	1.47E-14
HSPA1A	6.50E-32	-0.545793699	0.076	0.353	1.23E-27
TNRC6B	1.41E-47	-0.547826974	0.276	0.603	2.66E-43
RAB32	1.18E-55	-0.54793313	0.167	0.555	2.22E-51
REPIN1	1.90E-27	-0.55538406	0.82	0.827	3.59E-23
RAB11FIP1	4.98E-39	-0.558844681	0.66	0.793	9.41E-35
CCL20	1.48E-07	-0.569835695	0.256	0.368	0.002787418
TCEA3	3.39E-53	-0.574426467	0.169	0.53	6.39E-49
RPS5	1.62E-55	-0.576233643	0.987	0.99	3.07E-51
BACE2	6.16E-48	-0.577530463	0.789	0.879	1.16E-43
APIP	4.37E-31	-0.57974958	0.613	0.709	8.25E-27
TESC	3.92E-55	-0.586019809	0.011	0.399	7.39E-51
CCND2	4.32E-45	-0.586138312	0.347	0.687	8.15E-41
AGR3	1.10E-33	-0.588244433	0.782	0.88	2.08E-29
MUC1	8.75E-48	-0.588719498	0.182	0.531	1.65E-43
RGMB	1.22E-32	-0.591955232	0.267	0.517	2.30E-28
CTSS	5.41E-46	-0.592505062	0.604	0.791	1.02E-41
SLC40A1	9.08E-32	-0.593641785	0.649	0.784	1.71E-27
SLPI	8.49E-36	-0.598780475	0.34	0.599	1.60E-31
CLDN3	5.13E-47	-0.601498869	0.971	0.978	9.69E-43
ASCL2	8.78E-16	-0.609910224	0.878	0.88	1.66E-11
PDZK1IP1	7.64E-49	-0.6130345	0.576	0.806	1.44E-44
ITGA6	1.15E-40	-0.615558866	0.869	0.926	2.17E-36
RPL17	5.78E-52	-0.632685459	0.971	0.953	1.09E-47
PRR15	3.73E-34	-0.632854745	0.638	0.755	7.04E-30
B2M	1.16E-56	-0.67103588	1	0.998	2.18E-52
QTRT1	6.83E-50	-0.677959982	0.68	0.803	1.29E-45
MT-ND4	2.00E-62	-0.679144725	0.993	0.992	3.78E-58
MT-CO1	6.69E-53	-0.684936432	0.987	0.994	1.26E-48
NKD1	1.79E-25	-0.693575548	0.302	0.535	3.38E-21
TXNIP	7.81E-33	-0.703478159	0.482	0.68	1.48E-28
CD9	3.60E-47	-0.716504554	0.973	0.963	6.80E-43
HLA-B	1.08E-57	-0.727383585	0.978	0.985	2.05E-53
RPL26	8.75E-65	-0.730000693	1	0.995	1.65E-60
MUC12	2.44E-36	-0.731687325	0.453	0.71	4.61E-32
MT-ND3	6.73E-39	-0.732219969	0.984	0.988	1.27E-34
PHGR1	2.35E-35	-0.733326973	0.964	0.98	4.43E-31
CXCL1	9.27E-09	-0.741947651	0.364	0.464	0.000175111
CXCL2	3.39E-09	-0.786456627	0.344	0.452	6.41E-05
MT-CYB	2.59E-53	-0.841880746	0.987	0.987	4.89E-49
PRDX5	2.78E-72	-0.844867693	0.987	0.983	5.26E-68

ID4	4.22E-67	-0.846844424	0.033	0.481	7.96E-63
MT-ND2	3.79E-63	-0.868801029	0.982	0.989	7.15E-59
L1TD1	4.55E-61	-0.883385252	0.058	0.483	8.59E-57
MT-CO2	1.07E-78	-0.928314044	0.989	0.992	2.02E-74
S100P	2.58E-48	-0.961420169	0.291	0.656	4.87E-44
MT-CO3	3.63E-80	-1.010974816	0.989	0.994	6.86E-76
MT-ATP6	7.16E-77	-1.023019805	0.984	0.986	1.35E-72
CD74	3.64E-20	-1.042799797	0.538	0.662	6.88E-16
FERMT1	3.78E-85	-1.103446281	0.516	0.781	7.13E-81
FAM3D	3.60E-84	-1.125019061	0.827	0.924	6.79E-80
C10orf99	7.72E-77	-1.150578386	0.52	0.799	1.46E-72
DPEP1	6.69E-56	-1.2719226	0.32	0.641	1.26E-51
ID3	5.26E-40	-1.320825407	0.711	0.83	9.94E-36
LCN2	4.12E-49	-1.373019816	0.627	0.843	7.77E-45
APCDD1	1.27E-79	-1.452891671	0.009	0.506	2.40E-75
PCCA	8.17E-68	-1.6213849	0.324	0.654	1.54E-63
LEFTY1	2.16E-76	-1.743156288	0.224	0.667	4.08E-72
SELENBP1	6.28E-67	-1.990726576	0.713	0.845	1.19E-62
PIGR	7.68E-24	-2.698378458	0.113	0.33	1.45E-19
REG1A	1.61E-40	-3.280886002	0.051	0.361	3.05E-36
**Cluster 6**					
**Gene symbol**	**p_val**	**avg_log2FC**	**pct.1**	**pct.2**	**p_val_adj**
MMP7	4.16E-223	2.662900975	0.909	0.438	7.86E-219
FABP1	1.86E-185	2.4830445	0.881	0.386	3.51E-181
TFF1	1.80E-129	2.397041523	0.74	0.341	3.39E-125
CKB	1.01E-149	2.269179821	0.978	0.855	1.90E-145
NDRG1	4.00E-247	2.185879169	0.911	0.44	7.56E-243
NUPR1	6.09E-134	2.013719497	0.905	0.521	1.15E-129
PLAC8	4.81E-155	1.976412511	0.752	0.335	9.09E-151
TFF3	1.92E-127	1.951668822	0.999	0.989	3.63E-123
CSTB	3.85E-257	1.933436883	0.997	0.972	7.28E-253
AREG	6.04E-127	1.84461474	0.802	0.43	1.14E-122
CA9	1.64E-288	1.762151767	0.878	0.261	3.10E-284
ERO1A	1.48E-226	1.754153722	0.927	0.574	2.79E-222
LYZ	2.66E-139	1.704151211	0.959	0.774	5.02E-135
FXYD3	5.56E-150	1.679706358	0.995	0.971	1.05E-145
TNFRSF12A	2.20E-246	1.670010234	0.947	0.499	4.15E-242
SCD	6.34E-177	1.581550376	0.9	0.465	1.20E-172
TM4SF1	1.50E-123	1.542440431	0.882	0.642	2.84E-119
C8orf33	1.46E-154	1.530377795	0.881	0.561	2.75E-150
SERPINA1	2.26E-35	1.525244264	0.511	0.306	4.26E-31
LDHA	6.62E-189	1.506648765	0.996	0.965	1.25E-184
KRT20	1.04E-176	1.479998976	0.877	0.452	1.96E-172

MALL	3.59E-266	1.425509435	0.846	0.227	6.79E-262
PGK1	1.38E-197	1.418185001	0.989	0.871	2.60E-193
HSPB1	3.04E-101	1.415079268	0.938	0.768	5.75E-97
ANPEP	6.23E-133	1.356644436	0.6	0.167	1.18E-128
KRT19	4.66E-161	1.349101347	0.993	0.957	8.80E-157
FHL2	1.24E-150	1.31757951	0.954	0.807	2.35E-146
FAM13A	2.50E-141	1.307263867	0.734	0.334	4.71E-137
BST2	7.18E-195	1.304130658	0.755	0.211	1.36E-190
C4orf3	3.51E-206	1.296269763	0.982	0.905	6.64E-202
IFI6	2.63E-39	1.210987911	0.595	0.395	4.96E-35
LGALS2	1.44E-147	1.190240595	0.654	0.207	2.72E-143
ISG15	4.47E-96	1.190055715	0.743	0.419	8.44E-92
S100A11	3.49E-197	1.166538243	0.996	0.987	6.58E-193
TACSTD2	8.04E-130	1.159244657	0.527	0.14	1.52E-125
MT1X	4.36E-22	1.154192091	0.665	0.594	8.23E-18
PLAUR	3.34E-93	1.149983661	0.611	0.273	6.31E-89
LGALS3	2.88E-79	1.14428913	0.993	0.989	5.43E-75
NEAT1	1.74E-75	1.129154296	0.943	0.958	3.29E-71
SPINK1	2.05E-65	1.125089187	0.984	0.956	3.87E-61
C12orf75	5.04E-143	1.122707414	0.921	0.693	9.52E-139
SLC11A2	1.46E-115	1.122445346	0.751	0.419	2.75E-111
SLC16A3	2.12E-188	1.11354345	0.794	0.315	4.00E-184
S100A10	2.55E-184	1.091809972	1	0.994	4.81E-180
DDIT4	3.27E-38	1.076225809	0.699	0.534	6.17E-34
ANKRD37	9.57E-71	1.062600546	0.425	0.153	1.81E-66
SQLE	2.39E-92	1.062479854	0.772	0.455	4.52E-88
MRLN	5.49E-205	1.035843117	0.595	0.109	1.04E-200
AC020656.1	3.37E-72	1.028748202	0.57	0.255	6.36E-68
CFD	2.22E-134	1.027034689	0.74	0.327	4.19E-130
IL32	1.17E-115	1.021920592	0.974	0.83	2.22E-111
PHLDA2	4.47E-110	1.014305264	0.881	0.595	8.44E-106
FXYD5	6.95E-210	1.002813086	0.932	0.448	1.31E-205
ATP1B1	1.83E-77	0.98045481	0.995	0.978	3.46E-73
TMSB10	1.61E-190	0.973796505	1	0.998	3.04E-186
MAL2	1.94E-146	0.973790887	0.95	0.808	3.66E-142
IFI27	5.33E-58	0.953709439	0.965	0.981	1.01E-53
ADM	1.87E-81	0.947215721	0.504	0.192	3.54E-77
SLC2A1	3.69E-186	0.939700158	0.661	0.187	6.97E-182
CCND1	1.84E-99	0.928025619	0.892	0.701	3.47E-95
MIF	3.28E-169	0.927652255	0.991	0.972	6.19E-165
CEACAM6	2.55E-30	0.926087118	0.48	0.28	4.82E-26
GPRC5A	1.96E-87	0.924648071	0.888	0.737	3.71E-83
CYSTM1	6.43E-131	0.922831349	0.985	0.934	1.21E-126

FBXO2	4.96E-190	0.918117626	0.664	0.156	9.37E-186
EZR	9.82E-113	0.917437308	0.917	0.722	1.85E-108
PRAP1	2.78E-121	0.91560963	0.832	0.419	5.25E-117
SLC6A8	4.06E-127	0.910556502	0.58	0.187	7.66E-123
SAT1	1.86E-86	0.905835564	0.984	0.974	3.52E-82
APLP2	1.49E-146	0.902605869	0.942	0.746	2.82E-142
PGAM1	1.05E-134	0.896552154	0.95	0.803	1.98E-130
TRAM1	4.83E-140	0.89408477	0.913	0.693	9.12E-136
EIF2S2	1.34E-131	0.883887731	0.961	0.882	2.52E-127
CST3	8.44E-75	0.877065603	0.993	0.975	1.59E-70
ACTB	1.45E-124	0.874804288	1	0.994	2.74E-120
FDPS	3.45E-50	0.872724247	0.859	0.762	6.51E-46
YWHAZ	3.86E-159	0.870490731	0.989	0.96	7.28E-155
CD55	9.47E-42	0.868129815	0.676	0.488	1.79E-37
MTHFD2	7.70E-88	0.865988032	0.744	0.454	1.45E-83
CIB1	7.94E-130	0.865684071	0.962	0.859	1.50E-125
TIMP1	8.75E-105	0.860917904	0.923	0.734	1.65E-100
GCNT3	3.47E-170	0.859357514	0.569	0.127	6.54E-166
C4orf48	2.23E-132	0.85768149	0.962	0.801	4.21E-128
ZNF706	1.12E-152	0.855548158	0.972	0.913	2.12E-148
EIF4A2	2.56E-72	0.848195746	0.965	0.963	4.83E-68
EMP1	7.74E-122	0.84182292	0.562	0.17	1.46E-117
EREG	7.53E-106	0.841308632	0.423	0.099	1.42E-101
PRSS8	5.62E-116	0.837243096	0.855	0.609	1.06E-111
PRSS22	5.38E-139	0.832142693	0.652	0.225	1.02E-134
HSD17B2	5.27E-122	0.821870512	0.405	0.078	9.96E-118
PKM	7.93E-116	0.819158599	0.985	0.95	1.50E-111
TSPAN1	5.03E-31	0.811428299	0.745	0.595	9.50E-27
VEGFA	8.21E-117	0.807129343	0.793	0.425	1.55E-112
CEBPB	2.82E-72	0.801036388	0.881	0.734	5.33E-68
CLTB	8.02E-87	0.797834485	0.931	0.88	1.51E-82
EGLN3	1.88E-145	0.796259029	0.73	0.306	3.55E-141
TGFBI	8.52E-51	0.7948547	0.812	0.609	1.61E-46
P4HA1	8.36E-157	0.794067936	0.726	0.285	1.58E-152
TPI1	1.32E-102	0.7938379	0.984	0.988	2.49E-98
PDXK	5.39E-105	0.787232032	0.882	0.688	1.02E-100
CTSD	9.69E-58	0.786226464	0.95	0.867	1.83E-53
AGR2	7.40E-74	0.784568907	0.989	0.98	1.40E-69
RHOC	6.07E-119	0.784430171	0.969	0.885	1.15E-114
RAB5IF	1.30E-97	0.783386959	0.931	0.808	2.45E-93
CEACAM1	9.72E-71	0.783142382	0.612	0.313	1.84E-66
PMEPA1	1.74E-56	0.781308028	0.828	0.636	3.29E-52
PRSS3	1.41E-95	0.777528648	0.927	0.837	2.66E-91

KRT8	7.90E-96	0.772082584	0.997	0.996	1.49E-91
SH3BGRL3	7.78E-67	0.770724457	0.959	0.902	1.47E-62
CAPG	1.05E-115	0.770440359	0.912	0.661	1.98E-111
CNIH4	1.16E-125	0.761547615	0.9	0.687	2.19E-121
USP53	3.40E-69	0.759124174	0.724	0.468	6.42E-65
ERRFI1	1.50E-57	0.757672644	0.591	0.316	2.83E-53
GDF15	1.12E-10	0.751670669	0.776	0.703	2.12E-06
ANXA2	2.99E-91	0.750216688	0.993	0.98	5.65E-87
F3	5.07E-57	0.744190837	0.561	0.296	9.57E-53
CAMK2N1	1.33E-85	0.743214755	0.946	0.828	2.52E-81
HSPA5	1.64E-51	0.73658152	0.924	0.828	3.10E-47
MISP	1.73E-38	0.735347303	0.86	0.812	3.26E-34
ZFAS1	5.16E-67	0.733783021	0.988	0.977	9.74E-63
TMEM45B	3.26E-93	0.733601136	0.869	0.692	6.16E-89
ENO1	1.39E-79	0.729572418	0.989	0.957	2.62E-75
TMEM123	3.20E-97	0.718912048	0.978	0.95	6.04E-93
CEBPD	3.52E-59	0.717506317	0.862	0.747	6.64E-55
MSMO1	4.76E-73	0.717362515	0.691	0.396	8.99E-69
IDH2	3.79E-71	0.716600021	0.919	0.856	7.15E-67
MUC17	1.15E-167	0.716082859	0.528	0.104	2.17E-163
JPT1	5.39E-88	0.708886772	0.928	0.794	1.02E-83
VDAC2	1.22E-76	0.708659905	0.959	0.948	2.31E-72
CTGF	4.30E-71	0.706334311	0.425	0.146	8.12E-67
COTL1	6.38E-89	0.706121782	0.813	0.534	1.20E-84
TMBIM1	1.16E-86	0.704076314	0.79	0.533	2.19E-82
CHMP4B	2.35E-117	0.696957439	0.976	0.915	4.43E-113
GAPDH	1.02E-94	0.688889919	1	0.997	1.93E-90
CYP2S1	1.51E-121	0.687921227	0.654	0.262	2.85E-117
TSPAN3	7.73E-110	0.68686344	0.93	0.797	1.46E-105
ROMO1	1.43E-98	0.686187606	0.967	0.918	2.71E-94
LINC01133	1.29E-107	0.681941423	0.648	0.28	2.44E-103
EBP	5.10E-50	0.680291109	0.828	0.693	9.62E-46
ANXA4	6.53E-89	0.679700621	0.939	0.871	1.23E-84
KLF6	1.31E-43	0.679410107	0.908	0.837	2.47E-39
EIF1	6.38E-68	0.676739595	0.999	0.993	1.21E-63
C17orf78	2.45E-160	0.676296945	0.328	0.023	4.63E-156
ASPH	1.40E-105	0.675857362	0.894	0.749	2.64E-101
CES2	5.65E-58	0.673935124	0.691	0.444	1.07E-53
SORL1	4.55E-48	0.673900798	0.738	0.572	8.60E-44
SFN	8.36E-59	0.668273592	0.837	0.639	1.58E-54
AKAP7	2.68E-103	0.666816812	0.542	0.191	5.06E-99
SLC39A4	1.55E-88	0.666741283	0.881	0.704	2.93E-84
AZGP1	5.18E-112	0.665827739	0.654	0.244	9.78E-108

INSIG2	1.06E-97	0.664996993	0.737	0.398	2.00E-93
FLNB	1.84E-83	0.663378877	0.779	0.511	3.47E-79
CEACAM5	4.44E-40	0.66213657	0.921	0.882	8.38E-36
VDAC1	2.31E-104	0.656103309	0.989	0.964	4.36E-100
PTTG1IP	3.59E-80	0.65137582	0.886	0.717	6.79E-76
TFRC	3.62E-84	0.646008834	0.831	0.612	6.84E-80
SDCBP2	3.06E-110	0.64298138	0.61	0.217	5.78E-106
MYL12B	3.28E-100	0.641033452	0.993	0.981	6.20E-96
TPD52	1.48E-96	0.639252882	0.965	0.923	2.80E-92
PPDPF	1.84E-91	0.637939475	0.997	0.991	3.47E-87
RPN2	1.55E-86	0.635111775	0.94	0.84	2.92E-82
INSIG1	2.18E-41	0.633249448	0.626	0.414	4.11E-37
MYL9	9.89E-101	0.632918624	0.388	0.082	1.87E-96
AL121761.2	6.43E-135	0.631004181	0.489	0.104	1.21E-130
NPC2	9.89E-95	0.62889862	0.972	0.926	1.87E-90
MYL6	1.39E-111	0.625511066	0.993	0.993	2.63E-107
GNAS	4.80E-97	0.624413028	0.982	0.932	9.07E-93
ANXA3	7.13E-68	0.62315212	0.904	0.764	1.35E-63
SLC3A2	3.38E-35	0.622405378	0.771	0.667	6.39E-31
ALDOA	3.26E-103	0.622369675	0.814	0.554	6.16E-99
DMBT1	5.26E-10	0.621754707	0.267	0.177	9.94E-06
CEBPG	1.99E-85	0.616284872	0.831	0.595	3.76E-81
H2AFZ	7.51E-55	0.614887677	0.973	0.926	1.42E-50
AL133453.1	1.53E-100	0.613896378	0.595	0.227	2.88E-96
CFDP1	2.86E-79	0.611940585	0.888	0.751	5.40E-75
TRIB1	9.17E-46	0.611188095	0.743	0.55	1.73E-41
LIMA1	2.15E-80	0.610170224	0.944	0.875	4.06E-76
WDR1	6.35E-75	0.601424497	0.893	0.781	1.20E-70
ANGPTL4	1.32E-160	0.59966166	0.35	0.029	2.49E-156
IDI1	2.20E-37	0.599159746	0.682	0.507	4.16E-33
GSTP1	2.83E-87	0.594954789	0.993	0.991	5.34E-83
LIPH	3.99E-68	0.594821736	0.707	0.434	7.54E-64
ARPC2	9.35E-91	0.594105045	0.981	0.952	1.77E-86
SPATS2L	1.64E-76	0.593644669	0.855	0.652	3.09E-72
KRT18	3.55E-52	0.591821845	0.999	0.994	6.70E-48
DDIT3	7.13E-42	0.5896459	0.533	0.307	1.35E-37
SDCBP	4.85E-73	0.589639366	0.921	0.836	9.15E-69
HSP90B1	1.17E-63	0.589539262	0.976	0.948	2.20E-59
TMEM92	2.20E-135	0.5894316	0.533	0.141	4.16E-131
UBE2B	8.80E-72	0.586545637	0.902	0.785	1.66E-67
SPINT1	1.83E-57	0.585245286	0.901	0.824	3.45E-53
S100A6	9.84E-47	0.584515725	1	0.998	1.86E-42
SEC61G	2.98E-89	0.584127528	0.973	0.959	5.63E-85

RND3	2.08E-66	0.582692395	0.63	0.333	3.94E-62
ATF4	1.69E-49	0.581991757	0.893	0.826	3.19E-45
SMIM24	1.72E-86	0.581318046	0.561	0.202	3.24E-82
TOP1	2.63E-75	0.580085881	0.883	0.696	4.97E-71
PLIN2	1.93E-45	0.580068925	0.714	0.504	3.64E-41
SPINT2	2.92E-99	0.579615333	0.989	0.984	5.52E-95
DYNLRB1	1.11E-82	0.579045309	0.94	0.883	2.10E-78
P4HB	2.19E-59	0.578385005	0.974	0.948	4.13E-55
MYL12A	1.24E-95	0.575826253	0.991	0.979	2.35E-91
TMPRSS2	8.26E-56	0.575103221	0.78	0.593	1.56E-51
YWHAB	1.92E-69	0.57251364	0.97	0.953	3.62E-65
CDK2AP2	1.94E-59	0.570209084	0.79	0.579	3.66E-55
PSAT1	3.71E-114	0.569982325	0.573	0.184	7.01E-110
GLO1	3.69E-59	0.568609877	0.874	0.784	6.97E-55
PFKFB3	1.82E-97	0.567661814	0.447	0.126	3.44E-93
RHOF	6.85E-80	0.566797003	0.405	0.125	1.29E-75
TMCC1	2.61E-118	0.566648272	0.61	0.219	4.93E-114
PAM	1.77E-202	0.564689136	0.627	0.127	3.34E-198
PSMA7	5.43E-75	0.564005596	0.986	0.974	1.02E-70
TMED9	1.07E-71	0.563539566	0.936	0.88	2.02E-67
OAZ1	3.61E-112	0.56195563	0.989	0.987	6.82E-108
SMIM31	6.27E-77	0.559383551	0.706	0.385	1.18E-72
SERPINB6	1.35E-56	0.558681522	0.951	0.941	2.55E-52
SELENOS	8.54E-58	0.558219247	0.905	0.812	1.61E-53
AP3S1	5.71E-86	0.557470064	0.911	0.749	1.08E-81
ACAT2	3.63E-31	0.555899322	0.649	0.486	6.85E-27
PLEC	5.77E-50	0.553801884	0.682	0.443	1.09E-45
TMBIM6	4.02E-93	0.551039719	0.985	0.971	7.59E-89
CDH17	2.24E-50	0.550771904	0.92	0.884	4.23E-46
MAP1LC3B	1.11E-69	0.547624868	0.871	0.706	2.10E-65
ELOC	1.16E-88	0.54749022	0.942	0.854	2.19E-84
MARCKS	1.43E-53	0.546899087	0.916	0.851	2.70E-49
PRR13	1.52E-76	0.546073109	0.969	0.926	2.87E-72
BSG	2.30E-78	0.54432832	0.972	0.956	4.34E-74
PPARG	6.26E-62	0.543653882	0.771	0.561	1.18E-57
LSR	3.20E-79	0.54341952	0.94	0.883	6.05E-75
TRIB3	5.09E-74	0.543153914	0.369	0.108	9.61E-70
HSPA1B	2.56E-35	0.541871539	0.762	0.567	4.84E-31
PFKP	1.01E-89	0.541217367	0.706	0.386	1.90E-85
EPCAM	2.85E-77	0.541140019	0.999	0.994	5.37E-73
GSN	9.02E-35	0.541035775	0.736	0.569	1.70E-30
JUND	3.58E-57	0.540412496	0.86	0.734	6.76E-53
BLCAP	2.52E-77	0.538563488	0.749	0.471	4.76E-73

SLC1A5	3.18E-72	0.538204481	0.714	0.422	6.01E-68
CFL1	8.38E-89	0.537692939	0.999	0.993	1.58E-84
GSTM3	2.73E-95	0.536514849	0.659	0.279	5.15E-91
ACTG1	7.29E-42	0.535992247	1	0.995	1.38E-37
MX1	4.78E-78	0.533048839	0.424	0.134	9.03E-74
HKDC1	8.86E-147	0.530236893	0.572	0.149	1.67E-142
HBEGF	2.70E-35	0.528576903	0.458	0.245	5.10E-31
C19orf33	4.76E-07	0.525764649	0.816	0.79	0.008986982
LRRFIP1	2.99E-56	0.524901826	0.886	0.769	5.64E-52
TMC5	1.39E-28	0.5212087	0.822	0.759	2.63E-24
CDH1	1.46E-57	0.520772506	0.904	0.814	2.75E-53
LDLR	5.56E-64	0.520449396	0.653	0.365	1.05E-59
BCL2L1	7.65E-69	0.519626849	0.771	0.521	1.44E-64
ANXA13	5.22E-87	0.514775985	0.622	0.245	9.85E-83
TNFRSF21	1.00E-68	0.513365499	0.622	0.327	1.89E-64
SH3GLB1	1.23E-61	0.513205443	0.889	0.752	2.32E-57
LMO7	2.88E-24	0.512375538	0.786	0.673	5.43E-20
PRELID3B	1.03E-57	0.510728489	0.863	0.749	1.95E-53
IFNGR1	4.16E-61	0.509165539	0.763	0.532	7.86E-57
ATP2B1	9.36E-64	0.508608848	0.772	0.547	1.77E-59
HILPDA	3.63E-33	0.506858511	0.615	0.411	6.85E-29
OLFM4	2.09E-19	0.506497008	0.938	0.89	3.95E-15
GPX4	9.23E-51	0.50481716	0.965	0.957	1.74E-46
SERPINE2	3.92E-60	0.504554473	0.34	0.108	7.40E-56
BTG1	8.15E-22	0.504046602	0.911	0.868	1.54E-17
GFPT1	6.01E-54	0.503914644	0.854	0.692	1.13E-49
BHLHE40	1.42E-39	0.502709029	0.518	0.301	2.69E-35
PDIA3	2.21E-49	0.502229885	0.963	0.942	4.16E-45
ANXA5	5.17E-54	0.497441744	0.752	0.507	9.77E-50
FTL	2.52E-78	0.494102684	0.997	0.996	4.77E-74
HK2	3.28E-71	0.492222795	0.531	0.23	6.19E-67
P4HA2	1.33E-80	0.492044135	0.623	0.297	2.50E-76
WDR45B	9.08E-52	0.49126961	0.778	0.6	1.71E-47
TMSB4X	9.07E-54	0.490820898	0.999	0.999	1.71E-49
CXADR	2.27E-35	0.490796859	0.93	0.895	4.29E-31
WEE1	1.73E-52	0.490422426	0.653	0.389	3.27E-48
STMN1	4.26E-28	0.489238408	0.752	0.558	8.05E-24
GCHFR	1.36E-53	0.488520296	0.715	0.483	2.57E-49
MANF	1.75E-49	0.48794752	0.862	0.688	3.30E-45
MYH9	1.40E-50	0.487202558	0.907	0.844	2.65E-46
ISG20	7.54E-67	0.486025373	0.383	0.125	1.42E-62
HIGD1A	4.39E-39	0.485605022	0.827	0.708	8.29E-35
ZFAND5	3.65E-44	0.484858617	0.835	0.668	6.90E-40

DBI	4.38E-27	0.482128401	0.958	0.959	8.26E-23
GPI	3.72E-47	0.481811208	0.885	0.807	7.02E-43
PLA2G16	2.77E-10	0.481363966	0.55	0.459	5.23E-06
RBM39	3.72E-62	0.480952321	0.966	0.936	7.03E-58
GUK1	3.26E-63	0.47810753	0.954	0.936	6.16E-59
CDC42	3.68E-50	0.477901044	0.974	0.946	6.94E-46
IFITM1	6.79E-05	0.476341336	0.771	0.801	1
DHCR7	2.26E-32	0.474494576	0.569	0.382	4.26E-28
LACTB2	1.36E-65	0.473630706	0.752	0.494	2.58E-61
OCLN	1.66E-41	0.473122377	0.68	0.464	3.14E-37
CDS1	1.43E-83	0.473069165	0.747	0.434	2.69E-79
BNIP3L	9.89E-55	0.473025267	0.759	0.535	1.87E-50
STAU1	3.31E-55	0.47297997	0.889	0.778	6.25E-51
FAM84B	1.37E-82	0.469932072	0.709	0.374	2.59E-78
REEP3	5.10E-71	0.468494979	0.818	0.565	9.64E-67
LAMB3	2.49E-44	0.468346771	0.617	0.373	4.71E-40
CHMP2B	2.32E-61	0.468215205	0.886	0.799	4.38E-57
EDN1	1.00E-18	0.466910406	0.516	0.356	1.89E-14
WSB1	6.20E-26	0.466397653	0.789	0.672	1.17E-21
TTR	2.13E-65	0.465401709	0.312	0.079	4.01E-61
TINAGL1	3.52E-33	0.464693542	0.794	0.658	6.64E-29
RARRES1	4.21E-09	0.463067881	0.407	0.322	7.95E-05
HSP90AA1	8.89E-50	0.462327691	0.988	0.987	1.68E-45
RRM2	7.46E-22	0.461134705	0.291	0.15	1.41E-17
ENY2	3.61E-65	0.460549357	0.931	0.891	6.81E-61
SNX9	3.04E-38	0.459198339	0.645	0.435	5.73E-34
PLS1	5.59E-47	0.459082037	0.794	0.614	1.06E-42
LGALS4	6.71E-22	0.455684005	0.991	0.989	1.27E-17
AZIN1	6.27E-55	0.455371666	0.813	0.6	1.18E-50
PTGR1	9.66E-58	0.454264732	0.757	0.525	1.82E-53
SLC52A2	1.53E-58	0.45393831	0.805	0.633	2.89E-54
DSC2	2.70E-45	0.45382567	0.863	0.734	5.10E-41
MALAT1	4.77E-15	0.452609558	0.993	0.994	9.01E-11
HNF4A	5.34E-32	0.452524359	0.753	0.554	1.01E-27
TENT5A	3.21E-34	0.452262968	0.533	0.318	6.05E-30
MGST3	1.09E-52	0.450207343	0.963	0.945	2.06E-48
PRR15L	4.61E-52	0.448979023	0.808	0.645	8.70E-48
MVD	2.70E-26	0.448126356	0.595	0.437	5.09E-22
EPHA2	9.09E-67	0.446758393	0.489	0.199	1.72E-62
HMGCS1	5.37E-30	0.446378784	0.581	0.4	1.01E-25
CRIP1	2.56E-08	0.445328499	0.355	0.266	0.000483835
TDP2	1.95E-51	0.445057836	0.828	0.669	3.67E-47
LRP10	1.79E-36	0.444838017	0.817	0.686	3.38E-32

NBL1	8.91E-39	0.443777143	0.928	0.908	1.68E-34
ZMYND8	4.58E-53	0.443761388	0.755	0.52	8.64E-49
FAM107B	4.99E-99	0.441527743	0.504	0.157	9.43E-95
CAP1	2.91E-48	0.4413807	0.92	0.833	5.49E-44
ITGB1	6.51E-46	0.44058121	0.916	0.888	1.23E-41
PLBD1	8.43E-37	0.440398848	0.859	0.8	1.59E-32
EEF1D	2.99E-66	0.440332181	0.989	0.988	5.65E-62
AMIGO2	2.48E-176	0.439238152	0.438	0.052	4.68E-172
XBP1	1.95E-38	0.43775642	0.896	0.831	3.68E-34
MDK	5.73E-24	0.437352019	0.851	0.719	1.08E-19
RHPN2	6.76E-69	0.436102835	0.617	0.312	1.28E-64
NECTIN2	4.09E-56	0.435913813	0.768	0.536	7.72E-52
CYTOR	2.58E-90	0.435418306	0.446	0.131	4.88E-86
GNG5	1.75E-64	0.434849756	0.976	0.948	3.30E-60
PIM1	4.78E-82	0.434758976	0.393	0.113	9.03E-78
ALDOC	6.03E-94	0.434052871	0.491	0.158	1.14E-89
TNIP1	9.71E-51	0.430866902	0.785	0.591	1.83E-46
FAM49B	4.62E-63	0.430839422	0.797	0.573	8.73E-59
PRELID1	1.65E-45	0.430271513	0.955	0.952	3.12E-41
SCARB2	1.99E-54	0.429585913	0.854	0.679	3.75E-50
KRT10	1.97E-46	0.428825954	0.874	0.784	3.71E-42
GOLGA4	5.98E-48	0.428685648	0.9	0.822	1.13E-43
KIF5B	2.39E-42	0.427738685	0.957	0.951	4.51E-38
CISD2	6.53E-52	0.427132791	0.862	0.72	1.23E-47
C1orf21	2.15E-74	0.426906476	0.728	0.406	4.05E-70
OCIAD2	3.97E-23	0.426562079	0.959	0.956	7.49E-19
TARS	1.43E-49	0.4262979	0.717	0.492	2.70E-45
DNM2	1.86E-43	0.426275794	0.764	0.603	3.51E-39
SDC4	2.10E-23	0.424684748	0.715	0.563	3.96E-19
HEPH	4.89E-41	0.424666362	0.744	0.554	9.23E-37
MFSD10	2.20E-54	0.424234093	0.855	0.733	4.15E-50
EPS8L1	3.07E-43	0.423597373	0.508	0.276	5.79E-39
RAP1B	1.28E-46	0.423450137	0.87	0.774	2.42E-42
CTSV	1.89E-72	0.421446909	0.488	0.178	3.57E-68
ADIRF	0.192455149	0.420669361	0.238	0.279	1
CLIC3	4.29E-26	0.420363782	0.327	0.164	8.09E-22
SLC35A3	6.80E-44	0.420314658	0.82	0.713	1.28E-39
RBCK1	3.79E-14	0.419533461	0.787	0.764	7.16E-10
TAF7	1.91E-32	0.419358132	0.881	0.836	3.60E-28
RAD21	9.85E-35	0.419341568	0.818	0.686	1.86E-30
REG4	4.06E-15	0.419306779	0.461	0.317	7.66E-11
EIF6	2.31E-48	0.418899335	0.928	0.887	4.36E-44
PCLAF	9.24E-18	0.416923894	0.428	0.278	1.74E-13

IQGAP2	6.81E-63	0.416323825	0.615	0.319	1.29E-58
FAM162A	1.71E-33	0.415980676	0.915	0.884	3.22E-29
KRT80	3.98E-89	0.41527511	0.362	0.085	7.52E-85
ARL6IP1	1.21E-26	0.413236247	0.917	0.874	2.29E-22
ARPC1B	4.27E-35	0.412648714	0.932	0.872	8.07E-31
PDIA6	3.27E-38	0.411523667	0.951	0.945	6.18E-34
VCL	7.44E-39	0.410325939	0.606	0.384	1.41E-34
DYNLT1	2.12E-51	0.409461188	0.936	0.902	4.00E-47
FUT4	2.45E-53	0.408687094	0.579	0.327	4.62E-49
CKS1B	1.57E-20	0.406597801	0.695	0.577	2.97E-16
CCDC107	5.12E-46	0.405788018	0.74	0.549	9.67E-42
HSD17B12	3.54E-28	0.405737359	0.882	0.837	6.67E-24
DSG2	1.08E-39	0.405613679	0.924	0.899	2.04E-35
NEU1	1.19E-29	0.405081764	0.726	0.572	2.24E-25
SCARB1	9.70E-63	0.404073075	0.589	0.299	1.83E-58
AKR1B10	1.73E-61	0.402663784	0.489	0.195	3.26E-57
VPS28	2.57E-49	0.402430832	0.93	0.892	4.85E-45
ST14	1.37E-40	0.401789667	0.943	0.913	2.58E-36
BZW1	2.30E-42	0.400443647	0.911	0.799	4.34E-38
ATP1B3	1.88E-36	0.399986204	0.9	0.849	3.55E-32
KCMF1	5.55E-46	0.397951833	0.717	0.506	1.05E-41
CDKN2A	5.14E-106	0.397363536	0.608	0.202	9.70E-102
MAPRE1	1.59E-51	0.396784488	0.766	0.546	3.01E-47
ATP6V0E1	3.55E-57	0.396053152	0.97	0.952	6.70E-53
PAG1	4.01E-116	0.395268199	0.434	0.096	7.57E-112
DERL1	1.24E-60	0.395198708	0.763	0.504	2.34E-56
SLC7A5	2.00E-44	0.394923032	0.491	0.257	3.78E-40
LMO4	1.13E-60	0.393735857	0.562	0.264	2.14E-56
RNF114	1.39E-50	0.393458741	0.852	0.716	2.62E-46
OPTN	4.24E-29	0.392639411	0.614	0.433	8.00E-25
PRDX2	1.12E-35	0.391273584	0.954	0.961	2.12E-31
UBC	1.04E-26	0.390424414	0.984	0.98	1.96E-22
DNAJB1	2.48E-11	0.389421018	0.799	0.746	4.69E-07
F2RL1	1.63E-49	0.389342023	0.592	0.334	3.07E-45
IGFBP2	7.18E-10	0.388855788	0.888	0.921	1.36E-05
TNFRSF1A	8.29E-43	0.386743186	0.699	0.506	1.57E-38
PLA2G2A	0.43960531	0.386410681	0.477	0.45	1
EGLN1	6.77E-59	0.38489455	0.644	0.367	1.28E-54
CDA	6.49E-75	0.384421198	0.262	0.048	1.23E-70
ME1	1.02E-72	0.38427592	0.551	0.233	1.93E-68
TMPRSS4	1.08E-60	0.383239455	0.661	0.367	2.03E-56
PLEKHA1	5.65E-51	0.383192869	0.753	0.548	1.07E-46
YWHAH	2.01E-37	0.383016432	0.87	0.76	3.79E-33

CD164	2.85E-33	0.382422792	0.967	0.944	5.38E-29
HES4	1.44E-73	0.382391188	0.538	0.219	2.72E-69
CSNK1A1	4.62E-43	0.382310199	0.949	0.919	8.73E-39
ATP6V0B	1.11E-41	0.382196273	0.908	0.849	2.10E-37
ARF4	4.60E-38	0.380680766	0.902	0.821	8.69E-34
CAST	1.59E-28	0.380582772	0.958	0.912	2.99E-24
SINHCAF	1.17E-37	0.380500355	0.816	0.694	2.21E-33
PSAP	6.94E-29	0.380215505	0.962	0.932	1.31E-24
CYP2W1	6.24E-83	0.379517488	0.55	0.187	1.18E-78
PTP4A1	1.90E-38	0.379257632	0.79	0.617	3.59E-34
ARF6	9.29E-32	0.379222136	0.852	0.776	1.75E-27
RAB22A	3.49E-42	0.378190397	0.703	0.49	6.60E-38
PRELID2	1.45E-42	0.37618284	0.684	0.473	2.74E-38
DYNLL1	5.49E-39	0.375205464	0.984	0.976	1.04E-34
MPZL2	1.01E-47	0.374921015	0.672	0.436	1.91E-43
EPAS1	2.53E-69	0.374137197	0.527	0.228	4.77E-65
PLSCR1	1.87E-28	0.373856445	0.797	0.674	3.54E-24
SOD1	2.60E-40	0.372972656	0.984	0.98	4.91E-36
LRPAP1	1.27E-34	0.372874013	0.828	0.735	2.40E-30
GARS	3.04E-33	0.372852679	0.68	0.51	5.73E-29
SLC9A3R1	2.55E-52	0.372642759	0.576	0.32	4.81E-48
TXNDC17	2.62E-17	0.372319402	0.912	0.894	4.95E-13
SPTBN1	2.15E-30	0.372291888	0.871	0.8	4.06E-26
FAM213B	9.14E-57	0.372220817	0.612	0.336	1.73E-52
DNAJB9	1.22E-25	0.371799026	0.533	0.358	2.31E-21
RNASET2	7.61E-32	0.371119153	0.919	0.881	1.44E-27
RAB11A	3.88E-41	0.370784696	0.94	0.919	7.32E-37
CD63	8.24E-43	0.370728967	0.992	0.984	1.56E-38
ACSL3	3.83E-37	0.370481045	0.678	0.472	7.23E-33
CPS1	2.90E-57	0.368526443	0.446	0.163	5.48E-53
ATP5F1E	8.53E-53	0.367205937	0.992	0.992	1.61E-48
SUB1	2.01E-47	0.366227536	0.981	0.972	3.79E-43
ADRM1	8.54E-33	0.366084658	0.881	0.786	1.61E-28
BMP4	1.20E-11	0.365602411	0.561	0.431	2.26E-07
MYO6	1.72E-39	0.365222316	0.878	0.743	3.25E-35
CLIC1	3.13E-48	0.364731637	0.993	0.988	5.91E-44
RIOK3	4.06E-43	0.362850615	0.679	0.447	7.67E-39
ABRACL	1.78E-39	0.362655752	0.909	0.837	3.36E-35
DNAJB11	8.32E-40	0.362286243	0.762	0.554	1.57E-35
HMGCR	6.25E-28	0.362168531	0.553	0.371	1.18E-23
CD2AP	3.31E-34	0.361947765	0.808	0.685	6.25E-30
BCAS1	6.12E-41	0.361696034	0.547	0.311	1.16E-36
HERPUD1	1.05E-18	0.359464665	0.759	0.66	1.98E-14

GID8	3.12E-41	0.359295107	0.799	0.637	5.88E-37
PHF20L1	1.09E-43	0.358502507	0.755	0.564	2.05E-39
MTMR11	5.99E-55	0.358341357	0.588	0.306	1.13E-50
FDX1	1.25E-31	0.358197013	0.801	0.675	2.36E-27
TPM3	3.23E-42	0.358067385	0.942	0.859	6.09E-38
ATP5MD	4.21E-45	0.357865486	0.991	0.981	7.94E-41
TUBB2A	7.95E-64	0.356511793	0.421	0.154	1.50E-59
SEMA4B	1.37E-59	0.35647267	0.523	0.242	2.59E-55
IFNGR2	1.50E-24	0.356465817	0.77	0.636	2.83E-20
HSPD1	1.95E-19	0.356290713	0.946	0.967	3.68E-15
GABRA2	9.04E-88	0.355976791	0.381	0.093	1.71E-83
PPP2R2A	4.14E-30	0.354846822	0.695	0.508	7.82E-26
ANXA1	4.69E-19	0.354808629	0.278	0.148	8.86E-15
SURF4	3.99E-50	0.354776598	0.786	0.572	7.53E-46
SLK	2.43E-32	0.354141287	0.721	0.553	4.58E-28
SLC20A1	1.98E-50	0.354051557	0.501	0.242	3.73E-46
UBE2V2	7.34E-38	0.353677132	0.841	0.727	1.39E-33
MATN2	2.89E-65	0.353388875	0.505	0.207	5.45E-61
MCL1	4.18E-29	0.352490978	0.81	0.685	7.90E-25
STK17A	4.40E-32	0.352443245	0.449	0.248	8.31E-28
SCAND1	2.42E-37	0.352083162	0.916	0.874	4.57E-33
MKNK2	1.25E-20	0.351131798	0.684	0.557	2.36E-16
CTSH	1.20E-21	0.350815299	0.78	0.676	2.26E-17
NORAD	2.37E-30	0.349900383	0.859	0.789	4.47E-26
EIF4G2	1.73E-39	0.348766235	0.969	0.94	3.26E-35
AGO2	4.04E-52	0.348619431	0.73	0.464	7.62E-48
MTDH	1.79E-33	0.348158028	0.932	0.926	3.39E-29
TPD52L2	8.15E-47	0.347925903	0.667	0.426	1.54E-42
TFG	2.96E-37	0.346994112	0.832	0.686	5.59E-33
PKP3	1.45E-34	0.346880389	0.835	0.738	2.74E-30
ACE2	1.92E-92	0.344762384	0.473	0.143	3.62E-88
LAMC2	1.58E-24	0.342415599	0.451	0.266	2.98E-20
FUOM	2.65E-57	0.341896153	0.626	0.33	5.00E-53
TPM4	8.11E-25	0.340934218	0.893	0.844	1.53E-20
CD59	9.24E-15	0.340223153	0.846	0.817	1.75E-10
ACSS2	1.61E-44	0.340213049	0.537	0.297	3.03E-40
DIAPH1	1.04E-49	0.339610812	0.664	0.401	1.97E-45
PPP2R1A	2.15E-37	0.33861524	0.894	0.832	4.06E-33
RRAS	2.05E-46	0.338430833	0.599	0.351	3.88E-42
PFDN2	3.57E-27	0.33758614	0.878	0.822	6.75E-23
ARF1	4.17E-38	0.336748107	0.942	0.913	7.86E-34
C3orf85	2.01E-59	0.335883725	0.438	0.172	3.79E-55
FKBP2	5.88E-28	0.335301985	0.938	0.903	1.11E-23

COL17A1	2.68E-20	0.335122413	0.316	0.179	5.05E-16
UBL5	1.89E-45	0.335051748	0.974	0.974	3.56E-41
OAS1	2.58E-22	0.335043525	0.463	0.305	4.87E-18
CAPZB	1.44E-34	0.334585979	0.925	0.88	2.72E-30
MYBL2	5.50E-45	0.334373042	0.333	0.125	1.04E-40
AARS	2.95E-40	0.334039618	0.526	0.291	5.57E-36
B4GALT1	4.43E-22	0.334028562	0.596	0.417	8.36E-18
AGPAT2	5.38E-28	0.333695123	0.859	0.772	1.01E-23
CALM2	2.27E-35	0.333461203	0.985	0.981	4.28E-31
CD81	5.20E-24	0.333148793	0.785	0.699	9.82E-20
STC2	1.08E-115	0.333042821	0.293	0.034	2.04E-111
CHPF	2.42E-43	0.332947012	0.614	0.371	4.57E-39
OSTF1	1.50E-39	0.332803063	0.768	0.581	2.84E-35
MIR210HG	8.51E-96	0.331683604	0.431	0.115	1.61E-91
RAB6A	6.38E-35	0.331441064	0.776	0.6	1.20E-30
RHOB	1.39E-16	0.331406531	0.85	0.782	2.62E-12
BCL2L15	6.64E-32	0.331174633	0.722	0.554	1.25E-27
IDH1	1.05E-25	0.330972308	0.825	0.713	1.98E-21
ACTR3	7.45E-31	0.330499345	0.871	0.816	1.41E-26
ANXA11	1.28E-19	0.330114482	0.919	0.881	2.41E-15
DHCR24	6.83E-34	0.330003746	0.577	0.369	1.29E-29
FNDC3B	1.76E-32	0.329957119	0.638	0.431	3.32E-28
F11R	1.03E-29	0.329737885	0.706	0.533	1.95E-25
CTNNA1	1.53E-31	0.329466998	0.885	0.803	2.88E-27
HELZ2	1.49E-58	0.328711858	0.501	0.221	2.80E-54
DPP4	5.40E-90	0.32864694	0.44	0.126	1.02E-85
ARPC3	1.27E-40	0.328200851	0.982	0.974	2.40E-36
TMEM141	8.25E-25	0.327771658	0.943	0.949	1.56E-20
SEPHS2	2.20E-22	0.327623673	0.813	0.743	4.16E-18
TUBA1C	1.16E-23	0.327246186	0.84	0.745	2.19E-19
PUF60	6.03E-33	0.327180303	0.831	0.712	1.14E-28
MKI67	3.77E-21	0.327103914	0.314	0.165	7.12E-17
MMP24OS	1.62E-26	0.326776874	0.787	0.668	3.06E-22
PLCB3	7.57E-48	0.326677296	0.686	0.435	1.43E-43
CLDN4	1.35E-13	0.325911996	0.98	0.968	2.54E-09
ATP6V1G1	2.72E-31	0.325692833	0.961	0.936	5.14E-27
TMEM176A	9.90E-19	0.325347225	0.714	0.574	1.87E-14
RTN4	1.22E-30	0.325121597	0.958	0.923	2.29E-26
MXI1	5.49E-38	0.324310139	0.53	0.3	1.04E-33
UGCG	1.20E-41	0.324295134	0.528	0.289	2.27E-37
AGRN	5.16E-43	0.323606847	0.528	0.286	9.73E-39
OSER1	6.38E-41	0.323548619	0.621	0.391	1.20E-36
UGP2	2.86E-29	0.323330632	0.86	0.794	5.40E-25

SH3KBP1	2.85E-29	0.322703588	0.736	0.583	5.38E-25
G3BP2	1.19E-37	0.322690541	0.821	0.684	2.25E-33
DNAJC1	2.17E-36	0.32226511	0.74	0.569	4.09E-32
CHMP4C	1.09E-27	0.322215372	0.787	0.658	2.06E-23
ARFGAP3	1.17E-39	0.321510022	0.533	0.3	2.20E-35
MXD1	3.19E-24	0.320157419	0.423	0.252	6.02E-20
GNG12	3.38E-40	0.319400215	0.757	0.549	6.38E-36
B4GALT4	1.44E-31	0.319063454	0.539	0.331	2.73E-27
TMEM189	4.38E-46	0.31859925	0.583	0.331	8.27E-42
BCL10	9.63E-22	0.318554425	0.64	0.473	1.82E-17
MAPK6	4.96E-37	0.318389348	0.629	0.404	9.37E-33
ATP2A2	4.07E-37	0.317704161	0.766	0.582	7.69E-33
RAP2B	2.51E-44	0.317686338	0.439	0.204	4.73E-40
ENO2	1.19E-120	0.316779104	0.34	0.048	2.25E-116
ANKRD11	3.36E-26	0.316464	0.802	0.689	6.34E-22
ASNS	8.50E-36	0.315722295	0.405	0.204	1.60E-31
RAB2A	2.69E-42	0.315596462	0.943	0.915	5.09E-38
RIN2	1.04E-23	0.315512474	0.474	0.29	1.97E-19
DYNC1I2	7.30E-26	0.315087056	0.925	0.841	1.38E-21
PGRMC1	3.96E-32	0.314850821	0.867	0.794	7.49E-28
SULT2B1	1.45E-40	0.313574228	0.484	0.245	2.74E-36
BAMBI	2.36E-39	0.313227438	0.423	0.198	4.46E-35
VAPA	2.05E-23	0.312959996	0.925	0.9	3.86E-19
RBP2	7.03E-44	0.312943292	0.316	0.109	1.33E-39
SERPINH1	5.64E-33	0.312554202	0.644	0.452	1.06E-28
EPB41L2	4.60E-22	0.31178994	0.805	0.684	8.68E-18
FTH1	1.85E-25	0.311511214	0.999	0.998	3.50E-21
RHEB	2.12E-28	0.311500346	0.93	0.883	4.00E-24
GALNT1	6.09E-16	0.311106369	0.768	0.736	1.15E-11
FAT1	6.01E-27	0.310351885	0.757	0.611	1.13E-22
POMP	4.54E-27	0.310155	0.982	0.974	8.57E-23
S100A16	1.75E-09	0.309824778	0.813	0.786	3.31E-05
SLC22A18	1.61E-22	0.309507679	0.671	0.519	3.04E-18
GNB1	2.52E-31	0.309296296	0.772	0.656	4.77E-27
HMOX1	1.15E-61	0.308729727	0.428	0.159	2.16E-57
CDH3	7.12E-100	0.308704873	0.518	0.16	1.34E-95
HM13	4.76E-26	0.308146654	0.797	0.697	8.98E-22
PRDX6	8.66E-05	0.307761894	0.864	0.868	1
ITGB4	6.80E-23	0.30764816	0.721	0.585	1.28E-18
TCEAL9	3.29E-27	0.307270028	0.669	0.447	6.22E-23
DUSP6	3.92E-29	0.306878842	0.524	0.327	7.41E-25
SLC16A1	6.83E-66	0.306691304	0.511	0.212	1.29E-61
MLX	2.93E-34	0.306588867	0.668	0.486	5.53E-30

GADD45B	4.51E-36	0.305711659	0.377	0.175	8.51E-32
INAVA	1.07E-45	0.305603038	0.606	0.343	2.02E-41
CANX	3.16E-24	0.305551155	0.951	0.934	5.97E-20
SYBU	1.29E-38	0.305507214	0.602	0.366	2.43E-34
GATM	1.64E-101	0.30547287	0.438	0.109	3.10E-97
CYBRD1	8.93E-98	0.305245848	0.402	0.095	1.69E-93
LAPTM4B	2.15E-24	0.304968448	0.759	0.661	4.07E-20
COX17	3.72E-23	0.304764416	0.85	0.747	7.02E-19
FILIP1L	4.84E-62	0.303927342	0.32	0.092	9.14E-58
RAD23B	4.78E-29	0.302436177	0.844	0.753	9.03E-25
ARID3A	7.21E-28	0.301688453	0.575	0.374	1.36E-23
CDCA3	5.49E-75	0.301402159	0.402	0.118	1.04E-70
DST	2.42E-18	0.301359235	0.793	0.665	4.57E-14
SMS	3.55E-31	0.301152121	0.871	0.792	6.71E-27
RDH11	1.07E-29	0.301044218	0.694	0.505	2.03E-25
PHLDA1	0.10462298	0.30081993	0.593	0.626	1
GIPC1	1.33E-24	0.300723661	0.817	0.732	2.51E-20
CDV3	4.24E-24	0.300101674	0.829	0.742	8.00E-20
HSPE1	6.38E-22	0.299938748	0.974	0.978	1.21E-17
TK1	8.57E-16	0.298741382	0.348	0.221	1.62E-11
SBSPON	7.64E-55	0.29774868	0.371	0.129	1.44E-50
SMIM3	4.88E-52	0.297213994	0.47	0.205	9.22E-48
EFNA1	3.37E-09	0.297166692	0.557	0.467	6.37E-05
PDCD10	4.88E-26	0.296854093	0.824	0.693	9.22E-22
PPP1R15A	5.94E-11	0.296287959	0.76	0.699	1.12E-06
ERBIN	1.23E-38	0.295525442	0.637	0.398	2.32E-34
CLINT1	1.35E-24	0.295061011	0.732	0.612	2.55E-20
KLK10	3.51E-16	0.295015888	0.28	0.156	6.64E-12
TM4SF20	1.58E-67	0.294696906	0.341	0.094	2.98E-63
GRINA	8.12E-36	0.294689294	0.671	0.466	1.53E-31
REEP5	4.16E-27	0.294680292	0.878	0.83	7.86E-23
GALC	5.66E-89	0.294493735	0.476	0.148	1.07E-84
CLIP1	1.21E-37	0.294304412	0.638	0.418	2.28E-33
CD151	1.37E-21	0.29416378	0.963	0.924	2.60E-17
ABHD12	9.05E-23	0.293932694	0.679	0.533	1.71E-18
XPOT	4.75E-30	0.293559248	0.566	0.372	8.96E-26
SNX3	1.10E-32	0.293209694	0.938	0.912	2.09E-28
SELENOP	6.18E-27	0.293098325	0.412	0.219	1.17E-22
RAB1A	2.05E-29	0.292629549	0.908	0.851	3.86E-25
KDELR2	4.36E-30	0.292093984	0.955	0.927	8.24E-26
ARRDC3	9.33E-06	0.291908487	0.449	0.387	0.176193723
PAK2	4.45E-29	0.291407194	0.813	0.7	8.40E-25
MAF1	6.89E-29	0.290613335	0.831	0.763	1.30E-24

PTPRR	2.16E-124	0.290233075	0.278	0.023	4.08E-120
NDUFB9	1.13E-27	0.289869346	0.955	0.967	2.14E-23
ITGA2	1.85E-06	0.289818139	0.612	0.555	0.034994476
C1GALT1	1.06E-29	0.289621887	0.737	0.556	2.01E-25
CORO2A	1.06E-47	0.289290563	0.558	0.297	1.99E-43
CTSZ	1.33E-19	0.288406634	0.732	0.607	2.52E-15
CCNG1	1.21E-17	0.288065696	0.854	0.812	2.28E-13
DPM1	1.31E-28	0.287982048	0.804	0.641	2.47E-24
SLC25A1	9.41E-31	0.286958967	0.673	0.473	1.78E-26
NOP10	7.72E-31	0.286448105	0.919	0.909	1.46E-26
PPIC	1.92E-15	0.286182318	0.863	0.789	3.62E-11
MUC13	0.031167213	0.286160107	0.808	0.808	1
CLIC4	5.75E-65	0.285673048	0.396	0.133	1.09E-60
COMT	4.14E-25	0.284043889	0.881	0.824	7.82E-21
MYDGF	1.74E-26	0.283165653	0.93	0.891	3.29E-22
ARPC5	1.70E-23	0.282944099	0.923	0.885	3.21E-19
TRAF4	3.44E-25	0.282866076	0.728	0.585	6.50E-21
KCNK6	2.06E-30	0.282843786	0.518	0.318	3.88E-26
PTK2	4.49E-36	0.282324171	0.687	0.476	8.48E-32
ENC1	6.54E-10	0.282244406	0.73	0.667	1.23E-05
PTGES3	1.91E-24	0.281911307	0.967	0.961	3.61E-20
TMED10	1.68E-30	0.281872523	0.907	0.854	3.18E-26
FAM83H	1.20E-35	0.28180137	0.619	0.402	2.26E-31
CLDN2	0.255619831	0.281800223	0.569	0.597	1
RIMKLA	2.86E-73	0.281794359	0.472	0.169	5.40E-69
LAMTOR5	2.51E-28	0.281160971	0.962	0.945	4.74E-24
CDCP1	1.89E-35	0.280841774	0.589	0.365	3.58E-31
MORF4L2	2.61E-28	0.280785665	0.893	0.806	4.93E-24
CYR61	1.32E-48	0.280721003	0.332	0.116	2.50E-44
RAB7A	2.65E-29	0.280691723	0.866	0.805	5.00E-25
CARD19	1.74E-29	0.280469022	0.593	0.392	3.29E-25
CTSA	2.81E-16	0.280378767	0.745	0.639	5.31E-12
RTN3	3.86E-23	0.2802451	0.864	0.798	7.28E-19
AC025580.3	2.50E-77	0.279977003	0.339	0.082	4.71E-73
TMEM41B	1.22E-26	0.279712021	0.614	0.437	2.30E-22
DHRS7	5.27E-15	0.27950983	0.818	0.808	9.95E-11
IL18	1.51E-19	0.279227896	0.541	0.374	2.85E-15
EPS8	3.72E-13	0.278825892	0.81	0.74	7.02E-09
CCL20	0.894364677	0.278708085	0.354	0.355	1
TSTA3	4.18E-26	0.278632164	0.871	0.8	7.89E-22
SARS	6.38E-17	0.278524391	0.814	0.74	1.21E-12
CORO1C	4.73E-44	0.278278923	0.591	0.334	8.93E-40
KDM3A	3.22E-33	0.27825133	0.43	0.222	6.08E-29

LYN	2.35E-39	0.277813998	0.604	0.37	4.43E-35
UQCRQ	1.85E-26	0.277562505	0.988	0.986	3.49E-22
CHD1	1.23E-26	0.27735482	0.709	0.552	2.32E-22
MZT2B	5.07E-31	0.276547723	0.966	0.964	9.57E-27
SHARPIN	5.63E-35	0.27630308	0.775	0.616	1.06E-30
EIF2AK2	1.76E-21	0.275830075	0.73	0.589	3.33E-17
MGAT4A	5.63E-43	0.27557927	0.551	0.302	1.06E-38
SLC26A2	2.73E-09	0.275122353	0.297	0.204	5.16E-05
NAA20	0.729947677	0.27511398	0.74	0.762	1
RABL6	6.57E-20	0.27503824	0.817	0.772	1.24E-15
SRSF9	4.12E-31	0.274875139	0.943	0.938	7.78E-27
NPEPPS	5.02E-19	0.274653637	0.702	0.583	9.48E-15
TM7SF2	3.63E-20	0.274618103	0.608	0.443	6.86E-16
PICALM	9.69E-32	0.273486687	0.56	0.343	1.83E-27
PKN2	7.60E-25	0.273257933	0.77	0.642	1.44E-20
RAC1	3.39E-24	0.272831841	0.999	0.986	6.40E-20
ARPC5L	5.06E-25	0.272558814	0.806	0.687	9.55E-21
GPBP1	1.72E-15	0.272418407	0.84	0.764	3.25E-11
PABPC1	2.03E-28	0.271992976	1	0.993	3.84E-24
TMEM97	1.45E-22	0.271854102	0.608	0.439	2.74E-18
MYH14	9.83E-20	0.271817902	0.789	0.705	1.86E-15
HMGB2	0.011825681	0.271684332	0.499	0.616	1
HSF1	4.31E-29	0.271375733	0.745	0.611	8.14E-25
FAM177A1	3.52E-16	0.27101496	0.924	0.904	6.64E-12
TPM1	4.31E-27	0.270742023	0.967	0.952	8.14E-23
CYC1	6.99E-19	0.270035144	0.953	0.953	1.32E-14
TUBA1B	0.037713353	0.269723425	0.877	0.896	1
ECE1	1.32E-49	0.269149777	0.411	0.171	2.50E-45
RHOA	2.29E-24	0.269055937	0.961	0.945	4.33E-20
SAR1A	7.23E-27	0.268771536	0.757	0.597	1.36E-22
PTP4A2	6.14E-23	0.268614696	0.925	0.896	1.16E-18
FLOT1	1.51E-22	0.268298059	0.82	0.719	2.86E-18
SPON1	6.90E-87	0.267846205	0.385	0.095	1.30E-82
PDK1	1.92E-90	0.267577095	0.409	0.106	3.62E-86
PPP1R16A	2.05E-22	0.266275517	0.801	0.685	3.87E-18
RALY	1.10E-20	0.265972996	0.905	0.861	2.08E-16
DEGS1	4.03E-55	0.265777386	0.407	0.159	7.61E-51
SLC38A2	3.90E-25	0.265579612	0.659	0.484	7.37E-21
THEM6	3.30E-36	0.265316381	0.611	0.384	6.22E-32
MTFR1	4.69E-35	0.26521897	0.608	0.38	8.86E-31
RHOD	2.27E-61	0.265121466	0.382	0.131	4.29E-57
TRMT112	2.64E-24	0.26485329	0.932	0.911	4.99E-20
CLTA	2.90E-34	0.264626838	0.976	0.956	5.47E-30

SELENOK	7.67E-13	0.263883848	0.856	0.81	1.45E-08
SCOC	1.55E-24	0.263761296	0.766	0.619	2.93E-20
CLCN3	1.67E-16	0.263728224	0.791	0.686	3.16E-12
STX3	4.98E-24	0.263613896	0.683	0.522	9.41E-20
KLF3	2.88E-11	0.263293391	0.782	0.758	5.43E-07
ADD1	5.74E-28	0.262944201	0.678	0.503	1.08E-23
RPL8	8.67E-39	0.262860509	1	0.999	1.64E-34
MRFAP1	3.82E-23	0.262799661	0.879	0.862	7.21E-19
CPD	2.20E-31	0.262445616	0.636	0.425	4.16E-27
VSNL1	2.89E-25	0.261917968	0.462	0.277	5.46E-21
HDHD3	3.83E-26	0.261796034	0.797	0.687	7.24E-22
OTULINL	1.14E-26	0.261655933	0.672	0.485	2.15E-22
CNN2	4.79E-26	0.261280195	0.762	0.601	9.05E-22
MICAL2	1.09E-48	0.261138744	0.473	0.219	2.06E-44
MAP2K1	2.80E-34	0.260920182	0.593	0.381	5.29E-30
TMOD3	3.73E-25	0.260059554	0.728	0.577	7.04E-21
ACOT7	1.61E-29	0.259611614	0.593	0.381	3.03E-25
MIDN	1.97E-16	0.258145859	0.722	0.618	3.72E-12
FABP2	1.46E-44	0.25788805	0.351	0.136	2.75E-40
TPMT	7.41E-20	0.257174298	0.728	0.618	1.40E-15
SLC25A24	9.45E-25	0.256497378	0.706	0.541	1.78E-20
TGFB1	1.30E-72	0.255898299	0.289	0.063	2.45E-68
GNA11	7.94E-27	0.25575985	0.496	0.31	1.50E-22
CYP3A5	1.81E-10	0.255502237	0.766	0.712	3.42E-06
CTSC	8.36E-31	0.255359463	0.595	0.355	1.58E-26
RAB10	2.69E-19	0.255169985	0.859	0.79	5.07E-15
PARD6B	6.14E-39	0.2551623	0.486	0.252	1.16E-34
PBDC1	4.34E-22	0.255013133	0.753	0.632	8.19E-18
TM9SF3	2.65E-23	0.254971668	0.939	0.921	5.00E-19
TGFBR2	3.29E-24	0.254900355	0.589	0.412	6.21E-20
TJP3	6.75E-23	0.254894036	0.664	0.5	1.27E-18
SC5D	9.68E-32	0.254773287	0.492	0.285	1.83E-27
CARHSP1	4.32E-15	0.254674643	0.833	0.766	8.15E-11
TAGLN2	7.50E-20	0.254508461	0.916	0.824	1.42E-15
LGALS3BP	3.30E-07	0.25412506	0.886	0.923	0.00623151
SAPCD2	1.25E-32	0.253082881	0.467	0.252	2.37E-28
SMIM22	1.55E-07	0.252947576	0.97	0.962	0.002923201
JUP	1.59E-13	0.252939901	0.778	0.702	2.99E-09
PAIP2	4.01E-23	0.2522763	0.832	0.746	7.57E-19
SH3RF1	1.59E-23	0.252236873	0.577	0.407	3.00E-19
GNAI3	4.98E-25	0.252161417	0.722	0.562	9.40E-21
MAP2K3	1.43E-19	0.252076024	0.581	0.435	2.70E-15
SERP1	2.55E-14	0.252019168	0.939	0.943	4.81E-10

SNRPG	2.09E-19	0.251725295	0.953	0.942	3.95E-15
MIR4435-2HG	2.28E-49	0.251707193	0.347	0.126	4.31E-45
LAD1	4.35E-21	0.251319006	0.836	0.759	8.20E-17
STARD4	4.34E-35	0.251227751	0.401	0.2	8.20E-31
CYP27A1	4.80E-54	0.251206002	0.411	0.159	9.06E-50
HSD17B11	8.52E-15	0.251087899	0.894	0.885	1.61E-10
AMN	9.44E-22	0.250985598	0.641	0.486	1.78E-17
ZNHIT1	1.01E-22	-0.250033398	0.894	0.935	1.91E-18
FAU	5.64E-53	-0.250388513	0.996	0.994	1.07E-48
MUC1	5.63E-36	-0.250713538	0.275	0.541	1.06E-31
MPLKIP	6.88E-24	-0.250883886	0.701	0.81	1.30E-19
ADTRP	1.64E-46	-0.251234061	0.049	0.304	3.09E-42
TMEM106B	1.14E-29	-0.251448947	0.545	0.708	2.16E-25
AKAP9	2.37E-17	-0.251844715	0.801	0.87	4.48E-13
AP003774.4	5.41E-49	-0.252581903	0.031	0.293	1.02E-44
NDUFV1	3.67E-22	-0.252784906	0.751	0.838	6.92E-18
MEST	3.93E-29	-0.252877855	0.467	0.647	7.43E-25
CD47	3.57E-20	-0.252904217	0.747	0.843	6.74E-16
TXNL1	5.00E-23	-0.252933136	0.74	0.827	9.44E-19
EIF3M	3.25E-24	-0.252982683	0.867	0.927	6.14E-20
EEPD1	4.59E-31	-0.253018012	0.202	0.415	8.66E-27
PIAS2	2.52E-29	-0.253049754	0.253	0.446	4.77E-25
TMEM173	6.09E-43	-0.253450866	0.103	0.362	1.15E-38
CHMP2A	1.82E-24	-0.253487936	0.816	0.875	3.43E-20
GLUL	1.92E-14	-0.253554163	0.762	0.835	3.62E-10
TMC8	1.64E-42	-0.254946534	0.051	0.293	3.10E-38
CKMT1A	5.62E-26	-0.255160843	0.224	0.405	1.06E-21
BUD23	5.14E-23	-0.257357702	0.728	0.84	9.71E-19
SYNGR2	8.13E-21	-0.257489908	0.81	0.89	1.54E-16
SCNN1A	5.37E-45	-0.257532365	0.112	0.381	1.01E-40
RPL36	3.25E-42	-0.257665385	1	0.998	6.13E-38
RPS25	2.77E-46	-0.257702298	1	0.995	5.22E-42
DUS1L	7.48E-27	-0.258060753	0.432	0.607	1.41E-22
VPS36	1.07E-25	-0.258365865	0.602	0.737	2.01E-21
UBXN1	5.11E-20	-0.258671239	0.852	0.895	9.64E-16
JUN	1.08E-11	-0.259785451	0.969	0.973	2.03E-07
TFCP2L1	1.77E-53	-0.260867416	0.026	0.303	3.34E-49
TSPAN12	2.14E-28	-0.261234394	0.294	0.494	4.04E-24
HOXA11-AS	5.16E-30	-0.261529096	0.272	0.472	9.74E-26
TXN2	3.36E-25	-0.261599429	0.741	0.836	6.35E-21
NSD3	4.25E-26	-0.261834934	0.505	0.666	8.03E-22
RPS24	2.57E-53	-0.26262339	1	0.997	4.86E-49
ECSIT	5.28E-33	-0.262627932	0.405	0.602	9.96E-29

FKBP3	5.53E-24	-0.262697023	0.734	0.844	1.04E-19
CKMT1B	7.44E-30	-0.263007735	0.248	0.445	1.40E-25
PRCP	4.38E-40	-0.263734526	0.138	0.379	8.28E-36
MRPS7	3.45E-25	-0.264602749	0.633	0.761	6.52E-21
FBL	3.00E-22	-0.264664156	0.822	0.915	5.66E-18
RPL35A	1.62E-49	-0.265124468	0.999	0.996	3.07E-45
TXNL4A	1.29E-25	-0.265157169	0.665	0.765	2.43E-21
PEBP1	3.38E-25	-0.265778489	0.924	0.974	6.39E-21
LSM7	1.75E-27	-0.266104379	0.916	0.956	3.30E-23
COX4I1	1.52E-53	-0.266602285	0.996	0.994	2.87E-49
NDUFB2	1.95E-25	-0.266616335	0.961	0.976	3.68E-21
RPAIN	8.14E-31	-0.26743283	0.535	0.702	1.54E-26
KLHDC2	4.60E-21	-0.267569191	0.668	0.77	8.68E-17
FAM96A	2.54E-27	-0.268105042	0.625	0.765	4.79E-23
ETS2	2.44E-21	-0.268451398	0.901	0.939	4.60E-17
NDUFAF8	7.08E-23	-0.268892615	0.641	0.785	1.34E-18
GLOD4	5.03E-30	-0.269291789	0.507	0.671	9.50E-26
CASP1	2.48E-14	-0.27008964	0.383	0.5	4.69E-10
ATP8B1	1.44E-25	-0.270738039	0.611	0.742	2.72E-21
CYBA	6.57E-23	-0.271315189	0.931	0.969	1.24E-18
ZNRF3	5.57E-32	-0.271334227	0.268	0.487	1.05E-27
NCOR1	1.05E-23	-0.27213301	0.729	0.836	1.99E-19
RPL18	2.63E-55	-0.274539849	1	0.996	4.96E-51
APEX1	3.78E-24	-0.274894517	0.782	0.9	7.14E-20
RPL24	1.03E-49	-0.275154062	0.993	0.994	1.95E-45
ECH1	1.16E-24	-0.275459171	0.809	0.9	2.19E-20
TIMM13	1.80E-23	-0.275466902	0.875	0.954	3.41E-19
LINC00926	2.09E-55	-0.275872751	0.035	0.319	3.94E-51
PTEN	2.01E-35	-0.276110462	0.356	0.58	3.79E-31
CCDC25	2.24E-36	-0.276360919	0.396	0.596	4.22E-32
DLD	8.55E-26	-0.276595154	0.591	0.728	1.61E-21
MT1E	6.71E-07	-0.277004982	0.207	0.287	0.012674656
ABO	4.02E-40	-0.277538426	0.237	0.487	7.59E-36
RPL18A	8.56E-50	-0.27790115	1	0.997	1.62E-45
LSM5	1.09E-23	-0.278204668	0.827	0.916	2.06E-19
PSMA2	5.40E-30	-0.279233576	0.912	0.946	1.02E-25
TPT1	1.11E-34	-0.279370396	0.997	0.998	2.10E-30
CNDP2	9.02E-30	-0.279591785	0.459	0.636	1.70E-25
ALDH2	7.96E-16	-0.279631384	0.877	0.926	1.50E-11
MRPL32	2.55E-33	-0.280382597	0.696	0.827	4.81E-29
HIBCH	1.89E-43	-0.280513721	0.209	0.466	3.56E-39
ALDH3A2	1.24E-31	-0.280821132	0.405	0.595	2.34E-27
PFN2	2.57E-53	-0.281456755	0.034	0.307	4.85E-49

ICA1	4.76E-28	-0.282155884	0.472	0.645	9.00E-24
COX5A	7.47E-29	-0.283584359	0.959	0.98	1.41E-24
HOXB6	6.88E-29	-0.2839554	0.389	0.571	1.30E-24
CNOT7	2.46E-35	-0.284363765	0.534	0.712	4.65E-31
DNAJC15	2.39E-24	-0.284673037	0.898	0.947	4.51E-20
RPL11	4.92E-65	-0.285813944	1	0.998	9.30E-61
NOB1	1.44E-28	-0.287333054	0.618	0.774	2.72E-24
ATP5PB	8.74E-31	-0.287801229	0.92	0.952	1.65E-26
NARS	3.45E-29	-0.287840091	0.671	0.774	6.51E-25
SEMA5A	2.79E-19	-0.288107788	0.454	0.596	5.27E-15
FMC1	4.11E-33	-0.288212489	0.476	0.657	7.77E-29
HNMT	4.64E-27	-0.289955332	0.541	0.696	8.76E-23
RPL41	6.90E-66	-0.290090874	1	0.999	1.30E-61
ABHD11	2.82E-25	-0.291799697	0.642	0.755	5.32E-21
RPS7	1.12E-53	-0.292262708	1	0.995	2.11E-49
RPS16	2.74E-46	-0.292826542	0.997	0.996	5.18E-42
VDAC3	1.65E-34	-0.293056465	0.627	0.776	3.11E-30
NPM3	5.73E-31	-0.29315567	0.503	0.68	1.08E-26
SOD2	6.48E-24	-0.293387745	0.542	0.672	1.22E-19
SLC44A3	2.84E-30	-0.29354209	0.518	0.678	5.36E-26
APRT	8.18E-32	-0.29433221	0.885	0.961	1.54E-27
RPL14	1.12E-54	-0.295562628	0.999	0.995	2.12E-50
FOXP1	2.21E-23	-0.296052687	0.698	0.814	4.18E-19
ACAA2	1.64E-25	-0.296425399	0.717	0.819	3.10E-21
TMEM147	2.85E-27	-0.297263164	0.825	0.913	5.39E-23
NHLRC3	5.00E-35	-0.297509195	0.343	0.568	9.44E-31
FIS1	2.42E-27	-0.297674853	0.897	0.931	4.57E-23
CDHR1	4.65E-36	-0.298190177	0.201	0.43	8.78E-32
VPS51	1.43E-29	-0.298747576	0.652	0.785	2.70E-25
SUCLG2	5.09E-29	-0.299420045	0.778	0.874	9.61E-25
RPL13A	5.32E-63	-0.300468618	1	0.999	1.00E-58
MRPL11	1.07E-33	-0.301381285	0.663	0.823	2.01E-29
UPF3A	5.57E-32	-0.301584534	0.595	0.745	1.05E-27
NOXO1	5.22E-39	-0.301698167	0.325	0.573	9.86E-35
SLC40A1	8.58E-22	-0.302329063	0.68	0.789	1.62E-17
HMGCS2	3.02E-13	-0.303143433	0.344	0.467	5.70E-09
PRDX3	2.00E-31	-0.303823743	0.738	0.853	3.78E-27
CLRN3	5.23E-27	-0.304164887	0.45	0.606	9.87E-23
PLCB4	1.52E-25	-0.304744714	0.55	0.707	2.86E-21
HOXA7	3.82E-39	-0.306373622	0.249	0.487	7.20E-35
SEMA3C	6.05E-33	-0.306944599	0.411	0.609	1.14E-28
MRPL12	5.67E-26	-0.307404264	0.718	0.857	1.07E-21
PHF14	3.32E-29	-0.308723435	0.732	0.849	6.26E-25

HDDC2	5.13E-32	-0.309622319	0.656	0.784	9.68E-28
HLA-B	4.38E-22	-0.31227097	0.986	0.984	8.28E-18
GABARAP	4.00E-35	-0.312437482	0.572	0.735	7.56E-31
CIRBP	1.98E-27	-0.314206899	0.879	0.957	3.73E-23
IGBP1	5.16E-28	-0.31495685	0.732	0.838	9.75E-24
GALNT6	3.54E-38	-0.315257828	0.283	0.518	6.68E-34
RPL31	1.48E-56	-0.315873754	1	0.996	2.79E-52
NDUFB7	6.25E-38	-0.316470445	0.904	0.959	1.18E-33
CD44	2.34E-27	-0.316582246	0.82	0.92	4.42E-23
TP53TG1	4.35E-46	-0.316718501	0.255	0.514	8.21E-42
EIF2A	2.07E-31	-0.318580376	0.694	0.816	3.90E-27
ATP5MC2	5.92E-46	-0.319880014	0.98	0.983	1.12E-41
DECR1	5.68E-35	-0.320813986	0.696	0.825	1.07E-30
DEFA5	2.33E-14	-0.321085475	0.16	0.284	4.40E-10
HSD11B2	9.35E-34	-0.322235245	0.314	0.523	1.77E-29
SAMD5	1.32E-39	-0.322722069	0.306	0.536	2.49E-35
SNRPN	2.57E-61	-0.323493929	0.009	0.306	4.84E-57
MRPL4	5.34E-38	-0.324399225	0.549	0.739	1.01E-33
HIPK2	1.51E-34	-0.325087589	0.367	0.568	2.85E-30
EIF3G	4.40E-36	-0.326032494	0.827	0.928	8.31E-32
SULT1B1	5.41E-48	-0.326417755	0.159	0.446	1.02E-43
SRPK1	4.02E-25	-0.326703319	0.798	0.887	7.59E-21
IGHA1	2.44E-06	-0.327098542	0.576	0.581	0.046087391
CAT	1.42E-36	-0.32836742	0.5	0.702	2.68E-32
PSMB9	7.59E-24	-0.328725464	0.432	0.596	1.43E-19
SMDT1	9.54E-39	-0.328955801	0.835	0.913	1.80E-34
RPL23A	1.24E-67	-0.33031532	0.997	0.995	2.34E-63
WNK2	2.45E-49	-0.330591007	0.236	0.519	4.63E-45
PCM1	7.91E-40	-0.332132228	0.488	0.704	1.49E-35
NTHL1	1.42E-42	-0.332417768	0.443	0.665	2.68E-38
AIFM3	3.12E-67	-0.332623402	0.057	0.387	5.89E-63
DHRS4L2	4.16E-48	-0.333243802	0.377	0.615	7.86E-44
VSIG2	6.27E-46	-0.333332498	0.161	0.456	1.18E-41
ACADM	5.60E-50	-0.3345089	0.282	0.566	1.06E-45
RPL32	1.24E-80	-0.334730995	0.999	0.998	2.34E-76
EIF4A1	1.94E-28	-0.335669967	0.774	0.856	3.66E-24
PHB2	3.26E-39	-0.33760215	0.76	0.88	6.15E-35
ARGLU1	1.18E-31	-0.33800451	0.793	0.895	2.23E-27
SRI	2.41E-30	-0.338174877	0.962	0.97	4.55E-26
RPL23	1.20E-50	-0.338613251	0.981	0.991	2.27E-46
PLPP2	7.06E-37	-0.339104015	0.778	0.899	1.33E-32
PSMB8	2.39E-32	-0.339446084	0.768	0.87	4.52E-28
PNISR	2.88E-32	-0.341157003	0.829	0.906	5.44E-28

TMEM9	3.85E-34	-0.341244559	0.435	0.633	7.26E-30
TTC19	1.73E-42	-0.342085282	0.519	0.699	3.27E-38
PTMS	4.91E-25	-0.343190495	0.699	0.823	9.26E-21
EEF1G	2.42E-38	-0.343275995	0.61	0.764	4.57E-34
RPS3	1.32E-59	-0.346902637	0.999	0.998	2.49E-55
ATOH8	8.43E-59	-0.346981593	0.033	0.333	1.59E-54
S100A13	2.48E-42	-0.348369519	0.447	0.67	4.68E-38
NOTCH1	1.47E-57	-0.349513224	0.182	0.497	2.78E-53
RBMX	2.44E-37	-0.350510997	0.798	0.894	4.61E-33
CCT3	3.84E-40	-0.350825582	0.755	0.893	7.26E-36
TOMM7	5.24E-50	-0.351102	0.947	0.969	9.89E-46
FARP1	7.95E-73	-0.352680997	0.026	0.377	1.50E-68
UXT	2.88E-42	-0.352867154	0.883	0.93	5.43E-38
RPL27A	1.19E-73	-0.355159397	1	0.995	2.25E-69
YWHAE	1.27E-42	-0.356196933	0.949	0.971	2.39E-38
MUC4	8.57E-62	-0.357645555	0.1	0.448	1.62E-57
GTF2I	6.15E-35	-0.357923984	0.762	0.891	1.16E-30
RPS8	1.31E-73	-0.360578996	0.993	0.996	2.47E-69
MSRB2	1.04E-32	-0.36101336	0.748	0.868	1.97E-28
RPL10	6.56E-77	-0.361720396	1	0.998	1.24E-72
ITPA	1.01E-46	-0.362207868	0.485	0.693	1.90E-42
RPS27	1.98E-67	-0.365810145	1	0.998	3.74E-63
ASAH1	1.04E-41	-0.366095848	0.595	0.758	1.96E-37
PMPCB	1.04E-44	-0.36683798	0.553	0.74	1.97E-40
CYCS	2.63E-32	-0.36749654	0.92	0.957	4.97E-28
RPL12	9.88E-71	-0.367863995	0.999	0.998	1.87E-66
EEF2	2.13E-49	-0.367897517	0.98	0.988	4.02E-45
ID1	2.39E-20	-0.369195012	0.879	0.939	4.51E-16
RPL4	2.66E-56	-0.369310684	0.989	0.991	5.01E-52
CFTR	7.93E-47	-0.370356312	0.405	0.666	1.50E-42
RPS15A	5.35E-81	-0.372308288	0.999	0.996	1.01E-76
PFDN5	1.19E-69	-0.372770927	0.985	0.984	2.25E-65
CYP4X1	4.51E-51	-0.372967699	0.134	0.425	8.53E-47
SARAF	1.94E-41	-0.374520007	0.841	0.907	3.66E-37
EIF3D	1.01E-37	-0.375490515	0.846	0.934	1.90E-33
RPS23	9.98E-78	-0.3767269	1	0.995	1.88E-73
RPS27A	4.97E-95	-0.377515252	0.997	0.995	9.38E-91
VAMP2	5.24E-51	-0.383395815	0.388	0.641	9.89E-47
EPHB2	4.07E-42	-0.383878124	0.622	0.826	7.69E-38
BCL11A	8.61E-42	-0.383945199	0.404	0.637	1.63E-37
NPDC1	1.61E-32	-0.384878745	0.909	0.926	3.05E-28
NQO1	1.24E-27	-0.385441259	0.737	0.855	2.33E-23
ZNF655	4.65E-80	-0.385725149	0.066	0.439	8.78E-76

MDH2	6.92E-47	-0.389017881	0.902	0.956	1.31E-42
EIF3K	8.60E-56	-0.389505879	0.953	0.973	1.62E-51
COA3	3.30E-48	-0.390389226	0.828	0.921	6.23E-44
CUTA	3.99E-51	-0.392678409	0.883	0.952	7.54E-47
RACK1	1.23E-71	-0.393945564	0.997	0.995	2.33E-67
TSTD1	1.09E-44	-0.394345972	0.84	0.92	2.06E-40
RPL19	9.73E-103	-0.397740318	1	0.998	1.84E-98
RPS18	1.16E-77	-0.397905297	1	0.999	2.18E-73
RPS2	2.83E-64	-0.39996647	0.999	0.998	5.35E-60
EPHX2	2.11E-69	-0.402525763	0.169	0.507	3.98E-65
RPLP0	3.03E-83	-0.40271096	0.996	0.996	5.72E-79
MT-ND5	3.81E-23	-0.403122159	0.961	0.976	7.19E-19
CTSS	4.17E-36	-0.405340277	0.659	0.796	7.88E-32
RPL6	9.57E-79	-0.406148951	0.995	0.996	1.81E-74
RPS17	1.44E-75	-0.406968073	0.986	0.988	2.72E-71
MYB	4.39E-51	-0.407023604	0.499	0.74	8.28E-47
RPSA	1.55E-65	-0.407452243	0.984	0.992	2.92E-61
IFT57	1.71E-50	-0.407657093	0.486	0.715	3.22E-46
THRA	3.35E-54	-0.415223721	0.34	0.608	6.33E-50
CD99	2.34E-39	-0.417042963	0.856	0.922	4.43E-35
RPL7A	2.18E-96	-0.417834628	0.999	0.995	4.11E-92
MUC3A	4.54E-41	-0.41870286	0.535	0.718	8.58E-37
AGR3	1.19E-36	-0.420689831	0.776	0.892	2.25E-32
LGR5	1.09E-44	-0.421241749	0.168	0.444	2.06E-40
AC103702.2	8.33E-42	-0.42129092	0.592	0.752	1.57E-37
HTATIP2	4.24E-49	-0.421502652	0.694	0.833	8.00E-45
ZKSCAN1	4.74E-35	-0.422751275	0.748	0.86	8.95E-31
LAMTOR4	1.11E-48	-0.424148324	0.904	0.95	2.10E-44
TUFM	7.58E-54	-0.424258098	0.878	0.948	1.43E-49
N4BP2L2	1.71E-41	-0.425034894	0.793	0.909	3.23E-37
TRAPPC1	1.54E-53	-0.429253006	0.686	0.841	2.90E-49
HOXA9	1.08E-46	-0.431736905	0.453	0.676	2.04E-42
PHGR1	1.51E-30	-0.432239068	0.953	0.985	2.86E-26
FABP5	6.98E-14	-0.432329526	0.546	0.663	1.32E-09
ATP5F1D	4.42E-67	-0.433916395	0.954	0.979	8.35E-63
PSMB6	2.79E-46	-0.434365236	0.782	0.883	5.27E-42
SLC12A2	2.89E-44	-0.435924985	0.939	0.971	5.47E-40
LDLRAD4	1.15E-72	-0.436840171	0.077	0.43	2.17E-68
EIF3F	9.45E-61	-0.437126381	0.889	0.956	1.78E-56
UBB	1.47E-64	-0.439041987	0.974	0.988	2.78E-60
ACADVL	8.24E-41	-0.441680981	0.767	0.863	1.56E-36
C9orf152	1.87E-65	-0.442320226	0.35	0.652	3.53E-61
PROX1	7.78E-63	-0.442991467	0.119	0.464	1.47E-58

DMAC1	1.31E-62	-0.44402681	0.71	0.878	2.48E-58
RAB13	6.69E-60	-0.444723877	0.654	0.839	1.26E-55
SLC39A8	1.61E-78	-0.444755344	0.169	0.536	3.04E-74
RPS3A	2.80E-102	-0.445070741	0.996	0.996	5.29E-98
SOD3	1.49E-19	-0.447751911	0.168	0.327	2.81E-15
RUBCNL	4.00E-72	-0.448779417	0.138	0.489	7.55E-68
HSPA1A	1.30E-43	-0.449600124	0.104	0.373	2.45E-39
QARS	1.51E-64	-0.452135552	0.736	0.885	2.86E-60
SMIM19	3.25E-70	-0.452867133	0.415	0.705	6.13E-66
NDUFA5	2.55E-57	-0.456413525	0.767	0.901	4.81E-53
MLXIP	1.42E-53	-0.460051632	0.728	0.872	2.68E-49
RPL5	5.39E-102	-0.462357544	0.996	0.993	1.02E-97
ZNF703	3.25E-44	-0.46263377	0.606	0.776	6.14E-40
NIPSNAP2	3.06E-66	-0.463841668	0.547	0.795	5.79E-62
S100P	1.15E-36	-0.464093029	0.398	0.664	2.16E-32
HNRNPA1	4.46E-70	-0.466469351	0.984	0.991	8.42E-66
RPL9	7.26E-109	-0.467011138	0.992	0.995	1.37E-104
ZBTB38	1.11E-59	-0.47382196	0.485	0.725	2.11E-55
EEF1A1	4.27E-106	-0.474033039	0.999	0.997	8.07E-102
DAB2	4.58E-42	-0.476531788	0.366	0.584	8.64E-38
RPL15	3.07E-127	-0.483296895	0.999	0.996	5.80E-123
PRR15	5.01E-30	-0.488199997	0.65	0.763	9.45E-26
RPS13	3.00E-115	-0.491354852	0.992	0.994	5.67E-111
SATB2	2.27E-81	-0.491745326	0.186	0.545	4.29E-77
CDCA7	1.68E-45	-0.492686984	0.504	0.733	3.17E-41
ID2	3.43E-38	-0.493358459	0.725	0.854	6.47E-34
ALDH1B1	1.26E-64	-0.496488609	0.389	0.689	2.38E-60
SLC25A6	3.79E-85	-0.498700603	0.982	0.989	7.16E-81
RAB11FIP1	4.55E-56	-0.504355212	0.631	0.813	8.60E-52
ALDH1A1	2.51E-58	-0.50438386	0.255	0.583	4.74E-54
EBPL	7.99E-74	-0.505606424	0.71	0.894	1.51E-69
MRPS33	1.00E-71	-0.511430805	0.77	0.917	1.89E-67
RPL34	5.48E-113	-0.51294199	1	0.997	1.03E-108
NAA38	6.12E-67	-0.515539622	0.682	0.846	1.16E-62
ATP5MC1	8.05E-54	-0.516294422	0.812	0.939	1.52E-49
IGKC	2.42E-12	-0.517243454	0.656	0.676	4.57E-08
RPL3	6.63E-116	-0.519203121	0.997	0.998	1.25E-111
CXCL2	3.31E-13	-0.519554933	0.339	0.464	6.24E-09
AC020916.1	1.34E-41	-0.520603877	0.324	0.544	2.54E-37
TESC	3.90E-77	-0.525557861	0.046	0.428	7.37E-73
RPS9	1.06E-139	-0.526896181	0.999	0.996	2.01E-135
SMAD9	1.74E-78	-0.529179908	0.098	0.471	3.28E-74
ESD	5.44E-76	-0.532611965	0.774	0.915	1.03E-71

RPL13	6.34E-123	-0.534504983	1	0.999	1.20E-118
HOXB9	4.60E-79	-0.534898314	0.392	0.705	8.68E-75
FOSB	3.45E-37	-0.538728809	0.618	0.782	6.51E-33
RPS10	4.59E-105	-0.540492289	0.978	0.989	8.67E-101
RAB32	8.31E-86	-0.540839746	0.191	0.587	1.57E-81
MT-ND1	7.35E-73	-0.543989954	0.976	0.988	1.39E-68
NDUFA4	1.76E-95	-0.550209729	0.98	0.987	3.32E-91
TCEA3	2.30E-89	-0.552565346	0.146	0.571	4.35E-85
KIAA1324	9.37E-81	-0.557927646	0.348	0.725	1.77E-76
MUC5B	5.92E-101	-0.558705991	0.022	0.454	1.12E-96
ITGA6	1.35E-51	-0.564787226	0.885	0.928	2.55E-47
CHCHD10	6.63E-73	-0.57017664	0.92	0.959	1.25E-68
PYCARD	2.26E-71	-0.571160898	0.5	0.775	4.26E-67
GSTK1	2.53E-76	-0.571507053	0.86	0.938	4.77E-72
RPL10A	6.65E-146	-0.574363493	1	0.996	1.26E-141
RPS12	1.94E-134	-0.57458512	1	0.998	3.67E-130
RPL22L1	9.93E-48	-0.576032037	0.738	0.876	1.88E-43
MT-ND4	5.28E-82	-0.588527394	0.988	0.994	9.97E-78
TNRC6B	7.46E-95	-0.589276202	0.236	0.645	1.41E-90
CDX2	3.27E-66	-0.594388815	0.678	0.884	6.17E-62
ZFP36L2	1.26E-39	-0.599318022	0.894	0.957	2.39E-35
ITPR2	4.51E-52	-0.600264618	0.267	0.553	8.51E-48
COMMD6	1.29E-88	-0.600410254	0.938	0.975	2.44E-84
KRTCAP3	5.37E-91	-0.603411681	0.724	0.889	1.01E-86
TMEM230	1.26E-82	-0.603785469	0.692	0.859	2.38E-78
LCN2	2.23E-22	-0.61819135	0.736	0.837	4.21E-18
RPL36A	3.78E-103	-0.619185997	0.985	0.992	7.13E-99
RPL21	2.96E-151	-0.62435619	1	0.996	5.59E-147
SMOC2	2.15E-52	-0.628107687	0.003	0.259	4.05E-48
MT-CO1	2.33E-82	-0.63328409	0.992	0.993	4.39E-78
RNF43	2.78E-77	-0.646649171	0.736	0.903	5.25E-73
RPS6	8.37E-167	-0.652758282	0.999	0.996	1.58E-162
EIF3L	3.34E-104	-0.668397596	0.852	0.945	6.30E-100
C1QBP	1.36E-97	-0.676077186	0.793	0.935	2.57E-93
TXNIP	8.46E-70	-0.676879285	0.389	0.723	1.60E-65
HES1	6.77E-33	-0.695982507	0.774	0.846	1.28E-28
AXIN2	9.93E-86	-0.702039383	0.546	0.818	1.88E-81
GAS6	6.03E-89	-0.703301133	0.508	0.79	1.14E-84
MT-ND3	7.02E-76	-0.704557891	0.985	0.988	1.33E-71
RGMB	5.82E-81	-0.715556295	0.198	0.559	1.10E-76
NOP53	2.16E-97	-0.724207656	0.927	0.972	4.08E-93
APIP	5.19E-94	-0.732117787	0.463	0.756	9.81E-90
EPHB3	5.25E-96	-0.73438874	0.26	0.656	9.92E-92

IMPDH2	8.49E-117	-0.759221439	0.776	0.932	1.60E-112
RPS4X	6.02E-162	-0.771280712	0.997	0.994	1.14E-157
GPX2	4.75E-101	-0.781249683	0.928	0.975	8.96E-97
MUC12	1.54E-61	-0.791926276	0.486	0.726	2.91E-57
QTRT1	1.74E-132	-0.796984688	0.495	0.861	3.29E-128
CD74	1.10E-31	-0.800634779	0.516	0.679	2.08E-27
SPINK4	2.44E-11	-0.82315332	0.153	0.259	4.62E-07
ADH1C	1.65E-71	-0.824058405	0.164	0.509	3.11E-67
CCND2	2.55E-119	-0.842280798	0.28	0.736	4.82E-115
ATP5F1A	6.02E-149	-0.844676465	0.814	0.948	1.14E-144
GOLIM4	2.59E-96	-0.863705527	0.753	0.929	4.89E-92
MT-ATP6	1.58E-125	-0.880395108	0.977	0.988	2.99E-121
MT-CO2	1.27E-128	-0.894767133	0.986	0.993	2.40E-124
PRDX5	4.15E-132	-0.916418613	0.974	0.986	7.84E-128
RPS5	2.55E-166	-0.923262605	0.986	0.99	4.82E-162
ID4	9.58E-124	-0.924338031	0.024	0.527	1.81E-119
REPIN1	1.62E-135	-0.926309773	0.618	0.879	3.06E-131
RPL17	1.75E-151	-0.926385783	0.907	0.967	3.30E-147
BTG2	2.92E-97	-0.967537768	0.608	0.854	5.51E-93
MT-ND2	8.66E-126	-0.974767395	0.988	0.989	1.64E-121
MT-CYB	2.30E-119	-0.97726873	0.977	0.99	4.35E-115
FAM3D	4.86E-109	-1.00764722	0.852	0.927	9.17E-105
NKD1	4.94E-87	-1.014800369	0.187	0.586	9.32E-83
CD9	5.77E-127	-1.018475492	0.928	0.973	1.09E-122
L1TD1	4.88E-129	-1.045070637	0.02	0.534	9.22E-125
FERMT1	7.89E-146	-1.113644243	0.47	0.819	1.49E-141
RPL26	5.40E-203	-1.1148081	0.995	0.996	1.02E-198
C10orf99	6.61E-140	-1.230225844	0.497	0.832	1.25E-135
ASCL2	1.36E-128	-1.260095217	0.702	0.925	2.58E-124
MT-CO3	4.36E-201	-1.342292531	0.993	0.993	8.23E-197
DPEP1	1.01E-123	-1.4638558	0.255	0.688	1.91E-119
APCDD1	1.87E-145	-1.555702606	0.001	0.557	3.54E-141
ID3	3.96E-110	-1.655563853	0.642	0.859	7.47E-106
PCCA	4.51E-158	-1.812798618	0.217	0.714	8.52E-154
LEFTY1	3.31E-162	-1.996935821	0.163	0.725	6.25E-158
PIGR	2.40E-23	-2.049288861	0.167	0.338	4.53E-19
SELENBP1	2.45E-187	-2.504035967	0.58	0.891	4.63E-183
REG1A	3.09E-83	-3.44141078	0.022	0.398	5.83E-79
